# Weakly Positioned Nucleosomes Enhance the Transcriptional Competency of Chromatin

**DOI:** 10.1371/journal.pone.0012984

**Published:** 2010-09-24

**Authors:** Yaakov Belch, Jingyi Yang, Yang Liu, Sridhar A. Malkaram, Rong Liu, Jean-Jack M. Riethoven, Istvan Ladunga

**Affiliations:** 1 Department of Statistics, University of Nebraska-Lincoln, Lincoln, Nebraska, United States of America; 2 Department of Computer Science and Engineering, University of Nebraska-Lincoln, Lincoln, Nebraska, United States of America; 3 Center for Biotechnology, University of Nebraska-Lincoln, Lincoln, Nebraska, United States of America; 4 School of Biological Sciences, University of Nebraska-Lincoln, Lincoln, Nebraska, United States of America; University of Leuven, Belgium

## Abstract

**Background:**

Transcription is affected by nucleosomal resistance against polymerase passage. In turn, nucleosomal resistance is determined by DNA sequence, histone chaperones and remodeling enzymes. The contributions of these factors are widely debated: one recent title claims “… DNA-encoded nucleosome organization…” while another title states that “histone-DNA interactions are not the major determinant of nucleosome positions.” These opposing conclusions were drawn from similar experiments analyzed by idealized methods. We attempt to resolve this controversy to reveal nucleosomal competency for transcription.

**Methodology/Principal Findings:**

To this end, we analyzed 26 in vivo, nonlinked, and in vitro genome-wide nucleosome maps/replicates by new, rigorous methods. Individual H2A nucleosomes are reconstituted inaccurately by transcription, chaperones and remodeling enzymes. At gene centers, weakly positioned nucleosome arrays facilitate rapid histone eviction and remodeling, easing polymerase passage. Fuzzy positioning is not due to artefacts. At the regional level, transcriptional competency is strongly influenced by intrinsic histone-DNA affinities. This is confirmed by reproducing the high in vivo occupancy of translated regions and the low occupancy of intergenic regions in reconstitutions from purified DNA and histones. Regional level occupancy patterns are protected from invading histones by nucleosome excluding sequences and barrier nucleosomes at gene boundaries and within genes.

**Conclusions/Significance:**

Dense arrays of weakly positioned nucleosomes appear to be necessary for transcription. Weak positioning at exons facilitates temporary remodeling, polymerase passage and hence the competency for transcription. At regional levels, the DNA sequence plays a major role in determining these features but positions of individual nucleosomes are typically modified by transcription, chaperones and enzymes. This competency is reduced at intergenic regions by sequence features, barrier nucleosomes, and proteins, preventing accessibility regulation of untargeted genes. This combination of DNA- and protein-influenced positioning regulates DNA accessibility and competence for regulatory protein binding and transcription. Interactive nucleosome displays are offered at http://chromatin.unl.edu/cgi-bin/skyline.cgi.

## Introduction

Nucleosomal resistance against RNA polymerase II (Pol II)-induced remodeling and eviction may regulate the speed of transcription. What determines the strength of nucleosome positions is a subject to intense debate. Fundamentally “*DNA-encoded nucleosome organization*” is advocated by Kaplan *et al.* [1] in the Segal, Widom and Lieb laboratories, while “*intrinsic histone-DNA interactions*” are considered only as minor determinants of the in vivo nucleosome positions by Zhang *et al.* [2] in Struhl and Liu's laboratories. Most notably, Kaplan *et al.* [1] strongly correlated in vitro reconstruction with the five base pair (bp) sequence preferences of nucleosomes (*r* = 0.83). Zhang *et al.* [2] estimated that intrinsic histone-DNA interactions account for only ∼20% of the *in vivo* positions because “nucleosomes assembled in vitro have only limited preference for specific translational positions and do not show the patterns observed in vivo.” These opposing conclusions were drawn from compatible experiments but incompatible data analysis. These experiments shared an identical concept: reconstitute nucleosomes in vitro from purified chicken or *Drosophila* histones and yeast DNA. The differences were limited to experimental implementations and interpretations. As Zhang et al. pointed out, their group used a nonlimiting histone∶DNA ratio of 1∶1 to simulate the high in vivo nucleosome occupancy, while Kaplan et al. used a lower ratio of 0.4∶1 that allowed the limiting histones to occupy the highest affinity DNA loci and leave many less affine in vivo loci vacant. Because higher affinity reduces the probability of inaccurate remodeling, this competition inflated the correlation between in vivo and in vitro nucleosome occupancy (“histone density”, from 0.54 in Zhang et al. to 0.74 in Kaplan et al.).

This debate resurfaced on Correspondence pages of *Nature Structural and Molecular Biology*. Kaplan et al. [3] now published numerical estimates for the DNA's influence on nucleosome positioning. Depending on the methods and parameter values used, this ranges as wide as 34–57%. In their reply, Zhang et al. [4], and in a separate comment, Franklin Pugh [5] raised objections against using nucleosome occupancy to estimate nucleosome positioning. Zhang et al. [4] reiterated that as few as “∼20% of the in vivo positioned nucleosomes are positioned due to intrinsic histone-DNA interactions.” The most notable progress is some departure from the “code” concept for nucleosome positioning: now Kaplan et al. [3] “leave for others to debate” whether the influence of DNA on many aspects of the in vivo nucleosome organization reflects the use of a *code*.”

By partially resolving this controversy, we aimed to improve our understanding of the chromatin's competency for transcription. In doing so, we carefully avoided *directly* estimating the influence of intrinsic histone-DNA affinities on nucleosome positioning because of their extreme sensitivity for the choice of methods and parameter values. In a more robust approach, we compared nucleosome occupancy and dynamics patterns between different gene and genomic regions (GGRs). We observed similar occupancy and dynamism patterns both in vivo or in vitro under diverse conditions across twenty six high-coverage maps of nucleosomes in the yeast *Saccharomyces cerevisiae* [1], [2], [6], [7], [8], [9]. These maps were generated by Chromatin ImmunoPrecipitation (ChIP-seq) or micrococcal nuclease digestion (MNase-seq) and deep sequencing [10]. We analyzed signal and noise using admittedly unattractive but unbiased displays and statistics of the sequencing tag density profiles of the genome. We also introduce here a robust algorithm for calling nucleosome peaks. Specifically, we examined how nucleosomes were remodeled during transcription or by histone chaperones [11] and chromatin remodeling enzymes [12]. We compared in vivo positions to positions of nucleosomes reconstituted from purified DNA and histones in yeast [1], [2] and sheep [13], [14].

To allow Pol II passage, DNA replication and DNA repair, nucleosomes need to be evicted or remodeled at least partially or temporarily. In vitro, Pol II can pass through nucleosomes by forming DNA bubbles [15], [16] but in vivo, the Pol II complex may force histone octamers to completely dissociate from the DNA [17]. Alternatively, the FACilitator of Transcription (FACT) complex can evict only a single H2A–H2B dimer [18]. In either case, nucleosomes are reconstructed within a minute if FACT, SWI/SNF, and the histone chaperone ACT1 are present [19], [20], [21]. Independently of transcription, nucleosome octamers and hexamers can slide on the DNA either spontaneously or assisted by powerful ATP-dependent chromatin remodeling complexes [22]: RSC in yeast and ACHF in human can reposition nucleosomes to DNA loci that are thousand times less affine to histones than the original loci [23].

Traces of remodeling are reproducible in the nucleosome maps. We found that high-level nucleosome occupancy is similar in vivo and in maps of nucleosomes reconstituted by salt dialysis, showing that occupancy at the level of most GGRs is strongly influenced by the sequence of DNA. Weak histone-DNA affinities appear to facilitate nucleosome remodeling at transcriptional landmarks even when reconstituted in vitro in the absence of the transcriptional apparatus. However, at the level of individual nucleosomes, inaccurate in vivo remodeling and sliding are likely due to transcription, remodeling enzymes or chaperones [24], [25].

Remodeling signals can be deconvoluted from the considerable noise. Fuzzy reconstitutions are shown by the reproducibility, width and height of these peaks. Our minimally biased peak calling algorithm allowed us to overlay peak distributions on GGRs and to identify statistically significant trends and patterns from 26 experiments/replicates. We found that in vivo, most nucleosomes reposition in a range of 156–174 bp compared to the ∼147-bp footprint of a single histone octamer on DNA in crystallographic studies [26]. Nucleosomes slide and/or reposition more intensively in vivo in the presence of chaperones and remodeling enzymes than in nonlinked experiments, where histones were not cross-linked to their in vivo loci by formaldehyde. The fuzziest peaks were formed by nucleosomes reconstituted from purified histones and DNA either in yeast [1], [2] or sheep [13], [14], [27]. Intensive eviction and fuzzy remodeling at the centers of transcriptionally active genes indicate Pol II-complex-mediated remodeling. This remodeling is subject to at least two constraints. First, most individual nucleosomes reposition around well-defined centers and seldom invade ranges of other nucleosomes. Second, nucleosome remodeling is also constrained to gene boundaries or shorter limits, possibly to prevent the accessibility regulation of untargeted genes.

## Results

### Reproducible, regional nucleosome occupancy patterns are due to intrinsic histone-DNA affinities

**Figure 1:**
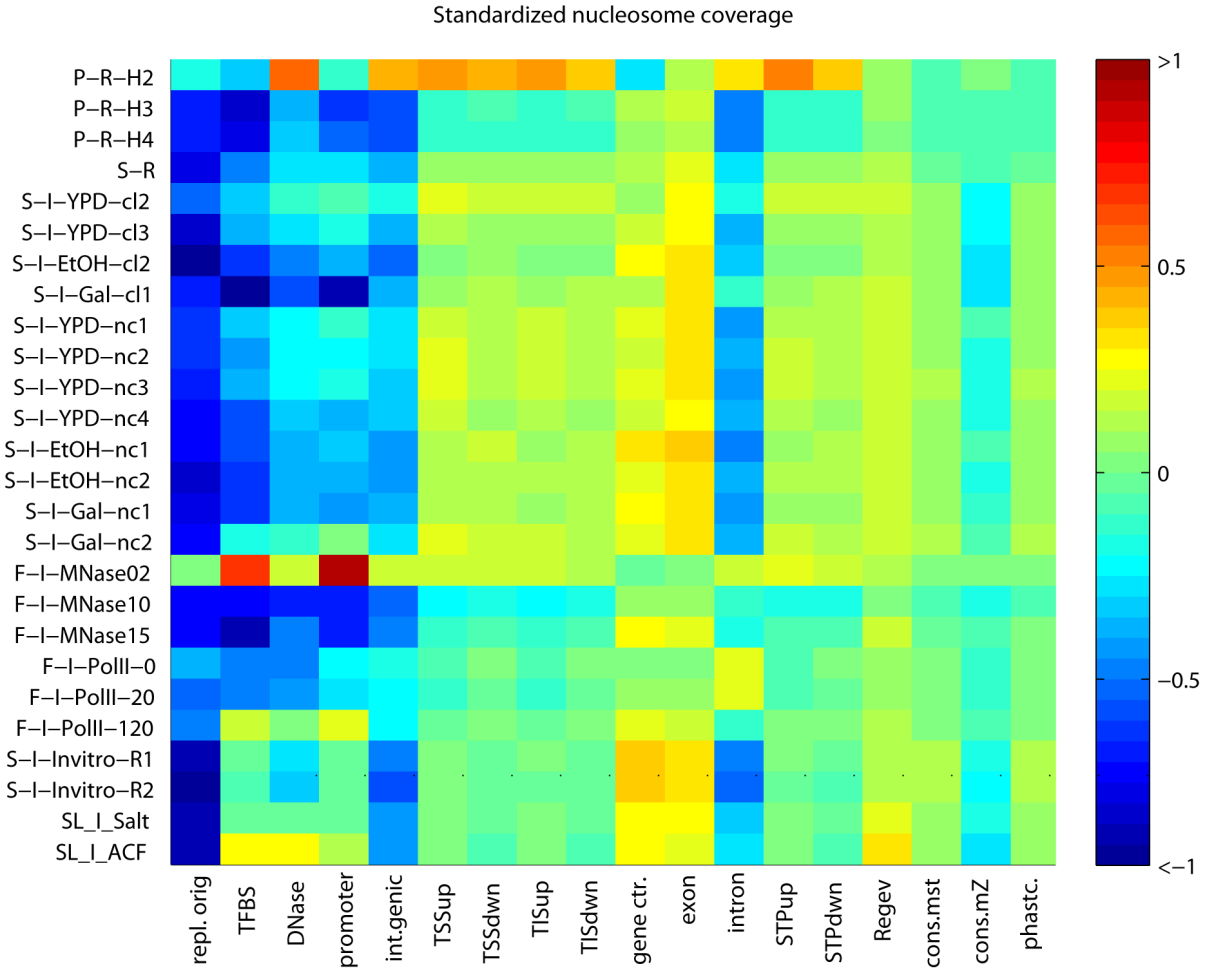
Standardized nucleosome occupancy at GGRs. Sequencing tag density values are standardized to zero mean and unit standard deviation. General nucleosomes are abundant in exons, and conserved regions. H2A.Z nucleosomes are overrepresented around the TIS, upstream of the STOP codons and in introns. Experiments are coded as follows: the first letter indicates the last or the last two authors (“P” for Pugh [7], [8], “S” for Segal [1], [6], “F” for Friedman [9],“A” for Allan [14], [27], “SL” for Liu and Struhl [2]); the second letter indicates the deep sequencing platform (“R” for Roche/454 and “I” for Illumina/Solexa); “H2A.Z”, “H3” or “H4” stands for the specificity of the antibody used; and the carbon source is indicated by “YPD” for glucose, “EtOH” for ethanol and “Gal” for galactose. “cl” indicates histones cross-linked to the DNA in vivo, “nc” indicates the no cross-linking, both followed by replicate numbers. The number after “MNase” indicates the micromolar concentration of the enzyme, and the number after RNAP (Pol II) show the minutes after heat inactivation of the thermo-sensitive Pol II mutant [9]. “Invitro” refers to the Segal group's in vitro reconstitutions, [1] “Salt” indicates reconstitution followed by salt extraction, and “ACF1” stands for reconstitution in the presence of the *Drosophila ACF1* and *Nap1* histone chaperones [2]. . “Regev” indicates the deep sequencing transcriptome published by the Regev laboratory [37], “cons. mst. stands for the most conserved regions, cons.mZ for multiZ conserved regions, phastcon. for phastcons regions [36].

**Figure 2 pone-0012984-g002:**
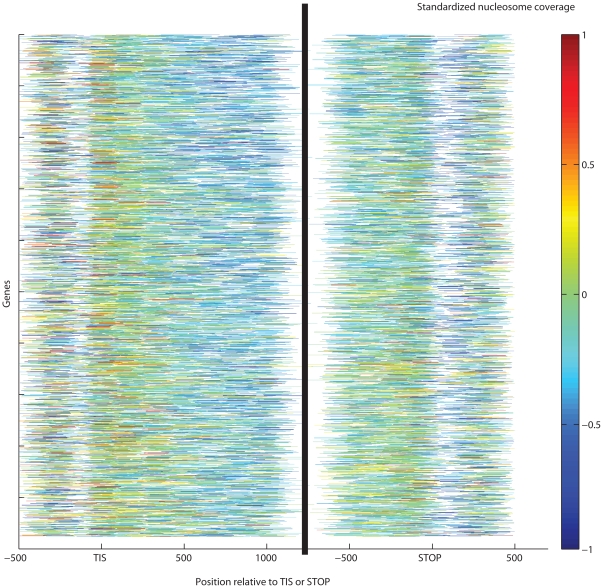
Patterns of standardized nucleosome occupancy across all yeast genes. Lower occupancy shows incomplete nucleosome reconstruction at the mid-thirds of abundantly transcribed genes. The ±300 bp neighborhoods of TIS and STOP codon display higher nucleosome coverage. Genes are ordered by median transcript level in a compendium of gene expression experiments [41] with the most actively transcribed genes at the top. We show the Segal laboratory's in vivo Illumina experiment with galactose carbon source [1].

We define protein-influenced positioning as the combined effects of the transcriptional apparatus, chaperones and remodeling enzymes. The extent to which transcriptional competency is influenced by the DNA versus proteins by can be estimated by comparing in vivo and in vitro maps [1], [2]. To resolve the published opposing conclusions, we perform such comparisons both at the levels of GGRs and at the level of individual nucleosomes. These comparisons are based both on nucleosome occupancy and the width of the nucleosome peaks. Occupancy roughly indicates the fraction of a region occupied by nucleosomes in the average of multiple cells. This robust measure does not depend on peak calls and works well even on maps generated from end-primed short reads. To allow comparisons of diverse experiments, we define *standardized nucleosome occupancy* as the difference between the average density of sequencing tags over a region and the genome-wise average divided by the genome-wide standard deviation of tag density. Conveniently, standardization leads to zero mean and unit standard deviation over the genome (see Methods and Figures 1–2). Standardization also mitigates extremely high read-density peaks caused by amplification bias [28], low sequence complexity or frequent sequence motifs. Because correction for such biases, to our best knowledge, remains an open problem, we cannot meaningfully estimate occupancy on a zero-to-one scale. Another issue is the division by genome-wise standard deviations that differ between experiments. This was necessary for visualization purposes but it may have inflated the similarity between experiments on Figure 1. Fortunately, we performed the statistical tests on raw, unstandardized occupancy distributions to find unbiased, significant differences between GGRs within the same experiment.

The nucleosome occupancy of most GGRs is specific and highly reproducible across the majority of experiments. This reproducibility is moderately biased by standardization. It is highly visible on heat maps of nucleosome occupancy where experiments are represented as rows and GGRs as columns (Figure 1). The striking column-wise patterns indicate the reproducibility of the GGR-wise occupancy across many experiments. Most notably, the occupancy of gene centers exceeds the genomic average by ∼0.2 standard deviation (sd) units. The exceptions are the H2A.Z nucleosomes [7], known to be depleted in translated regions and the experiment with low-concentration MNase that creates artificial coverage at linker regions [9]. At exons and certain noncoding transcripts, occupancy was ∼0.1 sd unit higher than in the genomic average. This indicates that nucleosome presence is necessary for transcription, and temporarily or partly evicted nucleosomes may be fully reconstituted following Pol II passage. In contrast, the occupancy of the ±300 bp neighborhoods of TIS and STOP codons are close to the genomic average. Possibly due to Pol II pausing or slow progression immediately downstream of the TIS [29], nucleosomes or potential agents anchoring them in the 5′ untranslated region and in the first third of the coding region may have the strongest role in downregulating transcription. Below we support these results by nucleosome dynamics results using consistently called nucleosome peaks.

AT-rich regions are known to disfavor nucleosomes [30], therefore a considerable nucleosome depletion at replication origins (∼−0.5 sd units below the genomic average) is in accordance with earlier small-scale experiments [31] . We also confirmed significant depletion at intergenic regions (∼−0.2–−0.3 sd units, *p*<10^−300^,Wilcoxon-Mann-Whitney (WMW) test performed on the raw, unstandardized data, see Methods), in particular at all regulatory regions such as promoters [7], [32], [33], transcription factor binding sites and DNase hypersensitive regions [34]. Significantly high occupancy was confirmed for H2A.Z nucleosomes at promoter termini and introns, in the vicinity of TSS, TIS and STOP codons (Figures 1–2).

### Wide nucleosome peaks indicate fuzzy remodeling of H2A nucleosomes

**Figure 3:**
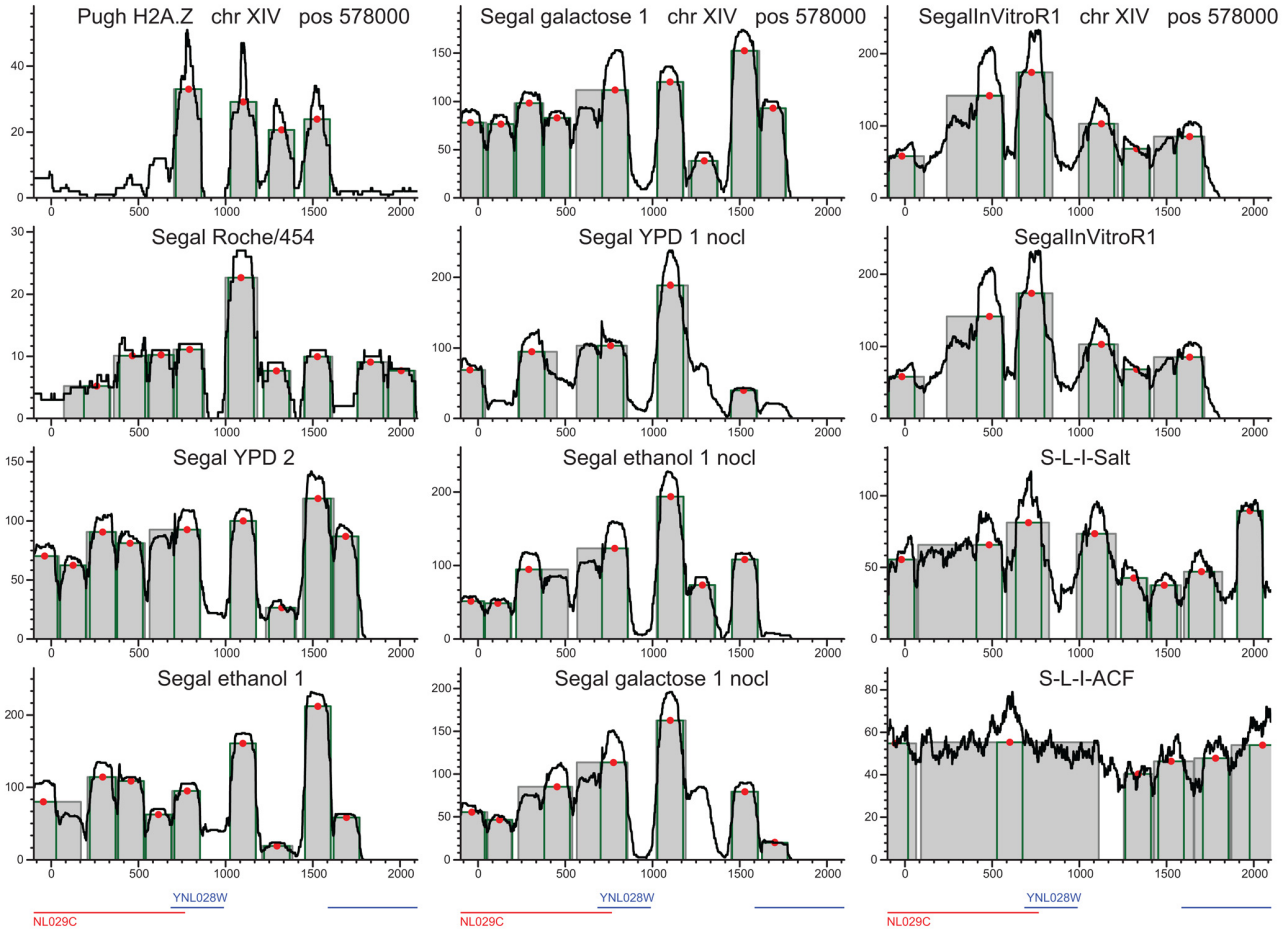
Reproducible positioning of four H2A.Z nucleosomes at chromosome XIV. H2A.Z nucleosomes are indicated on the same loci in vivo, nonlinked and in the salt extraction reconstitution experiment as well. The only exception is the reconstitution with the ACF1 histone scaffold, which interfered with accurate, DNA-influenced positioning. The density of sequencing tags is shown by black lines. Peak location calls are displayed as gray rectangles and green outlines indicate the single octamer footprint. Red dots represent the peak's center of gravity. These H2A.Z peaks barely extend beyond the single octamer footprint, in sharp contrast to the nucleosomes in the middle of the coding regions.

A simple visual inspection at our web site (http://chromatin.unl.edu/cgi-bin/skyline.cgi) reveals that sequencing tags mapped to the genome form peaks diverse in shape, width and height. Most of these peaks extend considerably wider than the single-nucleosome footprint obtained in crystallographic studies [26]. This is primarily due to fuzzy remodeling as opposed to incomplete digestion and other experimental issues. We confirm that by systematic comparisons to those nucleosomes where the canonical H2A subunits are replaced by variant H2A.Z subunits (Figure 3, for details, see the section “Are wide nucleosome peaks due to remodeling or experimental noise” below). Most H2A.Z nucleosomes are well-positioned [7], their average width barely exceeds the 147 bp footprint of a single histone octamer.

While Roger Kornberg and coworkers advocated nucleosome positioning by remodeling agents and random factors [24], [25], [35], [36], advocates of DNA-encoded positioning idealized nucleosome peaks to the width of the single-nucleosome footprint by loess normalization or wavelets [7], [8], [37]. Unbiased remodeling information needs to be preserved because each ChIP/MNase-seq DNA segment whose tags form a nucleosome peak may represent a different cell, and nucleosomes in different cells may occupy somewhat different genomic loci. To take this into account, Weiner et al. identified peaks by template filtering using seven templates [9]. As a further improvement, we introduce a consistent, template-free, and fully reproducible peak calling algorithm that is minimally biased by hypotheses about peak width, shape and other parameters (see Methods). Consistent calls across the whole genome and experiments allowed us to compare in vivo, nonlinked and in vitro nucleosome peaks. We also compared results of the Illumina vs. Roche/454 sequencing, growth media experiments, and replicates that quantify biological and technological variation. Users can access interactive, visual displays of peak calls and the undistorted density profiles of sequencing tags at our web server: http://chromatin.unl.edu/cgi-bin/skyline.cgi.

### Nucleosome remodeling and transcription

**Figure 4 pone-0012984-g004:**
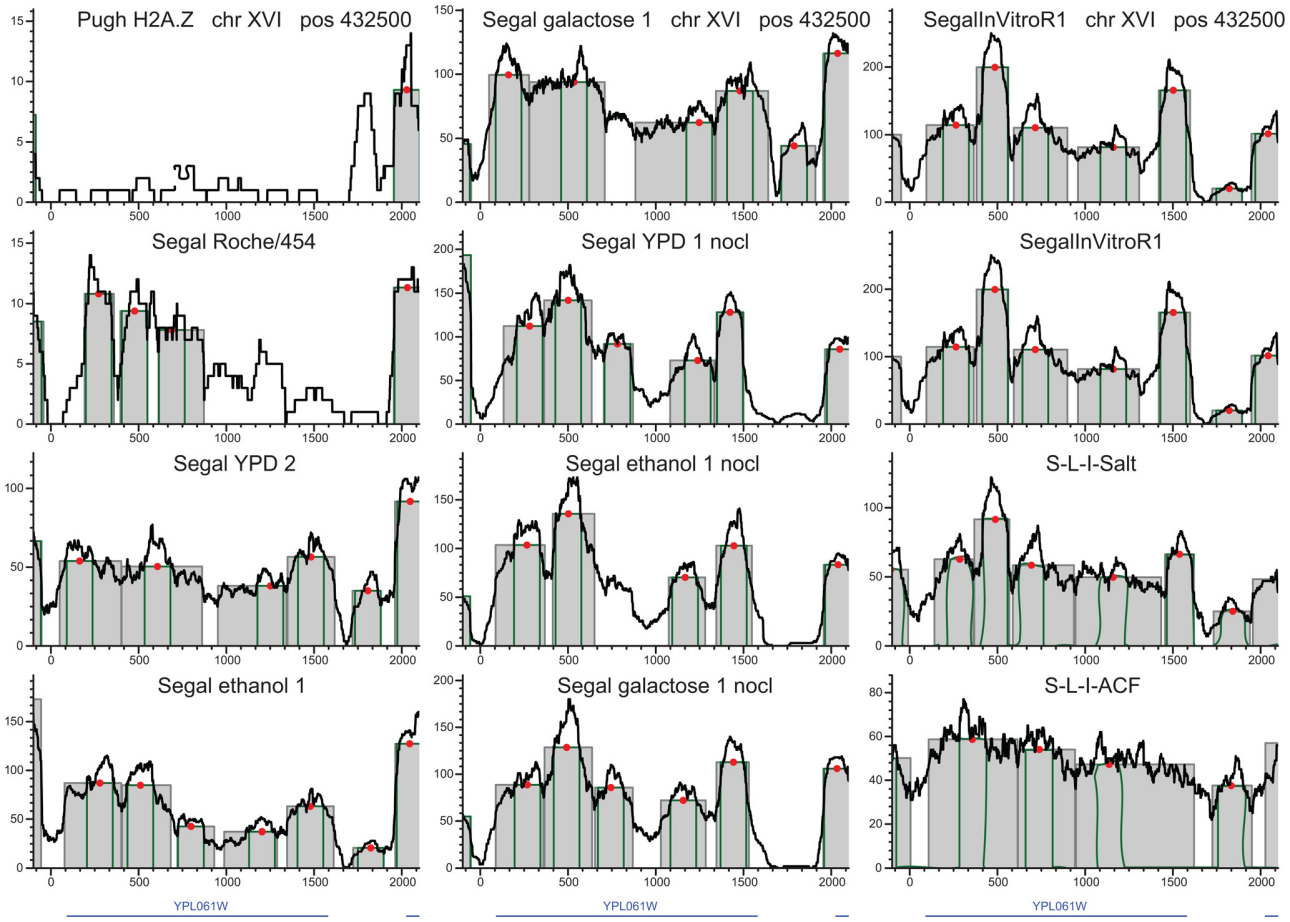
Nucleosome peaks at the constitutively expressed gene for cytosolic aldehyde dehydrogenase (*ALD6*). The peaks between the relative positions of 200 to 760 bp and 960 to 1560 bp show extensive sliding or remodeling. Nucleosome positioning is more statistical *in vivo* than in the nonlinked experiments, particularly in the mid-third of the coding region.

**Figure 5 pone-0012984-g005:**
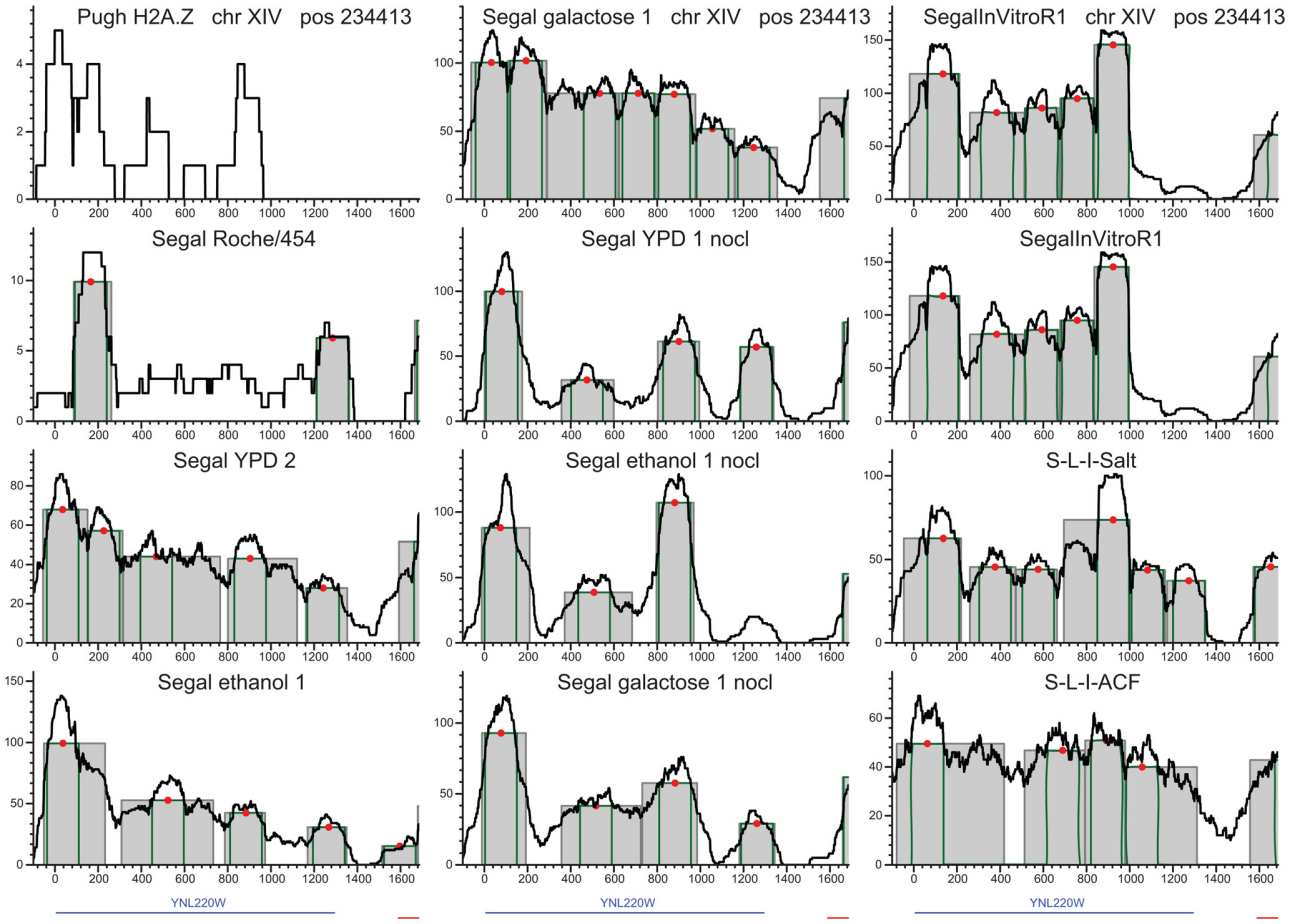
In vivo, nucleosomes occupy alternative positions, particularly in the middle of the ADE12 gene that codes for adenylosuccinate synthase. Better focused positioning is observed in nonlinked experiments and in reconstitutions following salt extraction. Reconstitutions with the ACF1 chaperone produce fuzzier positioning.

Our results confirmed the sharp contrast between the two basic classes of nucleosomes. H2A.Z nucleosomes are typically located around promoter regions. H2A.Z peaks are tall, well-defined, and barely exceed the 147 bp width of the single-octamer footprint [26] (Figure 3). In contrast, most nucleosomes with the canonical H2A subunits form wide, irregular peaks with multiple rises and drops in the tag density profile (Figures 4–5, S1, S2, S3, S4, S5). These patterns manifest on the gene for cytosolic aldehyde dehydrogenase 6 (ALDH6, Figure 4 [38]). Notwithstanding constitutive expression, four-to-five peaks cover most of ALDH6 gene, all negative for the H2A.Z variant. Peaks are moderately reproducible in vivo but separate better in nonlinked experiments. This indicates that intrinsic histone-DNA affinities play minor roles in positioning individual H2A nucleosomes in vivo, and that these positions are frequently modified by the transcriptional apparatus, chaperones and remodeling enzymes. Reconstitutions from purified DNA and histones [2] produced extremely fuzzy peaks. Within a peak, multiple summits in tag density indicate competing maxima of histone-DNA affinity, and nucleosomes appear to jump from one competing maximum to another. The histone chaperone ACF1 tends to further delocalize the nucleosome peaks in vitro. In our opinion, however, this observation does not exclude primarily DNA-influenced nucleosome positioning because positioning to the maxima of histone-DNA affinity may require remodeling enzymes. In vivo, fuzzy positioning is apparent in the gene for ADE12, adenylosuccinate synthase, particularly at the middle of the coding sequence (Figure 5). Mid-gene remodeling is abundant at the gene for the MED2 subunit of the Pol II mediator complex (Figure S1); SMC5, structural maintenance of chromosomes (Figure S2); LDB17 (Figure S3); or the SR077 gene (Figure S4).

**Figure 6 pone-0012984-g006:**
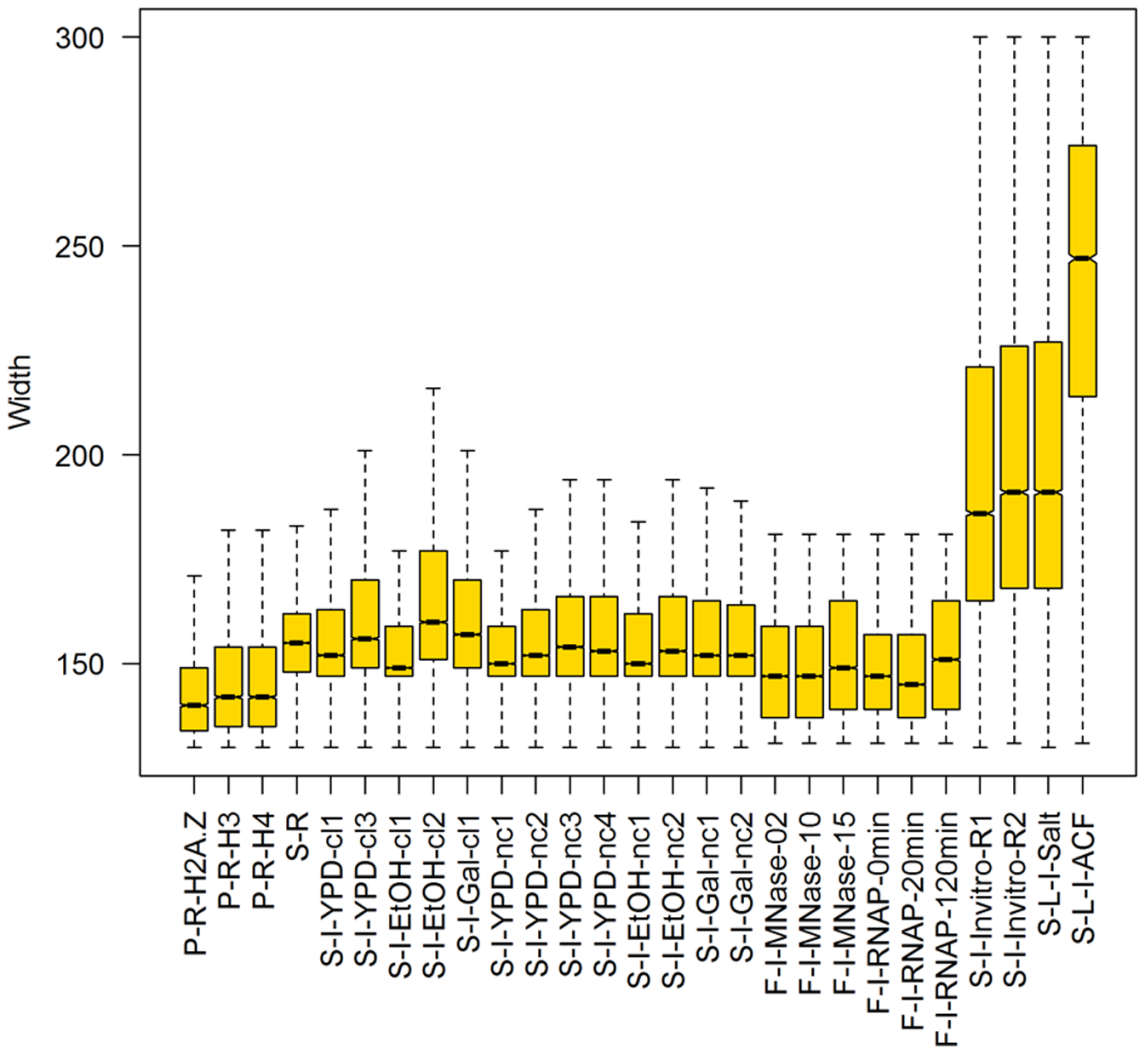
Nucleosome remodeling as reflected by the width distributions of nucleosome peaks. H2A.Z nucleosomes (less than 10% of the total) form regular peaks but most general nucleosome peaks span considerably wider than the single octamer footprint of 147 bp. In vivo peaks are significantly wider than nonlinked peaks. Reconstituted nucleosomes frequently delocalize to ill-defined peaks wider than 200 bp. Note that peaks in end-primed experiments with short reads (diverse MNase levels and RNA Pol II mutants) were called by template filtering and hence not comparable to the other experiments.

These individual observations are generalized by genome-wide statistics. A quarter of the in vivo H2A-peaks extend to 159–179 bp or wider (Figure 6, Table 1), and the widest 10% of peaks span to 177–200 bp or wider. Nonlinked nucleosomes form somewhat narrower peaks (average: 158–163 bp, *p*<10^−300^, WMW test). Each of these peaks consists of hundreds of sequencing tags. In turn, these tags come from a still considerable number of DNA segments, each representing a different yeast cell. Therefore peak widths reveal the positional variation of the histones on the DNA subject to experimental noise such as incomplete digestion by the MNase enzyme.

**Table 1:** The prevalence of extended nucleosome peaks.

*Experiment*	*Peak width*, *bp*					*Number of peaks*	*Mean peak coverage*
	*Mean*	*Median*	*75th*	*90^th^*	*std*		
			*percentile*				
P-R-H2A.Z	152	149	158	171	13	2,873	21
P-R-H3	157	152	163	179	18	4,833	43
P-R-H4	156	151	163	177	17	4,504	29
S-R	160	157	164	174	18	23,530	9
S-I-YPD-cl2	161	153	164	183	25	12,753	87
S-I-YPD-cl3	167	157	172	198	28	16,566	55
S-I-EtOH-cl1∧	157	149	159	177	20	14,934	114
S-I-EtOH-cl2	174	161	179	227	37	19,223	82
S-I-Gal-cl1	168	158	172	200	32	23,023	94
S-I-YPD-nc1	158	151	160	180	19	18,747	107
S-I-YPD-nc2	160	153	163	185	22	17,029	130
S-I-YPD-nc3	162	154	167	190	24	18,181	124
S-I-YPD-nc4	163	154	167	192	25	17,498	128
S-I-EtOH-nc1	160	151	163	187	23	21,523	98
S-I-EtOH-nc2	163	154	167	192	25	17,432	108
S-I-Gal-nc1	162	153	166	191	24	22,866	85
S-I-Gal-nc2	160	153	164	186	21	19,807	132
F-I-MNase-02*	150	147	159	173	14	31,261	29
F-I-MNase-10*	150	147	159	1173	14	32,048	15
F-I-MNase-15*	152	149	165	175	15	26,432	49
F-I-RNAP-0*	136	137	149	163	20	62,538	31
F-I-RNAP-20*	132	131	147	161	21	62,582	26
F-I-RNAP-120*	134	131	151	169	23	53,686	30
S-I-Invitro-R1	197	185	219	263	40	30,941	82
S-I-Invitro-R2	200	190	224	265	40	28,289	79
SL-I-Salt†	163	157	166	178	24	34,717	268
SL-I-ACF†	211	206	235	264	35	13,655	188
A-I-sheep†	174	169	190	232	39	29	8,793
A-R-sheep†	209	200	228	276	40	29	1,305

∧ The S-I-EtOH-cl1 replicate was more random than the comparable experiments and was excluded from further analyses.

*Peaks in these end-primed experiments are called by Weiner et al.'s (2010) template filtering method.

† In reconstitutions from purified DNA and histones, the highly random protein-influenced positioning frequently makes peak calling uncertain (see Figure S5).

The distributions of peak widths, the numbers of peaks are shown, along with the mean density of uniquely mapping sequencing tags at all peaks. For the abbreviations of experiments, refer to Figure 1.

**Figure 7:**
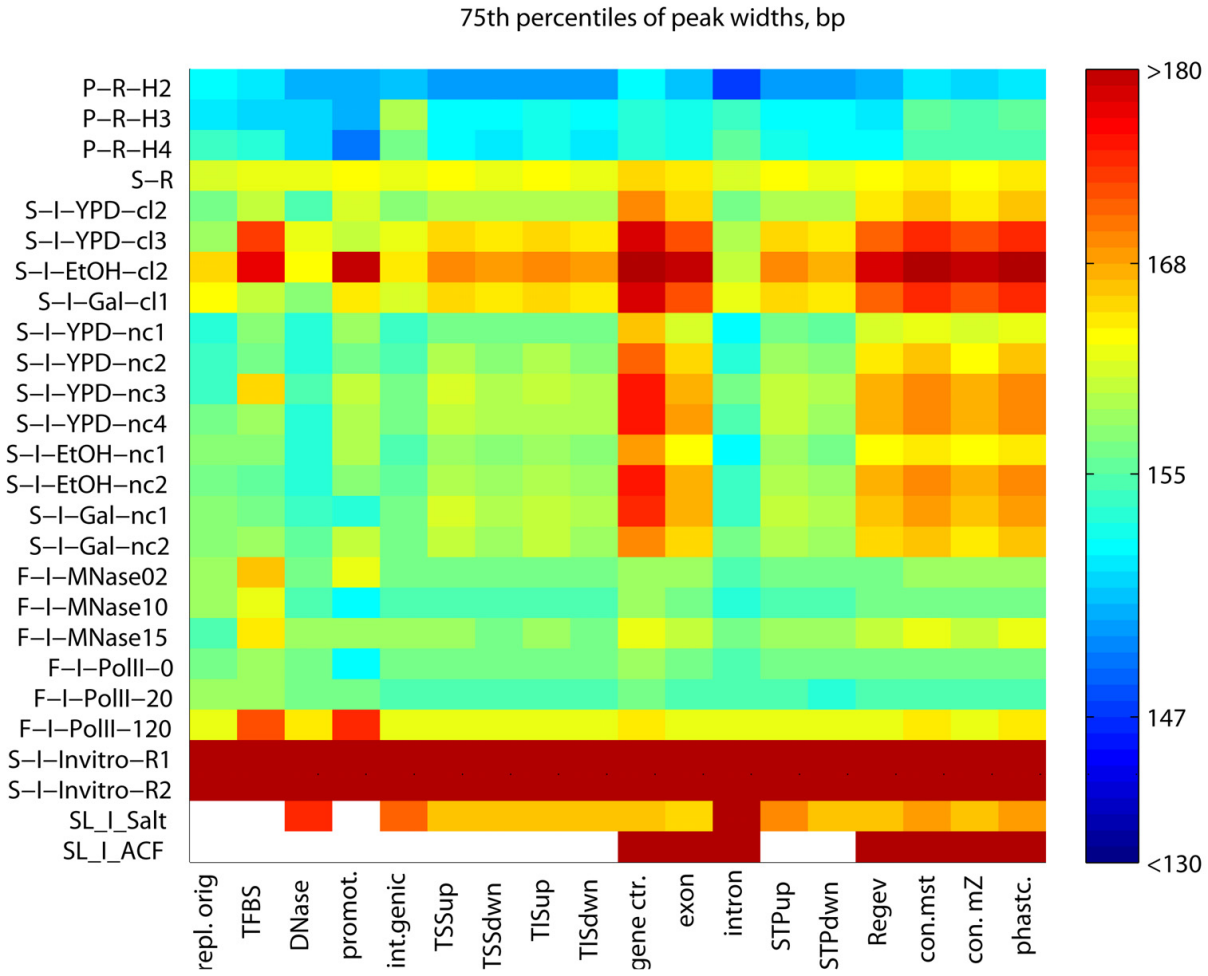
GGRs have characteristic patterns of nucleosome dynamism. We show the 75^th^ percentiles of the peak width distributions. Only H2A.Z peaks, the heavily size-fractionated P-R-H3 and P-R-H4 experiments, and peaks predicted by template filtering (F-I-MNase10 through F-I-RNAP-120m) do not exceed width of the single-octamer footprint.

**Figure 8:**
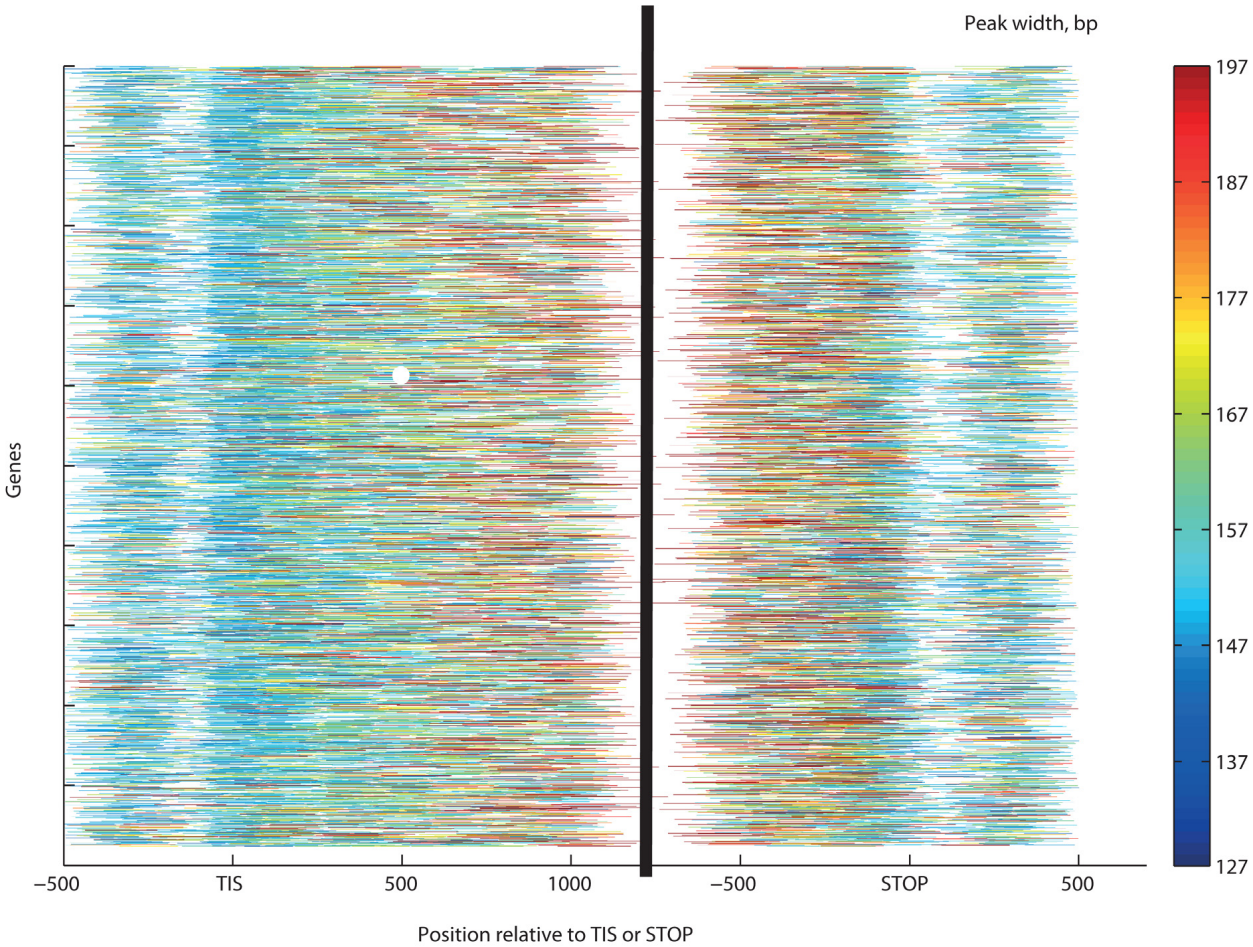
Gene-wise patterns of nucleosome repositioning as indicated by peak widths. Color codes indicate standardized nucleosome occupancy, and white indicates the absence of sequencing tags or nucleosomes callable by our method. The most active repositioning was found in the mid-third of coding sequences (171±44 bp). Significantly narrower peaks were found in the ±300 bp environment of the TIS (162±41 bp) or the STOP codon (161±41 bp). Most well-positioned nucleosomes are located in the proximity of TIS and the STOP codons.

Our results indicate that positional variation is caused primarily by transcription-related nucleosome remodeling and eviction. This would imply higher dynamism at genic regions where Pol II is most active. Indeed, GGRs have very specific positioning strength patterns that are reproducible across most experiments (Figure 7), much like the patterns of nucleosome occupancy above. Unusually wide nucleosome peaks (denoted by hot colors) are particularly abundant at the centers of the transcriptionally active genes and at conserved noncoding DNA elements [39], while narrow peaks (blue colors) are concentrated in promoter and intergenic regions. Most H2A.Z nucleosomes form narrow peaks, close to the single-octamer footprint. In vivo, peaks expanded wider (169±22 bp) than in the ±300 bp neighborhood of the TSS, TIS or STOP codons (160±21, 151±14 and 155±14 bp, respectively, all with *p*<10^−300^, WMW test; Figure 7). Peaks span wider in protein-coding genes and deep sequencing transcripts [40] than in intergenic regions or the few introns in yeast. The top expression quartile of genes from [41] harbors somewhat wider peaks than the lowest quartile (means: 176 vs. 172 bp, respectively, *p* = 0.002; Figure 8). From the extended peaks, particularly at the centers of transcriptionally active genes, we infer that nucleosomes are reconstituted with an inaccuracy of ∼20 bp. The more accurately positioned nucleosomes, however, stop repositioning near the intergenic regions.

The variable strength of DNA-influenced positioning is most visible on the in vitro nucleosome reconstitutions from purified ovine β-lactoglobulin DNA and chicken erythrocyte histones in the absence of histone chaperones and remodeling enzymes [13], [14], [27] (Figure S5). Intrinsic histone-DNA affinities are likely to be responsible for the high summits in these exceptionally high-coverage experiments. However, most peaks are not separated well from each other and many peaks appear to be merged. Similar, often fuzzy positioning was revealed by yeast reconstitution experiments (Figures 3–5 and S1, S2, S3, S4) [1], [2].

### Are wide nucleosome peaks due to remodeling or experimental noise?

We have been concerned regarding experimental error in chromatin immunoprecipitation followed by deep sequencing [ChIP-seq, 10, 42] and in the differential digestion of the excess DNA by micrococcal nuclease (MNase) [43]. Field *et al.* [6] found that in over a million of MNase cleavage sites, sequence-specific bias is limited to primarily two consecutive base pairs, and the preferred sequences can be found in nearly all short DNA segments. A more realistic concern is that linker histones, chaperones and remodeling agents may block interactions between the DNA and MNase, leading to incomplete digestion [44]. Hence too low or too high MNase concentrations lead to overly wide or overly narrow peaks in the density profiles of the ChIP-DNA or MNase-DNA sequencing tags [9]. Fortunately, that does not affect comparisons between experiments using similar MNase concentrations or comparisons of regions within one experiment.

#### The effects of limited nuclease accessibility, incomplete cleavage and bias

One could argue that crowding with other nucleosomes or nonhistone proteins could limit the accessibility of DNA for MNase digestion and that the observed wide peaks were only artifacts of crowding. Let us examine this argument using in vitro nucleosome reconstitutions and well-separated nucleosomes.

#### Nucleosome peaks span wide even when accessibility is not limited by nonhistone proteins

In vitro reconstituted nucleosomes have not been associated with or positioned by nonhistone proteins. Our algorithm called peaks in the extremely high-density tag profiles of the sheep β-lactoglobulin gene [13], [14], [27] and in yeast [2]. In the experiments with short Illumina tags, the average nucleosome peak extended to 174±39 bp and a quarter of peaks spanned wider than 190 bp. The long tags in the Roche/454 experiments resulted in even more extended peaks (Figure 6, Table 1). These extended peaks cannot be due to crowding or other effects of nonhistone proteins.

#### Reduced accessibility due to nucleosome crowding

One could also argue that typically short linker regions between H2A nucleosomes (38±55 bp) limit nuclease access to DNA and the resulting incomplete digestion creates artificially extended peaks. One could also argue that H2A.Z peaks are digested short because their long linker regions (88±247 bp) allow more access for digestion than the short linkers for H2A nucleosomes. To evaluate these arguments, we selected such H2A nucleosomes that are separated by long linkers (88 bp or more) on both sides and that, unless protected by nonhistone proteins, are accessible to MNase digestion. Because these accessible H2A nucleosomes still extend to as wide as 170±54 bp, at practical MNase concentrations, crowding with histones or other DNA-bound proteins are not likely to have major effects for extended peaks. Lowering MNase concentration from 10 µM/L to 2 µM/L did increase the length of core DNA segments both in yeast [9] and in *Caenorhabditis elegans* [45]. In the latter, decreasing the temperature also produced longer DNA segments but Johnson *et al.* [45] estimated that at room temperature and practical MNase levels under- and overdigestion ranges to as low as a few base pairs.

As a final test, we benchmarked the *reproducibility* of those nucleosomes that contained the H2A.Z variant subunits [46], and were mapped using selective antibodies [7]. We selected all the 297 peaks with high average tag densities (≥20x) so as to reduce the effect of limited H2A.Z specificity of the antibodies. These peaks are practically as wide (median: 149 bp) as the single-core footprint both in vivo and in nonlinked experiments (Figure 6). Many of them guard promoter regions against nucleosome invasion [8], [47] because they are firmly anchored to the DNA either by intrinsic histone-DNA affinity or by chaperones. Indeed, a total of 225–258 peaks overlapped with peaks in 21 high-coverage experiments (Table S1). Somewhat fewer (190) overlaps were found with the template-filtered peaks of the RNA-Pol II mutant after 120 minutes following induction [9].

We observed a reproducibility of 13.5 bp being defined as the median positional difference between known H2A.Z peaks and overlapping peaks in all other in vivo experiments. Consequently, peaks wider than 161 bp are highly likely to represent dynamic nucleosomes. Because this 13.5-bp reproducibility is acceptable but not negligible, we tested the contribution of nonhistone proteins to the location of H2A.Z-overlapping peaks by comparing in vivo and nonlinked nucleosomes. The latter are more reproducible (7.5 bp) but nucleosomes in vitro reconstitute into more fuzzy positions. The 6-bp difference from in vivo and nonlinked experiments is highly significant (*p*<10^−300^, WMW test) and shows a major role for chaperones [48] in positioning H2A.Z nucleosomes, which is confirmed by the in vitro reconstitutions in the presence of ACF1 and Nap1 [2]. We estimate that the total average inaccuracy caused by incomplete or biased cleavage, amplification bias and the bona fide mobility of H2A.Z nucleosomes is as low as ∼7.5 bp. Cleavage, amplification bias and limited DNA accessibility for MNase digestion combined are not the primary determinant of the extended peaks. Instead, we hypothesize that the extended peaks are primarily due to statistical (re)positioning [24], [25] or sliding [49] of nucleosomes. This hypothesis is supported by the significantly wider peaks found at the centers of highly transcribed genes compared to at either terminus or at intergenic regions.

### Remodeling stops at gene boundaries

Preserving regional differences in nucleosome occupancy and dynamism would not be possible without agents that arrest the progression of remodeling. These agents include nucleosome-excluding sequences on the DNA, histones or other DNA-associated proteins. Another reason for the spatial limits to nucleosome remodeling is the arrest and detachment of the transcriptional machinery at the 3′ termini of genes. The third reason for spatial limitations relates to effects of nucleosome presence and mobility to gene regulation [21]. To prevent regulating untargeted neighboring genes, remodeling events need to be contained within the limits of the target gene. The earlier proposed “barrier” nucleosomes guard only promoter regions against invasions of dynamic nucleosomes [8], [47]. We also found barrier nucleosomes within and downstream of the coding sequences. These well-positioned nucleosomes are reproducible across all the thirteen high-density in vivo Illumina experiments with random priming in 58% of the genes (Figures 3–5 and S1, S2, S3, S4, S5). Across seven or more experiments, 85% of genes contained at least one well-positioned nucleosome (Table S1). Across the entire yeast genome, almost every 2000-bp segment with two or more dynamic nucleosomes also contains at least one well-positioned nucleosome, whether removable or permanent. As few as 288 segments lacked well-positioned nucleosomes in each of Kaplan *et al.*'s [1] thirteen Illumina experiments. Several of these are nucleosomes are frequently evicted as shown by the low density of their peaks (only ∼2.5 times higher than at the linker regions). Arrays of well-positioned nucleosomes may arrest nucleosome remodeling at gene boundaries and hence prevent accessibility regulation of neighboring genes.

## Discussion

We offer partial reconciliation for the controversy between two claims: “… DNA-encoded nucleosome organization…” and “intrinsic histone-DNA interactions are not the major determinant of nucleosome positions in vivo.” Note that Kaplan et al.'s observed high correlation between in vivo and in vitro positions does not necessarily indicate well-positioned nucleosomes: two maps with similarly fuzzy remodeling patterns (dynamism) also produce high correlation. Indeed, most nucleosomes are dynamic even in Kaplan et al.'s in vitro reconstitutions where the 0.4∶1 histone∶DNA ratio allowed that histones occupy only the most affine and hence least dynamic positions. Dynamism is even more prevalent in Zhang et al.'s experiments with nonlimiting histone∶DNA ratios (Table 1, Figures 1–3, S1, S2, S3, S4, S5). This dynamism manifests at the level of individual H2A nucleosomes, the transcriptional apparatus, chaperones and remodeling enzymes cause inaccurate reconstitution or sliding. At the level of GGRs, however, histone-DNA affinities appear to be the primary determinant of nucleosome occupancy. Both the dynamism of individual nucleosomes and the GGR-level affinities are highly related to transcription. Nucleosomes, like transcription, are influenced by both DNA-specific features like the location of exons, promoters and temporal features like the momentary location and state of the transcriptional apparatus and regulatory events.

The dynamism of individual H2A nucleosomes is indicated by extended and low peaks both in vivo and in vitro. At gene centers, peaks shrink significantly lower than at other regions. Low but wide peaks indicate fuzzy reconstitution of nucleosomes evicted by the transcriptional apparatus. These peaks also spanned significantly wider in the mid-third of genes than in introns, regulatory regions, replication origins that are known to be AT-rich and therefore less likely to coordinate nucleosomes [30], and the rest of the genome (Figures 4–7, S1, S2, S3, S4, S5). Reconstitution is particularly inaccurate at highly expressed genes (Figures 2 and 8). This is in accord with earlier observed removal of at least one H2A/H2B dimer by the chromatin transcription-enabling activity (CTEA) complex [CTEA], [ 15], [18,29] to allow Pol II passage and the fast reconstitution of nucleosomes by CTEA and the FACT histone chaperone shortly after Pol II passage [29], [50]. The observed extended nucleosome peaks are primarily due to bona fide biological dynamism, which exceeds the combined bias of limited DNA accessibility, incomplete MNase cleavage, and amplification. Among others, this is shown by variant H2A.Z nucleosomes, which, in contrast to H2A nucleosomes, form narrow and highly reproducible peaks. Regional patterns of transcription-specific dynamism are also highly reproducible.

Nucleosome occupancy and dynamism are lower at the first and last thirds of genes than at the center, both in vivo and in reconstitution experiments. The high histone-DNA affinity of these regions may have evolved to facilitate Pol II pausing, deceleration or delay recovery from pauses, as shown by dual-trap optical tweezer assay experiments [16].

Most H2A peaks extend almost as wide in nonlinked experiments than in vivo (Table 1). In the absence of chaperones and remodeling enzymes, nucleosomes move more freely from one local affinity maximum to another, although these movements are not assisted by remodeling enzymes [1], [6], [51]. The most likely mechanism for nucleosome dynamism is ATP-independent histone sliding [52], which intensifies at higher temperatures [53]. Peaks of H2A.Z nucleosomes, however, are constrained to a median width equal to the single octamer footprint in vivo, in nonlinked and reconstitution experiments as well. This stability and the high positional reproducibility across experiments suggest that many H2A.Z nucleosomes are localized primarily at tall and locally unique maxima of histone-DNA affinity [2]. Note that a low-affinity maximum surrounded by two nucleosome-exclusion regions would also produce a well-positioned nucleosome. Alternatively, chaperones like CZF1 [48] may also anchor nucleosomes to specific loci. Long nucleosome-exclusion regions such as promoters allow binding of numerous regulatory proteins and protect from the invasion of other nucleosomes. Most peaks of barrier nucleosomes rise far above those of dynamic nucleosomes. This indicates that such barrier nucleosomes are more resistant to eviction than H2A nucleosomes and hence are preserved in most cells at almost identical genomic loci, regardless of cellular and environmental conditions. The positioning of H2A.Z nucleosomes is highly reproducible with the sole exception of ACF1-assisted reconstitution (Figure 3). These peaks are confined to the size of the single-octamer footprint with a variation of ∼9 bp, which may partly be due to experimental error.

We generalize the barrier hypothesis, which until now, was limited to H2A.Z nucleosomes [8], [47]. Nucleosome movements are limited, and in vivo, very few nucleosomes reposition in a range wider than ∼195 bp. This and the more or less well-defined peaks separated by troughs indicate that the probability of repositioning to new loci drops at some distance from the center. Occasionally, nucleosomes can invade each other's range, but this may require considerable momentum [54]. Surprisingly, remodeling events extended further than 2,000 bp in as few as 288 cases. On this basis, we postulate that long chains of remodeling events may be prevented by compounding energetic costs that contain remodeling within gene boundaries. This containment may prevent nucleosome invasions that would change the accessibility of untargeted genes. Relatively well-positioned nucleosomes may be responsible for Pol II pausing [15], [29]. Remodeling can be weakened, halted or arrested even by a series of dynamic and eviction-prone nucleosomes not necessarily containing the H2A.Z variant. Each nucleosome and possibly certain nonhistone proteins can absorb some sliding and remodeling momentum [36], depending upon the affinity of the histone octamer to the DNA segment and upon crowding with other nucleosomes and chromatin proteins. Individual nucleosomes can frequently be depositioned, but the remaining nucleosomes can still extinguish the momentum of remodeling before invading untargeted regulatory regions. This protection mechanism requires a lower but still considerable nucleosome presence at intergenic regions as well.

We conclude that dense arrays of weakly positioned nucleosomes appear to be necessary for transcription. The majority of canonical H2A nucleosomes repositions or slides to somewhat different genomic loci. Such nucleosome remodeling follows DNA-influenced, gene-wise patterns and is most intense at the centers of intensively transcribed genes. The weakness of positioning is partly due to either low or nonunique local maxima of intrinsic histone-DNA affinities, and its function is to give way to transcription, chaperones, and remodeling enzymes. Oscillations are centered at fixed positions, and their magnitude and frequency may reflect transcription or regulatory protein binding. Remodeling does not transgress to neighboring genes and may be weakened and ultimately arrested by a series of moderately or well-positioned nucleosomes, chaperones, or possibly by the lack of histone modifications. This combination of DNA- and protein-influenced positioning fine-tunes the accessibility of genomic regions and their competence for regulatory protein binding and transcription, affecting Pol II speed and the extent of its pausing.

## Methods

We mapped each sequencing tag to the *S. cerevisiae* genome's 2006 release [55] using the *bowtie* tool [56], [57]. Tags matching to multiple genomic loci were discarded. We also eliminated short Illumina reads with more than two mismatches to the genome and long Roche/454 tags with more than 2 percent mismatch. To minimize bias, we have not extended or trimmed the sequencing tags. We calculated the tag density of a genomic position as the number of tags that cover the position. Tag density profiles were calculated for each experiment/replicate for the entire genome.

To allow genome-wide comparisons, we created a consistent prediction algorithm and tools to call nucleosome peaks from ChIP-Seq tags (Figures 3–6 and S1, S2, S3, S4, S5). This consistency is a condition for minimally biased comparisons involving histone variants and GGRs. Methods designed for transcription factor binding sites [e.g., 58] are not applicable because nucleosome peaks vary in width and their sequences are not conserved. Nucleosome peaks are also diverse in height and shape. The ideal 147-bp wide rectangle is rather an exception than the rule. Instead of sharp vertical drops, observed peaks may terminate in gradual slopes (Figures 3 and S1, S2, S3, S4, S5). These valuable signals are compromised by smoothing procedures such as wavelets or loess [7], [37].

We created a peak calling algorithm for experiments with random priming with short or long sequencing tags and/or nucleosome-long sequencing tags. First, each chromosomal segment bordered by either the end of a chromosome or by nucleosome-free region(s) into such subsegments that may contain at most one full, possibly extended nucleosome peak. For each subsegment, we identify a set of candidate start positions *p* and end positions *q*, for which the subsegment cannot be divided into additional peaks: 1≤*p_1_*<*q_1_*<*p_2_*<*q_2_*,…,<*p_n_*<*q_n_*. Peaks with deep internal troughs are eliminated as follows. Any peak with a triplet of positions *x*, *y*, and *z* satisfying *p_i_*≤*x*<*y*<*z*≤*q_i_* with a deep trough *y* in a density *d* of sequencing tags
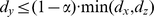
is eliminated. Here *α* is the maximal allowed fractional drop in density inside a peak (by default, *α* is set to 0.25). We optimize the widths (the start and end positions) of peaks that pass this filter by maximizing the objective function below. To prevent overextending the peaks, the cumulative tag density is assigned decreasing weight as the width increases:
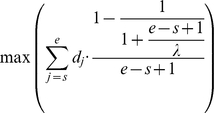
Here *s* and *e* denote the start and the end positions of the peak (*p_i_*≤*s*<*e*≤*q_i_*), respectively. Broad peaks are slightly penalized by the parameter λ. By setting λ to 147 bp, we promote conservative width estimates while still allowing peaks to grow or shrink to any size. Experimenting with several combinations for *α* and λ, we found that values of 0.25≤*α*≤0.28 and 146≤*λ*≤149 accounted for the peak calls with the visually highest quality. As a further quality assurance, we eliminate potential di- and multi-nucleosomes (widths exceeding 300 bp) and peaks with extreme asymmetry or low density.

Due to premature detachment of the reverse transcriptase from the ChIP-DNA, nucleosome calls are most accurate in experiments that use random priming (on any sequencing platform) and/or nucleosome-long reads (on the Roche/454 platform) [1], [6], [7], [8], [14]. Experiments with both end-priming and short reads [9], [32] produce dual peaks (one upstream and one downstream) on the two DNA strands. Detachment becomes increasingly likely as the enzyme progresses towards the 3′ end [42]. Calling the width of dual peaks may be biased by the use of peak shape templates [9].

The consistently predicted peak widths serve as a single-valued, practical measure for the comparative analyses of nucleosome dynamics. Standardized nucleosome occupancy roughly indicates the proportion of cells that harbor nucleosomes at a genomic locus. Peaks with high amplification bias or extremely high density are excluded from the analyses. Yeast nucleosome peak calls are interactively displayed at our web-server: http://chromatin.unl.edu/cgi-bin/skyline.cgi.

None of the statistical distributions analyzed were normal as shown by the Lilliefors test. We applied the two-sample Wilcoxon-Mann-Whitney (WMW) test in all statistical comparisons.

## Supporting Information

Figure S1 All nucleosomes are dynamic at the constitutively expressed MED2 (YDL005C) gene.In particular, the single nucleosome at relative position −40 and the twin peaks starting at relative position ∼450 are subject to extreme sliding or remodeling. The MED2 protein is a subunit of the RNA polymerase II mediator complex, and it is essential for transcriptional regulation.
(0.95 MB TIF)

Figure S2 Extensive sliding/remodeling at the SMC5 (YOL034W) gene. SMC5 encodes a protein responsible for the structural maintenance of chromosomes, required for growth and DNA repair. Note that in most unlinked experiments, peaks are absent from region 300–1200 and 1700–2500. This suggests that the nucleosomes anchored by formaldehyde cross-linking to their *in vivo* loci are positioned by chaperones or remodeling enzymes.
(1.21 MB TIF)

Figure S3 Extensive sliding/remodeling at the SMC5 (YOL034W) gene. SMC5 encodes a protein responsible for the structural maintenance of chromosomes, required for growth and DNA repair. Note that in most unlinked experiments, peaks are absent from region 300–1200 and 1700–2500. This suggests that the nucleosomes anchored by formaldehyde cross-linking to their *in vivo* loci are positioned by chaperones or remodeling enzymes.
(1.03 MB TIF)

Figure S4 The gene for the SRO77 protein with roles in exocytosis and cation homeostasis. Note the flat distribution of sequencing tag density at the center of the gene. Although clear summits are formed, most peaks are not separated from each other in the cross-linked experiments and in ACF1 reconstitutions.
(1.25 MB TIF)

Figure S5 Nucleosome reconstitutions from the purified DNA of the ovine beta-lactoglobulin gene and chicken erythrocyte histones (Fraser et al., 2009). These extremely high coverage experiments indicate fuzzy nucleosome positioning even in the absence of remodeling enzymes and histone chaperones.
(3.54 MB TIF)

Table S1 The presence of well-positioned (barrier) nucleosomes in the yeast genes. Experiments with well-positioned nucleosomes are shown for each gene. Most genes (including their flanking intergenic regions) contain one or more well-positioned nucleosome(s) in the majority if not all of the 13 Illumina experiments. Off-target transcriptional regulation is reduced by within-transcribed region “absorbers” of the momentum of histone sliding and remodeling. Cross-linked studies are color-coded by blue. Please refer to Figs. S1, S2, S3, S4 for graphical displays.
(2.74 MB PDF)
