# Supplementary figures and images for: Weakly Positioned Nucleosomes Enhance the Transcriptional Competency of Chromatin
Source: PLoS One. 2010 Sep 24;5(9):e12984. doi: 10.1371/journal.pone.0012984 (PMC2945322; doi:10.1371/journal.pone.0012984)

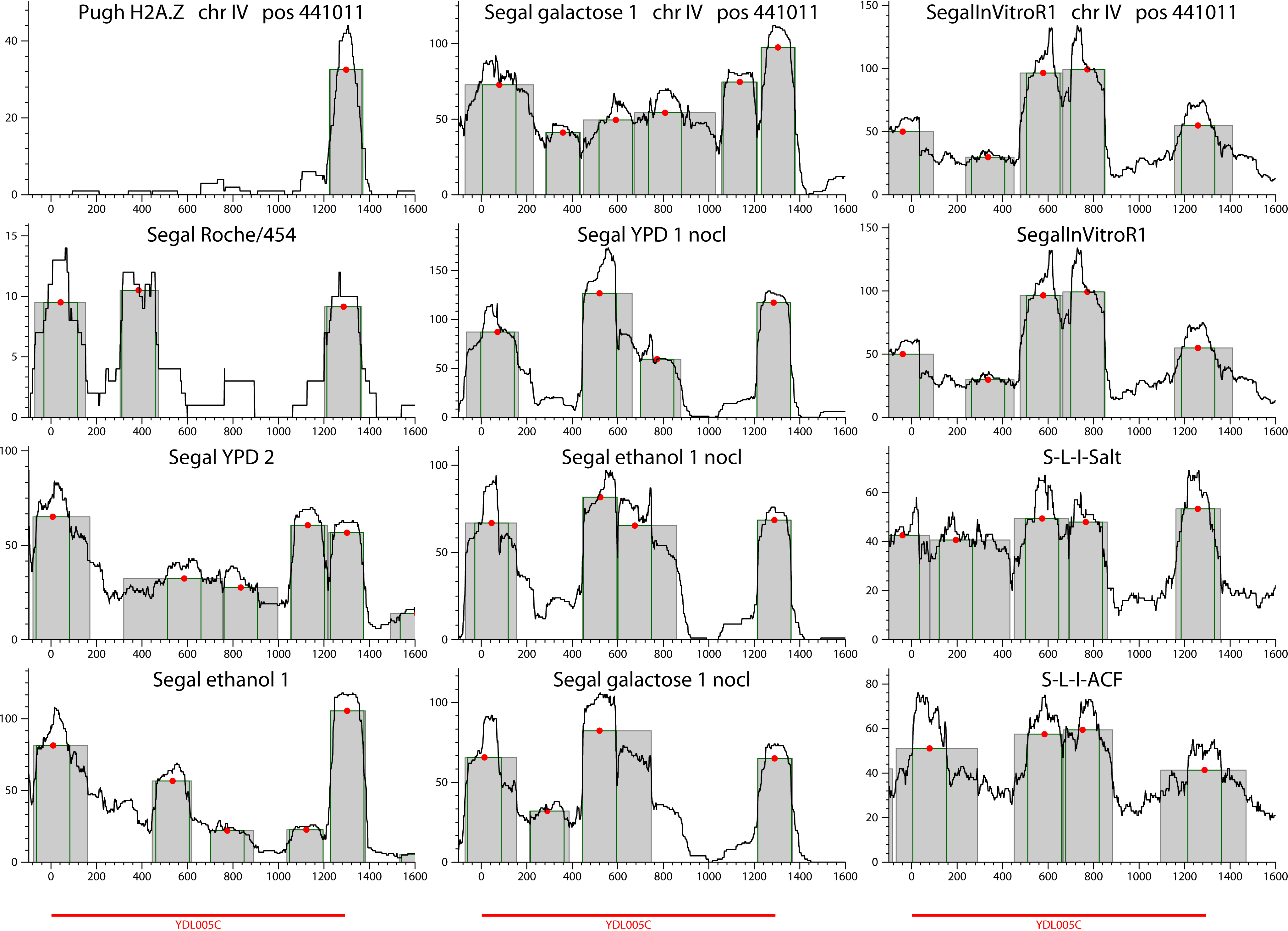

Supplement: Figure S1 — All nucleosomes are dynamic at the constitutively expressed MED2 (YDL005C) gene.In particular, the single nucleosome at relative position −40 and the twin peaks starting at relative position ∼450 are subject to extreme sliding or remodeling. The MED2 protein is a subunit of the RNA polymerase II mediator complex, and it is essential for transcriptional regulation. (0.95 MB TIF) [file pone.0012984.s001.tif]

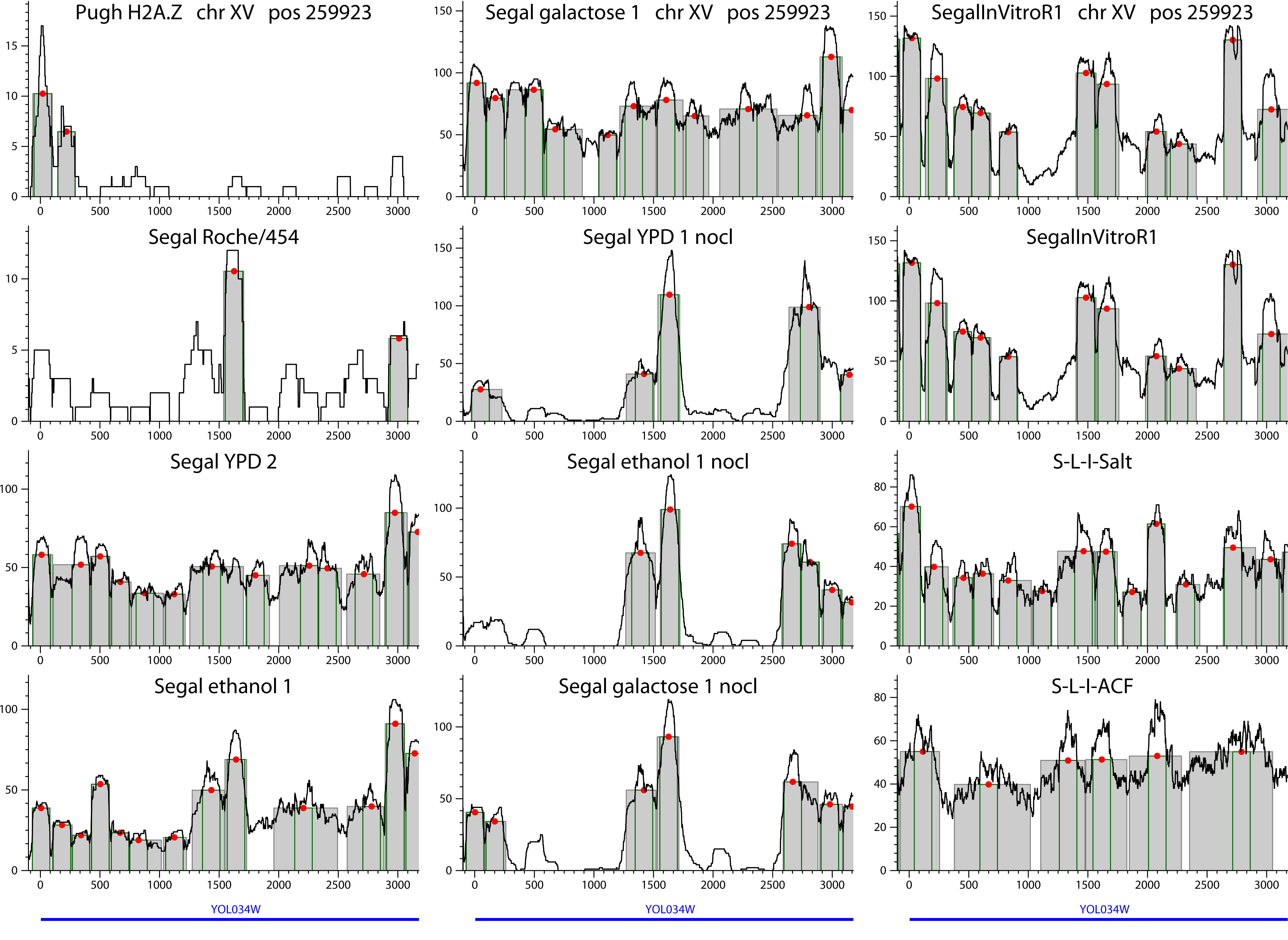

Supplement: Figure S2 — Extensive sliding/remodeling at the SMC5 (YOL034W) gene. SMC5 encodes a protein responsible for the structural maintenance of chromosomes, required for growth and DNA repair. Note that in most unlinked experiments, peaks are absent from region 300–1200 and 1700–2500. This suggests that the nucleosomes anchored by formaldehyde cross-linking to their in vivo loci are positioned by chaperones or remodeling enzymes. (1.21 MB TIF) [file pone.0012984.s002.tif]

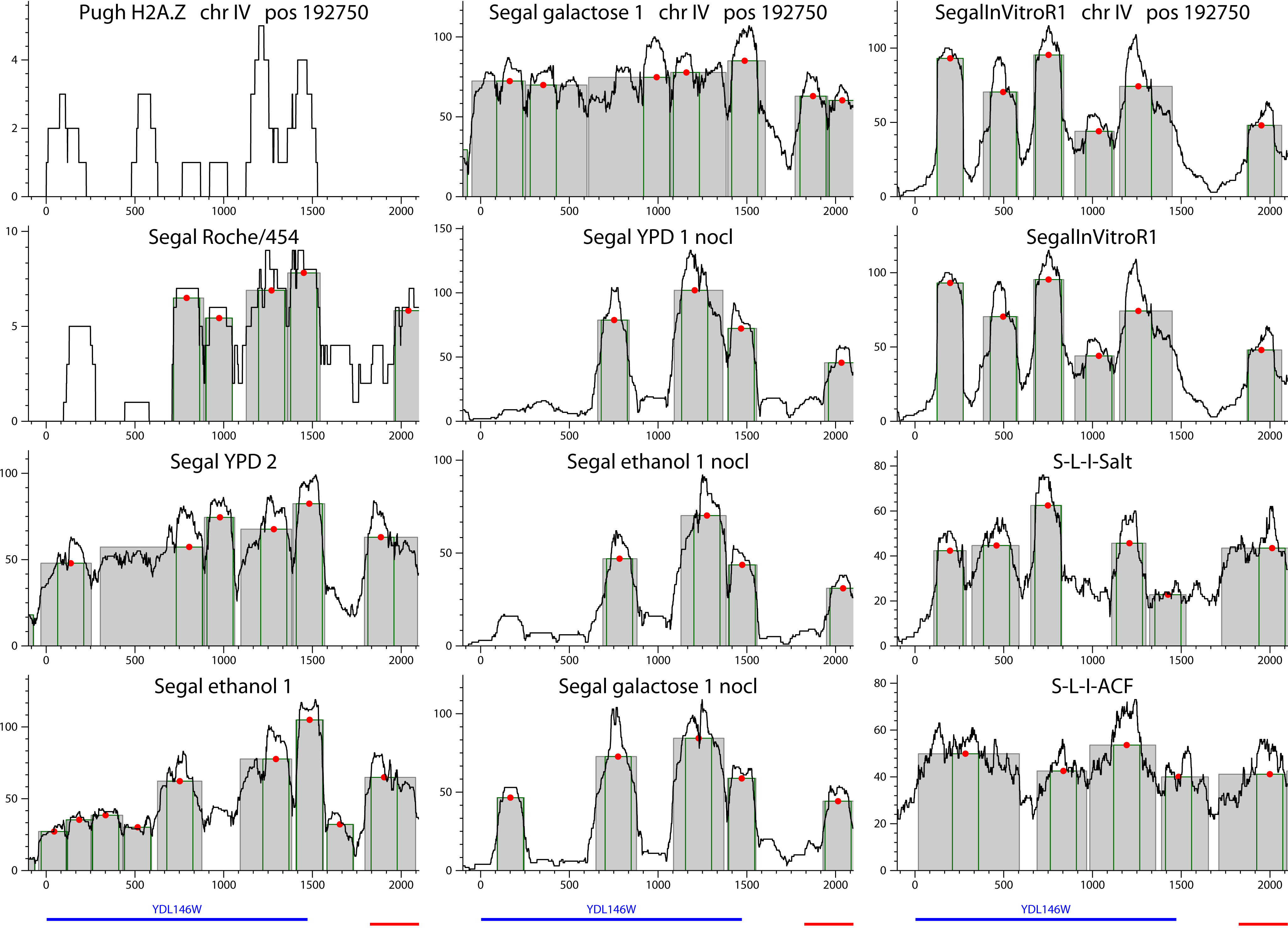

Supplement: Figure S3 — Extensive sliding/remodeling at the SMC5 (YOL034W) gene. SMC5 encodes a protein responsible for the structural maintenance of chromosomes, required for growth and DNA repair. Note that in most unlinked experiments, peaks are absent from region 300–1200 and 1700–2500. This suggests that the nucleosomes anchored by formaldehyde cross-linking to their in vivo loci are positioned by chaperones or remodeling enzymes. (1.03 MB TIF) [file pone.0012984.s003.tif]

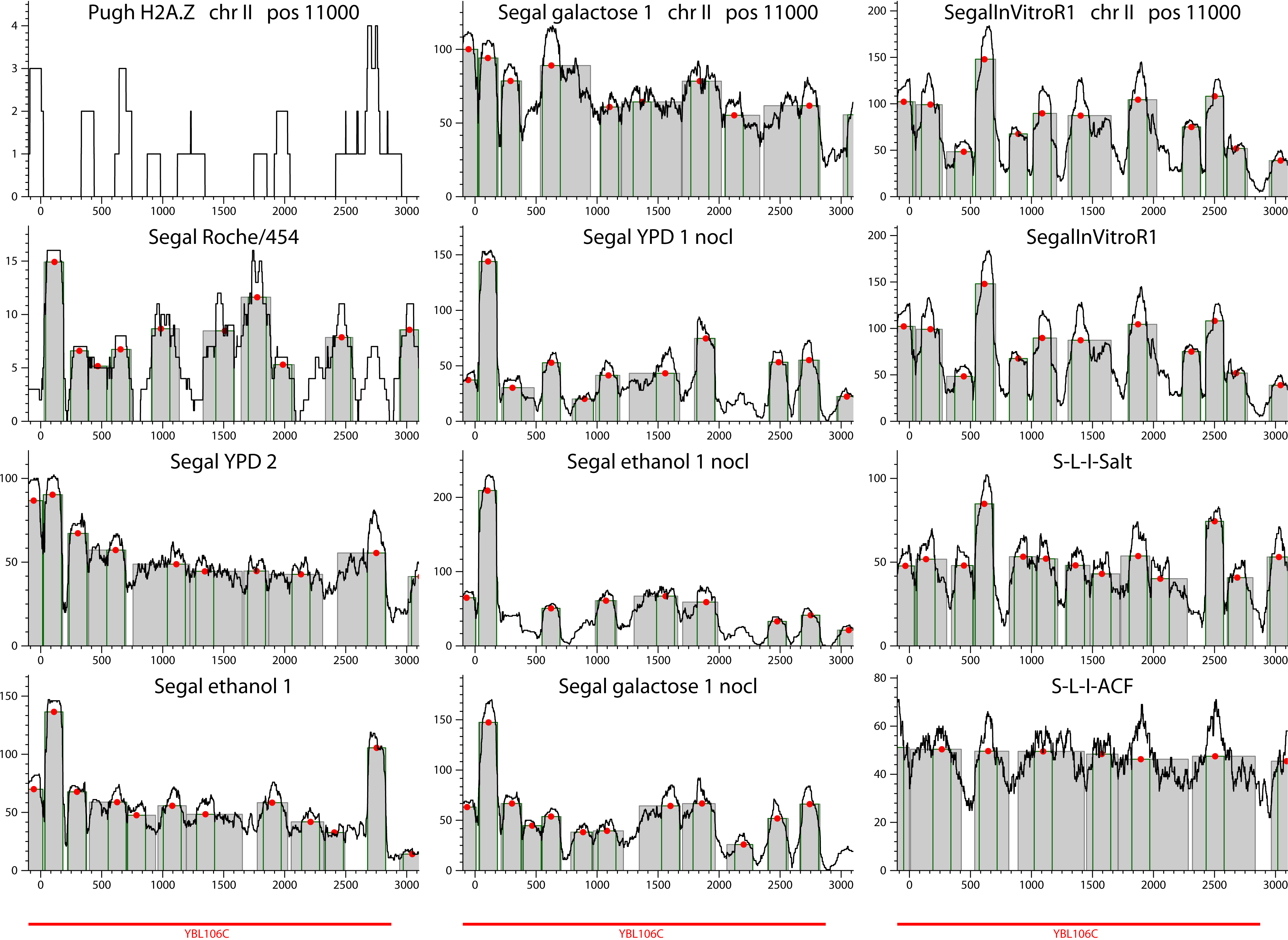

Supplement: Figure S4 — The gene for the SRO77 protein with roles in exocytosis and cation homeostasis. Note the flat distribution of sequencing tag density at the center of the gene. Although clear summits are formed, most peaks are not separated from each other in the cross-linked experiments and in ACF1 reconstitutions. (1.25 MB TIF) [file pone.0012984.s004.tif]

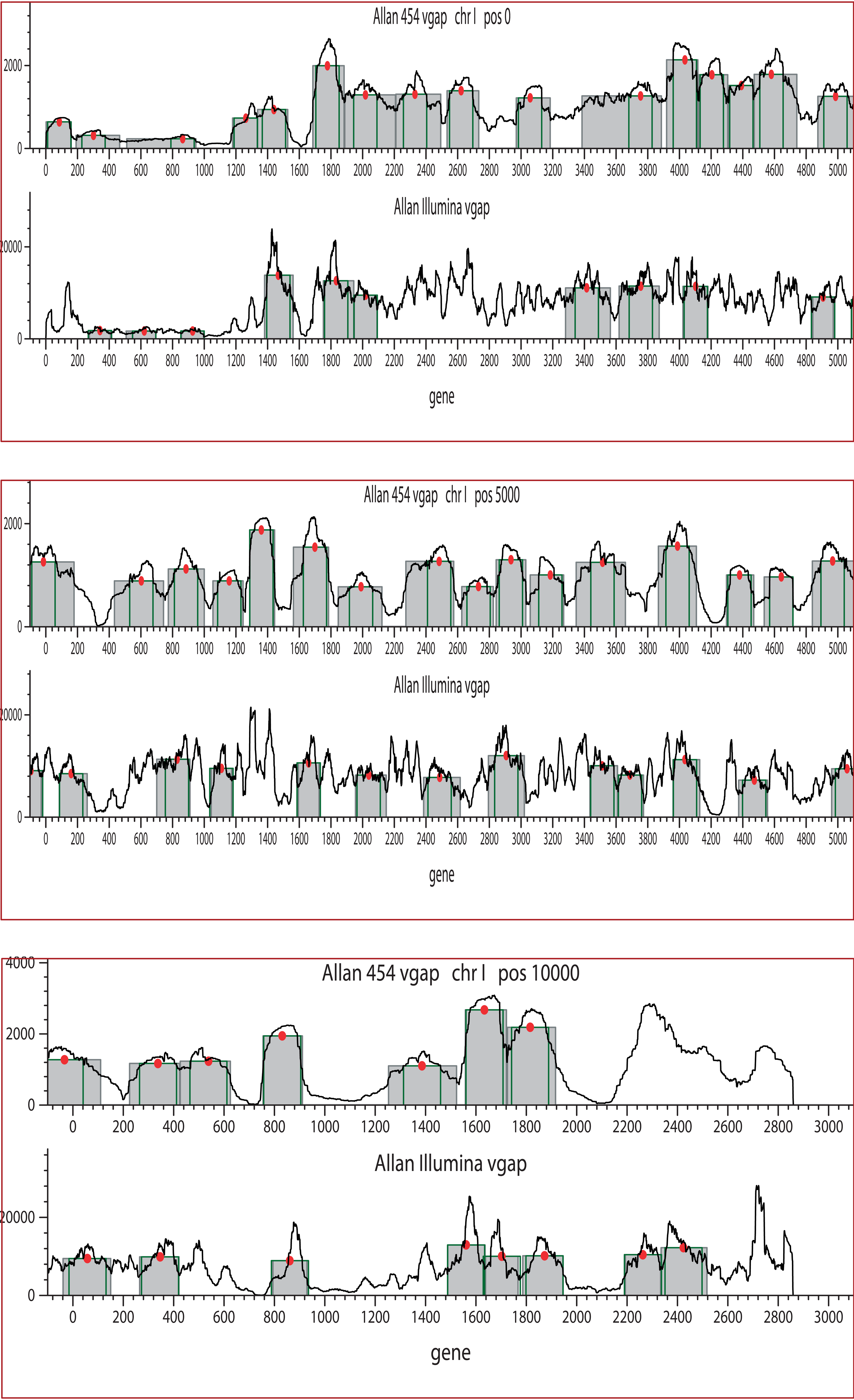

Supplement: Figure S5 — Nucleosome reconstitutions from the purified DNA of the ovine beta-lactoglobulin gene and chicken erythrocyte histones (Fraser et al., 2009). These extremely high coverage experiments indicate fuzzy nucleosome positioning even in the absence of remodeling enzymes and histone chaperones. (3.54 MB TIF) [file pone.0012984.s005.tif]
