# Supplementary material for: Weakly Positioned Nucleosomes Enhance the Transcriptional Competency of Chromatin
Source: PLoS One. 2010 Sep 24;5(9):e12984. doi: 10.1371/journal.pone.0012984 (PMC2945322; doi:10.1371/journal.pone.0012984)
Supplement: Table S1 — The presence of well-positioned (barrier) nucleosomes in the yeast genes. Experiments with well-positioned nucleosomes are shown for each gene. Most genes (including their flanking intergenic regions) contain one or more well-positioned nucleosome(s) in the majority if not all of the 13 Illumina experiments. Off-target transcriptional regulation is reduced by within-transcribed region “absorbers” of the momentum of histone sliding and remodeling. Cross-linked studies are color-coded by blue. Please refer to Figs. S1, S2, S3, S4 for graphical displays. (2.74 MB PDF) [file pone.0012984.s006.pdf]

Supplementary Table 2.

No. exps.	*Gene symbol*	*Accession*	*Experiments*												
			With cross-linking					*Without cross-linking*							
13	SGV1	YPR161C	YPDcl2	YPDcl3	EtOHcl1	EtOHcl2	Galcl1	YPDnc1	YPDnc2	YPDnc3	YPDnc4	EtOHnc1	EtOHnc2	Galnc1	Galnc2
13	RTC1	YOL138C	YPDcl2	YPDcl3	EtOHcl1	EtOHcl2	Galcl1	YPDnc1	YPDnc2	YPDnc3	YPDnc4	EtOHnc1	EtOHnc2	Galnc1	Galnc2
13	RHO1	YPR165W	YPDcl2	YPDcl3	EtOHcl1	EtOHcl2	Galcl1	YPDnc1	YPDnc2	YPDnc3	YPDnc4	EtOHnc1	EtOHnc2	Galnc1	Galnc2
13	HST2	YPL015C	YPDcl2	YPDcl3	EtOHcl1	EtOHcl2	Galcl1	YPDnc1	YPDnc2	YPDnc3	YPDnc4	EtOHnc1	EtOHnc2	Galnc1	Galnc2
13	APA1	YCL050C	YPDcl2	YPDcl3	EtOHcl1	EtOHcl2	Galcl1	YPDnc1	YPDnc2	YPDnc3	YPDnc4	EtOHnc1	EtOHnc2	Galnc1	Galnc2
13		YLR031W	YPDcl2	YPDcl3	EtOHcl1	EtOHcl2	Galcl1	YPDnc1	YPDnc2	YPDnc3	YPDnc4	EtOHnc1	EtOHnc2	Galnc1	Galnc2
13		YIL014C-A	YPDcl2	YPDcl3	EtOHcl1	EtOHcl2	Galcl1	YPDnc1	YPDnc2	YPDnc3	YPDnc4	EtOHnc1	EtOHnc2	Galnc1	Galnc2
13		YGR053C	YPDcl2	YPDcl3	EtOHcl1	EtOHcl2	Galcl1	YPDnc1	YPDnc2	YPDnc3	YPDnc4	EtOHnc1	EtOHnc2	Galnc1	Galnc2
13	SBH1	YER087C-B	YPDcl2	YPDcl3	EtOHcl1	EtOHcl2	Galcl1	YPDnc1	YPDnc2	YPDnc3	YPDnc4	EtOHnc1	EtOHnc2	Galnc1	Galnc2
13	FSH3	YOR280C	YPDcl2	YPDcl3	EtOHcl1	EtOHcl2	Galcl1	YPDnc1	YPDnc2	YPDnc3	YPDnc4	EtOHnc1	EtOHnc2	Galnc1	Galnc2
13	ASK10	YGR097W	YPDcl2	YPDcl3	EtOHcl1	EtOHcl2	Galcl1	YPDnc1	YPDnc2	YPDnc3	YPDnc4	EtOHnc1	EtOHnc2	Galnc1	Galnc2
13	PAN3	YKL025C	YPDcl2	YPDcl3	EtOHcl1	EtOHcl2	Galcl1	YPDnc1	YPDnc2	YPDnc3	YPDnc4	EtOHnc1	EtOHnc2	Galnc1	Galnc2
13		YKR011C	YPDcl2	YPDcl3	EtOHcl1	EtOHcl2	Galcl1	YPDnc1	YPDnc2	YPDnc3	YPDnc4	EtOHnc1	EtOHnc2	Galnc1	Galnc2
13	YEA4	YEL004W	YPDcl2	YPDcl3	EtOHcl1	EtOHcl2	Galcl1	YPDnc1	YPDnc2	YPDnc3	YPDnc4	EtOHnc1	EtOHnc2	Galnc1	Galnc2
13	ACO2	YJL200C	YPDcl2	YPDcl3	EtOHcl1	EtOHcl2	Galcl1	YPDnc1	YPDnc2	YPDnc3	YPDnc4	EtOHnc1	EtOHnc2	Galnc1	Galnc2
13	ISA1	YLL027W	YPDcl2	YPDcl3	EtOHcl1	EtOHcl2	Galcl1	YPDnc1	YPDnc2	YPDnc3	YPDnc4	EtOHnc1	EtOHnc2	Galnc1	Galnc2
13		YOR024W	YPDcl2	YPDcl3	EtOHcl1	EtOHcl2	Galcl1	YPDnc1	YPDnc2	YPDnc3	YPDnc4	EtOHnc1	EtOHnc2	Galnc1	Galnc2
13	STU2	YLR045C	YPDcl2	YPDcl3	EtOHcl1	EtOHcl2	Galcl1	YPDnc1	YPDnc2	YPDnc3	YPDnc4	EtOHnc1	EtOHnc2	Galnc1	Galnc2
13		YDR348C	YPDcl2	YPDcl3	EtOHcl1	EtOHcl2	Galcl1	YPDnc1	YPDnc2	YPDnc3	YPDnc4	EtOHnc1	EtOHnc2	Galnc1	Galnc2
13	CUL3	YGR003W	YPDcl2	YPDcl3	EtOHcl1	EtOHcl2	Galcl1	YPDnc1	YPDnc2	YPDnc3	YPDnc4	EtOHnc1	EtOHnc2	Galnc1	Galnc2
13		YLL007C	YPDcl2	YPDcl3	EtOHcl1	EtOHcl2	Galcl1	YPDnc1	YPDnc2	YPDnc3	YPDnc4	EtOHnc1	EtOHnc2	Galnc1	Galnc2
13	WHI5	YOR083W	YPDcl2	YPDcl3	EtOHcl1	EtOHcl2	Galcl1	YPDnc1	YPDnc2	YPDnc3	YPDnc4	EtOHnc1	EtOHnc2	Galnc1	Galnc2
13	EDC3	YEL015W	YPDcl2	YPDcl3	EtOHcl1	EtOHcl2	Galcl1	YPDnc1	YPDnc2	YPDnc3	YPDnc4	EtOHnc1	EtOHnc2	Galnc1	Galnc2
13	PEX3	YDR329C	YPDcl2	YPDcl3	EtOHcl1	EtOHcl2	Galcl1	YPDnc1	YPDnc2	YPDnc3	YPDnc4	EtOHnc1	EtOHnc2	Galnc1	Galnc2
13	DUT1	YBR252W	YPDcl2	YPDcl3	EtOHcl1	EtOHcl2	Galcl1	YPDnc1	YPDnc2	YPDnc3	YPDnc4	EtOHnc1	EtOHnc2	Galnc1	Galnc2
13	CRG1	YHR209W	YPDcl2	YPDcl3	EtOHcl1	EtOHcl2	Galcl1	YPDnc1	YPDnc2	YPDnc3	YPDnc4	EtOHnc1	EtOHnc2	Galnc1	Galnc2
13	PTP3	YER075C	YPDcl2	YPDcl3	EtOHcl1	EtOHcl2	Galcl1	YPDnc1	YPDnc2	YPDnc3	YPDnc4	EtOHnc1	EtOHnc2	Galnc1	Galnc2
13	ACK1	YDL203C	YPDcl2	YPDcl3	EtOHcl1	EtOHcl2	Galcl1	YPDnc1	YPDnc2	YPDnc3	YPDnc4	EtOHnc1	EtOHnc2	Galnc1	Galnc2
13	VTI1	YMR197C	YPDcl2	YPDcl3	EtOHcl1	EtOHcl2	Galcl1	YPDnc1	YPDnc2	YPDnc3	YPDnc4	EtOHnc1	EtOHnc2	Galnc1	Galnc2
13	GIP2	YER054C	YPDcl2	YPDcl3	EtOHcl1	EtOHcl2	Galcl1	YPDnc1	YPDnc2	YPDnc3	YPDnc4	EtOHnc1	EtOHnc2	Galnc1	Galnc2
13	HNT2	YDR305C	YPDcl2	YPDcl3	EtOHcl1	EtOHcl2	Galcl1	YPDnc1	YPDnc2	YPDnc3	YPDnc4	EtOHnc1	EtOHnc2	Galnc1	Galnc2

13		YHL019W-A	YPDcl2	YPDcl3	EtOHcl1	EtOHcl2	Galcl1	YPDnc1	YPDnc2	YPDnc3	YPDnc4	EtOHnc1	EtOHnc2	Galnc1	Galnc2
13	PSY2	YNL201C	YPDcl2	YPDcl3	EtOHcl1	EtOHcl2	Galcl1	YPDnc1	YPDnc2	YPDnc3	YPDnc4	EtOHnc1	EtOHnc2	Galnc1	Galnc2
13		YBR206W	YPDcl2	YPDcl3	EtOHcl1	EtOHcl2	Galcl1	YPDnc1	YPDnc2	YPDnc3	YPDnc4	EtOHnc1	EtOHnc2	Galnc1	Galnc2
13	OYE3	YPL171C	YPDcl2	YPDcl3	EtOHcl1	EtOHcl2	Galcl1	YPDnc1	YPDnc2	YPDnc3	YPDnc4	EtOHnc1	EtOHnc2	Galnc1	Galnc2
13	CDC36	YDL165W	YPDcl2	YPDcl3	EtOHcl1	EtOHcl2	Galcl1	YPDnc1	YPDnc2	YPDnc3	YPDnc4	EtOHnc1	EtOHnc2	Galnc1	Galnc2
13		YPL119C-A	YPDcl2	YPDcl3	EtOHcl1	EtOHcl2	Galcl1	YPDnc1	YPDnc2	YPDnc3	YPDnc4	EtOHnc1	EtOHnc2	Galnc1	Galnc2
13	SSL2	YIL143C	YPDcl2	YPDcl3	EtOHcl1	EtOHcl2	Galcl1	YPDnc1	YPDnc2	YPDnc3	YPDnc4	EtOHnc1	EtOHnc2	Galnc1	Galnc2
13		YBR292C	YPDcl2	YPDcl3	EtOHcl1	EtOHcl2	Galcl1	YPDnc1	YPDnc2	YPDnc3	YPDnc4	EtOHnc1	EtOHnc2	Galnc1	Galnc2
13	PFK1	YGR240C	YPDcl2	YPDcl3	EtOHcl1	EtOHcl2	Galcl1	YPDnc1	YPDnc2	YPDnc3	YPDnc4	EtOHnc1	EtOHnc2	Galnc1	Galnc2
13	TAF10	YDR167W	YPDcl2	YPDcl3	EtOHcl1	EtOHcl2	Galcl1	YPDnc1	YPDnc2	YPDnc3	YPDnc4	EtOHnc1	EtOHnc2	Galnc1	Galnc2
13	HOM3	YER052C	YPDcl2	YPDcl3	EtOHcl1	EtOHcl2	Galcl1	YPDnc1	YPDnc2	YPDnc3	YPDnc4	EtOHnc1	EtOHnc2	Galnc1	Galnc2
13	RPO26	YPR187W	YPDcl2	YPDcl3	EtOHcl1	EtOHcl2	Galcl1	YPDnc1	YPDnc2	YPDnc3	YPDnc4	EtOHnc1	EtOHnc2	Galnc1	Galnc2
13	PBP1	YGR178C	YPDcl2	YPDcl3	EtOHcl1	EtOHcl2	Galcl1	YPDnc1	YPDnc2	YPDnc3	YPDnc4	EtOHnc1	EtOHnc2	Galnc1	Galnc2
13	PPM2	YOL141W	YPDcl2	YPDcl3	EtOHcl1	EtOHcl2	Galcl1	YPDnc1	YPDnc2	YPDnc3	YPDnc4	EtOHnc1	EtOHnc2	Galnc1	Galnc2
13	MRPL36	YBR122C	YPDcl2	YPDcl3	EtOHcl1	EtOHcl2	Galcl1	YPDnc1	YPDnc2	YPDnc3	YPDnc4	EtOHnc1	EtOHnc2	Galnc1	Galnc2
13		YHR095W	YPDcl2	YPDcl3	EtOHcl1	EtOHcl2	Galcl1	YPDnc1	YPDnc2	YPDnc3	YPDnc4	EtOHnc1	EtOHnc2	Galnc1	Galnc2
13	PAF1	YBR279W	YPDcl2	YPDcl3	EtOHcl1	EtOHcl2	Galcl1	YPDnc1	YPDnc2	YPDnc3	YPDnc4	EtOHnc1	EtOHnc2	Galnc1	Galnc2
13	FMP42	YMR221C	YPDcl2	YPDcl3	EtOHcl1	EtOHcl2	Galcl1	YPDnc1	YPDnc2	YPDnc3	YPDnc4	EtOHnc1	EtOHnc2	Galnc1	Galnc2
13	BUD26	YDR241W	YPDcl2	YPDcl3	EtOHcl1	EtOHcl2	Galcl1	YPDnc1	YPDnc2	YPDnc3	YPDnc4	EtOHnc1	EtOHnc2	Galnc1	Galnc2
13	COM2	YER130C	YPDcl2	YPDcl3	EtOHcl1	EtOHcl2	Galcl1	YPDnc1	YPDnc2	YPDnc3	YPDnc4	EtOHnc1	EtOHnc2	Galnc1	Galnc2
13	CNS1	YBR155W	YPDcl2	YPDcl3	EtOHcl1	EtOHcl2	Galcl1	YPDnc1	YPDnc2	YPDnc3	YPDnc4	EtOHnc1	EtOHnc2	Galnc1	Galnc2
13	ESC2	YDR363W	YPDcl2	YPDcl3	EtOHcl1	EtOHcl2	Galcl1	YPDnc1	YPDnc2	YPDnc3	YPDnc4	EtOHnc1	EtOHnc2	Galnc1	Galnc2
13	ECM7	YLR443W	YPDcl2	YPDcl3	EtOHcl1	EtOHcl2	Galcl1	YPDnc1	YPDnc2	YPDnc3	YPDnc4	EtOHnc1	EtOHnc2	Galnc1	Galnc2
13	FUN19	YAL034C	YPDcl2	YPDcl3	EtOHcl1	EtOHcl2	Galcl1	YPDnc1	YPDnc2	YPDnc3	YPDnc4	EtOHnc1	EtOHnc2	Galnc1	Galnc2
13		YPR084W	YPDcl2	YPDcl3	EtOHcl1	EtOHcl2	Galcl1	YPDnc1	YPDnc2	YPDnc3	YPDnc4	EtOHnc1	EtOHnc2	Galnc1	Galnc2
13	FOL3	YMR113W	YPDcl2	YPDcl3	EtOHcl1	EtOHcl2	Galcl1	YPDnc1	YPDnc2	YPDnc3	YPDnc4	EtOHnc1	EtOHnc2	Galnc1	Galnc2
13		YBR184W	YPDcl2	YPDcl3	EtOHcl1	EtOHcl2	Galcl1	YPDnc1	YPDnc2	YPDnc3	YPDnc4	EtOHnc1	EtOHnc2	Galnc1	Galnc2
13	SIT1	YEL065W	YPDcl2	YPDcl3	EtOHcl1	EtOHcl2	Galcl1	YPDnc1	YPDnc2	YPDnc3	YPDnc4	EtOHnc1	EtOHnc2	Galnc1	Galnc2
13		YFR018C	YPDcl2	YPDcl3	EtOHcl1	EtOHcl2	Galcl1	YPDnc1	YPDnc2	YPDnc3	YPDnc4	EtOHnc1	EtOHnc2	Galnc1	Galnc2
13	CAP1	YKL007W	YPDcl2	YPDcl3	EtOHcl1	EtOHcl2	Galcl1	YPDnc1	YPDnc2	YPDnc3	YPDnc4	EtOHnc1	EtOHnc2	Galnc1	Galnc2
13	NOP6	YDL213C	YPDcl2	YPDcl3	EtOHcl1	EtOHcl2	Galcl1	YPDnc1	YPDnc2	YPDnc3	YPDnc4	EtOHnc1	EtOHnc2	Galnc1	Galnc2
13	SSN2	YDR443C	YPDcl2	YPDcl3	EtOHcl1	EtOHcl2	Galcl1	YPDnc1	YPDnc2	YPDnc3	YPDnc4	EtOHnc1	EtOHnc2	Galnc1	Galnc2
13	PUS4	YNL292W	YPDcl2	YPDcl3	EtOHcl1	EtOHcl2	Galcl1	YPDnc1	YPDnc2	YPDnc3	YPDnc4	EtOHnc1	EtOHnc2	Galnc1	Galnc2
13		YKL100C	YPDcl2	YPDcl3	EtOHcl1	EtOHcl2	Galcl1	YPDnc1	YPDnc2	YPDnc3	YPDnc4	EtOHnc1	EtOHnc2	Galnc1	Galnc2

13	ECM37	YIL146C	YPDcl2	YPDcl3	EtOHcl1	EtOHcl2	Galcl1	YPDnc1	YPDnc2	YPDnc3	YPDnc4	EtOHnc1	EtOHnc2	Galnc1	Galnc2
13	MGS1	YNL218W	YPDcl2	YPDcl3	EtOHcl1	EtOHcl2	Galcl1	YPDnc1	YPDnc2	YPDnc3	YPDnc4	EtOHnc1	EtOHnc2	Galnc1	Galnc2
13	QDR2	YIL121W	YPDcl2	YPDcl3	EtOHcl1	EtOHcl2	Galcl1	YPDnc1	YPDnc2	YPDnc3	YPDnc4	EtOHnc1	EtOHnc2	Galnc1	Galnc2
13	NRP1	YDL167C	YPDcl2	YPDcl3	EtOHcl1	EtOHcl2	Galcl1	YPDnc1	YPDnc2	YPDnc3	YPDnc4	EtOHnc1	EtOHnc2	Galnc1	Galnc2
13	THI6	YPL214C	YPDcl2	YPDcl3	EtOHcl1	EtOHcl2	Galcl1	YPDnc1	YPDnc2	YPDnc3	YPDnc4	EtOHnc1	EtOHnc2	Galnc1	Galnc2
13	ETC7	YOR228C	YPDcl2	YPDcl3	EtOHcl1	EtOHcl2	Galcl1	YPDnc1	YPDnc2	YPDnc3	YPDnc4	EtOHnc1	EtOHnc2	Galnc1	Galnc2
13	BIT2	YBR270C	YPDcl2	YPDcl3	EtOHcl1	EtOHcl2	Galcl1	YPDnc1	YPDnc2	YPDnc3	YPDnc4	EtOHnc1	EtOHnc2	Galnc1	Galnc2
13	PET54	YGR222W	YPDcl2	YPDcl3	EtOHcl1	EtOHcl2	Galcl1	YPDnc1	YPDnc2	YPDnc3	YPDnc4	EtOHnc1	EtOHnc2	Galnc1	Galnc2
13	CAN1	YEL063C	YPDcl2	YPDcl3	EtOHcl1	EtOHcl2	Galcl1	YPDnc1	YPDnc2	YPDnc3	YPDnc4	EtOHnc1	EtOHnc2	Galnc1	Galnc2
13		YML020W	YPDcl2	YPDcl3	EtOHcl1	EtOHcl2	Galcl1	YPDnc1	YPDnc2	YPDnc3	YPDnc4	EtOHnc1	EtOHnc2	Galnc1	Galnc2
13	SSO2	YMR183C	YPDcl2	YPDcl3	EtOHcl1	EtOHcl2	Galcl1	YPDnc1	YPDnc2	YPDnc3	YPDnc4	EtOHnc1	EtOHnc2	Galnc1	Galnc2
13	MOD5	YOR274W	YPDcl2	YPDcl3	EtOHcl1	EtOHcl2	Galcl1	YPDnc1	YPDnc2	YPDnc3	YPDnc4	EtOHnc1	EtOHnc2	Galnc1	Galnc2
13	LSM5	YER146W	YPDcl2	YPDcl3	EtOHcl1	EtOHcl2	Galcl1	YPDnc1	YPDnc2	YPDnc3	YPDnc4	EtOHnc1	EtOHnc2	Galnc1	Galnc2
13		YOL035C	YPDcl2	YPDcl3	EtOHcl1	EtOHcl2	Galcl1	YPDnc1	YPDnc2	YPDnc3	YPDnc4	EtOHnc1	EtOHnc2	Galnc1	Galnc2
13	HMG1	YML075C	YPDcl2	YPDcl3	EtOHcl1	EtOHcl2	Galcl1	YPDnc1	YPDnc2	YPDnc3	YPDnc4	EtOHnc1	EtOHnc2	Galnc1	Galnc2
13	PIG1	YLR273C	YPDcl2	YPDcl3	EtOHcl1	EtOHcl2	Galcl1	YPDnc1	YPDnc2	YPDnc3	YPDnc4	EtOHnc1	EtOHnc2	Galnc1	Galnc2
13	SAS4	YDR181C	YPDcl2	YPDcl3	EtOHcl1	EtOHcl2	Galcl1	YPDnc1	YPDnc2	YPDnc3	YPDnc4	EtOHnc1	EtOHnc2	Galnc1	Galnc2
13	SRM1	YGL097W	YPDcl2	YPDcl3	EtOHcl1	EtOHcl2	Galcl1	YPDnc1	YPDnc2	YPDnc3	YPDnc4	EtOHnc1	EtOHnc2	Galnc1	Galnc2
13		YCL056C	YPDcl2	YPDcl3	EtOHcl1	EtOHcl2	Galcl1	YPDnc1	YPDnc2	YPDnc3	YPDnc4	EtOHnc1	EtOHnc2	Galnc1	Galnc2
13		YMR242W-	YPDcl2	YPDcl3	EtOHcl1	EtOHcl2	Galcl1	YPDnc1	YPDnc2	YPDnc3	YPDnc4	EtOHnc1	EtOHnc2	Galnc1	Galnc2
13	MKK2	YPL140C	YPDcl2	YPDcl3	EtOHcl1	EtOHcl2	Galcl1	YPDnc1	YPDnc2	YPDnc3	YPDnc4	EtOHnc1	EtOHnc2	Galnc1	Galnc2
13	ACC1	YNR016C	YPDcl2	YPDcl3	EtOHcl1	EtOHcl2	Galcl1	YPDnc1	YPDnc2	YPDnc3	YPDnc4	EtOHnc1	EtOHnc2	Galnc1	Galnc2
13	YCK3	YER123W	YPDcl2	YPDcl3	EtOHcl1	EtOHcl2	Galcl1	YPDnc1	YPDnc2	YPDnc3	YPDnc4	EtOHnc1	EtOHnc2	Galnc1	Galnc2
13		YKL202W	YPDcl2	YPDcl3	EtOHcl1	EtOHcl2	Galcl1	YPDnc1	YPDnc2	YPDnc3	YPDnc4	EtOHnc1	EtOHnc2	Galnc1	Galnc2
13	LTE1	YAL024C	YPDcl2	YPDcl3	EtOHcl1	EtOHcl2	Galcl1	YPDnc1	YPDnc2	YPDnc3	YPDnc4	EtOHnc1	EtOHnc2	Galnc1	Galnc2
13		YDR396W	YPDcl2	YPDcl3	EtOHcl1	EtOHcl2	Galcl1	YPDnc1	YPDnc2	YPDnc3	YPDnc4	EtOHnc1	EtOHnc2	Galnc1	Galnc2
13	ARO3	YDR035W	YPDcl2	YPDcl3	EtOHcl1	EtOHcl2	Galcl1	YPDnc1	YPDnc2	YPDnc3	YPDnc4	EtOHnc1	EtOHnc2	Galnc1	Galnc2
13		YBL062W	YPDcl2	YPDcl3	EtOHcl1	EtOHcl2	Galcl1	YPDnc1	YPDnc2	YPDnc3	YPDnc4	EtOHnc1	EtOHnc2	Galnc1	Galnc2
13	PEX18	YHR160C	YPDcl2	YPDcl3	EtOHcl1	EtOHcl2	Galcl1	YPDnc1	YPDnc2	YPDnc3	YPDnc4	EtOHnc1	EtOHnc2	Galnc1	Galnc2
13	TFC4	YGR047C	YPDcl2	YPDcl3	EtOHcl1	EtOHcl2	Galcl1	YPDnc1	YPDnc2	YPDnc3	YPDnc4	EtOHnc1	EtOHnc2	Galnc1	Galnc2
13	UBP3	YER151C	YPDcl2	YPDcl3	EtOHcl1	EtOHcl2	Galcl1	YPDnc1	YPDnc2	YPDnc3	YPDnc4	EtOHnc1	EtOHnc2	Galnc1	Galnc2
13	DUR1,2	YBR208C	YPDcl2	YPDcl3	EtOHcl1	EtOHcl2	Galcl1	YPDnc1	YPDnc2	YPDnc3	YPDnc4	EtOHnc1	EtOHnc2	Galnc1	Galnc2
13	XYL2	YLR070C	YPDcl2	YPDcl3	EtOHcl1	EtOHcl2	Galcl1	YPDnc1	YPDnc2	YPDnc3	YPDnc4	EtOHnc1	EtOHnc2	Galnc1	Galnc2
13	RPC82	YPR190C	YPDcl2	YPDcl3	EtOHcl1	EtOHcl2	Galcl1	YPDnc1	YPDnc2	YPDnc3	YPDnc4	EtOHnc1	EtOHnc2	Galnc1	Galnc2

13	BUD9	YGR041W	YPDcl2	YPDcl3	EtOHcl1	EtOHcl2	Galcl1	YPDnc1	YPDnc2	YPDnc3	YPDnc4	EtOHnc1	EtOHnc2	Galnc1	Galnc2
13		YHR097C	YPDcl2	YPDcl3	EtOHcl1	EtOHcl2	Galcl1	YPDnc1	YPDnc2	YPDnc3	YPDnc4	EtOHnc1	EtOHnc2	Galnc1	Galnc2
13	TRR1	YDR353W	YPDcl2	YPDcl3	EtOHcl1	EtOHcl2	Galcl1	YPDnc1	YPDnc2	YPDnc3	YPDnc4	EtOHnc1	EtOHnc2	Galnc1	Galnc2
13	SGF11	YPL047W	YPDcl2	YPDcl3	EtOHcl1	EtOHcl2	Galcl1	YPDnc1	YPDnc2	YPDnc3	YPDnc4	EtOHnc1	EtOHnc2	Galnc1	Galnc2
13	ATR1	YML116W	YPDcl2	YPDcl3	EtOHcl1	EtOHcl2	Galcl1	YPDnc1	YPDnc2	YPDnc3	YPDnc4	EtOHnc1	EtOHnc2	Galnc1	Galnc2
13	BSC6	YOL137W	YPDcl2	YPDcl3	EtOHcl1	EtOHcl2	Galcl1	YPDnc1	YPDnc2	YPDnc3	YPDnc4	EtOHnc1	EtOHnc2	Galnc1	Galnc2
13	CDC43	YGL155W	YPDcl2	YPDcl3	EtOHcl1	EtOHcl2	Galcl1	YPDnc1	YPDnc2	YPDnc3	YPDnc4	EtOHnc1	EtOHnc2	Galnc1	Galnc2
13	YRA2	YKL214C	YPDcl2	YPDcl3	EtOHcl1	EtOHcl2	Galcl1	YPDnc1	YPDnc2	YPDnc3	YPDnc4	EtOHnc1	EtOHnc2	Galnc1	Galnc2
13	CCE1	YKL011C	YPDcl2	YPDcl3	EtOHcl1	EtOHcl2	Galcl1	YPDnc1	YPDnc2	YPDnc3	YPDnc4	EtOHnc1	EtOHnc2	Galnc1	Galnc2
13	KOG1	YHR186C	YPDcl2	YPDcl3	EtOHcl1	EtOHcl2	Galcl1	YPDnc1	YPDnc2	YPDnc3	YPDnc4	EtOHnc1	EtOHnc2	Galnc1	Galnc2
13	BUD4	YJR092W	YPDcl2	YPDcl3	EtOHcl1	EtOHcl2	Galcl1	YPDnc1	YPDnc2	YPDnc3	YPDnc4	EtOHnc1	EtOHnc2	Galnc1	Galnc2
13		YGL199C	YPDcl2	YPDcl3	EtOHcl1	EtOHcl2	Galcl1	YPDnc1	YPDnc2	YPDnc3	YPDnc4	EtOHnc1	EtOHnc2	Galnc1	Galnc2
13		YDR010C	YPDcl2	YPDcl3	EtOHcl1	EtOHcl2	Galcl1	YPDnc1	YPDnc2	YPDnc3	YPDnc4	EtOHnc1	EtOHnc2	Galnc1	Galnc2
13	COA2	YPL189C-A	YPDcl2	YPDcl3	EtOHcl1	EtOHcl2	Galcl1	YPDnc1	YPDnc2	YPDnc3	YPDnc4	EtOHnc1	EtOHnc2	Galnc1	Galnc2
13	CTP1	YBR291C	YPDcl2	YPDcl3	EtOHcl1	EtOHcl2	Galcl1	YPDnc1	YPDnc2	YPDnc3	YPDnc4	EtOHnc1	EtOHnc2	Galnc1	Galnc2
13		YBL059W	YPDcl2	YPDcl3	EtOHcl1	EtOHcl2	Galcl1	YPDnc1	YPDnc2	YPDnc3	YPDnc4	EtOHnc1	EtOHnc2	Galnc1	Galnc2
13	BOS1	YLR078C	YPDcl2	YPDcl3	EtOHcl1	EtOHcl2	Galcl1	YPDnc1	YPDnc2	YPDnc3	YPDnc4	EtOHnc1	EtOHnc2	Galnc1	Galnc2
13		YFL012W	YPDcl2	YPDcl3	EtOHcl1	EtOHcl2	Galcl1	YPDnc1	YPDnc2	YPDnc3	YPDnc4	EtOHnc1	EtOHnc2	Galnc1	Galnc2
13		YOR108C-A	YPDcl2	YPDcl3	EtOHcl1	EtOHcl2	Galcl1	YPDnc1	YPDnc2	YPDnc3	YPDnc4	EtOHnc1	EtOHnc2	Galnc1	Galnc2
13		YKL153W	YPDcl2	YPDcl3	EtOHcl1	EtOHcl2	Galcl1	YPDnc1	YPDnc2	YPDnc3	YPDnc4	EtOHnc1	EtOHnc2	Galnc1	Galnc2
13	MRPL9	YGR220C	YPDcl2	YPDcl3	EtOHcl1	EtOHcl2	Galcl1	YPDnc1	YPDnc2	YPDnc3	YPDnc4	EtOHnc1	EtOHnc2	Galnc1	Galnc2
13	HLJ1	YMR161W	YPDcl2	YPDcl3	EtOHcl1	EtOHcl2	Galcl1	YPDnc1	YPDnc2	YPDnc3	YPDnc4	EtOHnc1	EtOHnc2	Galnc1	Galnc2
13	TIR1	YER011W	YPDcl2	YPDcl3	EtOHcl1	EtOHcl2	Galcl1	YPDnc1	YPDnc2	YPDnc3	YPDnc4	EtOHnc1	EtOHnc2	Galnc1	Galnc2
13	SUT1	YGL162W	YPDcl2	YPDcl3	EtOHcl1	EtOHcl2	Galcl1	YPDnc1	YPDnc2	YPDnc3	YPDnc4	EtOHnc1	EtOHnc2	Galnc1	Galnc2
13	SLA2	YNL243W	YPDcl2	YPDcl3	EtOHcl1	EtOHcl2	Galcl1	YPDnc1	YPDnc2	YPDnc3	YPDnc4	EtOHnc1	EtOHnc2	Galnc1	Galnc2
13	ARC40	YBR234C	YPDcl2	YPDcl3	EtOHcl1	EtOHcl2	Galcl1	YPDnc1	YPDnc2	YPDnc3	YPDnc4	EtOHnc1	EtOHnc2	Galnc1	Galnc2
13	SLM2	YNL047C	YPDcl2	YPDcl3	EtOHcl1	EtOHcl2	Galcl1	YPDnc1	YPDnc2	YPDnc3	YPDnc4	EtOHnc1	EtOHnc2	Galnc1	Galnc2
13	DAK2	YFL053W	YPDcl2	YPDcl3	EtOHcl1	EtOHcl2	Galcl1	YPDnc1	YPDnc2	YPDnc3	YPDnc4	EtOHnc1	EtOHnc2	Galnc1	Galnc2
13		YOR200W	YPDcl2	YPDcl3	EtOHcl1	EtOHcl2	Galcl1	YPDnc1	YPDnc2	YPDnc3	YPDnc4	EtOHnc1	EtOHnc2	Galnc1	Galnc2
13	UTP30	YKR060W	YPDcl2	YPDcl3	EtOHcl1	EtOHcl2	Galcl1	YPDnc1	YPDnc2	YPDnc3	YPDnc4	EtOHnc1	EtOHnc2	Galnc1	Galnc2
13	ARR2	YPR200C	YPDcl2	YPDcl3	EtOHcl1	EtOHcl2	Galcl1	YPDnc1	YPDnc2	YPDnc3	YPDnc4	EtOHnc1	EtOHnc2	Galnc1	Galnc2
13	EST2	YLR318W	YPDcl2	YPDcl3	EtOHcl1	EtOHcl2	Galcl1	YPDnc1	YPDnc2	YPDnc3	YPDnc4	EtOHnc1	EtOHnc2	Galnc1	Galnc2
13	PEX28	YHR150W	YPDcl2	YPDcl3	EtOHcl1	EtOHcl2	Galcl1	YPDnc1	YPDnc2	YPDnc3	YPDnc4	EtOHnc1	EtOHnc2	Galnc1	Galnc2
13	CDC4	YFL009W	YPDcl2	YPDcl3	EtOHcl1	EtOHcl2	Galcl1	YPDnc1	YPDnc2	YPDnc3	YPDnc4	EtOHnc1	EtOHnc2	Galnc1	Galnc2

13	GIP1	YBR045C	YPDcl2	YPDcl3	EtOHcl1	EtOHcl2	Galcl1	YPDnc1	YPDnc2	YPDnc3	YPDnc4	EtOHnc1	EtOHnc2	Galnc1	Galnc2
13	MIA40	YKL195W	YPDcl2	YPDcl3	EtOHcl1	EtOHcl2	Galcl1	YPDnc1	YPDnc2	YPDnc3	YPDnc4	EtOHnc1	EtOHnc2	Galnc1	Galnc2
13		YPR127W	YPDcl2	YPDcl3	EtOHcl1	EtOHcl2	Galcl1	YPDnc1	YPDnc2	YPDnc3	YPDnc4	EtOHnc1	EtOHnc2	Galnc1	Galnc2
13	RAD9	YDR217C	YPDcl2	YPDcl3	EtOHcl1	EtOHcl2	Galcl1	YPDnc1	YPDnc2	YPDnc3	YPDnc4	EtOHnc1	EtOHnc2	Galnc1	Galnc2
13	TAF11	YML015C	YPDcl2	YPDcl3	EtOHcl1	EtOHcl2	Galcl1	YPDnc1	YPDnc2	YPDnc3	YPDnc4	EtOHnc1	EtOHnc2	Galnc1	Galnc2
13	VPS70	YJR126C	YPDcl2	YPDcl3	EtOHcl1	EtOHcl2	Galcl1	YPDnc1	YPDnc2	YPDnc3	YPDnc4	EtOHnc1	EtOHnc2	Galnc1	Galnc2
13	RCE1	YMR274C	YPDcl2	YPDcl3	EtOHcl1	EtOHcl2	Galcl1	YPDnc1	YPDnc2	YPDnc3	YPDnc4	EtOHnc1	EtOHnc2	Galnc1	Galnc2
13	PLB1	YMR008C	YPDcl2	YPDcl3	EtOHcl1	EtOHcl2	Galcl1	YPDnc1	YPDnc2	YPDnc3	YPDnc4	EtOHnc1	EtOHnc2	Galnc1	Galnc2
13		YHR125W	YPDcl2	YPDcl3	EtOHcl1	EtOHcl2	Galcl1	YPDnc1	YPDnc2	YPDnc3	YPDnc4	EtOHnc1	EtOHnc2	Galnc1	Galnc2
13	HO	YDL227C	YPDcl2	YPDcl3	EtOHcl1	EtOHcl2	Galcl1	YPDnc1	YPDnc2	YPDnc3	YPDnc4	EtOHnc1	EtOHnc2	Galnc1	Galnc2
13	SWS2	YNL081C	YPDcl2	YPDcl3	EtOHcl1	EtOHcl2	Galcl1	YPDnc1	YPDnc2	YPDnc3	YPDnc4	EtOHnc1	EtOHnc2	Galnc1	Galnc2
13		YDR532C	YPDcl2	YPDcl3	EtOHcl1	EtOHcl2	Galcl1	YPDnc1	YPDnc2	YPDnc3	YPDnc4	EtOHnc1	EtOHnc2	Galnc1	Galnc2
13		YNL319W	YPDcl2	YPDcl3	EtOHcl1	EtOHcl2	Galcl1	YPDnc1	YPDnc2	YPDnc3	YPDnc4	EtOHnc1	EtOHnc2	Galnc1	Galnc2
13		YBR113W	YPDcl2	YPDcl3	EtOHcl1	EtOHcl2	Galcl1	YPDnc1	YPDnc2	YPDnc3	YPDnc4	EtOHnc1	EtOHnc2	Galnc1	Galnc2
13		YJL043W	YPDcl2	YPDcl3	EtOHcl1	EtOHcl2	Galcl1	YPDnc1	YPDnc2	YPDnc3	YPDnc4	EtOHnc1	EtOHnc2	Galnc1	Galnc2
13	ALG13	YGL047W	YPDcl2	YPDcl3	EtOHcl1	EtOHcl2	Galcl1	YPDnc1	YPDnc2	YPDnc3	YPDnc4	EtOHnc1	EtOHnc2	Galnc1	Galnc2
13	SSK1	YLR006C	YPDcl2	YPDcl3	EtOHcl1	EtOHcl2	Galcl1	YPDnc1	YPDnc2	YPDnc3	YPDnc4	EtOHnc1	EtOHnc2	Galnc1	Galnc2
13	OCA5	YHL029C	YPDcl2	YPDcl3	EtOHcl1	EtOHcl2	Galcl1	YPDnc1	YPDnc2	YPDnc3	YPDnc4	EtOHnc1	EtOHnc2	Galnc1	Galnc2
13	PRI1	YIR008C	YPDcl2	YPDcl3	EtOHcl1	EtOHcl2	Galcl1	YPDnc1	YPDnc2	YPDnc3	YPDnc4	EtOHnc1	EtOHnc2	Galnc1	Galnc2
13		YBL006W-A	YPDcl2	YPDcl3	EtOHcl1	EtOHcl2	Galcl1	YPDnc1	YPDnc2	YPDnc3	YPDnc4	EtOHnc1	EtOHnc2	Galnc1	Galnc2
13	NCL1	YBL024W	YPDcl2	YPDcl3	EtOHcl1	EtOHcl2	Galcl1	YPDnc1	YPDnc2	YPDnc3	YPDnc4	EtOHnc1	EtOHnc2	Galnc1	Galnc2
13	HIM1	YDR317W	YPDcl2	YPDcl3	EtOHcl1	EtOHcl2	Galcl1	YPDnc1	YPDnc2	YPDnc3	YPDnc4	EtOHnc1	EtOHnc2	Galnc1	Galnc2
13	LOH1	YJL038C	YPDcl2	YPDcl3	EtOHcl1	EtOHcl2	Galcl1	YPDnc1	YPDnc2	YPDnc3	YPDnc4	EtOHnc1	EtOHnc2	Galnc1	Galnc2
13	NRG1	YDR043C	YPDcl2	YPDcl3	EtOHcl1	EtOHcl2	Galcl1	YPDnc1	YPDnc2	YPDnc3	YPDnc4	EtOHnc1	EtOHnc2	Galnc1	Galnc2
13	RPA43	YOR340C	YPDcl2	YPDcl3	EtOHcl1	EtOHcl2	Galcl1	YPDnc1	YPDnc2	YPDnc3	YPDnc4	EtOHnc1	EtOHnc2	Galnc1	Galnc2
13	MGR1	YCL044C	YPDcl2	YPDcl3	EtOHcl1	EtOHcl2	Galcl1	YPDnc1	YPDnc2	YPDnc3	YPDnc4	EtOHnc1	EtOHnc2	Galnc1	Galnc2
13	ROG3	YFR022W	YPDcl2	YPDcl3	EtOHcl1	EtOHcl2	Galcl1	YPDnc1	YPDnc2	YPDnc3	YPDnc4	EtOHnc1	EtOHnc2	Galnc1	Galnc2
13	RMD6	YEL072W	YPDcl2	YPDcl3	EtOHcl1	EtOHcl2	Galcl1	YPDnc1	YPDnc2	YPDnc3	YPDnc4	EtOHnc1	EtOHnc2	Galnc1	Galnc2
13	DSN1	YIR010W	YPDcl2	YPDcl3	EtOHcl1	EtOHcl2	Galcl1	YPDnc1	YPDnc2	YPDnc3	YPDnc4	EtOHnc1	EtOHnc2	Galnc1	Galnc2
13	HIR1	YBL008W	YPDcl2	YPDcl3	EtOHcl1	EtOHcl2	Galcl1	YPDnc1	YPDnc2	YPDnc3	YPDnc4	EtOHnc1	EtOHnc2	Galnc1	Galnc2
13		YMR178W	YPDcl2	YPDcl3	EtOHcl1	EtOHcl2	Galcl1	YPDnc1	YPDnc2	YPDnc3	YPDnc4	EtOHnc1	EtOHnc2	Galnc1	Galnc2
13	YSC83	YHR017W	YPDcl2	YPDcl3	EtOHcl1	EtOHcl2	Galcl1	YPDnc1	YPDnc2	YPDnc3	YPDnc4	EtOHnc1	EtOHnc2	Galnc1	Galnc2
13	SNO1	YMR095C	YPDcl2	YPDcl3	EtOHcl1	EtOHcl2	Galcl1	YPDnc1	YPDnc2	YPDnc3	YPDnc4	EtOHnc1	EtOHnc2	Galnc1	Galnc2
13	SIR1	YKR101W	YPDcl2	YPDcl3	EtOHcl1	EtOHcl2	Galcl1	YPDnc1	YPDnc2	YPDnc3	YPDnc4	EtOHnc1	EtOHnc2	Galnc1	Galnc2

13	AIM18	YHR198C	YPDcl2	YPDcl3	EtOHcl1	EtOHcl2	Galcl1	YPDnc1	YPDnc2	YPDnc3	YPDnc4	EtOHnc1	EtOHnc2	Galnc1	Galnc2
13	SKI7	YOR076C	YPDcl2	YPDcl3	EtOHcl1	EtOHcl2	Galcl1	YPDnc1	YPDnc2	YPDnc3	YPDnc4	EtOHnc1	EtOHnc2	Galnc1	Galnc2
13	ALG9	YNL219C	YPDcl2	YPDcl3	EtOHcl1	EtOHcl2	Galcl1	YPDnc1	YPDnc2	YPDnc3	YPDnc4	EtOHnc1	EtOHnc2	Galnc1	Galnc2
13	CAP2	YIL034C	YPDcl2	YPDcl3	EtOHcl1	EtOHcl2	Galcl1	YPDnc1	YPDnc2	YPDnc3	YPDnc4	EtOHnc1	EtOHnc2	Galnc1	Galnc2
13		YOR342C	YPDcl2	YPDcl3	EtOHcl1	EtOHcl2	Galcl1	YPDnc1	YPDnc2	YPDnc3	YPDnc4	EtOHnc1	EtOHnc2	Galnc1	Galnc2
13	NBA1	YOL070C	YPDcl2	YPDcl3	EtOHcl1	EtOHcl2	Galcl1	YPDnc1	YPDnc2	YPDnc3	YPDnc4	EtOHnc1	EtOHnc2	Galnc1	Galnc2
13		YLR124W	YPDcl2	YPDcl3	EtOHcl1	EtOHcl2	Galcl1	YPDnc1	YPDnc2	YPDnc3	YPDnc4	EtOHnc1	EtOHnc2	Galnc1	Galnc2
13	CBR1	YIL043C	YPDcl2	YPDcl3	EtOHcl1	EtOHcl2	Galcl1	YPDnc1	YPDnc2	YPDnc3	YPDnc4	EtOHnc1	EtOHnc2	Galnc1	Galnc2
13	DHH1	YDL160C	YPDcl2	YPDcl3	EtOHcl1	EtOHcl2	Galcl1	YPDnc1	YPDnc2	YPDnc3	YPDnc4	EtOHnc1	EtOHnc2	Galnc1	Galnc2
13	PHB2	YGR231C	YPDcl2	YPDcl3	EtOHcl1	EtOHcl2	Galcl1	YPDnc1	YPDnc2	YPDnc3	YPDnc4	EtOHnc1	EtOHnc2	Galnc1	Galnc2
13	HDA1	YNL021W	YPDcl2	YPDcl3	EtOHcl1	EtOHcl2	Galcl1	YPDnc1	YPDnc2	YPDnc3	YPDnc4	EtOHnc1	EtOHnc2	Galnc1	Galnc2
13	RSN1	YMR266W	YPDcl2	YPDcl3	EtOHcl1	EtOHcl2	Galcl1	YPDnc1	YPDnc2	YPDnc3	YPDnc4	EtOHnc1	EtOHnc2	Galnc1	Galnc2
13	CTT1	YGR088W	YPDcl2	YPDcl3	EtOHcl1	EtOHcl2	Galcl1	YPDnc1	YPDnc2	YPDnc3	YPDnc4	EtOHnc1	EtOHnc2	Galnc1	Galnc2
13	BUD31	YCR063W	YPDcl2	YPDcl3	EtOHcl1	EtOHcl2	Galcl1	YPDnc1	YPDnc2	YPDnc3	YPDnc4	EtOHnc1	EtOHnc2	Galnc1	Galnc2
13		YHR073W-[illegible]	YPDcl2	YPDcl3	EtOHcl1	EtOHcl2	Galcl1	YPDnc1	YPDnc2	YPDnc3	YPDnc4	EtOHnc1	EtOHnc2	Galnc1	Galnc2
13		YBR201C-A	YPDcl2	YPDcl3	EtOHcl1	EtOHcl2	Galcl1	YPDnc1	YPDnc2	YPDnc3	YPDnc4	EtOHnc1	EtOHnc2	Galnc1	Galnc2
13	OCA2	YNL056W	YPDcl2	YPDcl3	EtOHcl1	EtOHcl2	Galcl1	YPDnc1	YPDnc2	YPDnc3	YPDnc4	EtOHnc1	EtOHnc2	Galnc1	Galnc2
13	ATS1	YAL020C	YPDcl2	YPDcl3	EtOHcl1	EtOHcl2	Galcl1	YPDnc1	YPDnc2	YPDnc3	YPDnc4	EtOHnc1	EtOHnc2	Galnc1	Galnc2
13	MDS3	YGL197W	YPDcl2	YPDcl3	EtOHcl1	EtOHcl2	Galcl1	YPDnc1	YPDnc2	YPDnc3	YPDnc4	EtOHnc1	EtOHnc2	Galnc1	Galnc2
13	SOF1	YLL011W	YPDcl2	YPDcl3	EtOHcl1	EtOHcl2	Galcl1	YPDnc1	YPDnc2	YPDnc3	YPDnc4	EtOHnc1	EtOHnc2	Galnc1	Galnc2
13	AIF1	YNR074C	YPDcl2	YPDcl3	EtOHcl1	EtOHcl2	Galcl1	YPDnc1	YPDnc2	YPDnc3	YPDnc4	EtOHnc1	EtOHnc2	Galnc1	Galnc2
13	PDS1	YDR113C	YPDcl2	YPDcl3	EtOHcl1	EtOHcl2	Galcl1	YPDnc1	YPDnc2	YPDnc3	YPDnc4	EtOHnc1	EtOHnc2	Galnc1	Galnc2
13	RAD52	YML032C	YPDcl2	YPDcl3	EtOHcl1	EtOHcl2	Galcl1	YPDnc1	YPDnc2	YPDnc3	YPDnc4	EtOHnc1	EtOHnc2	Galnc1	Galnc2
13	CAF16	YFL028C	YPDcl2	YPDcl3	EtOHcl1	EtOHcl2	Galcl1	YPDnc1	YPDnc2	YPDnc3	YPDnc4	EtOHnc1	EtOHnc2	Galnc1	Galnc2
13	RIB5	YBR256C	YPDcl2	YPDcl3	EtOHcl1	EtOHcl2	Galcl1	YPDnc1	YPDnc2	YPDnc3	YPDnc4	EtOHnc1	EtOHnc2	Galnc1	Galnc2
13	RMI1	YPL024W	YPDcl2	YPDcl3	EtOHcl1	EtOHcl2	Galcl1	YPDnc1	YPDnc2	YPDnc3	YPDnc4	EtOHnc1	EtOHnc2	Galnc1	Galnc2
13	SIR3	YLR442C	YPDcl2	YPDcl3	EtOHcl1	EtOHcl2	Galcl1	YPDnc1	YPDnc2	YPDnc3	YPDnc4	EtOHnc1	EtOHnc2	Galnc1	Galnc2
13	PET111	YMR257C	YPDcl2	YPDcl3	EtOHcl1	EtOHcl2	Galcl1	YPDnc1	YPDnc2	YPDnc3	YPDnc4	EtOHnc1	EtOHnc2	Galnc1	Galnc2
13	SRP21	YKL122C	YPDcl2	YPDcl3	EtOHcl1	EtOHcl2	Galcl1	YPDnc1	YPDnc2	YPDnc3	YPDnc4	EtOHnc1	EtOHnc2	Galnc1	Galnc2
13	THI3	YDL080C	YPDcl2	YPDcl3	EtOHcl1	EtOHcl2	Galcl1	YPDnc1	YPDnc2	YPDnc3	YPDnc4	EtOHnc1	EtOHnc2	Galnc1	Galnc2
13	IRC22	YEL001C	YPDcl2	YPDcl3	EtOHcl1	EtOHcl2	Galcl1	YPDnc1	YPDnc2	YPDnc3	YPDnc4	EtOHnc1	EtOHnc2	Galnc1	Galnc2
13	NUT2	YPR168W	YPDcl2	YPDcl3	EtOHcl1	EtOHcl2	Galcl1	YPDnc1	YPDnc2	YPDnc3	YPDnc4	EtOHnc1	EtOHnc2	Galnc1	Galnc2
13		YLR374C	YPDcl2	YPDcl3	EtOHcl1	EtOHcl2	Galcl1	YPDnc1	YPDnc2	YPDnc3	YPDnc4	EtOHnc1	EtOHnc2	Galnc1	Galnc2
13	CTM1	YHR109W	YPDcl2	YPDcl3	EtOHcl1	EtOHcl2	Galcl1	YPDnc1	YPDnc2	YPDnc3	YPDnc4	EtOHnc1	EtOHnc2	Galnc1	Galnc2

13		YDL034W	YPDcl2	YPDcl3	EtOHcl1	EtOHcl2	Galcl1	YPDnc1	YPDnc2	YPDnc3	YPDnc4	EtOHnc1	EtOHnc2	Galnc1	Galnc2
13	FCY1	YPR062W	YPDcl2	YPDcl3	EtOHcl1	EtOHcl2	Galcl1	YPDnc1	YPDnc2	YPDnc3	YPDnc4	EtOHnc1	EtOHnc2	Galnc1	Galnc2
13	VPS34	YLR240W	YPDcl2	YPDcl3	EtOHcl1	EtOHcl2	Galcl1	YPDnc1	YPDnc2	YPDnc3	YPDnc4	EtOHnc1	EtOHnc2	Galnc1	Galnc2
13	SRB5	YGR104C	YPDcl2	YPDcl3	EtOHcl1	EtOHcl2	Galcl1	YPDnc1	YPDnc2	YPDnc3	YPDnc4	EtOHnc1	EtOHnc2	Galnc1	Galnc2
13	DAN2	YLR037C	YPDcl2	YPDcl3	EtOHcl1	EtOHcl2	Galcl1	YPDnc1	YPDnc2	YPDnc3	YPDnc4	EtOHnc1	EtOHnc2	Galnc1	Galnc2
13	DIT2	YDR402C	YPDcl2	YPDcl3	EtOHcl1	EtOHcl2	Galcl1	YPDnc1	YPDnc2	YPDnc3	YPDnc4	EtOHnc1	EtOHnc2	Galnc1	Galnc2
13	BRR1	YPR057W	YPDcl2	YPDcl3	EtOHcl1	EtOHcl2	Galcl1	YPDnc1	YPDnc2	YPDnc3	YPDnc4	EtOHnc1	EtOHnc2	Galnc1	Galnc2
13	RPL15A	YLR029C	YPDcl2	YPDcl3	EtOHcl1	EtOHcl2	Galcl1	YPDnc1	YPDnc2	YPDnc3	YPDnc4	EtOHnc1	EtOHnc2	Galnc1	Galnc2
13		YMR155W	YPDcl2	YPDcl3	EtOHcl1	EtOHcl2	Galcl1	YPDnc1	YPDnc2	YPDnc3	YPDnc4	EtOHnc1	EtOHnc2	Galnc1	Galnc2
13		YGR164W	YPDcl2	YPDcl3	EtOHcl1	EtOHcl2	Galcl1	YPDnc1	YPDnc2	YPDnc3	YPDnc4	EtOHnc1	EtOHnc2	Galnc1	Galnc2
13		YIL171W-A	YPDcl2	YPDcl3	EtOHcl1	EtOHcl2	Galcl1	YPDnc1	YPDnc2	YPDnc3	YPDnc4	EtOHnc1	EtOHnc2	Galnc1	Galnc2
13		YDR114C	YPDcl2	YPDcl3	EtOHcl1	EtOHcl2	Galcl1	YPDnc1	YPDnc2	YPDnc3	YPDnc4	EtOHnc1	EtOHnc2	Galnc1	Galnc2
13	AGA1	YNR044W	YPDcl2	YPDcl3	EtOHcl1	EtOHcl2	Galcl1	YPDnc1	YPDnc2	YPDnc3	YPDnc4	EtOHnc1	EtOHnc2	Galnc1	Galnc2
13	SUT2	YPR009W	YPDcl2	YPDcl3	EtOHcl1	EtOHcl2	Galcl1	YPDnc1	YPDnc2	YPDnc3	YPDnc4	EtOHnc1	EtOHnc2	Galnc1	Galnc2
13	TIF35	YDR429C	YPDcl2	YPDcl3	EtOHcl1	EtOHcl2	Galcl1	YPDnc1	YPDnc2	YPDnc3	YPDnc4	EtOHnc1	EtOHnc2	Galnc1	Galnc2
13	SSK2	YNR031C	YPDcl2	YPDcl3	EtOHcl1	EtOHcl2	Galcl1	YPDnc1	YPDnc2	YPDnc3	YPDnc4	EtOHnc1	EtOHnc2	Galnc1	Galnc2
13	STE12	YHR084W	YPDcl2	YPDcl3	EtOHcl1	EtOHcl2	Galcl1	YPDnc1	YPDnc2	YPDnc3	YPDnc4	EtOHnc1	EtOHnc2	Galnc1	Galnc2
13	UTP23	YOR004W	YPDcl2	YPDcl3	EtOHcl1	EtOHcl2	Galcl1	YPDnc1	YPDnc2	YPDnc3	YPDnc4	EtOHnc1	EtOHnc2	Galnc1	Galnc2
13	TRI1	YMR233W	YPDcl2	YPDcl3	EtOHcl1	EtOHcl2	Galcl1	YPDnc1	YPDnc2	YPDnc3	YPDnc4	EtOHnc1	EtOHnc2	Galnc1	Galnc2
13	PSR1	YLL010C	YPDcl2	YPDcl3	EtOHcl1	EtOHcl2	Galcl1	YPDnc1	YPDnc2	YPDnc3	YPDnc4	EtOHnc1	EtOHnc2	Galnc1	Galnc2
13	KAR1	YNL188W	YPDcl2	YPDcl3	EtOHcl1	EtOHcl2	Galcl1	YPDnc1	YPDnc2	YPDnc3	YPDnc4	EtOHnc1	EtOHnc2	Galnc1	Galnc2
13		YKL156C-A	YPDcl2	YPDcl3	EtOHcl1	EtOHcl2	Galcl1	YPDnc1	YPDnc2	YPDnc3	YPDnc4	EtOHnc1	EtOHnc2	Galnc1	Galnc2
13	EPS1	YIL005W	YPDcl2	YPDcl3	EtOHcl1	EtOHcl2	Galcl1	YPDnc1	YPDnc2	YPDnc3	YPDnc4	EtOHnc1	EtOHnc2	Galnc1	Galnc2
13	ALG12	YNR030W	YPDcl2	YPDcl3	EtOHcl1	EtOHcl2	Galcl1	YPDnc1	YPDnc2	YPDnc3	YPDnc4	EtOHnc1	EtOHnc2	Galnc1	Galnc2
13	TSC10	YBR265W	YPDcl2	YPDcl3	EtOHcl1	EtOHcl2	Galcl1	YPDnc1	YPDnc2	YPDnc3	YPDnc4	EtOHnc1	EtOHnc2	Galnc1	Galnc2
13	SNT2	YGL131C	YPDcl2	YPDcl3	EtOHcl1	EtOHcl2	Galcl1	YPDnc1	YPDnc2	YPDnc3	YPDnc4	EtOHnc1	EtOHnc2	Galnc1	Galnc2
13		YDL162C	YPDcl2	YPDcl3	EtOHcl1	EtOHcl2	Galcl1	YPDnc1	YPDnc2	YPDnc3	YPDnc4	EtOHnc1	EtOHnc2	Galnc1	Galnc2
13		YNR040W	YPDcl2	YPDcl3	EtOHcl1	EtOHcl2	Galcl1	YPDnc1	YPDnc2	YPDnc3	YPDnc4	EtOHnc1	EtOHnc2	Galnc1	Galnc2
13		YHR033W	YPDcl2	YPDcl3	EtOHcl1	EtOHcl2	Galcl1	YPDnc1	YPDnc2	YPDnc3	YPDnc4	EtOHnc1	EtOHnc2	Galnc1	Galnc2
13		YHL046W-A	YPDcl2	YPDcl3	EtOHcl1	EtOHcl2	Galcl1	YPDnc1	YPDnc2	YPDnc3	YPDnc4	EtOHnc1	EtOHnc2	Galnc1	Galnc2
13	FMP43	YGR243W	YPDcl2	YPDcl3	EtOHcl1	EtOHcl2	Galcl1	YPDnc1	YPDnc2	YPDnc3	YPDnc4	EtOHnc1	EtOHnc2	Galnc1	Galnc2
13	SEC1	YDR164C	YPDcl2	YPDcl3	EtOHcl1	EtOHcl2	Galcl1	YPDnc1	YPDnc2	YPDnc3	YPDnc4	EtOHnc1	EtOHnc2	Galnc1	Galnc2
13	RIM21	YNL294C	YPDcl2	YPDcl3	EtOHcl1	EtOHcl2	Galcl1	YPDnc1	YPDnc2	YPDnc3	YPDnc4	EtOHnc1	EtOHnc2	Galnc1	Galnc2
13	EGD2	YHR193C	YPDcl2	YPDcl3	EtOHcl1	EtOHcl2	Galcl1	YPDnc1	YPDnc2	YPDnc3	YPDnc4	EtOHnc1	EtOHnc2	Galnc1	Galnc2

13		YPR147C	YPDcl2	YPDcl3	EtOHcl1	EtOHcl2	Galcl1	YPDnc1	YPDnc2	YPDnc3	YPDnc4	EtOHnc1	EtOHnc2	Galnc1	Galnc2
13	PHO3	YBR092C	YPDcl2	YPDcl3	EtOHcl1	EtOHcl2	Galcl1	YPDnc1	YPDnc2	YPDnc3	YPDnc4	EtOHnc1	EtOHnc2	Galnc1	Galnc2
13	SSP2	YOR242C	YPDcl2	YPDcl3	EtOHcl1	EtOHcl2	Galcl1	YPDnc1	YPDnc2	YPDnc3	YPDnc4	EtOHnc1	EtOHnc2	Galnc1	Galnc2
13	FMP25	YLR077W	YPDcl2	YPDcl3	EtOHcl1	EtOHcl2	Galcl1	YPDnc1	YPDnc2	YPDnc3	YPDnc4	EtOHnc1	EtOHnc2	Galnc1	Galnc2
13	SRB8	YCR081W	YPDcl2	YPDcl3	EtOHcl1	EtOHcl2	Galcl1	YPDnc1	YPDnc2	YPDnc3	YPDnc4	EtOHnc1	EtOHnc2	Galnc1	Galnc2
13	SWI3	YJL176C	YPDcl2	YPDcl3	EtOHcl1	EtOHcl2	Galcl1	YPDnc1	YPDnc2	YPDnc3	YPDnc4	EtOHnc1	EtOHnc2	Galnc1	Galnc2
13	FUR1	YHR128W	YPDcl2	YPDcl3	EtOHcl1	EtOHcl2	Galcl1	YPDnc1	YPDnc2	YPDnc3	YPDnc4	EtOHnc1	EtOHnc2	Galnc1	Galnc2
13		YJR003C	YPDcl2	YPDcl3	EtOHcl1	EtOHcl2	Galcl1	YPDnc1	YPDnc2	YPDnc3	YPDnc4	EtOHnc1	EtOHnc2	Galnc1	Galnc2
13		YPR114W	YPDcl2	YPDcl3	EtOHcl1	EtOHcl2	Galcl1	YPDnc1	YPDnc2	YPDnc3	YPDnc4	EtOHnc1	EtOHnc2	Galnc1	Galnc2
13	GUP2	YPL189W	YPDcl2	YPDcl3	EtOHcl1	EtOHcl2	Galcl1	YPDnc1	YPDnc2	YPDnc3	YPDnc4	EtOHnc1	EtOHnc2	Galnc1	Galnc2
13	ZAP1	YJL056C	YPDcl2	YPDcl3	EtOHcl1	EtOHcl2	Galcl1	YPDnc1	YPDnc2	YPDnc3	YPDnc4	EtOHnc1	EtOHnc2	Galnc1	Galnc2
13		YPL247C	YPDcl2	YPDcl3	EtOHcl1	EtOHcl2	Galcl1	YPDnc1	YPDnc2	YPDnc3	YPDnc4	EtOHnc1	EtOHnc2	Galnc1	Galnc2
13	CWC21	YDR482C	YPDcl2	YPDcl3	EtOHcl1	EtOHcl2	Galcl1	YPDnc1	YPDnc2	YPDnc3	YPDnc4	EtOHnc1	EtOHnc2	Galnc1	Galnc2
13	ALB1	YJL122W	YPDcl2	YPDcl3	EtOHcl1	EtOHcl2	Galcl1	YPDnc1	YPDnc2	YPDnc3	YPDnc4	EtOHnc1	EtOHnc2	Galnc1	Galnc2
13	SHP1	YBL058W	YPDcl2	YPDcl3	EtOHcl1	EtOHcl2	Galcl1	YPDnc1	YPDnc2	YPDnc3	YPDnc4	EtOHnc1	EtOHnc2	Galnc1	Galnc2
13		YER085C	YPDcl2	YPDcl3	EtOHcl1	EtOHcl2	Galcl1	YPDnc1	YPDnc2	YPDnc3	YPDnc4	EtOHnc1	EtOHnc2	Galnc1	Galnc2
13	HCM1	YCR065W	YPDcl2	YPDcl3	EtOHcl1	EtOHcl2	Galcl1	YPDnc1	YPDnc2	YPDnc3	YPDnc4	EtOHnc1	EtOHnc2	Galnc1	Galnc2
13	SEC53	YFL045C	YPDcl2	YPDcl3	EtOHcl1	EtOHcl2	Galcl1	YPDnc1	YPDnc2	YPDnc3	YPDnc4	EtOHnc1	EtOHnc2	Galnc1	Galnc2
13		YPR091C	YPDcl2	YPDcl3	EtOHcl1	EtOHcl2	Galcl1	YPDnc1	YPDnc2	YPDnc3	YPDnc4	EtOHnc1	EtOHnc2	Galnc1	Galnc2
13		YOL083W	YPDcl2	YPDcl3	EtOHcl1	EtOHcl2	Galcl1	YPDnc1	YPDnc2	YPDnc3	YPDnc4	EtOHnc1	EtOHnc2	Galnc1	Galnc2
13		YLR307C-A	YPDcl2	YPDcl3	EtOHcl1	EtOHcl2	Galcl1	YPDnc1	YPDnc2	YPDnc3	YPDnc4	EtOHnc1	EtOHnc2	Galnc1	Galnc2
13	MET13	YGL125W	YPDcl2	YPDcl3	EtOHcl1	EtOHcl2	Galcl1	YPDnc1	YPDnc2	YPDnc3	YPDnc4	EtOHnc1	EtOHnc2	Galnc1	Galnc2
13	KAE1	YKR038C	YPDcl2	YPDcl3	EtOHcl1	EtOHcl2	Galcl1	YPDnc1	YPDnc2	YPDnc3	YPDnc4	EtOHnc1	EtOHnc2	Galnc1	Galnc2
13	GDH2	YDL215C	YPDcl2	YPDcl3	EtOHcl1	EtOHcl2	Galcl1	YPDnc1	YPDnc2	YPDnc3	YPDnc4	EtOHnc1	EtOHnc2	Galnc1	Galnc2
13	TAL1	YLR354C	YPDcl2	YPDcl3	EtOHcl1	EtOHcl2	Galcl1	YPDnc1	YPDnc2	YPDnc3	YPDnc4	EtOHnc1	EtOHnc2	Galnc1	Galnc2
13		YCR022C	YPDcl2	YPDcl3	EtOHcl1	EtOHcl2	Galcl1	YPDnc1	YPDnc2	YPDnc3	YPDnc4	EtOHnc1	EtOHnc2	Galnc1	Galnc2
13	FAR7	YFR008W	YPDcl2	YPDcl3	EtOHcl1	EtOHcl2	Galcl1	YPDnc1	YPDnc2	YPDnc3	YPDnc4	EtOHnc1	EtOHnc2	Galnc1	Galnc2
13	DPP1	YDR284C	YPDcl2	YPDcl3	EtOHcl1	EtOHcl2	Galcl1	YPDnc1	YPDnc2	YPDnc3	YPDnc4	EtOHnc1	EtOHnc2	Galnc1	Galnc2
13	SFK1	YKL051W	YPDcl2	YPDcl3	EtOHcl1	EtOHcl2	Galcl1	YPDnc1	YPDnc2	YPDnc3	YPDnc4	EtOHnc1	EtOHnc2	Galnc1	Galnc2
13		YPR174C	YPDcl2	YPDcl3	EtOHcl1	EtOHcl2	Galcl1	YPDnc1	YPDnc2	YPDnc3	YPDnc4	EtOHnc1	EtOHnc2	Galnc1	Galnc2
13	NPL6	YMR091C	YPDcl2	YPDcl3	EtOHcl1	EtOHcl2	Galcl1	YPDnc1	YPDnc2	YPDnc3	YPDnc4	EtOHnc1	EtOHnc2	Galnc1	Galnc2
13	IRC2	YDR112W	YPDcl2	YPDcl3	EtOHcl1	EtOHcl2	Galcl1	YPDnc1	YPDnc2	YPDnc3	YPDnc4	EtOHnc1	EtOHnc2	Galnc1	Galnc2
13	LST7	YGR057C	YPDcl2	YPDcl3	EtOHcl1	EtOHcl2	Galcl1	YPDnc1	YPDnc2	YPDnc3	YPDnc4	EtOHnc1	EtOHnc2	Galnc1	Galnc2
13	CDA2	YLR308W	YPDcl2	YPDcl3	EtOHcl1	EtOHcl2	Galcl1	YPDnc1	YPDnc2	YPDnc3	YPDnc4	EtOHnc1	EtOHnc2	Galnc1	Galnc2

13	ECM2	YBR065C	YPDcl2	YPDcl3	EtOHcl1	EtOHcl2	Galcl1	YPDnc1	YPDnc2	YPDnc3	YPDnc4	EtOHnc1	EtOHnc2	Galnc1	Galnc2
13	FAR8	YMR029C	YPDcl2	YPDcl3	EtOHcl1	EtOHcl2	Galcl1	YPDnc1	YPDnc2	YPDnc3	YPDnc4	EtOHnc1	EtOHnc2	Galnc1	Galnc2
13	THI22	YPR121W	YPDcl2	YPDcl3	EtOHcl1	EtOHcl2	Galcl1	YPDnc1	YPDnc2	YPDnc3	YPDnc4	EtOHnc1	EtOHnc2	Galnc1	Galnc2
13	ARI1	YGL157W	YPDcl2	YPDcl3	EtOHcl1	EtOHcl2	Galcl1	YPDnc1	YPDnc2	YPDnc3	YPDnc4	EtOHnc1	EtOHnc2	Galnc1	Galnc2
13		YMR148W	YPDcl2	YPDcl3	EtOHcl1	EtOHcl2	Galcl1	YPDnc1	YPDnc2	YPDnc3	YPDnc4	EtOHnc1	EtOHnc2	Galnc1	Galnc2
13	SML1	YML058W	YPDcl2	YPDcl3	EtOHcl1	EtOHcl2	Galcl1	YPDnc1	YPDnc2	YPDnc3	YPDnc4	EtOHnc1	EtOHnc2	Galnc1	Galnc2
13	UGA1	YGR019W	YPDcl2	YPDcl3	EtOHcl1	EtOHcl2	Galcl1	YPDnc1	YPDnc2	YPDnc3	YPDnc4	EtOHnc1	EtOHnc2	Galnc1	Galnc2
13	SHM1	YBR263W	YPDcl2	YPDcl3	EtOHcl1	EtOHcl2	Galcl1	YPDnc1	YPDnc2	YPDnc3	YPDnc4	EtOHnc1	EtOHnc2	Galnc1	Galnc2
13	APC5	YOR249C	YPDcl2	YPDcl3	EtOHcl1	EtOHcl2	Galcl1	YPDnc1	YPDnc2	YPDnc3	YPDnc4	EtOHnc1	EtOHnc2	Galnc1	Galnc2
13	HTL1	YCR020W-B	YPDcl2	YPDcl3	EtOHcl1	EtOHcl2	Galcl1	YPDnc1	YPDnc2	YPDnc3	YPDnc4	EtOHnc1	EtOHnc2	Galnc1	Galnc2
13	OPI11	YPR044C	YPDcl2	YPDcl3	EtOHcl1	EtOHcl2	Galcl1	YPDnc1	YPDnc2	YPDnc3	YPDnc4	EtOHnc1	EtOHnc2	Galnc1	Galnc2
13		YDR286C	YPDcl2	YPDcl3	EtOHcl1	EtOHcl2	Galcl1	YPDnc1	YPDnc2	YPDnc3	YPDnc4	EtOHnc1	EtOHnc2	Galnc1	Galnc2
13		YLR342W-A	YPDcl2	YPDcl3	EtOHcl1	EtOHcl2	Galcl1	YPDnc1	YPDnc2	YPDnc3	YPDnc4	EtOHnc1	EtOHnc2	Galnc1	Galnc2
13	POM34	YLR018C	YPDcl2	YPDcl3	EtOHcl1	EtOHcl2	Galcl1	YPDnc1	YPDnc2	YPDnc3	YPDnc4	EtOHnc1	EtOHnc2	Galnc1	Galnc2
13	PDS5	YMR076C	YPDcl2	YPDcl3	EtOHcl1	EtOHcl2	Galcl1	YPDnc1	YPDnc2	YPDnc3	YPDnc4	EtOHnc1	EtOHnc2	Galnc1	Galnc2
13	TUM1	YOR251C	YPDcl2	YPDcl3	EtOHcl1	EtOHcl2	Galcl1	YPDnc1	YPDnc2	YPDnc3	YPDnc4	EtOHnc1	EtOHnc2	Galnc1	Galnc2
13		YBR124W	YPDcl2	YPDcl3	EtOHcl1	EtOHcl2	Galcl1	YPDnc1	YPDnc2	YPDnc3	YPDnc4	EtOHnc1	EtOHnc2	Galnc1	Galnc2
13	TIM8	YJR135W-A	YPDcl2	YPDcl3	EtOHcl1	EtOHcl2	Galcl1	YPDnc1	YPDnc2	YPDnc3	YPDnc4	EtOHnc1	EtOHnc2	Galnc1	Galnc2
13	RRP8	YDR083W	YPDcl2	YPDcl3	EtOHcl1	EtOHcl2	Galcl1	YPDnc1	YPDnc2	YPDnc3	YPDnc4	EtOHnc1	EtOHnc2	Galnc1	Galnc2
13		YDR406W-A	YPDcl2	YPDcl3	EtOHcl1	EtOHcl2	Galcl1	YPDnc1	YPDnc2	YPDnc3	YPDnc4	EtOHnc1	EtOHnc2	Galnc1	Galnc2
13	SEC72	YLR292C	YPDcl2	YPDcl3	EtOHcl1	EtOHcl2	Galcl1	YPDnc1	YPDnc2	YPDnc3	YPDnc4	EtOHnc1	EtOHnc2	Galnc1	Galnc2
13	PER1	YCR044C	YPDcl2	YPDcl3	EtOHcl1	EtOHcl2	Galcl1	YPDnc1	YPDnc2	YPDnc3	YPDnc4	EtOHnc1	EtOHnc2	Galnc1	Galnc2
13	GIS1	YDR096W	YPDcl2	YPDcl3	EtOHcl1	EtOHcl2	Galcl1	YPDnc1	YPDnc2	YPDnc3	YPDnc4	EtOHnc1	EtOHnc2	Galnc1	Galnc2
13	RPS12	YOR369C	YPDcl2	YPDcl3	EtOHcl1	EtOHcl2	Galcl1	YPDnc1	YPDnc2	YPDnc3	YPDnc4	EtOHnc1	EtOHnc2	Galnc1	Galnc2
13	RUP1	YOR138C	YPDcl2	YPDcl3	EtOHcl1	EtOHcl2	Galcl1	YPDnc1	YPDnc2	YPDnc3	YPDnc4	EtOHnc1	EtOHnc2	Galnc1	Galnc2
13	BRF1	YGR246C	YPDcl2	YPDcl3	EtOHcl1	EtOHcl2	Galcl1	YPDnc1	YPDnc2	YPDnc3	YPDnc4	EtOHnc1	EtOHnc2	Galnc1	Galnc2
13	IML1	YJR138W	YPDcl2	YPDcl3	EtOHcl1	EtOHcl2	Galcl1	YPDnc1	YPDnc2	YPDnc3	YPDnc4	EtOHnc1	EtOHnc2	Galnc1	Galnc2
13	FYV4	YHR059W	YPDcl2	YPDcl3	EtOHcl1	EtOHcl2	Galcl1	YPDnc1	YPDnc2	YPDnc3	YPDnc4	EtOHnc1	EtOHnc2	Galnc1	Galnc2
13	NSG2	YNL156C	YPDcl2	YPDcl3	EtOHcl1	EtOHcl2	Galcl1	YPDnc1	YPDnc2	YPDnc3	YPDnc4	EtOHnc1	EtOHnc2	Galnc1	Galnc2
13	YPT53	YNL093W	YPDcl2	YPDcl3	EtOHcl1	EtOHcl2	Galcl1	YPDnc1	YPDnc2	YPDnc3	YPDnc4	EtOHnc1	EtOHnc2	Galnc1	Galnc2
13	APQ12	YIL040W	YPDcl2	YPDcl3	EtOHcl1	EtOHcl2	Galcl1	YPDnc1	YPDnc2	YPDnc3	YPDnc4	EtOHnc1	EtOHnc2	Galnc1	Galnc2
13	EMG1	YLR186W	YPDcl2	YPDcl3	EtOHcl1	EtOHcl2	Galcl1	YPDnc1	YPDnc2	YPDnc3	YPDnc4	EtOHnc1	EtOHnc2	Galnc1	Galnc2
13	PCL10	YGL134W	YPDcl2	YPDcl3	EtOHcl1	EtOHcl2	Galcl1	YPDnc1	YPDnc2	YPDnc3	YPDnc4	EtOHnc1	EtOHnc2	Galnc1	Galnc2
13	RPL43B	YJR094W-A	YPDcl2	YPDcl3	EtOHcl1	EtOHcl2	Galcl1	YPDnc1	YPDnc2	YPDnc3	YPDnc4	EtOHnc1	EtOHnc2	Galnc1	Galnc2

13	SSD1	YDR293C	YPDcl2	YPDcl3	EtOHcl1	EtOHcl2	Galcl1	YPDnc1	YPDnc2	YPDnc3	YPDnc4	EtOHnc1	EtOHnc2	Galnc1	Galnc2
13	SMM1	YNR015W	YPDcl2	YPDcl3	EtOHcl1	EtOHcl2	Galcl1	YPDnc1	YPDnc2	YPDnc3	YPDnc4	EtOHnc1	EtOHnc2	Galnc1	Galnc2
13		YIL166C	YPDcl2	YPDcl3	EtOHcl1	EtOHcl2	Galcl1	YPDnc1	YPDnc2	YPDnc3	YPDnc4	EtOHnc1	EtOHnc2	Galnc1	Galnc2
13		YER184C	YPDcl2	YPDcl3	EtOHcl1	EtOHcl2	Galcl1	YPDnc1	YPDnc2	YPDnc3	YPDnc4	EtOHnc1	EtOHnc2	Galnc1	Galnc2
13		YJL047C-A	YPDcl2	YPDcl3	EtOHcl1	EtOHcl2	Galcl1	YPDnc1	YPDnc2	YPDnc3	YPDnc4	EtOHnc1	EtOHnc2	Galnc1	Galnc2
13	TOA1	YOR194C	YPDcl2	YPDcl3	EtOHcl1	EtOHcl2	Galcl1	YPDnc1	YPDnc2	YPDnc3	YPDnc4	EtOHnc1	EtOHnc2	Galnc1	Galnc2
13	YJU2	YKL095W	YPDcl2	YPDcl3	EtOHcl1	EtOHcl2	Galcl1	YPDnc1	YPDnc2	YPDnc3	YPDnc4	EtOHnc1	EtOHnc2	Galnc1	Galnc2
13	SRB2	YHR041C	YPDcl2	YPDcl3	EtOHcl1	EtOHcl2	Galcl1	YPDnc1	YPDnc2	YPDnc3	YPDnc4	EtOHnc1	EtOHnc2	Galnc1	Galnc2
13	RTC2	YBR147W	YPDcl2	YPDcl3	EtOHcl1	EtOHcl2	Galcl1	YPDnc1	YPDnc2	YPDnc3	YPDnc4	EtOHnc1	EtOHnc2	Galnc1	Galnc2
13	MSH4	YFL003C	YPDcl2	YPDcl3	EtOHcl1	EtOHcl2	Galcl1	YPDnc1	YPDnc2	YPDnc3	YPDnc4	EtOHnc1	EtOHnc2	Galnc1	Galnc2
13	RPL18A	YOL120C	YPDcl2	YPDcl3	EtOHcl1	EtOHcl2	Galcl1	YPDnc1	YPDnc2	YPDnc3	YPDnc4	EtOHnc1	EtOHnc2	Galnc1	Galnc2
13	CAT5	YOR125C	YPDcl2	YPDcl3	EtOHcl1	EtOHcl2	Galcl1	YPDnc1	YPDnc2	YPDnc3	YPDnc4	EtOHnc1	EtOHnc2	Galnc1	Galnc2
13		YLR065C	YPDcl2	YPDcl3	EtOHcl1	EtOHcl2	Galcl1	YPDnc1	YPDnc2	YPDnc3	YPDnc4	EtOHnc1	EtOHnc2	Galnc1	Galnc2
13	ODC1	YPL134C	YPDcl2	YPDcl3	EtOHcl1	EtOHcl2	Galcl1	YPDnc1	YPDnc2	YPDnc3	YPDnc4	EtOHnc1	EtOHnc2	Galnc1	Galnc2
13	VRG4	YGL225W	YPDcl2	YPDcl3	EtOHcl1	EtOHcl2	Galcl1	YPDnc1	YPDnc2	YPDnc3	YPDnc4	EtOHnc1	EtOHnc2	Galnc1	Galnc2
13		YNL122C	YPDcl2	YPDcl3	EtOHcl1	EtOHcl2	Galcl1	YPDnc1	YPDnc2	YPDnc3	YPDnc4	EtOHnc1	EtOHnc2	Galnc1	Galnc2
13	SQS1	YNL224C	YPDcl2	YPDcl3	EtOHcl1	EtOHcl2	Galcl1	YPDnc1	YPDnc2	YPDnc3	YPDnc4	EtOHnc1	EtOHnc2	Galnc1	Galnc2
13		YNL217W	YPDcl2	YPDcl3	EtOHcl1	EtOHcl2	Galcl1	YPDnc1	YPDnc2	YPDnc3	YPDnc4	EtOHnc1	EtOHnc2	Galnc1	Galnc2
13	AIM31	YML030W	YPDcl2	YPDcl3	EtOHcl1	EtOHcl2	Galcl1	YPDnc1	YPDnc2	YPDnc3	YPDnc4	EtOHnc1	EtOHnc2	Galnc1	Galnc2
13	CYC3	YAL039C	YPDcl2	YPDcl3	EtOHcl1	EtOHcl2	Galcl1	YPDnc1	YPDnc2	YPDnc3	YPDnc4	EtOHnc1	EtOHnc2	Galnc1	Galnc2
13	RPN8	YOR261C	YPDcl2	YPDcl3	EtOHcl1	EtOHcl2	Galcl1	YPDnc1	YPDnc2	YPDnc3	YPDnc4	EtOHnc1	EtOHnc2	Galnc1	Galnc2
13	NPC2	YDL046W	YPDcl2	YPDcl3	EtOHcl1	EtOHcl2	Galcl1	YPDnc1	YPDnc2	YPDnc3	YPDnc4	EtOHnc1	EtOHnc2	Galnc1	Galnc2
13	SAP30	YMR263W	YPDcl2	YPDcl3	EtOHcl1	EtOHcl2	Galcl1	YPDnc1	YPDnc2	YPDnc3	YPDnc4	EtOHnc1	EtOHnc2	Galnc1	Galnc2
13	SKM1	YOL113W	YPDcl2	YPDcl3	EtOHcl1	EtOHcl2	Galcl1	YPDnc1	YPDnc2	YPDnc3	YPDnc4	EtOHnc1	EtOHnc2	Galnc1	Galnc2
13	GRH1	YDR517W	YPDcl2	YPDcl3	EtOHcl1	EtOHcl2	Galcl1	YPDnc1	YPDnc2	YPDnc3	YPDnc4	EtOHnc1	EtOHnc2	Galnc1	Galnc2
13	TFC3	YAL001C	YPDcl2	YPDcl3	EtOHcl1	EtOHcl2	Galcl1	YPDnc1	YPDnc2	YPDnc3	YPDnc4	EtOHnc1	EtOHnc2	Galnc1	Galnc2
13		YBR197C	YPDcl2	YPDcl3	EtOHcl1	EtOHcl2	Galcl1	YPDnc1	YPDnc2	YPDnc3	YPDnc4	EtOHnc1	EtOHnc2	Galnc1	Galnc2
13	NMD5	YJR132W	YPDcl2	YPDcl3	EtOHcl1	EtOHcl2	Galcl1	YPDnc1	YPDnc2	YPDnc3	YPDnc4	EtOHnc1	EtOHnc2	Galnc1	Galnc2
13	RPN3	YER021W	YPDcl2	YPDcl3	EtOHcl1	EtOHcl2	Galcl1	YPDnc1	YPDnc2	YPDnc3	YPDnc4	EtOHnc1	EtOHnc2	Galnc1	Galnc2
13	URB1	YKL014C	YPDcl2	YPDcl3	EtOHcl1	EtOHcl2	Galcl1	YPDnc1	YPDnc2	YPDnc3	YPDnc4	EtOHnc1	EtOHnc2	Galnc1	Galnc2
13		YGL034C	YPDcl2	YPDcl3	EtOHcl1	EtOHcl2	Galcl1	YPDnc1	YPDnc2	YPDnc3	YPDnc4	EtOHnc1	EtOHnc2	Galnc1	Galnc2
13	SNG1	YGR197C	YPDcl2	YPDcl3	EtOHcl1	EtOHcl2	Galcl1	YPDnc1	YPDnc2	YPDnc3	YPDnc4	EtOHnc1	EtOHnc2	Galnc1	Galnc2
13	PHO2	YDL106C	YPDcl2	YPDcl3	EtOHcl1	EtOHcl2	Galcl1	YPDnc1	YPDnc2	YPDnc3	YPDnc4	EtOHnc1	EtOHnc2	Galnc1	Galnc2
13	ERG24	YNL280C	YPDcl2	YPDcl3	EtOHcl1	EtOHcl2	Galcl1	YPDnc1	YPDnc2	YPDnc3	YPDnc4	EtOHnc1	EtOHnc2	Galnc1	Galnc2

13	PSP1	YDR505C	YPDcl2	YPDcl3	EtOHcl1	EtOHcl2	Galcl1	YPDnc1	YPDnc2	YPDnc3	YPDnc4	EtOHnc1	EtOHnc2	Galnc1	Galnc2
13	SYP1	YCR030C	YPDcl2	YPDcl3	EtOHcl1	EtOHcl2	Galcl1	YPDnc1	YPDnc2	YPDnc3	YPDnc4	EtOHnc1	EtOHnc2	Galnc1	Galnc2
13	ARO8	YGL202W	YPDcl2	YPDcl3	EtOHcl1	EtOHcl2	Galcl1	YPDnc1	YPDnc2	YPDnc3	YPDnc4	EtOHnc1	EtOHnc2	Galnc1	Galnc2
13		YBL036C	YPDcl2	YPDcl3	EtOHcl1	EtOHcl2	Galcl1	YPDnc1	YPDnc2	YPDnc3	YPDnc4	EtOHnc1	EtOHnc2	Galnc1	Galnc2
13		YBR221W-/	YPDcl2	YPDcl3	EtOHcl1	EtOHcl2	Galcl1	YPDnc1	YPDnc2	YPDnc3	YPDnc4	EtOHnc1	EtOHnc2	Galnc1	Galnc2
13	PEX11	YOL147C	YPDcl2	YPDcl3	EtOHcl1	EtOHcl2	Galcl1	YPDnc1	YPDnc2	YPDnc3	YPDnc4	EtOHnc1	EtOHnc2	Galnc1	Galnc2
13	YFH7	YFR007W	YPDcl2	YPDcl3	EtOHcl1	EtOHcl2	Galcl1	YPDnc1	YPDnc2	YPDnc3	YPDnc4	EtOHnc1	EtOHnc2	Galnc1	Galnc2
13	RPL16B	YNL069C	YPDcl2	YPDcl3	EtOHcl1	EtOHcl2	Galcl1	YPDnc1	YPDnc2	YPDnc3	YPDnc4	EtOHnc1	EtOHnc2	Galnc1	Galnc2
13	SRB4	YER022W	YPDcl2	YPDcl3	EtOHcl1	EtOHcl2	Galcl1	YPDnc1	YPDnc2	YPDnc3	YPDnc4	EtOHnc1	EtOHnc2	Galnc1	Galnc2
13		YJR008W	YPDcl2	YPDcl3	EtOHcl1	EtOHcl2	Galcl1	YPDnc1	YPDnc2	YPDnc3	YPDnc4	EtOHnc1	EtOHnc2	Galnc1	Galnc2
13	JEN1	YKL217W	YPDcl2	YPDcl3	EtOHcl1	EtOHcl2	Galcl1	YPDnc1	YPDnc2	YPDnc3	YPDnc4	EtOHnc1	EtOHnc2	Galnc1	Galnc2
13	AIM13	YFR011C	YPDcl2	YPDcl3	EtOHcl1	EtOHcl2	Galcl1	YPDnc1	YPDnc2	YPDnc3	YPDnc4	EtOHnc1	EtOHnc2	Galnc1	Galnc2
13	RPL34A	YER056C-A	YPDcl2	YPDcl3	EtOHcl1	EtOHcl2	Galcl1	YPDnc1	YPDnc2	YPDnc3	YPDnc4	EtOHnc1	EtOHnc2	Galnc1	Galnc2
13	CST26	YBR042C	YPDcl2	YPDcl3	EtOHcl1	EtOHcl2	Galcl1	YPDnc1	YPDnc2	YPDnc3	YPDnc4	EtOHnc1	EtOHnc2	Galnc1	Galnc2
13	SPC72	YAL047C	YPDcl2	YPDcl3	EtOHcl1	EtOHcl2	Galcl1	YPDnc1	YPDnc2	YPDnc3	YPDnc4	EtOHnc1	EtOHnc2	Galnc1	Galnc2
13	NOP7	YGR103W	YPDcl2	YPDcl3	EtOHcl1	EtOHcl2	Galcl1	YPDnc1	YPDnc2	YPDnc3	YPDnc4	EtOHnc1	EtOHnc2	Galnc1	Galnc2
13		YLR171W	YPDcl2	YPDcl3	EtOHcl1	EtOHcl2	Galcl1	YPDnc1	YPDnc2	YPDnc3	YPDnc4	EtOHnc1	EtOHnc2	Galnc1	Galnc2
13	PLP2	YOR281C	YPDcl2	YPDcl3	EtOHcl1	EtOHcl2	Galcl1	YPDnc1	YPDnc2	YPDnc3	YPDnc4	EtOHnc1	EtOHnc2	Galnc1	Galnc2
13	TOS1	YBR162C	YPDcl2	YPDcl3	EtOHcl1	EtOHcl2	Galcl1	YPDnc1	YPDnc2	YPDnc3	YPDnc4	EtOHnc1	EtOHnc2	Galnc1	Galnc2
13		YMR114C	YPDcl2	YPDcl3	EtOHcl1	EtOHcl2	Galcl1	YPDnc1	YPDnc2	YPDnc3	YPDnc4	EtOHnc1	EtOHnc2	Galnc1	Galnc2
13		YJL067W	YPDcl2	YPDcl3	EtOHcl1	EtOHcl2	Galcl1	YPDnc1	YPDnc2	YPDnc3	YPDnc4	EtOHnc1	EtOHnc2	Galnc1	Galnc2
13		YGR251W	YPDcl2	YPDcl3	EtOHcl1	EtOHcl2	Galcl1	YPDnc1	YPDnc2	YPDnc3	YPDnc4	EtOHnc1	EtOHnc2	Galnc1	Galnc2
13		YDR118W-/	YPDcl2	YPDcl3	EtOHcl1	EtOHcl2	Galcl1	YPDnc1	YPDnc2	YPDnc3	YPDnc4	EtOHnc1	EtOHnc2	Galnc1	Galnc2
13	PBY1	YBR094W	YPDcl2	YPDcl3	EtOHcl1	EtOHcl2	Galcl1	YPDnc1	YPDnc2	YPDnc3	YPDnc4	EtOHnc1	EtOHnc2	Galnc1	Galnc2
13	NAG1	YGR031C-A	YPDcl2	YPDcl3	EtOHcl1	EtOHcl2	Galcl1	YPDnc1	YPDnc2	YPDnc3	YPDnc4	EtOHnc1	EtOHnc2	Galnc1	Galnc2
13	HFM1	YGL251C	YPDcl2	YPDcl3	EtOHcl1	EtOHcl2	Galcl1	YPDnc1	YPDnc2	YPDnc3	YPDnc4	EtOHnc1	EtOHnc2	Galnc1	Galnc2
13	PMP2	YEL017C-A	YPDcl2	YPDcl3	EtOHcl1	EtOHcl2	Galcl1	YPDnc1	YPDnc2	YPDnc3	YPDnc4	EtOHnc1	EtOHnc2	Galnc1	Galnc2
13	MAK11	YKL021C	YPDcl2	YPDcl3	EtOHcl1	EtOHcl2	Galcl1	YPDnc1	YPDnc2	YPDnc3	YPDnc4	EtOHnc1	EtOHnc2	Galnc1	Galnc2
13	SAP190	YKR028W	YPDcl2	YPDcl3	EtOHcl1	EtOHcl2	Galcl1	YPDnc1	YPDnc2	YPDnc3	YPDnc4	EtOHnc1	EtOHnc2	Galnc1	Galnc2
13		YNR062C	YPDcl2	YPDcl3	EtOHcl1	EtOHcl2	Galcl1	YPDnc1	YPDnc2	YPDnc3	YPDnc4	EtOHnc1	EtOHnc2	Galnc1	Galnc2
13		YHL026C	YPDcl2	YPDcl3	EtOHcl1	EtOHcl2	Galcl1	YPDnc1	YPDnc2	YPDnc3	YPDnc4	EtOHnc1	EtOHnc2	Galnc1	Galnc2
13	PET117	YER058W	YPDcl2	YPDcl3	EtOHcl1	EtOHcl2	Galcl1	YPDnc1	YPDnc2	YPDnc3	YPDnc4	EtOHnc1	EtOHnc2	Galnc1	Galnc2
13		YCR016W	YPDcl2	YPDcl3	EtOHcl1	EtOHcl2	Galcl1	YPDnc1	YPDnc2	YPDnc3	YPDnc4	EtOHnc1	EtOHnc2	Galnc1	Galnc2
13	PEA2	YER149C	YPDcl2	YPDcl3	EtOHcl1	EtOHcl2	Galcl1	YPDnc1	YPDnc2	YPDnc3	YPDnc4	EtOHnc1	EtOHnc2	Galnc1	Galnc2

13		YJL147C	YPDcl2	YPDcl3	EtOHcl1	EtOHcl2	Galcl1	YPDnc1	YPDnc2	YPDnc3	YPDnc4	EtOHnc1	EtOHnc2	Galnc1	Galnc2
13	EDC1	YGL222C	YPDcl2	YPDcl3	EtOHcl1	EtOHcl2	Galcl1	YPDnc1	YPDnc2	YPDnc3	YPDnc4	EtOHnc1	EtOHnc2	Galnc1	Galnc2
13	RAM1	YDL090C	YPDcl2	YPDcl3	EtOHcl1	EtOHcl2	Galcl1	YPDnc1	YPDnc2	YPDnc3	YPDnc4	EtOHnc1	EtOHnc2	Galnc1	Galnc2
13		YOR034C-A	YPDcl2	YPDcl3	EtOHcl1	EtOHcl2	Galcl1	YPDnc1	YPDnc2	YPDnc3	YPDnc4	EtOHnc1	EtOHnc2	Galnc1	Galnc2
13	MNT4	YNR059W	YPDcl2	YPDcl3	EtOHcl1	EtOHcl2	Galcl1	YPDnc1	YPDnc2	YPDnc3	YPDnc4	EtOHnc1	EtOHnc2	Galnc1	Galnc2
13	SPC3	YLR066W	YPDcl2	YPDcl3	EtOHcl1	EtOHcl2	Galcl1	YPDnc1	YPDnc2	YPDnc3	YPDnc4	EtOHnc1	EtOHnc2	Galnc1	Galnc2
13	PTC4	YBR125C	YPDcl2	YPDcl3	EtOHcl1	EtOHcl2	Galcl1	YPDnc1	YPDnc2	YPDnc3	YPDnc4	EtOHnc1	EtOHnc2	Galnc1	Galnc2
13		YOR376W	YPDcl2	YPDcl3	EtOHcl1	EtOHcl2	Galcl1	YPDnc1	YPDnc2	YPDnc3	YPDnc4	EtOHnc1	EtOHnc2	Galnc1	Galnc2
13		YCL023C	YPDcl2	YPDcl3	EtOHcl1	EtOHcl2	Galcl1	YPDnc1	YPDnc2	YPDnc3	YPDnc4	EtOHnc1	EtOHnc2	Galnc1	Galnc2
13	UMP1	YBR173C	YPDcl2	YPDcl3	EtOHcl1	EtOHcl2	Galcl1	YPDnc1	YPDnc2	YPDnc3	YPDnc4	EtOHnc1	EtOHnc2	Galnc1	Galnc2
13	CPR2	YHR057C	YPDcl2	YPDcl3	EtOHcl1	EtOHcl2	Galcl1	YPDnc1	YPDnc2	YPDnc3	YPDnc4	EtOHnc1	EtOHnc2	Galnc1	Galnc2
13	RTS2	YOR077W	YPDcl2	YPDcl3	EtOHcl1	EtOHcl2	Galcl1	YPDnc1	YPDnc2	YPDnc3	YPDnc4	EtOHnc1	EtOHnc2	Galnc1	Galnc2
13	OAF1	YAL051W	YPDcl2	YPDcl3	EtOHcl1	EtOHcl2	Galcl1	YPDnc1	YPDnc2	YPDnc3	YPDnc4	EtOHnc1	EtOHnc2	Galnc1	Galnc2
13		YJR124C	YPDcl2	YPDcl3	EtOHcl1	EtOHcl2	Galcl1	YPDnc1	YPDnc2	YPDnc3	YPDnc4	EtOHnc1	EtOHnc2	Galnc1	Galnc2
13	CDC12	YHR107C	YPDcl2	YPDcl3	EtOHcl1	EtOHcl2	Galcl1	YPDnc1	YPDnc2	YPDnc3	YPDnc4	EtOHnc1	EtOHnc2	Galnc1	Galnc2
13	LSM4	YER112W	YPDcl2	YPDcl3	EtOHcl1	EtOHcl2	Galcl1	YPDnc1	YPDnc2	YPDnc3	YPDnc4	EtOHnc1	EtOHnc2	Galnc1	Galnc2
13	IDP2	YLR174W	YPDcl2	YPDcl3	EtOHcl1	EtOHcl2	Galcl1	YPDnc1	YPDnc2	YPDnc3	YPDnc4	EtOHnc1	EtOHnc2	Galnc1	Galnc2
13	FRM2	YCL026C-A	YPDcl2	YPDcl3	EtOHcl1	EtOHcl2	Galcl1	YPDnc1	YPDnc2	YPDnc3	YPDnc4	EtOHnc1	EtOHnc2	Galnc1	Galnc2
13	CLB1	YGR108W	YPDcl2	YPDcl3	EtOHcl1	EtOHcl2	Galcl1	YPDnc1	YPDnc2	YPDnc3	YPDnc4	EtOHnc1	EtOHnc2	Galnc1	Galnc2
13	SVF1	YDR346C	YPDcl2	YPDcl3	EtOHcl1	EtOHcl2	Galcl1	YPDnc1	YPDnc2	YPDnc3	YPDnc4	EtOHnc1	EtOHnc2	Galnc1	Galnc2
13	RLI1	YDR091C	YPDcl2	YPDcl3	EtOHcl1	EtOHcl2	Galcl1	YPDnc1	YPDnc2	YPDnc3	YPDnc4	EtOHnc1	EtOHnc2	Galnc1	Galnc2
13	OST4	YDL232W	YPDcl2	YPDcl3	EtOHcl1	EtOHcl2	Galcl1	YPDnc1	YPDnc2	YPDnc3	YPDnc4	EtOHnc1	EtOHnc2	Galnc1	Galnc2
13		YER165C-A	YPDcl2	YPDcl3	EtOHcl1	EtOHcl2	Galcl1	YPDnc1	YPDnc2	YPDnc3	YPDnc4	EtOHnc1	EtOHnc2	Galnc1	Galnc2
13	CLF1	YLR117C	YPDcl2	YPDcl3	EtOHcl1	EtOHcl2	Galcl1	YPDnc1	YPDnc2	YPDnc3	YPDnc4	EtOHnc1	EtOHnc2	Galnc1	Galnc2
13	HXT13	YEL069C	YPDcl2	YPDcl3	EtOHcl1	EtOHcl2	Galcl1	YPDnc1	YPDnc2	YPDnc3	YPDnc4	EtOHnc1	EtOHnc2	Galnc1	Galnc2
13	SPO23	YBR250W	YPDcl2	YPDcl3	EtOHcl1	EtOHcl2	Galcl1	YPDnc1	YPDnc2	YPDnc3	YPDnc4	EtOHnc1	EtOHnc2	Galnc1	Galnc2
13	SAN1	YDR143C	YPDcl2	YPDcl3	EtOHcl1	EtOHcl2	Galcl1	YPDnc1	YPDnc2	YPDnc3	YPDnc4	EtOHnc1	EtOHnc2	Galnc1	Galnc2
13	PUG1	YER185W	YPDcl2	YPDcl3	EtOHcl1	EtOHcl2	Galcl1	YPDnc1	YPDnc2	YPDnc3	YPDnc4	EtOHnc1	EtOHnc2	Galnc1	Galnc2
13	HRD1	YOL013C	YPDcl2	YPDcl3	EtOHcl1	EtOHcl2	Galcl1	YPDnc1	YPDnc2	YPDnc3	YPDnc4	EtOHnc1	EtOHnc2	Galnc1	Galnc2
13	GIP4	YAL031C	YPDcl2	YPDcl3	EtOHcl1	EtOHcl2	Galcl1	YPDnc1	YPDnc2	YPDnc3	YPDnc4	EtOHnc1	EtOHnc2	Galnc1	Galnc2
13		YGR139W	YPDcl2	YPDcl3	EtOHcl1	EtOHcl2	Galcl1	YPDnc1	YPDnc2	YPDnc3	YPDnc4	EtOHnc1	EtOHnc2	Galnc1	Galnc2
13	ALT1	YLR089C	YPDcl2	YPDcl3	EtOHcl1	EtOHcl2	Galcl1	YPDnc1	YPDnc2	YPDnc3	YPDnc4	EtOHnc1	EtOHnc2	Galnc1	Galnc2
13	RSM22	YKL155C	YPDcl2	YPDcl3	EtOHcl1	EtOHcl2	Galcl1	YPDnc1	YPDnc2	YPDnc3	YPDnc4	EtOHnc1	EtOHnc2	Galnc1	Galnc2
13		YKL222C	YPDcl2	YPDcl3	EtOHcl1	EtOHcl2	Galcl1	YPDnc1	YPDnc2	YPDnc3	YPDnc4	EtOHnc1	EtOHnc2	Galnc1	Galnc2

13	NOP1	YDL014W	YPDcl2	YPDcl3	EtOHcl1	EtOHcl2	Galcl1	YPDnc1	YPDnc2	YPDnc3	YPDnc4	EtOHnc1	EtOHnc2	Galnc1	Galnc2
13	CHA1	YCL064C	YPDcl2	YPDcl3	EtOHcl1	EtOHcl2	Galcl1	YPDnc1	YPDnc2	YPDnc3	YPDnc4	EtOHnc1	EtOHnc2	Galnc1	Galnc2
13	AIM8	YDR493W	YPDcl2	YPDcl3	EtOHcl1	EtOHcl2	Galcl1	YPDnc1	YPDnc2	YPDnc3	YPDnc4	EtOHnc1	EtOHnc2	Galnc1	Galnc2
13	MIC14	YDR031W	YPDcl2	YPDcl3	EtOHcl1	EtOHcl2	Galcl1	YPDnc1	YPDnc2	YPDnc3	YPDnc4	EtOHnc1	EtOHnc2	Galnc1	Galnc2
13	UFD2	YDL190C	YPDcl2	YPDcl3	EtOHcl1	EtOHcl2	Galcl1	YPDnc1	YPDnc2	YPDnc3	YPDnc4	EtOHnc1	EtOHnc2	Galnc1	Galnc2
13	DAD2	YKR083C	YPDcl2	YPDcl3	EtOHcl1	EtOHcl2	Galcl1	YPDnc1	YPDnc2	YPDnc3	YPDnc4	EtOHnc1	EtOHnc2	Galnc1	Galnc2
13		YBR141C	YPDcl2	YPDcl3	EtOHcl1	EtOHcl2	Galcl1	YPDnc1	YPDnc2	YPDnc3	YPDnc4	EtOHnc1	EtOHnc2	Galnc1	Galnc2
13	TSA2	YDR453C	YPDcl2	YPDcl3	EtOHcl1	EtOHcl2	Galcl1	YPDnc1	YPDnc2	YPDnc3	YPDnc4	EtOHnc1	EtOHnc2	Galnc1	Galnc2
13		YER107W-	YPDcl2	YPDcl3	EtOHcl1	EtOHcl2	Galcl1	YPDnc1	YPDnc2	YPDnc3	YPDnc4	EtOHnc1	EtOHnc2	Galnc1	Galnc2
13		YNL193W	YPDcl2	YPDcl3	EtOHcl1	EtOHcl2	Galcl1	YPDnc1	YPDnc2	YPDnc3	YPDnc4	EtOHnc1	EtOHnc2	Galnc1	Galnc2
13	TRA1	YHR099W	YPDcl2	YPDcl3	EtOHcl1	EtOHcl2	Galcl1	YPDnc1	YPDnc2	YPDnc3	YPDnc4	EtOHnc1	EtOHnc2	Galnc1	Galnc2
13		YPR170W-	YPDcl2	YPDcl3	EtOHcl1	EtOHcl2	Galcl1	YPDnc1	YPDnc2	YPDnc3	YPDnc4	EtOHnc1	EtOHnc2	Galnc1	Galnc2
13		YKL070W	YPDcl2	YPDcl3	EtOHcl1	EtOHcl2	Galcl1	YPDnc1	YPDnc2	YPDnc3	YPDnc4	EtOHnc1	EtOHnc2	Galnc1	Galnc2
13		YKL145W-	YPDcl2	YPDcl3	EtOHcl1	EtOHcl2	Galcl1	YPDnc1	YPDnc2	YPDnc3	YPDnc4	EtOHnc1	EtOHnc2	Galnc1	Galnc2
13	SEN1	YLR430W	YPDcl2	YPDcl3	EtOHcl1	EtOHcl2	Galcl1	YPDnc1	YPDnc2	YPDnc3	YPDnc4	EtOHnc1	EtOHnc2	Galnc1	Galnc2
13	NPR3	YHL023C	YPDcl2	YPDcl3	EtOHcl1	EtOHcl2	Galcl1	YPDnc1	YPDnc2	YPDnc3	YPDnc4	EtOHnc1	EtOHnc2	Galnc1	Galnc2
13		YOR283W	YPDcl2	YPDcl3	EtOHcl1	EtOHcl2	Galcl1	YPDnc1	YPDnc2	YPDnc3	YPDnc4	EtOHnc1	EtOHnc2	Galnc1	Galnc2
13		YMR052C-	YPDcl2	YPDcl3	EtOHcl1	EtOHcl2	Galcl1	YPDnc1	YPDnc2	YPDnc3	YPDnc4	EtOHnc1	EtOHnc2	Galnc1	Galnc2
13		YHL005C	YPDcl2	YPDcl3	EtOHcl1	EtOHcl2	Galcl1	YPDnc1	YPDnc2	YPDnc3	YPDnc4	EtOHnc1	EtOHnc2	Galnc1	Galnc2
13		YDR029W	YPDcl2	YPDcl3	EtOHcl1	EtOHcl2	Galcl1	YPDnc1	YPDnc2	YPDnc3	YPDnc4	EtOHnc1	EtOHnc2	Galnc1	Galnc2
13	MRH1	YDR033W	YPDcl2	YPDcl3	EtOHcl1	EtOHcl2	Galcl1	YPDnc1	YPDnc2	YPDnc3	YPDnc4	EtOHnc1	EtOHnc2	Galnc1	Galnc2
13		YNL043C	YPDcl2	YPDcl3	EtOHcl1	EtOHcl2	Galcl1	YPDnc1	YPDnc2	YPDnc3	YPDnc4	EtOHnc1	EtOHnc2	Galnc1	Galnc2
13	MET32	YDR253C	YPDcl2	YPDcl3	EtOHcl1	EtOHcl2	Galcl1	YPDnc1	YPDnc2	YPDnc3	YPDnc4	EtOHnc1	EtOHnc2	Galnc1	Galnc2
13	ZWF1	YNL241C	YPDcl2	YPDcl3	EtOHcl1	EtOHcl2	Galcl1	YPDnc1	YPDnc2	YPDnc3	YPDnc4	EtOHnc1	EtOHnc2	Galnc1	Galnc2
13	PRP45	YAL032C	YPDcl2	YPDcl3	EtOHcl1	EtOHcl2	Galcl1	YPDnc1	YPDnc2	YPDnc3	YPDnc4	EtOHnc1	EtOHnc2	Galnc1	Galnc2
13	MID1	YNL291C	YPDcl2	YPDcl3	EtOHcl1	EtOHcl2	Galcl1	YPDnc1	YPDnc2	YPDnc3	YPDnc4	EtOHnc1	EtOHnc2	Galnc1	Galnc2
13	PUT4	YOR348C	YPDcl2	YPDcl3	EtOHcl1	EtOHcl2	Galcl1	YPDnc1	YPDnc2	YPDnc3	YPDnc4	EtOHnc1	EtOHnc2	Galnc1	Galnc2
13		YOR097C	YPDcl2	YPDcl3	EtOHcl1	EtOHcl2	Galcl1	YPDnc1	YPDnc2	YPDnc3	YPDnc4	EtOHnc1	EtOHnc2	Galnc1	Galnc2
13	MDH2	YOL126C	YPDcl2	YPDcl3	EtOHcl1	EtOHcl2	Galcl1	YPDnc1	YPDnc2	YPDnc3	YPDnc4	EtOHnc1	EtOHnc2	Galnc1	Galnc2
13	DUO1	YGL061C	YPDcl2	YPDcl3	EtOHcl1	EtOHcl2	Galcl1	YPDnc1	YPDnc2	YPDnc3	YPDnc4	EtOHnc1	EtOHnc2	Galnc1	Galnc2
13	SIP4	YJL089W	YPDcl2	YPDcl3	EtOHcl1	EtOHcl2	Galcl1	YPDnc1	YPDnc2	YPDnc3	YPDnc4	EtOHnc1	EtOHnc2	Galnc1	Galnc2
13	WSS1	YHR134W	YPDcl2	YPDcl3	EtOHcl1	EtOHcl2	Galcl1	YPDnc1	YPDnc2	YPDnc3	YPDnc4	EtOHnc1	EtOHnc2	Galnc1	Galnc2
13		YOR022C	YPDcl2	YPDcl3	EtOHcl1	EtOHcl2	Galcl1	YPDnc1	YPDnc2	YPDnc3	YPDnc4	EtOHnc1	EtOHnc2	Galnc1	Galnc2
13	SLX1	YBR228W	YPDcl2	YPDcl3	EtOHcl1	EtOHcl2	Galcl1	YPDnc1	YPDnc2	YPDnc3	YPDnc4	EtOHnc1	EtOHnc2	Galnc1	Galnc2

13	PPT1	YGR123C	YPDcl2	YPDcl3	EtOHcl1	EtOHcl2	Galcl1	YPDnc1	YPDnc2	YPDnc3	YPDnc4	EtOHnc1	EtOHnc2	Galnc1	Galnc2
13	ERG8	YMR220W	YPDcl2	YPDcl3	EtOHcl1	EtOHcl2	Galcl1	YPDnc1	YPDnc2	YPDnc3	YPDnc4	EtOHnc1	EtOHnc2	Galnc1	Galnc2
13	FMP21	YBR269C	YPDcl2	YPDcl3	EtOHcl1	EtOHcl2	Galcl1	YPDnc1	YPDnc2	YPDnc3	YPDnc4	EtOHnc1	EtOHnc2	Galnc1	Galnc2
13	MRPS17	YMR188C	YPDcl2	YPDcl3	EtOHcl1	EtOHcl2	Galcl1	YPDnc1	YPDnc2	YPDnc3	YPDnc4	EtOHnc1	EtOHnc2	Galnc1	Galnc2
13		YLR290C	YPDcl2	YPDcl3	EtOHcl1	EtOHcl2	Galcl1	YPDnc1	YPDnc2	YPDnc3	YPDnc4	EtOHnc1	EtOHnc2	Galnc1	Galnc2
13	IMP4	YNL075W	YPDcl2	YPDcl3	EtOHcl1	EtOHcl2	Galcl1	YPDnc1	YPDnc2	YPDnc3	YPDnc4	EtOHnc1	EtOHnc2	Galnc1	Galnc2
13	DSL1	YNL258C	YPDcl2	YPDcl3	EtOHcl1	EtOHcl2	Galcl1	YPDnc1	YPDnc2	YPDnc3	YPDnc4	EtOHnc1	EtOHnc2	Galnc1	Galnc2
13	CIK1	YMR198W	YPDcl2	YPDcl3	EtOHcl1	EtOHcl2	Galcl1	YPDnc1	YPDnc2	YPDnc3	YPDnc4	EtOHnc1	EtOHnc2	Galnc1	Galnc2
13	GAR1	YHR089C	YPDcl2	YPDcl3	EtOHcl1	EtOHcl2	Galcl1	YPDnc1	YPDnc2	YPDnc3	YPDnc4	EtOHnc1	EtOHnc2	Galnc1	Galnc2
13	AIM4	YBR194W	YPDcl2	YPDcl3	EtOHcl1	EtOHcl2	Galcl1	YPDnc1	YPDnc2	YPDnc3	YPDnc4	EtOHnc1	EtOHnc2	Galnc1	Galnc2
13	AIM38	YNR018W	YPDcl2	YPDcl3	EtOHcl1	EtOHcl2	Galcl1	YPDnc1	YPDnc2	YPDnc3	YPDnc4	EtOHnc1	EtOHnc2	Galnc1	Galnc2
13	GCV3	YAL044C	YPDcl2	YPDcl3	EtOHcl1	EtOHcl2	Galcl1	YPDnc1	YPDnc2	YPDnc3	YPDnc4	EtOHnc1	EtOHnc2	Galnc1	Galnc2
13	PEX1	YKL197C	YPDcl2	YPDcl3	EtOHcl1	EtOHcl2	Galcl1	YPDnc1	YPDnc2	YPDnc3	YPDnc4	EtOHnc1	EtOHnc2	Galnc1	Galnc2
13	NAT5	YOR253W	YPDcl2	YPDcl3	EtOHcl1	EtOHcl2	Galcl1	YPDnc1	YPDnc2	YPDnc3	YPDnc4	EtOHnc1	EtOHnc2	Galnc1	Galnc2
13	SLM1	YIL105C	YPDcl2	YPDcl3	EtOHcl1	EtOHcl2	Galcl1	YPDnc1	YPDnc2	YPDnc3	YPDnc4	EtOHnc1	EtOHnc2	Galnc1	Galnc2
13	DIT1	YDR403W	YPDcl2	YPDcl3	EtOHcl1	EtOHcl2	Galcl1	YPDnc1	YPDnc2	YPDnc3	YPDnc4	EtOHnc1	EtOHnc2	Galnc1	Galnc2
13	ARP2	YDL029W	YPDcl2	YPDcl3	EtOHcl1	EtOHcl2	Galcl1	YPDnc1	YPDnc2	YPDnc3	YPDnc4	EtOHnc1	EtOHnc2	Galnc1	Galnc2
13	HBN1	YCL026C-B	YPDcl2	YPDcl3	EtOHcl1	EtOHcl2	Galcl1	YPDnc1	YPDnc2	YPDnc3	YPDnc4	EtOHnc1	EtOHnc2	Galnc1	Galnc2
13		YPR092W	YPDcl2	YPDcl3	EtOHcl1	EtOHcl2	Galcl1	YPDnc1	YPDnc2	YPDnc3	YPDnc4	EtOHnc1	EtOHnc2	Galnc1	Galnc2
13	BBP1	YPL255W	YPDcl2	YPDcl3	EtOHcl1	EtOHcl2	Galcl1	YPDnc1	YPDnc2	YPDnc3	YPDnc4	EtOHnc1	EtOHnc2	Galnc1	Galnc2
13	SAP4	YGL229C	YPDcl2	YPDcl3	EtOHcl1	EtOHcl2	Galcl1	YPDnc1	YPDnc2	YPDnc3	YPDnc4	EtOHnc1	EtOHnc2	Galnc1	Galnc2
13	ICL1	YER065C	YPDcl2	YPDcl3	EtOHcl1	EtOHcl2	Galcl1	YPDnc1	YPDnc2	YPDnc3	YPDnc4	EtOHnc1	EtOHnc2	Galnc1	Galnc2
13	RPB11	YOL005C	YPDcl2	YPDcl3	EtOHcl1	EtOHcl2	Galcl1	YPDnc1	YPDnc2	YPDnc3	YPDnc4	EtOHnc1	EtOHnc2	Galnc1	Galnc2
13	SUR7	YML052W	YPDcl2	YPDcl3	EtOHcl1	EtOHcl2	Galcl1	YPDnc1	YPDnc2	YPDnc3	YPDnc4	EtOHnc1	EtOHnc2	Galnc1	Galnc2
13	APM2	YHL019C	YPDcl2	YPDcl3	EtOHcl1	EtOHcl2	Galcl1	YPDnc1	YPDnc2	YPDnc3	YPDnc4	EtOHnc1	EtOHnc2	Galnc1	Galnc2
13	POP7	YBR167C	YPDcl2	YPDcl3	EtOHcl1	EtOHcl2	Galcl1	YPDnc1	YPDnc2	YPDnc3	YPDnc4	EtOHnc1	EtOHnc2	Galnc1	Galnc2
13	SMC6	YLR383W	YPDcl2	YPDcl3	EtOHcl1	EtOHcl2	Galcl1	YPDnc1	YPDnc2	YPDnc3	YPDnc4	EtOHnc1	EtOHnc2	Galnc1	Galnc2
13	MTQ2	YDR140W	YPDcl2	YPDcl3	EtOHcl1	EtOHcl2	Galcl1	YPDnc1	YPDnc2	YPDnc3	YPDnc4	EtOHnc1	EtOHnc2	Galnc1	Galnc2
13	MRPL17	YNL252C	YPDcl2	YPDcl3	EtOHcl1	EtOHcl2	Galcl1	YPDnc1	YPDnc2	YPDnc3	YPDnc4	EtOHnc1	EtOHnc2	Galnc1	Galnc2
13	BGL2	YGR282C	YPDcl2	YPDcl3	EtOHcl1	EtOHcl2	Galcl1	YPDnc1	YPDnc2	YPDnc3	YPDnc4	EtOHnc1	EtOHnc2	Galnc1	Galnc2
13		YMR196W	YPDcl2	YPDcl3	EtOHcl1	EtOHcl2	Galcl1	YPDnc1	YPDnc2	YPDnc3	YPDnc4	EtOHnc1	EtOHnc2	Galnc1	Galnc2
13	GEP3	YOR205C	YPDcl2	YPDcl3	EtOHcl1	EtOHcl2	Galcl1	YPDnc1	YPDnc2	YPDnc3	YPDnc4	EtOHnc1	EtOHnc2	Galnc1	Galnc2
13	STB5	YHR178W	YPDcl2	YPDcl3	EtOHcl1	EtOHcl2	Galcl1	YPDnc1	YPDnc2	YPDnc3	YPDnc4	EtOHnc1	EtOHnc2	Galnc1	Galnc2
13	SLK19	YOR195W	YPDcl2	YPDcl3	EtOHcl1	EtOHcl2	Galcl1	YPDnc1	YPDnc2	YPDnc3	YPDnc4	EtOHnc1	EtOHnc2	Galnc1	Galnc2

13	VEL1	YGL258W	YPDcl2	YPDcl3	EtOHcl1	EtOHcl2	Galcl1	YPDnc1	YPDnc2	YPDnc3	YPDnc4	EtOHnc1	EtOHnc2	Galnc1	Galnc2
13	INP51	YIL002C	YPDcl2	YPDcl3	EtOHcl1	EtOHcl2	Galcl1	YPDnc1	YPDnc2	YPDnc3	YPDnc4	EtOHnc1	EtOHnc2	Galnc1	Galnc2
13	ALY1	YKR021W	YPDcl2	YPDcl3	EtOHcl1	EtOHcl2	Galcl1	YPDnc1	YPDnc2	YPDnc3	YPDnc4	EtOHnc1	EtOHnc2	Galnc1	Galnc2
13	NMA1	YLR328W	YPDcl2	YPDcl3	EtOHcl1	EtOHcl2	Galcl1	YPDnc1	YPDnc2	YPDnc3	YPDnc4	EtOHnc1	EtOHnc2	Galnc1	Galnc2
13	SNU114	YKL173W	YPDcl2	YPDcl3	EtOHcl1	EtOHcl2	Galcl1	YPDnc1	YPDnc2	YPDnc3	YPDnc4	EtOHnc1	EtOHnc2	Galnc1	Galnc2
13	PHO91	YNR013C	YPDcl2	YPDcl3	EtOHcl1	EtOHcl2	Galcl1	YPDnc1	YPDnc2	YPDnc3	YPDnc4	EtOHnc1	EtOHnc2	Galnc1	Galnc2
13	MIR1	YJR077C	YPDcl2	YPDcl3	EtOHcl1	EtOHcl2	Galcl1	YPDnc1	YPDnc2	YPDnc3	YPDnc4	EtOHnc1	EtOHnc2	Galnc1	Galnc2
13		YPR078C	YPDcl2	YPDcl3	EtOHcl1	EtOHcl2	Galcl1	YPDnc1	YPDnc2	YPDnc3	YPDnc4	EtOHnc1	EtOHnc2	Galnc1	Galnc2
13	YKU70	YMR284W	YPDcl2	YPDcl3	EtOHcl1	EtOHcl2	Galcl1	YPDnc1	YPDnc2	YPDnc3	YPDnc4	EtOHnc1	EtOHnc2	Galnc1	Galnc2
13	PIF1	YML061C	YPDcl2	YPDcl3	EtOHcl1	EtOHcl2	Galcl1	YPDnc1	YPDnc2	YPDnc3	YPDnc4	EtOHnc1	EtOHnc2	Galnc1	Galnc2
13	FPR4	YLR449W	YPDcl2	YPDcl3	EtOHcl1	EtOHcl2	Galcl1	YPDnc1	YPDnc2	YPDnc3	YPDnc4	EtOHnc1	EtOHnc2	Galnc1	Galnc2
13	RPT2	YDL007W	YPDcl2	YPDcl3	EtOHcl1	EtOHcl2	Galcl1	YPDnc1	YPDnc2	YPDnc3	YPDnc4	EtOHnc1	EtOHnc2	Galnc1	Galnc2
13	PNO1	YOR145C	YPDcl2	YPDcl3	EtOHcl1	EtOHcl2	Galcl1	YPDnc1	YPDnc2	YPDnc3	YPDnc4	EtOHnc1	EtOHnc2	Galnc1	Galnc2
13		YMR317W	YPDcl2	YPDcl3	EtOHcl1	EtOHcl2	Galcl1	YPDnc1	YPDnc2	YPDnc3	YPDnc4	EtOHnc1	EtOHnc2	Galnc1	Galnc2
13	MET1	YKR069W	YPDcl2	YPDcl3	EtOHcl1	EtOHcl2	Galcl1	YPDnc1	YPDnc2	YPDnc3	YPDnc4	EtOHnc1	EtOHnc2	Galnc1	Galnc2
13		YOR287C	YPDcl2	YPDcl3	EtOHcl1	EtOHcl2	Galcl1	YPDnc1	YPDnc2	YPDnc3	YPDnc4	EtOHnc1	EtOHnc2	Galnc1	Galnc2
13		YHR071C-A	YPDcl2	YPDcl3	EtOHcl1	EtOHcl2	Galcl1	YPDnc1	YPDnc2	YPDnc3	YPDnc4	EtOHnc1	EtOHnc2	Galnc1	Galnc2
13	SAR1	YPL218W	YPDcl2	YPDcl3	EtOHcl1	EtOHcl2	Galcl1	YPDnc1	YPDnc2	YPDnc3	YPDnc4	EtOHnc1	EtOHnc2	Galnc1	Galnc2
13		YJR149W	YPDcl2	YPDcl3	EtOHcl1	EtOHcl2	Galcl1	YPDnc1	YPDnc2	YPDnc3	YPDnc4	EtOHnc1	EtOHnc2	Galnc1	Galnc2
13	SFH5	YJL145W	YPDcl2	YPDcl3	EtOHcl1	EtOHcl2	Galcl1	YPDnc1	YPDnc2	YPDnc3	YPDnc4	EtOHnc1	EtOHnc2	Galnc1	Galnc2
13	SFB3	YHR098C	YPDcl2	YPDcl3	EtOHcl1	EtOHcl2	Galcl1	YPDnc1	YPDnc2	YPDnc3	YPDnc4	EtOHnc1	EtOHnc2	Galnc1	Galnc2
13	ESA1	YOR244W	YPDcl2	YPDcl3	EtOHcl1	EtOHcl2	Galcl1	YPDnc1	YPDnc2	YPDnc3	YPDnc4	EtOHnc1	EtOHnc2	Galnc1	Galnc2
13		YLR173W	YPDcl2	YPDcl3	EtOHcl1	EtOHcl2	Galcl1	YPDnc1	YPDnc2	YPDnc3	YPDnc4	EtOHnc1	EtOHnc2	Galnc1	Galnc2
13	ERG9	YHR190W	YPDcl2	YPDcl3	EtOHcl1	EtOHcl2	Galcl1	YPDnc1	YPDnc2	YPDnc3	YPDnc4	EtOHnc1	EtOHnc2	Galnc1	Galnc2
13	MSY1	YPL097W	YPDcl2	YPDcl3	EtOHcl1	EtOHcl2	Galcl1	YPDnc1	YPDnc2	YPDnc3	YPDnc4	EtOHnc1	EtOHnc2	Galnc1	Galnc2
13		YNL115C	YPDcl2	YPDcl3	EtOHcl1	EtOHcl2	Galcl1	YPDnc1	YPDnc2	YPDnc3	YPDnc4	EtOHnc1	EtOHnc2	Galnc1	Galnc2
13	CDC45	YLR103C	YPDcl2	YPDcl3	EtOHcl1	EtOHcl2	Galcl1	YPDnc1	YPDnc2	YPDnc3	YPDnc4	EtOHnc1	EtOHnc2	Galnc1	Galnc2
13	CYC8	YBR112C	YPDcl2	YPDcl3	EtOHcl1	EtOHcl2	Galcl1	YPDnc1	YPDnc2	YPDnc3	YPDnc4	EtOHnc1	EtOHnc2	Galnc1	Galnc2
13		YLR123C	YPDcl2	YPDcl3	EtOHcl1	EtOHcl2	Galcl1	YPDnc1	YPDnc2	YPDnc3	YPDnc4	EtOHnc1	EtOHnc2	Galnc1	Galnc2
13	BUD25	YER014C-A	YPDcl2	YPDcl3	EtOHcl1	EtOHcl2	Galcl1	YPDnc1	YPDnc2	YPDnc3	YPDnc4	EtOHnc1	EtOHnc2	Galnc1	Galnc2
13	COX8	YLR395C	YPDcl2	YPDcl3	EtOHcl1	EtOHcl2	Galcl1	YPDnc1	YPDnc2	YPDnc3	YPDnc4	EtOHnc1	EtOHnc2	Galnc1	Galnc2
13	RPL42B	YHR141C	YPDcl2	YPDcl3	EtOHcl1	EtOHcl2	Galcl1	YPDnc1	YPDnc2	YPDnc3	YPDnc4	EtOHnc1	EtOHnc2	Galnc1	Galnc2
13	SAS3	YBL052C	YPDcl2	YPDcl3	EtOHcl1	EtOHcl2	Galcl1	YPDnc1	YPDnc2	YPDnc3	YPDnc4	EtOHnc1	EtOHnc2	Galnc1	Galnc2
13	EMC1	YCL045C	YPDcl2	YPDcl3	EtOHcl1	EtOHcl2	Galcl1	YPDnc1	YPDnc2	YPDnc3	YPDnc4	EtOHnc1	EtOHnc2	Galnc1	Galnc2

13		YNL011C	YPDcl2	YPDcl3	EtOHcl1	EtOHcl2	Galcl1	YPDnc1	YPDnc2	YPDnc3	YPDnc4	EtOHnc1	EtOHnc2	Galnc1	Galnc2
13	BCH1	YMR237W	YPDcl2	YPDcl3	EtOHcl1	EtOHcl2	Galcl1	YPDnc1	YPDnc2	YPDnc3	YPDnc4	EtOHnc1	EtOHnc2	Galnc1	Galnc2
13	RPS0A	YGR214W	YPDcl2	YPDcl3	EtOHcl1	EtOHcl2	Galcl1	YPDnc1	YPDnc2	YPDnc3	YPDnc4	EtOHnc1	EtOHnc2	Galnc1	Galnc2
13		YIR021W-A	YPDcl2	YPDcl3	EtOHcl1	EtOHcl2	Galcl1	YPDnc1	YPDnc2	YPDnc3	YPDnc4	EtOHnc1	EtOHnc2	Galnc1	Galnc2
13	TAM41	YGR046W	YPDcl2	YPDcl3	EtOHcl1	EtOHcl2	Galcl1	YPDnc1	YPDnc2	YPDnc3	YPDnc4	EtOHnc1	EtOHnc2	Galnc1	Galnc2
13	AZR1	YGR224W	YPDcl2	YPDcl3	EtOHcl1	EtOHcl2	Galcl1	YPDnc1	YPDnc2	YPDnc3	YPDnc4	EtOHnc1	EtOHnc2	Galnc1	Galnc2
13		YMR130W	YPDcl2	YPDcl3	EtOHcl1	EtOHcl2	Galcl1	YPDnc1	YPDnc2	YPDnc3	YPDnc4	EtOHnc1	EtOHnc2	Galnc1	Galnc2
13	CAF4	YKR036C	YPDcl2	YPDcl3	EtOHcl1	EtOHcl2	Galcl1	YPDnc1	YPDnc2	YPDnc3	YPDnc4	EtOHnc1	EtOHnc2	Galnc1	Galnc2
13		YIR030W-A	YPDcl2	YPDcl3	EtOHcl1	EtOHcl2	Galcl1	YPDnc1	YPDnc2	YPDnc3	YPDnc4	EtOHnc1	EtOHnc2	Galnc1	Galnc2
13		YIL047C-A	YPDcl2	YPDcl3	EtOHcl1	EtOHcl2	Galcl1	YPDnc1	YPDnc2	YPDnc3	YPDnc4	EtOHnc1	EtOHnc2	Galnc1	Galnc2
13	PDX3	YBR035C	YPDcl2	YPDcl3	EtOHcl1	EtOHcl2	Galcl1	YPDnc1	YPDnc2	YPDnc3	YPDnc4	EtOHnc1	EtOHnc2	Galnc1	Galnc2
13	PEX5	YDR244W	YPDcl2	YPDcl3	EtOHcl1	EtOHcl2	Galcl1	YPDnc1	YPDnc2	YPDnc3	YPDnc4	EtOHnc1	EtOHnc2	Galnc1	Galnc2
13	ASR1	YPR093C	YPDcl2	YPDcl3	EtOHcl1	EtOHcl2	Galcl1	YPDnc1	YPDnc2	YPDnc3	YPDnc4	EtOHnc1	EtOHnc2	Galnc1	Galnc2
13	RNT1	YMR239C	YPDcl2	YPDcl3	EtOHcl1	EtOHcl2	Galcl1	YPDnc1	YPDnc2	YPDnc3	YPDnc4	EtOHnc1	EtOHnc2	Galnc1	Galnc2
13	MAS1	YLR163C	YPDcl2	YPDcl3	EtOHcl1	EtOHcl2	Galcl1	YPDnc1	YPDnc2	YPDnc3	YPDnc4	EtOHnc1	EtOHnc2	Galnc1	Galnc2
13	NBL1	YHR199C-A	YPDcl2	YPDcl3	EtOHcl1	EtOHcl2	Galcl1	YPDnc1	YPDnc2	YPDnc3	YPDnc4	EtOHnc1	EtOHnc2	Galnc1	Galnc2
13	PCL6	YER059W	YPDcl2	YPDcl3	EtOHcl1	EtOHcl2	Galcl1	YPDnc1	YPDnc2	YPDnc3	YPDnc4	EtOHnc1	EtOHnc2	Galnc1	Galnc2
13	FTH1	YBR207W	YPDcl2	YPDcl3	EtOHcl1	EtOHcl2	Galcl1	YPDnc1	YPDnc2	YPDnc3	YPDnc4	EtOHnc1	EtOHnc2	Galnc1	Galnc2
13	DFG10	YIL049W	YPDcl2	YPDcl3	EtOHcl1	EtOHcl2	Galcl1	YPDnc1	YPDnc2	YPDnc3	YPDnc4	EtOHnc1	EtOHnc2	Galnc1	Galnc2
13	RPL6A	YML073C	YPDcl2	YPDcl3	EtOHcl1	EtOHcl2	Galcl1	YPDnc1	YPDnc2	YPDnc3	YPDnc4	EtOHnc1	EtOHnc2	Galnc1	Galnc2
13	RAX2	YLR084C	YPDcl2	YPDcl3	EtOHcl1	EtOHcl2	Galcl1	YPDnc1	YPDnc2	YPDnc3	YPDnc4	EtOHnc1	EtOHnc2	Galnc1	Galnc2
13	UBA3	YPR066W	YPDcl2	YPDcl3	EtOHcl1	EtOHcl2	Galcl1	YPDnc1	YPDnc2	YPDnc3	YPDnc4	EtOHnc1	EtOHnc2	Galnc1	Galnc2
13		YOR161C-C	YPDcl2	YPDcl3	EtOHcl1	EtOHcl2	Galcl1	YPDnc1	YPDnc2	YPDnc3	YPDnc4	EtOHnc1	EtOHnc2	Galnc1	Galnc2
13	UBC13	YDR092W	YPDcl2	YPDcl3	EtOHcl1	EtOHcl2	Galcl1	YPDnc1	YPDnc2	YPDnc3	YPDnc4	EtOHnc1	EtOHnc2	Galnc1	Galnc2
13	DIA2	YOR080W	YPDcl2	YPDcl3	EtOHcl1	EtOHcl2	Galcl1	YPDnc1	YPDnc2	YPDnc3	YPDnc4	EtOHnc1	EtOHnc2	Galnc1	Galnc2
13	PEP8	YJL053W	YPDcl2	YPDcl3	EtOHcl1	EtOHcl2	Galcl1	YPDnc1	YPDnc2	YPDnc3	YPDnc4	EtOHnc1	EtOHnc2	Galnc1	Galnc2
13	FYV10	YIL097W	YPDcl2	YPDcl3	EtOHcl1	EtOHcl2	Galcl1	YPDnc1	YPDnc2	YPDnc3	YPDnc4	EtOHnc1	EtOHnc2	Galnc1	Galnc2
13		YNR025C	YPDcl2	YPDcl3	EtOHcl1	EtOHcl2	Galcl1	YPDnc1	YPDnc2	YPDnc3	YPDnc4	EtOHnc1	EtOHnc2	Galnc1	Galnc2
13	PPR1	YLR014C	YPDcl2	YPDcl3	EtOHcl1	EtOHcl2	Galcl1	YPDnc1	YPDnc2	YPDnc3	YPDnc4	EtOHnc1	EtOHnc2	Galnc1	Galnc2
13	AVT4	YNL101W	YPDcl2	YPDcl3	EtOHcl1	EtOHcl2	Galcl1	YPDnc1	YPDnc2	YPDnc3	YPDnc4	EtOHnc1	EtOHnc2	Galnc1	Galnc2
13	KAP114	YGL241W	YPDcl2	YPDcl3	EtOHcl1	EtOHcl2	Galcl1	YPDnc1	YPDnc2	YPDnc3	YPDnc4	EtOHnc1	EtOHnc2	Galnc1	Galnc2
13	RPC10	YHR143W-A	YPDcl2	YPDcl3	EtOHcl1	EtOHcl2	Galcl1	YPDnc1	YPDnc2	YPDnc3	YPDnc4	EtOHnc1	EtOHnc2	Galnc1	Galnc2
13	COF1	YLL050C	YPDcl2	YPDcl3	EtOHcl1	EtOHcl2	Galcl1	YPDnc1	YPDnc2	YPDnc3	YPDnc4	EtOHnc1	EtOHnc2	Galnc1	Galnc2
13	PEX4	YGR133W	YPDcl2	YPDcl3	EtOHcl1	EtOHcl2	Galcl1	YPDnc1	YPDnc2	YPDnc3	YPDnc4	EtOHnc1	EtOHnc2	Galnc1	Galnc2

13	SOK1	YDR006C	YPDcl2	YPDcl3	EtOHcl1	EtOHcl2	Galcl1	YPDnc1	YPDnc2	YPDnc3	YPDnc4	EtOHnc1	EtOHnc2	Galnc1	Galnc2
13	FOL1	YNL256W	YPDcl2	YPDcl3	EtOHcl1	EtOHcl2	Galcl1	YPDnc1	YPDnc2	YPDnc3	YPDnc4	EtOHnc1	EtOHnc2	Galnc1	Galnc2
13	UBP9	YER098W	YPDcl2	YPDcl3	EtOHcl1	EtOHcl2	Galcl1	YPDnc1	YPDnc2	YPDnc3	YPDnc4	EtOHnc1	EtOHnc2	Galnc1	Galnc2
13		YDR401W	YPDcl2	YPDcl3	EtOHcl1	EtOHcl2	Galcl1	YPDnc1	YPDnc2	YPDnc3	YPDnc4	EtOHnc1	EtOHnc2	Galnc1	Galnc2
13	FIG4	YNL325C	YPDcl2	YPDcl3	EtOHcl1	EtOHcl2	Galcl1	YPDnc1	YPDnc2	YPDnc3	YPDnc4	EtOHnc1	EtOHnc2	Galnc1	Galnc2
13		YGL204C	YPDcl2	YPDcl3	EtOHcl1	EtOHcl2	Galcl1	YPDnc1	YPDnc2	YPDnc3	YPDnc4	EtOHnc1	EtOHnc2	Galnc1	Galnc2
13	ATG14	YBR128C	YPDcl2	YPDcl3	EtOHcl1	EtOHcl2	Galcl1	YPDnc1	YPDnc2	YPDnc3	YPDnc4	EtOHnc1	EtOHnc2	Galnc1	Galnc2
13	HUR1	YGL168W	YPDcl2	YPDcl3	EtOHcl1	EtOHcl2	Galcl1	YPDnc1	YPDnc2	YPDnc3	YPDnc4	EtOHnc1	EtOHnc2	Galnc1	Galnc2
13	IRC4	YDR540C	YPDcl2	YPDcl3	EtOHcl1	EtOHcl2	Galcl1	YPDnc1	YPDnc2	YPDnc3	YPDnc4	EtOHnc1	EtOHnc2	Galnc1	Galnc2
13	RAP1	YNL216W	YPDcl2	YPDcl3	EtOHcl1	EtOHcl2	Galcl1	YPDnc1	YPDnc2	YPDnc3	YPDnc4	EtOHnc1	EtOHnc2	Galnc1	Galnc2
13		YJL215C	YPDcl2	YPDcl3	EtOHcl1	EtOHcl2	Galcl1	YPDnc1	YPDnc2	YPDnc3	YPDnc4	EtOHnc1	EtOHnc2	Galnc1	Galnc2
13		YOR282W	YPDcl2	YPDcl3	EtOHcl1	EtOHcl2	Galcl1	YPDnc1	YPDnc2	YPDnc3	YPDnc4	EtOHnc1	EtOHnc2	Galnc1	Galnc2
13	ERI1	YPL096C-A	YPDcl2	YPDcl3	EtOHcl1	EtOHcl2	Galcl1	YPDnc1	YPDnc2	YPDnc3	YPDnc4	EtOHnc1	EtOHnc2	Galnc1	Galnc2
13		YML119W	YPDcl2	YPDcl3	EtOHcl1	EtOHcl2	Galcl1	YPDnc1	YPDnc2	YPDnc3	YPDnc4	EtOHnc1	EtOHnc2	Galnc1	Galnc2
13	SAM4	YPL273W	YPDcl2	YPDcl3	EtOHcl1	EtOHcl2	Galcl1	YPDnc1	YPDnc2	YPDnc3	YPDnc4	EtOHnc1	EtOHnc2	Galnc1	Galnc2
13		YLR046C	YPDcl2	YPDcl3	EtOHcl1	EtOHcl2	Galcl1	YPDnc1	YPDnc2	YPDnc3	YPDnc4	EtOHnc1	EtOHnc2	Galnc1	Galnc2
13		YFL019C	YPDcl2	YPDcl3	EtOHcl1	EtOHcl2	Galcl1	YPDnc1	YPDnc2	YPDnc3	YPDnc4	EtOHnc1	EtOHnc2	Galnc1	Galnc2
13	MRPL20	YKR085C	YPDcl2	YPDcl3	EtOHcl1	EtOHcl2	Galcl1	YPDnc1	YPDnc2	YPDnc3	YPDnc4	EtOHnc1	EtOHnc2	Galnc1	Galnc2
13	TIF5	YPR041W	YPDcl2	YPDcl3	EtOHcl1	EtOHcl2	Galcl1	YPDnc1	YPDnc2	YPDnc3	YPDnc4	EtOHnc1	EtOHnc2	Galnc1	Galnc2
13	ATG7	YHR171W	YPDcl2	YPDcl3	EtOHcl1	EtOHcl2	Galcl1	YPDnc1	YPDnc2	YPDnc3	YPDnc4	EtOHnc1	EtOHnc2	Galnc1	Galnc2
13	FCF2	YLR051C	YPDcl2	YPDcl3	EtOHcl1	EtOHcl2	Galcl1	YPDnc1	YPDnc2	YPDnc3	YPDnc4	EtOHnc1	EtOHnc2	Galnc1	Galnc2
13	IDH2	YOR136W	YPDcl2	YPDcl3	EtOHcl1	EtOHcl2	Galcl1	YPDnc1	YPDnc2	YPDnc3	YPDnc4	EtOHnc1	EtOHnc2	Galnc1	Galnc2
13		YPR016W-/	YPDcl2	YPDcl3	EtOHcl1	EtOHcl2	Galcl1	YPDnc1	YPDnc2	YPDnc3	YPDnc4	EtOHnc1	EtOHnc2	Galnc1	Galnc2
13	GRX8	YLR364W	YPDcl2	YPDcl3	EtOHcl1	EtOHcl2	Galcl1	YPDnc1	YPDnc2	YPDnc3	YPDnc4	EtOHnc1	EtOHnc2	Galnc1	Galnc2
13	VAC7	YNL054W	YPDcl2	YPDcl3	EtOHcl1	EtOHcl2	Galcl1	YPDnc1	YPDnc2	YPDnc3	YPDnc4	EtOHnc1	EtOHnc2	Galnc1	Galnc2
13	IFM1	YOL023W	YPDcl2	YPDcl3	EtOHcl1	EtOHcl2	Galcl1	YPDnc1	YPDnc2	YPDnc3	YPDnc4	EtOHnc1	EtOHnc2	Galnc1	Galnc2
13	AAD15	YOL165C	YPDcl2	YPDcl3	EtOHcl1	EtOHcl2	Galcl1	YPDnc1	YPDnc2	YPDnc3	YPDnc4	EtOHnc1	EtOHnc2	Galnc1	Galnc2
13	MTR2	YKL186C	YPDcl2	YPDcl3	EtOHcl1	EtOHcl2	Galcl1	YPDnc1	YPDnc2	YPDnc3	YPDnc4	EtOHnc1	EtOHnc2	Galnc1	Galnc2
13	GTB1	YDR221W	YPDcl2	YPDcl3	EtOHcl1	EtOHcl2	Galcl1	YPDnc1	YPDnc2	YPDnc3	YPDnc4	EtOHnc1	EtOHnc2	Galnc1	Galnc2
13	MES1	YGR264C	YPDcl2	YPDcl3	EtOHcl1	EtOHcl2	Galcl1	YPDnc1	YPDnc2	YPDnc3	YPDnc4	EtOHnc1	EtOHnc2	Galnc1	Galnc2
13	RPL41A	YDL184C	YPDcl2	YPDcl3	EtOHcl1	EtOHcl2	Galcl1	YPDnc1	YPDnc2	YPDnc3	YPDnc4	EtOHnc1	EtOHnc2	Galnc1	Galnc2
13	PTK1	YKL198C	YPDcl2	YPDcl3	EtOHcl1	EtOHcl2	Galcl1	YPDnc1	YPDnc2	YPDnc3	YPDnc4	EtOHnc1	EtOHnc2	Galnc1	Galnc2
13	EBS1	YDR206W	YPDcl2	YPDcl3	EtOHcl1	EtOHcl2	Galcl1	YPDnc1	YPDnc2	YPDnc3	YPDnc4	EtOHnc1	EtOHnc2	Galnc1	Galnc2
13		YLR112W	YPDcl2	YPDcl3	EtOHcl1	EtOHcl2	Galcl1	YPDnc1	YPDnc2	YPDnc3	YPDnc4	EtOHnc1	EtOHnc2	Galnc1	Galnc2

13	APL3	YBL037W	YPDcl2	YPDcl3	EtOHcl1	EtOHcl2	Galcl1	YPDnc1	YPDnc2	YPDnc3	YPDnc4	EtOHnc1	EtOHnc2	Galnc1	Galnc2
13	DAL5	YJR152W	YPDcl2	YPDcl3	EtOHcl1	EtOHcl2	Galcl1	YPDnc1	YPDnc2	YPDnc3	YPDnc4	EtOHnc1	EtOHnc2	Galnc1	Galnc2
13		YBL083C	YPDcl2	YPDcl3	EtOHcl1	EtOHcl2	Galcl1	YPDnc1	YPDnc2	YPDnc3	YPDnc4	EtOHnc1	EtOHnc2	Galnc1	Galnc2
13		YBR191W-/	YPDcl2	YPDcl3	EtOHcl1	EtOHcl2	Galcl1	YPDnc1	YPDnc2	YPDnc3	YPDnc4	EtOHnc1	EtOHnc2	Galnc1	Galnc2
13		YER068C-A	YPDcl2	YPDcl3	EtOHcl1	EtOHcl2	Galcl1	YPDnc1	YPDnc2	YPDnc3	YPDnc4	EtOHnc1	EtOHnc2	Galnc1	Galnc2
13	AAT1	YKL106W	YPDcl2	YPDcl3	EtOHcl1	EtOHcl2	Galcl1	YPDnc1	YPDnc2	YPDnc3	YPDnc4	EtOHnc1	EtOHnc2	Galnc1	Galnc2
13	DUG2	YBR281C	YPDcl2	YPDcl3	EtOHcl1	EtOHcl2	Galcl1	YPDnc1	YPDnc2	YPDnc3	YPDnc4	EtOHnc1	EtOHnc2	Galnc1	Galnc2
13	DNM1	YLL001W	YPDcl2	YPDcl3	EtOHcl1	EtOHcl2	Galcl1	YPDnc1	YPDnc2	YPDnc3	YPDnc4	EtOHnc1	EtOHnc2	Galnc1	Galnc2
13	RPC53	YDL150W	YPDcl2	YPDcl3	EtOHcl1	EtOHcl2	Galcl1	YPDnc1	YPDnc2	YPDnc3	YPDnc4	EtOHnc1	EtOHnc2	Galnc1	Galnc2
13	PFK27	YOL136C	YPDcl2	YPDcl3	EtOHcl1	EtOHcl2	Galcl1	YPDnc1	YPDnc2	YPDnc3	YPDnc4	EtOHnc1	EtOHnc2	Galnc1	Galnc2
13	FMP41	YNL168C	YPDcl2	YPDcl3	EtOHcl1	EtOHcl2	Galcl1	YPDnc1	YPDnc2	YPDnc3	YPDnc4	EtOHnc1	EtOHnc2	Galnc1	Galnc2
13		YIL077C	YPDcl2	YPDcl3	EtOHcl1	EtOHcl2	Galcl1	YPDnc1	YPDnc2	YPDnc3	YPDnc4	EtOHnc1	EtOHnc2	Galnc1	Galnc2
13	SPO14	YKR031C	YPDcl2	YPDcl3	EtOHcl1	EtOHcl2	Galcl1	YPDnc1	YPDnc2	YPDnc3	YPDnc4	EtOHnc1	EtOHnc2	Galnc1	Galnc2
13	DIM1	YPL266W	YPDcl2	YPDcl3	EtOHcl1	EtOHcl2	Galcl1	YPDnc1	YPDnc2	YPDnc3	YPDnc4	EtOHnc1	EtOHnc2	Galnc1	Galnc2
13	ECM14	YHR132C	YPDcl2	YPDcl3	EtOHcl1	EtOHcl2	Galcl1	YPDnc1	YPDnc2	YPDnc3	YPDnc4	EtOHnc1	EtOHnc2	Galnc1	Galnc2
13	SGD1	YLR336C	YPDcl2	YPDcl3	EtOHcl1	EtOHcl2	Galcl1	YPDnc1	YPDnc2	YPDnc3	YPDnc4	EtOHnc1	EtOHnc2	Galnc1	Galnc2
13	SPR6	YER115C	YPDcl2	YPDcl3	EtOHcl1	EtOHcl2	Galcl1	YPDnc1	YPDnc2	YPDnc3	YPDnc4	EtOHnc1	EtOHnc2	Galnc1	Galnc2
13	YLF2	YHL014C	YPDcl2	YPDcl3	EtOHcl1	EtOHcl2	Galcl1	YPDnc1	YPDnc2	YPDnc3	YPDnc4	EtOHnc1	EtOHnc2	Galnc1	Galnc2
13	ELP6	YMR312W	YPDcl2	YPDcl3	EtOHcl1	EtOHcl2	Galcl1	YPDnc1	YPDnc2	YPDnc3	YPDnc4	EtOHnc1	EtOHnc2	Galnc1	Galnc2
13	MSS11	YMR164C	YPDcl2	YPDcl3	EtOHcl1	EtOHcl2	Galcl1	YPDnc1	YPDnc2	YPDnc3	YPDnc4	EtOHnc1	EtOHnc2	Galnc1	Galnc2
13		YOR214C	YPDcl2	YPDcl3	EtOHcl1	EtOHcl2	Galcl1	YPDnc1	YPDnc2	YPDnc3	YPDnc4	EtOHnc1	EtOHnc2	Galnc1	Galnc2
13	RRN5	YLR141W	YPDcl2	YPDcl3	EtOHcl1	EtOHcl2	Galcl1	YPDnc1	YPDnc2	YPDnc3	YPDnc4	EtOHnc1	EtOHnc2	Galnc1	Galnc2
13	MAD2	YJL030W	YPDcl2	YPDcl3	EtOHcl1	EtOHcl2	Galcl1	YPDnc1	YPDnc2	YPDnc3	YPDnc4	EtOHnc1	EtOHnc2	Galnc1	Galnc2
13		YJR096W	YPDcl2	YPDcl3	EtOHcl1	EtOHcl2	Galcl1	YPDnc1	YPDnc2	YPDnc3	YPDnc4	EtOHnc1	EtOHnc2	Galnc1	Galnc2
13	SEC20	YDR498C	YPDcl2	YPDcl3	EtOHcl1	EtOHcl2	Galcl1	YPDnc1	YPDnc2	YPDnc3	YPDnc4	EtOHnc1	EtOHnc2	Galnc1	Galnc2
13	STE14	YDR410C	YPDcl2	YPDcl3	EtOHcl1	EtOHcl2	Galcl1	YPDnc1	YPDnc2	YPDnc3	YPDnc4	EtOHnc1	EtOHnc2	Galnc1	Galnc2
13	ALA1	YOR335C	YPDcl2	YPDcl3	EtOHcl1	EtOHcl2	Galcl1	YPDnc1	YPDnc2	YPDnc3	YPDnc4	EtOHnc1	EtOHnc2	Galnc1	Galnc2
13	TCB2	YNL087W	YPDcl2	YPDcl3	EtOHcl1	EtOHcl2	Galcl1	YPDnc1	YPDnc2	YPDnc3	YPDnc4	EtOHnc1	EtOHnc2	Galnc1	Galnc2
13	MIP6	YHR015W	YPDcl2	YPDcl3	EtOHcl1	EtOHcl2	Galcl1	YPDnc1	YPDnc2	YPDnc3	YPDnc4	EtOHnc1	EtOHnc2	Galnc1	Galnc2
13	PNC1	YGL037C	YPDcl2	YPDcl3	EtOHcl1	EtOHcl2	Galcl1	YPDnc1	YPDnc2	YPDnc3	YPDnc4	EtOHnc1	EtOHnc2	Galnc1	Galnc2
13	SAM2	YDR502C	YPDcl2	YPDcl3	EtOHcl1	EtOHcl2	Galcl1	YPDnc1	YPDnc2	YPDnc3	YPDnc4	EtOHnc1	EtOHnc2	Galnc1	Galnc2
13	EAF1	YDR359C	YPDcl2	YPDcl3	EtOHcl1	EtOHcl2	Galcl1	YPDnc1	YPDnc2	YPDnc3	YPDnc4	EtOHnc1	EtOHnc2	Galnc1	Galnc2
13	RFU1	YLR073C	YPDcl2	YPDcl3	EtOHcl1	EtOHcl2	Galcl1	YPDnc1	YPDnc2	YPDnc3	YPDnc4	EtOHnc1	EtOHnc2	Galnc1	Galnc2
13	CDC11	YJR076C	YPDcl2	YPDcl3	EtOHcl1	EtOHcl2	Galcl1	YPDnc1	YPDnc2	YPDnc3	YPDnc4	EtOHnc1	EtOHnc2	Galnc1	Galnc2

13	KIN3	YAR018C	YPDcl2	YPDcl3	EtOHcl1	EtOHcl2	Galcl1	YPDnc1	YPDnc2	YPDnc3	YPDnc4	EtOHnc1	EtOHnc2	Galnc1	Galnc2
13	CEX1	YOR112W	YPDcl2	YPDcl3	EtOHcl1	EtOHcl2	Galcl1	YPDnc1	YPDnc2	YPDnc3	YPDnc4	EtOHnc1	EtOHnc2	Galnc1	Galnc2
13		YBR121C-A	YPDcl2	YPDcl3	EtOHcl1	EtOHcl2	Galcl1	YPDnc1	YPDnc2	YPDnc3	YPDnc4	EtOHnc1	EtOHnc2	Galnc1	Galnc2
13	SHG1	YBR258C	YPDcl2	YPDcl3	EtOHcl1	EtOHcl2	Galcl1	YPDnc1	YPDnc2	YPDnc3	YPDnc4	EtOHnc1	EtOHnc2	Galnc1	Galnc2
13	TKL1	YPR074C	YPDcl2	YPDcl3	EtOHcl1	EtOHcl2	Galcl1	YPDnc1	YPDnc2	YPDnc3	YPDnc4	EtOHnc1	EtOHnc2	Galnc1	Galnc2
13		YHR162W	YPDcl2	YPDcl3	EtOHcl1	EtOHcl2	Galcl1	YPDnc1	YPDnc2	YPDnc3	YPDnc4	EtOHnc1	EtOHnc2	Galnc1	Galnc2
13		YFR020W	YPDcl2	YPDcl3	EtOHcl1	EtOHcl2	Galcl1	YPDnc1	YPDnc2	YPDnc3	YPDnc4	EtOHnc1	EtOHnc2	Galnc1	Galnc2
13	ULA1	YPL003W	YPDcl2	YPDcl3	EtOHcl1	EtOHcl2	Galcl1	YPDnc1	YPDnc2	YPDnc3	YPDnc4	EtOHnc1	EtOHnc2	Galnc1	Galnc2
13		YML089C	YPDcl2	YPDcl3	EtOHcl1	EtOHcl2	Galcl1	YPDnc1	YPDnc2	YPDnc3	YPDnc4	EtOHnc1	EtOHnc2	Galnc1	Galnc2
13		YLR297W	YPDcl2	YPDcl3	EtOHcl1	EtOHcl2	Galcl1	YPDnc1	YPDnc2	YPDnc3	YPDnc4	EtOHnc1	EtOHnc2	Galnc1	Galnc2
13	UGX2	YDL169C	YPDcl2	YPDcl3	EtOHcl1	EtOHcl2	Galcl1	YPDnc1	YPDnc2	YPDnc3	YPDnc4	EtOHnc1	EtOHnc2	Galnc1	Galnc2
13	RPL35B	YDL136W	YPDcl2	YPDcl3	EtOHcl1	EtOHcl2	Galcl1	YPDnc1	YPDnc2	YPDnc3	YPDnc4	EtOHnc1	EtOHnc2	Galnc1	Galnc2
13	RRI1	YDL216C	YPDcl2	YPDcl3	EtOHcl1	EtOHcl2	Galcl1	YPDnc1	YPDnc2	YPDnc3	YPDnc4	EtOHnc1	EtOHnc2	Galnc1	Galnc2
13	CDC14	YFR028C	YPDcl2	YPDcl3	EtOHcl1	EtOHcl2	Galcl1	YPDnc1	YPDnc2	YPDnc3	YPDnc4	EtOHnc1	EtOHnc2	Galnc1	Galnc2
13	ANS1	YHR126C	YPDcl2	YPDcl3	EtOHcl1	EtOHcl2	Galcl1	YPDnc1	YPDnc2	YPDnc3	YPDnc4	EtOHnc1	EtOHnc2	Galnc1	Galnc2
13	PHO80	YOL001W	YPDcl2	YPDcl3	EtOHcl1	EtOHcl2	Galcl1	YPDnc1	YPDnc2	YPDnc3	YPDnc4	EtOHnc1	EtOHnc2	Galnc1	Galnc2
13	BUL2	YML111W	YPDcl2	YPDcl3	EtOHcl1	EtOHcl2	Galcl1	YPDnc1	YPDnc2	YPDnc3	YPDnc4	EtOHnc1	EtOHnc2	Galnc1	Galnc2
13	SOK2	YMR016C	YPDcl2	YPDcl3	EtOHcl1	EtOHcl2	Galcl1	YPDnc1	YPDnc2	YPDnc3	YPDnc4	EtOHnc1	EtOHnc2	Galnc1	Galnc2
13		YKL023W	YPDcl2	YPDcl3	EtOHcl1	EtOHcl2	Galcl1	YPDnc1	YPDnc2	YPDnc3	YPDnc4	EtOHnc1	EtOHnc2	Galnc1	Galnc2
13	PEP1	YBL017C	YPDcl2	YPDcl3	EtOHcl1	EtOHcl2	Galcl1	YPDnc1	YPDnc2	YPDnc3	YPDnc4	EtOHnc1	EtOHnc2	Galnc1	Galnc2
13	HSL7	YBR133C	YPDcl2	YPDcl3	EtOHcl1	EtOHcl2	Galcl1	YPDnc1	YPDnc2	YPDnc3	YPDnc4	EtOHnc1	EtOHnc2	Galnc1	Galnc2
13		YDR015C	YPDcl2	YPDcl3	EtOHcl1	EtOHcl2	Galcl1	YPDnc1	YPDnc2	YPDnc3	YPDnc4	EtOHnc1	EtOHnc2	Galnc1	Galnc2
13	ECM3	YOR092W	YPDcl2	YPDcl3	EtOHcl1	EtOHcl2	Galcl1	YPDnc1	YPDnc2	YPDnc3	YPDnc4	EtOHnc1	EtOHnc2	Galnc1	Galnc2
13	SPT10	YJL127C	YPDcl2	YPDcl3	EtOHcl1	EtOHcl2	Galcl1	YPDnc1	YPDnc2	YPDnc3	YPDnc4	EtOHnc1	EtOHnc2	Galnc1	Galnc2
13	CSE2	YNR010W	YPDcl2	YPDcl3	EtOHcl1	EtOHcl2	Galcl1	YPDnc1	YPDnc2	YPDnc3	YPDnc4	EtOHnc1	EtOHnc2	Galnc1	Galnc2
13	HAT1	YPL001W	YPDcl2	YPDcl3	EtOHcl1	EtOHcl2	Galcl1	YPDnc1	YPDnc2	YPDnc3	YPDnc4	EtOHnc1	EtOHnc2	Galnc1	Galnc2
13		YMR247W-	YPDcl2	YPDcl3	EtOHcl1	EtOHcl2	Galcl1	YPDnc1	YPDnc2	YPDnc3	YPDnc4	EtOHnc1	EtOHnc2	Galnc1	Galnc2
13	IFA38	YBR159W	YPDcl2	YPDcl3	EtOHcl1	EtOHcl2	Galcl1	YPDnc1	YPDnc2	YPDnc3	YPDnc4	EtOHnc1	EtOHnc2	Galnc1	Galnc2
13	MTR3	YGR158C	YPDcl2	YPDcl3	EtOHcl1	EtOHcl2	Galcl1	YPDnc1	YPDnc2	YPDnc3	YPDnc4	EtOHnc1	EtOHnc2	Galnc1	Galnc2
13	RPB10	YOR210W	YPDcl2	YPDcl3	EtOHcl1	EtOHcl2	Galcl1	YPDnc1	YPDnc2	YPDnc3	YPDnc4	EtOHnc1	EtOHnc2	Galnc1	Galnc2
13	RRT16	YNL105W	YPDcl2	YPDcl3	EtOHcl1	EtOHcl2	Galcl1	YPDnc1	YPDnc2	YPDnc3	YPDnc4	EtOHnc1	EtOHnc2	Galnc1	Galnc2
13	TPO5	YKL174C	YPDcl2	YPDcl3	EtOHcl1	EtOHcl2	Galcl1	YPDnc1	YPDnc2	YPDnc3	YPDnc4	EtOHnc1	EtOHnc2	Galnc1	Galnc2
13	SHM2	YLR058C	YPDcl2	YPDcl3	EtOHcl1	EtOHcl2	Galcl1	YPDnc1	YPDnc2	YPDnc3	YPDnc4	EtOHnc1	EtOHnc2	Galnc1	Galnc2
13	ADE16	YLR028C	YPDcl2	YPDcl3	EtOHcl1	EtOHcl2	Galcl1	YPDnc1	YPDnc2	YPDnc3	YPDnc4	EtOHnc1	EtOHnc2	Galnc1	Galnc2

13	CTS1	YLR286C	YPDcl2	YPDcl3	EtOHcl1	EtOHcl2	Galcl1	YPDnc1	YPDnc2	YPDnc3	YPDnc4	EtOHnc1	EtOHnc2	Galnc1	Galnc2
13	CHK1	YBR274W	YPDcl2	YPDcl3	EtOHcl1	EtOHcl2	Galcl1	YPDnc1	YPDnc2	YPDnc3	YPDnc4	EtOHnc1	EtOHnc2	Galnc1	Galnc2
13	MTF2	YDL044C	YPDcl2	YPDcl3	EtOHcl1	EtOHcl2	Galcl1	YPDnc1	YPDnc2	YPDnc3	YPDnc4	EtOHnc1	EtOHnc2	Galnc1	Galnc2
13		YNL305C	YPDcl2	YPDcl3	EtOHcl1	EtOHcl2	Galcl1	YPDnc1	YPDnc2	YPDnc3	YPDnc4	EtOHnc1	EtOHnc2	Galnc1	Galnc2
13	SPT21	YMR179W	YPDcl2	YPDcl3	EtOHcl1	EtOHcl2	Galcl1	YPDnc1	YPDnc2	YPDnc3	YPDnc4	EtOHnc1	EtOHnc2	Galnc1	Galnc2
13	SER3	YER081W	YPDcl2	YPDcl3	EtOHcl1	EtOHcl2	Galcl1	YPDnc1	YPDnc2	YPDnc3	YPDnc4	EtOHnc1	EtOHnc2	Galnc1	Galnc2
13		YEL032C-A	YPDcl2	YPDcl3	EtOHcl1	EtOHcl2	Galcl1	YPDnc1	YPDnc2	YPDnc3	YPDnc4	EtOHnc1	EtOHnc2	Galnc1	Galnc2
13		YCR081C-A	YPDcl2	YPDcl3	EtOHcl1	EtOHcl2	Galcl1	YPDnc1	YPDnc2	YPDnc3	YPDnc4	EtOHnc1	EtOHnc2	Galnc1	Galnc2
13	YHC3	YJL059W	YPDcl2	YPDcl3	EtOHcl1	EtOHcl2	Galcl1	YPDnc1	YPDnc2	YPDnc3	YPDnc4	EtOHnc1	EtOHnc2	Galnc1	Galnc2
13	ASG1	YIL130W	YPDcl2	YPDcl3	EtOHcl1	EtOHcl2	Galcl1	YPDnc1	YPDnc2	YPDnc3	YPDnc4	EtOHnc1	EtOHnc2	Galnc1	Galnc2
13	IES6	YEL044W	YPDcl2	YPDcl3	EtOHcl1	EtOHcl2	Galcl1	YPDnc1	YPDnc2	YPDnc3	YPDnc4	EtOHnc1	EtOHnc2	Galnc1	Galnc2
13		YGL108C	YPDcl2	YPDcl3	EtOHcl1	EtOHcl2	Galcl1	YPDnc1	YPDnc2	YPDnc3	YPDnc4	EtOHnc1	EtOHnc2	Galnc1	Galnc2
13		YFR052C-A	YPDcl2	YPDcl3	EtOHcl1	EtOHcl2	Galcl1	YPDnc1	YPDnc2	YPDnc3	YPDnc4	EtOHnc1	EtOHnc2	Galnc1	Galnc2
13	MEK1	YOR351C	YPDcl2	YPDcl3	EtOHcl1	EtOHcl2	Galcl1	YPDnc1	YPDnc2	YPDnc3	YPDnc4	EtOHnc1	EtOHnc2	Galnc1	Galnc2
13	PIB2	YGL023C	YPDcl2	YPDcl3	EtOHcl1	EtOHcl2	Galcl1	YPDnc1	YPDnc2	YPDnc3	YPDnc4	EtOHnc1	EtOHnc2	Galnc1	Galnc2
13	PIN4	YBL051C	YPDcl2	YPDcl3	EtOHcl1	EtOHcl2	Galcl1	YPDnc1	YPDnc2	YPDnc3	YPDnc4	EtOHnc1	EtOHnc2	Galnc1	Galnc2
13	RFC2	YJR068W	YPDcl2	YPDcl3	EtOHcl1	EtOHcl2	Galcl1	YPDnc1	YPDnc2	YPDnc3	YPDnc4	EtOHnc1	EtOHnc2	Galnc1	Galnc2
13		YDR042C	YPDcl2	YPDcl3	EtOHcl1	EtOHcl2	Galcl1	YPDnc1	YPDnc2	YPDnc3	YPDnc4	EtOHnc1	EtOHnc2	Galnc1	Galnc2
13	SFA1	YDL168W	YPDcl2	YPDcl3	EtOHcl1	EtOHcl2	Galcl1	YPDnc1	YPDnc2	YPDnc3	YPDnc4	EtOHnc1	EtOHnc2	Galnc1	Galnc2
13	GAL1	YBR020W	YPDcl2	YPDcl3	EtOHcl1	EtOHcl2	Galcl1	YPDnc1	YPDnc2	YPDnc3	YPDnc4	EtOHnc1	EtOHnc2	Galnc1	Galnc2
13	FUN30	YAL019W	YPDcl2	YPDcl3	EtOHcl1	EtOHcl2	Galcl1	YPDnc1	YPDnc2	YPDnc3	YPDnc4	EtOHnc1	EtOHnc2	Galnc1	Galnc2
13	VPS52	YDR484W	YPDcl2	YPDcl3	EtOHcl1	EtOHcl2	Galcl1	YPDnc1	YPDnc2	YPDnc3	YPDnc4	EtOHnc1	EtOHnc2	Galnc1	Galnc2
13	RAD23	YEL037C	YPDcl2	YPDcl3	EtOHcl1	EtOHcl2	Galcl1	YPDnc1	YPDnc2	YPDnc3	YPDnc4	EtOHnc1	EtOHnc2	Galnc1	Galnc2
13	UBX7	YBR273C	YPDcl2	YPDcl3	EtOHcl1	EtOHcl2	Galcl1	YPDnc1	YPDnc2	YPDnc3	YPDnc4	EtOHnc1	EtOHnc2	Galnc1	Galnc2
13	FUS1	YCL027W	YPDcl2	YPDcl3	EtOHcl1	EtOHcl2	Galcl1	YPDnc1	YPDnc2	YPDnc3	YPDnc4	EtOHnc1	EtOHnc2	Galnc1	Galnc2
13		YDL114W	YPDcl2	YPDcl3	EtOHcl1	EtOHcl2	Galcl1	YPDnc1	YPDnc2	YPDnc3	YPDnc4	EtOHnc1	EtOHnc2	Galnc1	Galnc2
13	ARC15	YIL062C	YPDcl2	YPDcl3	EtOHcl1	EtOHcl2	Galcl1	YPDnc1	YPDnc2	YPDnc3	YPDnc4	EtOHnc1	EtOHnc2	Galnc1	Galnc2
13	NDD1	YOR372C	YPDcl2	YPDcl3	EtOHcl1	EtOHcl2	Galcl1	YPDnc1	YPDnc2	YPDnc3	YPDnc4	EtOHnc1	EtOHnc2	Galnc1	Galnc2
13	USE1	YGL098W	YPDcl2	YPDcl3	EtOHcl1	EtOHcl2	Galcl1	YPDnc1	YPDnc2	YPDnc3	YPDnc4	EtOHnc1	EtOHnc2	Galnc1	Galnc2
13	PEX6	YNL329C	YPDcl2	YPDcl3	EtOHcl1	EtOHcl2	Galcl1	YPDnc1	YPDnc2	YPDnc3	YPDnc4	EtOHnc1	EtOHnc2	Galnc1	Galnc2
13	CLN1	YMR199W	YPDcl2	YPDcl3	EtOHcl1	EtOHcl2	Galcl1	YPDnc1	YPDnc2	YPDnc3	YPDnc4	EtOHnc1	EtOHnc2	Galnc1	Galnc2
13	CWC2	YDL209C	YPDcl2	YPDcl3	EtOHcl1	EtOHcl2	Galcl1	YPDnc1	YPDnc2	YPDnc3	YPDnc4	EtOHnc1	EtOHnc2	Galnc1	Galnc2
13	CDC39	YCR093W	YPDcl2	YPDcl3	EtOHcl1	EtOHcl2	Galcl1	YPDnc1	YPDnc2	YPDnc3	YPDnc4	EtOHnc1	EtOHnc2	Galnc1	Galnc2
13	ADE4	YMR300C	YPDcl2	YPDcl3	EtOHcl1	EtOHcl2	Galcl1	YPDnc1	YPDnc2	YPDnc3	YPDnc4	EtOHnc1	EtOHnc2	Galnc1	Galnc2

13	PAM18	YLR008C	YPDcl2	YPDcl3	EtOHcl1	EtOHcl2	Galcl1	YPDnc1	YPDnc2	YPDnc3	YPDnc4	EtOHnc1	EtOHnc2	Galnc1	Galnc2
13	RGI2	YIL057C	YPDcl2	YPDcl3	EtOHcl1	EtOHcl2	Galcl1	YPDnc1	YPDnc2	YPDnc3	YPDnc4	EtOHnc1	EtOHnc2	Galnc1	Galnc2
13	PFK26	YIL107C	YPDcl2	YPDcl3	EtOHcl1	EtOHcl2	Galcl1	YPDnc1	YPDnc2	YPDnc3	YPDnc4	EtOHnc1	EtOHnc2	Galnc1	Galnc2
13		YBL107W-A	YPDcl2	YPDcl3	EtOHcl1	EtOHcl2	Galcl1	YPDnc1	YPDnc2	YPDnc3	YPDnc4	EtOHnc1	EtOHnc2	Galnc1	Galnc2
13	MAS2	YHR024C	YPDcl2	YPDcl3	EtOHcl1	EtOHcl2	Galcl1	YPDnc1	YPDnc2	YPDnc3	YPDnc4	EtOHnc1	EtOHnc2	Galnc1	Galnc2
13	YPS1	YLR120C	YPDcl2	YPDcl3	EtOHcl1	EtOHcl2	Galcl1	YPDnc1	YPDnc2	YPDnc3	YPDnc4	EtOHnc1	EtOHnc2	Galnc1	Galnc2
13		YPR089W	YPDcl2	YPDcl3	EtOHcl1	EtOHcl2	Galcl1	YPDnc1	YPDnc2	YPDnc3	YPDnc4	EtOHnc1	EtOHnc2	Galnc1	Galnc2
13	YAP1	YML007W	YPDcl2	YPDcl3	EtOHcl1	EtOHcl2	Galcl1	YPDnc1	YPDnc2	YPDnc3	YPDnc4	EtOHnc1	EtOHnc2	Galnc1	Galnc2
13		YEL030C-A	YPDcl2	YPDcl3	EtOHcl1	EtOHcl2	Galcl1	YPDnc1	YPDnc2	YPDnc3	YPDnc4	EtOHnc1	EtOHnc2	Galnc1	Galnc2
13		YPL039W	YPDcl2	YPDcl3	EtOHcl1	EtOHcl2	Galcl1	YPDnc1	YPDnc2	YPDnc3	YPDnc4	EtOHnc1	EtOHnc2	Galnc1	Galnc2
13	THI7	YLR237W	YPDcl2	YPDcl3	EtOHcl1	EtOHcl2	Galcl1	YPDnc1	YPDnc2	YPDnc3	YPDnc4	EtOHnc1	EtOHnc2	Galnc1	Galnc2
13	MSH1	YHR120W	YPDcl2	YPDcl3	EtOHcl1	EtOHcl2	Galcl1	YPDnc1	YPDnc2	YPDnc3	YPDnc4	EtOHnc1	EtOHnc2	Galnc1	Galnc2
13	RPL22B	YFL034C-A	YPDcl2	YPDcl3	EtOHcl1	EtOHcl2	Galcl1	YPDnc1	YPDnc2	YPDnc3	YPDnc4	EtOHnc1	EtOHnc2	Galnc1	Galnc2
13	IRC15	YPL017C	YPDcl2	YPDcl3	EtOHcl1	EtOHcl2	Galcl1	YPDnc1	YPDnc2	YPDnc3	YPDnc4	EtOHnc1	EtOHnc2	Galnc1	Galnc2
13	GCR1	YPL075W	YPDcl2	YPDcl3	EtOHcl1	EtOHcl2	Galcl1	YPDnc1	YPDnc2	YPDnc3	YPDnc4	EtOHnc1	EtOHnc2	Galnc1	Galnc2
13	GLO1	YML004C	YPDcl2	YPDcl3	EtOHcl1	EtOHcl2	Galcl1	YPDnc1	YPDnc2	YPDnc3	YPDnc4	EtOHnc1	EtOHnc2	Galnc1	Galnc2
13		YNL097W-A	YPDcl2	YPDcl3	EtOHcl1	EtOHcl2	Galcl1	YPDnc1	YPDnc2	YPDnc3	YPDnc4	EtOHnc1	EtOHnc2	Galnc1	Galnc2
13	GSG1	YDR108W	YPDcl2	YPDcl3	EtOHcl1	EtOHcl2	Galcl1	YPDnc1	YPDnc2	YPDnc3	YPDnc4	EtOHnc1	EtOHnc2	Galnc1	Galnc2
13		YKL102C	YPDcl2	YPDcl3	EtOHcl1	EtOHcl2	Galcl1	YPDnc1	YPDnc2	YPDnc3	YPDnc4	EtOHnc1	EtOHnc2	Galnc1	Galnc2
13	SLM6	YBR266C	YPDcl2	YPDcl3	EtOHcl1	EtOHcl2	Galcl1	YPDnc1	YPDnc2	YPDnc3	YPDnc4	EtOHnc1	EtOHnc2	Galnc1	Galnc2
13	BUD16	YEL029C	YPDcl2	YPDcl3	EtOHcl1	EtOHcl2	Galcl1	YPDnc1	YPDnc2	YPDnc3	YPDnc4	EtOHnc1	EtOHnc2	Galnc1	Galnc2
13	SDA1	YGR245C	YPDcl2	YPDcl3	EtOHcl1	EtOHcl2	Galcl1	YPDnc1	YPDnc2	YPDnc3	YPDnc4	EtOHnc1	EtOHnc2	Galnc1	Galnc2
13		YNL040W	YPDcl2	YPDcl3	EtOHcl1	EtOHcl2	Galcl1	YPDnc1	YPDnc2	YPDnc3	YPDnc4	EtOHnc1	EtOHnc2	Galnc1	Galnc2
13	CCP1	YKR066C	YPDcl2	YPDcl3	EtOHcl1	EtOHcl2	Galcl1	YPDnc1	YPDnc2	YPDnc3	YPDnc4	EtOHnc1	EtOHnc2	Galnc1	Galnc2
13	YIP5	YGL161C	YPDcl2	YPDcl3	EtOHcl1	EtOHcl2	Galcl1	YPDnc1	YPDnc2	YPDnc3	YPDnc4	EtOHnc1	EtOHnc2	Galnc1	Galnc2
13		YMR105W-	YPDcl2	YPDcl3	EtOHcl1	EtOHcl2	Galcl1	YPDnc1	YPDnc2	YPDnc3	YPDnc4	EtOHnc1	EtOHnc2	Galnc1	Galnc2
13	NOC4	YPR144C	YPDcl2	YPDcl3	EtOHcl1	EtOHcl2	Galcl1	YPDnc1	YPDnc2	YPDnc3	YPDnc4	EtOHnc1	EtOHnc2	Galnc1	Galnc2
13	AGP3	YFL055W	YPDcl2	YPDcl3	EtOHcl1	EtOHcl2	Galcl1	YPDnc1	YPDnc2	YPDnc3	YPDnc4	EtOHnc1	EtOHnc2	Galnc1	Galnc2
13		YIL086C	YPDcl2	YPDcl3	EtOHcl1	EtOHcl2	Galcl1	YPDnc1	YPDnc2	YPDnc3	YPDnc4	EtOHnc1	EtOHnc2	Galnc1	Galnc2
13	TMA20	YER007C-A	YPDcl2	YPDcl3	EtOHcl1	EtOHcl2	Galcl1	YPDnc1	YPDnc2	YPDnc3	YPDnc4	EtOHnc1	EtOHnc2	Galnc1	Galnc2
13	MSL5	YLR116W	YPDcl2	YPDcl3	EtOHcl1	EtOHcl2	Galcl1	YPDnc1	YPDnc2	YPDnc3	YPDnc4	EtOHnc1	EtOHnc2	Galnc1	Galnc2
13		YAL047W-A	YPDcl2	YPDcl3	EtOHcl1	EtOHcl2	Galcl1	YPDnc1	YPDnc2	YPDnc3	YPDnc4	EtOHnc1	EtOHnc2	Galnc1	Galnc2
13	COX10	YPL172C	YPDcl2	YPDcl3	EtOHcl1	EtOHcl2	Galcl1	YPDnc1	YPDnc2	YPDnc3	YPDnc4	EtOHnc1	EtOHnc2	Galnc1	Galnc2
13	MMT2	YPL224C	YPDcl2	YPDcl3	EtOHcl1	EtOHcl2	Galcl1	YPDnc1	YPDnc2	YPDnc3	YPDnc4	EtOHnc1	EtOHnc2	Galnc1	Galnc2

13	ADH3	YMR083W	YPDcl2	YPDcl3	EtOHcl1	EtOHcl2	Galcl1	YPDnc1	YPDnc2	YPDnc3	YPDnc4	EtOHnc1	EtOHnc2	Galnc1	Galnc2
13	TPP1	YMR156C	YPDcl2	YPDcl3	EtOHcl1	EtOHcl2	Galcl1	YPDnc1	YPDnc2	YPDnc3	YPDnc4	EtOHnc1	EtOHnc2	Galnc1	Galnc2
13	PIL1	YGR086C	YPDcl2	YPDcl3	EtOHcl1	EtOHcl2	Galcl1	YPDnc1	YPDnc2	YPDnc3	YPDnc4	EtOHnc1	EtOHnc2	Galnc1	Galnc2
13	SCH9	YHR205W	YPDcl2	YPDcl3	EtOHcl1	EtOHcl2	Galcl1	YPDnc1	YPDnc2	YPDnc3	YPDnc4	EtOHnc1	EtOHnc2	Galnc1	Galnc2
13	YOR1	YGR281W	YPDcl2	YPDcl3	EtOHcl1	EtOHcl2	Galcl1	YPDnc1	YPDnc2	YPDnc3	YPDnc4	EtOHnc1	EtOHnc2	Galnc1	Galnc2
13	ARE1	YCR048W	YPDcl2	YPDcl3	EtOHcl1	EtOHcl2	Galcl1	YPDnc1	YPDnc2	YPDnc3	YPDnc4	EtOHnc1	EtOHnc2	Galnc1	Galnc2
13	PEX19	YDL065C	YPDcl2	YPDcl3	EtOHcl1	EtOHcl2	Galcl1	YPDnc1	YPDnc2	YPDnc3	YPDnc4	EtOHnc1	EtOHnc2	Galnc1	Galnc2
13	DLT1	YMR126C	YPDcl2	YPDcl3	EtOHcl1	EtOHcl2	Galcl1	YPDnc1	YPDnc2	YPDnc3	YPDnc4	EtOHnc1	EtOHnc2	Galnc1	Galnc2
13	ENV6	YEL059W	YPDcl2	YPDcl3	EtOHcl1	EtOHcl2	Galcl1	YPDnc1	YPDnc2	YPDnc3	YPDnc4	EtOHnc1	EtOHnc2	Galnc1	Galnc2
13		YJL195C	YPDcl2	YPDcl3	EtOHcl1	EtOHcl2	Galcl1	YPDnc1	YPDnc2	YPDnc3	YPDnc4	EtOHnc1	EtOHnc2	Galnc1	Galnc2
13	BNA7	YDR428C	YPDcl2	YPDcl3	EtOHcl1	EtOHcl2	Galcl1	YPDnc1	YPDnc2	YPDnc3	YPDnc4	EtOHnc1	EtOHnc2	Galnc1	Galnc2
13	FIR1	YER032W	YPDcl2	YPDcl3	EtOHcl1	EtOHcl2	Galcl1	YPDnc1	YPDnc2	YPDnc3	YPDnc4	EtOHnc1	EtOHnc2	Galnc1	Galnc2
13	XKS1	YGR194C	YPDcl2	YPDcl3	EtOHcl1	EtOHcl2	Galcl1	YPDnc1	YPDnc2	YPDnc3	YPDnc4	EtOHnc1	EtOHnc2	Galnc1	Galnc2
13		YHR056W-/	YPDcl2	YPDcl3	EtOHcl1	EtOHcl2	Galcl1	YPDnc1	YPDnc2	YPDnc3	YPDnc4	EtOHnc1	EtOHnc2	Galnc1	Galnc2
13	PAC10	YGR078C	YPDcl2	YPDcl3	EtOHcl1	EtOHcl2	Galcl1	YPDnc1	YPDnc2	YPDnc3	YPDnc4	EtOHnc1	EtOHnc2	Galnc1	Galnc2
13		YMR265C	YPDcl2	YPDcl3	EtOHcl1	EtOHcl2	Galcl1	YPDnc1	YPDnc2	YPDnc3	YPDnc4	EtOHnc1	EtOHnc2	Galnc1	Galnc2
13	SOL3	YHR163W	YPDcl2	YPDcl3	EtOHcl1	EtOHcl2	Galcl1	YPDnc1	YPDnc2	YPDnc3	YPDnc4	EtOHnc1	EtOHnc2	Galnc1	Galnc2
13	ODC2	YOR222W	YPDcl2	YPDcl3	EtOHcl1	EtOHcl2	Galcl1	YPDnc1	YPDnc2	YPDnc3	YPDnc4	EtOHnc1	EtOHnc2	Galnc1	Galnc2
13	RTG3	YBL103C	YPDcl2	YPDcl3	EtOHcl1	EtOHcl2	Galcl1	YPDnc1	YPDnc2	YPDnc3	YPDnc4	EtOHnc1	EtOHnc2	Galnc1	Galnc2
13		YLR358C	YPDcl2	YPDcl3	EtOHcl1	EtOHcl2	Galcl1	YPDnc1	YPDnc2	YPDnc3	YPDnc4	EtOHnc1	EtOHnc2	Galnc1	Galnc2
13	SEN15	YMR059W	YPDcl2	YPDcl3	EtOHcl1	EtOHcl2	Galcl1	YPDnc1	YPDnc2	YPDnc3	YPDnc4	EtOHnc1	EtOHnc2	Galnc1	Galnc2
13	ESC8	YOL017W	YPDcl2	YPDcl3	EtOHcl1	EtOHcl2	Galcl1	YPDnc1	YPDnc2	YPDnc3	YPDnc4	EtOHnc1	EtOHnc2	Galnc1	Galnc2
13	NAM2	YLR382C	YPDcl2	YPDcl3	EtOHcl1	EtOHcl2	Galcl1	YPDnc1	YPDnc2	YPDnc3	YPDnc4	EtOHnc1	EtOHnc2	Galnc1	Galnc2
13		YBR242W	YPDcl2	YPDcl3	EtOHcl1	EtOHcl2	Galcl1	YPDnc1	YPDnc2	YPDnc3	YPDnc4	EtOHnc1	EtOHnc2	Galnc1	Galnc2
13	HAP3	YBL021C	YPDcl2	YPDcl3	EtOHcl1	EtOHcl2	Galcl1	YPDnc1	YPDnc2	YPDnc3	YPDnc4	EtOHnc1	EtOHnc2	Galnc1	Galnc2
13	PRE1	YER012W	YPDcl2	YPDcl3	EtOHcl1	EtOHcl2	Galcl1	YPDnc1	YPDnc2	YPDnc3	YPDnc4	EtOHnc1	EtOHnc2	Galnc1	Galnc2
13		YKL068W-/	YPDcl2	YPDcl3	EtOHcl1	EtOHcl2	Galcl1	YPDnc1	YPDnc2	YPDnc3	YPDnc4	EtOHnc1	EtOHnc2	Galnc1	Galnc2
13	HUL5	YGL141W	YPDcl2	YPDcl3	EtOHcl1	EtOHcl2	Galcl1	YPDnc1	YPDnc2	YPDnc3	YPDnc4	EtOHnc1	EtOHnc2	Galnc1	Galnc2
13	HEH2	YDR458C	YPDcl2	YPDcl3	EtOHcl1	EtOHcl2	Galcl1	YPDnc1	YPDnc2	YPDnc3	YPDnc4	EtOHnc1	EtOHnc2	Galnc1	Galnc2
13	HST1	YOL068C	YPDcl2	YPDcl3	EtOHcl1	EtOHcl2	Galcl1	YPDnc1	YPDnc2	YPDnc3	YPDnc4	EtOHnc1	EtOHnc2	Galnc1	Galnc2
13	AVL9	YLR114C	YPDcl2	YPDcl3	EtOHcl1	EtOHcl2	Galcl1	YPDnc1	YPDnc2	YPDnc3	YPDnc4	EtOHnc1	EtOHnc2	Galnc1	Galnc2
13	NPA3	YJR072C	YPDcl2	YPDcl3	EtOHcl1	EtOHcl2	Galcl1	YPDnc1	YPDnc2	YPDnc3	YPDnc4	EtOHnc1	EtOHnc2	Galnc1	Galnc2
13	CIA1	YDR267C	YPDcl2	YPDcl3	EtOHcl1	EtOHcl2	Galcl1	YPDnc1	YPDnc2	YPDnc3	YPDnc4	EtOHnc1	EtOHnc2	Galnc1	Galnc2
13	MPS3	YJL019W	YPDcl2	YPDcl3	EtOHcl1	EtOHcl2	Galcl1	YPDnc1	YPDnc2	YPDnc3	YPDnc4	EtOHnc1	EtOHnc2	Galnc1	Galnc2

13	HIS6	YIL020C	YPDcl2	YPDcl3	EtOHcl1	EtOHcl2	Galcl1	YPDnc1	YPDnc2	YPDnc3	YPDnc4	EtOHnc1	EtOHnc2	Galnc1	Galnc2
13		YOR073W-,	YPDcl2	YPDcl3	EtOHcl1	EtOHcl2	Galcl1	YPDnc1	YPDnc2	YPDnc3	YPDnc4	EtOHnc1	EtOHnc2	Galnc1	Galnc2
13	MTQ1	YNL063W	YPDcl2	YPDcl3	EtOHcl1	EtOHcl2	Galcl1	YPDnc1	YPDnc2	YPDnc3	YPDnc4	EtOHnc1	EtOHnc2	Galnc1	Galnc2
13	DUG1	YFR044C	YPDcl2	YPDcl3	EtOHcl1	EtOHcl2	Galcl1	YPDnc1	YPDnc2	YPDnc3	YPDnc4	EtOHnc1	EtOHnc2	Galnc1	Galnc2
13	TAO3	YIL129C	YPDcl2	YPDcl3	EtOHcl1	EtOHcl2	Galcl1	YPDnc1	YPDnc2	YPDnc3	YPDnc4	EtOHnc1	EtOHnc2	Galnc1	Galnc2
13		YGL176C	YPDcl2	YPDcl3	EtOHcl1	EtOHcl2	Galcl1	YPDnc1	YPDnc2	YPDnc3	YPDnc4	EtOHnc1	EtOHnc2	Galnc1	Galnc2
13	MNN10	YDR245W	YPDcl2	YPDcl3	EtOHcl1	EtOHcl2	Galcl1	YPDnc1	YPDnc2	YPDnc3	YPDnc4	EtOHnc1	EtOHnc2	Galnc1	Galnc2
13	TCP1	YDR212W	YPDcl2	YPDcl3	EtOHcl1	EtOHcl2	Galcl1	YPDnc1	YPDnc2	YPDnc3	YPDnc4	EtOHnc1	EtOHnc2	Galnc1	Galnc2
13		YDR149C	YPDcl2	YPDcl3	EtOHcl1	EtOHcl2	Galcl1	YPDnc1	YPDnc2	YPDnc3	YPDnc4	EtOHnc1	EtOHnc2	Galnc1	Galnc2
13	OST2	YOR103C	YPDcl2	YPDcl3	EtOHcl1	EtOHcl2	Galcl1	YPDnc1	YPDnc2	YPDnc3	YPDnc4	EtOHnc1	EtOHnc2	Galnc1	Galnc2
13	SEC63	YOR254C	YPDcl2	YPDcl3	EtOHcl1	EtOHcl2	Galcl1	YPDnc1	YPDnc2	YPDnc3	YPDnc4	EtOHnc1	EtOHnc2	Galnc1	Galnc2
13	YPK1	YKL126W	YPDcl2	YPDcl3	EtOHcl1	EtOHcl2	Galcl1	YPDnc1	YPDnc2	YPDnc3	YPDnc4	EtOHnc1	EtOHnc2	Galnc1	Galnc2
13	RPS1A	YLR441C	YPDcl2	YPDcl3	EtOHcl1	EtOHcl2	Galcl1	YPDnc1	YPDnc2	YPDnc3	YPDnc4	EtOHnc1	EtOHnc2	Galnc1	Galnc2
13		YFR012W	YPDcl2	YPDcl3	EtOHcl1	EtOHcl2	Galcl1	YPDnc1	YPDnc2	YPDnc3	YPDnc4	EtOHnc1	EtOHnc2	Galnc1	Galnc2
13		YBR204C	YPDcl2	YPDcl3	EtOHcl1	EtOHcl2	Galcl1	YPDnc1	YPDnc2	YPDnc3	YPDnc4	EtOHnc1	EtOHnc2	Galnc1	Galnc2
13	BUD13	YGL174W	YPDcl2	YPDcl3	EtOHcl1	EtOHcl2	Galcl1	YPDnc1	YPDnc2	YPDnc3	YPDnc4	EtOHnc1	EtOHnc2	Galnc1	Galnc2
13	PDR3	YBL005W	YPDcl2	YPDcl3	EtOHcl1	EtOHcl2	Galcl1	YPDnc1	YPDnc2	YPDnc3	YPDnc4	EtOHnc1	EtOHnc2	Galnc1	Galnc2
13		YIL108W	YPDcl2	YPDcl3	EtOHcl1	EtOHcl2	Galcl1	YPDnc1	YPDnc2	YPDnc3	YPDnc4	EtOHnc1	EtOHnc2	Galnc1	Galnc2
13	RPB7	YDR404C	YPDcl2	YPDcl3	EtOHcl1	EtOHcl2	Galcl1	YPDnc1	YPDnc2	YPDnc3	YPDnc4	EtOHnc1	EtOHnc2	Galnc1	Galnc2
13	ASE1	YOR058C	YPDcl2	YPDcl3	EtOHcl1	EtOHcl2	Galcl1	YPDnc1	YPDnc2	YPDnc3	YPDnc4	EtOHnc1	EtOHnc2	Galnc1	Galnc2
13		YBL104C	YPDcl2	YPDcl3	EtOHcl1	EtOHcl2	Galcl1	YPDnc1	YPDnc2	YPDnc3	YPDnc4	EtOHnc1	EtOHnc2	Galnc1	Galnc2
13	RXT2	YBR095C	YPDcl2	YPDcl3	EtOHcl1	EtOHcl2	Galcl1	YPDnc1	YPDnc2	YPDnc3	YPDnc4	EtOHnc1	EtOHnc2	Galnc1	Galnc2
13	BET1	YIL004C	YPDcl2	YPDcl3	EtOHcl1	EtOHcl2	Galcl1	YPDnc1	YPDnc2	YPDnc3	YPDnc4	EtOHnc1	EtOHnc2	Galnc1	Galnc2
13	TOR2	YKL203C	YPDcl2	YPDcl3	EtOHcl1	EtOHcl2	Galcl1	YPDnc1	YPDnc2	YPDnc3	YPDnc4	EtOHnc1	EtOHnc2	Galnc1	Galnc2
13	MCH2	YKL221W	YPDcl2	YPDcl3	EtOHcl1	EtOHcl2	Galcl1	YPDnc1	YPDnc2	YPDnc3	YPDnc4	EtOHnc1	EtOHnc2	Galnc1	Galnc2
13	IPI1	YHR085W	YPDcl2	YPDcl3	EtOHcl1	EtOHcl2	Galcl1	YPDnc1	YPDnc2	YPDnc3	YPDnc4	EtOHnc1	EtOHnc2	Galnc1	Galnc2
13	UGA4	YDL210W	YPDcl2	YPDcl3	EtOHcl1	EtOHcl2	Galcl1	YPDnc1	YPDnc2	YPDnc3	YPDnc4	EtOHnc1	EtOHnc2	Galnc1	Galnc2
13	MRS4	YKR052C	YPDcl2	YPDcl3	EtOHcl1	EtOHcl2	Galcl1	YPDnc1	YPDnc2	YPDnc3	YPDnc4	EtOHnc1	EtOHnc2	Galnc1	Galnc2
13	CDC37	YDR168W	YPDcl2	YPDcl3	EtOHcl1	EtOHcl2	Galcl1	YPDnc1	YPDnc2	YPDnc3	YPDnc4	EtOHnc1	EtOHnc2	Galnc1	Galnc2
13	KRI1	YNL308C	YPDcl2	YPDcl3	EtOHcl1	EtOHcl2	Galcl1	YPDnc1	YPDnc2	YPDnc3	YPDnc4	EtOHnc1	EtOHnc2	Galnc1	Galnc2
13	LCB4	YOR171C	YPDcl2	YPDcl3	EtOHcl1	EtOHcl2	Galcl1	YPDnc1	YPDnc2	YPDnc3	YPDnc4	EtOHnc1	EtOHnc2	Galnc1	Galnc2
13	TRX1	YLR043C	YPDcl2	YPDcl3	EtOHcl1	EtOHcl2	Galcl1	YPDnc1	YPDnc2	YPDnc3	YPDnc4	EtOHnc1	EtOHnc2	Galnc1	Galnc2
13	ATP1	YBL099W	YPDcl2	YPDcl3	EtOHcl1	EtOHcl2	Galcl1	YPDnc1	YPDnc2	YPDnc3	YPDnc4	EtOHnc1	EtOHnc2	Galnc1	Galnc2
13	BMS1	YPL217C	YPDcl2	YPDcl3	EtOHcl1	EtOHcl2	Galcl1	YPDnc1	YPDnc2	YPDnc3	YPDnc4	EtOHnc1	EtOHnc2	Galnc1	Galnc2

13	HES1	YOR237W	YPDcl2	YPDcl3	EtOHcl1	EtOHcl2	Galcl1	YPDnc1	YPDnc2	YPDnc3	YPDnc4	EtOHnc1	EtOHnc2	Galnc1	Galnc2
13	COX12	YLR038C	YPDcl2	YPDcl3	EtOHcl1	EtOHcl2	Galcl1	YPDnc1	YPDnc2	YPDnc3	YPDnc4	EtOHnc1	EtOHnc2	Galnc1	Galnc2
13		YMR030W-	YPDcl2	YPDcl3	EtOHcl1	EtOHcl2	Galcl1	YPDnc1	YPDnc2	YPDnc3	YPDnc4	EtOHnc1	EtOHnc2	Galnc1	Galnc2
13	TAF9	YMR236W	YPDcl2	YPDcl3	EtOHcl1	EtOHcl2	Galcl1	YPDnc1	YPDnc2	YPDnc3	YPDnc4	EtOHnc1	EtOHnc2	Galnc1	Galnc2
13		YJR056C	YPDcl2	YPDcl3	EtOHcl1	EtOHcl2	Galcl1	YPDnc1	YPDnc2	YPDnc3	YPDnc4	EtOHnc1	EtOHnc2	Galnc1	Galnc2
13		YPR130C	YPDcl2	YPDcl3	EtOHcl1	EtOHcl2	Galcl1	YPDnc1	YPDnc2	YPDnc3	YPDnc4	EtOHnc1	EtOHnc2	Galnc1	Galnc2
13	RPS30A	YLR287C-A	YPDcl2	YPDcl3	EtOHcl1	EtOHcl2	Galcl1	YPDnc1	YPDnc2	YPDnc3	YPDnc4	EtOHnc1	EtOHnc2	Galnc1	Galnc2
13	HVG1	YER039C	YPDcl2	YPDcl3	EtOHcl1	EtOHcl2	Galcl1	YPDnc1	YPDnc2	YPDnc3	YPDnc4	EtOHnc1	EtOHnc2	Galnc1	Galnc2
13	DAL82	YNL314W	YPDcl2	YPDcl3	EtOHcl1	EtOHcl2	Galcl1	YPDnc1	YPDnc2	YPDnc3	YPDnc4	EtOHnc1	EtOHnc2	Galnc1	Galnc2
13		YDR387C	YPDcl2	YPDcl3	EtOHcl1	EtOHcl2	Galcl1	YPDnc1	YPDnc2	YPDnc3	YPDnc4	EtOHnc1	EtOHnc2	Galnc1	Galnc2
13	PCI8	YIL071C	YPDcl2	YPDcl3	EtOHcl1	EtOHcl2	Galcl1	YPDnc1	YPDnc2	YPDnc3	YPDnc4	EtOHnc1	EtOHnc2	Galnc1	Galnc2
13	STB3	YDR169C	YPDcl2	YPDcl3	EtOHcl1	EtOHcl2	Galcl1	YPDnc1	YPDnc2	YPDnc3	YPDnc4	EtOHnc1	EtOHnc2	Galnc1	Galnc2
13		YBL094C	YPDcl2	YPDcl3	EtOHcl1	EtOHcl2	Galcl1	YPDnc1	YPDnc2	YPDnc3	YPDnc4	EtOHnc1	EtOHnc2	Galnc1	Galnc2
13		YGL177W	YPDcl2	YPDcl3	EtOHcl1	EtOHcl2	Galcl1	YPDnc1	YPDnc2	YPDnc3	YPDnc4	EtOHnc1	EtOHnc2	Galnc1	Galnc2
13		YDR333C	YPDcl2	YPDcl3	EtOHcl1	EtOHcl2	Galcl1	YPDnc1	YPDnc2	YPDnc3	YPDnc4	EtOHnc1	EtOHnc2	Galnc1	Galnc2
13		YDR124W	YPDcl2	YPDcl3	EtOHcl1	EtOHcl2	Galcl1	YPDnc1	YPDnc2	YPDnc3	YPDnc4	EtOHnc1	EtOHnc2	Galnc1	Galnc2
13	OPI7	YDR360W	YPDcl2	YPDcl3	EtOHcl1	EtOHcl2	Galcl1	YPDnc1	YPDnc2	YPDnc3	YPDnc4	EtOHnc1	EtOHnc2	Galnc1	Galnc2
13		YDR115W	YPDcl2	YPDcl3	EtOHcl1	EtOHcl2	Galcl1	YPDnc1	YPDnc2	YPDnc3	YPDnc4	EtOHnc1	EtOHnc2	Galnc1	Galnc2
13	AGC1	YPR021C	YPDcl2	YPDcl3	EtOHcl1	EtOHcl2	Galcl1	YPDnc1	YPDnc2	YPDnc3	YPDnc4	EtOHnc1	EtOHnc2	Galnc1	Galnc2
13	GND2	YGR256W	YPDcl2	YPDcl3	EtOHcl1	EtOHcl2	Galcl1	YPDnc1	YPDnc2	YPDnc3	YPDnc4	EtOHnc1	EtOHnc2	Galnc1	Galnc2
13	PCK1	YKR097W	YPDcl2	YPDcl3	EtOHcl1	EtOHcl2	Galcl1	YPDnc1	YPDnc2	YPDnc3	YPDnc4	EtOHnc1	EtOHnc2	Galnc1	Galnc2
13		YOL038C-A	YPDcl2	YPDcl3	EtOHcl1	EtOHcl2	Galcl1	YPDnc1	YPDnc2	YPDnc3	YPDnc4	EtOHnc1	EtOHnc2	Galnc1	Galnc2
13	YHP1	YDR451C	YPDcl2	YPDcl3	EtOHcl1	EtOHcl2	Galcl1	YPDnc1	YPDnc2	YPDnc3	YPDnc4	EtOHnc1	EtOHnc2	Galnc1	Galnc2
13	VAC17	YCL063W	YPDcl2	YPDcl3	EtOHcl1	EtOHcl2	Galcl1	YPDnc1	YPDnc2	YPDnc3	YPDnc4	EtOHnc1	EtOHnc2	Galnc1	Galnc2
13		YHR177W	YPDcl2	YPDcl3	EtOHcl1	EtOHcl2	Galcl1	YPDnc1	YPDnc2	YPDnc3	YPDnc4	EtOHnc1	EtOHnc2	Galnc1	Galnc2
13	ATP3	YBR039W	YPDcl2	YPDcl3	EtOHcl1	EtOHcl2	Galcl1	YPDnc1	YPDnc2	YPDnc3	YPDnc4	EtOHnc1	EtOHnc2	Galnc1	Galnc2
13	DAL1	YIR027C	YPDcl2	YPDcl3	EtOHcl1	EtOHcl2	Galcl1	YPDnc1	YPDnc2	YPDnc3	YPDnc4	EtOHnc1	EtOHnc2	Galnc1	Galnc2
13		YGL214W	YPDcl2	YPDcl3	EtOHcl1	EtOHcl2	Galcl1	YPDnc1	YPDnc2	YPDnc3	YPDnc4	EtOHnc1	EtOHnc2	Galnc1	Galnc2
13		YER023C-A	YPDcl2	YPDcl3	EtOHcl1	EtOHcl2	Galcl1	YPDnc1	YPDnc2	YPDnc3	YPDnc4	EtOHnc1	EtOHnc2	Galnc1	Galnc2
13	ERV46	YAL042W	YPDcl2	YPDcl3	EtOHcl1	EtOHcl2	Galcl1	YPDnc1	YPDnc2	YPDnc3	YPDnc4	EtOHnc1	EtOHnc2	Galnc1	Galnc2
13	NAS2	YIL007C	YPDcl2	YPDcl3	EtOHcl1	EtOHcl2	Galcl1	YPDnc1	YPDnc2	YPDnc3	YPDnc4	EtOHnc1	EtOHnc2	Galnc1	Galnc2
13	SVP26	YHR181W	YPDcl2	YPDcl3	EtOHcl1	EtOHcl2	Galcl1	YPDnc1	YPDnc2	YPDnc3	YPDnc4	EtOHnc1	EtOHnc2	Galnc1	Galnc2
13	ZRC1	YMR243C	YPDcl2	YPDcl3	EtOHcl1	EtOHcl2	Galcl1	YPDnc1	YPDnc2	YPDnc3	YPDnc4	EtOHnc1	EtOHnc2	Galnc1	Galnc2
13	CCT6	YDR188W	YPDcl2	YPDcl3	EtOHcl1	EtOHcl2	Galcl1	YPDnc1	YPDnc2	YPDnc3	YPDnc4	EtOHnc1	EtOHnc2	Galnc1	Galnc2

13		YNR034W-.	YPDcl2	YPDcl3	EtOHcl1	EtOHcl2	Galcl1	YPDnc1	YPDnc2	YPDnc3	YPDnc4	EtOHnc1	EtOHnc2	Galnc1	Galnc2
13		YEL023C	YPDcl2	YPDcl3	EtOHcl1	EtOHcl2	Galcl1	YPDnc1	YPDnc2	YPDnc3	YPDnc4	EtOHnc1	EtOHnc2	Galnc1	Galnc2
13		YNL057W	YPDcl2	YPDcl3	EtOHcl1	EtOHcl2	Galcl1	YPDnc1	YPDnc2	YPDnc3	YPDnc4	EtOHnc1	EtOHnc2	Galnc1	Galnc2
13	KAR3	YPR141C	YPDcl2	YPDcl3	EtOHcl1	EtOHcl2	Galcl1	YPDnc1	YPDnc2	YPDnc3	YPDnc4	EtOHnc1	EtOHnc2	Galnc1	Galnc2
13		YJR061W	YPDcl2	YPDcl3	EtOHcl1	EtOHcl2	Galcl1	YPDnc1	YPDnc2	YPDnc3	YPDnc4	EtOHnc1	EtOHnc2	Galnc1	Galnc2
13	GRC3	YLL035W	YPDcl2	YPDcl3	EtOHcl1	EtOHcl2	Galcl1	YPDnc1	YPDnc2	YPDnc3	YPDnc4	EtOHnc1	EtOHnc2	Galnc1	Galnc2
13	SPP382	YLR424W	YPDcl2	YPDcl3	EtOHcl1	EtOHcl2	Galcl1	YPDnc1	YPDnc2	YPDnc3	YPDnc4	EtOHnc1	EtOHnc2	Galnc1	Galnc2
13	ALG7	YBR243C	YPDcl2	YPDcl3	EtOHcl1	EtOHcl2	Galcl1	YPDnc1	YPDnc2	YPDnc3	YPDnc4	EtOHnc1	EtOHnc2	Galnc1	Galnc2
13	VID27	YNL212W	YPDcl2	YPDcl3	EtOHcl1	EtOHcl2	Galcl1	YPDnc1	YPDnc2	YPDnc3	YPDnc4	EtOHnc1	EtOHnc2	Galnc1	Galnc2
13	RNH1	YMR234W	YPDcl2	YPDcl3	EtOHcl1	EtOHcl2	Galcl1	YPDnc1	YPDnc2	YPDnc3	YPDnc4	EtOHnc1	EtOHnc2	Galnc1	Galnc2
13		YJL026C-A	YPDcl2	YPDcl3	EtOHcl1	EtOHcl2	Galcl1	YPDnc1	YPDnc2	YPDnc3	YPDnc4	EtOHnc1	EtOHnc2	Galnc1	Galnc2
13	MED11	YMR112C	YPDcl2	YPDcl3	EtOHcl1	EtOHcl2	Galcl1	YPDnc1	YPDnc2	YPDnc3	YPDnc4	EtOHnc1	EtOHnc2	Galnc1	Galnc2
13	LCB3	YJL134W	YPDcl2	YPDcl3	EtOHcl1	EtOHcl2	Galcl1	YPDnc1	YPDnc2	YPDnc3	YPDnc4	EtOHnc1	EtOHnc2	Galnc1	Galnc2
13		YOR105W	YPDcl2	YPDcl3	EtOHcl1	EtOHcl2	Galcl1	YPDnc1	YPDnc2	YPDnc3	YPDnc4	EtOHnc1	EtOHnc2	Galnc1	Galnc2
13		YJL127C-B	YPDcl2	YPDcl3	EtOHcl1	EtOHcl2	Galcl1	YPDnc1	YPDnc2	YPDnc3	YPDnc4	EtOHnc1	EtOHnc2	Galnc1	Galnc2
13	SNF12	YNR023W	YPDcl2	YPDcl3	EtOHcl1	EtOHcl2	Galcl1	YPDnc1	YPDnc2	YPDnc3	YPDnc4	EtOHnc1	EtOHnc2	Galnc1	Galnc2
13	VPS41	YDR080W	YPDcl2	YPDcl3	EtOHcl1	EtOHcl2	Galcl1	YPDnc1	YPDnc2	YPDnc3	YPDnc4	EtOHnc1	EtOHnc2	Galnc1	Galnc2
13		YHR073C-B	YPDcl2	YPDcl3	EtOHcl1	EtOHcl2	Galcl1	YPDnc1	YPDnc2	YPDnc3	YPDnc4	EtOHnc1	EtOHnc2	Galnc1	Galnc2
13		YBR096W	YPDcl2	YPDcl3	EtOHcl1	EtOHcl2	Galcl1	YPDnc1	YPDnc2	YPDnc3	YPDnc4	EtOHnc1	EtOHnc2	Galnc1	Galnc2
13	NUP85	YJR042W	YPDcl2	YPDcl3	EtOHcl1	EtOHcl2	Galcl1	YPDnc1	YPDnc2	YPDnc3	YPDnc4	EtOHnc1	EtOHnc2	Galnc1	Galnc2
13	SMD1	YGR074W	YPDcl2	YPDcl3	EtOHcl1	EtOHcl2	Galcl1	YPDnc1	YPDnc2	YPDnc3	YPDnc4	EtOHnc1	EtOHnc2	Galnc1	Galnc2
13		YDR089W	YPDcl2	YPDcl3	EtOHcl1	EtOHcl2	Galcl1	YPDnc1	YPDnc2	YPDnc3	YPDnc4	EtOHnc1	EtOHnc2	Galnc1	Galnc2
13	ATG1	YGL180W	YPDcl2	YPDcl3	EtOHcl1	EtOHcl2	Galcl1	YPDnc1	YPDnc2	YPDnc3	YPDnc4	EtOHnc1	EtOHnc2	Galnc1	Galnc2
13	MRPS9	YBR146W	YPDcl2	YPDcl3	EtOHcl1	EtOHcl2	Galcl1	YPDnc1	YPDnc2	YPDnc3	YPDnc4	EtOHnc1	EtOHnc2	Galnc1	Galnc2
13	KCS1	YDR017C	YPDcl2	YPDcl3	EtOHcl1	EtOHcl2	Galcl1	YPDnc1	YPDnc2	YPDnc3	YPDnc4	EtOHnc1	EtOHnc2	Galnc1	Galnc2
13	AFR1	YDR085C	YPDcl2	YPDcl3	EtOHcl1	EtOHcl2	Galcl1	YPDnc1	YPDnc2	YPDnc3	YPDnc4	EtOHnc1	EtOHnc2	Galnc1	Galnc2
13		YKL053W	YPDcl2	YPDcl3	EtOHcl1	EtOHcl2	Galcl1	YPDnc1	YPDnc2	YPDnc3	YPDnc4	EtOHnc1	EtOHnc2	Galnc1	Galnc2
13	DIA4	YHR011W	YPDcl2	YPDcl3	EtOHcl1	EtOHcl2	Galcl1	YPDnc1	YPDnc2	YPDnc3	YPDnc4	EtOHnc1	EtOHnc2	Galnc1	Galnc2
13	YPD1	YDL235C	YPDcl2	YPDcl3	EtOHcl1	EtOHcl2	Galcl1	YPDnc1	YPDnc2	YPDnc3	YPDnc4	EtOHnc1	EtOHnc2	Galnc1	Galnc2
13	FRT2	YAL028W	YPDcl2	YPDcl3	EtOHcl1	EtOHcl2	Galcl1	YPDnc1	YPDnc2	YPDnc3	YPDnc4	EtOHnc1	EtOHnc2	Galnc1	Galnc2
13	NHP10	YDL002C	YPDcl2	YPDcl3	EtOHcl1	EtOHcl2	Galcl1	YPDnc1	YPDnc2	YPDnc3	YPDnc4	EtOHnc1	EtOHnc2	Galnc1	Galnc2
13		YJL213W	YPDcl2	YPDcl3	EtOHcl1	EtOHcl2	Galcl1	YPDnc1	YPDnc2	YPDnc3	YPDnc4	EtOHnc1	EtOHnc2	Galnc1	Galnc2
13	MDG1	YNL173C	YPDcl2	YPDcl3	EtOHcl1	EtOHcl2	Galcl1	YPDnc1	YPDnc2	YPDnc3	YPDnc4	EtOHnc1	EtOHnc2	Galnc1	Galnc2
13	SKY1	YMR216C	YPDcl2	YPDcl3	EtOHcl1	EtOHcl2	Galcl1	YPDnc1	YPDnc2	YPDnc3	YPDnc4	EtOHnc1	EtOHnc2	Galnc1	Galnc2

13	SPH1	YLR313C	YPDcl2	YPDcl3	EtOHcl1	EtOHcl2	Galcl1	YPDnc1	YPDnc2	YPDnc3	YPDnc4	EtOHnc1	EtOHnc2	Galnc1	Galnc2
13		YER039C-A	YPDcl2	YPDcl3	EtOHcl1	EtOHcl2	Galcl1	YPDnc1	YPDnc2	YPDnc3	YPDnc4	EtOHnc1	EtOHnc2	Galnc1	Galnc2
13		YJL009W	YPDcl2	YPDcl3	EtOHcl1	EtOHcl2	Galcl1	YPDnc1	YPDnc2	YPDnc3	YPDnc4	EtOHnc1	EtOHnc2	Galnc1	Galnc2
13		YBR085C-A	YPDcl2	YPDcl3	EtOHcl1	EtOHcl2	Galcl1	YPDnc1	YPDnc2	YPDnc3	YPDnc4	EtOHnc1	EtOHnc2	Galnc1	Galnc2
13	RAD59	YDL059C	YPDcl2	YPDcl3	EtOHcl1	EtOHcl2	Galcl1	YPDnc1	YPDnc2	YPDnc3	YPDnc4	EtOHnc1	EtOHnc2	Galnc1	Galnc2
13	TDA4	YJR116W	YPDcl2	YPDcl3	EtOHcl1	EtOHcl2	Galcl1	YPDnc1	YPDnc2	YPDnc3	YPDnc4	EtOHnc1	EtOHnc2	Galnc1	Galnc2
13	RDH54	YBR073W	YPDcl2	YPDcl3	EtOHcl1	EtOHcl2	Galcl1	YPDnc1	YPDnc2	YPDnc3	YPDnc4	EtOHnc1	EtOHnc2	Galnc1	Galnc2
13		YPR064W	YPDcl2	YPDcl3	EtOHcl1	EtOHcl2	Galcl1	YPDnc1	YPDnc2	YPDnc3	YPDnc4	EtOHnc1	EtOHnc2	Galnc1	Galnc2
13	ASM4	YDL088C	YPDcl2	YPDcl3	EtOHcl1	EtOHcl2	Galcl1	YPDnc1	YPDnc2	YPDnc3	YPDnc4	EtOHnc1	EtOHnc2	Galnc1	Galnc2
13	CBK1	YNL161W	YPDcl2	YPDcl3	EtOHcl1	EtOHcl2	Galcl1	YPDnc1	YPDnc2	YPDnc3	YPDnc4	EtOHnc1	EtOHnc2	Galnc1	Galnc2
13	PPM1	YDR435C	YPDcl2	YPDcl3	EtOHcl1	EtOHcl2	Galcl1	YPDnc1	YPDnc2	YPDnc3	YPDnc4	EtOHnc1	EtOHnc2	Galnc1	Galnc2
13	JIP3	YLR331C	YPDcl2	YPDcl3	EtOHcl1	EtOHcl2	Galcl1	YPDnc1	YPDnc2	YPDnc3	YPDnc4	EtOHnc1	EtOHnc2	Galnc1	Galnc2
13	RPL19A	YBR084C-A	YPDcl2	YPDcl3	EtOHcl1	EtOHcl2	Galcl1	YPDnc1	YPDnc2	YPDnc3	YPDnc4	EtOHnc1	EtOHnc2	Galnc1	Galnc2
13		YDR161W	YPDcl2	YPDcl3	EtOHcl1	EtOHcl2	Galcl1	YPDnc1	YPDnc2	YPDnc3	YPDnc4	EtOHnc1	EtOHnc2	Galnc1	Galnc2
13		YOR285W	YPDcl2	YPDcl3	EtOHcl1	EtOHcl2	Galcl1	YPDnc1	YPDnc2	YPDnc3	YPDnc4	EtOHnc1	EtOHnc2	Galnc1	Galnc2
13		YLR253W	YPDcl2	YPDcl3	EtOHcl1	EtOHcl2	Galcl1	YPDnc1	YPDnc2	YPDnc3	YPDnc4	EtOHnc1	EtOHnc2	Galnc1	Galnc2
13	PRE3	YJL001W	YPDcl2	YPDcl3	EtOHcl1	EtOHcl2	Galcl1	YPDnc1	YPDnc2	YPDnc3	YPDnc4	EtOHnc1	EtOHnc2	Galnc1	Galnc2
13	HIF1	YLL022C	YPDcl2	YPDcl3	EtOHcl1	EtOHcl2	Galcl1	YPDnc1	YPDnc2	YPDnc3	YPDnc4	EtOHnc1	EtOHnc2	Galnc1	Galnc2
13	HTD2	YHR067W	YPDcl2	YPDcl3	EtOHcl1	EtOHcl2	Galcl1	YPDnc1	YPDnc2	YPDnc3	YPDnc4	EtOHnc1	EtOHnc2	Galnc1	Galnc2
13	HAP2	YGL237C	YPDcl2	YPDcl3	EtOHcl1	EtOHcl2	Galcl1	YPDnc1	YPDnc2	YPDnc3	YPDnc4	EtOHnc1	EtOHnc2	Galnc1	Galnc2
13	TCM62	YBR044C	YPDcl2	YPDcl3	EtOHcl1	EtOHcl2	Galcl1	YPDnc1	YPDnc2	YPDnc3	YPDnc4	EtOHnc1	EtOHnc2	Galnc1	Galnc2
13	RGR1	YLR071C	YPDcl2	YPDcl3	EtOHcl1	EtOHcl2	Galcl1	YPDnc1	YPDnc2	YPDnc3	YPDnc4	EtOHnc1	EtOHnc2	Galnc1	Galnc2
13	TRM1	YDR120C	YPDcl2	YPDcl3	EtOHcl1	EtOHcl2	Galcl1	YPDnc1	YPDnc2	YPDnc3	YPDnc4	EtOHnc1	EtOHnc2	Galnc1	Galnc2
13	NSA2	YER126C	YPDcl2	YPDcl3	EtOHcl1	EtOHcl2	Galcl1	YPDnc1	YPDnc2	YPDnc3	YPDnc4	EtOHnc1	EtOHnc2	Galnc1	Galnc2
13	PHO85	YPL031C	YPDcl2	YPDcl3	EtOHcl1	EtOHcl2	Galcl1	YPDnc1	YPDnc2	YPDnc3	YPDnc4	EtOHnc1	EtOHnc2	Galnc1	Galnc2
13	SAP185	YJL098W	YPDcl2	YPDcl3	EtOHcl1	EtOHcl2	Galcl1	YPDnc1	YPDnc2	YPDnc3	YPDnc4	EtOHnc1	EtOHnc2	Galnc1	Galnc2
13		YDR445C	YPDcl2	YPDcl3	EtOHcl1	EtOHcl2	Galcl1	YPDnc1	YPDnc2	YPDnc3	YPDnc4	EtOHnc1	EtOHnc2	Galnc1	Galnc2
13	AKL1	YBR059C	YPDcl2	YPDcl3	EtOHcl1	EtOHcl2	Galcl1	YPDnc1	YPDnc2	YPDnc3	YPDnc4	EtOHnc1	EtOHnc2	Galnc1	Galnc2
13	VMA7	YGR020C	YPDcl2	YPDcl3	EtOHcl1	EtOHcl2	Galcl1	YPDnc1	YPDnc2	YPDnc3	YPDnc4	EtOHnc1	EtOHnc2	Galnc1	Galnc2
13	SMA2	YML066C	YPDcl2	YPDcl3	EtOHcl1	EtOHcl2	Galcl1	YPDnc1	YPDnc2	YPDnc3	YPDnc4	EtOHnc1	EtOHnc2	Galnc1	Galnc2
13	PLM2	YDR501W	YPDcl2	YPDcl3	EtOHcl1	EtOHcl2	Galcl1	YPDnc1	YPDnc2	YPDnc3	YPDnc4	EtOHnc1	EtOHnc2	Galnc1	Galnc2
13	TOP2	YNL088W	YPDcl2	YPDcl3	EtOHcl1	EtOHcl2	Galcl1	YPDnc1	YPDnc2	YPDnc3	YPDnc4	EtOHnc1	EtOHnc2	Galnc1	Galnc2
13	MSG5	YNL053W	YPDcl2	YPDcl3	EtOHcl1	EtOHcl2	Galcl1	YPDnc1	YPDnc2	YPDnc3	YPDnc4	EtOHnc1	EtOHnc2	Galnc1	Galnc2
13		YKR043C	YPDcl2	YPDcl3	EtOHcl1	EtOHcl2	Galcl1	YPDnc1	YPDnc2	YPDnc3	YPDnc4	EtOHnc1	EtOHnc2	Galnc1	Galnc2

13	ECM34	YHL043W	YPDcl2	YPDcl3	EtOHcl1	EtOHcl2	Galcl1	YPDnc1	YPDnc2	YPDnc3	YPDnc4	EtOHnc1	EtOHnc2	Galnc1	Galnc2
13	UTP7	YER082C	YPDcl2	YPDcl3	EtOHcl1	EtOHcl2	Galcl1	YPDnc1	YPDnc2	YPDnc3	YPDnc4	EtOHnc1	EtOHnc2	Galnc1	Galnc2
13	ATG18	YFR021W	YPDcl2	YPDcl3	EtOHcl1	EtOHcl2	Galcl1	YPDnc1	YPDnc2	YPDnc3	YPDnc4	EtOHnc1	EtOHnc2	Galnc1	Galnc2
13	GTR1	YML121W	YPDcl2	YPDcl3	EtOHcl1	EtOHcl2	Galcl1	YPDnc1	YPDnc2	YPDnc3	YPDnc4	EtOHnc1	EtOHnc2	Galnc1	Galnc2
13	LSC2	YGR244C	YPDcl2	YPDcl3	EtOHcl1	EtOHcl2	Galcl1	YPDnc1	YPDnc2	YPDnc3	YPDnc4	EtOHnc1	EtOHnc2	Galnc1	Galnc2
13	ZRT1	YGL255W	YPDcl2	YPDcl3	EtOHcl1	EtOHcl2	Galcl1	YPDnc1	YPDnc2	YPDnc3	YPDnc4	EtOHnc1	EtOHnc2	Galnc1	Galnc2
13	NCE103	YNL036W	YPDcl2	YPDcl3	EtOHcl1	EtOHcl2	Galcl1	YPDnc1	YPDnc2	YPDnc3	YPDnc4	EtOHnc1	EtOHnc2	Galnc1	Galnc2
13	INM1	YHR046C	YPDcl2	YPDcl3	EtOHcl1	EtOHcl2	Galcl1	YPDnc1	YPDnc2	YPDnc3	YPDnc4	EtOHnc1	EtOHnc2	Galnc1	Galnc2
13	ERP4	YOR016C	YPDcl2	YPDcl3	EtOHcl1	EtOHcl2	Galcl1	YPDnc1	YPDnc2	YPDnc3	YPDnc4	EtOHnc1	EtOHnc2	Galnc1	Galnc2
13	DRE2	YKR071C	YPDcl2	YPDcl3	EtOHcl1	EtOHcl2	Galcl1	YPDnc1	YPDnc2	YPDnc3	YPDnc4	EtOHnc1	EtOHnc2	Galnc1	Galnc2
13	SPT4	YGR063C	YPDcl2	YPDcl3	EtOHcl1	EtOHcl2	Galcl1	YPDnc1	YPDnc2	YPDnc3	YPDnc4	EtOHnc1	EtOHnc2	Galnc1	Galnc2
13	ORC1	YML065W	YPDcl2	YPDcl3	EtOHcl1	EtOHcl2	Galcl1	YPDnc1	YPDnc2	YPDnc3	YPDnc4	EtOHnc1	EtOHnc2	Galnc1	Galnc2
13	HAL1	YPR005C	YPDcl2	YPDcl3	EtOHcl1	EtOHcl2	Galcl1	YPDnc1	YPDnc2	YPDnc3	YPDnc4	EtOHnc1	EtOHnc2	Galnc1	Galnc2
13	HSV2	YGR223C	YPDcl2	YPDcl3	EtOHcl1	EtOHcl2	Galcl1	YPDnc1	YPDnc2	YPDnc3	YPDnc4	EtOHnc1	EtOHnc2	Galnc1	Galnc2
13	SWD1	YAR003W	YPDcl2	YPDcl3	EtOHcl1	EtOHcl2	Galcl1	YPDnc1	YPDnc2	YPDnc3	YPDnc4	EtOHnc1	EtOHnc2	Galnc1	Galnc2
13	PPA2	YMR267W	YPDcl2	YPDcl3	EtOHcl1	EtOHcl2	Galcl1	YPDnc1	YPDnc2	YPDnc3	YPDnc4	EtOHnc1	EtOHnc2	Galnc1	Galnc2
13	NOG1	YPL093W	YPDcl2	YPDcl3	EtOHcl1	EtOHcl2	Galcl1	YPDnc1	YPDnc2	YPDnc3	YPDnc4	EtOHnc1	EtOHnc2	Galnc1	Galnc2
13	APJ1	YNL077W	YPDcl2	YPDcl3	EtOHcl1	EtOHcl2	Galcl1	YPDnc1	YPDnc2	YPDnc3	YPDnc4	EtOHnc1	EtOHnc2	Galnc1	Galnc2
13	PET191	YJR034W	YPDcl2	YPDcl3	EtOHcl1	EtOHcl2	Galcl1	YPDnc1	YPDnc2	YPDnc3	YPDnc4	EtOHnc1	EtOHnc2	Galnc1	Galnc2
13	CDC60	YPL160W	YPDcl2	YPDcl3	EtOHcl1	EtOHcl2	Galcl1	YPDnc1	YPDnc2	YPDnc3	YPDnc4	EtOHnc1	EtOHnc2	Galnc1	Galnc2
13		YGL185C	YPDcl2	YPDcl3	EtOHcl1	EtOHcl2	Galcl1	YPDnc1	YPDnc2	YPDnc3	YPDnc4	EtOHnc1	EtOHnc2	Galnc1	Galnc2
13	ZRT3	YKL175W	YPDcl2	YPDcl3	EtOHcl1	EtOHcl2	Galcl1	YPDnc1	YPDnc2	YPDnc3	YPDnc4	EtOHnc1	EtOHnc2	Galnc1	Galnc2
13	NHX1	YDR456W	YPDcl2	YPDcl3	EtOHcl1	EtOHcl2	Galcl1	YPDnc1	YPDnc2	YPDnc3	YPDnc4	EtOHnc1	EtOHnc2	Galnc1	Galnc2
13	COG7	YGL005C	YPDcl2	YPDcl3	EtOHcl1	EtOHcl2	Galcl1	YPDnc1	YPDnc2	YPDnc3	YPDnc4	EtOHnc1	EtOHnc2	Galnc1	Galnc2
13	ESBP6	YNL125C	YPDcl2	YPDcl3	EtOHcl1	EtOHcl2	Galcl1	YPDnc1	YPDnc2	YPDnc3	YPDnc4	EtOHnc1	EtOHnc2	Galnc1	Galnc2
13	QDR3	YBR043C	YPDcl2	YPDcl3	EtOHcl1	EtOHcl2	Galcl1	YPDnc1	YPDnc2	YPDnc3	YPDnc4	EtOHnc1	EtOHnc2	Galnc1	Galnc2
13	BRE4	YDL231C	YPDcl2	YPDcl3	EtOHcl1	EtOHcl2	Galcl1	YPDnc1	YPDnc2	YPDnc3	YPDnc4	EtOHnc1	EtOHnc2	Galnc1	Galnc2
13	RFC4	YOL094C	YPDcl2	YPDcl3	EtOHcl1	EtOHcl2	Galcl1	YPDnc1	YPDnc2	YPDnc3	YPDnc4	EtOHnc1	EtOHnc2	Galnc1	Galnc2
13	VPS20	YMR077C	YPDcl2	YPDcl3	EtOHcl1	EtOHcl2	Galcl1	YPDnc1	YPDnc2	YPDnc3	YPDnc4	EtOHnc1	EtOHnc2	Galnc1	Galnc2
13	COY1	YKL179C	YPDcl2	YPDcl3	EtOHcl1	EtOHcl2	Galcl1	YPDnc1	YPDnc2	YPDnc3	YPDnc4	EtOHnc1	EtOHnc2	Galnc1	Galnc2
13	PRP19	YLL036C	YPDcl2	YPDcl3	EtOHcl1	EtOHcl2	Galcl1	YPDnc1	YPDnc2	YPDnc3	YPDnc4	EtOHnc1	EtOHnc2	Galnc1	Galnc2
13	BAS1	YKR099W	YPDcl2	YPDcl3	EtOHcl1	EtOHcl2	Galcl1	YPDnc1	YPDnc2	YPDnc3	YPDnc4	EtOHnc1	EtOHnc2	Galnc1	Galnc2
13	MED2	YDL005C	YPDcl2	YPDcl3	EtOHcl1	EtOHcl2	Galcl1	YPDnc1	YPDnc2	YPDnc3	YPDnc4	EtOHnc1	EtOHnc2	Galnc1	Galnc2
13		YOR146W	YPDcl2	YPDcl3	EtOHcl1	EtOHcl2	Galcl1	YPDnc1	YPDnc2	YPDnc3	YPDnc4	EtOHnc1	EtOHnc2	Galnc1	Galnc2

13	MBP1	YDL056W	YPDcl2	YPDcl3	EtOHcl1	EtOHcl2	Galcl1	YPDnc1	YPDnc2	YPDnc3	YPDnc4	EtOHnc1	EtOHnc2	Galnc1	Galnc2
13	NDI1	YML120C	YPDcl2	YPDcl3	EtOHcl1	EtOHcl2	Galcl1	YPDnc1	YPDnc2	YPDnc3	YPDnc4	EtOHnc1	EtOHnc2	Galnc1	Galnc2
13	FRE5	YOR384W	YPDcl2	YPDcl3	EtOHcl1	EtOHcl2	Galcl1	YPDnc1	YPDnc2	YPDnc3	YPDnc4	EtOHnc1	EtOHnc2	Galnc1	Galnc2
13	DCP1	YOL149W	YPDcl2	YPDcl3	EtOHcl1	EtOHcl2	Galcl1	YPDnc1	YPDnc2	YPDnc3	YPDnc4	EtOHnc1	EtOHnc2	Galnc1	Galnc2
13	COX14	YML129C	YPDcl2	YPDcl3	EtOHcl1	EtOHcl2	Galcl1	YPDnc1	YPDnc2	YPDnc3	YPDnc4	EtOHnc1	EtOHnc2	Galnc1	Galnc2
13		YIL059C	YPDcl2	YPDcl3	EtOHcl1	EtOHcl2	Galcl1	YPDnc1	YPDnc2	YPDnc3	YPDnc4	EtOHnc1	EtOHnc2	Galnc1	Galnc2
13		YBR220C	YPDcl2	YPDcl3	EtOHcl1	EtOHcl2	Galcl1	YPDnc1	YPDnc2	YPDnc3	YPDnc4	EtOHnc1	EtOHnc2	Galnc1	Galnc2
13	ZPS1	YOL154W	YPDcl2	YPDcl3	EtOHcl1	EtOHcl2	Galcl1	YPDnc1	YPDnc2	YPDnc3	YPDnc4	EtOHnc1	EtOHnc2	Galnc1	Galnc2
13	BIO5	YNR056C	YPDcl2	YPDcl3	EtOHcl1	EtOHcl2	Galcl1	YPDnc1	YPDnc2	YPDnc3	YPDnc4	EtOHnc1	EtOHnc2	Galnc1	Galnc2
13	EGT2	YNL327W	YPDcl2	YPDcl3	EtOHcl1	EtOHcl2	Galcl1	YPDnc1	YPDnc2	YPDnc3	YPDnc4	EtOHnc1	EtOHnc2	Galnc1	Galnc2
13	ORT1	YOR130C	YPDcl2	YPDcl3	EtOHcl1	EtOHcl2	Galcl1	YPDnc1	YPDnc2	YPDnc3	YPDnc4	EtOHnc1	EtOHnc2	Galnc1	Galnc2
13		YJL181W	YPDcl2	YPDcl3	EtOHcl1	EtOHcl2	Galcl1	YPDnc1	YPDnc2	YPDnc3	YPDnc4	EtOHnc1	EtOHnc2	Galnc1	Galnc2
13	AIM43	YPL099C	YPDcl2	YPDcl3	EtOHcl1	EtOHcl2	Galcl1	YPDnc1	YPDnc2	YPDnc3	YPDnc4	EtOHnc1	EtOHnc2	Galnc1	Galnc2
13	ATG2	YNL242W	YPDcl2	YPDcl3	EtOHcl1	EtOHcl2	Galcl1	YPDnc1	YPDnc2	YPDnc3	YPDnc4	EtOHnc1	EtOHnc2	Galnc1	Galnc2
13	MON2	YNL297C	YPDcl2	YPDcl3	EtOHcl1	EtOHcl2	Galcl1	YPDnc1	YPDnc2	YPDnc3	YPDnc4	EtOHnc1	EtOHnc2	Galnc1	Galnc2
13	WBP1	YEL002C	YPDcl2	YPDcl3	EtOHcl1	EtOHcl2	Galcl1	YPDnc1	YPDnc2	YPDnc3	YPDnc4	EtOHnc1	EtOHnc2	Galnc1	Galnc2
13	SSY5	YJL156C	YPDcl2	YPDcl3	EtOHcl1	EtOHcl2	Galcl1	YPDnc1	YPDnc2	YPDnc3	YPDnc4	EtOHnc1	EtOHnc2	Galnc1	Galnc2
13		YMR166C	YPDcl2	YPDcl3	EtOHcl1	EtOHcl2	Galcl1	YPDnc1	YPDnc2	YPDnc3	YPDnc4	EtOHnc1	EtOHnc2	Galnc1	Galnc2
13	STE50	YCL032W	YPDcl2	YPDcl3	EtOHcl1	EtOHcl2	Galcl1	YPDnc1	YPDnc2	YPDnc3	YPDnc4	EtOHnc1	EtOHnc2	Galnc1	Galnc2
13		YDR034C-A	YPDcl2	YPDcl3	EtOHcl1	EtOHcl2	Galcl1	YPDnc1	YPDnc2	YPDnc3	YPDnc4	EtOHnc1	EtOHnc2	Galnc1	Galnc2
13	MNN9	YPL050C	YPDcl2	YPDcl3	EtOHcl1	EtOHcl2	Galcl1	YPDnc1	YPDnc2	YPDnc3	YPDnc4	EtOHnc1	EtOHnc2	Galnc1	Galnc2
13	SMT3	YDR510W	YPDcl2	YPDcl3	EtOHcl1	EtOHcl2	Galcl1	YPDnc1	YPDnc2	YPDnc3	YPDnc4	EtOHnc1	EtOHnc2	Galnc1	Galnc2
13		YER158W-A	YPDcl2	YPDcl3	EtOHcl1	EtOHcl2	Galcl1	YPDnc1	YPDnc2	YPDnc3	YPDnc4	EtOHnc1	EtOHnc2	Galnc1	Galnc2
13		YPL277C	YPDcl2	YPDcl3	EtOHcl1	EtOHcl2	Galcl1	YPDnc1	YPDnc2	YPDnc3	YPDnc4	EtOHnc1	EtOHnc2	Galnc1	Galnc2
13		YBR116C	YPDcl2	YPDcl3	EtOHcl1	EtOHcl2	Galcl1	YPDnc1	YPDnc2	YPDnc3	YPDnc4	EtOHnc1	EtOHnc2	Galnc1	Galnc2
13		YPL257W	YPDcl2	YPDcl3	EtOHcl1	EtOHcl2	Galcl1	YPDnc1	YPDnc2	YPDnc3	YPDnc4	EtOHnc1	EtOHnc2	Galnc1	Galnc2
13	PUT1	YLR142W	YPDcl2	YPDcl3	EtOHcl1	EtOHcl2	Galcl1	YPDnc1	YPDnc2	YPDnc3	YPDnc4	EtOHnc1	EtOHnc2	Galnc1	Galnc2
13	SSU72	YNL222W	YPDcl2	YPDcl3	EtOHcl1	EtOHcl2	Galcl1	YPDnc1	YPDnc2	YPDnc3	YPDnc4	EtOHnc1	EtOHnc2	Galnc1	Galnc2
13	GCD7	YLR291C	YPDcl2	YPDcl3	EtOHcl1	EtOHcl2	Galcl1	YPDnc1	YPDnc2	YPDnc3	YPDnc4	EtOHnc1	EtOHnc2	Galnc1	Galnc2
13		YKR051W	YPDcl2	YPDcl3	EtOHcl1	EtOHcl2	Galcl1	YPDnc1	YPDnc2	YPDnc3	YPDnc4	EtOHnc1	EtOHnc2	Galnc1	Galnc2
13	VBA2	YBR293W	YPDcl2	YPDcl3	EtOHcl1	EtOHcl2	Galcl1	YPDnc1	YPDnc2	YPDnc3	YPDnc4	EtOHnc1	EtOHnc2	Galnc1	Galnc2
13	SAP155	YFR040W	YPDcl2	YPDcl3	EtOHcl1	EtOHcl2	Galcl1	YPDnc1	YPDnc2	YPDnc3	YPDnc4	EtOHnc1	EtOHnc2	Galnc1	Galnc2
13	MLF3	YNL074C	YPDcl2	YPDcl3	EtOHcl1	EtOHcl2	Galcl1	YPDnc1	YPDnc2	YPDnc3	YPDnc4	EtOHnc1	EtOHnc2	Galnc1	Galnc2
13	UNG1	YML021C	YPDcl2	YPDcl3	EtOHcl1	EtOHcl2	Galcl1	YPDnc1	YPDnc2	YPDnc3	YPDnc4	EtOHnc1	EtOHnc2	Galnc1	Galnc2

13	FCY22	YER060W-	YPDcl2	YPDcl3	EtOHcl1	EtOHcl2	Galcl1	YPDnc1	YPDnc2	YPDnc3	YPDnc4	EtOHnc1	EtOHnc2	Galnc1	Galnc2
13		YDR203W	YPDcl2	YPDcl3	EtOHcl1	EtOHcl2	Galcl1	YPDnc1	YPDnc2	YPDnc3	YPDnc4	EtOHnc1	EtOHnc2	Galnc1	Galnc2
13	SPR1	YOR190W	YPDcl2	YPDcl3	EtOHcl1	EtOHcl2	Galcl1	YPDnc1	YPDnc2	YPDnc3	YPDnc4	EtOHnc1	EtOHnc2	Galnc1	Galnc2
13	SHE3	YBR130C	YPDcl2	YPDcl3	EtOHcl1	EtOHcl2	Galcl1	YPDnc1	YPDnc2	YPDnc3	YPDnc4	EtOHnc1	EtOHnc2	Galnc1	Galnc2
13	RPL12B	YDR418W	YPDcl2	YPDcl3	EtOHcl1	EtOHcl2	Galcl1	YPDnc1	YPDnc2	YPDnc3	YPDnc4	EtOHnc1	EtOHnc2	Galnc1	Galnc2
13		YGR270C-	YPDcl2	YPDcl3	EtOHcl1	EtOHcl2	Galcl1	YPDnc1	YPDnc2	YPDnc3	YPDnc4	EtOHnc1	EtOHnc2	Galnc1	Galnc2
13		YIR024C	YPDcl2	YPDcl3	EtOHcl1	EtOHcl2	Galcl1	YPDnc1	YPDnc2	YPDnc3	YPDnc4	EtOHnc1	EtOHnc2	Galnc1	Galnc2
13	HPF1	YOL155C	YPDcl2	YPDcl3	EtOHcl1	EtOHcl2	Galcl1	YPDnc1	YPDnc2	YPDnc3	YPDnc4	EtOHnc1	EtOHnc2	Galnc1	Galnc2
13	EST3	YIL009C-A	YPDcl2	YPDcl3	EtOHcl1	EtOHcl2	Galcl1	YPDnc1	YPDnc2	YPDnc3	YPDnc4	EtOHnc1	EtOHnc2	Galnc1	Galnc2
13	GEF1	YJR040W	YPDcl2	YPDcl3	EtOHcl1	EtOHcl2	Galcl1	YPDnc1	YPDnc2	YPDnc3	YPDnc4	EtOHnc1	EtOHnc2	Galnc1	Galnc2
13	IRC18	YJL037W	YPDcl2	YPDcl3	EtOHcl1	EtOHcl2	Galcl1	YPDnc1	YPDnc2	YPDnc3	YPDnc4	EtOHnc1	EtOHnc2	Galnc1	Galnc2
13		YOR059C	YPDcl2	YPDcl3	EtOHcl1	EtOHcl2	Galcl1	YPDnc1	YPDnc2	YPDnc3	YPDnc4	EtOHnc1	EtOHnc2	Galnc1	Galnc2
13		YGL149W	YPDcl2	YPDcl3	EtOHcl1	EtOHcl2	Galcl1	YPDnc1	YPDnc2	YPDnc3	YPDnc4	EtOHnc1	EtOHnc2	Galnc1	Galnc2
13	AIM20	YIL158W	YPDcl2	YPDcl3	EtOHcl1	EtOHcl2	Galcl1	YPDnc1	YPDnc2	YPDnc3	YPDnc4	EtOHnc1	EtOHnc2	Galnc1	Galnc2
13	IST2	YBR086C	YPDcl2	YPDcl3	EtOHcl1	EtOHcl2	Galcl1	YPDnc1	YPDnc2	YPDnc3	YPDnc4	EtOHnc1	EtOHnc2	Galnc1	Galnc2
13		YLR361C-A	YPDcl2	YPDcl3	EtOHcl1	EtOHcl2	Galcl1	YPDnc1	YPDnc2	YPDnc3	YPDnc4	EtOHnc1	EtOHnc2	Galnc1	Galnc2
13	ISD11	YER048W-	YPDcl2	YPDcl3	EtOHcl1	EtOHcl2	Galcl1	YPDnc1	YPDnc2	YPDnc3	YPDnc4	EtOHnc1	EtOHnc2	Galnc1	Galnc2
13		YML090W	YPDcl2	YPDcl3	EtOHcl1	EtOHcl2	Galcl1	YPDnc1	YPDnc2	YPDnc3	YPDnc4	EtOHnc1	EtOHnc2	Galnc1	Galnc2
13	DNA2	YHR164C	YPDcl2	YPDcl3	EtOHcl1	EtOHcl2	Galcl1	YPDnc1	YPDnc2	YPDnc3	YPDnc4	EtOHnc1	EtOHnc2	Galnc1	Galnc2
13	EAP1	YKL204W	YPDcl2	YPDcl3	EtOHcl1	EtOHcl2	Galcl1	YPDnc1	YPDnc2	YPDnc3	YPDnc4	EtOHnc1	EtOHnc2	Galnc1	Galnc2
13	MRPL28	YDR462W	YPDcl2	YPDcl3	EtOHcl1	EtOHcl2	Galcl1	YPDnc1	YPDnc2	YPDnc3	YPDnc4	EtOHnc1	EtOHnc2	Galnc1	Galnc2
13	ZDS1	YMR273C	YPDcl2	YPDcl3	EtOHcl1	EtOHcl2	Galcl1	YPDnc1	YPDnc2	YPDnc3	YPDnc4	EtOHnc1	EtOHnc2	Galnc1	Galnc2
13	JJJ2	YJL162C	YPDcl2	YPDcl3	EtOHcl1	EtOHcl2	Galcl1	YPDnc1	YPDnc2	YPDnc3	YPDnc4	EtOHnc1	EtOHnc2	Galnc1	Galnc2
13		YMR175W-	YPDcl2	YPDcl3	EtOHcl1	EtOHcl2	Galcl1	YPDnc1	YPDnc2	YPDnc3	YPDnc4	EtOHnc1	EtOHnc2	Galnc1	Galnc2
13		YLL054C	YPDcl2	YPDcl3	EtOHcl1	EtOHcl2	Galcl1	YPDnc1	YPDnc2	YPDnc3	YPDnc4	EtOHnc1	EtOHnc2	Galnc1	Galnc2
13	PSA1	YDL055C	YPDcl2	YPDcl3	EtOHcl1	EtOHcl2	Galcl1	YPDnc1	YPDnc2	YPDnc3	YPDnc4	EtOHnc1	EtOHnc2	Galnc1	Galnc2
13	TEF1	YPR080W	YPDcl2	YPDcl3	EtOHcl1	EtOHcl2	Galcl1	YPDnc1	YPDnc2	YPDnc3	YPDnc4	EtOHnc1	EtOHnc2	Galnc1	Galnc2
13		YOL019W	YPDcl2	YPDcl3	EtOHcl1	EtOHcl2	Galcl1	YPDnc1	YPDnc2	YPDnc3	YPDnc4	EtOHnc1	EtOHnc2	Galnc1	Galnc2
13	SOH1	YGL127C	YPDcl2	YPDcl3	EtOHcl1	EtOHcl2	Galcl1	YPDnc1	YPDnc2	YPDnc3	YPDnc4	EtOHnc1	EtOHnc2	Galnc1	Galnc2
13	PDR8	YLR266C	YPDcl2	YPDcl3	EtOHcl1	EtOHcl2	Galcl1	YPDnc1	YPDnc2	YPDnc3	YPDnc4	EtOHnc1	EtOHnc2	Galnc1	Galnc2
13	RPL8A	YHL033C	YPDcl2	YPDcl3	EtOHcl1	EtOHcl2	Galcl1	YPDnc1	YPDnc2	YPDnc3	YPDnc4	EtOHnc1	EtOHnc2	Galnc1	Galnc2
13	ECM4	YKR076W	YPDcl2	YPDcl3	EtOHcl1	EtOHcl2	Galcl1	YPDnc1	YPDnc2	YPDnc3	YPDnc4	EtOHnc1	EtOHnc2	Galnc1	Galnc2
13	NGL2	YMR285C	YPDcl2	YPDcl3	EtOHcl1	EtOHcl2	Galcl1	YPDnc1	YPDnc2	YPDnc3	YPDnc4	EtOHnc1	EtOHnc2	Galnc1	Galnc2
13	KEL3	YPL263C	YPDcl2	YPDcl3	EtOHcl1	EtOHcl2	Galcl1	YPDnc1	YPDnc2	YPDnc3	YPDnc4	EtOHnc1	EtOHnc2	Galnc1	Galnc2

13	SHC1	YER096W	YPDcl2	YPDcl3	EtOHcl1	EtOHcl2	Galcl1	YPDnc1	YPDnc2	YPDnc3	YPDnc4	EtOHnc1	EtOHnc2	Galnc1	Galnc2
13		YOR325W	YPDcl2	YPDcl3	EtOHcl1	EtOHcl2	Galcl1	YPDnc1	YPDnc2	YPDnc3	YPDnc4	EtOHnc1	EtOHnc2	Galnc1	Galnc2
13	MET18	YIL128W	YPDcl2	YPDcl3	EtOHcl1	EtOHcl2	Galcl1	YPDnc1	YPDnc2	YPDnc3	YPDnc4	EtOHnc1	EtOHnc2	Galnc1	Galnc2
13	AIM22	YJL046W	YPDcl2	YPDcl3	EtOHcl1	EtOHcl2	Galcl1	YPDnc1	YPDnc2	YPDnc3	YPDnc4	EtOHnc1	EtOHnc2	Galnc1	Galnc2
13		YER053C-A	YPDcl2	YPDcl3	EtOHcl1	EtOHcl2	Galcl1	YPDnc1	YPDnc2	YPDnc3	YPDnc4	EtOHnc1	EtOHnc2	Galnc1	Galnc2
13	EGD1	YPL037C	YPDcl2	YPDcl3	EtOHcl1	EtOHcl2	Galcl1	YPDnc1	YPDnc2	YPDnc3	YPDnc4	EtOHnc1	EtOHnc2	Galnc1	Galnc2
13	PFA5	YDR459C	YPDcl2	YPDcl3	EtOHcl1	EtOHcl2	Galcl1	YPDnc1	YPDnc2	YPDnc3	YPDnc4	EtOHnc1	EtOHnc2	Galnc1	Galnc2
13	SNX3	YOR357C	YPDcl2	YPDcl3	EtOHcl1	EtOHcl2	Galcl1	YPDnc1	YPDnc2	YPDnc3	YPDnc4	EtOHnc1	EtOHnc2	Galnc1	Galnc2
13	CHS1	YNL192W	YPDcl2	YPDcl3	EtOHcl1	EtOHcl2	Galcl1	YPDnc1	YPDnc2	YPDnc3	YPDnc4	EtOHnc1	EtOHnc2	Galnc1	Galnc2
13	MNP1	YGL068W	YPDcl2	YPDcl3	EtOHcl1	EtOHcl2	Galcl1	YPDnc1	YPDnc2	YPDnc3	YPDnc4	EtOHnc1	EtOHnc2	Galnc1	Galnc2
13		YDL157C	YPDcl2	YPDcl3	EtOHcl1	EtOHcl2	Galcl1	YPDnc1	YPDnc2	YPDnc3	YPDnc4	EtOHnc1	EtOHnc2	Galnc1	Galnc2
13	SGT2	YOR007C	YPDcl2	YPDcl3	EtOHcl1	EtOHcl2	Galcl1	YPDnc1	YPDnc2	YPDnc3	YPDnc4	EtOHnc1	EtOHnc2	Galnc1	Galnc2
13	VID24	YBR105C	YPDcl2	YPDcl3	EtOHcl1	EtOHcl2	Galcl1	YPDnc1	YPDnc2	YPDnc3	YPDnc4	EtOHnc1	EtOHnc2	Galnc1	Galnc2
13	SLX5	YDL013W	YPDcl2	YPDcl3	EtOHcl1	EtOHcl2	Galcl1	YPDnc1	YPDnc2	YPDnc3	YPDnc4	EtOHnc1	EtOHnc2	Galnc1	Galnc2
13	RAD7	YJR052W	YPDcl2	YPDcl3	EtOHcl1	EtOHcl2	Galcl1	YPDnc1	YPDnc2	YPDnc3	YPDnc4	EtOHnc1	EtOHnc2	Galnc1	Galnc2
13	RRP12	YPL012W	YPDcl2	YPDcl3	EtOHcl1	EtOHcl2	Galcl1	YPDnc1	YPDnc2	YPDnc3	YPDnc4	EtOHnc1	EtOHnc2	Galnc1	Galnc2
13	EDE1	YBL047C	YPDcl2	YPDcl3	EtOHcl1	EtOHcl2	Galcl1	YPDnc1	YPDnc2	YPDnc3	YPDnc4	EtOHnc1	EtOHnc2	Galnc1	Galnc2
13	VPS1	YKR001C	YPDcl2	YPDcl3	EtOHcl1	EtOHcl2	Galcl1	YPDnc1	YPDnc2	YPDnc3	YPDnc4	EtOHnc1	EtOHnc2	Galnc1	Galnc2
13	PRM4	YPL156C	YPDcl2	YPDcl3	EtOHcl1	EtOHcl2	Galcl1	YPDnc1	YPDnc2	YPDnc3	YPDnc4	EtOHnc1	EtOHnc2	Galnc1	Galnc2
13	BBC1	YJL020C	YPDcl2	YPDcl3	EtOHcl1	EtOHcl2	Galcl1	YPDnc1	YPDnc2	YPDnc3	YPDnc4	EtOHnc1	EtOHnc2	Galnc1	Galnc2
13	SYH1	YPL105C	YPDcl2	YPDcl3	EtOHcl1	EtOHcl2	Galcl1	YPDnc1	YPDnc2	YPDnc3	YPDnc4	EtOHnc1	EtOHnc2	Galnc1	Galnc2
13		YHR049C-A	YPDcl2	YPDcl3	EtOHcl1	EtOHcl2	Galcl1	YPDnc1	YPDnc2	YPDnc3	YPDnc4	EtOHnc1	EtOHnc2	Galnc1	Galnc2
13		YHL006W-A	YPDcl2	YPDcl3	EtOHcl1	EtOHcl2	Galcl1	YPDnc1	YPDnc2	YPDnc3	YPDnc4	EtOHnc1	EtOHnc2	Galnc1	Galnc2
13	SRB6	YBR253W	YPDcl2	YPDcl3	EtOHcl1	EtOHcl2	Galcl1	YPDnc1	YPDnc2	YPDnc3	YPDnc4	EtOHnc1	EtOHnc2	Galnc1	Galnc2
13	TUB4	YLR212C	YPDcl2	YPDcl3	EtOHcl1	EtOHcl2	Galcl1	YPDnc1	YPDnc2	YPDnc3	YPDnc4	EtOHnc1	EtOHnc2	Galnc1	Galnc2
13	PET18	YCR020C	YPDcl2	YPDcl3	EtOHcl1	EtOHcl2	Galcl1	YPDnc1	YPDnc2	YPDnc3	YPDnc4	EtOHnc1	EtOHnc2	Galnc1	Galnc2
13		YJR141W	YPDcl2	YPDcl3	EtOHcl1	EtOHcl2	Galcl1	YPDnc1	YPDnc2	YPDnc3	YPDnc4	EtOHnc1	EtOHnc2	Galnc1	Galnc2
13	BCP1	YDR361C	YPDcl2	YPDcl3	EtOHcl1	EtOHcl2	Galcl1	YPDnc1	YPDnc2	YPDnc3	YPDnc4	EtOHnc1	EtOHnc2	Galnc1	Galnc2
13		YPL009C	YPDcl2	YPDcl3	EtOHcl1	EtOHcl2	Galcl1	YPDnc1	YPDnc2	YPDnc3	YPDnc4	EtOHnc1	EtOHnc2	Galnc1	Galnc2
13	CRZ1	YNL027W	YPDcl2	YPDcl3	EtOHcl1	EtOHcl2	Galcl1	YPDnc1	YPDnc2	YPDnc3	YPDnc4	EtOHnc1	EtOHnc2	Galnc1	Galnc2
13		YNL211C	YPDcl2	YPDcl3	EtOHcl1	EtOHcl2	Galcl1	YPDnc1	YPDnc2	YPDnc3	YPDnc4	EtOHnc1	EtOHnc2	Galnc1	Galnc2
13	CBP2	YHL038C	YPDcl2	YPDcl3	EtOHcl1	EtOHcl2	Galcl1	YPDnc1	YPDnc2	YPDnc3	YPDnc4	EtOHnc1	EtOHnc2	Galnc1	Galnc2
13	MDY2	YOL111C	YPDcl2	YPDcl3	EtOHcl1	EtOHcl2	Galcl1	YPDnc1	YPDnc2	YPDnc3	YPDnc4	EtOHnc1	EtOHnc2	Galnc1	Galnc2
13	PPH3	YDR075W	YPDcl2	YPDcl3	EtOHcl1	EtOHcl2	Galcl1	YPDnc1	YPDnc2	YPDnc3	YPDnc4	EtOHnc1	EtOHnc2	Galnc1	Galnc2

13	NTF2	YER009W	YPDcl2	YPDcl3	EtOHcl1	EtOHcl2	Galcl1	YPDnc1	YPDnc2	YPDnc3	YPDnc4	EtOHnc1	EtOHnc2	Galnc1	Galnc2
13	MOB2	YFL034C-B	YPDcl2	YPDcl3	EtOHcl1	EtOHcl2	Galcl1	YPDnc1	YPDnc2	YPDnc3	YPDnc4	EtOHnc1	EtOHnc2	Galnc1	Galnc2
13	STS1	YIR011C	YPDcl2	YPDcl3	EtOHcl1	EtOHcl2	Galcl1	YPDnc1	YPDnc2	YPDnc3	YPDnc4	EtOHnc1	EtOHnc2	Galnc1	Galnc2
13	PRC1	YMR297W	YPDcl2	YPDcl3	EtOHcl1	EtOHcl2	Galcl1	YPDnc1	YPDnc2	YPDnc3	YPDnc4	EtOHnc1	EtOHnc2	Galnc1	Galnc2
13	REC107	YJR021C	YPDcl2	YPDcl3	EtOHcl1	EtOHcl2	Galcl1	YPDnc1	YPDnc2	YPDnc3	YPDnc4	EtOHnc1	EtOHnc2	Galnc1	Galnc2
13	CHL4	YDR254W	YPDcl2	YPDcl3	EtOHcl1	EtOHcl2	Galcl1	YPDnc1	YPDnc2	YPDnc3	YPDnc4	EtOHnc1	EtOHnc2	Galnc1	Galnc2
13	RPL18B	YNL301C	YPDcl2	YPDcl3	EtOHcl1	EtOHcl2	Galcl1	YPDnc1	YPDnc2	YPDnc3	YPDnc4	EtOHnc1	EtOHnc2	Galnc1	Galnc2
13		YBR255C-A	YPDcl2	YPDcl3	EtOHcl1	EtOHcl2	Galcl1	YPDnc1	YPDnc2	YPDnc3	YPDnc4	EtOHnc1	EtOHnc2	Galnc1	Galnc2
13	WHI4	YDL224C	YPDcl2	YPDcl3	EtOHcl1	EtOHcl2	Galcl1	YPDnc1	YPDnc2	YPDnc3	YPDnc4	EtOHnc1	EtOHnc2	Galnc1	Galnc2
13	ARC1	YGL105W	YPDcl2	YPDcl3	EtOHcl1	EtOHcl2	Galcl1	YPDnc1	YPDnc2	YPDnc3	YPDnc4	EtOHnc1	EtOHnc2	Galnc1	Galnc2
13	UTP22	YGR090W	YPDcl2	YPDcl3	EtOHcl1	EtOHcl2	Galcl1	YPDnc1	YPDnc2	YPDnc3	YPDnc4	EtOHnc1	EtOHnc2	Galnc1	Galnc2
13	ATP11	YNL315C	YPDcl2	YPDcl3	EtOHcl1	EtOHcl2	Galcl1	YPDnc1	YPDnc2	YPDnc3	YPDnc4	EtOHnc1	EtOHnc2	Galnc1	Galnc2
13	LDB18	YLL049W	YPDcl2	YPDcl3	EtOHcl1	EtOHcl2	Galcl1	YPDnc1	YPDnc2	YPDnc3	YPDnc4	EtOHnc1	EtOHnc2	Galnc1	Galnc2
13	URA1	YKL216W	YPDcl2	YPDcl3	EtOHcl1	EtOHcl2	Galcl1	YPDnc1	YPDnc2	YPDnc3	YPDnc4	EtOHnc1	EtOHnc2	Galnc1	Galnc2
13	NBP2	YDR162C	YPDcl2	YPDcl3	EtOHcl1	EtOHcl2	Galcl1	YPDnc1	YPDnc2	YPDnc3	YPDnc4	EtOHnc1	EtOHnc2	Galnc1	Galnc2
13	YIH1	YCR059C	YPDcl2	YPDcl3	EtOHcl1	EtOHcl2	Galcl1	YPDnc1	YPDnc2	YPDnc3	YPDnc4	EtOHnc1	EtOHnc2	Galnc1	Galnc2
13	PET10	YKR046C	YPDcl2	YPDcl3	EtOHcl1	EtOHcl2	Galcl1	YPDnc1	YPDnc2	YPDnc3	YPDnc4	EtOHnc1	EtOHnc2	Galnc1	Galnc2
13	GID7	YCL039W	YPDcl2	YPDcl3	EtOHcl1	EtOHcl2	Galcl1	YPDnc1	YPDnc2	YPDnc3	YPDnc4	EtOHnc1	EtOHnc2	Galnc1	Galnc2
13	BTS1	YPL069C	YPDcl2	YPDcl3	EtOHcl1	EtOHcl2	Galcl1	YPDnc1	YPDnc2	YPDnc3	YPDnc4	EtOHnc1	EtOHnc2	Galnc1	Galnc2
13	PRP46	YPL151C	YPDcl2	YPDcl3	EtOHcl1	EtOHcl2	Galcl1	YPDnc1	YPDnc2	YPDnc3	YPDnc4	EtOHnc1	EtOHnc2	Galnc1	Galnc2
13	BOP3	YNL042W	YPDcl2	YPDcl3	EtOHcl1	EtOHcl2	Galcl1	YPDnc1	YPDnc2	YPDnc3	YPDnc4	EtOHnc1	EtOHnc2	Galnc1	Galnc2
13	SET5	YHR207C	YPDcl2	YPDcl3	EtOHcl1	EtOHcl2	Galcl1	YPDnc1	YPDnc2	YPDnc3	YPDnc4	EtOHnc1	EtOHnc2	Galnc1	Galnc2
13	HSP42	YDR171W	YPDcl2	YPDcl3	EtOHcl1	EtOHcl2	Galcl1	YPDnc1	YPDnc2	YPDnc3	YPDnc4	EtOHnc1	EtOHnc2	Galnc1	Galnc2
13	PEX29	YDR479C	YPDcl2	YPDcl3	EtOHcl1	EtOHcl2	Galcl1	YPDnc1	YPDnc2	YPDnc3	YPDnc4	EtOHnc1	EtOHnc2	Galnc1	Galnc2
13	MTW1	YAL034W-	YPDcl2	YPDcl3	EtOHcl1	EtOHcl2	Galcl1	YPDnc1	YPDnc2	YPDnc3	YPDnc4	EtOHnc1	EtOHnc2	Galnc1	Galnc2
13	HNT3	YOR258W	YPDcl2	YPDcl3	EtOHcl1	EtOHcl2	Galcl1	YPDnc1	YPDnc2	YPDnc3	YPDnc4	EtOHnc1	EtOHnc2	Galnc1	Galnc2
13	BIO4	YNR057C	YPDcl2	YPDcl3	EtOHcl1	EtOHcl2	Galcl1	YPDnc1	YPDnc2	YPDnc3	YPDnc4	EtOHnc1	EtOHnc2	Galnc1	Galnc2
13		YGR161W-	YPDcl2	YPDcl3	EtOHcl1	EtOHcl2	Galcl1	YPDnc1	YPDnc2	YPDnc3	YPDnc4	EtOHnc1	EtOHnc2	Galnc1	Galnc2
13	LPD1	YFL018C	YPDcl2	YPDcl3	EtOHcl1	EtOHcl2	Galcl1	YPDnc1	YPDnc2	YPDnc3	YPDnc4	EtOHnc1	EtOHnc2	Galnc1	Galnc2
13	MDJ2	YNL328C	YPDcl2	YPDcl3	EtOHcl1	EtOHcl2	Galcl1	YPDnc1	YPDnc2	YPDnc3	YPDnc4	EtOHnc1	EtOHnc2	Galnc1	Galnc2
13		YCL049C	YPDcl2	YPDcl3	EtOHcl1	EtOHcl2	Galcl1	YPDnc1	YPDnc2	YPDnc3	YPDnc4	EtOHnc1	EtOHnc2	Galnc1	Galnc2
13		YLR072W	YPDcl2	YPDcl3	EtOHcl1	EtOHcl2	Galcl1	YPDnc1	YPDnc2	YPDnc3	YPDnc4	EtOHnc1	EtOHnc2	Galnc1	Galnc2
13	UBA4	YHR111W	YPDcl2	YPDcl3	EtOHcl1	EtOHcl2	Galcl1	YPDnc1	YPDnc2	YPDnc3	YPDnc4	EtOHnc1	EtOHnc2	Galnc1	Galnc2
13	APM3	YBR288C	YPDcl2	YPDcl3	EtOHcl1	EtOHcl2	Galcl1	YPDnc1	YPDnc2	YPDnc3	YPDnc4	EtOHnc1	EtOHnc2	Galnc1	Galnc2

13	THI20	YOL055C	YPDcl2	YPDcl3	EtOHcl1	EtOHcl2	Galcl1	YPDnc1	YPDnc2	YPDnc3	YPDnc4	EtOHnc1	EtOHnc2	Galnc1	Galnc2
13	ALG2	YGL065C	YPDcl2	YPDcl3	EtOHcl1	EtOHcl2	Galcl1	YPDnc1	YPDnc2	YPDnc3	YPDnc4	EtOHnc1	EtOHnc2	Galnc1	Galnc2
13	ARD1	YHR013C	YPDcl2	YPDcl3	EtOHcl1	EtOHcl2	Galcl1	YPDnc1	YPDnc2	YPDnc3	YPDnc4	EtOHnc1	EtOHnc2	Galnc1	Galnc2
13	SLY41	YOR307C	YPDcl2	YPDcl3	EtOHcl1	EtOHcl2	Galcl1	YPDnc1	YPDnc2	YPDnc3	YPDnc4	EtOHnc1	EtOHnc2	Galnc1	Galnc2
13		YAL042C-A	YPDcl2	YPDcl3	EtOHcl1	EtOHcl2	Galcl1	YPDnc1	YPDnc2	YPDnc3	YPDnc4	EtOHnc1	EtOHnc2	Galnc1	Galnc2
13	GTT1	YIR038C	YPDcl2	YPDcl3	EtOHcl1	EtOHcl2	Galcl1	YPDnc1	YPDnc2	YPDnc3	YPDnc4	EtOHnc1	EtOHnc2	Galnc1	Galnc2
13		YLR230W	YPDcl2	YPDcl3	EtOHcl1	EtOHcl2	Galcl1	YPDnc1	YPDnc2	YPDnc3	YPDnc4	EtOHnc1	EtOHnc2	Galnc1	Galnc2
13	AIM1	YAL046C	YPDcl2	YPDcl3	EtOHcl1	EtOHcl2	Galcl1	YPDnc1	YPDnc2	YPDnc3	YPDnc4	EtOHnc1	EtOHnc2	Galnc1	Galnc2
13	RSA1	YPL193W	YPDcl2	YPDcl3	EtOHcl1	EtOHcl2	Galcl1	YPDnc1	YPDnc2	YPDnc3	YPDnc4	EtOHnc1	EtOHnc2	Galnc1	Galnc2
13		YJL045W	YPDcl2	YPDcl3	EtOHcl1	EtOHcl2	Galcl1	YPDnc1	YPDnc2	YPDnc3	YPDnc4	EtOHnc1	EtOHnc2	Galnc1	Galnc2
13		YKR078W	YPDcl2	YPDcl3	EtOHcl1	EtOHcl2	Galcl1	YPDnc1	YPDnc2	YPDnc3	YPDnc4	EtOHnc1	EtOHnc2	Galnc1	Galnc2
13	VPS69	YPR087W	YPDcl2	YPDcl3	EtOHcl1	EtOHcl2	Galcl1	YPDnc1	YPDnc2	YPDnc3	YPDnc4	EtOHnc1	EtOHnc2	Galnc1	Galnc2
13	RIM20	YOR275C	YPDcl2	YPDcl3	EtOHcl1	EtOHcl2	Galcl1	YPDnc1	YPDnc2	YPDnc3	YPDnc4	EtOHnc1	EtOHnc2	Galnc1	Galnc2
13	BET5	YML077W	YPDcl2	YPDcl3	EtOHcl1	EtOHcl2	Galcl1	YPDnc1	YPDnc2	YPDnc3	YPDnc4	EtOHnc1	EtOHnc2	Galnc1	Galnc2
13	MED6	YHR058C	YPDcl2	YPDcl3	EtOHcl1	EtOHcl2	Galcl1	YPDnc1	YPDnc2	YPDnc3	YPDnc4	EtOHnc1	EtOHnc2	Galnc1	Galnc2
13	SEC23	YPR181C	YPDcl2	YPDcl3	EtOHcl1	EtOHcl2	Galcl1	YPDnc1	YPDnc2	YPDnc3	YPDnc4	EtOHnc1	EtOHnc2	Galnc1	Galnc2
13		YDL025W-A	YPDcl2	YPDcl3	EtOHcl1	EtOHcl2	Galcl1	YPDnc1	YPDnc2	YPDnc3	YPDnc4	EtOHnc1	EtOHnc2	Galnc1	Galnc2
13	PHO88	YBR106W	YPDcl2	YPDcl3	EtOHcl1	EtOHcl2	Galcl1	YPDnc1	YPDnc2	YPDnc3	YPDnc4	EtOHnc1	EtOHnc2	Galnc1	Galnc2
13	ARG4	YHR018C	YPDcl2	YPDcl3	EtOHcl1	EtOHcl2	Galcl1	YPDnc1	YPDnc2	YPDnc3	YPDnc4	EtOHnc1	EtOHnc2	Galnc1	Galnc2
13		YPL197C	YPDcl2	YPDcl3	EtOHcl1	EtOHcl2	Galcl1	YPDnc1	YPDnc2	YPDnc3	YPDnc4	EtOHnc1	EtOHnc2	Galnc1	Galnc2
13	NPL4	YBR170C	YPDcl2	YPDcl3	EtOHcl1	EtOHcl2	Galcl1	YPDnc1	YPDnc2	YPDnc3	YPDnc4	EtOHnc1	EtOHnc2	Galnc1	Galnc2
13		YDL211C	YPDcl2	YPDcl3	EtOHcl1	EtOHcl2	Galcl1	YPDnc1	YPDnc2	YPDnc3	YPDnc4	EtOHnc1	EtOHnc2	Galnc1	Galnc2
13	ATP4	YPL078C	YPDcl2	YPDcl3	EtOHcl1	EtOHcl2	Galcl1	YPDnc1	YPDnc2	YPDnc3	YPDnc4	EtOHnc1	EtOHnc2	Galnc1	Galnc2
13		YLR064W	YPDcl2	YPDcl3	EtOHcl1	EtOHcl2	Galcl1	YPDnc1	YPDnc2	YPDnc3	YPDnc4	EtOHnc1	EtOHnc2	Galnc1	Galnc2
13	TDH3	YGR192C	YPDcl2	YPDcl3	EtOHcl1	EtOHcl2	Galcl1	YPDnc1	YPDnc2	YPDnc3	YPDnc4	EtOHnc1	EtOHnc2	Galnc1	Galnc2
13	RIM8	YGL045W	YPDcl2	YPDcl3	EtOHcl1	EtOHcl2	Galcl1	YPDnc1	YPDnc2	YPDnc3	YPDnc4	EtOHnc1	EtOHnc2	Galnc1	Galnc2
13	MDH3	YDL078C	YPDcl2	YPDcl3	EtOHcl1	EtOHcl2	Galcl1	YPDnc1	YPDnc2	YPDnc3	YPDnc4	EtOHnc1	EtOHnc2	Galnc1	Galnc2
13	TMA29	YMR226C	YPDcl2	YPDcl3	EtOHcl1	EtOHcl2	Galcl1	YPDnc1	YPDnc2	YPDnc3	YPDnc4	EtOHnc1	EtOHnc2	Galnc1	Galnc2
13	RPN10	YHR200W	YPDcl2	YPDcl3	EtOHcl1	EtOHcl2	Galcl1	YPDnc1	YPDnc2	YPDnc3	YPDnc4	EtOHnc1	EtOHnc2	Galnc1	Galnc2
13		YBR219C	YPDcl2	YPDcl3	EtOHcl1	EtOHcl2	Galcl1	YPDnc1	YPDnc2	YPDnc3	YPDnc4	EtOHnc1	EtOHnc2	Galnc1	Galnc2
13		YLR379W	YPDcl2	YPDcl3	EtOHcl1	EtOHcl2	Galcl1	YPDnc1	YPDnc2	YPDnc3	YPDnc4	EtOHnc1	EtOHnc2	Galnc1	Galnc2
13	COQ4	YDR204W	YPDcl2	YPDcl3	EtOHcl1	EtOHcl2	Galcl1	YPDnc1	YPDnc2	YPDnc3	YPDnc4	EtOHnc1	EtOHnc2	Galnc1	Galnc2
13	BEM4	YPL161C	YPDcl2	YPDcl3	EtOHcl1	EtOHcl2	Galcl1	YPDnc1	YPDnc2	YPDnc3	YPDnc4	EtOHnc1	EtOHnc2	Galnc1	Galnc2
13	NDE1	YMR145C	YPDcl2	YPDcl3	EtOHcl1	EtOHcl2	Galcl1	YPDnc1	YPDnc2	YPDnc3	YPDnc4	EtOHnc1	EtOHnc2	Galnc1	Galnc2

13		YIR017W-A	YPDcl2	YPDcl3	EtOHcl1	EtOHcl2	Galcl1	YPDnc1	YPDnc2	YPDnc3	YPDnc4	EtOHnc1	EtOHnc2	Galnc1	Galnc2
13	PRO1	YDR300C	YPDcl2	YPDcl3	EtOHcl1	EtOHcl2	Galcl1	YPDnc1	YPDnc2	YPDnc3	YPDnc4	EtOHnc1	EtOHnc2	Galnc1	Galnc2
13	CDC42	YLR229C	YPDcl2	YPDcl3	EtOHcl1	EtOHcl2	Galcl1	YPDnc1	YPDnc2	YPDnc3	YPDnc4	EtOHnc1	EtOHnc2	Galnc1	Galnc2
13	RPN13	YLR421C	YPDcl2	YPDcl3	EtOHcl1	EtOHcl2	Galcl1	YPDnc1	YPDnc2	YPDnc3	YPDnc4	EtOHnc1	EtOHnc2	Galnc1	Galnc2
13	DAN4	YJR151C	YPDcl2	YPDcl3	EtOHcl1	EtOHcl2	Galcl1	YPDnc1	YPDnc2	YPDnc3	YPDnc4	EtOHnc1	EtOHnc2	Galnc1	Galnc2
13	PNG1	YPL096W	YPDcl2	YPDcl3	EtOHcl1	EtOHcl2	Galcl1	YPDnc1	YPDnc2	YPDnc3	YPDnc4	EtOHnc1	EtOHnc2	Galnc1	Galnc2
13	YMD8	YML038C	YPDcl2	YPDcl3	EtOHcl1	EtOHcl2	Galcl1	YPDnc1	YPDnc2	YPDnc3	YPDnc4	EtOHnc1	EtOHnc2	Galnc1	Galnc2
13	YIA6	YIL006W	YPDcl2	YPDcl3	EtOHcl1	EtOHcl2	Galcl1	YPDnc1	YPDnc2	YPDnc3	YPDnc4	EtOHnc1	EtOHnc2	Galnc1	Galnc2
13	OMS1	YDR316W	YPDcl2	YPDcl3	EtOHcl1	EtOHcl2	Galcl1	YPDnc1	YPDnc2	YPDnc3	YPDnc4	EtOHnc1	EtOHnc2	Galnc1	Galnc2
13	PDR18	YNR070W	YPDcl2	YPDcl3	EtOHcl1	EtOHcl2	Galcl1	YPDnc1	YPDnc2	YPDnc3	YPDnc4	EtOHnc1	EtOHnc2	Galnc1	Galnc2
13	VPS51	YKR020W	YPDcl2	YPDcl3	EtOHcl1	EtOHcl2	Galcl1	YPDnc1	YPDnc2	YPDnc3	YPDnc4	EtOHnc1	EtOHnc2	Galnc1	Galnc2
13		YOL164W-/	YPDcl2	YPDcl3	EtOHcl1	EtOHcl2	Galcl1	YPDnc1	YPDnc2	YPDnc3	YPDnc4	EtOHnc1	EtOHnc2	Galnc1	Galnc2
13	DYS1	YHR068W	YPDcl2	YPDcl3	EtOHcl1	EtOHcl2	Galcl1	YPDnc1	YPDnc2	YPDnc3	YPDnc4	EtOHnc1	EtOHnc2	Galnc1	Galnc2
13		YEL010W	YPDcl2	YPDcl3	EtOHcl1	EtOHcl2	Galcl1	YPDnc1	YPDnc2	YPDnc3	YPDnc4	EtOHnc1	EtOHnc2	Galnc1	Galnc2
13	AIM14	YGL160W	YPDcl2	YPDcl3	EtOHcl1	EtOHcl2	Galcl1	YPDnc1	YPDnc2	YPDnc3	YPDnc4	EtOHnc1	EtOHnc2	Galnc1	Galnc2
13		YGR042W	YPDcl2	YPDcl3	EtOHcl1	EtOHcl2	Galcl1	YPDnc1	YPDnc2	YPDnc3	YPDnc4	EtOHnc1	EtOHnc2	Galnc1	Galnc2
13		YCR041W	YPDcl2	YPDcl3	EtOHcl1	EtOHcl2	Galcl1	YPDnc1	YPDnc2	YPDnc3	YPDnc4	EtOHnc1	EtOHnc2	Galnc1	Galnc2
13	NPR2	YEL062W	YPDcl2	YPDcl3	EtOHcl1	EtOHcl2	Galcl1	YPDnc1	YPDnc2	YPDnc3	YPDnc4	EtOHnc1	EtOHnc2	Galnc1	Galnc2
13		YLR143W	YPDcl2	YPDcl3	EtOHcl1	EtOHcl2	Galcl1	YPDnc1	YPDnc2	YPDnc3	YPDnc4	EtOHnc1	EtOHnc2	Galnc1	Galnc2
13	STO1	YMR125W	YPDcl2	YPDcl3	EtOHcl1	EtOHcl2	Galcl1	YPDnc1	YPDnc2	YPDnc3	YPDnc4	EtOHnc1	EtOHnc2	Galnc1	Galnc2
13		YLR126C	YPDcl2	YPDcl3	EtOHcl1	EtOHcl2	Galcl1	YPDnc1	YPDnc2	YPDnc3	YPDnc4	EtOHnc1	EtOHnc2	Galnc1	Galnc2
13	NRK1	YNL129W	YPDcl2	YPDcl3	EtOHcl1	EtOHcl2	Galcl1	YPDnc1	YPDnc2	YPDnc3	YPDnc4	EtOHnc1	EtOHnc2	Galnc1	Galnc2
13	RPT6	YGL048C	YPDcl2	YPDcl3	EtOHcl1	EtOHcl2	Galcl1	YPDnc1	YPDnc2	YPDnc3	YPDnc4	EtOHnc1	EtOHnc2	Galnc1	Galnc2
13	TEC1	YBR083W	YPDcl2	YPDcl3	EtOHcl1	EtOHcl2	Galcl1	YPDnc1	YPDnc2	YPDnc3	YPDnc4	EtOHnc1	EtOHnc2	Galnc1	Galnc2
13		YJR012C	YPDcl2	YPDcl3	EtOHcl1	EtOHcl2	Galcl1	YPDnc1	YPDnc2	YPDnc3	YPDnc4	EtOHnc1	EtOHnc2	Galnc1	Galnc2
13	PUS6	YGR169C	YPDcl2	YPDcl3	EtOHcl1	EtOHcl2	Galcl1	YPDnc1	YPDnc2	YPDnc3	YPDnc4	EtOHnc1	EtOHnc2	Galnc1	Galnc2
13		YJL127W-A	YPDcl2	YPDcl3	EtOHcl1	EtOHcl2	Galcl1	YPDnc1	YPDnc2	YPDnc3	YPDnc4	EtOHnc1	EtOHnc2	Galnc1	Galnc2
13		YHR138C	YPDcl2	YPDcl3	EtOHcl1	EtOHcl2	Galcl1	YPDnc1	YPDnc2	YPDnc3	YPDnc4	EtOHnc1	EtOHnc2	Galnc1	Galnc2
13	RPS18B	YML026C	YPDcl2	YPDcl3	EtOHcl1	EtOHcl2	Galcl1	YPDnc1	YPDnc2	YPDnc3	YPDnc4	EtOHnc1	EtOHnc2	Galnc1	Galnc2
13	MAK10	YEL053C	YPDcl2	YPDcl3	EtOHcl1	EtOHcl2	Galcl1	YPDnc1	YPDnc2	YPDnc3	YPDnc4	EtOHnc1	EtOHnc2	Galnc1	Galnc2
13	VOA1	YGR106C	YPDcl2	YPDcl3	EtOHcl1	EtOHcl2	Galcl1	YPDnc1	YPDnc2	YPDnc3	YPDnc4	EtOHnc1	EtOHnc2	Galnc1	Galnc2
13		YLR444C	YPDcl2	YPDcl3	EtOHcl1	EtOHcl2	Galcl1	YPDnc1	YPDnc2	YPDnc3	YPDnc4	EtOHnc1	EtOHnc2	Galnc1	Galnc2
13		YJR128W	YPDcl2	YPDcl3	EtOHcl1	EtOHcl2	Galcl1	YPDnc1	YPDnc2	YPDnc3	YPDnc4	EtOHnc1	EtOHnc2	Galnc1	Galnc2
13		YOL099C	YPDcl2	YPDcl3	EtOHcl1	EtOHcl2	Galcl1	YPDnc1	YPDnc2	YPDnc3	YPDnc4	EtOHnc1	EtOHnc2	Galnc1	Galnc2

13	YKE4	YIL023C	YPDcl2	YPDcl3	EtOHcl1	EtOHcl2	Galcl1	YPDnc1	YPDnc2	YPDnc3	YPDnc4	EtOHnc1	EtOHnc2	Galnc1	Galnc2
13	SNF4	YGL115W	YPDcl2	YPDcl3	EtOHcl1	EtOHcl2	Galcl1	YPDnc1	YPDnc2	YPDnc3	YPDnc4	EtOHnc1	EtOHnc2	Galnc1	Galnc2
13	ZEO1	YOL109W	YPDcl2	YPDcl3	EtOHcl1	EtOHcl2	Galcl1	YPDnc1	YPDnc2	YPDnc3	YPDnc4	EtOHnc1	EtOHnc2	Galnc1	Galnc2
13	WSC4	YHL028W	YPDcl2	YPDcl3	EtOHcl1	EtOHcl2	Galcl1	YPDnc1	YPDnc2	YPDnc3	YPDnc4	EtOHnc1	EtOHnc2	Galnc1	Galnc2
13	TGL3	YMR313C	YPDcl2	YPDcl3	EtOHcl1	EtOHcl2	Galcl1	YPDnc1	YPDnc2	YPDnc3	YPDnc4	EtOHnc1	EtOHnc2	Galnc1	Galnc2
13	DCR2	YLR361C	YPDcl2	YPDcl3	EtOHcl1	EtOHcl2	Galcl1	YPDnc1	YPDnc2	YPDnc3	YPDnc4	EtOHnc1	EtOHnc2	Galnc1	Galnc2
13	OST5	YGL226C-A	YPDcl2	YPDcl3	EtOHcl1	EtOHcl2	Galcl1	YPDnc1	YPDnc2	YPDnc3	YPDnc4	EtOHnc1	EtOHnc2	Galnc1	Galnc2
13		YDL177C	YPDcl2	YPDcl3	EtOHcl1	EtOHcl2	Galcl1	YPDnc1	YPDnc2	YPDnc3	YPDnc4	EtOHnc1	EtOHnc2	Galnc1	Galnc2
13	MEF1	YLR069C	YPDcl2	YPDcl3	EtOHcl1	EtOHcl2	Galcl1	YPDnc1	YPDnc2	YPDnc3	YPDnc4	EtOHnc1	EtOHnc2	Galnc1	Galnc2
13	MAK21	YDR060W	YPDcl2	YPDcl3	EtOHcl1	EtOHcl2	Galcl1	YPDnc1	YPDnc2	YPDnc3	YPDnc4	EtOHnc1	EtOHnc2	Galnc1	Galnc2
13	LEA1	YPL213W	YPDcl2	YPDcl3	EtOHcl1	EtOHcl2	Galcl1	YPDnc1	YPDnc2	YPDnc3	YPDnc4	EtOHnc1	EtOHnc2	Galnc1	Galnc2
13	SDH2	YLL041C	YPDcl2	YPDcl3	EtOHcl1	EtOHcl2	Galcl1	YPDnc1	YPDnc2	YPDnc3	YPDnc4	EtOHnc1	EtOHnc2	Galnc1	Galnc2
13	LSM2	YBL026W	YPDcl2	YPDcl3	EtOHcl1	EtOHcl2	Galcl1	YPDnc1	YPDnc2	YPDnc3	YPDnc4	EtOHnc1	EtOHnc2	Galnc1	Galnc2
13		YBR076C-A	YPDcl2	YPDcl3	EtOHcl1	EtOHcl2	Galcl1	YPDnc1	YPDnc2	YPDnc3	YPDnc4	EtOHnc1	EtOHnc2	Galnc1	Galnc2
13		YNR021W	YPDcl2	YPDcl3	EtOHcl1	EtOHcl2	Galcl1	YPDnc1	YPDnc2	YPDnc3	YPDnc4	EtOHnc1	EtOHnc2	Galnc1	Galnc2
13	TWF1	YGR080W	YPDcl2	YPDcl3	EtOHcl1	EtOHcl2	Galcl1	YPDnc1	YPDnc2	YPDnc3	YPDnc4	EtOHnc1	EtOHnc2	Galnc1	Galnc2
13		YBL055C	YPDcl2	YPDcl3	EtOHcl1	EtOHcl2	Galcl1	YPDnc1	YPDnc2	YPDnc3	YPDnc4	EtOHnc1	EtOHnc2	Galnc1	Galnc2
13	PPT2	YPL148C	YPDcl2	YPDcl3	EtOHcl1	EtOHcl2	Galcl1	YPDnc1	YPDnc2	YPDnc3	YPDnc4	EtOHnc1	EtOHnc2	Galnc1	Galnc2
13	YPT1	YFL038C	YPDcl2	YPDcl3	EtOHcl1	EtOHcl2	Galcl1	YPDnc1	YPDnc2	YPDnc3	YPDnc4	EtOHnc1	EtOHnc2	Galnc1	Galnc2
13	HSP82	YPL240C	YPDcl2	YPDcl3	EtOHcl1	EtOHcl2	Galcl1	YPDnc1	YPDnc2	YPDnc3	YPDnc4	EtOHnc1	EtOHnc2	Galnc1	Galnc2
13	PRM2	YIL037C	YPDcl2	YPDcl3	EtOHcl1	EtOHcl2	Galcl1	YPDnc1	YPDnc2	YPDnc3	YPDnc4	EtOHnc1	EtOHnc2	Galnc1	Galnc2
13	TIP20	YGL145W	YPDcl2	YPDcl3	EtOHcl1	EtOHcl2	Galcl1	YPDnc1	YPDnc2	YPDnc3	YPDnc4	EtOHnc1	EtOHnc2	Galnc1	Galnc2
13	ARO7	YPR060C	YPDcl2	YPDcl3	EtOHcl1	EtOHcl2	Galcl1	YPDnc1	YPDnc2	YPDnc3	YPDnc4	EtOHnc1	EtOHnc2	Galnc1	Galnc2
13	HRB1	YNL004W	YPDcl2	YPDcl3	EtOHcl1	EtOHcl2	Galcl1	YPDnc1	YPDnc2	YPDnc3	YPDnc4	EtOHnc1	EtOHnc2	Galnc1	Galnc2
13		YLR232W	YPDcl2	YPDcl3	EtOHcl1	EtOHcl2	Galcl1	YPDnc1	YPDnc2	YPDnc3	YPDnc4	EtOHnc1	EtOHnc2	Galnc1	Galnc2
13	RRP4	YHR069C	YPDcl2	YPDcl3	EtOHcl1	EtOHcl2	Galcl1	YPDnc1	YPDnc2	YPDnc3	YPDnc4	EtOHnc1	EtOHnc2	Galnc1	Galnc2
13		YEL043W	YPDcl2	YPDcl3	EtOHcl1	EtOHcl2	Galcl1	YPDnc1	YPDnc2	YPDnc3	YPDnc4	EtOHnc1	EtOHnc2	Galnc1	Galnc2
13	CWC24	YLR323C	YPDcl2	YPDcl3	EtOHcl1	EtOHcl2	Galcl1	YPDnc1	YPDnc2	YPDnc3	YPDnc4	EtOHnc1	EtOHnc2	Galnc1	Galnc2
13	KTR6	YPL053C	YPDcl2	YPDcl3	EtOHcl1	EtOHcl2	Galcl1	YPDnc1	YPDnc2	YPDnc3	YPDnc4	EtOHnc1	EtOHnc2	Galnc1	Galnc2
13	NUP49	YGL172W	YPDcl2	YPDcl3	EtOHcl1	EtOHcl2	Galcl1	YPDnc1	YPDnc2	YPDnc3	YPDnc4	EtOHnc1	EtOHnc2	Galnc1	Galnc2
13		YKL162C	YPDcl2	YPDcl3	EtOHcl1	EtOHcl2	Galcl1	YPDnc1	YPDnc2	YPDnc3	YPDnc4	EtOHnc1	EtOHnc2	Galnc1	Galnc2
13	RIM101	YHL027W	YPDcl2	YPDcl3	EtOHcl1	EtOHcl2	Galcl1	YPDnc1	YPDnc2	YPDnc3	YPDnc4	EtOHnc1	EtOHnc2	Galnc1	Galnc2
13	NIP7	YPL211W	YPDcl2	YPDcl3	EtOHcl1	EtOHcl2	Galcl1	YPDnc1	YPDnc2	YPDnc3	YPDnc4	EtOHnc1	EtOHnc2	Galnc1	Galnc2
13	PPN1	YDR452W	YPDcl2	YPDcl3	EtOHcl1	EtOHcl2	Galcl1	YPDnc1	YPDnc2	YPDnc3	YPDnc4	EtOHnc1	EtOHnc2	Galnc1	Galnc2

13		YOL047C	YPDcl2	YPDcl3	EtOHcl1	EtOHcl2	Galcl1	YPDnc1	YPDnc2	YPDnc3	YPDnc4	EtOHnc1	EtOHnc2	Galnc1	Galnc2
13	SMD3	YLR147C	YPDcl2	YPDcl3	EtOHcl1	EtOHcl2	Galcl1	YPDnc1	YPDnc2	YPDnc3	YPDnc4	EtOHnc1	EtOHnc2	Galnc1	Galnc2
13	ERP3	YDL018C	YPDcl2	YPDcl3	EtOHcl1	EtOHcl2	Galcl1	YPDnc1	YPDnc2	YPDnc3	YPDnc4	EtOHnc1	EtOHnc2	Galnc1	Galnc2
13	ERV25	YML012W	YPDcl2	YPDcl3	EtOHcl1	EtOHcl2	Galcl1	YPDnc1	YPDnc2	YPDnc3	YPDnc4	EtOHnc1	EtOHnc2	Galnc1	Galnc2
13	SNP1	YIL061C	YPDcl2	YPDcl3	EtOHcl1	EtOHcl2	Galcl1	YPDnc1	YPDnc2	YPDnc3	YPDnc4	EtOHnc1	EtOHnc2	Galnc1	Galnc2
13	IKI1	YHR187W	YPDcl2	YPDcl3	EtOHcl1	EtOHcl2	Galcl1	YPDnc1	YPDnc2	YPDnc3	YPDnc4	EtOHnc1	EtOHnc2	Galnc1	Galnc2
13	TIS11	YLR136C	YPDcl2	YPDcl3	EtOHcl1	EtOHcl2	Galcl1	YPDnc1	YPDnc2	YPDnc3	YPDnc4	EtOHnc1	EtOHnc2	Galnc1	Galnc2
13	MMS22	YLR320W	YPDcl2	YPDcl3	EtOHcl1	EtOHcl2	Galcl1	YPDnc1	YPDnc2	YPDnc3	YPDnc4	EtOHnc1	EtOHnc2	Galnc1	Galnc2
13	TSR1	YDL060W	YPDcl2	YPDcl3	EtOHcl1	EtOHcl2	Galcl1	YPDnc1	YPDnc2	YPDnc3	YPDnc4	EtOHnc1	EtOHnc2	Galnc1	Galnc2
13	KTR7	YIL085C	YPDcl2	YPDcl3	EtOHcl1	EtOHcl2	Galcl1	YPDnc1	YPDnc2	YPDnc3	YPDnc4	EtOHnc1	EtOHnc2	Galnc1	Galnc2
13	PPE1	YHR075C	YPDcl2	YPDcl3	EtOHcl1	EtOHcl2	Galcl1	YPDnc1	YPDnc2	YPDnc3	YPDnc4	EtOHnc1	EtOHnc2	Galnc1	Galnc2
13	GCN1	YGL195W	YPDcl2	YPDcl3	EtOHcl1	EtOHcl2	Galcl1	YPDnc1	YPDnc2	YPDnc3	YPDnc4	EtOHnc1	EtOHnc2	Galnc1	Galnc2
13	MCH4	YOL119C	YPDcl2	YPDcl3	EtOHcl1	EtOHcl2	Galcl1	YPDnc1	YPDnc2	YPDnc3	YPDnc4	EtOHnc1	EtOHnc2	Galnc1	Galnc2
13	PCL1	YNL289W	YPDcl2	YPDcl3	EtOHcl1	EtOHcl2	Galcl1	YPDnc1	YPDnc2	YPDnc3	YPDnc4	EtOHnc1	EtOHnc2	Galnc1	Galnc2
13	ADO1	YJR105W	YPDcl2	YPDcl3	EtOHcl1	EtOHcl2	Galcl1	YPDnc1	YPDnc2	YPDnc3	YPDnc4	EtOHnc1	EtOHnc2	Galnc1	Galnc2
13	SPT2	YER161C	YPDcl2	YPDcl3	EtOHcl1	EtOHcl2	Galcl1	YPDnc1	YPDnc2	YPDnc3	YPDnc4	EtOHnc1	EtOHnc2	Galnc1	Galnc2
13	MRPS12	YNR036C	YPDcl2	YPDcl3	EtOHcl1	EtOHcl2	Galcl1	YPDnc1	YPDnc2	YPDnc3	YPDnc4	EtOHnc1	EtOHnc2	Galnc1	Galnc2
13	RKR1	YMR247C	YPDcl2	YPDcl3	EtOHcl1	EtOHcl2	Galcl1	YPDnc1	YPDnc2	YPDnc3	YPDnc4	EtOHnc1	EtOHnc2	Galnc1	Galnc2
13		YMR111C	YPDcl2	YPDcl3	EtOHcl1	EtOHcl2	Galcl1	YPDnc1	YPDnc2	YPDnc3	YPDnc4	EtOHnc1	EtOHnc2	Galnc1	Galnc2
13	MRPL49	YJL096W	YPDcl2	YPDcl3	EtOHcl1	EtOHcl2	Galcl1	YPDnc1	YPDnc2	YPDnc3	YPDnc4	EtOHnc1	EtOHnc2	Galnc1	Galnc2
13	MPE1	YKL059C	YPDcl2	YPDcl3	EtOHcl1	EtOHcl2	Galcl1	YPDnc1	YPDnc2	YPDnc3	YPDnc4	EtOHnc1	EtOHnc2	Galnc1	Galnc2
13		YMR090W	YPDcl2	YPDcl3	EtOHcl1	EtOHcl2	Galcl1	YPDnc1	YPDnc2	YPDnc3	YPDnc4	EtOHnc1	EtOHnc2	Galnc1	Galnc2
13		YNR061C	YPDcl2	YPDcl3	EtOHcl1	EtOHcl2	Galcl1	YPDnc1	YPDnc2	YPDnc3	YPDnc4	EtOHnc1	EtOHnc2	Galnc1	Galnc2
13		YGR235C	YPDcl2	YPDcl3	EtOHcl1	EtOHcl2	Galcl1	YPDnc1	YPDnc2	YPDnc3	YPDnc4	EtOHnc1	EtOHnc2	Galnc1	Galnc2
13	NUP57	YGR119C	YPDcl2	YPDcl3	EtOHcl1	EtOHcl2	Galcl1	YPDnc1	YPDnc2	YPDnc3	YPDnc4	EtOHnc1	EtOHnc2	Galnc1	Galnc2
13		YER121W	YPDcl2	YPDcl3	EtOHcl1	EtOHcl2	Galcl1	YPDnc1	YPDnc2	YPDnc3	YPDnc4	EtOHnc1	EtOHnc2	Galnc1	Galnc2
13	YCK2	YNL154C	YPDcl2	YPDcl3	EtOHcl1	EtOHcl2	Galcl1	YPDnc1	YPDnc2	YPDnc3	YPDnc4	EtOHnc1	EtOHnc2	Galnc1	Galnc2
13	TRX2	YGR209C	YPDcl2	YPDcl3	EtOHcl1	EtOHcl2	Galcl1	YPDnc1	YPDnc2	YPDnc3	YPDnc4	EtOHnc1	EtOHnc2	Galnc1	Galnc2
13	PRX1	YBL064C	YPDcl2	YPDcl3	EtOHcl1	EtOHcl2	Galcl1	YPDnc1	YPDnc2	YPDnc3	YPDnc4	EtOHnc1	EtOHnc2	Galnc1	Galnc2
13		YDL012C	YPDcl2	YPDcl3	EtOHcl1	EtOHcl2	Galcl1	YPDnc1	YPDnc2	YPDnc3	YPDnc4	EtOHnc1	EtOHnc2	Galnc1	Galnc2
13	INH1	YDL181W	YPDcl2	YPDcl3	EtOHcl1	EtOHcl2	Galcl1	YPDnc1	YPDnc2	YPDnc3	YPDnc4	EtOHnc1	EtOHnc2	Galnc1	Galnc2
13	MAE1	YKL029C	YPDcl2	YPDcl3	EtOHcl1	EtOHcl2	Galcl1	YPDnc1	YPDnc2	YPDnc3	YPDnc4	EtOHnc1	EtOHnc2	Galnc1	Galnc2
13	ISR1	YPR106W	YPDcl2	YPDcl3	EtOHcl1	EtOHcl2	Galcl1	YPDnc1	YPDnc2	YPDnc3	YPDnc4	EtOHnc1	EtOHnc2	Galnc1	Galnc2
13	ATG9	YDL149W	YPDcl2	YPDcl3	EtOHcl1	EtOHcl2	Galcl1	YPDnc1	YPDnc2	YPDnc3	YPDnc4	EtOHnc1	EtOHnc2	Galnc1	Galnc2

13	NSE1	YLR007W	YPDcl2	YPDcl3	EtOHcl1	EtOHcl2	Galcl1	YPDnc1	YPDnc2	YPDnc3	YPDnc4	EtOHnc1	EtOHnc2	Galnc1	Galnc2
13	HGH1	YGR187C	YPDcl2	YPDcl3	EtOHcl1	EtOHcl2	Galcl1	YPDnc1	YPDnc2	YPDnc3	YPDnc4	EtOHnc1	EtOHnc2	Galnc1	Galnc2
13	ADK2	YER170W	YPDcl2	YPDcl3	EtOHcl1	EtOHcl2	Galcl1	YPDnc1	YPDnc2	YPDnc3	YPDnc4	EtOHnc1	EtOHnc2	Galnc1	Galnc2
13	KAR9	YPL269W	YPDcl2	YPDcl3	EtOHcl1	EtOHcl2	Galcl1	YPDnc1	YPDnc2	YPDnc3	YPDnc4	EtOHnc1	EtOHnc2	Galnc1	Galnc2
13	PRP22	YER013W	YPDcl2	YPDcl3	EtOHcl1	EtOHcl2	Galcl1	YPDnc1	YPDnc2	YPDnc3	YPDnc4	EtOHnc1	EtOHnc2	Galnc1	Galnc2
13	TRS120	YDR407C	YPDcl2	YPDcl3	EtOHcl1	EtOHcl2	Galcl1	YPDnc1	YPDnc2	YPDnc3	YPDnc4	EtOHnc1	EtOHnc2	Galnc1	Galnc2
13	PEF1	YGR058W	YPDcl2	YPDcl3	EtOHcl1	EtOHcl2	Galcl1	YPDnc1	YPDnc2	YPDnc3	YPDnc4	EtOHnc1	EtOHnc2	Galnc1	Galnc2
13	URM1	YIL008W	YPDcl2	YPDcl3	EtOHcl1	EtOHcl2	Galcl1	YPDnc1	YPDnc2	YPDnc3	YPDnc4	EtOHnc1	EtOHnc2	Galnc1	Galnc2
13	BIG1	YHR101C	YPDcl2	YPDcl3	EtOHcl1	EtOHcl2	Galcl1	YPDnc1	YPDnc2	YPDnc3	YPDnc4	EtOHnc1	EtOHnc2	Galnc1	Galnc2
13	SMB1	YER029C	YPDcl2	YPDcl3	EtOHcl1	EtOHcl2	Galcl1	YPDnc1	YPDnc2	YPDnc3	YPDnc4	EtOHnc1	EtOHnc2	Galnc1	Galnc2
13	RRT8	YOL048C	YPDcl2	YPDcl3	EtOHcl1	EtOHcl2	Galcl1	YPDnc1	YPDnc2	YPDnc3	YPDnc4	EtOHnc1	EtOHnc2	Galnc1	Galnc2
13	RRN7	YJL025W	YPDcl2	YPDcl3	EtOHcl1	EtOHcl2	Galcl1	YPDnc1	YPDnc2	YPDnc3	YPDnc4	EtOHnc1	EtOHnc2	Galnc1	Galnc2
13	RPN9	YDR427W	YPDcl2	YPDcl3	EtOHcl1	EtOHcl2	Galcl1	YPDnc1	YPDnc2	YPDnc3	YPDnc4	EtOHnc1	EtOHnc2	Galnc1	Galnc2
13	SKG3	YLR187W	YPDcl2	YPDcl3	EtOHcl1	EtOHcl2	Galcl1	YPDnc1	YPDnc2	YPDnc3	YPDnc4	EtOHnc1	EtOHnc2	Galnc1	Galnc2
13	FMP10	YER182W	YPDcl2	YPDcl3	EtOHcl1	EtOHcl2	Galcl1	YPDnc1	YPDnc2	YPDnc3	YPDnc4	EtOHnc1	EtOHnc2	Galnc1	Galnc2
13	PSO2	YMR137C	YPDcl2	YPDcl3	EtOHcl1	EtOHcl2	Galcl1	YPDnc1	YPDnc2	YPDnc3	YPDnc4	EtOHnc1	EtOHnc2	Galnc1	Galnc2
13	SPT20	YOL148C	YPDcl2	YPDcl3	EtOHcl1	EtOHcl2	Galcl1	YPDnc1	YPDnc2	YPDnc3	YPDnc4	EtOHnc1	EtOHnc2	Galnc1	Galnc2
13	MTF1	YMR228W	YPDcl2	YPDcl3	EtOHcl1	EtOHcl2	Galcl1	YPDnc1	YPDnc2	YPDnc3	YPDnc4	EtOHnc1	EtOHnc2	Galnc1	Galnc2
13		YBR232C	YPDcl2	YPDcl3	EtOHcl1	EtOHcl2	Galcl1	YPDnc1	YPDnc2	YPDnc3	YPDnc4	EtOHnc1	EtOHnc2	Galnc1	Galnc2
13	DFM1	YDR411C	YPDcl2	YPDcl3	EtOHcl1	EtOHcl2	Galcl1	YPDnc1	YPDnc2	YPDnc3	YPDnc4	EtOHnc1	EtOHnc2	Galnc1	Galnc2
13	NOP2	YNL061W	YPDcl2	YPDcl3	EtOHcl1	EtOHcl2	Galcl1	YPDnc1	YPDnc2	YPDnc3	YPDnc4	EtOHnc1	EtOHnc2	Galnc1	Galnc2
13	GIC2	YDR309C	YPDcl2	YPDcl3	EtOHcl1	EtOHcl2	Galcl1	YPDnc1	YPDnc2	YPDnc3	YPDnc4	EtOHnc1	EtOHnc2	Galnc1	Galnc2
13	RAD51	YER095W	YPDcl2	YPDcl3	EtOHcl1	EtOHcl2	Galcl1	YPDnc1	YPDnc2	YPDnc3	YPDnc4	EtOHnc1	EtOHnc2	Galnc1	Galnc2
13	TIF2	YJL138C	YPDcl2	YPDcl3	EtOHcl1	EtOHcl2	Galcl1	YPDnc1	YPDnc2	YPDnc3	YPDnc4	EtOHnc1	EtOHnc2	Galnc1	Galnc2
13	ECM9	YKR004C	YPDcl2	YPDcl3	EtOHcl1	EtOHcl2	Galcl1	YPDnc1	YPDnc2	YPDnc3	YPDnc4	EtOHnc1	EtOHnc2	Galnc1	Galnc2
13	MRPL8	YJL063C	YPDcl2	YPDcl3	EtOHcl1	EtOHcl2	Galcl1	YPDnc1	YPDnc2	YPDnc3	YPDnc4	EtOHnc1	EtOHnc2	Galnc1	Galnc2
13		YMR206W	YPDcl2	YPDcl3	EtOHcl1	EtOHcl2	Galcl1	YPDnc1	YPDnc2	YPDnc3	YPDnc4	EtOHnc1	EtOHnc2	Galnc1	Galnc2
13	RIM13	YMR154C	YPDcl2	YPDcl3	EtOHcl1	EtOHcl2	Galcl1	YPDnc1	YPDnc2	YPDnc3	YPDnc4	EtOHnc1	EtOHnc2	Galnc1	Galnc2
13	IES5	YER092W	YPDcl2	YPDcl3	EtOHcl1	EtOHcl2	Galcl1	YPDnc1	YPDnc2	YPDnc3	YPDnc4	EtOHnc1	EtOHnc2	Galnc1	Galnc2
13	FES1	YBR101C	YPDcl2	YPDcl3	EtOHcl1	EtOHcl2	Galcl1	YPDnc1	YPDnc2	YPDnc3	YPDnc4	EtOHnc1	EtOHnc2	Galnc1	Galnc2
13	PKP1	YIL042C	YPDcl2	YPDcl3	EtOHcl1	EtOHcl2	Galcl1	YPDnc1	YPDnc2	YPDnc3	YPDnc4	EtOHnc1	EtOHnc2	Galnc1	Galnc2
13	SIN3	YOL004W	YPDcl2	YPDcl3	EtOHcl1	EtOHcl2	Galcl1	YPDnc1	YPDnc2	YPDnc3	YPDnc4	EtOHnc1	EtOHnc2	Galnc1	Galnc2
13		YHR112C	YPDcl2	YPDcl3	EtOHcl1	EtOHcl2	Galcl1	YPDnc1	YPDnc2	YPDnc3	YPDnc4	EtOHnc1	EtOHnc2	Galnc1	Galnc2
13	CGR1	YGL029W	YPDcl2	YPDcl3	EtOHcl1	EtOHcl2	Galcl1	YPDnc1	YPDnc2	YPDnc3	YPDnc4	EtOHnc1	EtOHnc2	Galnc1	Galnc2

13	CBF1	YJR060W	YPDcl2	YPDcl3	EtOHcl1	EtOHcl2	Galcl1	YPDnc1	YPDnc2	YPDnc3	YPDnc4	EtOHnc1	EtOHnc2	Galnc1	Galnc2
13	GPI19	YDR437W	YPDcl2	YPDcl3	EtOHcl1	EtOHcl2	Galcl1	YPDnc1	YPDnc2	YPDnc3	YPDnc4	EtOHnc1	EtOHnc2	Galnc1	Galnc2
13	SPO11	YHL022C	YPDcl2	YPDcl3	EtOHcl1	EtOHcl2	Galcl1	YPDnc1	YPDnc2	YPDnc3	YPDnc4	EtOHnc1	EtOHnc2	Galnc1	Galnc2
13	SAE2	YGL175C	YPDcl2	YPDcl3	EtOHcl1	EtOHcl2	Galcl1	YPDnc1	YPDnc2	YPDnc3	YPDnc4	EtOHnc1	EtOHnc2	Galnc1	Galnc2
13	STT4	YLR305C	YPDcl2	YPDcl3	EtOHcl1	EtOHcl2	Galcl1	YPDnc1	YPDnc2	YPDnc3	YPDnc4	EtOHnc1	EtOHnc2	Galnc1	Galnc2
13		YOR343C	YPDcl2	YPDcl3	EtOHcl1	EtOHcl2	Galcl1	YPDnc1	YPDnc2	YPDnc3	YPDnc4	EtOHnc1	EtOHnc2	Galnc1	Galnc2
13	YHB1	YGR234W	YPDcl2	YPDcl3	EtOHcl1	EtOHcl2	Galcl1	YPDnc1	YPDnc2	YPDnc3	YPDnc4	EtOHnc1	EtOHnc2	Galnc1	Galnc2
13		YKL115C	YPDcl2	YPDcl3	EtOHcl1	EtOHcl2	Galcl1	YPDnc1	YPDnc2	YPDnc3	YPDnc4	EtOHnc1	EtOHnc2	Galnc1	Galnc2
13	TFA1	YKL028W	YPDcl2	YPDcl3	EtOHcl1	EtOHcl2	Galcl1	YPDnc1	YPDnc2	YPDnc3	YPDnc4	EtOHnc1	EtOHnc2	Galnc1	Galnc2
13	VPS4	YPR173C	YPDcl2	YPDcl3	EtOHcl1	EtOHcl2	Galcl1	YPDnc1	YPDnc2	YPDnc3	YPDnc4	EtOHnc1	EtOHnc2	Galnc1	Galnc2
13	SWC3	YAL011W	YPDcl2	YPDcl3	EtOHcl1	EtOHcl2	Galcl1	YPDnc1	YPDnc2	YPDnc3	YPDnc4	EtOHnc1	EtOHnc2	Galnc1	Galnc2
13	PRM10	YJL108C	YPDcl2	YPDcl3	EtOHcl1	EtOHcl2	Galcl1	YPDnc1	YPDnc2	YPDnc3	YPDnc4	EtOHnc1	EtOHnc2	Galnc1	Galnc2
13	VMA10	YHR039C-A	YPDcl2	YPDcl3	EtOHcl1	EtOHcl2	Galcl1	YPDnc1	YPDnc2	YPDnc3	YPDnc4	EtOHnc1	EtOHnc2	Galnc1	Galnc2
13	FAR10	YLR238W	YPDcl2	YPDcl3	EtOHcl1	EtOHcl2	Galcl1	YPDnc1	YPDnc2	YPDnc3	YPDnc4	EtOHnc1	EtOHnc2	Galnc1	Galnc2
13	YEH2	YLR020C	YPDcl2	YPDcl3	EtOHcl1	EtOHcl2	Galcl1	YPDnc1	YPDnc2	YPDnc3	YPDnc4	EtOHnc1	EtOHnc2	Galnc1	Galnc2
13		YEL020C	YPDcl2	YPDcl3	EtOHcl1	EtOHcl2	Galcl1	YPDnc1	YPDnc2	YPDnc3	YPDnc4	EtOHnc1	EtOHnc2	Galnc1	Galnc2
13	NPP2	YEL016C	YPDcl2	YPDcl3	EtOHcl1	EtOHcl2	Galcl1	YPDnc1	YPDnc2	YPDnc3	YPDnc4	EtOHnc1	EtOHnc2	Galnc1	Galnc2
13		YIL156W-B	YPDcl2	YPDcl3	EtOHcl1	EtOHcl2	Galcl1	YPDnc1	YPDnc2	YPDnc3	YPDnc4	EtOHnc1	EtOHnc2	Galnc1	Galnc2
13	DUF1	YOL087C	YPDcl2	YPDcl3	EtOHcl1	EtOHcl2	Galcl1	YPDnc1	YPDnc2	YPDnc3	YPDnc4	EtOHnc1	EtOHnc2	Galnc1	Galnc2
13	RSC30	YHR056C	YPDcl2	YPDcl3	EtOHcl1	EtOHcl2	Galcl1	YPDnc1	YPDnc2	YPDnc3	YPDnc4	EtOHnc1	EtOHnc2	Galnc1	Galnc2
13		YNL092W	YPDcl2	YPDcl3	EtOHcl1	EtOHcl2	Galcl1	YPDnc1	YPDnc2	YPDnc3	YPDnc4	EtOHnc1	EtOHnc2	Galnc1	Galnc2
13	RRD2	YPL152W	YPDcl2	YPDcl3	EtOHcl1	EtOHcl2	Galcl1	YPDnc1	YPDnc2	YPDnc3	YPDnc4	EtOHnc1	EtOHnc2	Galnc1	Galnc2
13	YMR1	YJR110W	YPDcl2	YPDcl3	EtOHcl1	EtOHcl2	Galcl1	YPDnc1	YPDnc2	YPDnc3	YPDnc4	EtOHnc1	EtOHnc2	Galnc1	Galnc2
13	MAG2	YLR427W	YPDcl2	YPDcl3	EtOHcl1	EtOHcl2	Galcl1	YPDnc1	YPDnc2	YPDnc3	YPDnc4	EtOHnc1	EtOHnc2	Galnc1	Galnc2
13	OLA1	YBR025C	YPDcl2	YPDcl3	EtOHcl1	EtOHcl2	Galcl1	YPDnc1	YPDnc2	YPDnc3	YPDnc4	EtOHnc1	EtOHnc2	Galnc1	Galnc2
13		YNL208W	YPDcl2	YPDcl3	EtOHcl1	EtOHcl2	Galcl1	YPDnc1	YPDnc2	YPDnc3	YPDnc4	EtOHnc1	EtOHnc2	Galnc1	Galnc2
13	RPS5	YJR123W	YPDcl2	YPDcl3	EtOHcl1	EtOHcl2	Galcl1	YPDnc1	YPDnc2	YPDnc3	YPDnc4	EtOHnc1	EtOHnc2	Galnc1	Galnc2
13	TEP1	YNL128W	YPDcl2	YPDcl3	EtOHcl1	EtOHcl2	Galcl1	YPDnc1	YPDnc2	YPDnc3	YPDnc4	EtOHnc1	EtOHnc2	Galnc1	Galnc2
13	MRP20	YDR405W	YPDcl2	YPDcl3	EtOHcl1	EtOHcl2	Galcl1	YPDnc1	YPDnc2	YPDnc3	YPDnc4	EtOHnc1	EtOHnc2	Galnc1	Galnc2
13	RAD54	YGL163C	YPDcl2	YPDcl3	EtOHcl1	EtOHcl2	Galcl1	YPDnc1	YPDnc2	YPDnc3	YPDnc4	EtOHnc1	EtOHnc2	Galnc1	Galnc2
13	RIM11	YMR139W	YPDcl2	YPDcl3	EtOHcl1	EtOHcl2	Galcl1	YPDnc1	YPDnc2	YPDnc3	YPDnc4	EtOHnc1	EtOHnc2	Galnc1	Galnc2
13	PBS2	YJL128C	YPDcl2	YPDcl3	EtOHcl1	EtOHcl2	Galcl1	YPDnc1	YPDnc2	YPDnc3	YPDnc4	EtOHnc1	EtOHnc2	Galnc1	Galnc2
13		YDR442W	YPDcl2	YPDcl3	EtOHcl1	EtOHcl2	Galcl1	YPDnc1	YPDnc2	YPDnc3	YPDnc4	EtOHnc1	EtOHnc2	Galnc1	Galnc2
13	TIM22	YDL217C	YPDcl2	YPDcl3	EtOHcl1	EtOHcl2	Galcl1	YPDnc1	YPDnc2	YPDnc3	YPDnc4	EtOHnc1	EtOHnc2	Galnc1	Galnc2

13	SEC62	YPL094C	YPDcl2	YPDcl3	EtOHcl1	EtOHcl2	Galcl1	YPDnc1	YPDnc2	YPDnc3	YPDnc4	EtOHnc1	EtOHnc2	Galnc1	Galnc2
13		YOL085C	YPDcl2	YPDcl3	EtOHcl1	EtOHcl2	Galcl1	YPDnc1	YPDnc2	YPDnc3	YPDnc4	EtOHnc1	EtOHnc2	Galnc1	Galnc2
13		YAL037C-A	YPDcl2	YPDcl3	EtOHcl1	EtOHcl2	Galcl1	YPDnc1	YPDnc2	YPDnc3	YPDnc4	EtOHnc1	EtOHnc2	Galnc1	Galnc2
13	EMI2	YDR516C	YPDcl2	YPDcl3	EtOHcl1	EtOHcl2	Galcl1	YPDnc1	YPDnc2	YPDnc3	YPDnc4	EtOHnc1	EtOHnc2	Galnc1	Galnc2
13	SSE1	YPL106C	YPDcl2	YPDcl3	EtOHcl1	EtOHcl2	Galcl1	YPDnc1	YPDnc2	YPDnc3	YPDnc4	EtOHnc1	EtOHnc2	Galnc1	Galnc2
13	HAP1	YLR256W	YPDcl2	YPDcl3	EtOHcl1	EtOHcl2	Galcl1	YPDnc1	YPDnc2	YPDnc3	YPDnc4	EtOHnc1	EtOHnc2	Galnc1	Galnc2
13	QCR2	YPR191W	YPDcl2	YPDcl3	EtOHcl1	EtOHcl2	Galcl1	YPDnc1	YPDnc2	YPDnc3	YPDnc4	EtOHnc1	EtOHnc2	Galnc1	Galnc2
13	MDM32	YOR147W	YPDcl2	YPDcl3	EtOHcl1	EtOHcl2	Galcl1	YPDnc1	YPDnc2	YPDnc3	YPDnc4	EtOHnc1	EtOHnc2	Galnc1	Galnc2
13	SAE3	YHR079C-A	YPDcl2	YPDcl3	EtOHcl1	EtOHcl2	Galcl1	YPDnc1	YPDnc2	YPDnc3	YPDnc4	EtOHnc1	EtOHnc2	Galnc1	Galnc2
13	RGD1	YBR260C	YPDcl2	YPDcl3	EtOHcl1	EtOHcl2	Galcl1	YPDnc1	YPDnc2	YPDnc3	YPDnc4	EtOHnc1	EtOHnc2	Galnc1	Galnc2
13		YOL079W	YPDcl2	YPDcl3	EtOHcl1	EtOHcl2	Galcl1	YPDnc1	YPDnc2	YPDnc3	YPDnc4	EtOHnc1	EtOHnc2	Galnc1	Galnc2
13	VPS33	YLR396C	YPDcl2	YPDcl3	EtOHcl1	EtOHcl2	Galcl1	YPDnc1	YPDnc2	YPDnc3	YPDnc4	EtOHnc1	EtOHnc2	Galnc1	Galnc2
13	RSM19	YNR037C	YPDcl2	YPDcl3	EtOHcl1	EtOHcl2	Galcl1	YPDnc1	YPDnc2	YPDnc3	YPDnc4	EtOHnc1	EtOHnc2	Galnc1	Galnc2
13		YDL109C	YPDcl2	YPDcl3	EtOHcl1	EtOHcl2	Galcl1	YPDnc1	YPDnc2	YPDnc3	YPDnc4	EtOHnc1	EtOHnc2	Galnc1	Galnc2
13		YCR043C	YPDcl2	YPDcl3	EtOHcl1	EtOHcl2	Galcl1	YPDnc1	YPDnc2	YPDnc3	YPDnc4	EtOHnc1	EtOHnc2	Galnc1	Galnc2
13	MDL2	YPL270W	YPDcl2	YPDcl3	EtOHcl1	EtOHcl2	Galcl1	YPDnc1	YPDnc2	YPDnc3	YPDnc4	EtOHnc1	EtOHnc2	Galnc1	Galnc2
13	YRO2	YBR054W	YPDcl2	YPDcl3	EtOHcl1	EtOHcl2	Galcl1	YPDnc1	YPDnc2	YPDnc3	YPDnc4	EtOHnc1	EtOHnc2	Galnc1	Galnc2
13	GCD10	YNL062C	YPDcl2	YPDcl3	EtOHcl1	EtOHcl2	Galcl1	YPDnc1	YPDnc2	YPDnc3	YPDnc4	EtOHnc1	EtOHnc2	Galnc1	Galnc2
13	IBD2	YNL164C	YPDcl2	YPDcl3	EtOHcl1	EtOHcl2	Galcl1	YPDnc1	YPDnc2	YPDnc3	YPDnc4	EtOHnc1	EtOHnc2	Galnc1	Galnc2
13	AIM34	YMR003W	YPDcl2	YPDcl3	EtOHcl1	EtOHcl2	Galcl1	YPDnc1	YPDnc2	YPDnc3	YPDnc4	EtOHnc1	EtOHnc2	Galnc1	Galnc2
13	URA5	YML106W	YPDcl2	YPDcl3	EtOHcl1	EtOHcl2	Galcl1	YPDnc1	YPDnc2	YPDnc3	YPDnc4	EtOHnc1	EtOHnc2	Galnc1	Galnc2
13		YIL029W-A	YPDcl2	YPDcl3	EtOHcl1	EtOHcl2	Galcl1	YPDnc1	YPDnc2	YPDnc3	YPDnc4	EtOHnc1	EtOHnc2	Galnc1	Galnc2
13	VPS29	YHR012W	YPDcl2	YPDcl3	EtOHcl1	EtOHcl2	Galcl1	YPDnc1	YPDnc2	YPDnc3	YPDnc4	EtOHnc1	EtOHnc2	Galnc1	Galnc2
13	ITR1	YDR497C	YPDcl2	YPDcl3	EtOHcl1	EtOHcl2	Galcl1	YPDnc1	YPDnc2	YPDnc3	YPDnc4	EtOHnc1	EtOHnc2	Galnc1	Galnc2
13	AFI1	YOR129C	YPDcl2	YPDcl3	EtOHcl1	EtOHcl2	Galcl1	YPDnc1	YPDnc2	YPDnc3	YPDnc4	EtOHnc1	EtOHnc2	Galnc1	Galnc2
13	RSM10	YDR041W	YPDcl2	YPDcl3	EtOHcl1	EtOHcl2	Galcl1	YPDnc1	YPDnc2	YPDnc3	YPDnc4	EtOHnc1	EtOHnc2	Galnc1	Galnc2
13	RPP1A	YDL081C	YPDcl2	YPDcl3	EtOHcl1	EtOHcl2	Galcl1	YPDnc1	YPDnc2	YPDnc3	YPDnc4	EtOHnc1	EtOHnc2	Galnc1	Galnc2
13		YBL100C	YPDcl2	YPDcl3	EtOHcl1	EtOHcl2	Galcl1	YPDnc1	YPDnc2	YPDnc3	YPDnc4	EtOHnc1	EtOHnc2	Galnc1	Galnc2
13	UBA2	YDR390C	YPDcl2	YPDcl3	EtOHcl1	EtOHcl2	Galcl1	YPDnc1	YPDnc2	YPDnc3	YPDnc4	EtOHnc1	EtOHnc2	Galnc1	Galnc2
13	COG6	YNL041C	YPDcl2	YPDcl3	EtOHcl1	EtOHcl2	Galcl1	YPDnc1	YPDnc2	YPDnc3	YPDnc4	EtOHnc1	EtOHnc2	Galnc1	Galnc2
13	TRM8	YDL201W	YPDcl2	YPDcl3	EtOHcl1	EtOHcl2	Galcl1	YPDnc1	YPDnc2	YPDnc3	YPDnc4	EtOHnc1	EtOHnc2	Galnc1	Galnc2
13	TAP42	YMR028W	YPDcl2	YPDcl3	EtOHcl1	EtOHcl2	Galcl1	YPDnc1	YPDnc2	YPDnc3	YPDnc4	EtOHnc1	EtOHnc2	Galnc1	Galnc2
13	YEA6	YEL006W	YPDcl2	YPDcl3	EtOHcl1	EtOHcl2	Galcl1	YPDnc1	YPDnc2	YPDnc3	YPDnc4	EtOHnc1	EtOHnc2	Galnc1	Galnc2
13	SUI2	YJR007W	YPDcl2	YPDcl3	EtOHcl1	EtOHcl2	Galcl1	YPDnc1	YPDnc2	YPDnc3	YPDnc4	EtOHnc1	EtOHnc2	Galnc1	Galnc2

13	KEX2	YNL238W	YPDcl2	YPDcl3	EtOHcl1	EtOHcl2	Galcl1	YPDnc1	YPDnc2	YPDnc3	YPDnc4	EtOHnc1	EtOHnc2	Galnc1	Galnc2
13		YBR053C	YPDcl2	YPDcl3	EtOHcl1	EtOHcl2	Galcl1	YPDnc1	YPDnc2	YPDnc3	YPDnc4	EtOHnc1	EtOHnc2	Galnc1	Galnc2
13	SOL2	YCR073W-/	YPDcl2	YPDcl3	EtOHcl1	EtOHcl2	Galcl1	YPDnc1	YPDnc2	YPDnc3	YPDnc4	EtOHnc1	EtOHnc2	Galnc1	Galnc2
13	APQ13	YJL075C	YPDcl2	YPDcl3	EtOHcl1	EtOHcl2	Galcl1	YPDnc1	YPDnc2	YPDnc3	YPDnc4	EtOHnc1	EtOHnc2	Galnc1	Galnc2
13		YGL109W	YPDcl2	YPDcl3	EtOHcl1	EtOHcl2	Galcl1	YPDnc1	YPDnc2	YPDnc3	YPDnc4	EtOHnc1	EtOHnc2	Galnc1	Galnc2
13	GTT2	YLL060C	YPDcl2	YPDcl3	EtOHcl1	EtOHcl2	Galcl1	YPDnc1	YPDnc2	YPDnc3	YPDnc4	EtOHnc1	EtOHnc2	Galnc1	Galnc2
13	SFI1	YLL003W	YPDcl2	YPDcl3	EtOHcl1	EtOHcl2	Galcl1	YPDnc1	YPDnc2	YPDnc3	YPDnc4	EtOHnc1	EtOHnc2	Galnc1	Galnc2
13		YBR103C-A	YPDcl2	YPDcl3	EtOHcl1	EtOHcl2	Galcl1	YPDnc1	YPDnc2	YPDnc3	YPDnc4	EtOHnc1	EtOHnc2	Galnc1	Galnc2
13	PGA3	YML125C	YPDcl2	YPDcl3	EtOHcl1	EtOHcl2	Galcl1	YPDnc1	YPDnc2	YPDnc3	YPDnc4	EtOHnc1	EtOHnc2	Galnc1	Galnc2
13		YOL057W	YPDcl2	YPDcl3	EtOHcl1	EtOHcl2	Galcl1	YPDnc1	YPDnc2	YPDnc3	YPDnc4	EtOHnc1	EtOHnc2	Galnc1	Galnc2
13	MET10	YFR030W	YPDcl2	YPDcl3	EtOHcl1	EtOHcl2	Galcl1	YPDnc1	YPDnc2	YPDnc3	YPDnc4	EtOHnc1	EtOHnc2	Galnc1	Galnc2
13	MCM10	YIL150C	YPDcl2	YPDcl3	EtOHcl1	EtOHcl2	Galcl1	YPDnc1	YPDnc2	YPDnc3	YPDnc4	EtOHnc1	EtOHnc2	Galnc1	Galnc2
13		YOL083C-A	YPDcl2	YPDcl3	EtOHcl1	EtOHcl2	Galcl1	YPDnc1	YPDnc2	YPDnc3	YPDnc4	EtOHnc1	EtOHnc2	Galnc1	Galnc2
13	PTC1	YDL006W	YPDcl2	YPDcl3	EtOHcl1	EtOHcl2	Galcl1	YPDnc1	YPDnc2	YPDnc3	YPDnc4	EtOHnc1	EtOHnc2	Galnc1	Galnc2
13	CSI1	YMR025W	YPDcl2	YPDcl3	EtOHcl1	EtOHcl2	Galcl1	YPDnc1	YPDnc2	YPDnc3	YPDnc4	EtOHnc1	EtOHnc2	Galnc1	Galnc2
13	HER1	YOR227W	YPDcl2	YPDcl3	EtOHcl1	EtOHcl2	Galcl1	YPDnc1	YPDnc2	YPDnc3	YPDnc4	EtOHnc1	EtOHnc2	Galnc1	Galnc2
13		YGR287C	YPDcl2	YPDcl3	EtOHcl1	EtOHcl2	Galcl1	YPDnc1	YPDnc2	YPDnc3	YPDnc4	EtOHnc1	EtOHnc2	Galnc1	Galnc2
13		YDL233W	YPDcl2	YPDcl3	EtOHcl1	EtOHcl2	Galcl1	YPDnc1	YPDnc2	YPDnc3	YPDnc4	EtOHnc1	EtOHnc2	Galnc1	Galnc2
13		YCL057C-A	YPDcl2	YPDcl3	EtOHcl1	EtOHcl2	Galcl1	YPDnc1	YPDnc2	YPDnc3	YPDnc4	EtOHnc1	EtOHnc2	Galnc1	Galnc2
13	PRE10	YOR362C	YPDcl2	YPDcl3	EtOHcl1	EtOHcl2	Galcl1	YPDnc1	YPDnc2	YPDnc3	YPDnc4	EtOHnc1	EtOHnc2	Galnc1	Galnc2
13	DSK2	YMR276W	YPDcl2	YPDcl3	EtOHcl1	EtOHcl2	Galcl1	YPDnc1	YPDnc2	YPDnc3	YPDnc4	EtOHnc1	EtOHnc2	Galnc1	Galnc2
13	REB1	YBR049C	YPDcl2	YPDcl3	EtOHcl1	EtOHcl2	Galcl1	YPDnc1	YPDnc2	YPDnc3	YPDnc4	EtOHnc1	EtOHnc2	Galnc1	Galnc2
13	RBD2	YPL246C	YPDcl2	YPDcl3	EtOHcl1	EtOHcl2	Galcl1	YPDnc1	YPDnc2	YPDnc3	YPDnc4	EtOHnc1	EtOHnc2	Galnc1	Galnc2
13	DLD2	YDL178W	YPDcl2	YPDcl3	EtOHcl1	EtOHcl2	Galcl1	YPDnc1	YPDnc2	YPDnc3	YPDnc4	EtOHnc1	EtOHnc2	Galnc1	Galnc2
13		YOR111W	YPDcl2	YPDcl3	EtOHcl1	EtOHcl2	Galcl1	YPDnc1	YPDnc2	YPDnc3	YPDnc4	EtOHnc1	EtOHnc2	Galnc1	Galnc2
13	NAR1	YNL240C	YPDcl2	YPDcl3	EtOHcl1	EtOHcl2	Galcl1	YPDnc1	YPDnc2	YPDnc3	YPDnc4	EtOHnc1	EtOHnc2	Galnc1	Galnc2
13	ATG23	YLR431C	YPDcl2	YPDcl3	EtOHcl1	EtOHcl2	Galcl1	YPDnc1	YPDnc2	YPDnc3	YPDnc4	EtOHnc1	EtOHnc2	Galnc1	Galnc2
13	RAM2	YKL019W	YPDcl2	YPDcl3	EtOHcl1	EtOHcl2	Galcl1	YPDnc1	YPDnc2	YPDnc3	YPDnc4	EtOHnc1	EtOHnc2	Galnc1	Galnc2
13		YGR168C	YPDcl2	YPDcl3	EtOHcl1	EtOHcl2	Galcl1	YPDnc1	YPDnc2	YPDnc3	YPDnc4	EtOHnc1	EtOHnc2	Galnc1	Galnc2
13	RPS25A	YGR027C	YPDcl2	YPDcl3	EtOHcl1	EtOHcl2	Galcl1	YPDnc1	YPDnc2	YPDnc3	YPDnc4	EtOHnc1	EtOHnc2	Galnc1	Galnc2
13	TRK1	YJL129C	YPDcl2	YPDcl3	EtOHcl1	EtOHcl2	Galcl1	YPDnc1	YPDnc2	YPDnc3	YPDnc4	EtOHnc1	EtOHnc2	Galnc1	Galnc2
13	BUD19	YJL188C	YPDcl2	YPDcl3	EtOHcl1	EtOHcl2	Galcl1	YPDnc1	YPDnc2	YPDnc3	YPDnc4	EtOHnc1	EtOHnc2	Galnc1	Galnc2
13	SGA1	YIL099W	YPDcl2	YPDcl3	EtOHcl1	EtOHcl2	Galcl1	YPDnc1	YPDnc2	YPDnc3	YPDnc4	EtOHnc1	EtOHnc2	Galnc1	Galnc2
13	MCM22	YJR135C	YPDcl2	YPDcl3	EtOHcl1	EtOHcl2	Galcl1	YPDnc1	YPDnc2	YPDnc3	YPDnc4	EtOHnc1	EtOHnc2	Galnc1	Galnc2

13	EXO84	YBR102C	YPDcl2	YPDcl3	EtOHcl1	EtOHcl2	Galcl1	YPDnc1	YPDnc2	YPDnc3	YPDnc4	EtOHnc1	EtOHnc2	Galnc1	Galnc2
13	GAT4	YIR013C	YPDcl2	YPDcl3	EtOHcl1	EtOHcl2	Galcl1	YPDnc1	YPDnc2	YPDnc3	YPDnc4	EtOHnc1	EtOHnc2	Galnc1	Galnc2
13	MKC7	YDR144C	YPDcl2	YPDcl3	EtOHcl1	EtOHcl2	Galcl1	YPDnc1	YPDnc2	YPDnc3	YPDnc4	EtOHnc1	EtOHnc2	Galnc1	Galnc2
13	LYS12	YIL094C	YPDcl2	YPDcl3	EtOHcl1	EtOHcl2	Galcl1	YPDnc1	YPDnc2	YPDnc3	YPDnc4	EtOHnc1	EtOHnc2	Galnc1	Galnc2
13		YIL002W-A	YPDcl2	YPDcl3	EtOHcl1	EtOHcl2	Galcl1	YPDnc1	YPDnc2	YPDnc3	YPDnc4	EtOHnc1	EtOHnc2	Galnc1	Galnc2
13	AIM2	YAL049C	YPDcl2	YPDcl3	EtOHcl1	EtOHcl2	Galcl1	YPDnc1	YPDnc2	YPDnc3	YPDnc4	EtOHnc1	EtOHnc2	Galnc1	Galnc2
13	CTF18	YMR078C	YPDcl2	YPDcl3	EtOHcl1	EtOHcl2	Galcl1	YPDnc1	YPDnc2	YPDnc3	YPDnc4	EtOHnc1	EtOHnc2	Galnc1	Galnc2
13		YJL077W-A	YPDcl2	YPDcl3	EtOHcl1	EtOHcl2	Galcl1	YPDnc1	YPDnc2	YPDnc3	YPDnc4	EtOHnc1	EtOHnc2	Galnc1	Galnc2
13	EXO70	YJL085W	YPDcl2	YPDcl3	EtOHcl1	EtOHcl2	Galcl1	YPDnc1	YPDnc2	YPDnc3	YPDnc4	EtOHnc1	EtOHnc2	Galnc1	Galnc2
13	STE6	YKL209C	YPDcl2	YPDcl3	EtOHcl1	EtOHcl2	Galcl1	YPDnc1	YPDnc2	YPDnc3	YPDnc4	EtOHnc1	EtOHnc2	Galnc1	Galnc2
13	PMU1	YKL128C	YPDcl2	YPDcl3	EtOHcl1	EtOHcl2	Galcl1	YPDnc1	YPDnc2	YPDnc3	YPDnc4	EtOHnc1	EtOHnc2	Galnc1	Galnc2
13		YMR010W	YPDcl2	YPDcl3	EtOHcl1	EtOHcl2	Galcl1	YPDnc1	YPDnc2	YPDnc3	YPDnc4	EtOHnc1	EtOHnc2	Galnc1	Galnc2
13		YCR023C	YPDcl2	YPDcl3	EtOHcl1	EtOHcl2	Galcl1	YPDnc1	YPDnc2	YPDnc3	YPDnc4	EtOHnc1	EtOHnc2	Galnc1	Galnc2
13	MRM1	YOR201C	YPDcl2	YPDcl3	EtOHcl1	EtOHcl2	Galcl1	YPDnc1	YPDnc2	YPDnc3	YPDnc4	EtOHnc1	EtOHnc2	Galnc1	Galnc2
13		YPR109W	YPDcl2	YPDcl3	EtOHcl1	EtOHcl2	Galcl1	YPDnc1	YPDnc2	YPDnc3	YPDnc4	EtOHnc1	EtOHnc2	Galnc1	Galnc2
13	NOT3	YIL038C	YPDcl2	YPDcl3	EtOHcl1	EtOHcl2	Galcl1	YPDnc1	YPDnc2	YPDnc3	YPDnc4	EtOHnc1	EtOHnc2	Galnc1	Galnc2
13		YML082W	YPDcl2	YPDcl3	EtOHcl1	EtOHcl2	Galcl1	YPDnc1	YPDnc2	YPDnc3	YPDnc4	EtOHnc1	EtOHnc2	Galnc1	Galnc2
13	AIM42	YOR286W	YPDcl2	YPDcl3	EtOHcl1	EtOHcl2	Galcl1	YPDnc1	YPDnc2	YPDnc3	YPDnc4	EtOHnc1	EtOHnc2	Galnc1	Galnc2
13		YOR218C	YPDcl2	YPDcl3	EtOHcl1	EtOHcl2	Galcl1	YPDnc1	YPDnc2	YPDnc3	YPDnc4	EtOHnc1	EtOHnc2	Galnc1	Galnc2
13		YNL010W	YPDcl2	YPDcl3	EtOHcl1	EtOHcl2	Galcl1	YPDnc1	YPDnc2	YPDnc3	YPDnc4	EtOHnc1	EtOHnc2	Galnc1	Galnc2
13		YJL070C	YPDcl2	YPDcl3	EtOHcl1	EtOHcl2	Galcl1	YPDnc1	YPDnc2	YPDnc3	YPDnc4	EtOHnc1	EtOHnc2	Galnc1	Galnc2
13	SKG1	YKR100C	YPDcl2	YPDcl3	EtOHcl1	EtOHcl2	Galcl1	YPDnc1	YPDnc2	YPDnc3	YPDnc4	EtOHnc1	EtOHnc2	Galnc1	Galnc2
13	RPS29A	YLR388W	YPDcl2	YPDcl3	EtOHcl1	EtOHcl2	Galcl1	YPDnc1	YPDnc2	YPDnc3	YPDnc4	EtOHnc1	EtOHnc2	Galnc1	Galnc2
13	TPM1	YNL079C	YPDcl2	YPDcl3	EtOHcl1	EtOHcl2	Galcl1	YPDnc1	YPDnc2	YPDnc3	YPDnc4	EtOHnc1	EtOHnc2	Galnc1	Galnc2
13	GIC1	YHR061C	YPDcl2	YPDcl3	EtOHcl1	EtOHcl2	Galcl1	YPDnc1	YPDnc2	YPDnc3	YPDnc4	EtOHnc1	EtOHnc2	Galnc1	Galnc2
13	GRE3	YHR104W	YPDcl2	YPDcl3	EtOHcl1	EtOHcl2	Galcl1	YPDnc1	YPDnc2	YPDnc3	YPDnc4	EtOHnc1	EtOHnc2	Galnc1	Galnc2
13	DRS1	YLL008W	YPDcl2	YPDcl3	EtOHcl1	EtOHcl2	Galcl1	YPDnc1	YPDnc2	YPDnc3	YPDnc4	EtOHnc1	EtOHnc2	Galnc1	Galnc2
13	CAR1	YPL111W	YPDcl2	YPDcl3	EtOHcl1	EtOHcl2	Galcl1	YPDnc1	YPDnc2	YPDnc3	YPDnc4	EtOHnc1	EtOHnc2	Galnc1	Galnc2
13	AMN1	YBR158W	YPDcl2	YPDcl3	EtOHcl1	EtOHcl2	Galcl1	YPDnc1	YPDnc2	YPDnc3	YPDnc4	EtOHnc1	EtOHnc2	Galnc1	Galnc2
13	RRT6	YGL146C	YPDcl2	YPDcl3	EtOHcl1	EtOHcl2	Galcl1	YPDnc1	YPDnc2	YPDnc3	YPDnc4	EtOHnc1	EtOHnc2	Galnc1	Galnc2
13	STV1	YMR054W	YPDcl2	YPDcl3	EtOHcl1	EtOHcl2	Galcl1	YPDnc1	YPDnc2	YPDnc3	YPDnc4	EtOHnc1	EtOHnc2	Galnc1	Galnc2
13	CHO1	YER026C	YPDcl2	YPDcl3	EtOHcl1	EtOHcl2	Galcl1	YPDnc1	YPDnc2	YPDnc3	YPDnc4	EtOHnc1	EtOHnc2	Galnc1	Galnc2
13	AKR1	YDR264C	YPDcl2	YPDcl3	EtOHcl1	EtOHcl2	Galcl1	YPDnc1	YPDnc2	YPDnc3	YPDnc4	EtOHnc1	EtOHnc2	Galnc1	Galnc2
13	PEP5	YMR231W	YPDcl2	YPDcl3	EtOHcl1	EtOHcl2	Galcl1	YPDnc1	YPDnc2	YPDnc3	YPDnc4	EtOHnc1	EtOHnc2	Galnc1	Galnc2

13	SCM3	YDL139C	YPDcl2	YPDcl3	EtOHcl1	EtOHcl2	Galcl1	YPDnc1	YPDnc2	YPDnc3	YPDnc4	EtOHnc1	EtOHnc2	Galnc1	Galnc2
13	YAP7	YOL028C	YPDcl2	YPDcl3	EtOHcl1	EtOHcl2	Galcl1	YPDnc1	YPDnc2	YPDnc3	YPDnc4	EtOHnc1	EtOHnc2	Galnc1	Galnc2
13	DBR1	YKL149C	YPDcl2	YPDcl3	EtOHcl1	EtOHcl2	Galcl1	YPDnc1	YPDnc2	YPDnc3	YPDnc4	EtOHnc1	EtOHnc2	Galnc1	Galnc2
13		YBR072C-A	YPDcl2	YPDcl3	EtOHcl1	EtOHcl2	Galcl1	YPDnc1	YPDnc2	YPDnc3	YPDnc4	EtOHnc1	EtOHnc2	Galnc1	Galnc2
13	DSE1	YER124C	YPDcl2	YPDcl3	EtOHcl1	EtOHcl2	Galcl1	YPDnc1	YPDnc2	YPDnc3	YPDnc4	EtOHnc1	EtOHnc2	Galnc1	Galnc2
13		YBR209W	YPDcl2	YPDcl3	EtOHcl1	EtOHcl2	Galcl1	YPDnc1	YPDnc2	YPDnc3	YPDnc4	EtOHnc1	EtOHnc2	Galnc1	Galnc2
13	PST1	YDR055W	YPDcl2	YPDcl3	EtOHcl1	EtOHcl2	Galcl1	YPDnc1	YPDnc2	YPDnc3	YPDnc4	EtOHnc1	EtOHnc2	Galnc1	Galnc2
13	SGS1	YMR190C	YPDcl2	YPDcl3	EtOHcl1	EtOHcl2	Galcl1	YPDnc1	YPDnc2	YPDnc3	YPDnc4	EtOHnc1	EtOHnc2	Galnc1	Galnc2
13	TMA17	YDL110C	YPDcl2	YPDcl3	EtOHcl1	EtOHcl2	Galcl1	YPDnc1	YPDnc2	YPDnc3	YPDnc4	EtOHnc1	EtOHnc2	Galnc1	Galnc2
13	GOR1	YNL274C	YPDcl2	YPDcl3	EtOHcl1	EtOHcl2	Galcl1	YPDnc1	YPDnc2	YPDnc3	YPDnc4	EtOHnc1	EtOHnc2	Galnc1	Galnc2
13		YMR027W	YPDcl2	YPDcl3	EtOHcl1	EtOHcl2	Galcl1	YPDnc1	YPDnc2	YPDnc3	YPDnc4	EtOHnc1	EtOHnc2	Galnc1	Galnc2
13		YGR051C	YPDcl2	YPDcl3	EtOHcl1	EtOHcl2	Galcl1	YPDnc1	YPDnc2	YPDnc3	YPDnc4	EtOHnc1	EtOHnc2	Galnc1	Galnc2
13	CPR7	YJR032W	YPDcl2	YPDcl3	EtOHcl1	EtOHcl2	Galcl1	YPDnc1	YPDnc2	YPDnc3	YPDnc4	EtOHnc1	EtOHnc2	Galnc1	Galnc2
13	AGE2	YIL044C	YPDcl2	YPDcl3	EtOHcl1	EtOHcl2	Galcl1	YPDnc1	YPDnc2	YPDnc3	YPDnc4	EtOHnc1	EtOHnc2	Galnc1	Galnc2
13		YJL211C	YPDcl2	YPDcl3	EtOHcl1	EtOHcl2	Galcl1	YPDnc1	YPDnc2	YPDnc3	YPDnc4	EtOHnc1	EtOHnc2	Galnc1	Galnc2
13	IPP1	YBR011C	YPDcl2	YPDcl3	EtOHcl1	EtOHcl2	Galcl1	YPDnc1	YPDnc2	YPDnc3	YPDnc4	EtOHnc1	EtOHnc2	Galnc1	Galnc2
13	RAD30	YDR419W	YPDcl2	YPDcl3	EtOHcl1	EtOHcl2	Galcl1	YPDnc1	YPDnc2	YPDnc3	YPDnc4	EtOHnc1	EtOHnc2	Galnc1	Galnc2
13	ALG1	YBR110W	YPDcl2	YPDcl3	EtOHcl1	EtOHcl2	Galcl1	YPDnc1	YPDnc2	YPDnc3	YPDnc4	EtOHnc1	EtOHnc2	Galnc1	Galnc2
13	TIR4	YOR009W	YPDcl2	YPDcl3	EtOHcl1	EtOHcl2	Galcl1	YPDnc1	YPDnc2	YPDnc3	YPDnc4	EtOHnc1	EtOHnc2	Galnc1	Galnc2
13		YEL028W	YPDcl2	YPDcl3	EtOHcl1	EtOHcl2	Galcl1	YPDnc1	YPDnc2	YPDnc3	YPDnc4	EtOHnc1	EtOHnc2	Galnc1	Galnc2
13	NNF2	YGR089W	YPDcl2	YPDcl3	EtOHcl1	EtOHcl2	Galcl1	YPDnc1	YPDnc2	YPDnc3	YPDnc4	EtOHnc1	EtOHnc2	Galnc1	Galnc2
13	IRC20	YLR247C	YPDcl2	YPDcl3	EtOHcl1	EtOHcl2	Galcl1	YPDnc1	YPDnc2	YPDnc3	YPDnc4	EtOHnc1	EtOHnc2	Galnc1	Galnc2
13		YDR266C	YPDcl2	YPDcl3	EtOHcl1	EtOHcl2	Galcl1	YPDnc1	YPDnc2	YPDnc3	YPDnc4	EtOHnc1	EtOHnc2	Galnc1	Galnc2
13	RKM1	YPL208W	YPDcl2	YPDcl3	EtOHcl1	EtOHcl2	Galcl1	YPDnc1	YPDnc2	YPDnc3	YPDnc4	EtOHnc1	EtOHnc2	Galnc1	Galnc2
13	HMG2	YLR450W	YPDcl2	YPDcl3	EtOHcl1	EtOHcl2	Galcl1	YPDnc1	YPDnc2	YPDnc3	YPDnc4	EtOHnc1	EtOHnc2	Galnc1	Galnc2
13	HSH49	YOR319W	YPDcl2	YPDcl3	EtOHcl1	EtOHcl2	Galcl1	YPDnc1	YPDnc2	YPDnc3	YPDnc4	EtOHnc1	EtOHnc2	Galnc1	Galnc2
13	GAL80	YML051W	YPDcl2	YPDcl3	EtOHcl1	EtOHcl2	Galcl1	YPDnc1	YPDnc2	YPDnc3	YPDnc4	EtOHnc1	EtOHnc2	Galnc1	Galnc2
13		YGL117W	YPDcl2	YPDcl3	EtOHcl1	EtOHcl2	Galcl1	YPDnc1	YPDnc2	YPDnc3	YPDnc4	EtOHnc1	EtOHnc2	Galnc1	Galnc2
13	AUA1	YFL010W-A	YPDcl2	YPDcl3	EtOHcl1	EtOHcl2	Galcl1	YPDnc1	YPDnc2	YPDnc3	YPDnc4	EtOHnc1	EtOHnc2	Galnc1	Galnc2
13	PSF2	YJL072C	YPDcl2	YPDcl3	EtOHcl1	EtOHcl2	Galcl1	YPDnc1	YPDnc2	YPDnc3	YPDnc4	EtOHnc1	EtOHnc2	Galnc1	Galnc2
13	LRP1	YHR081W	YPDcl2	YPDcl3	EtOHcl1	EtOHcl2	Galcl1	YPDnc1	YPDnc2	YPDnc3	YPDnc4	EtOHnc1	EtOHnc2	Galnc1	Galnc2
13	CKA2	YOR061W	YPDcl2	YPDcl3	EtOHcl1	EtOHcl2	Galcl1	YPDnc1	YPDnc2	YPDnc3	YPDnc4	EtOHnc1	EtOHnc2	Galnc1	Galnc2
13		YDR090C	YPDcl2	YPDcl3	EtOHcl1	EtOHcl2	Galcl1	YPDnc1	YPDnc2	YPDnc3	YPDnc4	EtOHnc1	EtOHnc2	Galnc1	Galnc2
13	CLN2	YPL256C	YPDcl2	YPDcl3	EtOHcl1	EtOHcl2	Galcl1	YPDnc1	YPDnc2	YPDnc3	YPDnc4	EtOHnc1	EtOHnc2	Galnc1	Galnc2

13	GYP1	YOR070C	YPDcl2	YPDcl3	EtOHcl1	EtOHcl2	Galcl1	YPDnc1	YPDnc2	YPDnc3	YPDnc4	EtOHnc1	EtOHnc2	Galnc1	Galnc2
13		YNL024C	YPDcl2	YPDcl3	EtOHcl1	EtOHcl2	Galcl1	YPDnc1	YPDnc2	YPDnc3	YPDnc4	EtOHnc1	EtOHnc2	Galnc1	Galnc2
13	ENA1	YDR040C	YPDcl2	YPDcl3	EtOHcl1	EtOHcl2	Galcl1	YPDnc1	YPDnc2	YPDnc3	YPDnc4	EtOHnc1	EtOHnc2	Galnc1	Galnc2
13	AFG2	YLR397C	YPDcl2	YPDcl3	EtOHcl1	EtOHcl2	Galcl1	YPDnc1	YPDnc2	YPDnc3	YPDnc4	EtOHnc1	EtOHnc2	Galnc1	Galnc2
13	SPC105	YGL093W	YPDcl2	YPDcl3	EtOHcl1	EtOHcl2	Galcl1	YPDnc1	YPDnc2	YPDnc3	YPDnc4	EtOHnc1	EtOHnc2	Galnc1	Galnc2
13		YHL017W	YPDcl2	YPDcl3	EtOHcl1	EtOHcl2	Galcl1	YPDnc1	YPDnc2	YPDnc3	YPDnc4	EtOHnc1	EtOHnc2	Galnc1	Galnc2
13	DED1	YOR204W	YPDcl2	YPDcl3	EtOHcl1	EtOHcl2	Galcl1	YPDnc1	YPDnc2	YPDnc3	YPDnc4	EtOHnc1	EtOHnc2	Galnc1	Galnc2
13		YMR046W-	YPDcl2	YPDcl3	EtOHcl1	EtOHcl2	Galcl1	YPDnc1	YPDnc2	YPDnc3	YPDnc4	EtOHnc1	EtOHnc2	Galnc1	Galnc2
13	GPD2	YOL059W	YPDcl2	YPDcl3	EtOHcl1	EtOHcl2	Galcl1	YPDnc1	YPDnc2	YPDnc3	YPDnc4	EtOHnc1	EtOHnc2	Galnc1	Galnc2
13	CLP1	YOR250C	YPDcl2	YPDcl3	EtOHcl1	EtOHcl2	Galcl1	YPDnc1	YPDnc2	YPDnc3	YPDnc4	EtOHnc1	EtOHnc2	Galnc1	Galnc2
13	CLD1	YGR110W	YPDcl2	YPDcl3	EtOHcl1	EtOHcl2	Galcl1	YPDnc1	YPDnc2	YPDnc3	YPDnc4	EtOHnc1	EtOHnc2	Galnc1	Galnc2
13	MUP3	YHL036W	YPDcl2	YPDcl3	EtOHcl1	EtOHcl2	Galcl1	YPDnc1	YPDnc2	YPDnc3	YPDnc4	EtOHnc1	EtOHnc2	Galnc1	Galnc2
13	SEY1	YOR165W	YPDcl2	YPDcl3	EtOHcl1	EtOHcl2	Galcl1	YPDnc1	YPDnc2	YPDnc3	YPDnc4	EtOHnc1	EtOHnc2	Galnc1	Galnc2
13	EMC2	YJR088C	YPDcl2	YPDcl3	EtOHcl1	EtOHcl2	Galcl1	YPDnc1	YPDnc2	YPDnc3	YPDnc4	EtOHnc1	EtOHnc2	Galnc1	Galnc2
13	RNA1	YMR235C	YPDcl2	YPDcl3	EtOHcl1	EtOHcl2	Galcl1	YPDnc1	YPDnc2	YPDnc3	YPDnc4	EtOHnc1	EtOHnc2	Galnc1	Galnc2
13	LSG1	YGL099W	YPDcl2	YPDcl3	EtOHcl1	EtOHcl2	Galcl1	YPDnc1	YPDnc2	YPDnc3	YPDnc4	EtOHnc1	EtOHnc2	Galnc1	Galnc2
13		YJR039W	YPDcl2	YPDcl3	EtOHcl1	EtOHcl2	Galcl1	YPDnc1	YPDnc2	YPDnc3	YPDnc4	EtOHnc1	EtOHnc2	Galnc1	Galnc2
13	RPA34	YJL148W	YPDcl2	YPDcl3	EtOHcl1	EtOHcl2	Galcl1	YPDnc1	YPDnc2	YPDnc3	YPDnc4	EtOHnc1	EtOHnc2	Galnc1	Galnc2
13	UBX4	YMR067C	YPDcl2	YPDcl3	EtOHcl1	EtOHcl2	Galcl1	YPDnc1	YPDnc2	YPDnc3	YPDnc4	EtOHnc1	EtOHnc2	Galnc1	Galnc2
13	ALO1	YML086C	YPDcl2	YPDcl3	EtOHcl1	EtOHcl2	Galcl1	YPDnc1	YPDnc2	YPDnc3	YPDnc4	EtOHnc1	EtOHnc2	Galnc1	Galnc2
13	MLH1	YMR167W	YPDcl2	YPDcl3	EtOHcl1	EtOHcl2	Galcl1	YPDnc1	YPDnc2	YPDnc3	YPDnc4	EtOHnc1	EtOHnc2	Galnc1	Galnc2
13	DAD1	YDR016C	YPDcl2	YPDcl3	EtOHcl1	EtOHcl2	Galcl1	YPDnc1	YPDnc2	YPDnc3	YPDnc4	EtOHnc1	EtOHnc2	Galnc1	Galnc2
13	PFY1	YOR122C	YPDcl2	YPDcl3	EtOHcl1	EtOHcl2	Galcl1	YPDnc1	YPDnc2	YPDnc3	YPDnc4	EtOHnc1	EtOHnc2	Galnc1	Galnc2
13	SDP1	YIL113W	YPDcl2	YPDcl3	EtOHcl1	EtOHcl2	Galcl1	YPDnc1	YPDnc2	YPDnc3	YPDnc4	EtOHnc1	EtOHnc2	Galnc1	Galnc2
13		YNL234W	YPDcl2	YPDcl3	EtOHcl1	EtOHcl2	Galcl1	YPDnc1	YPDnc2	YPDnc3	YPDnc4	EtOHnc1	EtOHnc2	Galnc1	Galnc2
13	PTR2	YKR093W	YPDcl2	YPDcl3	EtOHcl1	EtOHcl2	Galcl1	YPDnc1	YPDnc2	YPDnc3	YPDnc4	EtOHnc1	EtOHnc2	Galnc1	Galnc2
13	RSA4	YCR072C	YPDcl2	YPDcl3	EtOHcl1	EtOHcl2	Galcl1	YPDnc1	YPDnc2	YPDnc3	YPDnc4	EtOHnc1	EtOHnc2	Galnc1	Galnc2
13	ERD1	YDR414C	YPDcl2	YPDcl3	EtOHcl1	EtOHcl2	Galcl1	YPDnc1	YPDnc2	YPDnc3	YPDnc4	EtOHnc1	EtOHnc2	Galnc1	Galnc2
13	TMN3	YER113C	YPDcl2	YPDcl3	EtOHcl1	EtOHcl2	Galcl1	YPDnc1	YPDnc2	YPDnc3	YPDnc4	EtOHnc1	EtOHnc2	Galnc1	Galnc2
13	RDR1	YOR380W	YPDcl2	YPDcl3	EtOHcl1	EtOHcl2	Galcl1	YPDnc1	YPDnc2	YPDnc3	YPDnc4	EtOHnc1	EtOHnc2	Galnc1	Galnc2
13	MSC7	YHR039C	YPDcl2	YPDcl3	EtOHcl1	EtOHcl2	Galcl1	YPDnc1	YPDnc2	YPDnc3	YPDnc4	EtOHnc1	EtOHnc2	Galnc1	Galnc2
13	OTU1	YFL044C	YPDcl2	YPDcl3	EtOHcl1	EtOHcl2	Galcl1	YPDnc1	YPDnc2	YPDnc3	YPDnc4	EtOHnc1	EtOHnc2	Galnc1	Galnc2
13	FAA3	YIL009W	YPDcl2	YPDcl3	EtOHcl1	EtOHcl2	Galcl1	YPDnc1	YPDnc2	YPDnc3	YPDnc4	EtOHnc1	EtOHnc2	Galnc1	Galnc2
13	ISW1	YBR245C	YPDcl2	YPDcl3	EtOHcl1	EtOHcl2	Galcl1	YPDnc1	YPDnc2	YPDnc3	YPDnc4	EtOHnc1	EtOHnc2	Galnc1	Galnc2

13		YNL046W	YPDcl2	YPDcl3	EtOHcl1	EtOHcl2	Galcl1	YPDnc1	YPDnc2	YPDnc3	YPDnc4	EtOHnc1	EtOHnc2	Galnc1	Galnc2
13	MCM21	YDR318W	YPDcl2	YPDcl3	EtOHcl1	EtOHcl2	Galcl1	YPDnc1	YPDnc2	YPDnc3	YPDnc4	EtOHnc1	EtOHnc2	Galnc1	Galnc2
13	DIP5	YPL265W	YPDcl2	YPDcl3	EtOHcl1	EtOHcl2	Galcl1	YPDnc1	YPDnc2	YPDnc3	YPDnc4	EtOHnc1	EtOHnc2	Galnc1	Galnc2
13		YGL152C	YPDcl2	YPDcl3	EtOHcl1	EtOHcl2	Galcl1	YPDnc1	YPDnc2	YPDnc3	YPDnc4	EtOHnc1	EtOHnc2	Galnc1	Galnc2
13	YNG2	YHR090C	YPDcl2	YPDcl3	EtOHcl1	EtOHcl2	Galcl1	YPDnc1	YPDnc2	YPDnc3	YPDnc4	EtOHnc1	EtOHnc2	Galnc1	Galnc2
13		YLR184W	YPDcl2	YPDcl3	EtOHcl1	EtOHcl2	Galcl1	YPDnc1	YPDnc2	YPDnc3	YPDnc4	EtOHnc1	EtOHnc2	Galnc1	Galnc2
13	JID1	YPR061C	YPDcl2	YPDcl3	EtOHcl1	EtOHcl2	Galcl1	YPDnc1	YPDnc2	YPDnc3	YPDnc4	EtOHnc1	EtOHnc2	Galnc1	Galnc2
13	MSH2	YOL090W	YPDcl2	YPDcl3	EtOHcl1	EtOHcl2	Galcl1	YPDnc1	YPDnc2	YPDnc3	YPDnc4	EtOHnc1	EtOHnc2	Galnc1	Galnc2
13	TYS1	YGR185C	YPDcl2	YPDcl3	EtOHcl1	EtOHcl2	Galcl1	YPDnc1	YPDnc2	YPDnc3	YPDnc4	EtOHnc1	EtOHnc2	Galnc1	Galnc2
13		YIL020C-A	YPDcl2	YPDcl3	EtOHcl1	EtOHcl2	Galcl1	YPDnc1	YPDnc2	YPDnc3	YPDnc4	EtOHnc1	EtOHnc2	Galnc1	Galnc2
13	AIM32	YML050W	YPDcl2	YPDcl3	EtOHcl1	EtOHcl2	Galcl1	YPDnc1	YPDnc2	YPDnc3	YPDnc4	EtOHnc1	EtOHnc2	Galnc1	Galnc2
13	VPS65	YLR322W	YPDcl2	YPDcl3	EtOHcl1	EtOHcl2	Galcl1	YPDnc1	YPDnc2	YPDnc3	YPDnc4	EtOHnc1	EtOHnc2	Galnc1	Galnc2
13		YDR034W-	YPDcl2	YPDcl3	EtOHcl1	EtOHcl2	Galcl1	YPDnc1	YPDnc2	YPDnc3	YPDnc4	EtOHnc1	EtOHnc2	Galnc1	Galnc2
13	NIS1	YNL078W	YPDcl2	YPDcl3	EtOHcl1	EtOHcl2	Galcl1	YPDnc1	YPDnc2	YPDnc3	YPDnc4	EtOHnc1	EtOHnc2	Galnc1	Galnc2
13		YLR349W	YPDcl2	YPDcl3	EtOHcl1	EtOHcl2	Galcl1	YPDnc1	YPDnc2	YPDnc3	YPDnc4	EtOHnc1	EtOHnc2	Galnc1	Galnc2
13		YKR018C	YPDcl2	YPDcl3	EtOHcl1	EtOHcl2	Galcl1	YPDnc1	YPDnc2	YPDnc3	YPDnc4	EtOHnc1	EtOHnc2	Galnc1	Galnc2
13	COS3	YML132W	YPDcl2	YPDcl3	EtOHcl1	EtOHcl2	Galcl1	YPDnc1	YPDnc2	YPDnc3	YPDnc4	EtOHnc1	EtOHnc2	Galnc1	Galnc2
13	YSC84	YHR016C	YPDcl2	YPDcl3	EtOHcl1	EtOHcl2	Galcl1	YPDnc1	YPDnc2	YPDnc3	YPDnc4	EtOHnc1	EtOHnc2	Galnc1	Galnc2
13	VTC2	YFL004W	YPDcl2	YPDcl3	EtOHcl1	EtOHcl2	Galcl1	YPDnc1	YPDnc2	YPDnc3	YPDnc4	EtOHnc1	EtOHnc2	Galnc1	Galnc2
13	RFC3	YNL290W	YPDcl2	YPDcl3	EtOHcl1	EtOHcl2	Galcl1	YPDnc1	YPDnc2	YPDnc3	YPDnc4	EtOHnc1	EtOHnc2	Galnc1	Galnc2
13	HHT1	YBR010W	YPDcl2	YPDcl3	EtOHcl1	EtOHcl2	Galcl1	YPDnc1	YPDnc2	YPDnc3	YPDnc4	EtOHnc1	EtOHnc2	Galnc1	Galnc2
13	LCL3	YGL085W	YPDcl2	YPDcl3	EtOHcl1	EtOHcl2	Galcl1	YPDnc1	YPDnc2	YPDnc3	YPDnc4	EtOHnc1	EtOHnc2	Galnc1	Galnc2
13	ENT2	YLR206W	YPDcl2	YPDcl3	EtOHcl1	EtOHcl2	Galcl1	YPDnc1	YPDnc2	YPDnc3	YPDnc4	EtOHnc1	EtOHnc2	Galnc1	Galnc2
13	TGL4	YKR089C	YPDcl2	YPDcl3	EtOHcl1	EtOHcl2	Galcl1	YPDnc1	YPDnc2	YPDnc3	YPDnc4	EtOHnc1	EtOHnc2	Galnc1	Galnc2
13		YGL088W	YPDcl2	YPDcl3	EtOHcl1	EtOHcl2	Galcl1	YPDnc1	YPDnc2	YPDnc3	YPDnc4	EtOHnc1	EtOHnc2	Galnc1	Galnc2
13	SNF11	YDR073W	YPDcl2	YPDcl3	EtOHcl1	EtOHcl2	Galcl1	YPDnc1	YPDnc2	YPDnc3	YPDnc4	EtOHnc1	EtOHnc2	Galnc1	Galnc2
13	DCI1	YOR180C	YPDcl2	YPDcl3	EtOHcl1	EtOHcl2	Galcl1	YPDnc1	YPDnc2	YPDnc3	YPDnc4	EtOHnc1	EtOHnc2	Galnc1	Galnc2
13	FLC3	YGL139W	YPDcl2	YPDcl3	EtOHcl1	EtOHcl2	Galcl1	YPDnc1	YPDnc2	YPDnc3	YPDnc4	EtOHnc1	EtOHnc2	Galnc1	Galnc2
13		YAL044W-A	YPDcl2	YPDcl3	EtOHcl1	EtOHcl2	Galcl1	YPDnc1	YPDnc2	YPDnc3	YPDnc4	EtOHnc1	EtOHnc2	Galnc1	Galnc2
13		YGL140C	YPDcl2	YPDcl3	EtOHcl1	EtOHcl2	Galcl1	YPDnc1	YPDnc2	YPDnc3	YPDnc4	EtOHnc1	EtOHnc2	Galnc1	Galnc2
13	RNR4	YGR180C	YPDcl2	YPDcl3	EtOHcl1	EtOHcl2	Galcl1	YPDnc1	YPDnc2	YPDnc3	YPDnc4	EtOHnc1	EtOHnc2	Galnc1	Galnc2
13	CTF19	YPL018W	YPDcl2	YPDcl3	EtOHcl1	EtOHcl2	Galcl1	YPDnc1	YPDnc2	YPDnc3	YPDnc4	EtOHnc1	EtOHnc2	Galnc1	Galnc2
13		YPL229W	YPDcl2	YPDcl3	EtOHcl1	EtOHcl2	Galcl1	YPDnc1	YPDnc2	YPDnc3	YPDnc4	EtOHnc1	EtOHnc2	Galnc1	Galnc2
13	PET100	YDR079W	YPDcl2	YPDcl3	EtOHcl1	EtOHcl2	Galcl1	YPDnc1	YPDnc2	YPDnc3	YPDnc4	EtOHnc1	EtOHnc2	Galnc1	Galnc2

13	SHO1	YER118C	YPDcl2	YPDcl3	EtOHcl1	EtOHcl2	Galcl1	YPDnc1	YPDnc2	YPDnc3	YPDnc4	EtOHnc1	EtOHnc2	Galnc1	Galnc2
13	UBP13	YBL067C	YPDcl2	YPDcl3	EtOHcl1	EtOHcl2	Galcl1	YPDnc1	YPDnc2	YPDnc3	YPDnc4	EtOHnc1	EtOHnc2	Galnc1	Galnc2
13	RPL42A	YNL162W	YPDcl2	YPDcl3	EtOHcl1	EtOHcl2	Galcl1	YPDnc1	YPDnc2	YPDnc3	YPDnc4	EtOHnc1	EtOHnc2	Galnc1	Galnc2
13	CMR2	YOR093C	YPDcl2	YPDcl3	EtOHcl1	EtOHcl2	Galcl1	YPDnc1	YPDnc2	YPDnc3	YPDnc4	EtOHnc1	EtOHnc2	Galnc1	Galnc2
13	RPC25	YKL144C	YPDcl2	YPDcl3	EtOHcl1	EtOHcl2	Galcl1	YPDnc1	YPDnc2	YPDnc3	YPDnc4	EtOHnc1	EtOHnc2	Galnc1	Galnc2
13	YCT1	YLL055W	YPDcl2	YPDcl3	EtOHcl1	EtOHcl2	Galcl1	YPDnc1	YPDnc2	YPDnc3	YPDnc4	EtOHnc1	EtOHnc2	Galnc1	Galnc2
13	AEP1	YMR064W	YPDcl2	YPDcl3	EtOHcl1	EtOHcl2	Galcl1	YPDnc1	YPDnc2	YPDnc3	YPDnc4	EtOHnc1	EtOHnc2	Galnc1	Galnc2
13	TIF11	YMR260C	YPDcl2	YPDcl3	EtOHcl1	EtOHcl2	Galcl1	YPDnc1	YPDnc2	YPDnc3	YPDnc4	EtOHnc1	EtOHnc2	Galnc1	Galnc2
13		YOR060C	YPDcl2	YPDcl3	EtOHcl1	EtOHcl2	Galcl1	YPDnc1	YPDnc2	YPDnc3	YPDnc4	EtOHnc1	EtOHnc2	Galnc1	Galnc2
13		YNL024C-A	YPDcl2	YPDcl3	EtOHcl1	EtOHcl2	Galcl1	YPDnc1	YPDnc2	YPDnc3	YPDnc4	EtOHnc1	EtOHnc2	Galnc1	Galnc2
13		YJR015W	YPDcl2	YPDcl3	EtOHcl1	EtOHcl2	Galcl1	YPDnc1	YPDnc2	YPDnc3	YPDnc4	EtOHnc1	EtOHnc2	Galnc1	Galnc2
13	CTF8	YHR191C	YPDcl2	YPDcl3	EtOHcl1	EtOHcl2	Galcl1	YPDnc1	YPDnc2	YPDnc3	YPDnc4	EtOHnc1	EtOHnc2	Galnc1	Galnc2
13	UPS3	YDR185C	YPDcl2	YPDcl3	EtOHcl1	EtOHcl2	Galcl1	YPDnc1	YPDnc2	YPDnc3	YPDnc4	EtOHnc1	EtOHnc2	Galnc1	Galnc2
13	UGA3	YDL170W	YPDcl2	YPDcl3	EtOHcl1	EtOHcl2	Galcl1	YPDnc1	YPDnc2	YPDnc3	YPDnc4	EtOHnc1	EtOHnc2	Galnc1	Galnc2
13		YEL073C	YPDcl2	YPDcl3	EtOHcl1	EtOHcl2	Galcl1	YPDnc1	YPDnc2	YPDnc3	YPDnc4	EtOHnc1	EtOHnc2	Galnc1	Galnc2
13	TCB3	YML072C	YPDcl2	YPDcl3	EtOHcl1	EtOHcl2	Galcl1	YPDnc1	YPDnc2	YPDnc3	YPDnc4	EtOHnc1	EtOHnc2	Galnc1	Galnc2
13	CAX4	YGR036C	YPDcl2	YPDcl3	EtOHcl1	EtOHcl2	Galcl1	YPDnc1	YPDnc2	YPDnc3	YPDnc4	EtOHnc1	EtOHnc2	Galnc1	Galnc2
13	SSH1	YBR283C	YPDcl2	YPDcl3	EtOHcl1	EtOHcl2	Galcl1	YPDnc1	YPDnc2	YPDnc3	YPDnc4	EtOHnc1	EtOHnc2	Galnc1	Galnc2
13	MTC3	YGL226W	YPDcl2	YPDcl3	EtOHcl1	EtOHcl2	Galcl1	YPDnc1	YPDnc2	YPDnc3	YPDnc4	EtOHnc1	EtOHnc2	Galnc1	Galnc2
13		YPR096C	YPDcl2	YPDcl3	EtOHcl1	EtOHcl2	Galcl1	YPDnc1	YPDnc2	YPDnc3	YPDnc4	EtOHnc1	EtOHnc2	Galnc1	Galnc2
13	FYV8	YGR196C	YPDcl2	YPDcl3	EtOHcl1	EtOHcl2	Galcl1	YPDnc1	YPDnc2	YPDnc3	YPDnc4	EtOHnc1	EtOHnc2	Galnc1	Galnc2
13	RPS18A	YDR450W	YPDcl2	YPDcl3	EtOHcl1	EtOHcl2	Galcl1	YPDnc1	YPDnc2	YPDnc3	YPDnc4	EtOHnc1	EtOHnc2	Galnc1	Galnc2
13	RPD3	YNL330C	YPDcl2	YPDcl3	EtOHcl1	EtOHcl2	Galcl1	YPDnc1	YPDnc2	YPDnc3	YPDnc4	EtOHnc1	EtOHnc2	Galnc1	Galnc2
13	PTH1	YHR189W	YPDcl2	YPDcl3	EtOHcl1	EtOHcl2	Galcl1	YPDnc1	YPDnc2	YPDnc3	YPDnc4	EtOHnc1	EtOHnc2	Galnc1	Galnc2
13		YJR030C	YPDcl2	YPDcl3	EtOHcl1	EtOHcl2	Galcl1	YPDnc1	YPDnc2	YPDnc3	YPDnc4	EtOHnc1	EtOHnc2	Galnc1	Galnc2
13	GRX2	YDR513W	YPDcl2	YPDcl3	EtOHcl1	EtOHcl2	Galcl1	YPDnc1	YPDnc2	YPDnc3	YPDnc4	EtOHnc1	EtOHnc2	Galnc1	Galnc2
13	PSD2	YGR170W	YPDcl2	YPDcl3	EtOHcl1	EtOHcl2	Galcl1	YPDnc1	YPDnc2	YPDnc3	YPDnc4	EtOHnc1	EtOHnc2	Galnc1	Galnc2
13	YDJ1	YNL064C	YPDcl2	YPDcl3	EtOHcl1	EtOHcl2	Galcl1	YPDnc1	YPDnc2	YPDnc3	YPDnc4	EtOHnc1	EtOHnc2	Galnc1	Galnc2
13	TRM7	YBR061C	YPDcl2	YPDcl3	EtOHcl1	EtOHcl2	Galcl1	YPDnc1	YPDnc2	YPDnc3	YPDnc4	EtOHnc1	EtOHnc2	Galnc1	Galnc2
13	TIR2	YOR010C	YPDcl2	YPDcl3	EtOHcl1	EtOHcl2	Galcl1	YPDnc1	YPDnc2	YPDnc3	YPDnc4	EtOHnc1	EtOHnc2	Galnc1	Galnc2
13	SNF8	YPL002C	YPDcl2	YPDcl3	EtOHcl1	EtOHcl2	Galcl1	YPDnc1	YPDnc2	YPDnc3	YPDnc4	EtOHnc1	EtOHnc2	Galnc1	Galnc2
13	RPL27A	YHR010W	YPDcl2	YPDcl3	EtOHcl1	EtOHcl2	Galcl1	YPDnc1	YPDnc2	YPDnc3	YPDnc4	EtOHnc1	EtOHnc2	Galnc1	Galnc2
13	RPS8B	YER102W	YPDcl2	YPDcl3	EtOHcl1	EtOHcl2	Galcl1	YPDnc1	YPDnc2	YPDnc3	YPDnc4	EtOHnc1	EtOHnc2	Galnc1	Galnc2
13	MUD2	YKL074C	YPDcl2	YPDcl3	EtOHcl1	EtOHcl2	Galcl1	YPDnc1	YPDnc2	YPDnc3	YPDnc4	EtOHnc1	EtOHnc2	Galnc1	Galnc2

13		YKR023W	YPDcl2	YPDcl3	EtOHcl1	EtOHcl2	Galcl1	YPDnc1	YPDnc2	YPDnc3	YPDnc4	EtOHnc1	EtOHnc2	Galnc1	Galnc2
13	RBG1	YAL036C	YPDcl2	YPDcl3	EtOHcl1	EtOHcl2	Galcl1	YPDnc1	YPDnc2	YPDnc3	YPDnc4	EtOHnc1	EtOHnc2	Galnc1	Galnc2
13	RRP14	YKL082C	YPDcl2	YPDcl3	EtOHcl1	EtOHcl2	Galcl1	YPDnc1	YPDnc2	YPDnc3	YPDnc4	EtOHnc1	EtOHnc2	Galnc1	Galnc2
13	CDH1	YGL003C	YPDcl2	YPDcl3	EtOHcl1	EtOHcl2	Galcl1	YPDnc1	YPDnc2	YPDnc3	YPDnc4	EtOHnc1	EtOHnc2	Galnc1	Galnc2
13		YGR237C	YPDcl2	YPDcl3	EtOHcl1	EtOHcl2	Galcl1	YPDnc1	YPDnc2	YPDnc3	YPDnc4	EtOHnc1	EtOHnc2	Galnc1	Galnc2
13	FKH1	YIL131C	YPDcl2	YPDcl3	EtOHcl1	EtOHcl2	Galcl1	YPDnc1	YPDnc2	YPDnc3	YPDnc4	EtOHnc1	EtOHnc2	Galnc1	Galnc2
13	DBF20	YPR111W	YPDcl2	YPDcl3	EtOHcl1	EtOHcl2	Galcl1	YPDnc1	YPDnc2	YPDnc3	YPDnc4	EtOHnc1	EtOHnc2	Galnc1	Galnc2
13	INM2	YDR287W	YPDcl2	YPDcl3	EtOHcl1	EtOHcl2	Galcl1	YPDnc1	YPDnc2	YPDnc3	YPDnc4	EtOHnc1	EtOHnc2	Galnc1	Galnc2
13	MIC17	YMR002W	YPDcl2	YPDcl3	EtOHcl1	EtOHcl2	Galcl1	YPDnc1	YPDnc2	YPDnc3	YPDnc4	EtOHnc1	EtOHnc2	Galnc1	Galnc2
13	KIN1	YDR122W	YPDcl2	YPDcl3	EtOHcl1	EtOHcl2	Galcl1	YPDnc1	YPDnc2	YPDnc3	YPDnc4	EtOHnc1	EtOHnc2	Galnc1	Galnc2
13	SRO77	YBL106C	YPDcl2	YPDcl3	EtOHcl1	EtOHcl2	Galcl1	YPDnc1	YPDnc2	YPDnc3	YPDnc4	EtOHnc1	EtOHnc2	Galnc1	Galnc2
13	SHE4	YOR035C	YPDcl2	YPDcl3	EtOHcl1	EtOHcl2	Galcl1	YPDnc1	YPDnc2	YPDnc3	YPDnc4	EtOHnc1	EtOHnc2	Galnc1	Galnc2
13	VPS64	YDR200C	YPDcl2	YPDcl3	EtOHcl1	EtOHcl2	Galcl1	YPDnc1	YPDnc2	YPDnc3	YPDnc4	EtOHnc1	EtOHnc2	Galnc1	Galnc2
13	RRT14	YIL127C	YPDcl2	YPDcl3	EtOHcl1	EtOHcl2	Galcl1	YPDnc1	YPDnc2	YPDnc3	YPDnc4	EtOHnc1	EtOHnc2	Galnc1	Galnc2
13	PRS4	YBL068W	YPDcl2	YPDcl3	EtOHcl1	EtOHcl2	Galcl1	YPDnc1	YPDnc2	YPDnc3	YPDnc4	EtOHnc1	EtOHnc2	Galnc1	Galnc2
13	SEC8	YPR055W	YPDcl2	YPDcl3	EtOHcl1	EtOHcl2	Galcl1	YPDnc1	YPDnc2	YPDnc3	YPDnc4	EtOHnc1	EtOHnc2	Galnc1	Galnc2
13	GPI12	YMR281W	YPDcl2	YPDcl3	EtOHcl1	EtOHcl2	Galcl1	YPDnc1	YPDnc2	YPDnc3	YPDnc4	EtOHnc1	EtOHnc2	Galnc1	Galnc2
13	NTH2	YBR001C	YPDcl2	YPDcl3	EtOHcl1	EtOHcl2	Galcl1	YPDnc1	YPDnc2	YPDnc3	YPDnc4	EtOHnc1	EtOHnc2	Galnc1	Galnc2
13	TRE2	YOR256C	YPDcl2	YPDcl3	EtOHcl1	EtOHcl2	Galcl1	YPDnc1	YPDnc2	YPDnc3	YPDnc4	EtOHnc1	EtOHnc2	Galnc1	Galnc2
13	RTR1	YER139C	YPDcl2	YPDcl3	EtOHcl1	EtOHcl2	Galcl1	YPDnc1	YPDnc2	YPDnc3	YPDnc4	EtOHnc1	EtOHnc2	Galnc1	Galnc2
13		YNL174W	YPDcl2	YPDcl3	EtOHcl1	EtOHcl2	Galcl1	YPDnc1	YPDnc2	YPDnc3	YPDnc4	EtOHnc1	EtOHnc2	Galnc1	Galnc2
13	CDC28	YBR160W	YPDcl2	YPDcl3	EtOHcl1	EtOHcl2	Galcl1	YPDnc1	YPDnc2	YPDnc3	YPDnc4	EtOHnc1	EtOHnc2	Galnc1	Galnc2
13	CCL1	YPR025C	YPDcl2	YPDcl3	EtOHcl1	EtOHcl2	Galcl1	YPDnc1	YPDnc2	YPDnc3	YPDnc4	EtOHnc1	EtOHnc2	Galnc1	Galnc2
13	PAU18	YLL064C	YPDcl2	YPDcl3	EtOHcl1	EtOHcl2	Galcl1	YPDnc1	YPDnc2	YPDnc3	YPDnc4	EtOHnc1	EtOHnc2	Galnc1	Galnc2
13	HRP1	YOL123W	YPDcl2	YPDcl3	EtOHcl1	EtOHcl2	Galcl1	YPDnc1	YPDnc2	YPDnc3	YPDnc4	EtOHnc1	EtOHnc2	Galnc1	Galnc2
13	PAN2	YGL094C	YPDcl2	YPDcl3	EtOHcl1	EtOHcl2	Galcl1	YPDnc1	YPDnc2	YPDnc3	YPDnc4	EtOHnc1	EtOHnc2	Galnc1	Galnc2
13	PDC2	YDR081C	YPDcl2	YPDcl3	EtOHcl1	EtOHcl2	Galcl1	YPDnc1	YPDnc2	YPDnc3	YPDnc4	EtOHnc1	EtOHnc2	Galnc1	Galnc2
13	MNN5	YJL186W	YPDcl2	YPDcl3	EtOHcl1	EtOHcl2	Galcl1	YPDnc1	YPDnc2	YPDnc3	YPDnc4	EtOHnc1	EtOHnc2	Galnc1	Galnc2
13		YIL024C	YPDcl2	YPDcl3	EtOHcl1	EtOHcl2	Galcl1	YPDnc1	YPDnc2	YPDnc3	YPDnc4	EtOHnc1	EtOHnc2	Galnc1	Galnc2
13	CSN12	YJR084W	YPDcl2	YPDcl3	EtOHcl1	EtOHcl2	Galcl1	YPDnc1	YPDnc2	YPDnc3	YPDnc4	EtOHnc1	EtOHnc2	Galnc1	Galnc2
13		YMR087W	YPDcl2	YPDcl3	EtOHcl1	EtOHcl2	Galcl1	YPDnc1	YPDnc2	YPDnc3	YPDnc4	EtOHnc1	EtOHnc2	Galnc1	Galnc2
13	PSR2	YLR019W	YPDcl2	YPDcl3	EtOHcl1	EtOHcl2	Galcl1	YPDnc1	YPDnc2	YPDnc3	YPDnc4	EtOHnc1	EtOHnc2	Galnc1	Galnc2
13	RSM18	YER050C	YPDcl2	YPDcl3	EtOHcl1	EtOHcl2	Galcl1	YPDnc1	YPDnc2	YPDnc3	YPDnc4	EtOHnc1	EtOHnc2	Galnc1	Galnc2
13	SPR3	YGR059W	YPDcl2	YPDcl3	EtOHcl1	EtOHcl2	Galcl1	YPDnc1	YPDnc2	YPDnc3	YPDnc4	EtOHnc1	EtOHnc2	Galnc1	Galnc2

13		YER046W-/	YPDcl2	YPDcl3	EtOHcl1	EtOHcl2	Galcl1	YPDnc1	YPDnc2	YPDnc3	YPDnc4	EtOHnc1	EtOHnc2	Galnc1	Galnc2
13	PET309	YLR067C	YPDcl2	YPDcl3	EtOHcl1	EtOHcl2	Galcl1	YPDnc1	YPDnc2	YPDnc3	YPDnc4	EtOHnc1	EtOHnc2	Galnc1	Galnc2
13		YMR082C	YPDcl2	YPDcl3	EtOHcl1	EtOHcl2	Galcl1	YPDnc1	YPDnc2	YPDnc3	YPDnc4	EtOHnc1	EtOHnc2	Galnc1	Galnc2
13	VMA5	YKL080W	YPDcl2	YPDcl3	EtOHcl1	EtOHcl2	Galcl1	YPDnc1	YPDnc2	YPDnc3	YPDnc4	EtOHnc1	EtOHnc2	Galnc1	Galnc2
13	TMA108	YIL137C	YPDcl2	YPDcl3	EtOHcl1	EtOHcl2	Galcl1	YPDnc1	YPDnc2	YPDnc3	YPDnc4	EtOHnc1	EtOHnc2	Galnc1	Galnc2
13	SSA3	YBL075C	YPDcl2	YPDcl3	EtOHcl1	EtOHcl2	Galcl1	YPDnc1	YPDnc2	YPDnc3	YPDnc4	EtOHnc1	EtOHnc2	Galnc1	Galnc2
13	RAI1	YGL246C	YPDcl2	YPDcl3	EtOHcl1	EtOHcl2	Galcl1	YPDnc1	YPDnc2	YPDnc3	YPDnc4	EtOHnc1	EtOHnc2	Galnc1	Galnc2
13	ISN1	YOR155C	YPDcl2	YPDcl3	EtOHcl1	EtOHcl2	Galcl1	YPDnc1	YPDnc2	YPDnc3	YPDnc4	EtOHnc1	EtOHnc2	Galnc1	Galnc2
13	MRF1	YGL143C	YPDcl2	YPDcl3	EtOHcl1	EtOHcl2	Galcl1	YPDnc1	YPDnc2	YPDnc3	YPDnc4	EtOHnc1	EtOHnc2	Galnc1	Galnc2
13	LAP3	YNL239W	YPDcl2	YPDcl3	EtOHcl1	EtOHcl2	Galcl1	YPDnc1	YPDnc2	YPDnc3	YPDnc4	EtOHnc1	EtOHnc2	Galnc1	Galnc2
13		YNR073C	YPDcl2	YPDcl3	EtOHcl1	EtOHcl2	Galcl1	YPDnc1	YPDnc2	YPDnc3	YPDnc4	EtOHnc1	EtOHnc2	Galnc1	Galnc2
13	COX19	YLL018C-A	YPDcl2	YPDcl3	EtOHcl1	EtOHcl2	Galcl1	YPDnc1	YPDnc2	YPDnc3	YPDnc4	EtOHnc1	EtOHnc2	Galnc1	Galnc2
13	CYK3	YDL117W	YPDcl2	YPDcl3	EtOHcl1	EtOHcl2	Galcl1	YPDnc1	YPDnc2	YPDnc3	YPDnc4	EtOHnc1	EtOHnc2	Galnc1	Galnc2
13		YOL075C	YPDcl2	YPDcl3	EtOHcl1	EtOHcl2	Galcl1	YPDnc1	YPDnc2	YPDnc3	YPDnc4	EtOHnc1	EtOHnc2	Galnc1	Galnc2
13	GPM3	YOL056W	YPDcl2	YPDcl3	EtOHcl1	EtOHcl2	Galcl1	YPDnc1	YPDnc2	YPDnc3	YPDnc4	EtOHnc1	EtOHnc2	Galnc1	Galnc2
13		YCR045W-/	YPDcl2	YPDcl3	EtOHcl1	EtOHcl2	Galcl1	YPDnc1	YPDnc2	YPDnc3	YPDnc4	EtOHnc1	EtOHnc2	Galnc1	Galnc2
13	TIM44	YIL022W	YPDcl2	YPDcl3	EtOHcl1	EtOHcl2	Galcl1	YPDnc1	YPDnc2	YPDnc3	YPDnc4	EtOHnc1	EtOHnc2	Galnc1	Galnc2
13	IRC21	YMR073C	YPDcl2	YPDcl3	EtOHcl1	EtOHcl2	Galcl1	YPDnc1	YPDnc2	YPDnc3	YPDnc4	EtOHnc1	EtOHnc2	Galnc1	Galnc2
13	RAD3	YER171W	YPDcl2	YPDcl3	EtOHcl1	EtOHcl2	Galcl1	YPDnc1	YPDnc2	YPDnc3	YPDnc4	EtOHnc1	EtOHnc2	Galnc1	Galnc2
13	NUP1	YOR098C	YPDcl2	YPDcl3	EtOHcl1	EtOHcl2	Galcl1	YPDnc1	YPDnc2	YPDnc3	YPDnc4	EtOHnc1	EtOHnc2	Galnc1	Galnc2
13	IRR1	YIL026C	YPDcl2	YPDcl3	EtOHcl1	EtOHcl2	Galcl1	YPDnc1	YPDnc2	YPDnc3	YPDnc4	EtOHnc1	EtOHnc2	Galnc1	Galnc2
13	OCA6	YDR067C	YPDcl2	YPDcl3	EtOHcl1	EtOHcl2	Galcl1	YPDnc1	YPDnc2	YPDnc3	YPDnc4	EtOHnc1	EtOHnc2	Galnc1	Galnc2
13	SEC9	YGR009C	YPDcl2	YPDcl3	EtOHcl1	EtOHcl2	Galcl1	YPDnc1	YPDnc2	YPDnc3	YPDnc4	EtOHnc1	EtOHnc2	Galnc1	Galnc2
13		YJL175W	YPDcl2	YPDcl3	EtOHcl1	EtOHcl2	Galcl1	YPDnc1	YPDnc2	YPDnc3	YPDnc4	EtOHnc1	EtOHnc2	Galnc1	Galnc2
13		YGR204C-A	YPDcl2	YPDcl3	EtOHcl1	EtOHcl2	Galcl1	YPDnc1	YPDnc2	YPDnc3	YPDnc4	EtOHnc1	EtOHnc2	Galnc1	Galnc2
13	PSP2	YML017W	YPDcl2	YPDcl3	EtOHcl1	EtOHcl2	Galcl1	YPDnc1	YPDnc2	YPDnc3	YPDnc4	EtOHnc1	EtOHnc2	Galnc1	Galnc2
13	NAB2	YGL122C	YPDcl2	YPDcl3	EtOHcl1	EtOHcl2	Galcl1	YPDnc1	YPDnc2	YPDnc3	YPDnc4	EtOHnc1	EtOHnc2	Galnc1	Galnc2
13	ECM16	YMR128W	YPDcl2	YPDcl3	EtOHcl1	EtOHcl2	Galcl1	YPDnc1	YPDnc2	YPDnc3	YPDnc4	EtOHnc1	EtOHnc2	Galnc1	Galnc2
13	VAC8	YEL013W	YPDcl2	YPDcl3	EtOHcl1	EtOHcl2	Galcl1	YPDnc1	YPDnc2	YPDnc3	YPDnc4	EtOHnc1	EtOHnc2	Galnc1	Galnc2
13	KAR5	YMR065W	YPDcl2	YPDcl3	EtOHcl1	EtOHcl2	Galcl1	YPDnc1	YPDnc2	YPDnc3	YPDnc4	EtOHnc1	EtOHnc2	Galnc1	Galnc2
13		YIR020W-A	YPDcl2	YPDcl3	EtOHcl1	EtOHcl2	Galcl1	YPDnc1	YPDnc2	YPDnc3	YPDnc4	EtOHnc1	EtOHnc2	Galnc1	Galnc2
13	SEC6	YIL068C	YPDcl2	YPDcl3	EtOHcl1	EtOHcl2	Galcl1	YPDnc1	YPDnc2	YPDnc3	YPDnc4	EtOHnc1	EtOHnc2	Galnc1	Galnc2
13	DPM1	YPR183W	YPDcl2	YPDcl3	EtOHcl1	EtOHcl2	Galcl1	YPDnc1	YPDnc2	YPDnc3	YPDnc4	EtOHnc1	EtOHnc2	Galnc1	Galnc2
13		YLR125W	YPDcl2	YPDcl3	EtOHcl1	EtOHcl2	Galcl1	YPDnc1	YPDnc2	YPDnc3	YPDnc4	EtOHnc1	EtOHnc2	Galnc1	Galnc2

13		YOL024W	YPDcl2	YPDcl3	EtOHcl1	EtOHcl2	Galcl1	YPDnc1	YPDnc2	YPDnc3	YPDnc4	EtOHnc1	EtOHnc2	Galnc1	Galnc2
13		YGR012W	YPDcl2	YPDcl3	EtOHcl1	EtOHcl2	Galcl1	YPDnc1	YPDnc2	YPDnc3	YPDnc4	EtOHnc1	EtOHnc2	Galnc1	Galnc2
13		YNL095C	YPDcl2	YPDcl3	EtOHcl1	EtOHcl2	Galcl1	YPDnc1	YPDnc2	YPDnc3	YPDnc4	EtOHnc1	EtOHnc2	Galnc1	Galnc2
13	BUD2	YKL092C	YPDcl2	YPDcl3	EtOHcl1	EtOHcl2	Galcl1	YPDnc1	YPDnc2	YPDnc3	YPDnc4	EtOHnc1	EtOHnc2	Galnc1	Galnc2
13	DSF2	YBR007C	YPDcl2	YPDcl3	EtOHcl1	EtOHcl2	Galcl1	YPDnc1	YPDnc2	YPDnc3	YPDnc4	EtOHnc1	EtOHnc2	Galnc1	Galnc2
13	NTG1	YAL015C	YPDcl2	YPDcl3	EtOHcl1	EtOHcl2	Galcl1	YPDnc1	YPDnc2	YPDnc3	YPDnc4	EtOHnc1	EtOHnc2	Galnc1	Galnc2
13	HXK2	YGL253W	YPDcl2	YPDcl3	EtOHcl1	EtOHcl2	Galcl1	YPDnc1	YPDnc2	YPDnc3	YPDnc4	EtOHnc1	EtOHnc2	Galnc1	Galnc2
13	LIP5	YOR196C	YPDcl2	YPDcl3	EtOHcl1	EtOHcl2	Galcl1	YPDnc1	YPDnc2	YPDnc3	YPDnc4	EtOHnc1	EtOHnc2	Galnc1	Galnc2
13	MDM20	YOL076W	YPDcl2	YPDcl3	EtOHcl1	EtOHcl2	Galcl1	YPDnc1	YPDnc2	YPDnc3	YPDnc4	EtOHnc1	EtOHnc2	Galnc1	Galnc2
13	RTN1	YDR233C	YPDcl2	YPDcl3	EtOHcl1	EtOHcl2	Galcl1	YPDnc1	YPDnc2	YPDnc3	YPDnc4	EtOHnc1	EtOHnc2	Galnc1	Galnc2
13		YCR075W-/	YPDcl2	YPDcl3	EtOHcl1	EtOHcl2	Galcl1	YPDnc1	YPDnc2	YPDnc3	YPDnc4	EtOHnc1	EtOHnc2	Galnc1	Galnc2
13	UTR2	YEL040W	YPDcl2	YPDcl3	EtOHcl1	EtOHcl2	Galcl1	YPDnc1	YPDnc2	YPDnc3	YPDnc4	EtOHnc1	EtOHnc2	Galnc1	Galnc2
13		YMR252C	YPDcl2	YPDcl3	EtOHcl1	EtOHcl2	Galcl1	YPDnc1	YPDnc2	YPDnc3	YPDnc4	EtOHnc1	EtOHnc2	Galnc1	Galnc2
13	IME2	YJL106W	YPDcl2	YPDcl3	EtOHcl1	EtOHcl2	Galcl1	YPDnc1	YPDnc2	YPDnc3	YPDnc4	EtOHnc1	EtOHnc2	Galnc1	Galnc2
13	HYM1	YKL189W	YPDcl2	YPDcl3	EtOHcl1	EtOHcl2	Galcl1	YPDnc1	YPDnc2	YPDnc3	YPDnc4	EtOHnc1	EtOHnc2	Galnc1	Galnc2
13	MXR2	YCL033C	YPDcl2	YPDcl3	EtOHcl1	EtOHcl2	Galcl1	YPDnc1	YPDnc2	YPDnc3	YPDnc4	EtOHnc1	EtOHnc2	Galnc1	Galnc2
13		YOR041C	YPDcl2	YPDcl3	EtOHcl1	EtOHcl2	Galcl1	YPDnc1	YPDnc2	YPDnc3	YPDnc4	EtOHnc1	EtOHnc2	Galnc1	Galnc2
13	PRP18	YGR006W	YPDcl2	YPDcl3	EtOHcl1	EtOHcl2	Galcl1	YPDnc1	YPDnc2	YPDnc3	YPDnc4	EtOHnc1	EtOHnc2	Galnc1	Galnc2
13		YPL041C	YPDcl2	YPDcl3	EtOHcl1	EtOHcl2	Galcl1	YPDnc1	YPDnc2	YPDnc3	YPDnc4	EtOHnc1	EtOHnc2	Galnc1	Galnc2
13		YOL131W	YPDcl2	YPDcl3	EtOHcl1	EtOHcl2	Galcl1	YPDnc1	YPDnc2	YPDnc3	YPDnc4	EtOHnc1	EtOHnc2	Galnc1	Galnc2
13	PIR3	YKL163W	YPDcl2	YPDcl3	EtOHcl1	EtOHcl2	Galcl1	YPDnc1	YPDnc2	YPDnc3	YPDnc4	EtOHnc1	EtOHnc2	Galnc1	Galnc2
13	PTC6	YCR079W	YPDcl2	YPDcl3	EtOHcl1	EtOHcl2	Galcl1	YPDnc1	YPDnc2	YPDnc3	YPDnc4	EtOHnc1	EtOHnc2	Galnc1	Galnc2
13	MRPS18	YNL306W	YPDcl2	YPDcl3	EtOHcl1	EtOHcl2	Galcl1	YPDnc1	YPDnc2	YPDnc3	YPDnc4	EtOHnc1	EtOHnc2	Galnc1	Galnc2
13	HMT1	YBR034C	YPDcl2	YPDcl3	EtOHcl1	EtOHcl2	Galcl1	YPDnc1	YPDnc2	YPDnc3	YPDnc4	EtOHnc1	EtOHnc2	Galnc1	Galnc2
13	SLT2	YHR030C	YPDcl2	YPDcl3	EtOHcl1	EtOHcl2	Galcl1	YPDnc1	YPDnc2	YPDnc3	YPDnc4	EtOHnc1	EtOHnc2	Galnc1	Galnc2
13	ASN2	YGR124W	YPDcl2	YPDcl3	EtOHcl1	EtOHcl2	Galcl1	YPDnc1	YPDnc2	YPDnc3	YPDnc4	EtOHnc1	EtOHnc2	Galnc1	Galnc2
13	RPS14A	YCR031C	YPDcl2	YPDcl3	EtOHcl1	EtOHcl2	Galcl1	YPDnc1	YPDnc2	YPDnc3	YPDnc4	EtOHnc1	EtOHnc2	Galnc1	Galnc2
13	DDP1	YOR163W	YPDcl2	YPDcl3	EtOHcl1	EtOHcl2	Galcl1	YPDnc1	YPDnc2	YPDnc3	YPDnc4	EtOHnc1	EtOHnc2	Galnc1	Galnc2
13	ATG27	YJL178C	YPDcl2	YPDcl3	EtOHcl1	EtOHcl2	Galcl1	YPDnc1	YPDnc2	YPDnc3	YPDnc4	EtOHnc1	EtOHnc2	Galnc1	Galnc2
13		YOR051C	YPDcl2	YPDcl3	EtOHcl1	EtOHcl2	Galcl1	YPDnc1	YPDnc2	YPDnc3	YPDnc4	EtOHnc1	EtOHnc2	Galnc1	Galnc2
13		YLR352W	YPDcl2	YPDcl3	EtOHcl1	EtOHcl2	Galcl1	YPDnc1	YPDnc2	YPDnc3	YPDnc4	EtOHnc1	EtOHnc2	Galnc1	Galnc2
13		YKL018C-A	YPDcl2	YPDcl3	EtOHcl1	EtOHcl2	Galcl1	YPDnc1	YPDnc2	YPDnc3	YPDnc4	EtOHnc1	EtOHnc2	Galnc1	Galnc2
13	MRD1	YPR112C	YPDcl2	YPDcl3	EtOHcl1	EtOHcl2	Galcl1	YPDnc1	YPDnc2	YPDnc3	YPDnc4	EtOHnc1	EtOHnc2	Galnc1	Galnc2
13		YDR354C-A	YPDcl2	YPDcl3	EtOHcl1	EtOHcl2	Galcl1	YPDnc1	YPDnc2	YPDnc3	YPDnc4	EtOHnc1	EtOHnc2	Galnc1	Galnc2

13	DBP6	YNR038W	YPDcl2	YPDcl3	EtOHcl1	EtOHcl2	Galcl1	YPDnc1	YPDnc2	YPDnc3	YPDnc4	EtOHnc1	EtOHnc2	Galnc1	Galnc2
13	SEC24	YIL109C	YPDcl2	YPDcl3	EtOHcl1	EtOHcl2	Galcl1	YPDnc1	YPDnc2	YPDnc3	YPDnc4	EtOHnc1	EtOHnc2	Galnc1	Galnc2
13	MNT2	YGL257C	YPDcl2	YPDcl3	EtOHcl1	EtOHcl2	Galcl1	YPDnc1	YPDnc2	YPDnc3	YPDnc4	EtOHnc1	EtOHnc2	Galnc1	Galnc2
13	PRS2	YER099C	YPDcl2	YPDcl3	EtOHcl1	EtOHcl2	Galcl1	YPDnc1	YPDnc2	YPDnc3	YPDnc4	EtOHnc1	EtOHnc2	Galnc1	Galnc2
13	IPI3	YNL182C	YPDcl2	YPDcl3	EtOHcl1	EtOHcl2	Galcl1	YPDnc1	YPDnc2	YPDnc3	YPDnc4	EtOHnc1	EtOHnc2	Galnc1	Galnc2
13	TIF3	YPR163C	YPDcl2	YPDcl3	EtOHcl1	EtOHcl2	Galcl1	YPDnc1	YPDnc2	YPDnc3	YPDnc4	EtOHnc1	EtOHnc2	Galnc1	Galnc2
13	CAT2	YML042W	YPDcl2	YPDcl3	EtOHcl1	EtOHcl2	Galcl1	YPDnc1	YPDnc2	YPDnc3	YPDnc4	EtOHnc1	EtOHnc2	Galnc1	Galnc2
13	RSR1	YGR152C	YPDcl2	YPDcl3	EtOHcl1	EtOHcl2	Galcl1	YPDnc1	YPDnc2	YPDnc3	YPDnc4	EtOHnc1	EtOHnc2	Galnc1	Galnc2
13	SEC7	YDR170C	YPDcl2	YPDcl3	EtOHcl1	EtOHcl2	Galcl1	YPDnc1	YPDnc2	YPDnc3	YPDnc4	EtOHnc1	EtOHnc2	Galnc1	Galnc2
13	TPM2	YIL138C	YPDcl2	YPDcl3	EtOHcl1	EtOHcl2	Galcl1	YPDnc1	YPDnc2	YPDnc3	YPDnc4	EtOHnc1	EtOHnc2	Galnc1	Galnc2
13	MSW1	YDR268W	YPDcl2	YPDcl3	EtOHcl1	EtOHcl2	Galcl1	YPDnc1	YPDnc2	YPDnc3	YPDnc4	EtOHnc1	EtOHnc2	Galnc1	Galnc2
13	MET22	YOL064C	YPDcl2	YPDcl3	EtOHcl1	EtOHcl2	Galcl1	YPDnc1	YPDnc2	YPDnc3	YPDnc4	EtOHnc1	EtOHnc2	Galnc1	Galnc2
13	IGO2	YHR132W-/	YPDcl2	YPDcl3	EtOHcl1	EtOHcl2	Galcl1	YPDnc1	YPDnc2	YPDnc3	YPDnc4	EtOHnc1	EtOHnc2	Galnc1	Galnc2
13		YJL197C-A	YPDcl2	YPDcl3	EtOHcl1	EtOHcl2	Galcl1	YPDnc1	YPDnc2	YPDnc3	YPDnc4	EtOHnc1	EtOHnc2	Galnc1	Galnc2
13	HUA2	YOR284W	YPDcl2	YPDcl3	EtOHcl1	EtOHcl2	Galcl1	YPDnc1	YPDnc2	YPDnc3	YPDnc4	EtOHnc1	EtOHnc2	Galnc1	Galnc2
13	SME1	YOR159C	YPDcl2	YPDcl3	EtOHcl1	EtOHcl2	Galcl1	YPDnc1	YPDnc2	YPDnc3	YPDnc4	EtOHnc1	EtOHnc2	Galnc1	Galnc2
13	ATP16	YDL004W	YPDcl2	YPDcl3	EtOHcl1	EtOHcl2	Galcl1	YPDnc1	YPDnc2	YPDnc3	YPDnc4	EtOHnc1	EtOHnc2	Galnc1	Galnc2
13	MSB1	YOR188W	YPDcl2	YPDcl3	EtOHcl1	EtOHcl2	Galcl1	YPDnc1	YPDnc2	YPDnc3	YPDnc4	EtOHnc1	EtOHnc2	Galnc1	Galnc2
13	DID4	YKL002W	YPDcl2	YPDcl3	EtOHcl1	EtOHcl2	Galcl1	YPDnc1	YPDnc2	YPDnc3	YPDnc4	EtOHnc1	EtOHnc2	Galnc1	Galnc2
13	TPK1	YJL164C	YPDcl2	YPDcl3	EtOHcl1	EtOHcl2	Galcl1	YPDnc1	YPDnc2	YPDnc3	YPDnc4	EtOHnc1	EtOHnc2	Galnc1	Galnc2
13	UBC6	YER100W	YPDcl2	YPDcl3	EtOHcl1	EtOHcl2	Galcl1	YPDnc1	YPDnc2	YPDnc3	YPDnc4	EtOHnc1	EtOHnc2	Galnc1	Galnc2
13	HFD1	YMR110C	YPDcl2	YPDcl3	EtOHcl1	EtOHcl2	Galcl1	YPDnc1	YPDnc2	YPDnc3	YPDnc4	EtOHnc1	EtOHnc2	Galnc1	Galnc2
13	PCF11	YDR228C	YPDcl2	YPDcl3	EtOHcl1	EtOHcl2	Galcl1	YPDnc1	YPDnc2	YPDnc3	YPDnc4	EtOHnc1	EtOHnc2	Galnc1	Galnc2
13	EMP47	YFL048C	YPDcl2	YPDcl3	EtOHcl1	EtOHcl2	Galcl1	YPDnc1	YPDnc2	YPDnc3	YPDnc4	EtOHnc1	EtOHnc2	Galnc1	Galnc2
13		YPR117W	YPDcl2	YPDcl3	EtOHcl1	EtOHcl2	Galcl1	YPDnc1	YPDnc2	YPDnc3	YPDnc4	EtOHnc1	EtOHnc2	Galnc1	Galnc2
13	ENP1	YBR247C	YPDcl2	YPDcl3	EtOHcl1	EtOHcl2	Galcl1	YPDnc1	YPDnc2	YPDnc3	YPDnc4	EtOHnc1	EtOHnc2	Galnc1	Galnc2
13	UBR2	YLR024C	YPDcl2	YPDcl3	EtOHcl1	EtOHcl2	Galcl1	YPDnc1	YPDnc2	YPDnc3	YPDnc4	EtOHnc1	EtOHnc2	Galnc1	Galnc2
13	SWD2	YKL018W	YPDcl2	YPDcl3	EtOHcl1	EtOHcl2	Galcl1	YPDnc1	YPDnc2	YPDnc3	YPDnc4	EtOHnc1	EtOHnc2	Galnc1	Galnc2
13	GCN3	YKR026C	YPDcl2	YPDcl3	EtOHcl1	EtOHcl2	Galcl1	YPDnc1	YPDnc2	YPDnc3	YPDnc4	EtOHnc1	EtOHnc2	Galnc1	Galnc2
13	SSA2	YLL024C	YPDcl2	YPDcl3	EtOHcl1	EtOHcl2	Galcl1	YPDnc1	YPDnc2	YPDnc3	YPDnc4	EtOHnc1	EtOHnc2	Galnc1	Galnc2
13	SAC3	YDR159W	YPDcl2	YPDcl3	EtOHcl1	EtOHcl2	Galcl1	YPDnc1	YPDnc2	YPDnc3	YPDnc4	EtOHnc1	EtOHnc2	Galnc1	Galnc2
13	NTO1	YPR031W	YPDcl2	YPDcl3	EtOHcl1	EtOHcl2	Galcl1	YPDnc1	YPDnc2	YPDnc3	YPDnc4	EtOHnc1	EtOHnc2	Galnc1	Galnc2
13	GLO4	YOR040W	YPDcl2	YPDcl3	EtOHcl1	EtOHcl2	Galcl1	YPDnc1	YPDnc2	YPDnc3	YPDnc4	EtOHnc1	EtOHnc2	Galnc1	Galnc2
13	COX4	YGL187C	YPDcl2	YPDcl3	EtOHcl1	EtOHcl2	Galcl1	YPDnc1	YPDnc2	YPDnc3	YPDnc4	EtOHnc1	EtOHnc2	Galnc1	Galnc2

13	PEP7	YDR323C	YPDcl2	YPDcl3	EtOHcl1	EtOHcl2	Galcl1	YPDnc1	YPDnc2	YPDnc3	YPDnc4	EtOHnc1	EtOHnc2	Galnc1	Galnc2
13	SFC1	YJR095W	YPDcl2	YPDcl3	EtOHcl1	EtOHcl2	Galcl1	YPDnc1	YPDnc2	YPDnc3	YPDnc4	EtOHnc1	EtOHnc2	Galnc1	Galnc2
13	VPS72	YDR485C	YPDcl2	YPDcl3	EtOHcl1	EtOHcl2	Galcl1	YPDnc1	YPDnc2	YPDnc3	YPDnc4	EtOHnc1	EtOHnc2	Galnc1	Galnc2
13	KTR5	YNL029C	YPDcl2	YPDcl3	EtOHcl1	EtOHcl2	Galcl1	YPDnc1	YPDnc2	YPDnc3	YPDnc4	EtOHnc1	EtOHnc2	Galnc1	Galnc2
13	MAD3	YJL013C	YPDcl2	YPDcl3	EtOHcl1	EtOHcl2	Galcl1	YPDnc1	YPDnc2	YPDnc3	YPDnc4	EtOHnc1	EtOHnc2	Galnc1	Galnc2
13		YGR011W	YPDcl2	YPDcl3	EtOHcl1	EtOHcl2	Galcl1	YPDnc1	YPDnc2	YPDnc3	YPDnc4	EtOHnc1	EtOHnc2	Galnc1	Galnc2
13	EAF7	YNL136W	YPDcl2	YPDcl3	EtOHcl1	EtOHcl2	Galcl1	YPDnc1	YPDnc2	YPDnc3	YPDnc4	EtOHnc1	EtOHnc2	Galnc1	Galnc2
13	JLP1	YLL057C	YPDcl2	YPDcl3	EtOHcl1	EtOHcl2	Galcl1	YPDnc1	YPDnc2	YPDnc3	YPDnc4	EtOHnc1	EtOHnc2	Galnc1	Galnc2
13	USO1	YDL058W	YPDcl2	YPDcl3	EtOHcl1	EtOHcl2	Galcl1	YPDnc1	YPDnc2	YPDnc3	YPDnc4	EtOHnc1	EtOHnc2	Galnc1	Galnc2
13	PTH2	YBL057C	YPDcl2	YPDcl3	EtOHcl1	EtOHcl2	Galcl1	YPDnc1	YPDnc2	YPDnc3	YPDnc4	EtOHnc1	EtOHnc2	Galnc1	Galnc2
13		YER064C	YPDcl2	YPDcl3	EtOHcl1	EtOHcl2	Galcl1	YPDnc1	YPDnc2	YPDnc3	YPDnc4	EtOHnc1	EtOHnc2	Galnc1	Galnc2
13	CEG1	YGL130W	YPDcl2	YPDcl3	EtOHcl1	EtOHcl2	Galcl1	YPDnc1	YPDnc2	YPDnc3	YPDnc4	EtOHnc1	EtOHnc2	Galnc1	Galnc2
13	PCA1	YBR295W	YPDcl2	YPDcl3	EtOHcl1	EtOHcl2	Galcl1	YPDnc1	YPDnc2	YPDnc3	YPDnc4	EtOHnc1	EtOHnc2	Galnc1	Galnc2
13	PRP16	YKR086W	YPDcl2	YPDcl3	EtOHcl1	EtOHcl2	Galcl1	YPDnc1	YPDnc2	YPDnc3	YPDnc4	EtOHnc1	EtOHnc2	Galnc1	Galnc2
13	CTI6	YPL181W	YPDcl2	YPDcl3	EtOHcl1	EtOHcl2	Galcl1	YPDnc1	YPDnc2	YPDnc3	YPDnc4	EtOHnc1	EtOHnc2	Galnc1	Galnc2
13	UFE1	YOR075W	YPDcl2	YPDcl3	EtOHcl1	EtOHcl2	Galcl1	YPDnc1	YPDnc2	YPDnc3	YPDnc4	EtOHnc1	EtOHnc2	Galnc1	Galnc2
13	ATG16	YMR159C	YPDcl2	YPDcl3	EtOHcl1	EtOHcl2	Galcl1	YPDnc1	YPDnc2	YPDnc3	YPDnc4	EtOHnc1	EtOHnc2	Galnc1	Galnc2
13	GUP1	YGL084C	YPDcl2	YPDcl3	EtOHcl1	EtOHcl2	Galcl1	YPDnc1	YPDnc2	YPDnc3	YPDnc4	EtOHnc1	EtOHnc2	Galnc1	Galnc2
13		YDR541C	YPDcl2	YPDcl3	EtOHcl1	EtOHcl2	Galcl1	YPDnc1	YPDnc2	YPDnc3	YPDnc4	EtOHnc1	EtOHnc2	Galnc1	Galnc2
13		YPL245W	YPDcl2	YPDcl3	EtOHcl1	EtOHcl2	Galcl1	YPDnc1	YPDnc2	YPDnc3	YPDnc4	EtOHnc1	EtOHnc2	Galnc1	Galnc2
13	XRS2	YDR369C	YPDcl2	YPDcl3	EtOHcl1	EtOHcl2	Galcl1	YPDnc1	YPDnc2	YPDnc3	YPDnc4	EtOHnc1	EtOHnc2	Galnc1	Galnc2
13	GSH2	YOL049W	YPDcl2	YPDcl3	EtOHcl1	EtOHcl2	Galcl1	YPDnc1	YPDnc2	YPDnc3	YPDnc4	EtOHnc1	EtOHnc2	Galnc1	Galnc2
13		YJR140W-A	YPDcl2	YPDcl3	EtOHcl1	EtOHcl2	Galcl1	YPDnc1	YPDnc2	YPDnc3	YPDnc4	EtOHnc1	EtOHnc2	Galnc1	Galnc2
13	VBA1	YMR088C	YPDcl2	YPDcl3	EtOHcl1	EtOHcl2	Galcl1	YPDnc1	YPDnc2	YPDnc3	YPDnc4	EtOHnc1	EtOHnc2	Galnc1	Galnc2
13	VPS55	YJR044C	YPDcl2	YPDcl3	EtOHcl1	EtOHcl2	Galcl1	YPDnc1	YPDnc2	YPDnc3	YPDnc4	EtOHnc1	EtOHnc2	Galnc1	Galnc2
13	PPZ2	YDR436W	YPDcl2	YPDcl3	EtOHcl1	EtOHcl2	Galcl1	YPDnc1	YPDnc2	YPDnc3	YPDnc4	EtOHnc1	EtOHnc2	Galnc1	Galnc2
13	CAB2	YIL083C	YPDcl2	YPDcl3	EtOHcl1	EtOHcl2	Galcl1	YPDnc1	YPDnc2	YPDnc3	YPDnc4	EtOHnc1	EtOHnc2	Galnc1	Galnc2
13	UBP2	YOR124C	YPDcl2	YPDcl3	EtOHcl1	EtOHcl2	Galcl1	YPDnc1	YPDnc2	YPDnc3	YPDnc4	EtOHnc1	EtOHnc2	Galnc1	Galnc2
13	NSG1	YHR133C	YPDcl2	YPDcl3	EtOHcl1	EtOHcl2	Galcl1	YPDnc1	YPDnc2	YPDnc3	YPDnc4	EtOHnc1	EtOHnc2	Galnc1	Galnc2
13		YDR230W	YPDcl2	YPDcl3	EtOHcl1	EtOHcl2	Galcl1	YPDnc1	YPDnc2	YPDnc3	YPDnc4	EtOHnc1	EtOHnc2	Galnc1	Galnc2
13		YIL058W	YPDcl2	YPDcl3	EtOHcl1	EtOHcl2	Galcl1	YPDnc1	YPDnc2	YPDnc3	YPDnc4	EtOHnc1	EtOHnc2	Galnc1	Galnc2
13	GSM1	YJL103C	YPDcl2	YPDcl3	EtOHcl1	EtOHcl2	Galcl1	YPDnc1	YPDnc2	YPDnc3	YPDnc4	EtOHnc1	EtOHnc2	Galnc1	Galnc2
13	COG5	YNL051W	YPDcl2	YPDcl3	EtOHcl1	EtOHcl2	Galcl1	YPDnc1	YPDnc2	YPDnc3	YPDnc4	EtOHnc1	EtOHnc2	Galnc1	Galnc2
13	VPS9	YML097C	YPDcl2	YPDcl3	EtOHcl1	EtOHcl2	Galcl1	YPDnc1	YPDnc2	YPDnc3	YPDnc4	EtOHnc1	EtOHnc2	Galnc1	Galnc2

13	SLN1	YIL147C	YPDcl2	YPDcl3	EtOHcl1	EtOHcl2	Galcl1	YPDnc1	YPDnc2	YPDnc3	YPDnc4	EtOHnc1	EtOHnc2	Galnc1	Galnc2
13	ARP5	YNL059C	YPDcl2	YPDcl3	EtOHcl1	EtOHcl2	Galcl1	YPDnc1	YPDnc2	YPDnc3	YPDnc4	EtOHnc1	EtOHnc2	Galnc1	Galnc2
13	ZIP2	YGL249W	YPDcl2	YPDcl3	EtOHcl1	EtOHcl2	Galcl1	YPDnc1	YPDnc2	YPDnc3	YPDnc4	EtOHnc1	EtOHnc2	Galnc1	Galnc2
13	IZH3	YLR023C	YPDcl2	YPDcl3	EtOHcl1	EtOHcl2	Galcl1	YPDnc1	YPDnc2	YPDnc3	YPDnc4	EtOHnc1	EtOHnc2	Galnc1	Galnc2
13	BNA4	YBL098W	YPDcl2	YPDcl3	EtOHcl1	EtOHcl2	Galcl1	YPDnc1	YPDnc2	YPDnc3	YPDnc4	EtOHnc1	EtOHnc2	Galnc1	Galnc2
13	TDH2	YJR009C	YPDcl2	YPDcl3	EtOHcl1	EtOHcl2	Galcl1	YPDnc1	YPDnc2	YPDnc3	YPDnc4	EtOHnc1	EtOHnc2	Galnc1	Galnc2
13	AOS1	YPR180W	YPDcl2	YPDcl3	EtOHcl1	EtOHcl2	Galcl1	YPDnc1	YPDnc2	YPDnc3	YPDnc4	EtOHnc1	EtOHnc2	Galnc1	Galnc2
13	INP1	YMR204C	YPDcl2	YPDcl3	EtOHcl1	EtOHcl2	Galcl1	YPDnc1	YPDnc2	YPDnc3	YPDnc4	EtOHnc1	EtOHnc2	Galnc1	Galnc2
13	ARP6	YLR085C	YPDcl2	YPDcl3	EtOHcl1	EtOHcl2	Galcl1	YPDnc1	YPDnc2	YPDnc3	YPDnc4	EtOHnc1	EtOHnc2	Galnc1	Galnc2
13		YLR137W	YPDcl2	YPDcl3	EtOHcl1	EtOHcl2	Galcl1	YPDnc1	YPDnc2	YPDnc3	YPDnc4	EtOHnc1	EtOHnc2	Galnc1	Galnc2
13	BZZ1	YHR114W	YPDcl2	YPDcl3	EtOHcl1	EtOHcl2	Galcl1	YPDnc1	YPDnc2	YPDnc3	YPDnc4	EtOHnc1	EtOHnc2	Galnc1	Galnc2
13	BRE1	YDL074C	YPDcl2	YPDcl3	EtOHcl1	EtOHcl2	Galcl1	YPDnc1	YPDnc2	YPDnc3	YPDnc4	EtOHnc1	EtOHnc2	Galnc1	Galnc2
13	RPA135	YPR010C	YPDcl2	YPDcl3	EtOHcl1	EtOHcl2	Galcl1	YPDnc1	YPDnc2	YPDnc3	YPDnc4	EtOHnc1	EtOHnc2	Galnc1	Galnc2
13	MTM1	YGR257C	YPDcl2	YPDcl3	EtOHcl1	EtOHcl2	Galcl1	YPDnc1	YPDnc2	YPDnc3	YPDnc4	EtOHnc1	EtOHnc2	Galnc1	Galnc2
13	RFM1	YOR279C	YPDcl2	YPDcl3	EtOHcl1	EtOHcl2	Galcl1	YPDnc1	YPDnc2	YPDnc3	YPDnc4	EtOHnc1	EtOHnc2	Galnc1	Galnc2
13	UBC12	YLR306W	YPDcl2	YPDcl3	EtOHcl1	EtOHcl2	Galcl1	YPDnc1	YPDnc2	YPDnc3	YPDnc4	EtOHnc1	EtOHnc2	Galnc1	Galnc2
13	MDV1	YJL112W	YPDcl2	YPDcl3	EtOHcl1	EtOHcl2	Galcl1	YPDnc1	YPDnc2	YPDnc3	YPDnc4	EtOHnc1	EtOHnc2	Galnc1	Galnc2
13	CSG2	YBR036C	YPDcl2	YPDcl3	EtOHcl1	EtOHcl2	Galcl1	YPDnc1	YPDnc2	YPDnc3	YPDnc4	EtOHnc1	EtOHnc2	Galnc1	Galnc2
13	SSU1	YPL092W	YPDcl2	YPDcl3	EtOHcl1	EtOHcl2	Galcl1	YPDnc1	YPDnc2	YPDnc3	YPDnc4	EtOHnc1	EtOHnc2	Galnc1	Galnc2
13	IMH1	YLR309C	YPDcl2	YPDcl3	EtOHcl1	EtOHcl2	Galcl1	YPDnc1	YPDnc2	YPDnc3	YPDnc4	EtOHnc1	EtOHnc2	Galnc1	Galnc2
13		YIR018C-A	YPDcl2	YPDcl3	EtOHcl1	EtOHcl2	Galcl1	YPDnc1	YPDnc2	YPDnc3	YPDnc4	EtOHnc1	EtOHnc2	Galnc1	Galnc2
13		YDR282C	YPDcl2	YPDcl3	EtOHcl1	EtOHcl2	Galcl1	YPDnc1	YPDnc2	YPDnc3	YPDnc4	EtOHnc1	EtOHnc2	Galnc1	Galnc2
13	ALG5	YPL227C	YPDcl2	YPDcl3	EtOHcl1	EtOHcl2	Galcl1	YPDnc1	YPDnc2	YPDnc3	YPDnc4	EtOHnc1	EtOHnc2	Galnc1	Galnc2
13		YLR446W	YPDcl2	YPDcl3	EtOHcl1	EtOHcl2	Galcl1	YPDnc1	YPDnc2	YPDnc3	YPDnc4	EtOHnc1	EtOHnc2	Galnc1	Galnc2
13	IBI1	YGR273C	YPDcl2	YPDcl3	EtOHcl1	EtOHcl2	Galcl1	YPDnc1	YPDnc2	YPDnc3	YPDnc4	EtOHnc1	EtOHnc2	Galnc1	Galnc2
13	ADD37	YMR184W	YPDcl2	YPDcl3	EtOHcl1	EtOHcl2	Galcl1	YPDnc1	YPDnc2	YPDnc3	YPDnc4	EtOHnc1	EtOHnc2	Galnc1	Galnc2
13		YBL081W	YPDcl2	YPDcl3	EtOHcl1	EtOHcl2	Galcl1	YPDnc1	YPDnc2	YPDnc3	YPDnc4	EtOHnc1	EtOHnc2	Galnc1	Galnc2
13		YJR142W	YPDcl2	YPDcl3	EtOHcl1	EtOHcl2	Galcl1	YPDnc1	YPDnc2	YPDnc3	YPDnc4	EtOHnc1	EtOHnc2	Galnc1	Galnc2
13	MDM31	YHR194W	YPDcl2	YPDcl3	EtOHcl1	EtOHcl2	Galcl1	YPDnc1	YPDnc2	YPDnc3	YPDnc4	EtOHnc1	EtOHnc2	Galnc1	Galnc2
13	CNN1	YFR046C	YPDcl2	YPDcl3	EtOHcl1	EtOHcl2	Galcl1	YPDnc1	YPDnc2	YPDnc3	YPDnc4	EtOHnc1	EtOHnc2	Galnc1	Galnc2
13	CWH41	YGL027C	YPDcl2	YPDcl3	EtOHcl1	EtOHcl2	Galcl1	YPDnc1	YPDnc2	YPDnc3	YPDnc4	EtOHnc1	EtOHnc2	Galnc1	Galnc2
13	ATG17	YLR423C	YPDcl2	YPDcl3	EtOHcl1	EtOHcl2	Galcl1	YPDnc1	YPDnc2	YPDnc3	YPDnc4	EtOHnc1	EtOHnc2	Galnc1	Galnc2
13	MRPL33	YMR286W	YPDcl2	YPDcl3	EtOHcl1	EtOHcl2	Galcl1	YPDnc1	YPDnc2	YPDnc3	YPDnc4	EtOHnc1	EtOHnc2	Galnc1	Galnc2
13	PUS9	YDL036C	YPDcl2	YPDcl3	EtOHcl1	EtOHcl2	Galcl1	YPDnc1	YPDnc2	YPDnc3	YPDnc4	EtOHnc1	EtOHnc2	Galnc1	Galnc2

13		YOL159C-A	YPDcl2	YPDcl3	EtOHcl1	EtOHcl2	Galcl1	YPDnc1	YPDnc2	YPDnc3	YPDnc4	EtOHnc1	EtOHnc2	Galnc1	Galnc2
13	REX3	YLR107W	YPDcl2	YPDcl3	EtOHcl1	EtOHcl2	Galcl1	YPDnc1	YPDnc2	YPDnc3	YPDnc4	EtOHnc1	EtOHnc2	Galnc1	Galnc2
13	PET494	YNR045W	YPDcl2	YPDcl3	EtOHcl1	EtOHcl2	Galcl1	YPDnc1	YPDnc2	YPDnc3	YPDnc4	EtOHnc1	EtOHnc2	Galnc1	Galnc2
13		YER137W-	YPDcl2	YPDcl3	EtOHcl1	EtOHcl2	Galcl1	YPDnc1	YPDnc2	YPDnc3	YPDnc4	EtOHnc1	EtOHnc2	Galnc1	Galnc2
13	ABF2	YMR072W	YPDcl2	YPDcl3	EtOHcl1	EtOHcl2	Galcl1	YPDnc1	YPDnc2	YPDnc3	YPDnc4	EtOHnc1	EtOHnc2	Galnc1	Galnc2
13	DNF1	YER166W	YPDcl2	YPDcl3	EtOHcl1	EtOHcl2	Galcl1	YPDnc1	YPDnc2	YPDnc3	YPDnc4	EtOHnc1	EtOHnc2	Galnc1	Galnc2
13		YAL026C-A	YPDcl2	YPDcl3	EtOHcl1	EtOHcl2	Galcl1	YPDnc1	YPDnc2	YPDnc3	YPDnc4	EtOHnc1	EtOHnc2	Galnc1	Galnc2
13		YLR163W-	YPDcl2	YPDcl3	EtOHcl1	EtOHcl2	Galcl1	YPDnc1	YPDnc2	YPDnc3	YPDnc4	EtOHnc1	EtOHnc2	Galnc1	Galnc2
13	AHC2	YCR082W	YPDcl2	YPDcl3	EtOHcl1	EtOHcl2	Galcl1	YPDnc1	YPDnc2	YPDnc3	YPDnc4	EtOHnc1	EtOHnc2	Galnc1	Galnc2
13	ADR1	YDR216W	YPDcl2	YPDcl3	EtOHcl1	EtOHcl2	Galcl1	YPDnc1	YPDnc2	YPDnc3	YPDnc4	EtOHnc1	EtOHnc2	Galnc1	Galnc2
13	RHO2	YNL090W	YPDcl2	YPDcl3	EtOHcl1	EtOHcl2	Galcl1	YPDnc1	YPDnc2	YPDnc3	YPDnc4	EtOHnc1	EtOHnc2	Galnc1	Galnc2
13	UBP16	YPL072W	YPDcl2	YPDcl3	EtOHcl1	EtOHcl2	Galcl1	YPDnc1	YPDnc2	YPDnc3	YPDnc4	EtOHnc1	EtOHnc2	Galnc1	Galnc2
13	MIP1	YOR330C	YPDcl2	YPDcl3	EtOHcl1	EtOHcl2	Galcl1	YPDnc1	YPDnc2	YPDnc3	YPDnc4	EtOHnc1	EtOHnc2	Galnc1	Galnc2
13	SFB2	YNL049C	YPDcl2	YPDcl3	EtOHcl1	EtOHcl2	Galcl1	YPDnc1	YPDnc2	YPDnc3	YPDnc4	EtOHnc1	EtOHnc2	Galnc1	Galnc2
13	GEP7	YGL057C	YPDcl2	YPDcl3	EtOHcl1	EtOHcl2	Galcl1	YPDnc1	YPDnc2	YPDnc3	YPDnc4	EtOHnc1	EtOHnc2	Galnc1	Galnc2
13		YNL108C	YPDcl2	YPDcl3	EtOHcl1	EtOHcl2	Galcl1	YPDnc1	YPDnc2	YPDnc3	YPDnc4	EtOHnc1	EtOHnc2	Galnc1	Galnc2
13	GDA1	YEL042W	YPDcl2	YPDcl3	EtOHcl1	EtOHcl2	Galcl1	YPDnc1	YPDnc2	YPDnc3	YPDnc4	EtOHnc1	EtOHnc2	Galnc1	Galnc2
13		YJR018W	YPDcl2	YPDcl3	EtOHcl1	EtOHcl2	Galcl1	YPDnc1	YPDnc2	YPDnc3	YPDnc4	EtOHnc1	EtOHnc2	Galnc1	Galnc2
13	MSC3	YLR219W	YPDcl2	YPDcl3	EtOHcl1	EtOHcl2	Galcl1	YPDnc1	YPDnc2	YPDnc3	YPDnc4	EtOHnc1	EtOHnc2	Galnc1	Galnc2
13	SRV2	YNL138W	YPDcl2	YPDcl3	EtOHcl1	EtOHcl2	Galcl1	YPDnc1	YPDnc2	YPDnc3	YPDnc4	EtOHnc1	EtOHnc2	Galnc1	Galnc2
13	UBC4	YBR082C	YPDcl2	YPDcl3	EtOHcl1	EtOHcl2	Galcl1	YPDnc1	YPDnc2	YPDnc3	YPDnc4	EtOHnc1	EtOHnc2	Galnc1	Galnc2
13	KEM1	YGL173C	YPDcl2	YPDcl3	EtOHcl1	EtOHcl2	Galcl1	YPDnc1	YPDnc2	YPDnc3	YPDnc4	EtOHnc1	EtOHnc2	Galnc1	Galnc2
13		YOR072W-	YPDcl2	YPDcl3	EtOHcl1	EtOHcl2	Galcl1	YPDnc1	YPDnc2	YPDnc3	YPDnc4	EtOHnc1	EtOHnc2	Galnc1	Galnc2
13	ILV5	YLR355C	YPDcl2	YPDcl3	EtOHcl1	EtOHcl2	Galcl1	YPDnc1	YPDnc2	YPDnc3	YPDnc4	EtOHnc1	EtOHnc2	Galnc1	Galnc2
13	LEU5	YHR002W	YPDcl2	YPDcl3	EtOHcl1	EtOHcl2	Galcl1	YPDnc1	YPDnc2	YPDnc3	YPDnc4	EtOHnc1	EtOHnc2	Galnc1	Galnc2
13	MET31	YPL038W	YPDcl2	YPDcl3	EtOHcl1	EtOHcl2	Galcl1	YPDnc1	YPDnc2	YPDnc3	YPDnc4	EtOHnc1	EtOHnc2	Galnc1	Galnc2
13	OXR1	YPL196W	YPDcl2	YPDcl3	EtOHcl1	EtOHcl2	Galcl1	YPDnc1	YPDnc2	YPDnc3	YPDnc4	EtOHnc1	EtOHnc2	Galnc1	Galnc2
13		YER088W-	YPDcl2	YPDcl3	EtOHcl1	EtOHcl2	Galcl1	YPDnc1	YPDnc2	YPDnc3	YPDnc4	EtOHnc1	EtOHnc2	Galnc1	Galnc2
13	BPH1	YCR032W	YPDcl2	YPDcl3	EtOHcl1	EtOHcl2	Galcl1	YPDnc1	YPDnc2	YPDnc3	YPDnc4	EtOHnc1	EtOHnc2	Galnc1	Galnc2
13		YJL015C	YPDcl2	YPDcl3	EtOHcl1	EtOHcl2	Galcl1	YPDnc1	YPDnc2	YPDnc3	YPDnc4	EtOHnc1	EtOHnc2	Galnc1	Galnc2
13		YIL169C	YPDcl2	YPDcl3	EtOHcl1	EtOHcl2	Galcl1	YPDnc1	YPDnc2	YPDnc3	YPDnc4	EtOHnc1	EtOHnc2	Galnc1	Galnc2
13	RMT2	YDR465C	YPDcl2	YPDcl3	EtOHcl1	EtOHcl2	Galcl1	YPDnc1	YPDnc2	YPDnc3	YPDnc4	EtOHnc1	EtOHnc2	Galnc1	Galnc2
13	HIS4	YCL030C	YPDcl2	YPDcl3	EtOHcl1	EtOHcl2	Galcl1	YPDnc1	YPDnc2	YPDnc3	YPDnc4	EtOHnc1	EtOHnc2	Galnc1	Galnc2
13	ECI1	YLR284C	YPDcl2	YPDcl3	EtOHcl1	EtOHcl2	Galcl1	YPDnc1	YPDnc2	YPDnc3	YPDnc4	EtOHnc1	EtOHnc2	Galnc1	Galnc2

13	RAD16	YBR114W	YPDcl2	YPDcl3	EtOHcl1	EtOHcl2	Galcl1	YPDnc1	YPDnc2	YPDnc3	YPDnc4	EtOHnc1	EtOHnc2	Galnc1	Galnc2
13	PET127	YOR017W	YPDcl2	YPDcl3	EtOHcl1	EtOHcl2	Galcl1	YPDnc1	YPDnc2	YPDnc3	YPDnc4	EtOHnc1	EtOHnc2	Galnc1	Galnc2
13		YML099W-	YPDcl2	YPDcl3	EtOHcl1	EtOHcl2	Galcl1	YPDnc1	YPDnc2	YPDnc3	YPDnc4	EtOHnc1	EtOHnc2	Galnc1	Galnc2
13	PRK1	YIL095W	YPDcl2	YPDcl3	EtOHcl1	EtOHcl2	Galcl1	YPDnc1	YPDnc2	YPDnc3	YPDnc4	EtOHnc1	EtOHnc2	Galnc1	Galnc2
13	YSY6	YBR162W-/	YPDcl2	YPDcl3	EtOHcl1	EtOHcl2	Galcl1	YPDnc1	YPDnc2	YPDnc3	YPDnc4	EtOHnc1	EtOHnc2	Galnc1	Galnc2
13	NUP157	YER105C	YPDcl2	YPDcl3	EtOHcl1	EtOHcl2	Galcl1	YPDnc1	YPDnc2	YPDnc3	YPDnc4	EtOHnc1	EtOHnc2	Galnc1	Galnc2
13	GAL2	YLR081W	YPDcl2	YPDcl3	EtOHcl1	EtOHcl2	Galcl1	YPDnc1	YPDnc2	YPDnc3	YPDnc4	EtOHnc1	EtOHnc2	Galnc1	Galnc2
13		YEL053W-A	YPDcl2	YPDcl3	EtOHcl1	EtOHcl2	Galcl1	YPDnc1	YPDnc2	YPDnc3	YPDnc4	EtOHnc1	EtOHnc2	Galnc1	Galnc2
13	RPO41	YFL036W	YPDcl2	YPDcl3	EtOHcl1	EtOHcl2	Galcl1	YPDnc1	YPDnc2	YPDnc3	YPDnc4	EtOHnc1	EtOHnc2	Galnc1	Galnc2
13	RAD27	YKL113C	YPDcl2	YPDcl3	EtOHcl1	EtOHcl2	Galcl1	YPDnc1	YPDnc2	YPDnc3	YPDnc4	EtOHnc1	EtOHnc2	Galnc1	Galnc2
13	TRM3	YDL112W	YPDcl2	YPDcl3	EtOHcl1	EtOHcl2	Galcl1	YPDnc1	YPDnc2	YPDnc3	YPDnc4	EtOHnc1	EtOHnc2	Galnc1	Galnc2
13		YDL228C	YPDcl2	YPDcl3	EtOHcl1	EtOHcl2	Galcl1	YPDnc1	YPDnc2	YPDnc3	YPDnc4	EtOHnc1	EtOHnc2	Galnc1	Galnc2
13	TPA1	YER049W	YPDcl2	YPDcl3	EtOHcl1	EtOHcl2	Galcl1	YPDnc1	YPDnc2	YPDnc3	YPDnc4	EtOHnc1	EtOHnc2	Galnc1	Galnc2
13		YDR179W-/	YPDcl2	YPDcl3	EtOHcl1	EtOHcl2	Galcl1	YPDnc1	YPDnc2	YPDnc3	YPDnc4	EtOHnc1	EtOHnc2	Galnc1	Galnc2
13	SSQ1	YLR369W	YPDcl2	YPDcl3	EtOHcl1	EtOHcl2	Galcl1	YPDnc1	YPDnc2	YPDnc3	YPDnc4	EtOHnc1	EtOHnc2	Galnc1	Galnc2
13		YJL171C	YPDcl2	YPDcl3	EtOHcl1	EtOHcl2	Galcl1	YPDnc1	YPDnc2	YPDnc3	YPDnc4	EtOHnc1	EtOHnc2	Galnc1	Galnc2
13	SAF1	YBR280C	YPDcl2	YPDcl3	EtOHcl1	EtOHcl2	Galcl1	YPDnc1	YPDnc2	YPDnc3	YPDnc4	EtOHnc1	EtOHnc2	Galnc1	Galnc2
13		YBR259W	YPDcl2	YPDcl3	EtOHcl1	EtOHcl2	Galcl1	YPDnc1	YPDnc2	YPDnc3	YPDnc4	EtOHnc1	EtOHnc2	Galnc1	Galnc2
13		YML084W	YPDcl2	YPDcl3	EtOHcl1	EtOHcl2	Galcl1	YPDnc1	YPDnc2	YPDnc3	YPDnc4	EtOHnc1	EtOHnc2	Galnc1	Galnc2
13	GTS1	YGL181W	YPDcl2	YPDcl3	EtOHcl1	EtOHcl2	Galcl1	YPDnc1	YPDnc2	YPDnc3	YPDnc4	EtOHnc1	EtOHnc2	Galnc1	Galnc2
13	BCD1	YHR040W	YPDcl2	YPDcl3	EtOHcl1	EtOHcl2	Galcl1	YPDnc1	YPDnc2	YPDnc3	YPDnc4	EtOHnc1	EtOHnc2	Galnc1	Galnc2
13	FLO10	YKR102W	YPDcl2	YPDcl3	EtOHcl1	EtOHcl2	Galcl1	YPDnc1	YPDnc2	YPDnc3	YPDnc4	EtOHnc1	EtOHnc2	Galnc1	Galnc2
13	RRN9	YMR270C	YPDcl2	YPDcl3	EtOHcl1	EtOHcl2	Galcl1	YPDnc1	YPDnc2	YPDnc3	YPDnc4	EtOHnc1	EtOHnc2	Galnc1	Galnc2
13	JIP4	YDR475C	YPDcl2	YPDcl3	EtOHcl1	EtOHcl2	Galcl1	YPDnc1	YPDnc2	YPDnc3	YPDnc4	EtOHnc1	EtOHnc2	Galnc1	Galnc2
13		YJR023C	YPDcl2	YPDcl3	EtOHcl1	EtOHcl2	Galcl1	YPDnc1	YPDnc2	YPDnc3	YPDnc4	EtOHnc1	EtOHnc2	Galnc1	Galnc2
13	GCS1	YDL226C	YPDcl2	YPDcl3	EtOHcl1	EtOHcl2	Galcl1	YPDnc1	YPDnc2	YPDnc3	YPDnc4	EtOHnc1	EtOHnc2	Galnc1	Galnc2
13	UTP4	YDR324C	YPDcl2	YPDcl3	EtOHcl1	EtOHcl2	Galcl1	YPDnc1	YPDnc2	YPDnc3	YPDnc4	EtOHnc1	EtOHnc2	Galnc1	Galnc2
13		YER128W	YPDcl2	YPDcl3	EtOHcl1	EtOHcl2	Galcl1	YPDnc1	YPDnc2	YPDnc3	YPDnc4	EtOHnc1	EtOHnc2	Galnc1	Galnc2
13	YIP4	YGL198W	YPDcl2	YPDcl3	EtOHcl1	EtOHcl2	Galcl1	YPDnc1	YPDnc2	YPDnc3	YPDnc4	EtOHnc1	EtOHnc2	Galnc1	Galnc2
13	SEC14	YMR079W	YPDcl2	YPDcl3	EtOHcl1	EtOHcl2	Galcl1	YPDnc1	YPDnc2	YPDnc3	YPDnc4	EtOHnc1	EtOHnc2	Galnc1	Galnc2
13	FUN26	YAL022C	YPDcl2	YPDcl3	EtOHcl1	EtOHcl2	Galcl1	YPDnc1	YPDnc2	YPDnc3	YPDnc4	EtOHnc1	EtOHnc2	Galnc1	Galnc2
13	RPF2	YKR081C	YPDcl2	YPDcl3	EtOHcl1	EtOHcl2	Galcl1	YPDnc1	YPDnc2	YPDnc3	YPDnc4	EtOHnc1	EtOHnc2	Galnc1	Galnc2
13	CUE1	YMR264W	YPDcl2	YPDcl3	EtOHcl1	EtOHcl2	Galcl1	YPDnc1	YPDnc2	YPDnc3	YPDnc4	EtOHnc1	EtOHnc2	Galnc1	Galnc2
13	DUS3	YLR401C	YPDcl2	YPDcl3	EtOHcl1	EtOHcl2	Galcl1	YPDnc1	YPDnc2	YPDnc3	YPDnc4	EtOHnc1	EtOHnc2	Galnc1	Galnc2

13	UBS1	YBR165W	YPDcl2	YPDcl3	EtOHcl1	EtOHcl2	Galcl1	YPDnc1	YPDnc2	YPDnc3	YPDnc4	EtOHnc1	EtOHnc2	Galnc1	Galnc2
13		YOL046C	YPDcl2	YPDcl3	EtOHcl1	EtOHcl2	Galcl1	YPDnc1	YPDnc2	YPDnc3	YPDnc4	EtOHnc1	EtOHnc2	Galnc1	Galnc2
13	KRE27	YIL027C	YPDcl2	YPDcl3	EtOHcl1	EtOHcl2	Galcl1	YPDnc1	YPDnc2	YPDnc3	YPDnc4	EtOHnc1	EtOHnc2	Galnc1	Galnc2
13	MSO1	YNR049C	YPDcl2	YPDcl3	EtOHcl1	EtOHcl2	Galcl1	YPDnc1	YPDnc2	YPDnc3	YPDnc4	EtOHnc1	EtOHnc2	Galnc1	Galnc2
13	GYP5	YPL249C	YPDcl2	YPDcl3	EtOHcl1	EtOHcl2	Galcl1	YPDnc1	YPDnc2	YPDnc3	YPDnc4	EtOHnc1	EtOHnc2	Galnc1	Galnc2
13	BEM2	YER155C	YPDcl2	YPDcl3	EtOHcl1	EtOHcl2	Galcl1	YPDnc1	YPDnc2	YPDnc3	YPDnc4	EtOHnc1	EtOHnc2	Galnc1	Galnc2
13	ACE2	YLR131C	YPDcl2	YPDcl3	EtOHcl1	EtOHcl2	Galcl1	YPDnc1	YPDnc2	YPDnc3	YPDnc4	EtOHnc1	EtOHnc2	Galnc1	Galnc2
13	SNC1	YAL030W	YPDcl2	YPDcl3	EtOHcl1	EtOHcl2	Galcl1	YPDnc1	YPDnc2	YPDnc3	YPDnc4	EtOHnc1	EtOHnc2	Galnc1	Galnc2
13	BUD14	YAR014C	YPDcl2	YPDcl3	EtOHcl1	EtOHcl2	Galcl1	YPDnc1	YPDnc2	YPDnc3	YPDnc4	EtOHnc1	EtOHnc2	Galnc1	Galnc2
13		YER156C	YPDcl2	YPDcl3	EtOHcl1	EtOHcl2	Galcl1	YPDnc1	YPDnc2	YPDnc3	YPDnc4	EtOHnc1	EtOHnc2	Galnc1	Galnc2
13		YBR238C	YPDcl2	YPDcl3	EtOHcl1	EtOHcl2	Galcl1	YPDnc1	YPDnc2	YPDnc3	YPDnc4	EtOHnc1	EtOHnc2	Galnc1	Galnc2
13		YIL156W-A	YPDcl2	YPDcl3	EtOHcl1	EtOHcl2	Galcl1	YPDnc1	YPDnc2	YPDnc3	YPDnc4	EtOHnc1	EtOHnc2	Galnc1	Galnc2
13	STF1	YDL130W-A	YPDcl2	YPDcl3	EtOHcl1	EtOHcl2	Galcl1	YPDnc1	YPDnc2	YPDnc3	YPDnc4	EtOHnc1	EtOHnc2	Galnc1	Galnc2
13	EST1	YLR233C	YPDcl2	YPDcl3	EtOHcl1	EtOHcl2	Galcl1	YPDnc1	YPDnc2	YPDnc3	YPDnc4	EtOHnc1	EtOHnc2	Galnc1	Galnc2
13		YCL046W	YPDcl2	YPDcl3	EtOHcl1	EtOHcl2	Galcl1	YPDnc1	YPDnc2	YPDnc3	YPDnc4	EtOHnc1	EtOHnc2	Galnc1	Galnc2
13	HHO1	YPL127C	YPDcl2	YPDcl3	EtOHcl1	EtOHcl2	Galcl1	YPDnc1	YPDnc2	YPDnc3	YPDnc4	EtOHnc1	EtOHnc2	Galnc1	Galnc2
13		YPL077C	YPDcl2	YPDcl3	EtOHcl1	EtOHcl2	Galcl1	YPDnc1	YPDnc2	YPDnc3	YPDnc4	EtOHnc1	EtOHnc2	Galnc1	Galnc2
13	AIM36	YMR157C	YPDcl2	YPDcl3	EtOHcl1	EtOHcl2	Galcl1	YPDnc1	YPDnc2	YPDnc3	YPDnc4	EtOHnc1	EtOHnc2	Galnc1	Galnc2
13	ALG6	YOR002W	YPDcl2	YPDcl3	EtOHcl1	EtOHcl2	Galcl1	YPDnc1	YPDnc2	YPDnc3	YPDnc4	EtOHnc1	EtOHnc2	Galnc1	Galnc2
13	ECM5	YMR176W	YPDcl2	YPDcl3	EtOHcl1	EtOHcl2	Galcl1	YPDnc1	YPDnc2	YPDnc3	YPDnc4	EtOHnc1	EtOHnc2	Galnc1	Galnc2
13	SWI4	YER111C	YPDcl2	YPDcl3	EtOHcl1	EtOHcl2	Galcl1	YPDnc1	YPDnc2	YPDnc3	YPDnc4	EtOHnc1	EtOHnc2	Galnc1	Galnc2
13		YGL182C	YPDcl2	YPDcl3	EtOHcl1	EtOHcl2	Galcl1	YPDnc1	YPDnc2	YPDnc3	YPDnc4	EtOHnc1	EtOHnc2	Galnc1	Galnc2
13	ALE1	YOR175C	YPDcl2	YPDcl3	EtOHcl1	EtOHcl2	Galcl1	YPDnc1	YPDnc2	YPDnc3	YPDnc4	EtOHnc1	EtOHnc2	Galnc1	Galnc2
13	BDH2	YAL061W	YPDcl2	YPDcl3	EtOHcl1	EtOHcl2	Galcl1	YPDnc1	YPDnc2	YPDnc3	YPDnc4	EtOHnc1	EtOHnc2	Galnc1	Galnc2
13	RSM27	YGR215W	YPDcl2	YPDcl3	EtOHcl1	EtOHcl2	Galcl1	YPDnc1	YPDnc2	YPDnc3	YPDnc4	EtOHnc1	EtOHnc2	Galnc1	Galnc2
13		YKL161C	YPDcl2	YPDcl3	EtOHcl1	EtOHcl2	Galcl1	YPDnc1	YPDnc2	YPDnc3	YPDnc4	EtOHnc1	EtOHnc2	Galnc1	Galnc2
13	SLD5	YDR489W	YPDcl2	YPDcl3	EtOHcl1	EtOHcl2	Galcl1	YPDnc1	YPDnc2	YPDnc3	YPDnc4	EtOHnc1	EtOHnc2	Galnc1	Galnc2
13		YML096W	YPDcl2	YPDcl3	EtOHcl1	EtOHcl2	Galcl1	YPDnc1	YPDnc2	YPDnc3	YPDnc4	EtOHnc1	EtOHnc2	Galnc1	Galnc2
13	UTP10	YJL109C	YPDcl2	YPDcl3	EtOHcl1	EtOHcl2	Galcl1	YPDnc1	YPDnc2	YPDnc3	YPDnc4	EtOHnc1	EtOHnc2	Galnc1	Galnc2
13	TOS3	YGL179C	YPDcl2	YPDcl3	EtOHcl1	EtOHcl2	Galcl1	YPDnc1	YPDnc2	YPDnc3	YPDnc4	EtOHnc1	EtOHnc2	Galnc1	Galnc2
13		YGL235W	YPDcl2	YPDcl3	EtOHcl1	EtOHcl2	Galcl1	YPDnc1	YPDnc2	YPDnc3	YPDnc4	EtOHnc1	EtOHnc2	Galnc1	Galnc2
13	YTA12	YMR089C	YPDcl2	YPDcl3	EtOHcl1	EtOHcl2	Galcl1	YPDnc1	YPDnc2	YPDnc3	YPDnc4	EtOHnc1	EtOHnc2	Galnc1	Galnc2
13	BUB1	YGR188C	YPDcl2	YPDcl3	EtOHcl1	EtOHcl2	Galcl1	YPDnc1	YPDnc2	YPDnc3	YPDnc4	EtOHnc1	EtOHnc2	Galnc1	Galnc2
13	PSK2	YOL045W	YPDcl2	YPDcl3	EtOHcl1	EtOHcl2	Galcl1	YPDnc1	YPDnc2	YPDnc3	YPDnc4	EtOHnc1	EtOHnc2	Galnc1	Galnc2

13	TYW1	YPL207W	YPDcl2	YPDcl3	EtOHcl1	EtOHcl2	Galcl1	YPDnc1	YPDnc2	YPDnc3	YPDnc4	EtOHnc1	EtOHnc2	Galnc1	Galnc2
13	CNB1	YKL190W	YPDcl2	YPDcl3	EtOHcl1	EtOHcl2	Galcl1	YPDnc1	YPDnc2	YPDnc3	YPDnc4	EtOHnc1	EtOHnc2	Galnc1	Galnc2
13	KIN82	YCR091W	YPDcl2	YPDcl3	EtOHcl1	EtOHcl2	Galcl1	YPDnc1	YPDnc2	YPDnc3	YPDnc4	EtOHnc1	EtOHnc2	Galnc1	Galnc2
13	RPG1	YBR079C	YPDcl2	YPDcl3	EtOHcl1	EtOHcl2	Galcl1	YPDnc1	YPDnc2	YPDnc3	YPDnc4	EtOHnc1	EtOHnc2	Galnc1	Galnc2
13	KEX1	YGL203C	YPDcl2	YPDcl3	EtOHcl1	EtOHcl2	Galcl1	YPDnc1	YPDnc2	YPDnc3	YPDnc4	EtOHnc1	EtOHnc2	Galnc1	Galnc2
13	PRM5	YIL117C	YPDcl2	YPDcl3	EtOHcl1	EtOHcl2	Galcl1	YPDnc1	YPDnc2	YPDnc3	YPDnc4	EtOHnc1	EtOHnc2	Galnc1	Galnc2
13	NMD2	YHR077C	YPDcl2	YPDcl3	EtOHcl1	EtOHcl2	Galcl1	YPDnc1	YPDnc2	YPDnc3	YPDnc4	EtOHnc1	EtOHnc2	Galnc1	Galnc2
13	FRE7	YOL152W	YPDcl2	YPDcl3	EtOHcl1	EtOHcl2	Galcl1	YPDnc1	YPDnc2	YPDnc3	YPDnc4	EtOHnc1	EtOHnc2	Galnc1	Galnc2
13		YDL240C-A	YPDcl2	YPDcl3	EtOHcl1	EtOHcl2	Galcl1	YPDnc1	YPDnc2	YPDnc3	YPDnc4	EtOHnc1	EtOHnc2	Galnc1	Galnc2
13	MNS1	YJR131W	YPDcl2	YPDcl3	EtOHcl1	EtOHcl2	Galcl1	YPDnc1	YPDnc2	YPDnc3	YPDnc4	EtOHnc1	EtOHnc2	Galnc1	Galnc2
13	COX16	YJL003W	YPDcl2	YPDcl3	EtOHcl1	EtOHcl2	Galcl1	YPDnc1	YPDnc2	YPDnc3	YPDnc4	EtOHnc1	EtOHnc2	Galnc1	Galnc2
13	PMS1	YNL082W	YPDcl2	YPDcl3	EtOHcl1	EtOHcl2	Galcl1	YPDnc1	YPDnc2	YPDnc3	YPDnc4	EtOHnc1	EtOHnc2	Galnc1	Galnc2
13	CAB5	YDR196C	YPDcl2	YPDcl3	EtOHcl1	EtOHcl2	Galcl1	YPDnc1	YPDnc2	YPDnc3	YPDnc4	EtOHnc1	EtOHnc2	Galnc1	Galnc2
13		YLR286W-A	YPDcl2	YPDcl3	EtOHcl1	EtOHcl2	Galcl1	YPDnc1	YPDnc2	YPDnc3	YPDnc4	EtOHnc1	EtOHnc2	Galnc1	Galnc2
13	SEC31	YDL195W	YPDcl2	YPDcl3	EtOHcl1	EtOHcl2	Galcl1	YPDnc1	YPDnc2	YPDnc3	YPDnc4	EtOHnc1	EtOHnc2	Galnc1	Galnc2
13	SYT1	YPR095C	YPDcl2	YPDcl3	EtOHcl1	EtOHcl2	Galcl1	YPDnc1	YPDnc2	YPDnc3	YPDnc4	EtOHnc1	EtOHnc2	Galnc1	Galnc2
13	SUM1	YDR310C	YPDcl2	YPDcl3	EtOHcl1	EtOHcl2	Galcl1	YPDnc1	YPDnc2	YPDnc3	YPDnc4	EtOHnc1	EtOHnc2	Galnc1	Galnc2
13	DGA1	YOR245C	YPDcl2	YPDcl3	EtOHcl1	EtOHcl2	Galcl1	YPDnc1	YPDnc2	YPDnc3	YPDnc4	EtOHnc1	EtOHnc2	Galnc1	Galnc2
13		YPL102C	YPDcl2	YPDcl3	EtOHcl1	EtOHcl2	Galcl1	YPDnc1	YPDnc2	YPDnc3	YPDnc4	EtOHnc1	EtOHnc2	Galnc1	Galnc2
13		YOR335W-A	YPDcl2	YPDcl3	EtOHcl1	EtOHcl2	Galcl1	YPDnc1	YPDnc2	YPDnc3	YPDnc4	EtOHnc1	EtOHnc2	Galnc1	Galnc2
13	THI73	YLR004C	YPDcl2	YPDcl3	EtOHcl1	EtOHcl2	Galcl1	YPDnc1	YPDnc2	YPDnc3	YPDnc4	EtOHnc1	EtOHnc2	Galnc1	Galnc2
13		YDL241W	YPDcl2	YPDcl3	EtOHcl1	EtOHcl2	Galcl1	YPDnc1	YPDnc2	YPDnc3	YPDnc4	EtOHnc1	EtOHnc2	Galnc1	Galnc2
13	TRF5	YNL299W	YPDcl2	YPDcl3	EtOHcl1	EtOHcl2	Galcl1	YPDnc1	YPDnc2	YPDnc3	YPDnc4	EtOHnc1	EtOHnc2	Galnc1	Galnc2
13	IKS1	YJL057C	YPDcl2	YPDcl3	EtOHcl1	EtOHcl2	Galcl1	YPDnc1	YPDnc2	YPDnc3	YPDnc4	EtOHnc1	EtOHnc2	Galnc1	Galnc2
13	NOP8	YOL144W	YPDcl2	YPDcl3	EtOHcl1	EtOHcl2	Galcl1	YPDnc1	YPDnc2	YPDnc3	YPDnc4	EtOHnc1	EtOHnc2	Galnc1	Galnc2
13	BUD28	YLR062C	YPDcl2	YPDcl3	EtOHcl1	EtOHcl2	Galcl1	YPDnc1	YPDnc2	YPDnc3	YPDnc4	EtOHnc1	EtOHnc2	Galnc1	Galnc2
13	ATG22	YCL038C	YPDcl2	YPDcl3	EtOHcl1	EtOHcl2	Galcl1	YPDnc1	YPDnc2	YPDnc3	YPDnc4	EtOHnc1	EtOHnc2	Galnc1	Galnc2
13		YFR036W-A	YPDcl2	YPDcl3	EtOHcl1	EtOHcl2	Galcl1	YPDnc1	YPDnc2	YPDnc3	YPDnc4	EtOHnc1	EtOHnc2	Galnc1	Galnc2
13	GLE1	YDL207W	YPDcl2	YPDcl3	EtOHcl1	EtOHcl2	Galcl1	YPDnc1	YPDnc2	YPDnc3	YPDnc4	EtOHnc1	EtOHnc2	Galnc1	Galnc2
13	ARG81	YML099C	YPDcl2	YPDcl3	EtOHcl1	EtOHcl2	Galcl1	YPDnc1	YPDnc2	YPDnc3	YPDnc4	EtOHnc1	EtOHnc2	Galnc1	Galnc2
13		YAR019W-A	YPDcl2	YPDcl3	EtOHcl1	EtOHcl2	Galcl1	YPDnc1	YPDnc2	YPDnc3	YPDnc4	EtOHnc1	EtOHnc2	Galnc1	Galnc2
13		YCR006C	YPDcl2	YPDcl3	EtOHcl1	EtOHcl2	Galcl1	YPDnc1	YPDnc2	YPDnc3	YPDnc4	EtOHnc1	EtOHnc2	Galnc1	Galnc2
13	FRA2	YGL220W	YPDcl2	YPDcl3	EtOHcl1	EtOHcl2	Galcl1	YPDnc1	YPDnc2	YPDnc3	YPDnc4	EtOHnc1	EtOHnc2	Galnc1	Galnc2
13		YLR042C	YPDcl2	YPDcl3	EtOHcl1	EtOHcl2	Galcl1	YPDnc1	YPDnc2	YPDnc3	YPDnc4	EtOHnc1	EtOHnc2	Galnc1	Galnc2

13	AIM11	YER093C-A	YPDcl2	YPDcl3	EtOHcl1	EtOHcl2	Galcl1	YPDnc1	YPDnc2	YPDnc3	YPDnc4	EtOHnc1	EtOHnc2	Galnc1	Galnc2
13	TUS1	YLR425W	YPDcl2	YPDcl3	EtOHcl1	EtOHcl2	Galcl1	YPDnc1	YPDnc2	YPDnc3	YPDnc4	EtOHnc1	EtOHnc2	Galnc1	Galnc2
13	TUF1	YOR187W	YPDcl2	YPDcl3	EtOHcl1	EtOHcl2	Galcl1	YPDnc1	YPDnc2	YPDnc3	YPDnc4	EtOHnc1	EtOHnc2	Galnc1	Galnc2
13	YAF9	YNL107W	YPDcl2	YPDcl3	EtOHcl1	EtOHcl2	Galcl1	YPDnc1	YPDnc2	YPDnc3	YPDnc4	EtOHnc1	EtOHnc2	Galnc1	Galnc2
13	YGP1	YNL160W	YPDcl2	YPDcl3	EtOHcl1	EtOHcl2	Galcl1	YPDnc1	YPDnc2	YPDnc3	YPDnc4	EtOHnc1	EtOHnc2	Galnc1	Galnc2
13	ANP1	YEL036C	YPDcl2	YPDcl3	EtOHcl1	EtOHcl2	Galcl1	YPDnc1	YPDnc2	YPDnc3	YPDnc4	EtOHnc1	EtOHnc2	Galnc1	Galnc2
13	TIM54	YJL054W	YPDcl2	YPDcl3	EtOHcl1	EtOHcl2	Galcl1	YPDnc1	YPDnc2	YPDnc3	YPDnc4	EtOHnc1	EtOHnc2	Galnc1	Galnc2
13	SCJ1	YMR214W	YPDcl2	YPDcl3	EtOHcl1	EtOHcl2	Galcl1	YPDnc1	YPDnc2	YPDnc3	YPDnc4	EtOHnc1	EtOHnc2	Galnc1	Galnc2
13	UBX5	YDR330W	YPDcl2	YPDcl3	EtOHcl1	EtOHcl2	Galcl1	YPDnc1	YPDnc2	YPDnc3	YPDnc4	EtOHnc1	EtOHnc2	Galnc1	Galnc2
13	AME1	YBR211C	YPDcl2	YPDcl3	EtOHcl1	EtOHcl2	Galcl1	YPDnc1	YPDnc2	YPDnc3	YPDnc4	EtOHnc1	EtOHnc2	Galnc1	Galnc2
13	LRO1	YNR008W	YPDcl2	YPDcl3	EtOHcl1	EtOHcl2	Galcl1	YPDnc1	YPDnc2	YPDnc3	YPDnc4	EtOHnc1	EtOHnc2	Galnc1	Galnc2
13	CBT1	YKL208W	YPDcl2	YPDcl3	EtOHcl1	EtOHcl2	Galcl1	YPDnc1	YPDnc2	YPDnc3	YPDnc4	EtOHnc1	EtOHnc2	Galnc1	Galnc2
13	HNM1	YGL077C	YPDcl2	YPDcl3	EtOHcl1	EtOHcl2	Galcl1	YPDnc1	YPDnc2	YPDnc3	YPDnc4	EtOHnc1	EtOHnc2	Galnc1	Galnc2
13	SWI6	YLR182W	YPDcl2	YPDcl3	EtOHcl1	EtOHcl2	Galcl1	YPDnc1	YPDnc2	YPDnc3	YPDnc4	EtOHnc1	EtOHnc2	Galnc1	Galnc2
13	CDC15	YAR019C	YPDcl2	YPDcl3	EtOHcl1	EtOHcl2	Galcl1	YPDnc1	YPDnc2	YPDnc3	YPDnc4	EtOHnc1	EtOHnc2	Galnc1	Galnc2
13	TOM70	YNL121C	YPDcl2	YPDcl3	EtOHcl1	EtOHcl2	Galcl1	YPDnc1	YPDnc2	YPDnc3	YPDnc4	EtOHnc1	EtOHnc2	Galnc1	Galnc2
13	PSE1	YMR308C	YPDcl2	YPDcl3	EtOHcl1	EtOHcl2	Galcl1	YPDnc1	YPDnc2	YPDnc3	YPDnc4	EtOHnc1	EtOHnc2	Galnc1	Galnc2
13	ECM17	YJR137C	YPDcl2	YPDcl3	EtOHcl1	EtOHcl2	Galcl1	YPDnc1	YPDnc2	YPDnc3	YPDnc4	EtOHnc1	EtOHnc2	Galnc1	Galnc2
13	NIP1	YMR309C	YPDcl2	YPDcl3	EtOHcl1	EtOHcl2	Galcl1	YPDnc1	YPDnc2	YPDnc3	YPDnc4	EtOHnc1	EtOHnc2	Galnc1	Galnc2
13	ALD2	YMR170C	YPDcl2	YPDcl3	EtOHcl1	EtOHcl2	Galcl1	YPDnc1	YPDnc2	YPDnc3	YPDnc4	EtOHnc1	EtOHnc2	Galnc1	Galnc2
13	NHP2	YDL208W	YPDcl2	YPDcl3	EtOHcl1	EtOHcl2	Galcl1	YPDnc1	YPDnc2	YPDnc3	YPDnc4	EtOHnc1	EtOHnc2	Galnc1	Galnc2
13	KTR1	YOR099W	YPDcl2	YPDcl3	EtOHcl1	EtOHcl2	Galcl1	YPDnc1	YPDnc2	YPDnc3	YPDnc4	EtOHnc1	EtOHnc2	Galnc1	Galnc2
13	PTA1	YAL043C	YPDcl2	YPDcl3	EtOHcl1	EtOHcl2	Galcl1	YPDnc1	YPDnc2	YPDnc3	YPDnc4	EtOHnc1	EtOHnc2	Galnc1	Galnc2
13		YBR028C	YPDcl2	YPDcl3	EtOHcl1	EtOHcl2	Galcl1	YPDnc1	YPDnc2	YPDnc3	YPDnc4	EtOHnc1	EtOHnc2	Galnc1	Galnc2
13	LAP4	YKL103C	YPDcl2	YPDcl3	EtOHcl1	EtOHcl2	Galcl1	YPDnc1	YPDnc2	YPDnc3	YPDnc4	EtOHnc1	EtOHnc2	Galnc1	Galnc2
13	CNM67	YNL225C	YPDcl2	YPDcl3	EtOHcl1	EtOHcl2	Galcl1	YPDnc1	YPDnc2	YPDnc3	YPDnc4	EtOHnc1	EtOHnc2	Galnc1	Galnc2
13	SPT8	YLR055C	YPDcl2	YPDcl3	EtOHcl1	EtOHcl2	Galcl1	YPDnc1	YPDnc2	YPDnc3	YPDnc4	EtOHnc1	EtOHnc2	Galnc1	Galnc2
13		YPR074W-/	YPDcl2	YPDcl3	EtOHcl1	EtOHcl2	Galcl1	YPDnc1	YPDnc2	YPDnc3	YPDnc4	EtOHnc1	EtOHnc2	Galnc1	Galnc2
13	ATP17	YDR377W	YPDcl2	YPDcl3	EtOHcl1	EtOHcl2	Galcl1	YPDnc1	YPDnc2	YPDnc3	YPDnc4	EtOHnc1	EtOHnc2	Galnc1	Galnc2
13	VPS60	YDR486C	YPDcl2	YPDcl3	EtOHcl1	EtOHcl2	Galcl1	YPDnc1	YPDnc2	YPDnc3	YPDnc4	EtOHnc1	EtOHnc2	Galnc1	Galnc2
13	DMA1	YHR115C	YPDcl2	YPDcl3	EtOHcl1	EtOHcl2	Galcl1	YPDnc1	YPDnc2	YPDnc3	YPDnc4	EtOHnc1	EtOHnc2	Galnc1	Galnc2
13	TFC8	YPL007C	YPDcl2	YPDcl3	EtOHcl1	EtOHcl2	Galcl1	YPDnc1	YPDnc2	YPDnc3	YPDnc4	EtOHnc1	EtOHnc2	Galnc1	Galnc2
13		YDR248C	YPDcl2	YPDcl3	EtOHcl1	EtOHcl2	Galcl1	YPDnc1	YPDnc2	YPDnc3	YPDnc4	EtOHnc1	EtOHnc2	Galnc1	Galnc2
13		YHL037C	YPDcl2	YPDcl3	EtOHcl1	EtOHcl2	Galcl1	YPDnc1	YPDnc2	YPDnc3	YPDnc4	EtOHnc1	EtOHnc2	Galnc1	Galnc2

13	SRO7	YPR032W	YPDcl2	YPDcl3	EtOHcl1	EtOHcl2	Galcl1	YPDnc1	YPDnc2	YPDnc3	YPDnc4	EtOHnc1	EtOHnc2	Galnc1	Galnc2
13	RFA3	YJL173C	YPDcl2	YPDcl3	EtOHcl1	EtOHcl2	Galcl1	YPDnc1	YPDnc2	YPDnc3	YPDnc4	EtOHnc1	EtOHnc2	Galnc1	Galnc2
13		YOR114W	YPDcl2	YPDcl3	EtOHcl1	EtOHcl2	Galcl1	YPDnc1	YPDnc2	YPDnc3	YPDnc4	EtOHnc1	EtOHnc2	Galnc1	Galnc2
13	ESF2	YNR054C	YPDcl2	YPDcl3	EtOHcl1	EtOHcl2	Galcl1	YPDnc1	YPDnc2	YPDnc3	YPDnc4	EtOHnc1	EtOHnc2	Galnc1	Galnc2
13	IMG2	YCR071C	YPDcl2	YPDcl3	EtOHcl1	EtOHcl2	Galcl1	YPDnc1	YPDnc2	YPDnc3	YPDnc4	EtOHnc1	EtOHnc2	Galnc1	Galnc2
13	AIM46	YHR199C	YPDcl2	YPDcl3	EtOHcl1	EtOHcl2	Galcl1	YPDnc1	YPDnc2	YPDnc3	YPDnc4	EtOHnc1	EtOHnc2	Galnc1	Galnc2
13	INO4	YOL108C	YPDcl2	YPDcl3	EtOHcl1	EtOHcl2	Galcl1	YPDnc1	YPDnc2	YPDnc3	YPDnc4	EtOHnc1	EtOHnc2	Galnc1	Galnc2
13	RTT107	YHR154W	YPDcl2	YPDcl3	EtOHcl1	EtOHcl2	Galcl1	YPDnc1	YPDnc2	YPDnc3	YPDnc4	EtOHnc1	EtOHnc2	Galnc1	Galnc2
13	RRF1	YHR038W	YPDcl2	YPDcl3	EtOHcl1	EtOHcl2	Galcl1	YPDnc1	YPDnc2	YPDnc3	YPDnc4	EtOHnc1	EtOHnc2	Galnc1	Galnc2
13	PDE2	YOR360C	YPDcl2	YPDcl3	EtOHcl1	EtOHcl2	Galcl1	YPDnc1	YPDnc2	YPDnc3	YPDnc4	EtOHnc1	EtOHnc2	Galnc1	Galnc2
13	ASI3	YNL008C	YPDcl2	YPDcl3	EtOHcl1	EtOHcl2	Galcl1	YPDnc1	YPDnc2	YPDnc3	YPDnc4	EtOHnc1	EtOHnc2	Galnc1	Galnc2
13		YOR309C	YPDcl2	YPDcl3	EtOHcl1	EtOHcl2	Galcl1	YPDnc1	YPDnc2	YPDnc3	YPDnc4	EtOHnc1	EtOHnc2	Galnc1	Galnc2
13	YET1	YKL065C	YPDcl2	YPDcl3	EtOHcl1	EtOHcl2	Galcl1	YPDnc1	YPDnc2	YPDnc3	YPDnc4	EtOHnc1	EtOHnc2	Galnc1	Galnc2
13	SPP2	YOR148C	YPDcl2	YPDcl3	EtOHcl1	EtOHcl2	Galcl1	YPDnc1	YPDnc2	YPDnc3	YPDnc4	EtOHnc1	EtOHnc2	Galnc1	Galnc2
13	CDC6	YJL194W	YPDcl2	YPDcl3	EtOHcl1	EtOHcl2	Galcl1	YPDnc1	YPDnc2	YPDnc3	YPDnc4	EtOHnc1	EtOHnc2	Galnc1	Galnc2
13	RPN1	YHR027C	YPDcl2	YPDcl3	EtOHcl1	EtOHcl2	Galcl1	YPDnc1	YPDnc2	YPDnc3	YPDnc4	EtOHnc1	EtOHnc2	Galnc1	Galnc2
13	TRK2	YKR050W	YPDcl2	YPDcl3	EtOHcl1	EtOHcl2	Galcl1	YPDnc1	YPDnc2	YPDnc3	YPDnc4	EtOHnc1	EtOHnc2	Galnc1	Galnc2
13		YCR090C	YPDcl2	YPDcl3	EtOHcl1	EtOHcl2	Galcl1	YPDnc1	YPDnc2	YPDnc3	YPDnc4	EtOHnc1	EtOHnc2	Galnc1	Galnc2
13	CEP3	YMR168C	YPDcl2	YPDcl3	EtOHcl1	EtOHcl2	Galcl1	YPDnc1	YPDnc2	YPDnc3	YPDnc4	EtOHnc1	EtOHnc2	Galnc1	Galnc2
13	TVP23	YDR084C	YPDcl2	YPDcl3	EtOHcl1	EtOHcl2	Galcl1	YPDnc1	YPDnc2	YPDnc3	YPDnc4	EtOHnc1	EtOHnc2	Galnc1	Galnc2
13	RTG2	YGL252C	YPDcl2	YPDcl3	EtOHcl1	EtOHcl2	Galcl1	YPDnc1	YPDnc2	YPDnc3	YPDnc4	EtOHnc1	EtOHnc2	Galnc1	Galnc2
13	NIF3	YGL221C	YPDcl2	YPDcl3	EtOHcl1	EtOHcl2	Galcl1	YPDnc1	YPDnc2	YPDnc3	YPDnc4	EtOHnc1	EtOHnc2	Galnc1	Galnc2
13	APN1	YKL114C	YPDcl2	YPDcl3	EtOHcl1	EtOHcl2	Galcl1	YPDnc1	YPDnc2	YPDnc3	YPDnc4	EtOHnc1	EtOHnc2	Galnc1	Galnc2
13		YJR020W	YPDcl2	YPDcl3	EtOHcl1	EtOHcl2	Galcl1	YPDnc1	YPDnc2	YPDnc3	YPDnc4	EtOHnc1	EtOHnc2	Galnc1	Galnc2
13	FMP30	YPL103C	YPDcl2	YPDcl3	EtOHcl1	EtOHcl2	Galcl1	YPDnc1	YPDnc2	YPDnc3	YPDnc4	EtOHnc1	EtOHnc2	Galnc1	Galnc2
13	OSM1	YJR051W	YPDcl2	YPDcl3	EtOHcl1	EtOHcl2	Galcl1	YPDnc1	YPDnc2	YPDnc3	YPDnc4	EtOHnc1	EtOHnc2	Galnc1	Galnc2
13	BUL1	YMR275C	YPDcl2	YPDcl3	EtOHcl1	EtOHcl2	Galcl1	YPDnc1	YPDnc2	YPDnc3	YPDnc4	EtOHnc1	EtOHnc2	Galnc1	Galnc2
13	VPS25	YJR102C	YPDcl2	YPDcl3	EtOHcl1	EtOHcl2	Galcl1	YPDnc1	YPDnc2	YPDnc3	YPDnc4	EtOHnc1	EtOHnc2	Galnc1	Galnc2
13		YLR428C	YPDcl2	YPDcl3	EtOHcl1	EtOHcl2	Galcl1	YPDnc1	YPDnc2	YPDnc3	YPDnc4	EtOHnc1	EtOHnc2	Galnc1	Galnc2
13	RPL23B	YER117W	YPDcl2	YPDcl3	EtOHcl1	EtOHcl2	Galcl1	YPDnc1	YPDnc2	YPDnc3	YPDnc4	EtOHnc1	EtOHnc2	Galnc1	Galnc2
13	TBF1	YPL128C	YPDcl2	YPDcl3	EtOHcl1	EtOHcl2	Galcl1	YPDnc1	YPDnc2	YPDnc3	YPDnc4	EtOHnc1	EtOHnc2	Galnc1	Galnc2
13	HOC1	YJR075W	YPDcl2	YPDcl3	EtOHcl1	EtOHcl2	Galcl1	YPDnc1	YPDnc2	YPDnc3	YPDnc4	EtOHnc1	EtOHnc2	Galnc1	Galnc2
13	NAT2	YGR147C	YPDcl2	YPDcl3	EtOHcl1	EtOHcl2	Galcl1	YPDnc1	YPDnc2	YPDnc3	YPDnc4	EtOHnc1	EtOHnc2	Galnc1	Galnc2
13	UTP21	YLR409C	YPDcl2	YPDcl3	EtOHcl1	EtOHcl2	Galcl1	YPDnc1	YPDnc2	YPDnc3	YPDnc4	EtOHnc1	EtOHnc2	Galnc1	Galnc2

13	HEM15	YOR176W	YPDcl2	YPDcl3	EtOHcl1	EtOHcl2	Galcl1	YPDnc1	YPDnc2	YPDnc3	YPDnc4	EtOHnc1	EtOHnc2	Galnc1	Galnc2
13	DPB4	YDR121W	YPDcl2	YPDcl3	EtOHcl1	EtOHcl2	Galcl1	YPDnc1	YPDnc2	YPDnc3	YPDnc4	EtOHnc1	EtOHnc2	Galnc1	Galnc2
13		YNL190W	YPDcl2	YPDcl3	EtOHcl1	EtOHcl2	Galcl1	YPDnc1	YPDnc2	YPDnc3	YPDnc4	EtOHnc1	EtOHnc2	Galnc1	Galnc2
13	IRA2	YOL081W	YPDcl2	YPDcl3	EtOHcl1	EtOHcl2	Galcl1	YPDnc1	YPDnc2	YPDnc3	YPDnc4	EtOHnc1	EtOHnc2	Galnc1	Galnc2
13		YKL050C	YPDcl2	YPDcl3	EtOHcl1	EtOHcl2	Galcl1	YPDnc1	YPDnc2	YPDnc3	YPDnc4	EtOHnc1	EtOHnc2	Galnc1	Galnc2
13	RTN2	YDL204W	YPDcl2	YPDcl3	EtOHcl1	EtOHcl2	Galcl1	YPDnc1	YPDnc2	YPDnc3	YPDnc4	EtOHnc1	EtOHnc2	Galnc1	Galnc2
13	CSM3	YMR048W	YPDcl2	YPDcl3	EtOHcl1	EtOHcl2	Galcl1	YPDnc1	YPDnc2	YPDnc3	YPDnc4	EtOHnc1	EtOHnc2	Galnc1	Galnc2
13	PLB3	YOL011W	YPDcl2	YPDcl3	EtOHcl1	EtOHcl2	Galcl1	YPDnc1	YPDnc2	YPDnc3	YPDnc4	EtOHnc1	EtOHnc2	Galnc1	Galnc2
13	SMC5	YOL034W	YPDcl2	YPDcl3	EtOHcl1	EtOHcl2	Galcl1	YPDnc1	YPDnc2	YPDnc3	YPDnc4	EtOHnc1	EtOHnc2	Galnc1	Galnc2
13	MDL1	YLR188W	YPDcl2	YPDcl3	EtOHcl1	EtOHcl2	Galcl1	YPDnc1	YPDnc2	YPDnc3	YPDnc4	EtOHnc1	EtOHnc2	Galnc1	Galnc2
13	TOR1	YJR066W	YPDcl2	YPDcl3	EtOHcl1	EtOHcl2	Galcl1	YPDnc1	YPDnc2	YPDnc3	YPDnc4	EtOHnc1	EtOHnc2	Galnc1	Galnc2
13		YCR050C	YPDcl2	YPDcl3	EtOHcl1	EtOHcl2	Galcl1	YPDnc1	YPDnc2	YPDnc3	YPDnc4	EtOHnc1	EtOHnc2	Galnc1	Galnc2
13	MRP21	YBL090W	YPDcl2	YPDcl3	EtOHcl1	EtOHcl2	Galcl1	YPDnc1	YPDnc2	YPDnc3	YPDnc4	EtOHnc1	EtOHnc2	Galnc1	Galnc2
13	UME1	YPL139C	YPDcl2	YPDcl3	EtOHcl1	EtOHcl2	Galcl1	YPDnc1	YPDnc2	YPDnc3	YPDnc4	EtOHnc1	EtOHnc2	Galnc1	Galnc2
13	COS111	YBR203W	YPDcl2	YPDcl3	EtOHcl1	EtOHcl2	Galcl1	YPDnc1	YPDnc2	YPDnc3	YPDnc4	EtOHnc1	EtOHnc2	Galnc1	Galnc2
13	CLB6	YGR109C	YPDcl2	YPDcl3	EtOHcl1	EtOHcl2	Galcl1	YPDnc1	YPDnc2	YPDnc3	YPDnc4	EtOHnc1	EtOHnc2	Galnc1	Galnc2
13	VPS8	YAL002W	YPDcl2	YPDcl3	EtOHcl1	EtOHcl2	Galcl1	YPDnc1	YPDnc2	YPDnc3	YPDnc4	EtOHnc1	EtOHnc2	Galnc1	Galnc2
13		YKL165C-A	YPDcl2	YPDcl3	EtOHcl1	EtOHcl2	Galcl1	YPDnc1	YPDnc2	YPDnc3	YPDnc4	EtOHnc1	EtOHnc2	Galnc1	Galnc2
13	COQ6	YGR255C	YPDcl2	YPDcl3	EtOHcl1	EtOHcl2	Galcl1	YPDnc1	YPDnc2	YPDnc3	YPDnc4	EtOHnc1	EtOHnc2	Galnc1	Galnc2
13	RPL40B	YKR094C	YPDcl2	YPDcl3	EtOHcl1	EtOHcl2	Galcl1	YPDnc1	YPDnc2	YPDnc3	YPDnc4	EtOHnc1	EtOHnc2	Galnc1	Galnc2
13	TMT1	YER175C	YPDcl2	YPDcl3	EtOHcl1	EtOHcl2	Galcl1	YPDnc1	YPDnc2	YPDnc3	YPDnc4	EtOHnc1	EtOHnc2	Galnc1	Galnc2
13	AVT2	YEL064C	YPDcl2	YPDcl3	EtOHcl1	EtOHcl2	Galcl1	YPDnc1	YPDnc2	YPDnc3	YPDnc4	EtOHnc1	EtOHnc2	Galnc1	Galnc2
13	TPO3	YPR156C	YPDcl2	YPDcl3	EtOHcl1	EtOHcl2	Galcl1	YPDnc1	YPDnc2	YPDnc3	YPDnc4	EtOHnc1	EtOHnc2	Galnc1	Galnc2
13	YSP3	YOR003W	YPDcl2	YPDcl3	EtOHcl1	EtOHcl2	Galcl1	YPDnc1	YPDnc2	YPDnc3	YPDnc4	EtOHnc1	EtOHnc2	Galnc1	Galnc2
13	ARC35	YNR035C	YPDcl2	YPDcl3	EtOHcl1	EtOHcl2	Galcl1	YPDnc1	YPDnc2	YPDnc3	YPDnc4	EtOHnc1	EtOHnc2	Galnc1	Galnc2
13	MGE1	YOR232W	YPDcl2	YPDcl3	EtOHcl1	EtOHcl2	Galcl1	YPDnc1	YPDnc2	YPDnc3	YPDnc4	EtOHnc1	EtOHnc2	Galnc1	Galnc2
13	AIP1	YMR092C	YPDcl2	YPDcl3	EtOHcl1	EtOHcl2	Galcl1	YPDnc1	YPDnc2	YPDnc3	YPDnc4	EtOHnc1	EtOHnc2	Galnc1	Galnc2
13	MFA1	YDR461W	YPDcl2	YPDcl3	EtOHcl1	EtOHcl2	Galcl1	YPDnc1	YPDnc2	YPDnc3	YPDnc4	EtOHnc1	EtOHnc2	Galnc1	Galnc2
13	TIF4631	YGR162W	YPDcl2	YPDcl3	EtOHcl1	EtOHcl2	Galcl1	YPDnc1	YPDnc2	YPDnc3	YPDnc4	EtOHnc1	EtOHnc2	Galnc1	Galnc2
13	IDP1	YDL066W	YPDcl2	YPDcl3	EtOHcl1	EtOHcl2	Galcl1	YPDnc1	YPDnc2	YPDnc3	YPDnc4	EtOHnc1	EtOHnc2	Galnc1	Galnc2
13	RPB9	YGL070C	YPDcl2	YPDcl3	EtOHcl1	EtOHcl2	Galcl1	YPDnc1	YPDnc2	YPDnc3	YPDnc4	EtOHnc1	EtOHnc2	Galnc1	Galnc2
13	HSM3	YBR272C	YPDcl2	YPDcl3	EtOHcl1	EtOHcl2	Galcl1	YPDnc1	YPDnc2	YPDnc3	YPDnc4	EtOHnc1	EtOHnc2	Galnc1	Galnc2
13	RRP42	YDL111C	YPDcl2	YPDcl3	EtOHcl1	EtOHcl2	Galcl1	YPDnc1	YPDnc2	YPDnc3	YPDnc4	EtOHnc1	EtOHnc2	Galnc1	Galnc2
13	RLF2	YPR018W	YPDcl2	YPDcl3	EtOHcl1	EtOHcl2	Galcl1	YPDnc1	YPDnc2	YPDnc3	YPDnc4	EtOHnc1	EtOHnc2	Galnc1	Galnc2

13	PHA2	YNL316C	YPDcl2	YPDcl3	EtOHcl1	EtOHcl2	Galcl1	YPDnc1	YPDnc2	YPDnc3	YPDnc4	EtOHnc1	EtOHnc2	Galnc1	Galnc2
13	RTS3	YGR161C	YPDcl2	YPDcl3	EtOHcl1	EtOHcl2	Galcl1	YPDnc1	YPDnc2	YPDnc3	YPDnc4	EtOHnc1	EtOHnc2	Galnc1	Galnc2
13	KKQ8	YKL168C	YPDcl2	YPDcl3	EtOHcl1	EtOHcl2	Galcl1	YPDnc1	YPDnc2	YPDnc3	YPDnc4	EtOHnc1	EtOHnc2	Galnc1	Galnc2
13	AVO2	YMR068W	YPDcl2	YPDcl3	EtOHcl1	EtOHcl2	Galcl1	YPDnc1	YPDnc2	YPDnc3	YPDnc4	EtOHnc1	EtOHnc2	Galnc1	Galnc2
13		YDR476C	YPDcl2	YPDcl3	EtOHcl1	EtOHcl2	Galcl1	YPDnc1	YPDnc2	YPDnc3	YPDnc4	EtOHnc1	EtOHnc2	Galnc1	Galnc2
13		YGL165C	YPDcl2	YPDcl3	EtOHcl1	EtOHcl2	Galcl1	YPDnc1	YPDnc2	YPDnc3	YPDnc4	EtOHnc1	EtOHnc2	Galnc1	Galnc2
13		YPL272C	YPDcl2	YPDcl3	EtOHcl1	EtOHcl2	Galcl1	YPDnc1	YPDnc2	YPDnc3	YPDnc4	EtOHnc1	EtOHnc2	Galnc1	Galnc2
13		YHR080C	YPDcl2	YPDcl3	EtOHcl1	EtOHcl2	Galcl1	YPDnc1	YPDnc2	YPDnc3	YPDnc4	EtOHnc1	EtOHnc2	Galnc1	Galnc2
13	ATG20	YDL113C	YPDcl2	YPDcl3	EtOHcl1	EtOHcl2	Galcl1	YPDnc1	YPDnc2	YPDnc3	YPDnc4	EtOHnc1	EtOHnc2	Galnc1	Galnc2
13		YBL086C	YPDcl2	YPDcl3	EtOHcl1	EtOHcl2	Galcl1	YPDnc1	YPDnc2	YPDnc3	YPDnc4	EtOHnc1	EtOHnc2	Galnc1	Galnc2
13	PMC1	YGL006W	YPDcl2	YPDcl3	EtOHcl1	EtOHcl2	Galcl1	YPDnc1	YPDnc2	YPDnc3	YPDnc4	EtOHnc1	EtOHnc2	Galnc1	Galnc2
13		YMR007W	YPDcl2	YPDcl3	EtOHcl1	EtOHcl2	Galcl1	YPDnc1	YPDnc2	YPDnc3	YPDnc4	EtOHnc1	EtOHnc2	Galnc1	Galnc2
13	ULI1	YFR026C	YPDcl2	YPDcl3	EtOHcl1	EtOHcl2	Galcl1	YPDnc1	YPDnc2	YPDnc3	YPDnc4	EtOHnc1	EtOHnc2	Galnc1	Galnc2
13	SET1	YHR119W	YPDcl2	YPDcl3	EtOHcl1	EtOHcl2	Galcl1	YPDnc1	YPDnc2	YPDnc3	YPDnc4	EtOHnc1	EtOHnc2	Galnc1	Galnc2
13	AIM33	YML087C	YPDcl2	YPDcl3	EtOHcl1	EtOHcl2	Galcl1	YPDnc1	YPDnc2	YPDnc3	YPDnc4	EtOHnc1	EtOHnc2	Galnc1	Galnc2
13	MRPL44	YMR225C	YPDcl2	YPDcl3	EtOHcl1	EtOHcl2	Galcl1	YPDnc1	YPDnc2	YPDnc3	YPDnc4	EtOHnc1	EtOHnc2	Galnc1	Galnc2
13	TPT1	YOL102C	YPDcl2	YPDcl3	EtOHcl1	EtOHcl2	Galcl1	YPDnc1	YPDnc2	YPDnc3	YPDnc4	EtOHnc1	EtOHnc2	Galnc1	Galnc2
13	SSE2	YBR169C	YPDcl2	YPDcl3	EtOHcl1	EtOHcl2	Galcl1	YPDnc1	YPDnc2	YPDnc3	YPDnc4	EtOHnc1	EtOHnc2	Galnc1	Galnc2
13	FAB1	YFR019W	YPDcl2	YPDcl3	EtOHcl1	EtOHcl2	Galcl1	YPDnc1	YPDnc2	YPDnc3	YPDnc4	EtOHnc1	EtOHnc2	Galnc1	Galnc2
13		YBR126W-I	YPDcl2	YPDcl3	EtOHcl1	EtOHcl2	Galcl1	YPDnc1	YPDnc2	YPDnc3	YPDnc4	EtOHnc1	EtOHnc2	Galnc1	Galnc2
13	PGC1	YPL206C	YPDcl2	YPDcl3	EtOHcl1	EtOHcl2	Galcl1	YPDnc1	YPDnc2	YPDnc3	YPDnc4	EtOHnc1	EtOHnc2	Galnc1	Galnc2
13	ASG7	YJL170C	YPDcl2	YPDcl3	EtOHcl1	EtOHcl2	Galcl1	YPDnc1	YPDnc2	YPDnc3	YPDnc4	EtOHnc1	EtOHnc2	Galnc1	Galnc2
13		YKR073C	YPDcl2	YPDcl3	EtOHcl1	EtOHcl2	Galcl1	YPDnc1	YPDnc2	YPDnc3	YPDnc4	EtOHnc1	EtOHnc2	Galnc1	Galnc2
13	KRE1	YNL322C	YPDcl2	YPDcl3	EtOHcl1	EtOHcl2	Galcl1	YPDnc1	YPDnc2	YPDnc3	YPDnc4	EtOHnc1	EtOHnc2	Galnc1	Galnc2
13		YBL100W-(	YPDcl2	YPDcl3	EtOHcl1	EtOHcl2	Galcl1	YPDnc1	YPDnc2	YPDnc3	YPDnc4	EtOHnc1	EtOHnc2	Galnc1	Galnc2
13	YSP1	YHR155W	YPDcl2	YPDcl3	EtOHcl1	EtOHcl2	Galcl1	YPDnc1	YPDnc2	YPDnc3	YPDnc4	EtOHnc1	EtOHnc2	Galnc1	Galnc2
13	MVD1	YNR043W	YPDcl2	YPDcl3	EtOHcl1	EtOHcl2	Galcl1	YPDnc1	YPDnc2	YPDnc3	YPDnc4	EtOHnc1	EtOHnc2	Galnc1	Galnc2
13		YBR223W-/	YPDcl2	YPDcl3	EtOHcl1	EtOHcl2	Galcl1	YPDnc1	YPDnc2	YPDnc3	YPDnc4	EtOHnc1	EtOHnc2	Galnc1	Galnc2
13	EMP70	YLR083C	YPDcl2	YPDcl3	EtOHcl1	EtOHcl2	Galcl1	YPDnc1	YPDnc2	YPDnc3	YPDnc4	EtOHnc1	EtOHnc2	Galnc1	Galnc2
13	TOM20	YGR082W	YPDcl2	YPDcl3	EtOHcl1	EtOHcl2	Galcl1	YPDnc1	YPDnc2	YPDnc3	YPDnc4	EtOHnc1	EtOHnc2	Galnc1	Galnc2
13	SPO16	YHR153C	YPDcl2	YPDcl3	EtOHcl1	EtOHcl2	Galcl1	YPDnc1	YPDnc2	YPDnc3	YPDnc4	EtOHnc1	EtOHnc2	Galnc1	Galnc2
13	RHO4	YKR055W	YPDcl2	YPDcl3	EtOHcl1	EtOHcl2	Galcl1	YPDnc1	YPDnc2	YPDnc3	YPDnc4	EtOHnc1	EtOHnc2	Galnc1	Galnc2
13	SKN7	YHR206W	YPDcl2	YPDcl3	EtOHcl1	EtOHcl2	Galcl1	YPDnc1	YPDnc2	YPDnc3	YPDnc4	EtOHnc1	EtOHnc2	Galnc1	Galnc2
13	RPS6A	YPL090C	YPDcl2	YPDcl3	EtOHcl1	EtOHcl2	Galcl1	YPDnc1	YPDnc2	YPDnc3	YPDnc4	EtOHnc1	EtOHnc2	Galnc1	Galnc2

13		YLR132C	YPDcl2	YPDcl3	EtOHcl1	EtOHcl2	Galcl1	YPDnc1	YPDnc2	YPDnc3	YPDnc4	EtOHnc1	EtOHnc2	Galnc1	Galnc2
13	SSN8	YNL025C	YPDcl2	YPDcl3	EtOHcl1	EtOHcl2	Galcl1	YPDnc1	YPDnc2	YPDnc3	YPDnc4	EtOHnc1	EtOHnc2	Galnc1	Galnc2
13	PRR2	YDL214C	YPDcl2	YPDcl3	EtOHcl1	EtOHcl2	Galcl1	YPDnc1	YPDnc2	YPDnc3	YPDnc4	EtOHnc1	EtOHnc2	Galnc1	Galnc2
13	ALG3	YBL082C	YPDcl2	YPDcl3	EtOHcl1	EtOHcl2	Galcl1	YPDnc1	YPDnc2	YPDnc3	YPDnc4	EtOHnc1	EtOHnc2	Galnc1	Galnc2
13	NUP145	YGL092W	YPDcl2	YPDcl3	EtOHcl1	EtOHcl2	Galcl1	YPDnc1	YPDnc2	YPDnc3	YPDnc4	EtOHnc1	EtOHnc2	Galnc1	Galnc2
13		YGL159W	YPDcl2	YPDcl3	EtOHcl1	EtOHcl2	Galcl1	YPDnc1	YPDnc2	YPDnc3	YPDnc4	EtOHnc1	EtOHnc2	Galnc1	Galnc2
13	RBA50	YDR527W	YPDcl2	YPDcl3	EtOHcl1	EtOHcl2	Galcl1	YPDnc1	YPDnc2	YPDnc3	YPDnc4	EtOHnc1	EtOHnc2	Galnc1	Galnc2
13	PHO5	YBR093C	YPDcl2	YPDcl3	EtOHcl1	EtOHcl2	Galcl1	YPDnc1	YPDnc2	YPDnc3	YPDnc4	EtOHnc1	EtOHnc2	Galnc1	Galnc2
13		YGL138C	YPDcl2	YPDcl3	EtOHcl1	EtOHcl2	Galcl1	YPDnc1	YPDnc2	YPDnc3	YPDnc4	EtOHnc1	EtOHnc2	Galnc1	Galnc2
13		YDR391C	YPDcl2	YPDcl3	EtOHcl1	EtOHcl2	Galcl1	YPDnc1	YPDnc2	YPDnc3	YPDnc4	EtOHnc1	EtOHnc2	Galnc1	Galnc2
13		YOR131C	YPDcl2	YPDcl3	EtOHcl1	EtOHcl2	Galcl1	YPDnc1	YPDnc2	YPDnc3	YPDnc4	EtOHnc1	EtOHnc2	Galnc1	Galnc2
13	HXT3	YDR345C	YPDcl2	YPDcl3	EtOHcl1	EtOHcl2	Galcl1	YPDnc1	YPDnc2	YPDnc3	YPDnc4	EtOHnc1	EtOHnc2	Galnc1	Galnc2
13	MNN2	YBR015C	YPDcl2	YPDcl3	EtOHcl1	EtOHcl2	Galcl1	YPDnc1	YPDnc2	YPDnc3	YPDnc4	EtOHnc1	EtOHnc2	Galnc1	Galnc2
13	ESL1	YIL151C	YPDcl2	YPDcl3	EtOHcl1	EtOHcl2	Galcl1	YPDnc1	YPDnc2	YPDnc3	YPDnc4	EtOHnc1	EtOHnc2	Galnc1	Galnc2
13	PML1	YLR016C	YPDcl2	YPDcl3	EtOHcl1	EtOHcl2	Galcl1	YPDnc1	YPDnc2	YPDnc3	YPDnc4	EtOHnc1	EtOHnc2	Galnc1	Galnc2
13	APC2	YLR127C	YPDcl2	YPDcl3	EtOHcl1	EtOHcl2	Galcl1	YPDnc1	YPDnc2	YPDnc3	YPDnc4	EtOHnc1	EtOHnc2	Galnc1	Galnc2
13	UBP10	YNL186W	YPDcl2	YPDcl3	EtOHcl1	EtOHcl2	Galcl1	YPDnc1	YPDnc2	YPDnc3	YPDnc4	EtOHnc1	EtOHnc2	Galnc1	Galnc2
13	LSB3	YFR024C-A	YPDcl2	YPDcl3	EtOHcl1	EtOHcl2	Galcl1	YPDnc1	YPDnc2	YPDnc3	YPDnc4	EtOHnc1	EtOHnc2	Galnc1	Galnc2
13	RPC40	YPR110C	YPDcl2	YPDcl3	EtOHcl1	EtOHcl2	Galcl1	YPDnc1	YPDnc2	YPDnc3	YPDnc4	EtOHnc1	EtOHnc2	Galnc1	Galnc2
13		YNL324W	YPDcl2	YPDcl3	EtOHcl1	EtOHcl2	Galcl1	YPDnc1	YPDnc2	YPDnc3	YPDnc4	EtOHnc1	EtOHnc2	Galnc1	Galnc2
13	FEN1	YCR034W	YPDcl2	YPDcl3	EtOHcl1	EtOHcl2	Galcl1	YPDnc1	YPDnc2	YPDnc3	YPDnc4	EtOHnc1	EtOHnc2	Galnc1	Galnc2
13	CKA1	YIL035C	YPDcl2	YPDcl3	EtOHcl1	EtOHcl2	Galcl1	YPDnc1	YPDnc2	YPDnc3	YPDnc4	EtOHnc1	EtOHnc2	Galnc1	Galnc2
13	HEK2	YBL032W	YPDcl2	YPDcl3	EtOHcl1	EtOHcl2	Galcl1	YPDnc1	YPDnc2	YPDnc3	YPDnc4	EtOHnc1	EtOHnc2	Galnc1	Galnc2
13	RSE1	YML049C	YPDcl2	YPDcl3	EtOHcl1	EtOHcl2	Galcl1	YPDnc1	YPDnc2	YPDnc3	YPDnc4	EtOHnc1	EtOHnc2	Galnc1	Galnc2
13		YKL083W	YPDcl2	YPDcl3	EtOHcl1	EtOHcl2	Galcl1	YPDnc1	YPDnc2	YPDnc3	YPDnc4	EtOHnc1	EtOHnc2	Galnc1	Galnc2
13	PIM1	YBL022C	YPDcl2	YPDcl3	EtOHcl1	EtOHcl2	Galcl1	YPDnc1	YPDnc2	YPDnc3	YPDnc4	EtOHnc1	EtOHnc2	Galnc1	Galnc2
13	CUS1	YMR240C	YPDcl2	YPDcl3	EtOHcl1	EtOHcl2	Galcl1	YPDnc1	YPDnc2	YPDnc3	YPDnc4	EtOHnc1	EtOHnc2	Galnc1	Galnc2
13		YIL089W	YPDcl2	YPDcl3	EtOHcl1	EtOHcl2	Galcl1	YPDnc1	YPDnc2	YPDnc3	YPDnc4	EtOHnc1	EtOHnc2	Galnc1	Galnc2
13	AFT1	YGL071W	YPDcl2	YPDcl3	EtOHcl1	EtOHcl2	Galcl1	YPDnc1	YPDnc2	YPDnc3	YPDnc4	EtOHnc1	EtOHnc2	Galnc1	Galnc2
13	BEM3	YPL115C	YPDcl2	YPDcl3	EtOHcl1	EtOHcl2	Galcl1	YPDnc1	YPDnc2	YPDnc3	YPDnc4	EtOHnc1	EtOHnc2	Galnc1	Galnc2
13	ERB1	YMR049C	YPDcl2	YPDcl3	EtOHcl1	EtOHcl2	Galcl1	YPDnc1	YPDnc2	YPDnc3	YPDnc4	EtOHnc1	EtOHnc2	Galnc1	Galnc2
13	MET30	YIL046W	YPDcl2	YPDcl3	EtOHcl1	EtOHcl2	Galcl1	YPDnc1	YPDnc2	YPDnc3	YPDnc4	EtOHnc1	EtOHnc2	Galnc1	Galnc2
13	GOT1	YMR292W	YPDcl2	YPDcl3	EtOHcl1	EtOHcl2	Galcl1	YPDnc1	YPDnc2	YPDnc3	YPDnc4	EtOHnc1	EtOHnc2	Galnc1	Galnc2
13	PRP28	YDR243C	YPDcl2	YPDcl3	EtOHcl1	EtOHcl2	Galcl1	YPDnc1	YPDnc2	YPDnc3	YPDnc4	EtOHnc1	EtOHnc2	Galnc1	Galnc2

13	APC11	YDL008W	YPDcl2	YPDcl3	EtOHcl1	EtOHcl2	Galcl1	YPDnc1	YPDnc2	YPDnc3	YPDnc4	EtOHnc1	EtOHnc2	Galnc1	Galnc2
13	TIM23	YNR017W	YPDcl2	YPDcl3	EtOHcl1	EtOHcl2	Galcl1	YPDnc1	YPDnc2	YPDnc3	YPDnc4	EtOHnc1	EtOHnc2	Galnc1	Galnc2
13	FIT3	YOR383C	YPDcl2	YPDcl3	EtOHcl1	EtOHcl2	Galcl1	YPDnc1	YPDnc2	YPDnc3	YPDnc4	EtOHnc1	EtOHnc2	Galnc1	Galnc2
13		YOR246C	YPDcl2	YPDcl3	EtOHcl1	EtOHcl2	Galcl1	YPDnc1	YPDnc2	YPDnc3	YPDnc4	EtOHnc1	EtOHnc2	Galnc1	Galnc2
13	IOC2	YLR095C	YPDcl2	YPDcl3	EtOHcl1	EtOHcl2	Galcl1	YPDnc1	YPDnc2	YPDnc3	YPDnc4	EtOHnc1	EtOHnc2	Galnc1	Galnc2
13		YNL050C	YPDcl2	YPDcl3	EtOHcl1	EtOHcl2	Galcl1	YPDnc1	YPDnc2	YPDnc3	YPDnc4	EtOHnc1	EtOHnc2	Galnc1	Galnc2
13	TRS33	YOR115C	YPDcl2	YPDcl3	EtOHcl1	EtOHcl2	Galcl1	YPDnc1	YPDnc2	YPDnc3	YPDnc4	EtOHnc1	EtOHnc2	Galnc1	Galnc2
13	RPS7B	YNL096C	YPDcl2	YPDcl3	EtOHcl1	EtOHcl2	Galcl1	YPDnc1	YPDnc2	YPDnc3	YPDnc4	EtOHnc1	EtOHnc2	Galnc1	Galnc2
13	RIX7	YLL034C	YPDcl2	YPDcl3	EtOHcl1	EtOHcl2	Galcl1	YPDnc1	YPDnc2	YPDnc3	YPDnc4	EtOHnc1	EtOHnc2	Galnc1	Galnc2
13	DED81	YHR019C	YPDcl2	YPDcl3	EtOHcl1	EtOHcl2	Galcl1	YPDnc1	YPDnc2	YPDnc3	YPDnc4	EtOHnc1	EtOHnc2	Galnc1	Galnc2
13		YKL033W-A	YPDcl2	YPDcl3	EtOHcl1	EtOHcl2	Galcl1	YPDnc1	YPDnc2	YPDnc3	YPDnc4	EtOHnc1	EtOHnc2	Galnc1	Galnc2
13	NMA111	YNL123W	YPDcl2	YPDcl3	EtOHcl1	EtOHcl2	Galcl1	YPDnc1	YPDnc2	YPDnc3	YPDnc4	EtOHnc1	EtOHnc2	Galnc1	Galnc2
13	BRO1	YPL084W	YPDcl2	YPDcl3	EtOHcl1	EtOHcl2	Galcl1	YPDnc1	YPDnc2	YPDnc3	YPDnc4	EtOHnc1	EtOHnc2	Galnc1	Galnc2
13		YNL162W-A	YPDcl2	YPDcl3	EtOHcl1	EtOHcl2	Galcl1	YPDnc1	YPDnc2	YPDnc3	YPDnc4	EtOHnc1	EtOHnc2	Galnc1	Galnc2
13	TNA1	YGR260W	YPDcl2	YPDcl3	EtOHcl1	EtOHcl2	Galcl1	YPDnc1	YPDnc2	YPDnc3	YPDnc4	EtOHnc1	EtOHnc2	Galnc1	Galnc2
13		YMR122W-	YPDcl2	YPDcl3	EtOHcl1	EtOHcl2	Galcl1	YPDnc1	YPDnc2	YPDnc3	YPDnc4	EtOHnc1	EtOHnc2	Galnc1	Galnc2
13	RPL2A	YFR031C-A	YPDcl2	YPDcl3	EtOHcl1	EtOHcl2	Galcl1	YPDnc1	YPDnc2	YPDnc3	YPDnc4	EtOHnc1	EtOHnc2	Galnc1	Galnc2
13	RPL4B	YDR012W	YPDcl2	YPDcl3	EtOHcl1	EtOHcl2	Galcl1	YPDnc1	YPDnc2	YPDnc3	YPDnc4	EtOHnc1	EtOHnc2	Galnc1	Galnc2
13	AZF1	YOR113W	YPDcl2	YPDcl3	EtOHcl1	EtOHcl2	Galcl1	YPDnc1	YPDnc2	YPDnc3	YPDnc4	EtOHnc1	EtOHnc2	Galnc1	Galnc2
13	AIM21	YIR003W	YPDcl2	YPDcl3	EtOHcl1	EtOHcl2	Galcl1	YPDnc1	YPDnc2	YPDnc3	YPDnc4	EtOHnc1	EtOHnc2	Galnc1	Galnc2
13	DAD3	YBR233W-A	YPDcl2	YPDcl3	EtOHcl1	EtOHcl2	Galcl1	YPDnc1	YPDnc2	YPDnc3	YPDnc4	EtOHnc1	EtOHnc2	Galnc1	Galnc2
13	ECM19	YLR390W	YPDcl2	YPDcl3	EtOHcl1	EtOHcl2	Galcl1	YPDnc1	YPDnc2	YPDnc3	YPDnc4	EtOHnc1	EtOHnc2	Galnc1	Galnc2
13	CDC16	YKL022C	YPDcl2	YPDcl3	EtOHcl1	EtOHcl2	Galcl1	YPDnc1	YPDnc2	YPDnc3	YPDnc4	EtOHnc1	EtOHnc2	Galnc1	Galnc2
13	REC104	YHR157W	YPDcl2	YPDcl3	EtOHcl1	EtOHcl2	Galcl1	YPDnc1	YPDnc2	YPDnc3	YPDnc4	EtOHnc1	EtOHnc2	Galnc1	Galnc2
13	SYN8	YAL014C	YPDcl2	YPDcl3	EtOHcl1	EtOHcl2	Galcl1	YPDnc1	YPDnc2	YPDnc3	YPDnc4	EtOHnc1	EtOHnc2	Galnc1	Galnc2
13	THP1	YOL072W	YPDcl2	YPDcl3	EtOHcl1	EtOHcl2	Galcl1	YPDnc1	YPDnc2	YPDnc3	YPDnc4	EtOHnc1	EtOHnc2	Galnc1	Galnc2
13	HIT1	YJR055W	YPDcl2	YPDcl3	EtOHcl1	EtOHcl2	Galcl1	YPDnc1	YPDnc2	YPDnc3	YPDnc4	EtOHnc1	EtOHnc2	Galnc1	Galnc2
13	FZO1	YBR179C	YPDcl2	YPDcl3	EtOHcl1	EtOHcl2	Galcl1	YPDnc1	YPDnc2	YPDnc3	YPDnc4	EtOHnc1	EtOHnc2	Galnc1	Galnc2
13	BUR2	YLR226W	YPDcl2	YPDcl3	EtOHcl1	EtOHcl2	Galcl1	YPDnc1	YPDnc2	YPDnc3	YPDnc4	EtOHnc1	EtOHnc2	Galnc1	Galnc2
13		YBR230W-A	YPDcl2	YPDcl3	EtOHcl1	EtOHcl2	Galcl1	YPDnc1	YPDnc2	YPDnc3	YPDnc4	EtOHnc1	EtOHnc2	Galnc1	Galnc2
13	LDB19	YOR322C	YPDcl2	YPDcl3	EtOHcl1	EtOHcl2	Galcl1	YPDnc1	YPDnc2	YPDnc3	YPDnc4	EtOHnc1	EtOHnc2	Galnc1	Galnc2
13	COP1	YDL145C	YPDcl2	YPDcl3	EtOHcl1	EtOHcl2	Galcl1	YPDnc1	YPDnc2	YPDnc3	YPDnc4	EtOHnc1	EtOHnc2	Galnc1	Galnc2
13	EXO1	YOR033C	YPDcl2	YPDcl3	EtOHcl1	EtOHcl2	Galcl1	YPDnc1	YPDnc2	YPDnc3	YPDnc4	EtOHnc1	EtOHnc2	Galnc1	Galnc2
13	MSA2	YKR077W	YPDcl2	YPDcl3	EtOHcl1	EtOHcl2	Galcl1	YPDnc1	YPDnc2	YPDnc3	YPDnc4	EtOHnc1	EtOHnc2	Galnc1	Galnc2

13	SRP54	YPR088C	YPDcl2	YPDcl3	EtOHcl1	EtOHcl2	Galcl1	YPDnc1	YPDnc2	YPDnc3	YPDnc4	EtOHnc1	EtOHnc2	Galnc1	Galnc2
13		YPR063C	YPDcl2	YPDcl3	EtOHcl1	EtOHcl2	Galcl1	YPDnc1	YPDnc2	YPDnc3	YPDnc4	EtOHnc1	EtOHnc2	Galnc1	Galnc2
13	DCW1	YKL046C	YPDcl2	YPDcl3	EtOHcl1	EtOHcl2	Galcl1	YPDnc1	YPDnc2	YPDnc3	YPDnc4	EtOHnc1	EtOHnc2	Galnc1	Galnc2
13	GLE2	YER107C	YPDcl2	YPDcl3	EtOHcl1	EtOHcl2	Galcl1	YPDnc1	YPDnc2	YPDnc3	YPDnc4	EtOHnc1	EtOHnc2	Galnc1	Galnc2
13	SMK1	YPR054W	YPDcl2	YPDcl3	EtOHcl1	EtOHcl2	Galcl1	YPDnc1	YPDnc2	YPDnc3	YPDnc4	EtOHnc1	EtOHnc2	Galnc1	Galnc2
13	AIM5	YBR262C	YPDcl2	YPDcl3	EtOHcl1	EtOHcl2	Galcl1	YPDnc1	YPDnc2	YPDnc3	YPDnc4	EtOHnc1	EtOHnc2	Galnc1	Galnc2
13	SLU7	YDR088C	YPDcl2	YPDcl3	EtOHcl1	EtOHcl2	Galcl1	YPDnc1	YPDnc2	YPDnc3	YPDnc4	EtOHnc1	EtOHnc2	Galnc1	Galnc2
13	ECM10	YEL030W	YPDcl2	YPDcl3	EtOHcl1	EtOHcl2	Galcl1	YPDnc1	YPDnc2	YPDnc3	YPDnc4	EtOHnc1	EtOHnc2	Galnc1	Galnc2
13	LYS1	YIR034C	YPDcl2	YPDcl3	EtOHcl1	EtOHcl2	Galcl1	YPDnc1	YPDnc2	YPDnc3	YPDnc4	EtOHnc1	EtOHnc2	Galnc1	Galnc2
13	LAC1	YKL008C	YPDcl2	YPDcl3	EtOHcl1	EtOHcl2	Galcl1	YPDnc1	YPDnc2	YPDnc3	YPDnc4	EtOHnc1	EtOHnc2	Galnc1	Galnc2
13	TDA1	YMR291W	YPDcl2	YPDcl3	EtOHcl1	EtOHcl2	Galcl1	YPDnc1	YPDnc2	YPDnc3	YPDnc4	EtOHnc1	EtOHnc2	Galnc1	Galnc2
13	RAD33	YML011C	YPDcl2	YPDcl3	EtOHcl1	EtOHcl2	Galcl1	YPDnc1	YPDnc2	YPDnc3	YPDnc4	EtOHnc1	EtOHnc2	Galnc1	Galnc2
13	CAM1	YPL048W	YPDcl2	YPDcl3	EtOHcl1	EtOHcl2	Galcl1	YPDnc1	YPDnc2	YPDnc3	YPDnc4	EtOHnc1	EtOHnc2	Galnc1	Galnc2
13	BNR1	YIL159W	YPDcl2	YPDcl3	EtOHcl1	EtOHcl2	Galcl1	YPDnc1	YPDnc2	YPDnc3	YPDnc4	EtOHnc1	EtOHnc2	Galnc1	Galnc2
13	PRP43	YGL120C	YPDcl2	YPDcl3	EtOHcl1	EtOHcl2	Galcl1	YPDnc1	YPDnc2	YPDnc3	YPDnc4	EtOHnc1	EtOHnc2	Galnc1	Galnc2
13	SEN34	YAR008W	YPDcl2	YPDcl3	EtOHcl1	EtOHcl2	Galcl1	YPDnc1	YPDnc2	YPDnc3	YPDnc4	EtOHnc1	EtOHnc2	Galnc1	Galnc2
13	CDC20	YGL116W	YPDcl2	YPDcl3	EtOHcl1	EtOHcl2	Galcl1	YPDnc1	YPDnc2	YPDnc3	YPDnc4	EtOHnc1	EtOHnc2	Galnc1	Galnc2
13	VID30	YGL227W	YPDcl2	YPDcl3	EtOHcl1	EtOHcl2	Galcl1	YPDnc1	YPDnc2	YPDnc3	YPDnc4	EtOHnc1	EtOHnc2	Galnc1	Galnc2
13		YDR506C	YPDcl2	YPDcl3	EtOHcl1	EtOHcl2	Galcl1	YPDnc1	YPDnc2	YPDnc3	YPDnc4	EtOHnc1	EtOHnc2	Galnc1	Galnc2
13		YER135C	YPDcl2	YPDcl3	EtOHcl1	EtOHcl2	Galcl1	YPDnc1	YPDnc2	YPDnc3	YPDnc4	EtOHnc1	EtOHnc2	Galnc1	Galnc2
13	BFR2	YDR299W	YPDcl2	YPDcl3	EtOHcl1	EtOHcl2	Galcl1	YPDnc1	YPDnc2	YPDnc3	YPDnc4	EtOHnc1	EtOHnc2	Galnc1	Galnc2
13		YOR223W	YPDcl2	YPDcl3	EtOHcl1	EtOHcl2	Galcl1	YPDnc1	YPDnc2	YPDnc3	YPDnc4	EtOHnc1	EtOHnc2	Galnc1	Galnc2
13		YLR001C	YPDcl2	YPDcl3	EtOHcl1	EtOHcl2	Galcl1	YPDnc1	YPDnc2	YPDnc3	YPDnc4	EtOHnc1	EtOHnc2	Galnc1	Galnc2
13	EFB1	YAL003W	YPDcl2	YPDcl3	EtOHcl1	EtOHcl2	Galcl1	YPDnc1	YPDnc2	YPDnc3	YPDnc4	EtOHnc1	EtOHnc2	Galnc1	Galnc2
13	PFS1	YHR185C	YPDcl2	YPDcl3	EtOHcl1	EtOHcl2	Galcl1	YPDnc1	YPDnc2	YPDnc3	YPDnc4	EtOHnc1	EtOHnc2	Galnc1	Galnc2
13	DIG1	YPL049C	YPDcl2	YPDcl3	EtOHcl1	EtOHcl2	Galcl1	YPDnc1	YPDnc2	YPDnc3	YPDnc4	EtOHnc1	EtOHnc2	Galnc1	Galnc2
13	RCN1	YKL159C	YPDcl2	YPDcl3	EtOHcl1	EtOHcl2	Galcl1	YPDnc1	YPDnc2	YPDnc3	YPDnc4	EtOHnc1	EtOHnc2	Galnc1	Galnc2
13	PEX2	YJL210W	YPDcl2	YPDcl3	EtOHcl1	EtOHcl2	Galcl1	YPDnc1	YPDnc2	YPDnc3	YPDnc4	EtOHnc1	EtOHnc2	Galnc1	Galnc2
13	ACH1	YBL015W	YPDcl2	YPDcl3	EtOHcl1	EtOHcl2	Galcl1	YPDnc1	YPDnc2	YPDnc3	YPDnc4	EtOHnc1	EtOHnc2	Galnc1	Galnc2
13	ACS1	YAL054C	YPDcl2	YPDcl3	EtOHcl1	EtOHcl2	Galcl1	YPDnc1	YPDnc2	YPDnc3	YPDnc4	EtOHnc1	EtOHnc2	Galnc1	Galnc2
13	RPN4	YDL020C	YPDcl2	YPDcl3	EtOHcl1	EtOHcl2	Galcl1	YPDnc1	YPDnc2	YPDnc3	YPDnc4	EtOHnc1	EtOHnc2	Galnc1	Galnc2
13	PRP6	YBR055C	YPDcl2	YPDcl3	EtOHcl1	EtOHcl2	Galcl1	YPDnc1	YPDnc2	YPDnc3	YPDnc4	EtOHnc1	EtOHnc2	Galnc1	Galnc2
13	RPL2B	YIL018W	YPDcl2	YPDcl3	EtOHcl1	EtOHcl2	Galcl1	YPDnc1	YPDnc2	YPDnc3	YPDnc4	EtOHnc1	EtOHnc2	Galnc1	Galnc2
13	SET4	YJL105W	YPDcl2	YPDcl3	EtOHcl1	EtOHcl2	Galcl1	YPDnc1	YPDnc2	YPDnc3	YPDnc4	EtOHnc1	EtOHnc2	Galnc1	Galnc2

13	KSS1	YGR040W	YPDcl2	YPDcl3	EtOHcl1	EtOHcl2	Galcl1	YPDnc1	YPDnc2	YPDnc3	YPDnc4	EtOHnc1	EtOHnc2	Galnc1	Galnc2
13	URA8	YJR103W	YPDcl2	YPDcl3	EtOHcl1	EtOHcl2	Galcl1	YPDnc1	YPDnc2	YPDnc3	YPDnc4	EtOHnc1	EtOHnc2	Galnc1	Galnc2
13		YLL037W	YPDcl2	YPDcl3	EtOHcl1	EtOHcl2	Galcl1	YPDnc1	YPDnc2	YPDnc3	YPDnc4	EtOHnc1	EtOHnc2	Galnc1	Galnc2
13	ULS1	YOR191W	YPDcl2	YPDcl3	EtOHcl1	EtOHcl2	Galcl1	YPDnc1	YPDnc2	YPDnc3	YPDnc4	EtOHnc1	EtOHnc2	Galnc1	Galnc2
13	IRC11	YOR013W	YPDcl2	YPDcl3	EtOHcl1	EtOHcl2	Galcl1	YPDnc1	YPDnc2	YPDnc3	YPDnc4	EtOHnc1	EtOHnc2	Galnc1	Galnc2
13	YPT32	YGL210W	YPDcl2	YPDcl3	EtOHcl1	EtOHcl2	Galcl1	YPDnc1	YPDnc2	YPDnc3	YPDnc4	EtOHnc1	EtOHnc2	Galnc1	Galnc2
13		YFL051C	YPDcl2	YPDcl3	EtOHcl1	EtOHcl2	Galcl1	YPDnc1	YPDnc2	YPDnc3	YPDnc4	EtOHnc1	EtOHnc2	Galnc1	Galnc2
13		YDR413C	YPDcl2	YPDcl3	EtOHcl1	EtOHcl2	Galcl1	YPDnc1	YPDnc2	YPDnc3	YPDnc4	EtOHnc1	EtOHnc2	Galnc1	Galnc2
13	RRM3	YHR031C	YPDcl2	YPDcl3	EtOHcl1	EtOHcl2	Galcl1	YPDnc1	YPDnc2	YPDnc3	YPDnc4	EtOHnc1	EtOHnc2	Galnc1	Galnc2
13	ILM1	YJR118C	YPDcl2	YPDcl3	EtOHcl1	EtOHcl2	Galcl1	YPDnc1	YPDnc2	YPDnc3	YPDnc4	EtOHnc1	EtOHnc2	Galnc1	Galnc2
13		YIR040C	YPDcl2	YPDcl3	EtOHcl1	EtOHcl2	Galcl1	YPDnc1	YPDnc2	YPDnc3	YPDnc4	EtOHnc1	EtOHnc2	Galnc1	Galnc2
13		YMR031W-	YPDcl2	YPDcl3	EtOHcl1	EtOHcl2	Galcl1	YPDnc1	YPDnc2	YPDnc3	YPDnc4	EtOHnc1	EtOHnc2	Galnc1	Galnc2
13	SCW4	YGR279C	YPDcl2	YPDcl3	EtOHcl1	EtOHcl2	Galcl1	YPDnc1	YPDnc2	YPDnc3	YPDnc4	EtOHnc1	EtOHnc2	Galnc1	Galnc2
13	BSD2	YBR290W	YPDcl2	YPDcl3	EtOHcl1	EtOHcl2	Galcl1	YPDnc1	YPDnc2	YPDnc3	YPDnc4	EtOHnc1	EtOHnc2	Galnc1	Galnc2
13	ECM40	YMR062C	YPDcl2	YPDcl3	EtOHcl1	EtOHcl2	Galcl1	YPDnc1	YPDnc2	YPDnc3	YPDnc4	EtOHnc1	EtOHnc2	Galnc1	Galnc2
13	SGM1	YJR134C	YPDcl2	YPDcl3	EtOHcl1	EtOHcl2	Galcl1	YPDnc1	YPDnc2	YPDnc3	YPDnc4	EtOHnc1	EtOHnc2	Galnc1	Galnc2
13	FRE6	YLL051C	YPDcl2	YPDcl3	EtOHcl1	EtOHcl2	Galcl1	YPDnc1	YPDnc2	YPDnc3	YPDnc4	EtOHnc1	EtOHnc2	Galnc1	Galnc2
13		YDL094C	YPDcl2	YPDcl3	EtOHcl1	EtOHcl2	Galcl1	YPDnc1	YPDnc2	YPDnc3	YPDnc4	EtOHnc1	EtOHnc2	Galnc1	Galnc2
13	SRN2	YLR119W	YPDcl2	YPDcl3	EtOHcl1	EtOHcl2	Galcl1	YPDnc1	YPDnc2	YPDnc3	YPDnc4	EtOHnc1	EtOHnc2	Galnc1	Galnc2
13	SEE1	YIL064W	YPDcl2	YPDcl3	EtOHcl1	EtOHcl2	Galcl1	YPDnc1	YPDnc2	YPDnc3	YPDnc4	EtOHnc1	EtOHnc2	Galnc1	Galnc2
13	MEP2	YNL142W	YPDcl2	YPDcl3	EtOHcl1	EtOHcl2	Galcl1	YPDnc1	YPDnc2	YPDnc3	YPDnc4	EtOHnc1	EtOHnc2	Galnc1	Galnc2
13	FSH2	YMR222C	YPDcl2	YPDcl3	EtOHcl1	EtOHcl2	Galcl1	YPDnc1	YPDnc2	YPDnc3	YPDnc4	EtOHnc1	EtOHnc2	Galnc1	Galnc2
13	DCD1	YHR144C	YPDcl2	YPDcl3	EtOHcl1	EtOHcl2	Galcl1	YPDnc1	YPDnc2	YPDnc3	YPDnc4	EtOHnc1	EtOHnc2	Galnc1	Galnc2
13		YBR178W	YPDcl2	YPDcl3	EtOHcl1	EtOHcl2	Galcl1	YPDnc1	YPDnc2	YPDnc3	YPDnc4	EtOHnc1	EtOHnc2	Galnc1	Galnc2
13		YNL311C	YPDcl2	YPDcl3	EtOHcl1	EtOHcl2	Galcl1	YPDnc1	YPDnc2	YPDnc3	YPDnc4	EtOHnc1	EtOHnc2	Galnc1	Galnc2
13	ARV1	YLR242C	YPDcl2	YPDcl3	EtOHcl1	EtOHcl2	Galcl1	YPDnc1	YPDnc2	YPDnc3	YPDnc4	EtOHnc1	EtOHnc2	Galnc1	Galnc2
13	HMF1	YER057C	YPDcl2	YPDcl3	EtOHcl1	EtOHcl2	Galcl1	YPDnc1	YPDnc2	YPDnc3	YPDnc4	EtOHnc1	EtOHnc2	Galnc1	Galnc2
13	LUG1	YCR087C-A	YPDcl2	YPDcl3	EtOHcl1	EtOHcl2	Galcl1	YPDnc1	YPDnc2	YPDnc3	YPDnc4	EtOHnc1	EtOHnc2	Galnc1	Galnc2
13	USA1	YML029W	YPDcl2	YPDcl3	EtOHcl1	EtOHcl2	Galcl1	YPDnc1	YPDnc2	YPDnc3	YPDnc4	EtOHnc1	EtOHnc2	Galnc1	Galnc2
13	VRP1	YLR337C	YPDcl2	YPDcl3	EtOHcl1	EtOHcl2	Galcl1	YPDnc1	YPDnc2	YPDnc3	YPDnc4	EtOHnc1	EtOHnc2	Galnc1	Galnc2
13	POP5	YAL033W	YPDcl2	YPDcl3	EtOHcl1	EtOHcl2	Galcl1	YPDnc1	YPDnc2	YPDnc3	YPDnc4	EtOHnc1	EtOHnc2	Galnc1	Galnc2
13	FRE4	YNR060W	YPDcl2	YPDcl3	EtOHcl1	EtOHcl2	Galcl1	YPDnc1	YPDnc2	YPDnc3	YPDnc4	EtOHnc1	EtOHnc2	Galnc1	Galnc2
13	MMS2	YGL087C	YPDcl2	YPDcl3	EtOHcl1	EtOHcl2	Galcl1	YPDnc1	YPDnc2	YPDnc3	YPDnc4	EtOHnc1	EtOHnc2	Galnc1	Galnc2
13		YML037C	YPDcl2	YPDcl3	EtOHcl1	EtOHcl2	Galcl1	YPDnc1	YPDnc2	YPDnc3	YPDnc4	EtOHnc1	EtOHnc2	Galnc1	Galnc2

13	DUN1	YDL101C	YPDcl2	YPDcl3	EtOHcl1	EtOHcl2	Galcl1	YPDnc1	YPDnc2	YPDnc3	YPDnc4	EtOHnc1	EtOHnc2	Galnc1	Galnc2
13	REF2	YDR195W	YPDcl2	YPDcl3	EtOHcl1	EtOHcl2	Galcl1	YPDnc1	YPDnc2	YPDnc3	YPDnc4	EtOHnc1	EtOHnc2	Galnc1	Galnc2
13	POL3	YDL102W	YPDcl2	YPDcl3	EtOHcl1	EtOHcl2	Galcl1	YPDnc1	YPDnc2	YPDnc3	YPDnc4	EtOHnc1	EtOHnc2	Galnc1	Galnc2
13		YOR170W	YPDcl2	YPDcl3	EtOHcl1	EtOHcl2	Galcl1	YPDnc1	YPDnc2	YPDnc3	YPDnc4	EtOHnc1	EtOHnc2	Galnc1	Galnc2
13	RNH70	YGR276C	YPDcl2	YPDcl3	EtOHcl1	EtOHcl2	Galcl1	YPDnc1	YPDnc2	YPDnc3	YPDnc4	EtOHnc1	EtOHnc2	Galnc1	Galnc2
13		YLR363W-A	YPDcl2	YPDcl3	EtOHcl1	EtOHcl2	Galcl1	YPDnc1	YPDnc2	YPDnc3	YPDnc4	EtOHnc1	EtOHnc2	Galnc1	Galnc2
13	MRPL25	YGR076C	YPDcl2	YPDcl3	EtOHcl1	EtOHcl2	Galcl1	YPDnc1	YPDnc2	YPDnc3	YPDnc4	EtOHnc1	EtOHnc2	Galnc1	Galnc2
13	MAK31	YCR020C-A	YPDcl2	YPDcl3	EtOHcl1	EtOHcl2	Galcl1	YPDnc1	YPDnc2	YPDnc3	YPDnc4	EtOHnc1	EtOHnc2	Galnc1	Galnc2
13	BPL1	YDL141W	YPDcl2	YPDcl3	EtOHcl1	EtOHcl2	Galcl1	YPDnc1	YPDnc2	YPDnc3	YPDnc4	EtOHnc1	EtOHnc2	Galnc1	Galnc2
13		YMR075C-A	YPDcl2	YPDcl3	EtOHcl1	EtOHcl2	Galcl1	YPDnc1	YPDnc2	YPDnc3	YPDnc4	EtOHnc1	EtOHnc2	Galnc1	Galnc2
13	STP1	YDR463W	YPDcl2	YPDcl3	EtOHcl1	EtOHcl2	Galcl1	YPDnc1	YPDnc2	YPDnc3	YPDnc4	EtOHnc1	EtOHnc2	Galnc1	Galnc2
13		YPL168W	YPDcl2	YPDcl3	EtOHcl1	EtOHcl2	Galcl1	YPDnc1	YPDnc2	YPDnc3	YPDnc4	EtOHnc1	EtOHnc2	Galnc1	Galnc2
13	SKP1	YDR328C	YPDcl2	YPDcl3	EtOHcl1	EtOHcl2	Galcl1	YPDnc1	YPDnc2	YPDnc3	YPDnc4	EtOHnc1	EtOHnc2	Galnc1	Galnc2
13		YGR021W	YPDcl2	YPDcl3	EtOHcl1	EtOHcl2	Galcl1	YPDnc1	YPDnc2	YPDnc3	YPDnc4	EtOHnc1	EtOHnc2	Galnc1	Galnc2
13	SUI3	YPL237W	YPDcl2	YPDcl3	EtOHcl1	EtOHcl2	Galcl1	YPDnc1	YPDnc2	YPDnc3	YPDnc4	EtOHnc1	EtOHnc2	Galnc1	Galnc2
13	TRM2	YKR056W	YPDcl2	YPDcl3	EtOHcl1	EtOHcl2	Galcl1	YPDnc1	YPDnc2	YPDnc3	YPDnc4	EtOHnc1	EtOHnc2	Galnc1	Galnc2
13	MRE11	YMR224C	YPDcl2	YPDcl3	EtOHcl1	EtOHcl2	Galcl1	YPDnc1	YPDnc2	YPDnc3	YPDnc4	EtOHnc1	EtOHnc2	Galnc1	Galnc2
13	COG4	YPR105C	YPDcl2	YPDcl3	EtOHcl1	EtOHcl2	Galcl1	YPDnc1	YPDnc2	YPDnc3	YPDnc4	EtOHnc1	EtOHnc2	Galnc1	Galnc2
13		YKL030W	YPDcl2	YPDcl3	EtOHcl1	EtOHcl2	Galcl1	YPDnc1	YPDnc2	YPDnc3	YPDnc4	EtOHnc1	EtOHnc2	Galnc1	Galnc2
13	SEN54	YPL083C	YPDcl2	YPDcl3	EtOHcl1	EtOHcl2	Galcl1	YPDnc1	YPDnc2	YPDnc3	YPDnc4	EtOHnc1	EtOHnc2	Galnc1	Galnc2
13	CMC2	YBL059C-A	YPDcl2	YPDcl3	EtOHcl1	EtOHcl2	Galcl1	YPDnc1	YPDnc2	YPDnc3	YPDnc4	EtOHnc1	EtOHnc2	Galnc1	Galnc2
13		YGL217C	YPDcl2	YPDcl3	EtOHcl1	EtOHcl2	Galcl1	YPDnc1	YPDnc2	YPDnc3	YPDnc4	EtOHnc1	EtOHnc2	Galnc1	Galnc2
13	ARG8	YOL140W	YPDcl2	YPDcl3	EtOHcl1	EtOHcl2	Galcl1	YPDnc1	YPDnc2	YPDnc3	YPDnc4	EtOHnc1	EtOHnc2	Galnc1	Galnc2
13	NOC2	YOR206W	YPDcl2	YPDcl3	EtOHcl1	EtOHcl2	Galcl1	YPDnc1	YPDnc2	YPDnc3	YPDnc4	EtOHnc1	EtOHnc2	Galnc1	Galnc2
13	DSS4	YPR017C	YPDcl2	YPDcl3	EtOHcl1	EtOHcl2	Galcl1	YPDnc1	YPDnc2	YPDnc3	YPDnc4	EtOHnc1	EtOHnc2	Galnc1	Galnc2
13	ZRG17	YNR039C	YPDcl2	YPDcl3	EtOHcl1	EtOHcl2	Galcl1	YPDnc1	YPDnc2	YPDnc3	YPDnc4	EtOHnc1	EtOHnc2	Galnc1	Galnc2
13	FLO9	YAL063C	YPDcl2	YPDcl3	EtOHcl1	EtOHcl2	Galcl1	YPDnc1	YPDnc2	YPDnc3	YPDnc4	EtOHnc1	EtOHnc2	Galnc1	Galnc2
13	GCN2	YDR283C	YPDcl2	YPDcl3	EtOHcl1	EtOHcl2	Galcl1	YPDnc1	YPDnc2	YPDnc3	YPDnc4	EtOHnc1	EtOHnc2	Galnc1	Galnc2
13	QCR9	YGR183C	YPDcl2	YPDcl3	EtOHcl1	EtOHcl2	Galcl1	YPDnc1	YPDnc2	YPDnc3	YPDnc4	EtOHnc1	EtOHnc2	Galnc1	Galnc2
13	RPS16A	YMR143W	YPDcl2	YPDcl3	EtOHcl1	EtOHcl2	Galcl1	YPDnc1	YPDnc2	YPDnc3	YPDnc4	EtOHnc1	EtOHnc2	Galnc1	Galnc2
13	OKP1	YGR179C	YPDcl2	YPDcl3	EtOHcl1	EtOHcl2	Galcl1	YPDnc1	YPDnc2	YPDnc3	YPDnc4	EtOHnc1	EtOHnc2	Galnc1	Galnc2
13	PAU7	YAR020C	YPDcl2	YPDcl3	EtOHcl1	EtOHcl2	Galcl1	YPDnc1	YPDnc2	YPDnc3	YPDnc4	EtOHnc1	EtOHnc2	Galnc1	Galnc2
13	SIS2	YKR072C	YPDcl2	YPDcl3	EtOHcl1	EtOHcl2	Galcl1	YPDnc1	YPDnc2	YPDnc3	YPDnc4	EtOHnc1	EtOHnc2	Galnc1	Galnc2
13	IST1	YNL265C	YPDcl2	YPDcl3	EtOHcl1	EtOHcl2	Galcl1	YPDnc1	YPDnc2	YPDnc3	YPDnc4	EtOHnc1	EtOHnc2	Galnc1	Galnc2

13	TRP3	YKL211C	YPDcl2	YPDcl3	EtOHcl1	EtOHcl2	Galcl1	YPDnc1	YPDnc2	YPDnc3	YPDnc4	EtOHnc1	EtOHnc2	Galnc1	Galnc2
13	SKI3	YPR189W	YPDcl2	YPDcl3	EtOHcl1	EtOHcl2	Galcl1	YPDnc1	YPDnc2	YPDnc3	YPDnc4	EtOHnc1	EtOHnc2	Galnc1	Galnc2
13	QRI1	YDL103C	YPDcl2	YPDcl3	EtOHcl1	EtOHcl2	Galcl1	YPDnc1	YPDnc2	YPDnc3	YPDnc4	EtOHnc1	EtOHnc2	Galnc1	Galnc2
13	RPL1B	YGL135W	YPDcl2	YPDcl3	EtOHcl1	EtOHcl2	Galcl1	YPDnc1	YPDnc2	YPDnc3	YPDnc4	EtOHnc1	EtOHnc2	Galnc1	Galnc2
13	FBP26	YJL155C	YPDcl2	YPDcl3	EtOHcl1	EtOHcl2	Galcl1	YPDnc1	YPDnc2	YPDnc3	YPDnc4	EtOHnc1	EtOHnc2	Galnc1	Galnc2
13		YGL082W	YPDcl2	YPDcl3	EtOHcl1	EtOHcl2	Galcl1	YPDnc1	YPDnc2	YPDnc3	YPDnc4	EtOHnc1	EtOHnc2	Galnc1	Galnc2
13		YNR048W	YPDcl2	YPDcl3	EtOHcl1	EtOHcl2	Galcl1	YPDnc1	YPDnc2	YPDnc3	YPDnc4	EtOHnc1	EtOHnc2	Galnc1	Galnc2
13	SAM35	YHR083W	YPDcl2	YPDcl3	EtOHcl1	EtOHcl2	Galcl1	YPDnc1	YPDnc2	YPDnc3	YPDnc4	EtOHnc1	EtOHnc2	Galnc1	Galnc2
13		YLL023C	YPDcl2	YPDcl3	EtOHcl1	EtOHcl2	Galcl1	YPDnc1	YPDnc2	YPDnc3	YPDnc4	EtOHnc1	EtOHnc2	Galnc1	Galnc2
13		YLR202C	YPDcl2	YPDcl3	EtOHcl1	EtOHcl2	Galcl1	YPDnc1	YPDnc2	YPDnc3	YPDnc4	EtOHnc1	EtOHnc2	Galnc1	Galnc2
13	SAD1	YFR005C	YPDcl2	YPDcl3	EtOHcl1	EtOHcl2	Galcl1	YPDnc1	YPDnc2	YPDnc3	YPDnc4	EtOHnc1	EtOHnc2	Galnc1	Galnc2
13	YBP1	YBR216C	YPDcl2	YPDcl3	EtOHcl1	EtOHcl2	Galcl1	YPDnc1	YPDnc2	YPDnc3	YPDnc4	EtOHnc1	EtOHnc2	Galnc1	Galnc2
13		YGR265W	YPDcl2	YPDcl3	EtOHcl1	EtOHcl2	Galcl1	YPDnc1	YPDnc2	YPDnc3	YPDnc4	EtOHnc1	EtOHnc2	Galnc1	Galnc2
13	TIM9	YEL020W-A	YPDcl2	YPDcl3	EtOHcl1	EtOHcl2	Galcl1	YPDnc1	YPDnc2	YPDnc3	YPDnc4	EtOHnc1	EtOHnc2	Galnc1	Galnc2
13	UTR4	YEL038W	YPDcl2	YPDcl3	EtOHcl1	EtOHcl2	Galcl1	YPDnc1	YPDnc2	YPDnc3	YPDnc4	EtOHnc1	EtOHnc2	Galnc1	Galnc2
13	RRP43	YCR035C	YPDcl2	YPDcl3	EtOHcl1	EtOHcl2	Galcl1	YPDnc1	YPDnc2	YPDnc3	YPDnc4	EtOHnc1	EtOHnc2	Galnc1	Galnc2
13	PEX12	YMR026C	YPDcl2	YPDcl3	EtOHcl1	EtOHcl2	Galcl1	YPDnc1	YPDnc2	YPDnc3	YPDnc4	EtOHnc1	EtOHnc2	Galnc1	Galnc2
13	NGG1	YDR176W	YPDcl2	YPDcl3	EtOHcl1	EtOHcl2	Galcl1	YPDnc1	YPDnc2	YPDnc3	YPDnc4	EtOHnc1	EtOHnc2	Galnc1	Galnc2
13	RPN14	YGL004C	YPDcl2	YPDcl3	EtOHcl1	EtOHcl2	Galcl1	YPDnc1	YPDnc2	YPDnc3	YPDnc4	EtOHnc1	EtOHnc2	Galnc1	Galnc2
13		YDR222W	YPDcl2	YPDcl3	EtOHcl1	EtOHcl2	Galcl1	YPDnc1	YPDnc2	YPDnc3	YPDnc4	EtOHnc1	EtOHnc2	Galnc1	Galnc2
13	ORC4	YPR162C	YPDcl2	YPDcl3	EtOHcl1	EtOHcl2	Galcl1	YPDnc1	YPDnc2	YPDnc3	YPDnc4	EtOHnc1	EtOHnc2	Galnc1	Galnc2
13	MCD4	YKL165C	YPDcl2	YPDcl3	EtOHcl1	EtOHcl2	Galcl1	YPDnc1	YPDnc2	YPDnc3	YPDnc4	EtOHnc1	EtOHnc2	Galnc1	Galnc2
13	UBX3	YDL091C	YPDcl2	YPDcl3	EtOHcl1	EtOHcl2	Galcl1	YPDnc1	YPDnc2	YPDnc3	YPDnc4	EtOHnc1	EtOHnc2	Galnc1	Galnc2
13	VPS35	YJL154C	YPDcl2	YPDcl3	EtOHcl1	EtOHcl2	Galcl1	YPDnc1	YPDnc2	YPDnc3	YPDnc4	EtOHnc1	EtOHnc2	Galnc1	Galnc2
13		YDR220C	YPDcl2	YPDcl3	EtOHcl1	EtOHcl2	Galcl1	YPDnc1	YPDnc2	YPDnc3	YPDnc4	EtOHnc1	EtOHnc2	Galnc1	Galnc2
13	TPS2	YDR074W	YPDcl2	YPDcl3	EtOHcl1	EtOHcl2	Galcl1	YPDnc1	YPDnc2	YPDnc3	YPDnc4	EtOHnc1	EtOHnc2	Galnc1	Galnc2
13	THI2	YBR240C	YPDcl2	YPDcl3	EtOHcl1	EtOHcl2	Galcl1	YPDnc1	YPDnc2	YPDnc3	YPDnc4	EtOHnc1	EtOHnc2	Galnc1	Galnc2
13	PAM1	YDR251W	YPDcl2	YPDcl3	EtOHcl1	EtOHcl2	Galcl1	YPDnc1	YPDnc2	YPDnc3	YPDnc4	EtOHnc1	EtOHnc2	Galnc1	Galnc2
13	UTP9	YHR196W	YPDcl2	YPDcl3	EtOHcl1	EtOHcl2	Galcl1	YPDnc1	YPDnc2	YPDnc3	YPDnc4	EtOHnc1	EtOHnc2	Galnc1	Galnc2
13	GPI10	YGL142C	YPDcl2	YPDcl3	EtOHcl1	EtOHcl2	Galcl1	YPDnc1	YPDnc2	YPDnc3	YPDnc4	EtOHnc1	EtOHnc2	Galnc1	Galnc2
13	MHT1	YLL062C	YPDcl2	YPDcl3	EtOHcl1	EtOHcl2	Galcl1	YPDnc1	YPDnc2	YPDnc3	YPDnc4	EtOHnc1	EtOHnc2	Galnc1	Galnc2
13	PTC3	YBL056W	YPDcl2	YPDcl3	EtOHcl1	EtOHcl2	Galcl1	YPDnc1	YPDnc2	YPDnc3	YPDnc4	EtOHnc1	EtOHnc2	Galnc1	Galnc2
13	PTC2	YER089C	YPDcl2	YPDcl3	EtOHcl1	EtOHcl2	Galcl1	YPDnc1	YPDnc2	YPDnc3	YPDnc4	EtOHnc1	EtOHnc2	Galnc1	Galnc2
13	AIR2	YDL175C	YPDcl2	YPDcl3	EtOHcl1	EtOHcl2	Galcl1	YPDnc1	YPDnc2	YPDnc3	YPDnc4	EtOHnc1	EtOHnc2	Galnc1	Galnc2

13	KRE5	YOR336W	YPDcl2	YPDcl3	EtOHcl1	EtOHcl2	Galcl1	YPDnc1	YPDnc2	YPDnc3	YPDnc4	EtOHnc1	EtOHnc2	Galnc1	Galnc2
13		YKL100W-A	YPDcl2	YPDcl3	EtOHcl1	EtOHcl2	Galcl1	YPDnc1	YPDnc2	YPDnc3	YPDnc4	EtOHnc1	EtOHnc2	Galnc1	Galnc2
13		YML131W	YPDcl2	YPDcl3	EtOHcl1	EtOHcl2	Galcl1	YPDnc1	YPDnc2	YPDnc3	YPDnc4	EtOHnc1	EtOHnc2	Galnc1	Galnc2
13	THO2	YNL139C	YPDcl2	YPDcl3	EtOHcl1	EtOHcl2	Galcl1	YPDnc1	YPDnc2	YPDnc3	YPDnc4	EtOHnc1	EtOHnc2	Galnc1	Galnc2
13		YHR192W	YPDcl2	YPDcl3	EtOHcl1	EtOHcl2	Galcl1	YPDnc1	YPDnc2	YPDnc3	YPDnc4	EtOHnc1	EtOHnc2	Galnc1	Galnc2
13		YNL313C	YPDcl2	YPDcl3	EtOHcl1	EtOHcl2	Galcl1	YPDnc1	YPDnc2	YPDnc3	YPDnc4	EtOHnc1	EtOHnc2	Galnc1	Galnc2
13		YCR051W	YPDcl2	YPDcl3	EtOHcl1	EtOHcl2	Galcl1	YPDnc1	YPDnc2	YPDnc3	YPDnc4	EtOHnc1	EtOHnc2	Galnc1	Galnc2
13	RIM4	YHL024W	YPDcl2	YPDcl3	EtOHcl1	EtOHcl2	Galcl1	YPDnc1	YPDnc2	YPDnc3	YPDnc4	EtOHnc1	EtOHnc2	Galnc1	Galnc2
13		YJL185C	YPDcl2	YPDcl3	EtOHcl1	EtOHcl2	Galcl1	YPDnc1	YPDnc2	YPDnc3	YPDnc4	EtOHnc1	EtOHnc2	Galnc1	Galnc2
13	RPL3	YOR063W	YPDcl2	YPDcl3	EtOHcl1	EtOHcl2	Galcl1	YPDnc1	YPDnc2	YPDnc3	YPDnc4	EtOHnc1	EtOHnc2	Galnc1	Galnc2
13	GPI2	YPL076W	YPDcl2	YPDcl3	EtOHcl1	EtOHcl2	Galcl1	YPDnc1	YPDnc2	YPDnc3	YPDnc4	EtOHnc1	EtOHnc2	Galnc1	Galnc2
13	MPH1	YIR002C	YPDcl2	YPDcl3	EtOHcl1	EtOHcl2	Galcl1	YPDnc1	YPDnc2	YPDnc3	YPDnc4	EtOHnc1	EtOHnc2	Galnc1	Galnc2
13	MEI4	YER044C-A	YPDcl2	YPDcl3	EtOHcl1	EtOHcl2	Galcl1	YPDnc1	YPDnc2	YPDnc3	YPDnc4	EtOHnc1	EtOHnc2	Galnc1	Galnc2
13	VID28	YIL017C	YPDcl2	YPDcl3	EtOHcl1	EtOHcl2	Galcl1	YPDnc1	YPDnc2	YPDnc3	YPDnc4	EtOHnc1	EtOHnc2	Galnc1	Galnc2
13	COQ5	YML110C	YPDcl2	YPDcl3	EtOHcl1	EtOHcl2	Galcl1	YPDnc1	YPDnc2	YPDnc3	YPDnc4	EtOHnc1	EtOHnc2	Galnc1	Galnc2
13	ROX1	YPR065W	YPDcl2	YPDcl3	EtOHcl1	EtOHcl2	Galcl1	YPDnc1	YPDnc2	YPDnc3	YPDnc4	EtOHnc1	EtOHnc2	Galnc1	Galnc2
13	MEC3	YLR288C	YPDcl2	YPDcl3	EtOHcl1	EtOHcl2	Galcl1	YPDnc1	YPDnc2	YPDnc3	YPDnc4	EtOHnc1	EtOHnc2	Galnc1	Galnc2
13	TOK1	YJL093C	YPDcl2	YPDcl3	EtOHcl1	EtOHcl2	Galcl1	YPDnc1	YPDnc2	YPDnc3	YPDnc4	EtOHnc1	EtOHnc2	Galnc1	Galnc2
13	BSC5	YNR069C	YPDcl2	YPDcl3	EtOHcl1	EtOHcl2	Galcl1	YPDnc1	YPDnc2	YPDnc3	YPDnc4	EtOHnc1	EtOHnc2	Galnc1	Galnc2
13	PWP2	YCR057C	YPDcl2	YPDcl3	EtOHcl1	EtOHcl2	Galcl1	YPDnc1	YPDnc2	YPDnc3	YPDnc4	EtOHnc1	EtOHnc2	Galnc1	Galnc2
13	ACF4	YJR083C	YPDcl2	YPDcl3	EtOHcl1	EtOHcl2	Galcl1	YPDnc1	YPDnc2	YPDnc3	YPDnc4	EtOHnc1	EtOHnc2	Galnc1	Galnc2
13	PEX21	YGR239C	YPDcl2	YPDcl3	EtOHcl1	EtOHcl2	Galcl1	YPDnc1	YPDnc2	YPDnc3	YPDnc4	EtOHnc1	EtOHnc2	Galnc1	Galnc2
13	YPT31	YER031C	YPDcl2	YPDcl3	EtOHcl1	EtOHcl2	Galcl1	YPDnc1	YPDnc2	YPDnc3	YPDnc4	EtOHnc1	EtOHnc2	Galnc1	Galnc2
13	WSC2	YNL283C	YPDcl2	YPDcl3	EtOHcl1	EtOHcl2	Galcl1	YPDnc1	YPDnc2	YPDnc3	YPDnc4	EtOHnc1	EtOHnc2	Galnc1	Galnc2
13	OPI10	YOL032W	YPDcl2	YPDcl3	EtOHcl1	EtOHcl2	Galcl1	YPDnc1	YPDnc2	YPDnc3	YPDnc4	EtOHnc1	EtOHnc2	Galnc1	Galnc2
13		YLR194C	YPDcl2	YPDcl3	EtOHcl1	EtOHcl2	Galcl1	YPDnc1	YPDnc2	YPDnc3	YPDnc4	EtOHnc1	EtOHnc2	Galnc1	Galnc2
13	CCA1	YER168C	YPDcl2	YPDcl3	EtOHcl1	EtOHcl2	Galcl1	YPDnc1	YPDnc2	YPDnc3	YPDnc4	EtOHnc1	EtOHnc2	Galnc1	Galnc2
13		YML031C-A	YPDcl2	YPDcl3	EtOHcl1	EtOHcl2	Galcl1	YPDnc1	YPDnc2	YPDnc3	YPDnc4	EtOHnc1	EtOHnc2	Galnc1	Galnc2
13	TFB5	YDR079C-A	YPDcl2	YPDcl3	EtOHcl1	EtOHcl2	Galcl1	YPDnc1	YPDnc2	YPDnc3	YPDnc4	EtOHnc1	EtOHnc2	Galnc1	Galnc2
13		YMR181C	YPDcl2	YPDcl3	EtOHcl1	EtOHcl2	Galcl1	YPDnc1	YPDnc2	YPDnc3	YPDnc4	EtOHnc1	EtOHnc2	Galnc1	Galnc2
13	SIR2	YDL042C	YPDcl2	YPDcl3	EtOHcl1	EtOHcl2	Galcl1	YPDnc1	YPDnc2	YPDnc3	YPDnc4	EtOHnc1	EtOHnc2	Galnc1	Galnc2
13		YNL109W	YPDcl2	YPDcl3	EtOHcl1	EtOHcl2	Galcl1	YPDnc1	YPDnc2	YPDnc3	YPDnc4	EtOHnc1	EtOHnc2	Galnc1	Galnc2
13	NTR2	YKR022C	YPDcl2	YPDcl3	EtOHcl1	EtOHcl2	Galcl1	YPDnc1	YPDnc2	YPDnc3	YPDnc4	EtOHnc1	EtOHnc2	Galnc1	Galnc2
13	ADH6	YMR318C	YPDcl2	YPDcl3	EtOHcl1	EtOHcl2	Galcl1	YPDnc1	YPDnc2	YPDnc3	YPDnc4	EtOHnc1	EtOHnc2	Galnc1	Galnc2

13	MAG1	YER142C	YPDcl2	YPDcl3	EtOHcl1	EtOHcl2	Galcl1	YPDnc1	YPDnc2	YPDnc3	YPDnc4	EtOHnc1	EtOHnc2	Galnc1	Galnc2
13	FIS1	YIL065C	YPDcl2	YPDcl3	EtOHcl1	EtOHcl2	Galcl1	YPDnc1	YPDnc2	YPDnc3	YPDnc4	EtOHnc1	EtOHnc2	Galnc1	Galnc2
13	MDM36	YPR083W	YPDcl2	YPDcl3	EtOHcl1	EtOHcl2	Galcl1	YPDnc1	YPDnc2	YPDnc3	YPDnc4	EtOHnc1	EtOHnc2	Galnc1	Galnc2
13	RIB1	YBL033C	YPDcl2	YPDcl3	EtOHcl1	EtOHcl2	Galcl1	YPDnc1	YPDnc2	YPDnc3	YPDnc4	EtOHnc1	EtOHnc2	Galnc1	Galnc2
13		YDL159W-A	YPDcl2	YPDcl3	EtOHcl1	EtOHcl2	Galcl1	YPDnc1	YPDnc2	YPDnc3	YPDnc4	EtOHnc1	EtOHnc2	Galnc1	Galnc2
13	PRD1	YCL057W	YPDcl2	YPDcl3	EtOHcl1	EtOHcl2	Galcl1	YPDnc1	YPDnc2	YPDnc3	YPDnc4	EtOHnc1	EtOHnc2	Galnc1	Galnc2
13	TMA64	YDR117C	YPDcl2	YPDcl3	EtOHcl1	EtOHcl2	Galcl1	YPDnc1	YPDnc2	YPDnc3	YPDnc4	EtOHnc1	EtOHnc2	Galnc1	Galnc2
13	CDD1	YLR245C	YPDcl2	YPDcl3	EtOHcl1	EtOHcl2	Galcl1	YPDnc1	YPDnc2	YPDnc3	YPDnc4	EtOHnc1	EtOHnc2	Galnc1	Galnc2
13	KIP3	YGL216W	YPDcl2	YPDcl3	EtOHcl1	EtOHcl2	Galcl1	YPDnc1	YPDnc2	YPDnc3	YPDnc4	EtOHnc1	EtOHnc2	Galnc1	Galnc2
13	RPL37A	YLR185W	YPDcl2	YPDcl3	EtOHcl1	EtOHcl2	Galcl1	YPDnc1	YPDnc2	YPDnc3	YPDnc4	EtOHnc1	EtOHnc2	Galnc1	Galnc2
13	MRN1	YPL184C	YPDcl2	YPDcl3	EtOHcl1	EtOHcl2	Galcl1	YPDnc1	YPDnc2	YPDnc3	YPDnc4	EtOHnc1	EtOHnc2	Galnc1	Galnc2
13	IZH1	YDR492W	YPDcl2	YPDcl3	EtOHcl1	EtOHcl2	Galcl1	YPDnc1	YPDnc2	YPDnc3	YPDnc4	EtOHnc1	EtOHnc2	Galnc1	Galnc2
13	CAF40	YNL288W	YPDcl2	YPDcl3	EtOHcl1	EtOHcl2	Galcl1	YPDnc1	YPDnc2	YPDnc3	YPDnc4	EtOHnc1	EtOHnc2	Galnc1	Galnc2
13	TPC1	YGR096W	YPDcl2	YPDcl3	EtOHcl1	EtOHcl2	Galcl1	YPDnc1	YPDnc2	YPDnc3	YPDnc4	EtOHnc1	EtOHnc2	Galnc1	Galnc2
13	PMD1	YER132C	YPDcl2	YPDcl3	EtOHcl1	EtOHcl2	Galcl1	YPDnc1	YPDnc2	YPDnc3	YPDnc4	EtOHnc1	EtOHnc2	Galnc1	Galnc2
13		YHR202W	YPDcl2	YPDcl3	EtOHcl1	EtOHcl2	Galcl1	YPDnc1	YPDnc2	YPDnc3	YPDnc4	EtOHnc1	EtOHnc2	Galnc1	Galnc2
13	URB2	YJR041C	YPDcl2	YPDcl3	EtOHcl1	EtOHcl2	Galcl1	YPDnc1	YPDnc2	YPDnc3	YPDnc4	EtOHnc1	EtOHnc2	Galnc1	Galnc2
13		YKR045C	YPDcl2	YPDcl3	EtOHcl1	EtOHcl2	Galcl1	YPDnc1	YPDnc2	YPDnc3	YPDnc4	EtOHnc1	EtOHnc2	Galnc1	Galnc2
13	APL4	YPR029C	YPDcl2	YPDcl3	EtOHcl1	EtOHcl2	Galcl1	YPDnc1	YPDnc2	YPDnc3	YPDnc4	EtOHnc1	EtOHnc2	Galnc1	Galnc2
13	HXT14	YNL318C	YPDcl2	YPDcl3	EtOHcl1	EtOHcl2	Galcl1	YPDnc1	YPDnc2	YPDnc3	YPDnc4	EtOHnc1	EtOHnc2	Galnc1	Galnc2
13		YLL056C	YPDcl2	YPDcl3	EtOHcl1	EtOHcl2	Galcl1	YPDnc1	YPDnc2	YPDnc3	YPDnc4	EtOHnc1	EtOHnc2	Galnc1	Galnc2
13	SET6	YPL165C	YPDcl2	YPDcl3	EtOHcl1	EtOHcl2	Galcl1	YPDnc1	YPDnc2	YPDnc3	YPDnc4	EtOHnc1	EtOHnc2	Galnc1	Galnc2
13	SEC12	YNR026C	YPDcl2	YPDcl3	EtOHcl1	EtOHcl2	Galcl1	YPDnc1	YPDnc2	YPDnc3	YPDnc4	EtOHnc1	EtOHnc2	Galnc1	Galnc2
13		YLR120W-A	YPDcl2	YPDcl3	EtOHcl1	EtOHcl2	Galcl1	YPDnc1	YPDnc2	YPDnc3	YPDnc4	EtOHnc1	EtOHnc2	Galnc1	Galnc2
13	FMP49	YER038W-A	YPDcl2	YPDcl3	EtOHcl1	EtOHcl2	Galcl1	YPDnc1	YPDnc2	YPDnc3	YPDnc4	EtOHnc1	EtOHnc2	Galnc1	Galnc2
13		YMR013W-	YPDcl2	YPDcl3	EtOHcl1	EtOHcl2	Galcl1	YPDnc1	YPDnc2	YPDnc3	YPDnc4	EtOHnc1	EtOHnc2	Galnc1	Galnc2
13		YOR302W	YPDcl2	YPDcl3	EtOHcl1	EtOHcl2	Galcl1	YPDnc1	YPDnc2	YPDnc3	YPDnc4	EtOHnc1	EtOHnc2	Galnc1	Galnc2
13		YLR076C	YPDcl2	YPDcl3	EtOHcl1	EtOHcl2	Galcl1	YPDnc1	YPDnc2	YPDnc3	YPDnc4	EtOHnc1	EtOHnc2	Galnc1	Galnc2
13	TOS2	YGR221C	YPDcl2	YPDcl3	EtOHcl1	EtOHcl2	Galcl1	YPDnc1	YPDnc2	YPDnc3	YPDnc4	EtOHnc1	EtOHnc2	Galnc1	Galnc2
13	DPS1	YLL018C	YPDcl2	YPDcl3	EtOHcl1	EtOHcl2	Galcl1	YPDnc1	YPDnc2	YPDnc3	YPDnc4	EtOHnc1	EtOHnc2	Galnc1	Galnc2
13	HDA2	YDR295C	YPDcl2	YPDcl3	EtOHcl1	EtOHcl2	Galcl1	YPDnc1	YPDnc2	YPDnc3	YPDnc4	EtOHnc1	EtOHnc2	Galnc1	Galnc2
13	PGM1	YKL127W	YPDcl2	YPDcl3	EtOHcl1	EtOHcl2	Galcl1	YPDnc1	YPDnc2	YPDnc3	YPDnc4	EtOHnc1	EtOHnc2	Galnc1	Galnc2
13	AIM23	YJL131C	YPDcl2	YPDcl3	EtOHcl1	EtOHcl2	Galcl1	YPDnc1	YPDnc2	YPDnc3	YPDnc4	EtOHnc1	EtOHnc2	Galnc1	Galnc2
13	VPS68	YOL129W	YPDcl2	YPDcl3	EtOHcl1	EtOHcl2	Galcl1	YPDnc1	YPDnc2	YPDnc3	YPDnc4	EtOHnc1	EtOHnc2	Galnc1	Galnc2

13	POL1	YNL102W	YPDcl2	YPDcl3	EtOHcl1	EtOHcl2	Galcl1	YPDnc1	YPDnc2	YPDnc3	YPDnc4	EtOHnc1	EtOHnc2	Galnc1	Galnc2
13	TRM9	YML014W	YPDcl2	YPDcl3	EtOHcl1	EtOHcl2	Galcl1	YPDnc1	YPDnc2	YPDnc3	YPDnc4	EtOHnc1	EtOHnc2	Galnc1	Galnc2
13	MRPL7	YDR237W	YPDcl2	YPDcl3	EtOHcl1	EtOHcl2	Galcl1	YPDnc1	YPDnc2	YPDnc3	YPDnc4	EtOHnc1	EtOHnc2	Galnc1	Galnc2
13	FOX2	YKR009C	YPDcl2	YPDcl3	EtOHcl1	EtOHcl2	Galcl1	YPDnc1	YPDnc2	YPDnc3	YPDnc4	EtOHnc1	EtOHnc2	Galnc1	Galnc2
13		YPR039W	YPDcl2	YPDcl3	EtOHcl1	EtOHcl2	Galcl1	YPDnc1	YPDnc2	YPDnc3	YPDnc4	EtOHnc1	EtOHnc2	Galnc1	Galnc2
13	YME1	YPR024W	YPDcl2	YPDcl3	EtOHcl1	EtOHcl2	Galcl1	YPDnc1	YPDnc2	YPDnc3	YPDnc4	EtOHnc1	EtOHnc2	Galnc1	Galnc2
13		YOR289W	YPDcl2	YPDcl3	EtOHcl1	EtOHcl2	Galcl1	YPDnc1	YPDnc2	YPDnc3	YPDnc4	EtOHnc1	EtOHnc2	Galnc1	Galnc2
13	HSP60	YLR259C	YPDcl2	YPDcl3	EtOHcl1	EtOHcl2	Galcl1	YPDnc1	YPDnc2	YPDnc3	YPDnc4	EtOHnc1	EtOHnc2	Galnc1	Galnc2
13	FMP46	YKR049C	YPDcl2	YPDcl3	EtOHcl1	EtOHcl2	Galcl1	YPDnc1	YPDnc2	YPDnc3	YPDnc4	EtOHnc1	EtOHnc2	Galnc1	Galnc2
13	LHS1	YKL073W	YPDcl2	YPDcl3	EtOHcl1	EtOHcl2	Galcl1	YPDnc1	YPDnc2	YPDnc3	YPDnc4	EtOHnc1	EtOHnc2	Galnc1	Galnc2
13	SSN3	YPL042C	YPDcl2	YPDcl3	EtOHcl1	EtOHcl2	Galcl1	YPDnc1	YPDnc2	YPDnc3	YPDnc4	EtOHnc1	EtOHnc2	Galnc1	Galnc2
13	MRS3	YJL133W	YPDcl2	YPDcl3	EtOHcl1	EtOHcl2	Galcl1	YPDnc1	YPDnc2	YPDnc3	YPDnc4	EtOHnc1	EtOHnc2	Galnc1	Galnc2
13	ATP19	YOL077W-/	YPDcl2	YPDcl3	EtOHcl1	EtOHcl2	Galcl1	YPDnc1	YPDnc2	YPDnc3	YPDnc4	EtOHnc1	EtOHnc2	Galnc1	Galnc2
13	GLK1	YCL040W	YPDcl2	YPDcl3	EtOHcl1	EtOHcl2	Galcl1	YPDnc1	YPDnc2	YPDnc3	YPDnc4	EtOHnc1	EtOHnc2	Galnc1	Galnc2
13	KES1	YPL145C	YPDcl2	YPDcl3	EtOHcl1	EtOHcl2	Galcl1	YPDnc1	YPDnc2	YPDnc3	YPDnc4	EtOHnc1	EtOHnc2	Galnc1	Galnc2
13	LDB17	YDL146W	YPDcl2	YPDcl3	EtOHcl1	EtOHcl2	Galcl1	YPDnc1	YPDnc2	YPDnc3	YPDnc4	EtOHnc1	EtOHnc2	Galnc1	Galnc2
13	SAG1	YJR004C	YPDcl2	YPDcl3	EtOHcl1	EtOHcl2	Galcl1	YPDnc1	YPDnc2	YPDnc3	YPDnc4	EtOHnc1	EtOHnc2	Galnc1	Galnc2
13	PCH2	YBR186W	YPDcl2	YPDcl3	EtOHcl1	EtOHcl2	Galcl1	YPDnc1	YPDnc2	YPDnc3	YPDnc4	EtOHnc1	EtOHnc2	Galnc1	Galnc2
13	ERJ5	YFR041C	YPDcl2	YPDcl3	EtOHcl1	EtOHcl2	Galcl1	YPDnc1	YPDnc2	YPDnc3	YPDnc4	EtOHnc1	EtOHnc2	Galnc1	Galnc2
13	SFT1	YKL006C-A	YPDcl2	YPDcl3	EtOHcl1	EtOHcl2	Galcl1	YPDnc1	YPDnc2	YPDnc3	YPDnc4	EtOHnc1	EtOHnc2	Galnc1	Galnc2
13	AQY1	YPR192W	YPDcl2	YPDcl3	EtOHcl1	EtOHcl2	Galcl1	YPDnc1	YPDnc2	YPDnc3	YPDnc4	EtOHnc1	EtOHnc2	Galnc1	Galnc2
13	GCV2	YMR189W	YPDcl2	YPDcl3	EtOHcl1	EtOHcl2	Galcl1	YPDnc1	YPDnc2	YPDnc3	YPDnc4	EtOHnc1	EtOHnc2	Galnc1	Galnc2
13	SIA1	YOR137C	YPDcl2	YPDcl3	EtOHcl1	EtOHcl2	Galcl1	YPDnc1	YPDnc2	YPDnc3	YPDnc4	EtOHnc1	EtOHnc2	Galnc1	Galnc2
13	IES2	YNL215W	YPDcl2	YPDcl3	EtOHcl1	EtOHcl2	Galcl1	YPDnc1	YPDnc2	YPDnc3	YPDnc4	EtOHnc1	EtOHnc2	Galnc1	Galnc2
13	NSE4	YDL105W	YPDcl2	YPDcl3	EtOHcl1	EtOHcl2	Galcl1	YPDnc1	YPDnc2	YPDnc3	YPDnc4	EtOHnc1	EtOHnc2	Galnc1	Galnc2
13	BIK1	YCL029C	YPDcl2	YPDcl3	EtOHcl1	EtOHcl2	Galcl1	YPDnc1	YPDnc2	YPDnc3	YPDnc4	EtOHnc1	EtOHnc2	Galnc1	Galnc2
13	CWC27	YPL064C	YPDcl2	YPDcl3	EtOHcl1	EtOHcl2	Galcl1	YPDnc1	YPDnc2	YPDnc3	YPDnc4	EtOHnc1	EtOHnc2	Galnc1	Galnc2
13		YML054C-/	YPDcl2	YPDcl3	EtOHcl1	EtOHcl2	Galcl1	YPDnc1	YPDnc2	YPDnc3	YPDnc4	EtOHnc1	EtOHnc2	Galnc1	Galnc2
13	MKT1	YNL085W	YPDcl2	YPDcl3	EtOHcl1	EtOHcl2	Galcl1	YPDnc1	YPDnc2	YPDnc3	YPDnc4	EtOHnc1	EtOHnc2	Galnc1	Galnc2
13		YAL031W-/	YPDcl2	YPDcl3	EtOHcl1	EtOHcl2	Galcl1	YPDnc1	YPDnc2	YPDnc3	YPDnc4	EtOHnc1	EtOHnc2	Galnc1	Galnc2
13	HIS2	YFR025C	YPDcl2	YPDcl3	EtOHcl1	EtOHcl2	Galcl1	YPDnc1	YPDnc2	YPDnc3	YPDnc4	EtOHnc1	EtOHnc2	Galnc1	Galnc2
13	PDR17	YNL264C	YPDcl2	YPDcl3	EtOHcl1	EtOHcl2	Galcl1	YPDnc1	YPDnc2	YPDnc3	YPDnc4	EtOHnc1	EtOHnc2	Galnc1	Galnc2
13	IMP3	YHR148W	YPDcl2	YPDcl3	EtOHcl1	EtOHcl2	Galcl1	YPDnc1	YPDnc2	YPDnc3	YPDnc4	EtOHnc1	EtOHnc2	Galnc1	Galnc2
13		YIL165C	YPDcl2	YPDcl3	EtOHcl1	EtOHcl2	Galcl1	YPDnc1	YPDnc2	YPDnc3	YPDnc4	EtOHnc1	EtOHnc2	Galnc1	Galnc2

13	CYR1	YJL005W	YPDcl2	YPDcl3	EtOHcl1	EtOHcl2	Galcl1	YPDnc1	YPDnc2	YPDnc3	YPDnc4	EtOHnc1	EtOHnc2	Galnc1	Galnc2
13	STE5	YDR103W	YPDcl2	YPDcl3	EtOHcl1	EtOHcl2	Galcl1	YPDnc1	YPDnc2	YPDnc3	YPDnc4	EtOHnc1	EtOHnc2	Galnc1	Galnc2
13	ASF1	YJL115W	YPDcl2	YPDcl3	EtOHcl1	EtOHcl2	Galcl1	YPDnc1	YPDnc2	YPDnc3	YPDnc4	EtOHnc1	EtOHnc2	Galnc1	Galnc2
13	PDC1	YLR044C	YPDcl2	YPDcl3	EtOHcl1	EtOHcl2	Galcl1	YPDnc1	YPDnc2	YPDnc3	YPDnc4	EtOHnc1	EtOHnc2	Galnc1	Galnc2
13	MUS81	YDR386W	YPDcl2	YPDcl3	EtOHcl1	EtOHcl2	Galcl1	YPDnc1	YPDnc2	YPDnc3	YPDnc4	EtOHnc1	EtOHnc2	Galnc1	Galnc2
13	FMP33	YJL161W	YPDcl2	YPDcl3	EtOHcl1	EtOHcl2	Galcl1	YPDnc1	YPDnc2	YPDnc3	YPDnc4	EtOHnc1	EtOHnc2	Galnc1	Galnc2
13	JJJ3	YJR097W	YPDcl2	YPDcl3	EtOHcl1	EtOHcl2	Galcl1	YPDnc1	YPDnc2	YPDnc3	YPDnc4	EtOHnc1	EtOHnc2	Galnc1	Galnc2
13	MRS1	YIR021W	YPDcl2	YPDcl3	EtOHcl1	EtOHcl2	Galcl1	YPDnc1	YPDnc2	YPDnc3	YPDnc4	EtOHnc1	EtOHnc2	Galnc1	Galnc2
13		YCR047W-/	YPDcl2	YPDcl3	EtOHcl1	EtOHcl2	Galcl1	YPDnc1	YPDnc2	YPDnc3	YPDnc4	EtOHnc1	EtOHnc2	Galnc1	Galnc2
13	SEC65	YML105C	YPDcl2	YPDcl3	EtOHcl1	EtOHcl2	Galcl1	YPDnc1	YPDnc2	YPDnc3	YPDnc4	EtOHnc1	EtOHnc2	Galnc1	Galnc2
13	UGP1	YKL035W	YPDcl2	YPDcl3	EtOHcl1	EtOHcl2	Galcl1	YPDnc1	YPDnc2	YPDnc3	YPDnc4	EtOHnc1	EtOHnc2	Galnc1	Galnc2
13	DOP1	YDR141C	YPDcl2	YPDcl3	EtOHcl1	EtOHcl2	Galcl1	YPDnc1	YPDnc2	YPDnc3	YPDnc4	EtOHnc1	EtOHnc2	Galnc1	Galnc2
13	CFT2	YLR115W	YPDcl2	YPDcl3	EtOHcl1	EtOHcl2	Galcl1	YPDnc1	YPDnc2	YPDnc3	YPDnc4	EtOHnc1	EtOHnc2	Galnc1	Galnc2
13	COQ3	YOL096C	YPDcl2	YPDcl3	EtOHcl1	EtOHcl2	Galcl1	YPDnc1	YPDnc2	YPDnc3	YPDnc4	EtOHnc1	EtOHnc2	Galnc1	Galnc2
13	MGA1	YGR249W	YPDcl2	YPDcl3	EtOHcl1	EtOHcl2	Galcl1	YPDnc1	YPDnc2	YPDnc3	YPDnc4	EtOHnc1	EtOHnc2	Galnc1	Galnc2
13	YPP1	YGR198W	YPDcl2	YPDcl3	EtOHcl1	EtOHcl2	Galcl1	YPDnc1	YPDnc2	YPDnc3	YPDnc4	EtOHnc1	EtOHnc2	Galnc1	Galnc2
13	SPT7	YBR081C	YPDcl2	YPDcl3	EtOHcl1	EtOHcl2	Galcl1	YPDnc1	YPDnc2	YPDnc3	YPDnc4	EtOHnc1	EtOHnc2	Galnc1	Galnc2
13	MRPL50	YNR022C	YPDcl2	YPDcl3	EtOHcl1	EtOHcl2	Galcl1	YPDnc1	YPDnc2	YPDnc3	YPDnc4	EtOHnc1	EtOHnc2	Galnc1	Galnc2
13	LYS14	YDR034C	YPDcl2	YPDcl3	EtOHcl1	EtOHcl2	Galcl1	YPDnc1	YPDnc2	YPDnc3	YPDnc4	EtOHnc1	EtOHnc2	Galnc1	Galnc2
13		YOR352W	YPDcl2	YPDcl3	EtOHcl1	EtOHcl2	Galcl1	YPDnc1	YPDnc2	YPDnc3	YPDnc4	EtOHnc1	EtOHnc2	Galnc1	Galnc2
13	PRP9	YDL030W	YPDcl2	YPDcl3	EtOHcl1	EtOHcl2	Galcl1	YPDnc1	YPDnc2	YPDnc3	YPDnc4	EtOHnc1	EtOHnc2	Galnc1	Galnc2
13	DEP1	YAL013W	YPDcl2	YPDcl3	EtOHcl1	EtOHcl2	Galcl1	YPDnc1	YPDnc2	YPDnc3	YPDnc4	EtOHnc1	EtOHnc2	Galnc1	Galnc2
13		YLR294C	YPDcl2	YPDcl3	EtOHcl1	EtOHcl2	Galcl1	YPDnc1	YPDnc2	YPDnc3	YPDnc4	EtOHnc1	EtOHnc2	Galnc1	Galnc2
13	BET4	YJL031C	YPDcl2	YPDcl3	EtOHcl1	EtOHcl2	Galcl1	YPDnc1	YPDnc2	YPDnc3	YPDnc4	EtOHnc1	EtOHnc2	Galnc1	Galnc2
13	HBS1	YKR084C	YPDcl2	YPDcl3	EtOHcl1	EtOHcl2	Galcl1	YPDnc1	YPDnc2	YPDnc3	YPDnc4	EtOHnc1	EtOHnc2	Galnc1	Galnc2
13	QRI7	YDL104C	YPDcl2	YPDcl3	EtOHcl1	EtOHcl2	Galcl1	YPDnc1	YPDnc2	YPDnc3	YPDnc4	EtOHnc1	EtOHnc2	Galnc1	Galnc2
13		YGR174W-.	YPDcl2	YPDcl3	EtOHcl1	EtOHcl2	Galcl1	YPDnc1	YPDnc2	YPDnc3	YPDnc4	EtOHnc1	EtOHnc2	Galnc1	Galnc2
13		YBL008W-A	YPDcl2	YPDcl3	EtOHcl1	EtOHcl2	Galcl1	YPDnc1	YPDnc2	YPDnc3	YPDnc4	EtOHnc1	EtOHnc2	Galnc1	Galnc2
13	RER2	YBR002C	YPDcl2	YPDcl3	EtOHcl1	EtOHcl2	Galcl1	YPDnc1	YPDnc2	YPDnc3	YPDnc4	EtOHnc1	EtOHnc2	Galnc1	Galnc2
13		YDR119W-.	YPDcl2	YPDcl3	EtOHcl1	EtOHcl2	Galcl1	YPDnc1	YPDnc2	YPDnc3	YPDnc4	EtOHnc1	EtOHnc2	Galnc1	Galnc2
13	LSM12	YHR121W	YPDcl2	YPDcl3	EtOHcl1	EtOHcl2	Galcl1	YPDnc1	YPDnc2	YPDnc3	YPDnc4	EtOHnc1	EtOHnc2	Galnc1	Galnc2
13	GTT3	YEL017W	YPDcl2	YPDcl3	EtOHcl1	EtOHcl2	Galcl1	YPDnc1	YPDnc2	YPDnc3	YPDnc4	EtOHnc1	EtOHnc2	Galnc1	Galnc2
13	COX9	YDL067C	YPDcl2	YPDcl3	EtOHcl1	EtOHcl2	Galcl1	YPDnc1	YPDnc2	YPDnc3	YPDnc4	EtOHnc1	EtOHnc2	Galnc1	Galnc2
13	ACB1	YGR037C	YPDcl2	YPDcl3	EtOHcl1	EtOHcl2	Galcl1	YPDnc1	YPDnc2	YPDnc3	YPDnc4	EtOHnc1	EtOHnc2	Galnc1	Galnc2

13	SCY1	YGL083W	YPDcl2	YPDcl3	EtOHcl1	EtOHcl2	Galcl1	YPDnc1	YPDnc2	YPDnc3	YPDnc4	EtOHnc1	EtOHnc2	Galnc1	Galnc2
13	GFD2	YCL036W	YPDcl2	YPDcl3	EtOHcl1	EtOHcl2	Galcl1	YPDnc1	YPDnc2	YPDnc3	YPDnc4	EtOHnc1	EtOHnc2	Galnc1	Galnc2
13	ELC1	YPL046C	YPDcl2	YPDcl3	EtOHcl1	EtOHcl2	Galcl1	YPDnc1	YPDnc2	YPDnc3	YPDnc4	EtOHnc1	EtOHnc2	Galnc1	Galnc2
13	MAK32	YCR019W	YPDcl2	YPDcl3	EtOHcl1	EtOHcl2	Galcl1	YPDnc1	YPDnc2	YPDnc3	YPDnc4	EtOHnc1	EtOHnc2	Galnc1	Galnc2
13	LAA1	YJL207C	YPDcl2	YPDcl3	EtOHcl1	EtOHcl2	Galcl1	YPDnc1	YPDnc2	YPDnc3	YPDnc4	EtOHnc1	EtOHnc2	Galnc1	Galnc2
13	AST1	YBL069W	YPDcl2	YPDcl3	EtOHcl1	EtOHcl2	Galcl1	YPDnc1	YPDnc2	YPDnc3	YPDnc4	EtOHnc1	EtOHnc2	Galnc1	Galnc2
13	PRT1	YOR361C	YPDcl2	YPDcl3	EtOHcl1	EtOHcl2	Galcl1	YPDnc1	YPDnc2	YPDnc3	YPDnc4	EtOHnc1	EtOHnc2	Galnc1	Galnc2
13	SET3	YKR029C	YPDcl2	YPDcl3	EtOHcl1	EtOHcl2	Galcl1	YPDnc1	YPDnc2	YPDnc3	YPDnc4	EtOHnc1	EtOHnc2	Galnc1	Galnc2
13	NDC1	YML031W	YPDcl2	YPDcl3	EtOHcl1	EtOHcl2	Galcl1	YPDnc1	YPDnc2	YPDnc3	YPDnc4	EtOHnc1	EtOHnc2	Galnc1	Galnc2
13		YNR014W	YPDcl2	YPDcl3	EtOHcl1	EtOHcl2	Galcl1	YPDnc1	YPDnc2	YPDnc3	YPDnc4	EtOHnc1	EtOHnc2	Galnc1	Galnc2
13	POL12	YBL035C	YPDcl2	YPDcl3	EtOHcl1	EtOHcl2	Galcl1	YPDnc1	YPDnc2	YPDnc3	YPDnc4	EtOHnc1	EtOHnc2	Galnc1	Galnc2
13	TRP5	YGL026C	YPDcl2	YPDcl3	EtOHcl1	EtOHcl2	Galcl1	YPDnc1	YPDnc2	YPDnc3	YPDnc4	EtOHnc1	EtOHnc2	Galnc1	Galnc2
13	SAP1	YER047C	YPDcl2	YPDcl3	EtOHcl1	EtOHcl2	Galcl1	YPDnc1	YPDnc2	YPDnc3	YPDnc4	EtOHnc1	EtOHnc2	Galnc1	Galnc2
13	RML2	YEL050C	YPDcl2	YPDcl3	EtOHcl1	EtOHcl2	Galcl1	YPDnc1	YPDnc2	YPDnc3	YPDnc4	EtOHnc1	EtOHnc2	Galnc1	Galnc2
13		YKL136W	YPDcl2	YPDcl3	EtOHcl1	EtOHcl2	Galcl1	YPDnc1	YPDnc2	YPDnc3	YPDnc4	EtOHnc1	EtOHnc2	Galnc1	Galnc2
13	RSM25	YIL093C	YPDcl2	YPDcl3	EtOHcl1	EtOHcl2	Galcl1	YPDnc1	YPDnc2	YPDnc3	YPDnc4	EtOHnc1	EtOHnc2	Galnc1	Galnc2
13	LUC7	YDL087C	YPDcl2	YPDcl3	EtOHcl1	EtOHcl2	Galcl1	YPDnc1	YPDnc2	YPDnc3	YPDnc4	EtOHnc1	EtOHnc2	Galnc1	Galnc2
13	SUP45	YBR143C	YPDcl2	YPDcl3	EtOHcl1	EtOHcl2	Galcl1	YPDnc1	YPDnc2	YPDnc3	YPDnc4	EtOHnc1	EtOHnc2	Galnc1	Galnc2
13		YDR374W-/	YPDcl2	YPDcl3	EtOHcl1	EtOHcl2	Galcl1	YPDnc1	YPDnc2	YPDnc3	YPDnc4	EtOHnc1	EtOHnc2	Galnc1	Galnc2
13		YNR068C	YPDcl2	YPDcl3	EtOHcl1	EtOHcl2	Galcl1	YPDnc1	YPDnc2	YPDnc3	YPDnc4	EtOHnc1	EtOHnc2	Galnc1	Galnc2
13	CCT5	YJR064W	YPDcl2	YPDcl3	EtOHcl1	EtOHcl2	Galcl1	YPDnc1	YPDnc2	YPDnc3	YPDnc4	EtOHnc1	EtOHnc2	Galnc1	Galnc2
13		YGR035W-/	YPDcl2	YPDcl3	EtOHcl1	EtOHcl2	Galcl1	YPDnc1	YPDnc2	YPDnc3	YPDnc4	EtOHnc1	EtOHnc2	Galnc1	Galnc2
13	CAF20	YOR276W	YPDcl2	YPDcl3	EtOHcl1	EtOHcl2	Galcl1	YPDnc1	YPDnc2	YPDnc3	YPDnc4	EtOHnc1	EtOHnc2	Galnc1	Galnc2
13	APL6	YGR261C	YPDcl2	YPDcl3	EtOHcl1	EtOHcl2	Galcl1	YPDnc1	YPDnc2	YPDnc3	YPDnc4	EtOHnc1	EtOHnc2	Galnc1	Galnc2
13	SFT2	YBL102W	YPDcl2	YPDcl3	EtOHcl1	EtOHcl2	Galcl1	YPDnc1	YPDnc2	YPDnc3	YPDnc4	EtOHnc1	EtOHnc2	Galnc1	Galnc2
13	SQT1	YIR012W	YPDcl2	YPDcl3	EtOHcl1	EtOHcl2	Galcl1	YPDnc1	YPDnc2	YPDnc3	YPDnc4	EtOHnc1	EtOHnc2	Galnc1	Galnc2
13	SAC6	YDR129C	YPDcl2	YPDcl3	EtOHcl1	EtOHcl2	Galcl1	YPDnc1	YPDnc2	YPDnc3	YPDnc4	EtOHnc1	EtOHnc2	Galnc1	Galnc2
13	ERG3	YLR056W	YPDcl2	YPDcl3	EtOHcl1	EtOHcl2	Galcl1	YPDnc1	YPDnc2	YPDnc3	YPDnc4	EtOHnc1	EtOHnc2	Galnc1	Galnc2
13	GET2	YER083C	YPDcl2	YPDcl3	EtOHcl1	EtOHcl2	Galcl1	YPDnc1	YPDnc2	YPDnc3	YPDnc4	EtOHnc1	EtOHnc2	Galnc1	Galnc2
13		YGL132W	YPDcl2	YPDcl3	EtOHcl1	EtOHcl2	Galcl1	YPDnc1	YPDnc2	YPDnc3	YPDnc4	EtOHnc1	EtOHnc2	Galnc1	Galnc2
13	TEL2	YGR099W	YPDcl2	YPDcl3	EtOHcl1	EtOHcl2	Galcl1	YPDnc1	YPDnc2	YPDnc3	YPDnc4	EtOHnc1	EtOHnc2	Galnc1	Galnc2
13	NTA1	YJR062C	YPDcl2	YPDcl3	EtOHcl1	EtOHcl2	Galcl1	YPDnc1	YPDnc2	YPDnc3	YPDnc4	EtOHnc1	EtOHnc2	Galnc1	Galnc2
13	APP1	YNL094W	YPDcl2	YPDcl3	EtOHcl1	EtOHcl2	Galcl1	YPDnc1	YPDnc2	YPDnc3	YPDnc4	EtOHnc1	EtOHnc2	Galnc1	Galnc2
13	HCH1	YNL281W	YPDcl2	YPDcl3	EtOHcl1	EtOHcl2	Galcl1	YPDnc1	YPDnc2	YPDnc3	YPDnc4	EtOHnc1	EtOHnc2	Galnc1	Galnc2

13		YHR022C	YPDcl2	YPDcl3	EtOHcl1	EtOHcl2	Galcl1	YPDnc1	YPDnc2	YPDnc3	YPDnc4	EtOHnc1	EtOHnc2	Galnc1	Galnc2
13		YDR320W-	YPDcl2	YPDcl3	EtOHcl1	EtOHcl2	Galcl1	YPDnc1	YPDnc2	YPDnc3	YPDnc4	EtOHnc1	EtOHnc2	Galnc1	Galnc2
13	PAU5	YFL020C	YPDcl2	YPDcl3	EtOHcl1	EtOHcl2	Galcl1	YPDnc1	YPDnc2	YPDnc3	YPDnc4	EtOHnc1	EtOHnc2	Galnc1	Galnc2
13	QCR10	YHR001W-/	YPDcl2	YPDcl3	EtOHcl1	EtOHcl2	Galcl1	YPDnc1	YPDnc2	YPDnc3	YPDnc4	EtOHnc1	EtOHnc2	Galnc1	Galnc2
13	SNU23	YDL098C	YPDcl2	YPDcl3	EtOHcl1	EtOHcl2	Galcl1	YPDnc1	YPDnc2	YPDnc3	YPDnc4	EtOHnc1	EtOHnc2	Galnc1	Galnc2
13	NMA2	YGR010W	YPDcl2	YPDcl3	EtOHcl1	EtOHcl2	Galcl1	YPDnc1	YPDnc2	YPDnc3	YPDnc4	EtOHnc1	EtOHnc2	Galnc1	Galnc2
13		YGL024W	YPDcl2	YPDcl3	EtOHcl1	EtOHcl2	Galcl1	YPDnc1	YPDnc2	YPDnc3	YPDnc4	EtOHnc1	EtOHnc2	Galnc1	Galnc2
13	PDR5	YOR153W	YPDcl2	YPDcl3	EtOHcl1	EtOHcl2	Galcl1	YPDnc1	YPDnc2	YPDnc3	YPDnc4	EtOHnc1	EtOHnc2	Galnc1	Galnc2
13	MUM2	YBR057C	YPDcl2	YPDcl3	EtOHcl1	EtOHcl2	Galcl1	YPDnc1	YPDnc2	YPDnc3	YPDnc4	EtOHnc1	EtOHnc2	Galnc1	Galnc2
13	SVL3	YPL032C	YPDcl2	YPDcl3	EtOHcl1	EtOHcl2	Galcl1	YPDnc1	YPDnc2	YPDnc3	YPDnc4	EtOHnc1	EtOHnc2	Galnc1	Galnc2
13	SMF3	YLR034C	YPDcl2	YPDcl3	EtOHcl1	EtOHcl2	Galcl1	YPDnc1	YPDnc2	YPDnc3	YPDnc4	EtOHnc1	EtOHnc2	Galnc1	Galnc2
13		YOR277C	YPDcl2	YPDcl3	EtOHcl1	EtOHcl2	Galcl1	YPDnc1	YPDnc2	YPDnc3	YPDnc4	EtOHnc1	EtOHnc2	Galnc1	Galnc2
13	CHO2	YGR157W	YPDcl2	YPDcl3	EtOHcl1	EtOHcl2	Galcl1	YPDnc1	YPDnc2	YPDnc3	YPDnc4	EtOHnc1	EtOHnc2	Galnc1	Galnc2
13		YCL047C	YPDcl2	YPDcl3	EtOHcl1	EtOHcl2	Galcl1	YPDnc1	YPDnc2	YPDnc3	YPDnc4	EtOHnc1	EtOHnc2	Galnc1	Galnc2
13		YNL213C	YPDcl2	YPDcl3	EtOHcl1	EtOHcl2	Galcl1	YPDnc1	YPDnc2	YPDnc3	YPDnc4	EtOHnc1	EtOHnc2	Galnc1	Galnc2
13	RPL19B	YBL027W	YPDcl2	YPDcl3	EtOHcl1	EtOHcl2	Galcl1	YPDnc1	YPDnc2	YPDnc3	YPDnc4	EtOHnc1	EtOHnc2	Galnc1	Galnc2
13		YMR018W	YPDcl2	YPDcl3	EtOHcl1	EtOHcl2	Galcl1	YPDnc1	YPDnc2	YPDnc3	YPDnc4	EtOHnc1	EtOHnc2	Galnc1	Galnc2
13	KEL2	YGR238C	YPDcl2	YPDcl3	EtOHcl1	EtOHcl2	Galcl1	YPDnc1	YPDnc2	YPDnc3	YPDnc4	EtOHnc1	EtOHnc2	Galnc1	Galnc2
13	SHE2	YKL130C	YPDcl2	YPDcl3	EtOHcl1	EtOHcl2	Galcl1	YPDnc1	YPDnc2	YPDnc3	YPDnc4	EtOHnc1	EtOHnc2	Galnc1	Galnc2
13	BIO3	YNR058W	YPDcl2	YPDcl3	EtOHcl1	EtOHcl2	Galcl1	YPDnc1	YPDnc2	YPDnc3	YPDnc4	EtOHnc1	EtOHnc2	Galnc1	Galnc2
13	NUP188	YML103C	YPDcl2	YPDcl3	EtOHcl1	EtOHcl2	Galcl1	YPDnc1	YPDnc2	YPDnc3	YPDnc4	EtOHnc1	EtOHnc2	Galnc1	Galnc2
13		YOR305W	YPDcl2	YPDcl3	EtOHcl1	EtOHcl2	Galcl1	YPDnc1	YPDnc2	YPDnc3	YPDnc4	EtOHnc1	EtOHnc2	Galnc1	Galnc2
13	TAF14	YPL129W	YPDcl2	YPDcl3	EtOHcl1	EtOHcl2	Galcl1	YPDnc1	YPDnc2	YPDnc3	YPDnc4	EtOHnc1	EtOHnc2	Galnc1	Galnc2
13	REX2	YLR059C	YPDcl2	YPDcl3	EtOHcl1	EtOHcl2	Galcl1	YPDnc1	YPDnc2	YPDnc3	YPDnc4	EtOHnc1	EtOHnc2	Galnc1	Galnc2
13	LAS1	YKR063C	YPDcl2	YPDcl3	EtOHcl1	EtOHcl2	Galcl1	YPDnc1	YPDnc2	YPDnc3	YPDnc4	EtOHnc1	EtOHnc2	Galnc1	Galnc2
13		YPR003C	YPDcl2	YPDcl3	EtOHcl1	EtOHcl2	Galcl1	YPDnc1	YPDnc2	YPDnc3	YPDnc4	EtOHnc1	EtOHnc2	Galnc1	Galnc2
13		YDR461C-A	YPDcl2	YPDcl3	EtOHcl1	EtOHcl2	Galcl1	YPDnc1	YPDnc2	YPDnc3	YPDnc4	EtOHnc1	EtOHnc2	Galnc1	Galnc2
13		YIL082W	YPDcl2	YPDcl3	EtOHcl1	EtOHcl2	Galcl1	YPDnc1	YPDnc2	YPDnc3	YPDnc4	EtOHnc1	EtOHnc2	Galnc1	Galnc2
13	ASC1	YMR116C	YPDcl2	YPDcl3	EtOHcl1	EtOHcl2	Galcl1	YPDnc1	YPDnc2	YPDnc3	YPDnc4	EtOHnc1	EtOHnc2	Galnc1	Galnc2
13	CWP1	YKL096W	YPDcl2	YPDcl3	EtOHcl1	EtOHcl2	Galcl1	YPDnc1	YPDnc2	YPDnc3	YPDnc4	EtOHnc1	EtOHnc2	Galnc1	Galnc2
13		YKL091C	YPDcl2	YPDcl3	EtOHcl1	EtOHcl2	Galcl1	YPDnc1	YPDnc2	YPDnc3	YPDnc4	EtOHnc1	EtOHnc2	Galnc1	Galnc2
13	SPB1	YCL054W	YPDcl2	YPDcl3	EtOHcl1	EtOHcl2	Galcl1	YPDnc1	YPDnc2	YPDnc3	YPDnc4	EtOHnc1	EtOHnc2	Galnc1	Galnc2
13	FMS1	YMR020W	YPDcl2	YPDcl3	EtOHcl1	EtOHcl2	Galcl1	YPDnc1	YPDnc2	YPDnc3	YPDnc4	EtOHnc1	EtOHnc2	Galnc1	Galnc2
13	DIB1	YPR082C	YPDcl2	YPDcl3	EtOHcl1	EtOHcl2	Galcl1	YPDnc1	YPDnc2	YPDnc3	YPDnc4	EtOHnc1	EtOHnc2	Galnc1	Galnc2

13	STD1	YOR047C	YPDcl2	YPDcl3	EtOHcl1	EtOHcl2	Galcl1	YPDnc1	YPDnc2	YPDnc3	YPDnc4	EtOHnc1	EtOHnc2	Galnc1	Galnc2
13	INO2	YDR123C	YPDcl2	YPDcl3	EtOHcl1	EtOHcl2	Galcl1	YPDnc1	YPDnc2	YPDnc3	YPDnc4	EtOHnc1	EtOHnc2	Galnc1	Galnc2
13		YBR196C-B	YPDcl2	YPDcl3	EtOHcl1	EtOHcl2	Galcl1	YPDnc1	YPDnc2	YPDnc3	YPDnc4	EtOHnc1	EtOHnc2	Galnc1	Galnc2
13	ORC5	YNL261W	YPDcl2	YPDcl3	EtOHcl1	EtOHcl2	Galcl1	YPDnc1	YPDnc2	YPDnc3	YPDnc4	EtOHnc1	EtOHnc2	Galnc1	Galnc2
13		YML003W	YPDcl2	YPDcl3	EtOHcl1	EtOHcl2	Galcl1	YPDnc1	YPDnc2	YPDnc3	YPDnc4	EtOHnc1	EtOHnc2	Galnc1	Galnc2
13	TID3	YIL144W	YPDcl2	YPDcl3	EtOHcl1	EtOHcl2	Galcl1	YPDnc1	YPDnc2	YPDnc3	YPDnc4	EtOHnc1	EtOHnc2	Galnc1	Galnc2
13	VPS21	YOR089C	YPDcl2	YPDcl3	EtOHcl1	EtOHcl2	Galcl1	YPDnc1	YPDnc2	YPDnc3	YPDnc4	EtOHnc1	EtOHnc2	Galnc1	Galnc2
13	ARP7	YPR034W	YPDcl2	YPDcl3	EtOHcl1	EtOHcl2	Galcl1	YPDnc1	YPDnc2	YPDnc3	YPDnc4	EtOHnc1	EtOHnc2	Galnc1	Galnc2
13	RPL40A	YIL148W	YPDcl2	YPDcl3	EtOHcl1	EtOHcl2	Galcl1	YPDnc1	YPDnc2	YPDnc3	YPDnc4	EtOHnc1	EtOHnc2	Galnc1	Galnc2
13		YMR316C-[illegible]	YPDcl2	YPDcl3	EtOHcl1	EtOHcl2	Galcl1	YPDnc1	YPDnc2	YPDnc3	YPDnc4	EtOHnc1	EtOHnc2	Galnc1	Galnc2
13		YGR122C-A	YPDcl2	YPDcl3	EtOHcl1	EtOHcl2	Galcl1	YPDnc1	YPDnc2	YPDnc3	YPDnc4	EtOHnc1	EtOHnc2	Galnc1	Galnc2
13	ARL1	YBR164C	YPDcl2	YPDcl3	EtOHcl1	EtOHcl2	Galcl1	YPDnc1	YPDnc2	YPDnc3	YPDnc4	EtOHnc1	EtOHnc2	Galnc1	Galnc2
13	SAM37	YMR060C	YPDcl2	YPDcl3	EtOHcl1	EtOHcl2	Galcl1	YPDnc1	YPDnc2	YPDnc3	YPDnc4	EtOHnc1	EtOHnc2	Galnc1	Galnc2
13	SEC28	YIL076W	YPDcl2	YPDcl3	EtOHcl1	EtOHcl2	Galcl1	YPDnc1	YPDnc2	YPDnc3	YPDnc4	EtOHnc1	EtOHnc2	Galnc1	Galnc2
13	GAC1	YOR178C	YPDcl2	YPDcl3	EtOHcl1	EtOHcl2	Galcl1	YPDnc1	YPDnc2	YPDnc3	YPDnc4	EtOHnc1	EtOHnc2	Galnc1	Galnc2
13		YBR109W-A	YPDcl2	YPDcl3	EtOHcl1	EtOHcl2	Galcl1	YPDnc1	YPDnc2	YPDnc3	YPDnc4	EtOHnc1	EtOHnc2	Galnc1	Galnc2
13	MTD1	YKR080W	YPDcl2	YPDcl3	EtOHcl1	EtOHcl2	Galcl1	YPDnc1	YPDnc2	YPDnc3	YPDnc4	EtOHnc1	EtOHnc2	Galnc1	Galnc2
13	RTT102	YGR275W	YPDcl2	YPDcl3	EtOHcl1	EtOHcl2	Galcl1	YPDnc1	YPDnc2	YPDnc3	YPDnc4	EtOHnc1	EtOHnc2	Galnc1	Galnc2
13	SLI1	YGR212W	YPDcl2	YPDcl3	EtOHcl1	EtOHcl2	Galcl1	YPDnc1	YPDnc2	YPDnc3	YPDnc4	EtOHnc1	EtOHnc2	Galnc1	Galnc2
13		YJR115W	YPDcl2	YPDcl3	EtOHcl1	EtOHcl2	Galcl1	YPDnc1	YPDnc2	YPDnc3	YPDnc4	EtOHnc1	EtOHnc2	Galnc1	Galnc2
13		YPL225W	YPDcl2	YPDcl3	EtOHcl1	EtOHcl2	Galcl1	YPDnc1	YPDnc2	YPDnc3	YPDnc4	EtOHnc1	EtOHnc2	Galnc1	Galnc2
13	ICY1	YMR195W	YPDcl2	YPDcl3	EtOHcl1	EtOHcl2	Galcl1	YPDnc1	YPDnc2	YPDnc3	YPDnc4	EtOHnc1	EtOHnc2	Galnc1	Galnc2
13	MSS18	YPR134W	YPDcl2	YPDcl3	EtOHcl1	EtOHcl2	Galcl1	YPDnc1	YPDnc2	YPDnc3	YPDnc4	EtOHnc1	EtOHnc2	Galnc1	Galnc2
13	CDC34	YDR054C	YPDcl2	YPDcl3	EtOHcl1	EtOHcl2	Galcl1	YPDnc1	YPDnc2	YPDnc3	YPDnc4	EtOHnc1	EtOHnc2	Galnc1	Galnc2
13		YDL119C	YPDcl2	YPDcl3	EtOHcl1	EtOHcl2	Galcl1	YPDnc1	YPDnc2	YPDnc3	YPDnc4	EtOHnc1	EtOHnc2	Galnc1	Galnc2
13	SMC1	YFL008W	YPDcl2	YPDcl3	EtOHcl1	EtOHcl2	Galcl1	YPDnc1	YPDnc2	YPDnc3	YPDnc4	EtOHnc1	EtOHnc2	Galnc1	Galnc2
13	PEX31	YGR004W	YPDcl2	YPDcl3	EtOHcl1	EtOHcl2	Galcl1	YPDnc1	YPDnc2	YPDnc3	YPDnc4	EtOHnc1	EtOHnc2	Galnc1	Galnc2
13	ATM1	YMR301C	YPDcl2	YPDcl3	EtOHcl1	EtOHcl2	Galcl1	YPDnc1	YPDnc2	YPDnc3	YPDnc4	EtOHnc1	EtOHnc2	Galnc1	Galnc2
13		YIL046W-A	YPDcl2	YPDcl3	EtOHcl1	EtOHcl2	Galcl1	YPDnc1	YPDnc2	YPDnc3	YPDnc4	EtOHnc1	EtOHnc2	Galnc1	Galnc2
13		YPR116W	YPDcl2	YPDcl3	EtOHcl1	EtOHcl2	Galcl1	YPDnc1	YPDnc2	YPDnc3	YPDnc4	EtOHnc1	EtOHnc2	Galnc1	Galnc2
13	ITR2	YOL103W	YPDcl2	YPDcl3	EtOHcl1	EtOHcl2	Galcl1	YPDnc1	YPDnc2	YPDnc3	YPDnc4	EtOHnc1	EtOHnc2	Galnc1	Galnc2
13		YDR199W	YPDcl2	YPDcl3	EtOHcl1	EtOHcl2	Galcl1	YPDnc1	YPDnc2	YPDnc3	YPDnc4	EtOHnc1	EtOHnc2	Galnc1	Galnc2
13	VAC14	YLR386W	YPDcl2	YPDcl3	EtOHcl1	EtOHcl2	Galcl1	YPDnc1	YPDnc2	YPDnc3	YPDnc4	EtOHnc1	EtOHnc2	Galnc1	Galnc2
13		YNL143C	YPDcl2	YPDcl3	EtOHcl1	EtOHcl2	Galcl1	YPDnc1	YPDnc2	YPDnc3	YPDnc4	EtOHnc1	EtOHnc2	Galnc1	Galnc2

13	SCO1	YBR037C	YPDcl2	YPDcl3	EtOHcl1	EtOHcl2	Galcl1	YPDnc1	YPDnc2	YPDnc3	YPDnc4	EtOHnc1	EtOHnc2	Galnc1	Galnc2
13		YER181C	YPDcl2	YPDcl3	EtOHcl1	EtOHcl2	Galcl1	YPDnc1	YPDnc2	YPDnc3	YPDnc4	EtOHnc1	EtOHnc2	Galnc1	Galnc2
13		YFR045W	YPDcl2	YPDcl3	EtOHcl1	EtOHcl2	Galcl1	YPDnc1	YPDnc2	YPDnc3	YPDnc4	EtOHnc1	EtOHnc2	Galnc1	Galnc2
13	ASN1	YPR145W	YPDcl2	YPDcl3	EtOHcl1	EtOHcl2	Galcl1	YPDnc1	YPDnc2	YPDnc3	YPDnc4	EtOHnc1	EtOHnc2	Galnc1	Galnc2
13	PKR1	YMR123W	YPDcl2	YPDcl3	EtOHcl1	EtOHcl2	Galcl1	YPDnc1	YPDnc2	YPDnc3	YPDnc4	EtOHnc1	EtOHnc2	Galnc1	Galnc2
13	CPA1	YOR303W	YPDcl2	YPDcl3	EtOHcl1	EtOHcl2	Galcl1	YPDnc1	YPDnc2	YPDnc3	YPDnc4	EtOHnc1	EtOHnc2	Galnc1	Galnc2
13	TRZ1	YKR079C	YPDcl2	YPDcl3	EtOHcl1	EtOHcl2	Galcl1	YPDnc1	YPDnc2	YPDnc3	YPDnc4	EtOHnc1	EtOHnc2	Galnc1	Galnc2
13	ORC3	YLL004W	YPDcl2	YPDcl3	EtOHcl1	EtOHcl2	Galcl1	YPDnc1	YPDnc2	YPDnc3	YPDnc4	EtOHnc1	EtOHnc2	Galnc1	Galnc2
13	RSA3	YLR221C	YPDcl2	YPDcl3	EtOHcl1	EtOHcl2	Galcl1	YPDnc1	YPDnc2	YPDnc3	YPDnc4	EtOHnc1	EtOHnc2	Galnc1	Galnc2
13	SRL4	YPL033C	YPDcl2	YPDcl3	EtOHcl1	EtOHcl2	Galcl1	YPDnc1	YPDnc2	YPDnc3	YPDnc4	EtOHnc1	EtOHnc2	Galnc1	Galnc2
13	INN1	YNL152W	YPDcl2	YPDcl3	EtOHcl1	EtOHcl2	Galcl1	YPDnc1	YPDnc2	YPDnc3	YPDnc4	EtOHnc1	EtOHnc2	Galnc1	Galnc2
13	SUI1	YNL244C	YPDcl2	YPDcl3	EtOHcl1	EtOHcl2	Galcl1	YPDnc1	YPDnc2	YPDnc3	YPDnc4	EtOHnc1	EtOHnc2	Galnc1	Galnc2
13	CCT7	YJL111W	YPDcl2	YPDcl3	EtOHcl1	EtOHcl2	Galcl1	YPDnc1	YPDnc2	YPDnc3	YPDnc4	EtOHnc1	EtOHnc2	Galnc1	Galnc2
13	FPR1	YNL135C	YPDcl2	YPDcl3	EtOHcl1	EtOHcl2	Galcl1	YPDnc1	YPDnc2	YPDnc3	YPDnc4	EtOHnc1	EtOHnc2	Galnc1	Galnc2
13	RPS20	YHL015W	YPDcl2	YPDcl3	EtOHcl1	EtOHcl2	Galcl1	YPDnc1	YPDnc2	YPDnc3	YPDnc4	EtOHnc1	EtOHnc2	Galnc1	Galnc2
13	IMP2'	YIL154C	YPDcl2	YPDcl3	EtOHcl1	EtOHcl2	Galcl1	YPDnc1	YPDnc2	YPDnc3	YPDnc4	EtOHnc1	EtOHnc2	Galnc1	Galnc2
13	ALY2	YJL084C	YPDcl2	YPDcl3	EtOHcl1	EtOHcl2	Galcl1	YPDnc1	YPDnc2	YPDnc3	YPDnc4	EtOHnc1	EtOHnc2	Galnc1	Galnc2
13	VAN1	YML115C	YPDcl2	YPDcl3	EtOHcl1	EtOHcl2	Galcl1	YPDnc1	YPDnc2	YPDnc3	YPDnc4	EtOHnc1	EtOHnc2	Galnc1	Galnc2
13	DPB3	YBR278W	YPDcl2	YPDcl3	EtOHcl1	EtOHcl2	Galcl1	YPDnc1	YPDnc2	YPDnc3	YPDnc4	EtOHnc1	EtOHnc2	Galnc1	Galnc2
13		YJL150W	YPDcl2	YPDcl3	EtOHcl1	EtOHcl2	Galcl1	YPDnc1	YPDnc2	YPDnc3	YPDnc4	EtOHnc1	EtOHnc2	Galnc1	Galnc2
13	FMN1	YDR236C	YPDcl2	YPDcl3	EtOHcl1	EtOHcl2	Galcl1	YPDnc1	YPDnc2	YPDnc3	YPDnc4	EtOHnc1	EtOHnc2	Galnc1	Galnc2
13	GDB1	YPR184W	YPDcl2	YPDcl3	EtOHcl1	EtOHcl2	Galcl1	YPDnc1	YPDnc2	YPDnc3	YPDnc4	EtOHnc1	EtOHnc2	Galnc1	Galnc2
13		YDR371C-A	YPDcl2	YPDcl3	EtOHcl1	EtOHcl2	Galcl1	YPDnc1	YPDnc2	YPDnc3	YPDnc4	EtOHnc1	EtOHnc2	Galnc1	Galnc2
13	RPL35A	YDL191W	YPDcl2	YPDcl3	EtOHcl1	EtOHcl2	Galcl1	YPDnc1	YPDnc2	YPDnc3	YPDnc4	EtOHnc1	EtOHnc2	Galnc1	Galnc2
13		YPR145C-A	YPDcl2	YPDcl3	EtOHcl1	EtOHcl2	Galcl1	YPDnc1	YPDnc2	YPDnc3	YPDnc4	EtOHnc1	EtOHnc2	Galnc1	Galnc2
13		YBR144C	YPDcl2	YPDcl3	EtOHcl1	EtOHcl2	Galcl1	YPDnc1	YPDnc2	YPDnc3	YPDnc4	EtOHnc1	EtOHnc2	Galnc1	Galnc2
13	RSC8	YFR037C	YPDcl2	YPDcl3	EtOHcl1	EtOHcl2	Galcl1	YPDnc1	YPDnc2	YPDnc3	YPDnc4	EtOHnc1	EtOHnc2	Galnc1	Galnc2
13	OST1	YJL002C	YPDcl2	YPDcl3	EtOHcl1	EtOHcl2	Galcl1	YPDnc1	YPDnc2	YPDnc3	YPDnc4	EtOHnc1	EtOHnc2	Galnc1	Galnc2
13	DUR3	YHL016C	YPDcl2	YPDcl3	EtOHcl1	EtOHcl2	Galcl1	YPDnc1	YPDnc2	YPDnc3	YPDnc4	EtOHnc1	EtOHnc2	Galnc1	Galnc2
13	SLX4	YLR135W	YPDcl2	YPDcl3	EtOHcl1	EtOHcl2	Galcl1	YPDnc1	YPDnc2	YPDnc3	YPDnc4	EtOHnc1	EtOHnc2	Galnc1	Galnc2
13	MPD2	YOL088C	YPDcl2	YPDcl3	EtOHcl1	EtOHcl2	Galcl1	YPDnc1	YPDnc2	YPDnc3	YPDnc4	EtOHnc1	EtOHnc2	Galnc1	Galnc2
13	ELP3	YPL086C	YPDcl2	YPDcl3	EtOHcl1	EtOHcl2	Galcl1	YPDnc1	YPDnc2	YPDnc3	YPDnc4	EtOHnc1	EtOHnc2	Galnc1	Galnc2
13		YBR027C	YPDcl2	YPDcl3	EtOHcl1	EtOHcl2	Galcl1	YPDnc1	YPDnc2	YPDnc3	YPDnc4	EtOHnc1	EtOHnc2	Galnc1	Galnc2
13	CHS3	YBR023C	YPDcl2	YPDcl3	EtOHcl1	EtOHcl2	Galcl1	YPDnc1	YPDnc2	YPDnc3	YPDnc4	EtOHnc1	EtOHnc2	Galnc1	Galnc2

13	MND2	YIR025W	YPDcl2	YPDcl3	EtOHcl1	EtOHcl2	Galcl1	YPDnc1	YPDnc2	YPDnc3	YPDnc4	EtOHnc1	EtOHnc2	Galnc1	Galnc2
13	APL2	YKL135C	YPDcl2	YPDcl3	EtOHcl1	EtOHcl2	Galcl1	YPDnc1	YPDnc2	YPDnc3	YPDnc4	EtOHnc1	EtOHnc2	Galnc1	Galnc2
13	UBP8	YMR223W	YPDcl2	YPDcl3	EtOHcl1	EtOHcl2	Galcl1	YPDnc1	YPDnc2	YPDnc3	YPDnc4	EtOHnc1	EtOHnc2	Galnc1	Galnc2
13		YML009C-A	YPDcl2	YPDcl3	EtOHcl1	EtOHcl2	Galcl1	YPDnc1	YPDnc2	YPDnc3	YPDnc4	EtOHnc1	EtOHnc2	Galnc1	Galnc2
13	NAT1	YDL040C	YPDcl2	YPDcl3	EtOHcl1	EtOHcl2	Galcl1	YPDnc1	YPDnc2	YPDnc3	YPDnc4	EtOHnc1	EtOHnc2	Galnc1	Galnc2
13		YBL071C-B	YPDcl2	YPDcl3	EtOHcl1	EtOHcl2	Galcl1	YPDnc1	YPDnc2	YPDnc3	YPDnc4	EtOHnc1	EtOHnc2	Galnc1	Galnc2
13	MIF2	YKL089W	YPDcl2	YPDcl3	EtOHcl1	EtOHcl2	Galcl1	YPDnc1	YPDnc2	YPDnc3	YPDnc4	EtOHnc1	EtOHnc2	Galnc1	Galnc2
13	LIP2	YLR239C	YPDcl2	YPDcl3	EtOHcl1	EtOHcl2	Galcl1	YPDnc1	YPDnc2	YPDnc3	YPDnc4	EtOHnc1	EtOHnc2	Galnc1	Galnc2
13	VTC3	YPL019C	YPDcl2	YPDcl3	EtOHcl1	EtOHcl2	Galcl1	YPDnc1	YPDnc2	YPDnc3	YPDnc4	EtOHnc1	EtOHnc2	Galnc1	Galnc2
13		YMR158W-	YPDcl2	YPDcl3	EtOHcl1	EtOHcl2	Galcl1	YPDnc1	YPDnc2	YPDnc3	YPDnc4	EtOHnc1	EtOHnc2	Galnc1	Galnc2
13	APC9	YLR102C	YPDcl2	YPDcl3	EtOHcl1	EtOHcl2	Galcl1	YPDnc1	YPDnc2	YPDnc3	YPDnc4	EtOHnc1	EtOHnc2	Galnc1	Galnc2
13	ARG2	YJL071W	YPDcl2	YPDcl3	EtOHcl1	EtOHcl2	Galcl1	YPDnc1	YPDnc2	YPDnc3	YPDnc4	EtOHnc1	EtOHnc2	Galnc1	Galnc2
13	URA2	YJL130C	YPDcl2	YPDcl3	EtOHcl1	EtOHcl2	Galcl1	YPDnc1	YPDnc2	YPDnc3	YPDnc4	EtOHnc1	EtOHnc2	Galnc1	Galnc2
13		YBR277C	YPDcl2	YPDcl3	EtOHcl1	EtOHcl2	Galcl1	YPDnc1	YPDnc2	YPDnc3	YPDnc4	EtOHnc1	EtOHnc2	Galnc1	Galnc2
13	CRT10	YOL063C	YPDcl2	YPDcl3	EtOHcl1	EtOHcl2	Galcl1	YPDnc1	YPDnc2	YPDnc3	YPDnc4	EtOHnc1	EtOHnc2	Galnc1	Galnc2
13	TIF4632	YGL049C	YPDcl2	YPDcl3	EtOHcl1	EtOHcl2	Galcl1	YPDnc1	YPDnc2	YPDnc3	YPDnc4	EtOHnc1	EtOHnc2	Galnc1	Galnc2
13	YIP1	YGR172C	YPDcl2	YPDcl3	EtOHcl1	EtOHcl2	Galcl1	YPDnc1	YPDnc2	YPDnc3	YPDnc4	EtOHnc1	EtOHnc2	Galnc1	Galnc2
13		YGR111W	YPDcl2	YPDcl3	EtOHcl1	EtOHcl2	Galcl1	YPDnc1	YPDnc2	YPDnc3	YPDnc4	EtOHnc1	EtOHnc2	Galnc1	Galnc2
13	CKB2	YOR039W	YPDcl2	YPDcl3	EtOHcl1	EtOHcl2	Galcl1	YPDnc1	YPDnc2	YPDnc3	YPDnc4	EtOHnc1	EtOHnc2	Galnc1	Galnc2
13	YDC1	YPL087W	YPDcl2	YPDcl3	EtOHcl1	EtOHcl2	Galcl1	YPDnc1	YPDnc2	YPDnc3	YPDnc4	EtOHnc1	EtOHnc2	Galnc1	Galnc2
13	SRT1	YMR101C	YPDcl2	YPDcl3	EtOHcl1	EtOHcl2	Galcl1	YPDnc1	YPDnc2	YPDnc3	YPDnc4	EtOHnc1	EtOHnc2	Galnc1	Galnc2
13	YCF1	YDR135C	YPDcl2	YPDcl3	EtOHcl1	EtOHcl2	Galcl1	YPDnc1	YPDnc2	YPDnc3	YPDnc4	EtOHnc1	EtOHnc2	Galnc1	Galnc2
13	PUT3	YKL015W	YPDcl2	YPDcl3	EtOHcl1	EtOHcl2	Galcl1	YPDnc1	YPDnc2	YPDnc3	YPDnc4	EtOHnc1	EtOHnc2	Galnc1	Galnc2
13	PSF3	YOL146W	YPDcl2	YPDcl3	EtOHcl1	EtOHcl2	Galcl1	YPDnc1	YPDnc2	YPDnc3	YPDnc4	EtOHnc1	EtOHnc2	Galnc1	Galnc2
13	VAS1	YGR094W	YPDcl2	YPDcl3	EtOHcl1	EtOHcl2	Galcl1	YPDnc1	YPDnc2	YPDnc3	YPDnc4	EtOHnc1	EtOHnc2	Galnc1	Galnc2
13	MID2	YLR332W	YPDcl2	YPDcl3	EtOHcl1	EtOHcl2	Galcl1	YPDnc1	YPDnc2	YPDnc3	YPDnc4	EtOHnc1	EtOHnc2	Galnc1	Galnc2
13	SLX9	YGR081C	YPDcl2	YPDcl3	EtOHcl1	EtOHcl2	Galcl1	YPDnc1	YPDnc2	YPDnc3	YPDnc4	EtOHnc1	EtOHnc2	Galnc1	Galnc2
13	TSC3	YBR058C-A	YPDcl2	YPDcl3	EtOHcl1	EtOHcl2	Galcl1	YPDnc1	YPDnc2	YPDnc3	YPDnc4	EtOHnc1	EtOHnc2	Galnc1	Galnc2
13	MTG2	YHR168W	YPDcl2	YPDcl3	EtOHcl1	EtOHcl2	Galcl1	YPDnc1	YPDnc2	YPDnc3	YPDnc4	EtOHnc1	EtOHnc2	Galnc1	Galnc2
13	RPP1	YHR062C	YPDcl2	YPDcl3	EtOHcl1	EtOHcl2	Galcl1	YPDnc1	YPDnc2	YPDnc3	YPDnc4	EtOHnc1	EtOHnc2	Galnc1	Galnc2
13	SOL4	YGR248W	YPDcl2	YPDcl3	EtOHcl1	EtOHcl2	Galcl1	YPDnc1	YPDnc2	YPDnc3	YPDnc4	EtOHnc1	EtOHnc2	Galnc1	Galnc2
13	ARG1	YOL058W	YPDcl2	YPDcl3	EtOHcl1	EtOHcl2	Galcl1	YPDnc1	YPDnc2	YPDnc3	YPDnc4	EtOHnc1	EtOHnc2	Galnc1	Galnc2
13	TUL1	YKL034W	YPDcl2	YPDcl3	EtOHcl1	EtOHcl2	Galcl1	YPDnc1	YPDnc2	YPDnc3	YPDnc4	EtOHnc1	EtOHnc2	Galnc1	Galnc2
13	CIN4	YMR138W	YPDcl2	YPDcl3	EtOHcl1	EtOHcl2	Galcl1	YPDnc1	YPDnc2	YPDnc3	YPDnc4	EtOHnc1	EtOHnc2	Galnc1	Galnc2

13	RAD1	YPL022W	YPDcl2	YPDcl3	EtOHcl1	EtOHcl2	Galcl1	YPDnc1	YPDnc2	YPDnc3	YPDnc4	EtOHnc1	EtOHnc2	Galnc1	Galnc2
13	GZF3	YJL110C	YPDcl2	YPDcl3	EtOHcl1	EtOHcl2	Galcl1	YPDnc1	YPDnc2	YPDnc3	YPDnc4	EtOHnc1	EtOHnc2	Galnc1	Galnc2
13	SWH1	YAR042W	YPDcl2	YPDcl3	EtOHcl1	EtOHcl2	Galcl1	YPDnc1	YPDnc2	YPDnc3	YPDnc4	EtOHnc1	EtOHnc2	Galnc1	Galnc2
13		YFL012W-A	YPDcl2	YPDcl3	EtOHcl1	EtOHcl2	Galcl1	YPDnc1	YPDnc2	YPDnc3	YPDnc4	EtOHnc1	EtOHnc2	Galnc1	Galnc2
13	PRY2	YKR013W	YPDcl2	YPDcl3	EtOHcl1	EtOHcl2	Galcl1	YPDnc1	YPDnc2	YPDnc3	YPDnc4	EtOHnc1	EtOHnc2	Galnc1	Galnc2
13	SMC3	YJL074C	YPDcl2	YPDcl3	EtOHcl1	EtOHcl2	Galcl1	YPDnc1	YPDnc2	YPDnc3	YPDnc4	EtOHnc1	EtOHnc2	Galnc1	Galnc2
13	IKI3	YLR384C	YPDcl2	YPDcl3	EtOHcl1	EtOHcl2	Galcl1	YPDnc1	YPDnc2	YPDnc3	YPDnc4	EtOHnc1	EtOHnc2	Galnc1	Galnc2
13	MLC2	YPR188C	YPDcl2	YPDcl3	EtOHcl1	EtOHcl2	Galcl1	YPDnc1	YPDnc2	YPDnc3	YPDnc4	EtOHnc1	EtOHnc2	Galnc1	Galnc2
13	RGA1	YOR127W	YPDcl2	YPDcl3	EtOHcl1	EtOHcl2	Galcl1	YPDnc1	YPDnc2	YPDnc3	YPDnc4	EtOHnc1	EtOHnc2	Galnc1	Galnc2
13	OAF3	YKR064W	YPDcl2	YPDcl3	EtOHcl1	EtOHcl2	Galcl1	YPDnc1	YPDnc2	YPDnc3	YPDnc4	EtOHnc1	EtOHnc2	Galnc1	Galnc2
13	JAC1	YGL018C	YPDcl2	YPDcl3	EtOHcl1	EtOHcl2	Galcl1	YPDnc1	YPDnc2	YPDnc3	YPDnc4	EtOHnc1	EtOHnc2	Galnc1	Galnc2
13	PHO90	YJL198W	YPDcl2	YPDcl3	EtOHcl1	EtOHcl2	Galcl1	YPDnc1	YPDnc2	YPDnc3	YPDnc4	EtOHnc1	EtOHnc2	Galnc1	Galnc2
13	SYC1	YOR179C	YPDcl2	YPDcl3	EtOHcl1	EtOHcl2	Galcl1	YPDnc1	YPDnc2	YPDnc3	YPDnc4	EtOHnc1	EtOHnc2	Galnc1	Galnc2
13		YBR134W	YPDcl2	YPDcl3	EtOHcl1	EtOHcl2	Galcl1	YPDnc1	YPDnc2	YPDnc3	YPDnc4	EtOHnc1	EtOHnc2	Galnc1	Galnc2
13	SPT6	YGR116W	YPDcl2	YPDcl3	EtOHcl1	EtOHcl2	Galcl1	YPDnc1	YPDnc2	YPDnc3	YPDnc4	EtOHnc1	EtOHnc2	Galnc1	Galnc2
13	TRM44	YPL030W	YPDcl2	YPDcl3	EtOHcl1	EtOHcl2	Galcl1	YPDnc1	YPDnc2	YPDnc3	YPDnc4	EtOHnc1	EtOHnc2	Galnc1	Galnc2
13	ADE5,7	YGL234W	YPDcl2	YPDcl3	EtOHcl1	EtOHcl2	Galcl1	YPDnc1	YPDnc2	YPDnc3	YPDnc4	EtOHnc1	EtOHnc2	Galnc1	Galnc2
13	SWC4	YGR002C	YPDcl2	YPDcl3	EtOHcl1	EtOHcl2	Galcl1	YPDnc1	YPDnc2	YPDnc3	YPDnc4	EtOHnc1	EtOHnc2	Galnc1	Galnc2
13	URA10	YMR271C	YPDcl2	YPDcl3	EtOHcl1	EtOHcl2	Galcl1	YPDnc1	YPDnc2	YPDnc3	YPDnc4	EtOHnc1	EtOHnc2	Galnc1	Galnc2
13	CMD1	YBR109C	YPDcl2	YPDcl3	EtOHcl1	EtOHcl2	Galcl1	YPDnc1	YPDnc2	YPDnc3	YPDnc4	EtOHnc1	EtOHnc2	Galnc1	Galnc2
13	DIG2	YDR480W	YPDcl2	YPDcl3	EtOHcl1	EtOHcl2	Galcl1	YPDnc1	YPDnc2	YPDnc3	YPDnc4	EtOHnc1	EtOHnc2	Galnc1	Galnc2
13	AVT5	YBL089W	YPDcl2	YPDcl3	EtOHcl1	EtOHcl2	Galcl1	YPDnc1	YPDnc2	YPDnc3	YPDnc4	EtOHnc1	EtOHnc2	Galnc1	Galnc2
13	SHY1	YGR112W	YPDcl2	YPDcl3	EtOHcl1	EtOHcl2	Galcl1	YPDnc1	YPDnc2	YPDnc3	YPDnc4	EtOHnc1	EtOHnc2	Galnc1	Galnc2
13	YEH1	YLL012W	YPDcl2	YPDcl3	EtOHcl1	EtOHcl2	Galcl1	YPDnc1	YPDnc2	YPDnc3	YPDnc4	EtOHnc1	EtOHnc2	Galnc1	Galnc2
13		YMR013C-A	YPDcl2	YPDcl3	EtOHcl1	EtOHcl2	Galcl1	YPDnc1	YPDnc2	YPDnc3	YPDnc4	EtOHnc1	EtOHnc2	Galnc1	Galnc2
13	KSP1	YHR082C	YPDcl2	YPDcl3	EtOHcl1	EtOHcl2	Galcl1	YPDnc1	YPDnc2	YPDnc3	YPDnc4	EtOHnc1	EtOHnc2	Galnc1	Galnc2
13	GAT3	YLR013W	YPDcl2	YPDcl3	EtOHcl1	EtOHcl2	Galcl1	YPDnc1	YPDnc2	YPDnc3	YPDnc4	EtOHnc1	EtOHnc2	Galnc1	Galnc2
13	LRS4	YDR439W	YPDcl2	YPDcl3	EtOHcl1	EtOHcl2	Galcl1	YPDnc1	YPDnc2	YPDnc3	YPDnc4	EtOHnc1	EtOHnc2	Galnc1	Galnc2
13	YPS7	YDR349C	YPDcl2	YPDcl3	EtOHcl1	EtOHcl2	Galcl1	YPDnc1	YPDnc2	YPDnc3	YPDnc4	EtOHnc1	EtOHnc2	Galnc1	Galnc2
13		YLR408C	YPDcl2	YPDcl3	EtOHcl1	EtOHcl2	Galcl1	YPDnc1	YPDnc2	YPDnc3	YPDnc4	EtOHnc1	EtOHnc2	Galnc1	Galnc2
13	MEI5	YPL121C	YPDcl2	YPDcl3	EtOHcl1	EtOHcl2	Galcl1	YPDnc1	YPDnc2	YPDnc3	YPDnc4	EtOHnc1	EtOHnc2	Galnc1	Galnc2
13		YDR290W	YPDcl2	YPDcl3	EtOHcl1	EtOHcl2	Galcl1	YPDnc1	YPDnc2	YPDnc3	YPDnc4	EtOHnc1	EtOHnc2	Galnc1	Galnc2
13	LEM3	YNL323W	YPDcl2	YPDcl3	EtOHcl1	EtOHcl2	Galcl1	YPDnc1	YPDnc2	YPDnc3	YPDnc4	EtOHnc1	EtOHnc2	Galnc1	Galnc2
13	ENO1	YGR254W	YPDcl2	YPDcl3	EtOHcl1	EtOHcl2	Galcl1	YPDnc1	YPDnc2	YPDnc3	YPDnc4	EtOHnc1	EtOHnc2	Galnc1	Galnc2

13	PAN6	YIL145C	YPDcl2	YPDcl3	EtOHcl1	EtOHcl2	Galcl1	YPDnc1	YPDnc2	YPDnc3	YPDnc4	EtOHnc1	EtOHnc2	Galnc1	Galnc2
13		YMR209C	YPDcl2	YPDcl3	EtOHcl1	EtOHcl2	Galcl1	YPDnc1	YPDnc2	YPDnc3	YPDnc4	EtOHnc1	EtOHnc2	Galnc1	Galnc2
13	RNY1	YPL123C	YPDcl2	YPDcl3	EtOHcl1	EtOHcl2	Galcl1	YPDnc1	YPDnc2	YPDnc3	YPDnc4	EtOHnc1	EtOHnc2	Galnc1	Galnc2
13	DAS1	YJL149W	YPDcl2	YPDcl3	EtOHcl1	EtOHcl2	Galcl1	YPDnc1	YPDnc2	YPDnc3	YPDnc4	EtOHnc1	EtOHnc2	Galnc1	Galnc2
13	AAD14	YNL331C	YPDcl2	YPDcl3	EtOHcl1	EtOHcl2	Galcl1	YPDnc1	YPDnc2	YPDnc3	YPDnc4	EtOHnc1	EtOHnc2	Galnc1	Galnc2
13	YCG1	YDR325W	YPDcl2	YPDcl3	EtOHcl1	EtOHcl2	Galcl1	YPDnc1	YPDnc2	YPDnc3	YPDnc4	EtOHnc1	EtOHnc2	Galnc1	Galnc2
13	HEM2	YGL040C	YPDcl2	YPDcl3	EtOHcl1	EtOHcl2	Galcl1	YPDnc1	YPDnc2	YPDnc3	YPDnc4	EtOHnc1	EtOHnc2	Galnc1	Galnc2
13	RPT4	YOR259C	YPDcl2	YPDcl3	EtOHcl1	EtOHcl2	Galcl1	YPDnc1	YPDnc2	YPDnc3	YPDnc4	EtOHnc1	EtOHnc2	Galnc1	Galnc2
13		YDL085C-A	YPDcl2	YPDcl3	EtOHcl1	EtOHcl2	Galcl1	YPDnc1	YPDnc2	YPDnc3	YPDnc4	EtOHnc1	EtOHnc2	Galnc1	Galnc2
13	PRM3	YPL192C	YPDcl2	YPDcl3	EtOHcl1	EtOHcl2	Galcl1	YPDnc1	YPDnc2	YPDnc3	YPDnc4	EtOHnc1	EtOHnc2	Galnc1	Galnc2
13		YER084W	YPDcl2	YPDcl3	EtOHcl1	EtOHcl2	Galcl1	YPDnc1	YPDnc2	YPDnc3	YPDnc4	EtOHnc1	EtOHnc2	Galnc1	Galnc2
13	IDP3	YNL009W	YPDcl2	YPDcl3	EtOHcl1	EtOHcl2	Galcl1	YPDnc1	YPDnc2	YPDnc3	YPDnc4	EtOHnc1	EtOHnc2	Galnc1	Galnc2
13	AIM3	YBR108W	YPDcl2	YPDcl3	EtOHcl1	EtOHcl2	Galcl1	YPDnc1	YPDnc2	YPDnc3	YPDnc4	EtOHnc1	EtOHnc2	Galnc1	Galnc2
13	HOM6	YJR139C	YPDcl2	YPDcl3	EtOHcl1	EtOHcl2	Galcl1	YPDnc1	YPDnc2	YPDnc3	YPDnc4	EtOHnc1	EtOHnc2	Galnc1	Galnc2
13	SRP14	YDL092W	YPDcl2	YPDcl3	EtOHcl1	EtOHcl2	Galcl1	YPDnc1	YPDnc2	YPDnc3	YPDnc4	EtOHnc1	EtOHnc2	Galnc1	Galnc2
13	GYP6	YJL044C	YPDcl2	YPDcl3	EtOHcl1	EtOHcl2	Galcl1	YPDnc1	YPDnc2	YPDnc3	YPDnc4	EtOHnc1	EtOHnc2	Galnc1	Galnc2
13	FLD1	YLR404W	YPDcl2	YPDcl3	EtOHcl1	EtOHcl2	Galcl1	YPDnc1	YPDnc2	YPDnc3	YPDnc4	EtOHnc1	EtOHnc2	Galnc1	Galnc2
13		YCL058W-A	YPDcl2	YPDcl3	EtOHcl1	EtOHcl2	Galcl1	YPDnc1	YPDnc2	YPDnc3	YPDnc4	EtOHnc1	EtOHnc2	Galnc1	Galnc2
13	RTT106	YNL206C	YPDcl2	YPDcl3	EtOHcl1	EtOHcl2	Galcl1	YPDnc1	YPDnc2	YPDnc3	YPDnc4	EtOHnc1	EtOHnc2	Galnc1	Galnc2
13	CDS1	YBR029C	YPDcl2	YPDcl3	EtOHcl1	EtOHcl2	Galcl1	YPDnc1	YPDnc2	YPDnc3	YPDnc4	EtOHnc1	EtOHnc2	Galnc1	Galnc2
13	BCS1	YDR375C	YPDcl2	YPDcl3	EtOHcl1	EtOHcl2	Galcl1	YPDnc1	YPDnc2	YPDnc3	YPDnc4	EtOHnc1	EtOHnc2	Galnc1	Galnc2
13	ANR2	YKL047W	YPDcl2	YPDcl3	EtOHcl1	EtOHcl2	Galcl1	YPDnc1	YPDnc2	YPDnc3	YPDnc4	EtOHnc1	EtOHnc2	Galnc1	Galnc2
13	STP3	YLR375W	YPDcl2	YPDcl3	EtOHcl1	EtOHcl2	Galcl1	YPDnc1	YPDnc2	YPDnc3	YPDnc4	EtOHnc1	EtOHnc2	Galnc1	Galnc2
13	RTC6	YPL183W-A	YPDcl2	YPDcl3	EtOHcl1	EtOHcl2	Galcl1	YPDnc1	YPDnc2	YPDnc3	YPDnc4	EtOHnc1	EtOHnc2	Galnc1	Galnc2
13	RRD1	YIL153W	YPDcl2	YPDcl3	EtOHcl1	EtOHcl2	Galcl1	YPDnc1	YPDnc2	YPDnc3	YPDnc4	EtOHnc1	EtOHnc2	Galnc1	Galnc2
13		YLR252W	YPDcl2	YPDcl3	EtOHcl1	EtOHcl2	Galcl1	YPDnc1	YPDnc2	YPDnc3	YPDnc4	EtOHnc1	EtOHnc2	Galnc1	Galnc2
13		YER010C	YPDcl2	YPDcl3	EtOHcl1	EtOHcl2	Galcl1	YPDnc1	YPDnc2	YPDnc3	YPDnc4	EtOHnc1	EtOHnc2	Galnc1	Galnc2
13	ESP1	YGR098C	YPDcl2	YPDcl3	EtOHcl1	EtOHcl2	Galcl1	YPDnc1	YPDnc2	YPDnc3	YPDnc4	EtOHnc1	EtOHnc2	Galnc1	Galnc2
13	SKG6	YHR149C	YPDcl2	YPDcl3	EtOHcl1	EtOHcl2	Galcl1	YPDnc1	YPDnc2	YPDnc3	YPDnc4	EtOHnc1	EtOHnc2	Galnc1	Galnc2
13	SHR3	YDL212W	YPDcl2	YPDcl3	EtOHcl1	EtOHcl2	Galcl1	YPDnc1	YPDnc2	YPDnc3	YPDnc4	EtOHnc1	EtOHnc2	Galnc1	Galnc2
13		YJL068C	YPDcl2	YPDcl3	EtOHcl1	EtOHcl2	Galcl1	YPDnc1	YPDnc2	YPDnc3	YPDnc4	EtOHnc1	EtOHnc2	Galnc1	Galnc2
13	SCL1	YGL011C	YPDcl2	YPDcl3	EtOHcl1	EtOHcl2	Galcl1	YPDnc1	YPDnc2	YPDnc3	YPDnc4	EtOHnc1	EtOHnc2	Galnc1	Galnc2
13	CSE1	YGL238W	YPDcl2	YPDcl3	EtOHcl1	EtOHcl2	Galcl1	YPDnc1	YPDnc2	YPDnc3	YPDnc4	EtOHnc1	EtOHnc2	Galnc1	Galnc2
13	RFS1	YBR052C	YPDcl2	YPDcl3	EtOHcl1	EtOHcl2	Galcl1	YPDnc1	YPDnc2	YPDnc3	YPDnc4	EtOHnc1	EtOHnc2	Galnc1	Galnc2

13	ASA1	YPR085C	YPDcl2	YPDcl3	EtOHcl1	EtOHcl2	Galcl1	YPDnc1	YPDnc2	YPDnc3	YPDnc4	EtOHnc1	EtOHnc2	Galnc1	Galnc2
13	MET16	YPR167C	YPDcl2	YPDcl3	EtOHcl1	EtOHcl2	Galcl1	YPDnc1	YPDnc2	YPDnc3	YPDnc4	EtOHnc1	EtOHnc2	Galnc1	Galnc2
13	CAJ1	YER048C	YPDcl2	YPDcl3	EtOHcl1	EtOHcl2	Galcl1	YPDnc1	YPDnc2	YPDnc3	YPDnc4	EtOHnc1	EtOHnc2	Galnc1	Galnc2
13	DBP8	YHR169W	YPDcl2	YPDcl3	EtOHcl1	EtOHcl2	Galcl1	YPDnc1	YPDnc2	YPDnc3	YPDnc4	EtOHnc1	EtOHnc2	Galnc1	Galnc2
13	SFH1	YLR321C	YPDcl2	YPDcl3	EtOHcl1	EtOHcl2	Galcl1	YPDnc1	YPDnc2	YPDnc3	YPDnc4	EtOHnc1	EtOHnc2	Galnc1	Galnc2
13		YFL034W	YPDcl2	YPDcl3	EtOHcl1	EtOHcl2	Galcl1	YPDnc1	YPDnc2	YPDnc3	YPDnc4	EtOHnc1	EtOHnc2	Galnc1	Galnc2
13		YIR023C-A	YPDcl2	YPDcl3	EtOHcl1	EtOHcl2	Galcl1	YPDnc1	YPDnc2	YPDnc3	YPDnc4	EtOHnc1	EtOHnc2	Galnc1	Galnc2
13		YIL032C	YPDcl2	YPDcl3	EtOHcl1	EtOHcl2	Galcl1	YPDnc1	YPDnc2	YPDnc3	YPDnc4	EtOHnc1	EtOHnc2	Galnc1	Galnc2
13	VPH1	YOR270C	YPDcl2	YPDcl3	EtOHcl1	EtOHcl2	Galcl1	YPDnc1	YPDnc2	YPDnc3	YPDnc4	EtOHnc1	EtOHnc2	Galnc1	Galnc2
13		YNL184C	YPDcl2	YPDcl3	EtOHcl1	EtOHcl2	Galcl1	YPDnc1	YPDnc2	YPDnc3	YPDnc4	EtOHnc1	EtOHnc2	Galnc1	Galnc2
13	ACO1	YLR304C	YPDcl2	YPDcl3	EtOHcl1	EtOHcl2	Galcl1	YPDnc1	YPDnc2	YPDnc3	YPDnc4	EtOHnc1	EtOHnc2	Galnc1	Galnc2
13	ATE1	YGL017W	YPDcl2	YPDcl3	EtOHcl1	EtOHcl2	Galcl1	YPDnc1	YPDnc2	YPDnc3	YPDnc4	EtOHnc1	EtOHnc2	Galnc1	Galnc2
13		YHR145C	YPDcl2	YPDcl3	EtOHcl1	EtOHcl2	Galcl1	YPDnc1	YPDnc2	YPDnc3	YPDnc4	EtOHnc1	EtOHnc2	Galnc1	Galnc2
13		YDR133C	YPDcl2	YPDcl3	EtOHcl1	EtOHcl2	Galcl1	YPDnc1	YPDnc2	YPDnc3	YPDnc4	EtOHnc1	EtOHnc2	Galnc1	Galnc2
13	YVC1	YOR087W	YPDcl2	YPDcl3	EtOHcl1	EtOHcl2	Galcl1	YPDnc1	YPDnc2	YPDnc3	YPDnc4	EtOHnc1	EtOHnc2	Galnc1	Galnc2
13	STE4	YOR212W	YPDcl2	YPDcl3	EtOHcl1	EtOHcl2	Galcl1	YPDnc1	YPDnc2	YPDnc3	YPDnc4	EtOHnc1	EtOHnc2	Galnc1	Galnc2
13	HXK1	YFR053C	YPDcl2	YPDcl3	EtOHcl1	EtOHcl2	Galcl1	YPDnc1	YPDnc2	YPDnc3	YPDnc4	EtOHnc1	EtOHnc2	Galnc1	Galnc2
13	FMC1	YIL098C	YPDcl2	YPDcl3	EtOHcl1	EtOHcl2	Galcl1	YPDnc1	YPDnc2	YPDnc3	YPDnc4	EtOHnc1	EtOHnc2	Galnc1	Galnc2
13	MSC1	YML128C	YPDcl2	YPDcl3	EtOHcl1	EtOHcl2	Galcl1	YPDnc1	YPDnc2	YPDnc3	YPDnc4	EtOHnc1	EtOHnc2	Galnc1	Galnc2
13	RRP6	YOR001W	YPDcl2	YPDcl3	EtOHcl1	EtOHcl2	Galcl1	YPDnc1	YPDnc2	YPDnc3	YPDnc4	EtOHnc1	EtOHnc2	Galnc1	Galnc2
13	SAK1	YER129W	YPDcl2	YPDcl3	EtOHcl1	EtOHcl2	Galcl1	YPDnc1	YPDnc2	YPDnc3	YPDnc4	EtOHnc1	EtOHnc2	Galnc1	Galnc2
13		YOR304C-A	YPDcl2	YPDcl3	EtOHcl1	EtOHcl2	Galcl1	YPDnc1	YPDnc2	YPDnc3	YPDnc4	EtOHnc1	EtOHnc2	Galnc1	Galnc2
13	ECM15	YBL001C	YPDcl2	YPDcl3	EtOHcl1	EtOHcl2	Galcl1	YPDnc1	YPDnc2	YPDnc3	YPDnc4	EtOHnc1	EtOHnc2	Galnc1	Galnc2
13	SGT1	YOR057W	YPDcl2	YPDcl3	EtOHcl1	EtOHcl2	Galcl1	YPDnc1	YPDnc2	YPDnc3	YPDnc4	EtOHnc1	EtOHnc2	Galnc1	Galnc2
13	FRQ1	YDR373W	YPDcl2	YPDcl3	EtOHcl1	EtOHcl2	Galcl1	YPDnc1	YPDnc2	YPDnc3	YPDnc4	EtOHnc1	EtOHnc2	Galnc1	Galnc2
13	PRP31	YGR091W	YPDcl2	YPDcl3	EtOHcl1	EtOHcl2	Galcl1	YPDnc1	YPDnc2	YPDnc3	YPDnc4	EtOHnc1	EtOHnc2	Galnc1	Galnc2
13	CSF1	YLR087C	YPDcl2	YPDcl3	EtOHcl1	EtOHcl2	Galcl1	YPDnc1	YPDnc2	YPDnc3	YPDnc4	EtOHnc1	EtOHnc2	Galnc1	Galnc2
13	CYS3	YAL012W	YPDcl2	YPDcl3	EtOHcl1	EtOHcl2	Galcl1	YPDnc1	YPDnc2	YPDnc3	YPDnc4	EtOHnc1	EtOHnc2	Galnc1	Galnc2
13	IZH2	YOL002C	YPDcl2	YPDcl3	EtOHcl1	EtOHcl2	Galcl1	YPDnc1	YPDnc2	YPDnc3	YPDnc4	EtOHnc1	EtOHnc2	Galnc1	Galnc2
13	RTF1	YGL244W	YPDcl2	YPDcl3	EtOHcl1	EtOHcl2	Galcl1	YPDnc1	YPDnc2	YPDnc3	YPDnc4	EtOHnc1	EtOHnc2	Galnc1	Galnc2
13	GRX1	YCL035C	YPDcl2	YPDcl3	EtOHcl1	EtOHcl2	Galcl1	YPDnc1	YPDnc2	YPDnc3	YPDnc4	EtOHnc1	EtOHnc2	Galnc1	Galnc2
13	URN1	YPR152C	YPDcl2	YPDcl3	EtOHcl1	EtOHcl2	Galcl1	YPDnc1	YPDnc2	YPDnc3	YPDnc4	EtOHnc1	EtOHnc2	Galnc1	Galnc2
13	TFG2	YGR005C	YPDcl2	YPDcl3	EtOHcl1	EtOHcl2	Galcl1	YPDnc1	YPDnc2	YPDnc3	YPDnc4	EtOHnc1	EtOHnc2	Galnc1	Galnc2
13	SUN4	YNL066W	YPDcl2	YPDcl3	EtOHcl1	EtOHcl2	Galcl1	YPDnc1	YPDnc2	YPDnc3	YPDnc4	EtOHnc1	EtOHnc2	Galnc1	Galnc2

13		YNL235C	YPDcl2	YPDcl3	EtOHcl1	EtOHcl2	Galcl1	YPDnc1	YPDnc2	YPDnc3	YPDnc4	EtOHnc1	EtOHnc2	Galnc1	Galnc2
13		YOL092W	YPDcl2	YPDcl3	EtOHcl1	EtOHcl2	Galcl1	YPDnc1	YPDnc2	YPDnc3	YPDnc4	EtOHnc1	EtOHnc2	Galnc1	Galnc2
13		YJL107C	YPDcl2	YPDcl3	EtOHcl1	EtOHcl2	Galcl1	YPDnc1	YPDnc2	YPDnc3	YPDnc4	EtOHnc1	EtOHnc2	Galnc1	Galnc2
13	ADD66	YKL206C	YPDcl2	YPDcl3	EtOHcl1	EtOHcl2	Galcl1	YPDnc1	YPDnc2	YPDnc3	YPDnc4	EtOHnc1	EtOHnc2	Galnc1	Galnc2
13		YGL014C-A	YPDcl2	YPDcl3	EtOHcl1	EtOHcl2	Galcl1	YPDnc1	YPDnc2	YPDnc3	YPDnc4	EtOHnc1	EtOHnc2	Galnc1	Galnc2
13	MGR3	YMR115W	YPDcl2	YPDcl3	EtOHcl1	EtOHcl2	Galcl1	YPDnc1	YPDnc2	YPDnc3	YPDnc4	EtOHnc1	EtOHnc2	Galnc1	Galnc2
13		YCL048W-/	YPDcl2	YPDcl3	EtOHcl1	EtOHcl2	Galcl1	YPDnc1	YPDnc2	YPDnc3	YPDnc4	EtOHnc1	EtOHnc2	Galnc1	Galnc2
13	VAB2	YEL005C	YPDcl2	YPDcl3	EtOHcl1	EtOHcl2	Galcl1	YPDnc1	YPDnc2	YPDnc3	YPDnc4	EtOHnc1	EtOHnc2	Galnc1	Galnc2
13	MET28	YIR017C	YPDcl2	YPDcl3	EtOHcl1	EtOHcl2	Galcl1	YPDnc1	YPDnc2	YPDnc3	YPDnc4	EtOHnc1	EtOHnc2	Galnc1	Galnc2
13	UBR1	YGR184C	YPDcl2	YPDcl3	EtOHcl1	EtOHcl2	Galcl1	YPDnc1	YPDnc2	YPDnc3	YPDnc4	EtOHnc1	EtOHnc2	Galnc1	Galnc2
13	PTM1	YKL039W	YPDcl2	YPDcl3	EtOHcl1	EtOHcl2	Galcl1	YPDnc1	YPDnc2	YPDnc3	YPDnc4	EtOHnc1	EtOHnc2	Galnc1	Galnc2
13	SCW11	YGL028C	YPDcl2	YPDcl3	EtOHcl1	EtOHcl2	Galcl1	YPDnc1	YPDnc2	YPDnc3	YPDnc4	EtOHnc1	EtOHnc2	Galnc1	Galnc2
13	SAS2	YMR127C	YPDcl2	YPDcl3	EtOHcl1	EtOHcl2	Galcl1	YPDnc1	YPDnc2	YPDnc3	YPDnc4	EtOHnc1	EtOHnc2	Galnc1	Galnc2
13	VHS1	YDR247W	YPDcl2	YPDcl3	EtOHcl1	EtOHcl2	Galcl1	YPDnc1	YPDnc2	YPDnc3	YPDnc4	EtOHnc1	EtOHnc2	Galnc1	Galnc2
13		YBL029C-A	YPDcl2	YPDcl3	EtOHcl1	EtOHcl2	Galcl1	YPDnc1	YPDnc2	YPDnc3	YPDnc4	EtOHnc1	EtOHnc2	Galnc1	Galnc2
13	RSC4	YKR008W	YPDcl2	YPDcl3	EtOHcl1	EtOHcl2	Galcl1	YPDnc1	YPDnc2	YPDnc3	YPDnc4	EtOHnc1	EtOHnc2	Galnc1	Galnc2
13	IRC6	YFR043C	YPDcl2	YPDcl3	EtOHcl1	EtOHcl2	Galcl1	YPDnc1	YPDnc2	YPDnc3	YPDnc4	EtOHnc1	EtOHnc2	Galnc1	Galnc2
13		YIL029C	YPDcl2	YPDcl3	EtOHcl1	EtOHcl2	Galcl1	YPDnc1	YPDnc2	YPDnc3	YPDnc4	EtOHnc1	EtOHnc2	Galnc1	Galnc2
13	OSW1	YOR255W	YPDcl2	YPDcl3	EtOHcl1	EtOHcl2	Galcl1	YPDnc1	YPDnc2	YPDnc3	YPDnc4	EtOHnc1	EtOHnc2	Galnc1	Galnc2
13	RFA2	YNL312W	YPDcl2	YPDcl3	EtOHcl1	EtOHcl2	Galcl1	YPDnc1	YPDnc2	YPDnc3	YPDnc4	EtOHnc1	EtOHnc2	Galnc1	Galnc2
13	AST2	YER101C	YPDcl2	YPDcl3	EtOHcl1	EtOHcl2	Galcl1	YPDnc1	YPDnc2	YPDnc3	YPDnc4	EtOHnc1	EtOHnc2	Galnc1	Galnc2
13	GAS3	YMR215W	YPDcl2	YPDcl3	EtOHcl1	EtOHcl2	Galcl1	YPDnc1	YPDnc2	YPDnc3	YPDnc4	EtOHnc1	EtOHnc2	Galnc1	Galnc2
13	CPS1	YJL172W	YPDcl2	YPDcl3	EtOHcl1	EtOHcl2	Galcl1	YPDnc1	YPDnc2	YPDnc3	YPDnc4	EtOHnc1	EtOHnc2	Galnc1	Galnc2
13	BUR6	YER159C	YPDcl2	YPDcl3	EtOHcl1	EtOHcl2	Galcl1	YPDnc1	YPDnc2	YPDnc3	YPDnc4	EtOHnc1	EtOHnc2	Galnc1	Galnc2
13		YEL047C	YPDcl2	YPDcl3	EtOHcl1	EtOHcl2	Galcl1	YPDnc1	YPDnc2	YPDnc3	YPDnc4	EtOHnc1	EtOHnc2	Galnc1	Galnc2
13	ORC6	YHR118C	YPDcl2	YPDcl3	EtOHcl1	EtOHcl2	Galcl1	YPDnc1	YPDnc2	YPDnc3	YPDnc4	EtOHnc1	EtOHnc2	Galnc1	Galnc2
13	ATP7	YKL016C	YPDcl2	YPDcl3	EtOHcl1	EtOHcl2	Galcl1	YPDnc1	YPDnc2	YPDnc3	YPDnc4	EtOHnc1	EtOHnc2	Galnc1	Galnc2
13	TFB4	YPR056W	YPDcl2	YPDcl3	EtOHcl1	EtOHcl2	Galcl1	YPDnc1	YPDnc2	YPDnc3	YPDnc4	EtOHnc1	EtOHnc2	Galnc1	Galnc2
13		YHL045W	YPDcl2	YPDcl3	EtOHcl1	EtOHcl2	Galcl1	YPDnc1	YPDnc2	YPDnc3	YPDnc4	EtOHnc1	EtOHnc2	Galnc1	Galnc2
13	DER1	YBR201W	YPDcl2	YPDcl3	EtOHcl1	EtOHcl2	Galcl1	YPDnc1	YPDnc2	YPDnc3	YPDnc4	EtOHnc1	EtOHnc2	Galnc1	Galnc2
13	UBP5	YER144C	YPDcl2	YPDcl3	EtOHcl1	EtOHcl2	Galcl1	YPDnc1	YPDnc2	YPDnc3	YPDnc4	EtOHnc1	EtOHnc2	Galnc1	Galnc2
13	YPT10	YBR264C	YPDcl2	YPDcl3	EtOHcl1	EtOHcl2	Galcl1	YPDnc1	YPDnc2	YPDnc3	YPDnc4	EtOHnc1	EtOHnc2	Galnc1	Galnc2
13		YDR239C	YPDcl2	YPDcl3	EtOHcl1	EtOHcl2	Galcl1	YPDnc1	YPDnc2	YPDnc3	YPDnc4	EtOHnc1	EtOHnc2	Galnc1	Galnc2
13	PPZ1	YML016C	YPDcl2	YPDcl3	EtOHcl1	EtOHcl2	Galcl1	YPDnc1	YPDnc2	YPDnc3	YPDnc4	EtOHnc1	EtOHnc2	Galnc1	Galnc2

13		YDL180W	YPDcl2	YPDcl3	EtOHcl1	EtOHcl2	Galcl1	YPDnc1	YPDnc2	YPDnc3	YPDnc4	EtOHnc1	EtOHnc2	Galnc1	Galnc2
13	PCM1	YEL058W	YPDcl2	YPDcl3	EtOHcl1	EtOHcl2	Galcl1	YPDnc1	YPDnc2	YPDnc3	YPDnc4	EtOHnc1	EtOHnc2	Galnc1	Galnc2
13	DPH5	YLR172C	YPDcl2	YPDcl3	EtOHcl1	EtOHcl2	Galcl1	YPDnc1	YPDnc2	YPDnc3	YPDnc4	EtOHnc1	EtOHnc2	Galnc1	Galnc2
13	SCO2	YBR024W	YPDcl2	YPDcl3	EtOHcl1	EtOHcl2	Galcl1	YPDnc1	YPDnc2	YPDnc3	YPDnc4	EtOHnc1	EtOHnc2	Galnc1	Galnc2
13	AHC1	YOR023C	YPDcl2	YPDcl3	EtOHcl1	EtOHcl2	Galcl1	YPDnc1	YPDnc2	YPDnc3	YPDnc4	EtOHnc1	EtOHnc2	Galnc1	Galnc2
13	SET2	YJL168C	YPDcl2	YPDcl3	EtOHcl1	EtOHcl2	Galcl1	YPDnc1	YPDnc2	YPDnc3	YPDnc4	EtOHnc1	EtOHnc2	Galnc1	Galnc2
13	PUS1	YPL212C	YPDcl2	YPDcl3	EtOHcl1	EtOHcl2	Galcl1	YPDnc1	YPDnc2	YPDnc3	YPDnc4	EtOHnc1	EtOHnc2	Galnc1	Galnc2
13	SNX4	YJL036W	YPDcl2	YPDcl3	EtOHcl1	EtOHcl2	Galcl1	YPDnc1	YPDnc2	YPDnc3	YPDnc4	EtOHnc1	EtOHnc2	Galnc1	Galnc2
13	BRX1	YOL077C	YPDcl2	YPDcl3	EtOHcl1	EtOHcl2	Galcl1	YPDnc1	YPDnc2	YPDnc3	YPDnc4	EtOHnc1	EtOHnc2	Galnc1	Galnc2
13	ECM25	YJL201W	YPDcl2	YPDcl3	EtOHcl1	EtOHcl2	Galcl1	YPDnc1	YPDnc2	YPDnc3	YPDnc4	EtOHnc1	EtOHnc2	Galnc1	Galnc2
13		YPL141C	YPDcl2	YPDcl3	EtOHcl1	EtOHcl2	Galcl1	YPDnc1	YPDnc2	YPDnc3	YPDnc4	EtOHnc1	EtOHnc2	Galnc1	Galnc2
13	CIT3	YPR001W	YPDcl2	YPDcl3	EtOHcl1	EtOHcl2	Galcl1	YPDnc1	YPDnc2	YPDnc3	YPDnc4	EtOHnc1	EtOHnc2	Galnc1	Galnc2
13	CKS1	YBR135W	YPDcl2	YPDcl3	EtOHcl1	EtOHcl2	Galcl1	YPDnc1	YPDnc2	YPDnc3	YPDnc4	EtOHnc1	EtOHnc2	Galnc1	Galnc2
13	PFS2	YNL317W	YPDcl2	YPDcl3	EtOHcl1	EtOHcl2	Galcl1	YPDnc1	YPDnc2	YPDnc3	YPDnc4	EtOHnc1	EtOHnc2	Galnc1	Galnc2
13	IRC19	YLL033W	YPDcl2	YPDcl3	EtOHcl1	EtOHcl2	Galcl1	YPDnc1	YPDnc2	YPDnc3	YPDnc4	EtOHnc1	EtOHnc2	Galnc1	Galnc2
13	FHL1	YPR104C	YPDcl2	YPDcl3	EtOHcl1	EtOHcl2	Galcl1	YPDnc1	YPDnc2	YPDnc3	YPDnc4	EtOHnc1	EtOHnc2	Galnc1	Galnc2
13	HUT1	YPL244C	YPDcl2	YPDcl3	EtOHcl1	EtOHcl2	Galcl1	YPDnc1	YPDnc2	YPDnc3	YPDnc4	EtOHnc1	EtOHnc2	Galnc1	Galnc2
13	FRE2	YKL220C	YPDcl2	YPDcl3	EtOHcl1	EtOHcl2	Galcl1	YPDnc1	YPDnc2	YPDnc3	YPDnc4	EtOHnc1	EtOHnc2	Galnc1	Galnc2
13	YRB30	YGL164C	YPDcl2	YPDcl3	EtOHcl1	EtOHcl2	Galcl1	YPDnc1	YPDnc2	YPDnc3	YPDnc4	EtOHnc1	EtOHnc2	Galnc1	Galnc2
13	ATG11	YPR049C	YPDcl2	YPDcl3	EtOHcl1	EtOHcl2	Galcl1	YPDnc1	YPDnc2	YPDnc3	YPDnc4	EtOHnc1	EtOHnc2	Galnc1	Galnc2
13	SED5	YLR026C	YPDcl2	YPDcl3	EtOHcl1	EtOHcl2	Galcl1	YPDnc1	YPDnc2	YPDnc3	YPDnc4	EtOHnc1	EtOHnc2	Galnc1	Galnc2
13	MGT1	YDL200C	YPDcl2	YPDcl3	EtOHcl1	EtOHcl2	Galcl1	YPDnc1	YPDnc2	YPDnc3	YPDnc4	EtOHnc1	EtOHnc2	Galnc1	Galnc2
13	UIP3	YAR027W	YPDcl2	YPDcl3	EtOHcl1	EtOHcl2	Galcl1	YPDnc1	YPDnc2	YPDnc3	YPDnc4	EtOHnc1	EtOHnc2	Galnc1	Galnc2
13	YAP5	YIR018W	YPDcl2	YPDcl3	EtOHcl1	EtOHcl2	Galcl1	YPDnc1	YPDnc2	YPDnc3	YPDnc4	EtOHnc1	EtOHnc2	Galnc1	Galnc2
13	POR2	YIL114C	YPDcl2	YPDcl3	EtOHcl1	EtOHcl2	Galcl1	YPDnc1	YPDnc2	YPDnc3	YPDnc4	EtOHnc1	EtOHnc2	Galnc1	Galnc2
13	GYP8	YFL027C	YPDcl2	YPDcl3	EtOHcl1	EtOHcl2	Galcl1	YPDnc1	YPDnc2	YPDnc3	YPDnc4	EtOHnc1	EtOHnc2	Galnc1	Galnc2
13	CSR1	YLR380W	YPDcl2	YPDcl3	EtOHcl1	EtOHcl2	Galcl1	YPDnc1	YPDnc2	YPDnc3	YPDnc4	EtOHnc1	EtOHnc2	Galnc1	Galnc2
13		YGR169C-A	YPDcl2	YPDcl3	EtOHcl1	EtOHcl2	Galcl1	YPDnc1	YPDnc2	YPDnc3	YPDnc4	EtOHnc1	EtOHnc2	Galnc1	Galnc2
13	GIM3	YNL153C	YPDcl2	YPDcl3	EtOHcl1	EtOHcl2	Galcl1	YPDnc1	YPDnc2	YPDnc3	YPDnc4	EtOHnc1	EtOHnc2	Galnc1	Galnc2
13		YGR079W	YPDcl2	YPDcl3	EtOHcl1	EtOHcl2	Galcl1	YPDnc1	YPDnc2	YPDnc3	YPDnc4	EtOHnc1	EtOHnc2	Galnc1	Galnc2
13	NIT1	YIL164C	YPDcl2	YPDcl3	EtOHcl1	EtOHcl2	Galcl1	YPDnc1	YPDnc2	YPDnc3	YPDnc4	EtOHnc1	EtOHnc2	Galnc1	Galnc2
13	MRPL39	YML009C	YPDcl2	YPDcl3	EtOHcl1	EtOHcl2	Galcl1	YPDnc1	YPDnc2	YPDnc3	YPDnc4	EtOHnc1	EtOHnc2	Galnc1	Galnc2
13		YOL073C	YPDcl2	YPDcl3	EtOHcl1	EtOHcl2	Galcl1	YPDnc1	YPDnc2	YPDnc3	YPDnc4	EtOHnc1	EtOHnc2	Galnc1	Galnc2
13	GAD1	YMR250W	YPDcl2	YPDcl3	EtOHcl1	EtOHcl2	Galcl1	YPDnc1	YPDnc2	YPDnc3	YPDnc4	EtOHnc1	EtOHnc2	Galnc1	Galnc2

13		YDR444W	YPDcl2	YPDcl3	EtOHcl1	EtOHcl2	Galcl1	YPDnc1	YPDnc2	YPDnc3	YPDnc4	EtOHnc1	EtOHnc2	Galnc1	Galnc2
13	GIN4	YDR507C	YPDcl2	YPDcl3	EtOHcl1	EtOHcl2	Galcl1	YPDnc1	YPDnc2	YPDnc3	YPDnc4	EtOHnc1	EtOHnc2	Galnc1	Galnc2
13	RDS3	YPR094W	YPDcl2	YPDcl3	EtOHcl1	EtOHcl2	Galcl1	YPDnc1	YPDnc2	YPDnc3	YPDnc4	EtOHnc1	EtOHnc2	Galnc1	Galnc2
13	ADH2	YMR303C	YPDcl2	YPDcl3	EtOHcl1	EtOHcl2	Galcl1	YPDnc1	YPDnc2	YPDnc3	YPDnc4	EtOHnc1	EtOHnc2	Galnc1	Galnc2
13	PRP5	YBR237W	YPDcl2	YPDcl3	EtOHcl1	EtOHcl2	Galcl1	YPDnc1	YPDnc2	YPDnc3	YPDnc4	EtOHnc1	EtOHnc2	Galnc1	Galnc2
13	SPC25	YER018C	YPDcl2	YPDcl3	EtOHcl1	EtOHcl2	Galcl1	YPDnc1	YPDnc2	YPDnc3	YPDnc4	EtOHnc1	EtOHnc2	Galnc1	Galnc2
13	YSF3	YNL138W-A	YPDcl2	YPDcl3	EtOHcl1	EtOHcl2	Galcl1	YPDnc1	YPDnc2	YPDnc3	YPDnc4	EtOHnc1	EtOHnc2	Galnc1	Galnc2
13	RPP1B	YDL130W	YPDcl2	YPDcl3	EtOHcl1	EtOHcl2	Galcl1	YPDnc1	YPDnc2	YPDnc3	YPDnc4	EtOHnc1	EtOHnc2	Galnc1	Galnc2
13	YAP1801	YHR161C	YPDcl2	YPDcl3	EtOHcl1	EtOHcl2	Galcl1	YPDnc1	YPDnc2	YPDnc3	YPDnc4	EtOHnc1	EtOHnc2	Galnc1	Galnc2
13	MRPL1	YDR116C	YPDcl2	YPDcl3	EtOHcl1	EtOHcl2	Galcl1	YPDnc1	YPDnc2	YPDnc3	YPDnc4	EtOHnc1	EtOHnc2	Galnc1	Galnc2
13	AMD2	YDR242W	YPDcl2	YPDcl3	EtOHcl1	EtOHcl2	Galcl1	YPDnc1	YPDnc2	YPDnc3	YPDnc4	EtOHnc1	EtOHnc2	Galnc1	Galnc2
13	CTR9	YOL145C	YPDcl2	YPDcl3	EtOHcl1	EtOHcl2	Galcl1	YPDnc1	YPDnc2	YPDnc3	YPDnc4	EtOHnc1	EtOHnc2	Galnc1	Galnc2
13	SNF2	YOR290C	YPDcl2	YPDcl3	EtOHcl1	EtOHcl2	Galcl1	YPDnc1	YPDnc2	YPDnc3	YPDnc4	EtOHnc1	EtOHnc2	Galnc1	Galnc2
13	GEA1	YJR031C	YPDcl2	YPDcl3	EtOHcl1	EtOHcl2	Galcl1	YPDnc1	YPDnc2	YPDnc3	YPDnc4	EtOHnc1	EtOHnc2	Galnc1	Galnc2
13	PAM17	YKR065C	YPDcl2	YPDcl3	EtOHcl1	EtOHcl2	Galcl1	YPDnc1	YPDnc2	YPDnc3	YPDnc4	EtOHnc1	EtOHnc2	Galnc1	Galnc2
13		YBR139W	YPDcl2	YPDcl3	EtOHcl1	EtOHcl2	Galcl1	YPDnc1	YPDnc2	YPDnc3	YPDnc4	EtOHnc1	EtOHnc2	Galnc1	Galnc2
13	ATG21	YPL100W	YPDcl2	YPDcl3	EtOHcl1	EtOHcl2	Galcl1	YPDnc1	YPDnc2	YPDnc3	YPDnc4	EtOHnc1	EtOHnc2	Galnc1	Galnc2
13	NOP53	YPL146C	YPDcl2	YPDcl3	EtOHcl1	EtOHcl2	Galcl1	YPDnc1	YPDnc2	YPDnc3	YPDnc4	EtOHnc1	EtOHnc2	Galnc1	Galnc2
13	KAR4	YCL055W	YPDcl2	YPDcl3	EtOHcl1	EtOHcl2	Galcl1	YPDnc1	YPDnc2	YPDnc3	YPDnc4	EtOHnc1	EtOHnc2	Galnc1	Galnc2
13	RPS17A	YML024W	YPDcl2	YPDcl3	EtOHcl1	EtOHcl2	Galcl1	YPDnc1	YPDnc2	YPDnc3	YPDnc4	EtOHnc1	EtOHnc2	Galnc1	Galnc2
13	MRPL22	YNL177C	YPDcl2	YPDcl3	EtOHcl1	EtOHcl2	Galcl1	YPDnc1	YPDnc2	YPDnc3	YPDnc4	EtOHnc1	EtOHnc2	Galnc1	Galnc2
13	BCH2	YKR027W	YPDcl2	YPDcl3	EtOHcl1	EtOHcl2	Galcl1	YPDnc1	YPDnc2	YPDnc3	YPDnc4	EtOHnc1	EtOHnc2	Galnc1	Galnc2
13		YBR138C	YPDcl2	YPDcl3	EtOHcl1	EtOHcl2	Galcl1	YPDnc1	YPDnc2	YPDnc3	YPDnc4	EtOHnc1	EtOHnc2	Galnc1	Galnc2
13		YJL055W	YPDcl2	YPDcl3	EtOHcl1	EtOHcl2	Galcl1	YPDnc1	YPDnc2	YPDnc3	YPDnc4	EtOHnc1	EtOHnc2	Galnc1	Galnc2
13	RPS21B	YJL136C	YPDcl2	YPDcl3	EtOHcl1	EtOHcl2	Galcl1	YPDnc1	YPDnc2	YPDnc3	YPDnc4	EtOHnc1	EtOHnc2	Galnc1	Galnc2
13	MLH2	YLR035C	YPDcl2	YPDcl3	EtOHcl1	EtOHcl2	Galcl1	YPDnc1	YPDnc2	YPDnc3	YPDnc4	EtOHnc1	EtOHnc2	Galnc1	Galnc2
13		YBR016W	YPDcl2	YPDcl3	EtOHcl1	EtOHcl2	Galcl1	YPDnc1	YPDnc2	YPDnc3	YPDnc4	EtOHnc1	EtOHnc2	Galnc1	Galnc2
13		YFL054C	YPDcl2	YPDcl3	EtOHcl1	EtOHcl2	Galcl1	YPDnc1	YPDnc2	YPDnc3	YPDnc4	EtOHnc1	EtOHnc2	Galnc1	Galnc2
13	RPC37	YKR025W	YPDcl2	YPDcl3	EtOHcl1	EtOHcl2	Galcl1	YPDnc1	YPDnc2	YPDnc3	YPDnc4	EtOHnc1	EtOHnc2	Galnc1	Galnc2
13	CHS7	YHR142W	YPDcl2	YPDcl3	EtOHcl1	EtOHcl2	Galcl1	YPDnc1	YPDnc2	YPDnc3	YPDnc4	EtOHnc1	EtOHnc2	Galnc1	Galnc2
13	ENT4	YLL038C	YPDcl2	YPDcl3	EtOHcl1	EtOHcl2	Galcl1	YPDnc1	YPDnc2	YPDnc3	YPDnc4	EtOHnc1	EtOHnc2	Galnc1	Galnc2
13		YLR146W-A	YPDcl2	YPDcl3	EtOHcl1	EtOHcl2	Galcl1	YPDnc1	YPDnc2	YPDnc3	YPDnc4	EtOHnc1	EtOHnc2	Galnc1	Galnc2
13	SPT23	YKL020C	YPDcl2	YPDcl3	EtOHcl1	EtOHcl2	Galcl1	YPDnc1	YPDnc2	YPDnc3	YPDnc4	EtOHnc1	EtOHnc2	Galnc1	Galnc2
13	MLH3	YPL164C	YPDcl2	YPDcl3	EtOHcl1	EtOHcl2	Galcl1	YPDnc1	YPDnc2	YPDnc3	YPDnc4	EtOHnc1	EtOHnc2	Galnc1	Galnc2

13	PUP3	YER094C	YPDcl2	YPDcl3	EtOHcl1	EtOHcl2	Galcl1	YPDnc1	YPDnc2	YPDnc3	YPDnc4	EtOHnc1	EtOHnc2	Galnc1	Galnc2
13	RSM26	YJR101W	YPDcl2	YPDcl3	EtOHcl1	EtOHcl2	Galcl1	YPDnc1	YPDnc2	YPDnc3	YPDnc4	EtOHnc1	EtOHnc2	Galnc1	Galnc2
13	NEJ1	YLR265C	YPDcl2	YPDcl3	EtOHcl1	EtOHcl2	Galcl1	YPDnc1	YPDnc2	YPDnc3	YPDnc4	EtOHnc1	EtOHnc2	Galnc1	Galnc2
13	RPS17B	YDR447C	YPDcl2	YPDcl3	EtOHcl1	EtOHcl2	Galcl1	YPDnc1	YPDnc2	YPDnc3	YPDnc4	EtOHnc1	EtOHnc2	Galnc1	Galnc2
13	OM45	YIL136W	YPDcl2	YPDcl3	EtOHcl1	EtOHcl2	Galcl1	YPDnc1	YPDnc2	YPDnc3	YPDnc4	EtOHnc1	EtOHnc2	Galnc1	Galnc2
13		YOR052C	YPDcl2	YPDcl3	EtOHcl1	EtOHcl2	Galcl1	YPDnc1	YPDnc2	YPDnc3	YPDnc4	EtOHnc1	EtOHnc2	Galnc1	Galnc2
13	RIB4	YOL143C	YPDcl2	YPDcl3	EtOHcl1	EtOHcl2	Galcl1	YPDnc1	YPDnc2	YPDnc3	YPDnc4	EtOHnc1	EtOHnc2	Galnc1	Galnc2
13		YPL278C	YPDcl2	YPDcl3	EtOHcl1	EtOHcl2	Galcl1	YPDnc1	YPDnc2	YPDnc3	YPDnc4	EtOHnc1	EtOHnc2	Galnc1	Galnc2
13	BUD21	YOR078W	YPDcl2	YPDcl3	EtOHcl1	EtOHcl2	Galcl1	YPDnc1	YPDnc2	YPDnc3	YPDnc4	EtOHnc1	EtOHnc2	Galnc1	Galnc2
13	DDR2	YOL052C-A	YPDcl2	YPDcl3	EtOHcl1	EtOHcl2	Galcl1	YPDnc1	YPDnc2	YPDnc3	YPDnc4	EtOHnc1	EtOHnc2	Galnc1	Galnc2
13	YME2	YMR302C	YPDcl2	YPDcl3	EtOHcl1	EtOHcl2	Galcl1	YPDnc1	YPDnc2	YPDnc3	YPDnc4	EtOHnc1	EtOHnc2	Galnc1	Galnc2
13	TIP41	YPR040W	YPDcl2	YPDcl3	EtOHcl1	EtOHcl2	Galcl1	YPDnc1	YPDnc2	YPDnc3	YPDnc4	EtOHnc1	EtOHnc2	Galnc1	Galnc2
13	CWC23	YGL128C	YPDcl2	YPDcl3	EtOHcl1	EtOHcl2	Galcl1	YPDnc1	YPDnc2	YPDnc3	YPDnc4	EtOHnc1	EtOHnc2	Galnc1	Galnc2
13	MSH3	YCR092C	YPDcl2	YPDcl3	EtOHcl1	EtOHcl2	Galcl1	YPDnc1	YPDnc2	YPDnc3	YPDnc4	EtOHnc1	EtOHnc2	Galnc1	Galnc2
13	STB6	YKL072W	YPDcl2	YPDcl3	EtOHcl1	EtOHcl2	Galcl1	YPDnc1	YPDnc2	YPDnc3	YPDnc4	EtOHnc1	EtOHnc2	Galnc1	Galnc2
13	THI74	YDR438W	YPDcl2	YPDcl3	EtOHcl1	EtOHcl2	Galcl1	YPDnc1	YPDnc2	YPDnc3	YPDnc4	EtOHnc1	EtOHnc2	Galnc1	Galnc2
13	MGR2	YPL098C	YPDcl2	YPDcl3	EtOHcl1	EtOHcl2	Galcl1	YPDnc1	YPDnc2	YPDnc3	YPDnc4	EtOHnc1	EtOHnc2	Galnc1	Galnc2
13	MVP1	YMR004W	YPDcl2	YPDcl3	EtOHcl1	EtOHcl2	Galcl1	YPDnc1	YPDnc2	YPDnc3	YPDnc4	EtOHnc1	EtOHnc2	Galnc1	Galnc2
13	HOG1	YLR113W	YPDcl2	YPDcl3	EtOHcl1	EtOHcl2	Galcl1	YPDnc1	YPDnc2	YPDnc3	YPDnc4	EtOHnc1	EtOHnc2	Galnc1	Galnc2
13	COA1	YIL157C	YPDcl2	YPDcl3	EtOHcl1	EtOHcl2	Galcl1	YPDnc1	YPDnc2	YPDnc3	YPDnc4	EtOHnc1	EtOHnc2	Galnc1	Galnc2
13		YBR141W-/	YPDcl2	YPDcl3	EtOHcl1	EtOHcl2	Galcl1	YPDnc1	YPDnc2	YPDnc3	YPDnc4	EtOHnc1	EtOHnc2	Galnc1	Galnc2
13	SUE1	YPR151C	YPDcl2	YPDcl3	EtOHcl1	EtOHcl2	Galcl1	YPDnc1	YPDnc2	YPDnc3	YPDnc4	EtOHnc1	EtOHnc2	Galnc1	Galnc2
13	TOP1	YOL006C	YPDcl2	YPDcl3	EtOHcl1	EtOHcl2	Galcl1	YPDnc1	YPDnc2	YPDnc3	YPDnc4	EtOHnc1	EtOHnc2	Galnc1	Galnc2
13	DOG1	YHR044C	YPDcl2	YPDcl3	EtOHcl1	EtOHcl2	Galcl1	YPDnc1	YPDnc2	YPDnc3	YPDnc4	EtOHnc1	EtOHnc2	Galnc1	Galnc2
13	EPL1	YFL024C	YPDcl2	YPDcl3	EtOHcl1	EtOHcl2	Galcl1	YPDnc1	YPDnc2	YPDnc3	YPDnc4	EtOHnc1	EtOHnc2	Galnc1	Galnc2
13	SCC2	YDR180W	YPDcl2	YPDcl3	EtOHcl1	EtOHcl2	Galcl1	YPDnc1	YPDnc2	YPDnc3	YPDnc4	EtOHnc1	EtOHnc2	Galnc1	Galnc2
13	KAP120	YPL125W	YPDcl2	YPDcl3	EtOHcl1	EtOHcl2	Galcl1	YPDnc1	YPDnc2	YPDnc3	YPDnc4	EtOHnc1	EtOHnc2	Galnc1	Galnc2
13		YBL077W	YPDcl2	YPDcl3	EtOHcl1	EtOHcl2	Galcl1	YPDnc1	YPDnc2	YPDnc3	YPDnc4	EtOHnc1	EtOHnc2	Galnc1	Galnc2
13	RFX1	YLR176C	YPDcl2	YPDcl3	EtOHcl1	EtOHcl2	Galcl1	YPDnc1	YPDnc2	YPDnc3	YPDnc4	EtOHnc1	EtOHnc2	Galnc1	Galnc2
13	JEM1	YJL073W	YPDcl2	YPDcl3	EtOHcl1	EtOHcl2	Galcl1	YPDnc1	YPDnc2	YPDnc3	YPDnc4	EtOHnc1	EtOHnc2	Galnc1	Galnc2
13	TEN1	YLR010C	YPDcl2	YPDcl3	EtOHcl1	EtOHcl2	Galcl1	YPDnc1	YPDnc2	YPDnc3	YPDnc4	EtOHnc1	EtOHnc2	Galnc1	Galnc2
13	GID8	YMR135C	YPDcl2	YPDcl3	EtOHcl1	EtOHcl2	Galcl1	YPDnc1	YPDnc2	YPDnc3	YPDnc4	EtOHnc1	EtOHnc2	Galnc1	Galnc2
13	EMI5	YOL071W	YPDcl2	YPDcl3	EtOHcl1	EtOHcl2	Galcl1	YPDnc1	YPDnc2	YPDnc3	YPDnc4	EtOHnc1	EtOHnc2	Galnc1	Galnc2
13	FUS2	YMR232W	YPDcl2	YPDcl3	EtOHcl1	EtOHcl2	Galcl1	YPDnc1	YPDnc2	YPDnc3	YPDnc4	EtOHnc1	EtOHnc2	Galnc1	Galnc2

13	SCS3	YGL126W	YPDcl2	YPDcl3	EtOHcl1	EtOHcl2	Galcl1	YPDnc1	YPDnc2	YPDnc3	YPDnc4	EtOHnc1	EtOHnc2	Galnc1	Galnc2
13	TRX3	YCR083W	YPDcl2	YPDcl3	EtOHcl1	EtOHcl2	Galcl1	YPDnc1	YPDnc2	YPDnc3	YPDnc4	EtOHnc1	EtOHnc2	Galnc1	Galnc2
13	SLD3	YGL113W	YPDcl2	YPDcl3	EtOHcl1	EtOHcl2	Galcl1	YPDnc1	YPDnc2	YPDnc3	YPDnc4	EtOHnc1	EtOHnc2	Galnc1	Galnc2
13	FLO8	YER109C	YPDcl2	YPDcl3	EtOHcl1	EtOHcl2	Galcl1	YPDnc1	YPDnc2	YPDnc3	YPDnc4	EtOHnc1	EtOHnc2	Galnc1	Galnc2
13		YBR200W-/	YPDcl2	YPDcl3	EtOHcl1	EtOHcl2	Galcl1	YPDnc1	YPDnc2	YPDnc3	YPDnc4	EtOHnc1	EtOHnc2	Galnc1	Galnc2
13		YNL022C	YPDcl2	YPDcl3	EtOHcl1	EtOHcl2	Galcl1	YPDnc1	YPDnc2	YPDnc3	YPDnc4	EtOHnc1	EtOHnc2	Galnc1	Galnc2
13	HUB1	YNR032C-A	YPDcl2	YPDcl3	EtOHcl1	EtOHcl2	Galcl1	YPDnc1	YPDnc2	YPDnc3	YPDnc4	EtOHnc1	EtOHnc2	Galnc1	Galnc2
13	TMA16	YOR252W	YPDcl2	YPDcl3	EtOHcl1	EtOHcl2	Galcl1	YPDnc1	YPDnc2	YPDnc3	YPDnc4	EtOHnc1	EtOHnc2	Galnc1	Galnc2
13	SPC42	YKL042W	YPDcl2	YPDcl3	EtOHcl1	EtOHcl2	Galcl1	YPDnc1	YPDnc2	YPDnc3	YPDnc4	EtOHnc1	EtOHnc2	Galnc1	Galnc2
13	FMP48	YGR052W	YPDcl2	YPDcl3	EtOHcl1	EtOHcl2	Galcl1	YPDnc1	YPDnc2	YPDnc3	YPDnc4	EtOHnc1	EtOHnc2	Galnc1	Galnc2
13		YDL196W	YPDcl2	YPDcl3	EtOHcl1	EtOHcl2	Galcl1	YPDnc1	YPDnc2	YPDnc3	YPDnc4	EtOHnc1	EtOHnc2	Galnc1	Galnc2
13	ORC2	YBR060C	YPDcl2	YPDcl3	EtOHcl1	EtOHcl2	Galcl1	YPDnc1	YPDnc2	YPDnc3	YPDnc4	EtOHnc1	EtOHnc2	Galnc1	Galnc2
13	COX15	YER141W	YPDcl2	YPDcl3	EtOHcl1	EtOHcl2	Galcl1	YPDnc1	YPDnc2	YPDnc3	YPDnc4	EtOHnc1	EtOHnc2	Galnc1	Galnc2
13		YPR172W	YPDcl2	YPDcl3	EtOHcl1	EtOHcl2	Galcl1	YPDnc1	YPDnc2	YPDnc3	YPDnc4	EtOHnc1	EtOHnc2	Galnc1	Galnc2
13	LSM1	YJL124C	YPDcl2	YPDcl3	EtOHcl1	EtOHcl2	Galcl1	YPDnc1	YPDnc2	YPDnc3	YPDnc4	EtOHnc1	EtOHnc2	Galnc1	Galnc2
13	NBP35	YGL091C	YPDcl2	YPDcl3	EtOHcl1	EtOHcl2	Galcl1	YPDnc1	YPDnc2	YPDnc3	YPDnc4	EtOHnc1	EtOHnc2	Galnc1	Galnc2
13	LSB6	YJL100W	YPDcl2	YPDcl3	EtOHcl1	EtOHcl2	Galcl1	YPDnc1	YPDnc2	YPDnc3	YPDnc4	EtOHnc1	EtOHnc2	Galnc1	Galnc2
13	AAP1	YHR047C	YPDcl2	YPDcl3	EtOHcl1	EtOHcl2	Galcl1	YPDnc1	YPDnc2	YPDnc3	YPDnc4	EtOHnc1	EtOHnc2	Galnc1	Galnc2
13	DAN1	YJR150C	YPDcl2	YPDcl3	EtOHcl1	EtOHcl2	Galcl1	YPDnc1	YPDnc2	YPDnc3	YPDnc4	EtOHnc1	EtOHnc2	Galnc1	Galnc2
13	FUN12	YAL035W	YPDcl2	YPDcl3	EtOHcl1	EtOHcl2	Galcl1	YPDnc1	YPDnc2	YPDnc3	YPDnc4	EtOHnc1	EtOHnc2	Galnc1	Galnc2
13	CMR3	YPR013C	YPDcl2	YPDcl3	EtOHcl1	EtOHcl2	Galcl1	YPDnc1	YPDnc2	YPDnc3	YPDnc4	EtOHnc1	EtOHnc2	Galnc1	Galnc2
13	PBI2	YNL015W	YPDcl2	YPDcl3	EtOHcl1	EtOHcl2	Galcl1	YPDnc1	YPDnc2	YPDnc3	YPDnc4	EtOHnc1	EtOHnc2	Galnc1	Galnc2
13	MRP49	YKL167C	YPDcl2	YPDcl3	EtOHcl1	EtOHcl2	Galcl1	YPDnc1	YPDnc2	YPDnc3	YPDnc4	EtOHnc1	EtOHnc2	Galnc1	Galnc2
13	MOT3	YMR070W	YPDcl2	YPDcl3	EtOHcl1	EtOHcl2	Galcl1	YPDnc1	YPDnc2	YPDnc3	YPDnc4	EtOHnc1	EtOHnc2	Galnc1	Galnc2
13		YML083C	YPDcl2	YPDcl3	EtOHcl1	EtOHcl2	Galcl1	YPDnc1	YPDnc2	YPDnc3	YPDnc4	EtOHnc1	EtOHnc2	Galnc1	Galnc2
13	MHR1	YDR296W	YPDcl2	YPDcl3	EtOHcl1	EtOHcl2	Galcl1	YPDnc1	YPDnc2	YPDnc3	YPDnc4	EtOHnc1	EtOHnc2	Galnc1	Galnc2
13	HMX1	YLR205C	YPDcl2	YPDcl3	EtOHcl1	EtOHcl2	Galcl1	YPDnc1	YPDnc2	YPDnc3	YPDnc4	EtOHnc1	EtOHnc2	Galnc1	Galnc2
13	SEC18	YBR080C	YPDcl2	YPDcl3	EtOHcl1	EtOHcl2	Galcl1	YPDnc1	YPDnc2	YPDnc3	YPDnc4	EtOHnc1	EtOHnc2	Galnc1	Galnc2
13	ADI1	YMR009W	YPDcl2	YPDcl3	EtOHcl1	EtOHcl2	Galcl1	YPDnc1	YPDnc2	YPDnc3	YPDnc4	EtOHnc1	EtOHnc2	Galnc1	Galnc2
13	PUS7	YOR243C	YPDcl2	YPDcl3	EtOHcl1	EtOHcl2	Galcl1	YPDnc1	YPDnc2	YPDnc3	YPDnc4	EtOHnc1	EtOHnc2	Galnc1	Galnc2
13		YBR235W	YPDcl2	YPDcl3	EtOHcl1	EtOHcl2	Galcl1	YPDnc1	YPDnc2	YPDnc3	YPDnc4	EtOHnc1	EtOHnc2	Galnc1	Galnc2
13	RIO2	YNL207W	YPDcl2	YPDcl3	EtOHcl1	EtOHcl2	Galcl1	YPDnc1	YPDnc2	YPDnc3	YPDnc4	EtOHnc1	EtOHnc2	Galnc1	Galnc2
13		YDR193W	YPDcl2	YPDcl3	EtOHcl1	EtOHcl2	Galcl1	YPDnc1	YPDnc2	YPDnc3	YPDnc4	EtOHnc1	EtOHnc2	Galnc1	Galnc2
13	SPB4	YFL002C	YPDcl2	YPDcl3	EtOHcl1	EtOHcl2	Galcl1	YPDnc1	YPDnc2	YPDnc3	YPDnc4	EtOHnc1	EtOHnc2	Galnc1	Galnc2

13	KAP123	YER110C	YPDcl2	YPDcl3	EtOHcl1	EtOHcl2	Galcl1	YPDnc1	YPDnc2	YPDnc3	YPDnc4	EtOHnc1	EtOHnc2	Galnc1	Galnc2
13	ERT1	YBR239C	YPDcl2	YPDcl3	EtOHcl1	EtOHcl2	Galcl1	YPDnc1	YPDnc2	YPDnc3	YPDnc4	EtOHnc1	EtOHnc2	Galnc1	Galnc2
13	YND1	YER005W	YPDcl2	YPDcl3	EtOHcl1	EtOHcl2	Galcl1	YPDnc1	YPDnc2	YPDnc3	YPDnc4	EtOHnc1	EtOHnc2	Galnc1	Galnc2
13	ECO1	YFR027W	YPDcl2	YPDcl3	EtOHcl1	EtOHcl2	Galcl1	YPDnc1	YPDnc2	YPDnc3	YPDnc4	EtOHnc1	EtOHnc2	Galnc1	Galnc2
13	ISM1	YPL040C	YPDcl2	YPDcl3	EtOHcl1	EtOHcl2	Galcl1	YPDnc1	YPDnc2	YPDnc3	YPDnc4	EtOHnc1	EtOHnc2	Galnc1	Galnc2
13		YJL064W	YPDcl2	YPDcl3	EtOHcl1	EtOHcl2	Galcl1	YPDnc1	YPDnc2	YPDnc3	YPDnc4	EtOHnc1	EtOHnc2	Galnc1	Galnc2
13	TRM12	YML005W	YPDcl2	YPDcl3	EtOHcl1	EtOHcl2	Galcl1	YPDnc1	YPDnc2	YPDnc3	YPDnc4	EtOHnc1	EtOHnc2	Galnc1	Galnc2
13		YGR283C	YPDcl2	YPDcl3	EtOHcl1	EtOHcl2	Galcl1	YPDnc1	YPDnc2	YPDnc3	YPDnc4	EtOHnc1	EtOHnc2	Galnc1	Galnc2
13	SDS3	YIL084C	YPDcl2	YPDcl3	EtOHcl1	EtOHcl2	Galcl1	YPDnc1	YPDnc2	YPDnc3	YPDnc4	EtOHnc1	EtOHnc2	Galnc1	Galnc2
13	AVT6	YER119C	YPDcl2	YPDcl3	EtOHcl1	EtOHcl2	Galcl1	YPDnc1	YPDnc2	YPDnc3	YPDnc4	EtOHnc1	EtOHnc2	Galnc1	Galnc2
13	AGX1	YFL030W	YPDcl2	YPDcl3	EtOHcl1	EtOHcl2	Galcl1	YPDnc1	YPDnc2	YPDnc3	YPDnc4	EtOHnc1	EtOHnc2	Galnc1	Galnc2
13	MRP2	YPR166C	YPDcl2	YPDcl3	EtOHcl1	EtOHcl2	Galcl1	YPDnc1	YPDnc2	YPDnc3	YPDnc4	EtOHnc1	EtOHnc2	Galnc1	Galnc2
13		YML108W	YPDcl2	YPDcl3	EtOHcl1	EtOHcl2	Galcl1	YPDnc1	YPDnc2	YPDnc3	YPDnc4	EtOHnc1	EtOHnc2	Galnc1	Galnc2
13	TIM11	YDR322C-A	YPDcl2	YPDcl3	EtOHcl1	EtOHcl2	Galcl1	YPDnc1	YPDnc2	YPDnc3	YPDnc4	EtOHnc1	EtOHnc2	Galnc1	Galnc2
13	CSM2	YIL132C	YPDcl2	YPDcl3	EtOHcl1	EtOHcl2	Galcl1	YPDnc1	YPDnc2	YPDnc3	YPDnc4	EtOHnc1	EtOHnc2	Galnc1	Galnc2
13	PSF1	YDR013W	YPDcl2	YPDcl3	EtOHcl1	EtOHcl2	Galcl1	YPDnc1	YPDnc2	YPDnc3	YPDnc4	EtOHnc1	EtOHnc2	Galnc1	Galnc2
13	DAK1	YML070W	YPDcl2	YPDcl3	EtOHcl1	EtOHcl2	Galcl1	YPDnc1	YPDnc2	YPDnc3	YPDnc4	EtOHnc1	EtOHnc2	Galnc1	Galnc2
13	CHL1	YPL008W	YPDcl2	YPDcl3	EtOHcl1	EtOHcl2	Galcl1	YPDnc1	YPDnc2	YPDnc3	YPDnc4	EtOHnc1	EtOHnc2	Galnc1	Galnc2
13	CMR1	YDL156W	YPDcl2	YPDcl3	EtOHcl1	EtOHcl2	Galcl1	YPDnc1	YPDnc2	YPDnc3	YPDnc4	EtOHnc1	EtOHnc2	Galnc1	Galnc2
13		YIR036W-A	YPDcl2	YPDcl3	EtOHcl1	EtOHcl2	Galcl1	YPDnc1	YPDnc2	YPDnc3	YPDnc4	EtOHnc1	EtOHnc2	Galnc1	Galnc2
13	1-Oct	YKL134C	YPDcl2	YPDcl3	EtOHcl1	EtOHcl2	Galcl1	YPDnc1	YPDnc2	YPDnc3	YPDnc4	EtOHnc1	EtOHnc2	Galnc1	Galnc2
13	RVB1	YDR190C	YPDcl2	YPDcl3	EtOHcl1	EtOHcl2	Galcl1	YPDnc1	YPDnc2	YPDnc3	YPDnc4	EtOHnc1	EtOHnc2	Galnc1	Galnc2
13		YDR109C	YPDcl2	YPDcl3	EtOHcl1	EtOHcl2	Galcl1	YPDnc1	YPDnc2	YPDnc3	YPDnc4	EtOHnc1	EtOHnc2	Galnc1	Galnc2
13		YLR356W	YPDcl2	YPDcl3	EtOHcl1	EtOHcl2	Galcl1	YPDnc1	YPDnc2	YPDnc3	YPDnc4	EtOHnc1	EtOHnc2	Galnc1	Galnc2
13	GSY1	YFR015C	YPDcl2	YPDcl3	EtOHcl1	EtOHcl2	Galcl1	YPDnc1	YPDnc2	YPDnc3	YPDnc4	EtOHnc1	EtOHnc2	Galnc1	Galnc2
13	ATG10	YLL042C	YPDcl2	YPDcl3	EtOHcl1	EtOHcl2	Galcl1	YPDnc1	YPDnc2	YPDnc3	YPDnc4	EtOHnc1	EtOHnc2	Galnc1	Galnc2
13	DIN7	YDR263C	YPDcl2	YPDcl3	EtOHcl1	EtOHcl2	Galcl1	YPDnc1	YPDnc2	YPDnc3	YPDnc4	EtOHnc1	EtOHnc2	Galnc1	Galnc2
13		YOL118C	YPDcl2	YPDcl3	EtOHcl1	EtOHcl2	Galcl1	YPDnc1	YPDnc2	YPDnc3	YPDnc4	EtOHnc1	EtOHnc2	Galnc1	Galnc2
13	TRR2	YHR106W	YPDcl2	YPDcl3	EtOHcl1	EtOHcl2	Galcl1	YPDnc1	YPDnc2	YPDnc3	YPDnc4	EtOHnc1	EtOHnc2	Galnc1	Galnc2
13		YGL118C	YPDcl2	YPDcl3	EtOHcl1	EtOHcl2	Galcl1	YPDnc1	YPDnc2	YPDnc3	YPDnc4	EtOHnc1	EtOHnc2	Galnc1	Galnc2
13	RPL38	YLR325C	YPDcl2	YPDcl3	EtOHcl1	EtOHcl2	Galcl1	YPDnc1	YPDnc2	YPDnc3	YPDnc4	EtOHnc1	EtOHnc2	Galnc1	Galnc2
13	MF(ALPHA	YGL089C	YPDcl2	YPDcl3	EtOHcl1	EtOHcl2	Galcl1	YPDnc1	YPDnc2	YPDnc3	YPDnc4	EtOHnc1	EtOHnc2	Galnc1	Galnc2
13	HFA1	YMR207C	YPDcl2	YPDcl3	EtOHcl1	EtOHcl2	Galcl1	YPDnc1	YPDnc2	YPDnc3	YPDnc4	EtOHnc1	EtOHnc2	Galnc1	Galnc2
13	RPA49	YNL248C	YPDcl2	YPDcl3	EtOHcl1	EtOHcl2	Galcl1	YPDnc1	YPDnc2	YPDnc3	YPDnc4	EtOHnc1	EtOHnc2	Galnc1	Galnc2

13	PET123	YOR158W	YPDcl2	YPDcl3	EtOHcl1	EtOHcl2	Galcl1	YPDnc1	YPDnc2	YPDnc3	YPDnc4	EtOHnc1	EtOHnc2	Galnc1	Galnc2
13	SIS1	YNL007C	YPDcl2	YPDcl3	EtOHcl1	EtOHcl2	Galcl1	YPDnc1	YPDnc2	YPDnc3	YPDnc4	EtOHnc1	EtOHnc2	Galnc1	Galnc2
13	PRM6	YML047C	YPDcl2	YPDcl3	EtOHcl1	EtOHcl2	Galcl1	YPDnc1	YPDnc2	YPDnc3	YPDnc4	EtOHnc1	EtOHnc2	Galnc1	Galnc2
13	CLC1	YGR167W	YPDcl2	YPDcl3	EtOHcl1	EtOHcl2	Galcl1	YPDnc1	YPDnc2	YPDnc3	YPDnc4	EtOHnc1	EtOHnc2	Galnc1	Galnc2
13		YJL182C	YPDcl2	YPDcl3	EtOHcl1	EtOHcl2	Galcl1	YPDnc1	YPDnc2	YPDnc3	YPDnc4	EtOHnc1	EtOHnc2	Galnc1	Galnc2
13	GAT2	YMR136W	YPDcl2	YPDcl3	EtOHcl1	EtOHcl2	Galcl1	YPDnc1	YPDnc2	YPDnc3	YPDnc4	EtOHnc1	EtOHnc2	Galnc1	Galnc2
13		YCR076C	YPDcl2	YPDcl3	EtOHcl1	EtOHcl2	Galcl1	YPDnc1	YPDnc2	YPDnc3	YPDnc4	EtOHnc1	EtOHnc2	Galnc1	Galnc2
13	COG1	YGL223C	YPDcl2	YPDcl3	EtOHcl1	EtOHcl2	Galcl1	YPDnc1	YPDnc2	YPDnc3	YPDnc4	EtOHnc1	EtOHnc2	Galnc1	Galnc2
13		YHR131C	YPDcl2	YPDcl3	EtOHcl1	EtOHcl2	Galcl1	YPDnc1	YPDnc2	YPDnc3	YPDnc4	EtOHnc1	EtOHnc2	Galnc1	Galnc2
13	POT1	YIL160C	YPDcl2	YPDcl3	EtOHcl1	EtOHcl2	Galcl1	YPDnc1	YPDnc2	YPDnc3	YPDnc4	EtOHnc1	EtOHnc2	Galnc1	Galnc2
13	ENA2	YDR039C	YPDcl2	YPDcl3	EtOHcl1	EtOHcl2	Galcl1	YPDnc1	YPDnc2	YPDnc3	YPDnc4	EtOHnc1	EtOHnc2	Galnc1	Galnc2
13	RFT1	YBL020W	YPDcl2	YPDcl3	EtOHcl1	EtOHcl2	Galcl1	YPDnc1	YPDnc2	YPDnc3	YPDnc4	EtOHnc1	EtOHnc2	Galnc1	Galnc2
13	VMA2	YBR127C	YPDcl2	YPDcl3	EtOHcl1	EtOHcl2	Galcl1	YPDnc1	YPDnc2	YPDnc3	YPDnc4	EtOHnc1	EtOHnc2	Galnc1	Galnc2
13	RSM23	YGL129C	YPDcl2	YPDcl3	EtOHcl1	EtOHcl2	Galcl1	YPDnc1	YPDnc2	YPDnc3	YPDnc4	EtOHnc1	EtOHnc2	Galnc1	Galnc2
13	LST4	YKL176C	YPDcl2	YPDcl3	EtOHcl1	EtOHcl2	Galcl1	YPDnc1	YPDnc2	YPDnc3	YPDnc4	EtOHnc1	EtOHnc2	Galnc1	Galnc2
13		YER152W-A	YPDcl2	YPDcl3	EtOHcl1	EtOHcl2	Galcl1	YPDnc1	YPDnc2	YPDnc3	YPDnc4	EtOHnc1	EtOHnc2	Galnc1	Galnc2
13	KAP104	YBR017C	YPDcl2	YPDcl3	EtOHcl1	EtOHcl2	Galcl1	YPDnc1	YPDnc2	YPDnc3	YPDnc4	EtOHnc1	EtOHnc2	Galnc1	Galnc2
13	STB2	YMR053C	YPDcl2	YPDcl3	EtOHcl1	EtOHcl2	Galcl1	YPDnc1	YPDnc2	YPDnc3	YPDnc4	EtOHnc1	EtOHnc2	Galnc1	Galnc2
13	SPE4	YLR146C	YPDcl2	YPDcl3	EtOHcl1	EtOHcl2	Galcl1	YPDnc1	YPDnc2	YPDnc3	YPDnc4	EtOHnc1	EtOHnc2	Galnc1	Galnc2
13	MRP7	YNL005C	YPDcl2	YPDcl3	EtOHcl1	EtOHcl2	Galcl1	YPDnc1	YPDnc2	YPDnc3	YPDnc4	EtOHnc1	EtOHnc2	Galnc1	Galnc2
13		YHL039W	YPDcl2	YPDcl3	EtOHcl1	EtOHcl2	Galcl1	YPDnc1	YPDnc2	YPDnc3	YPDnc4	EtOHnc1	EtOHnc2	Galnc1	Galnc2
13	CDC9	YDL164C	YPDcl2	YPDcl3	EtOHcl1	EtOHcl2	Galcl1	YPDnc1	YPDnc2	YPDnc3	YPDnc4	EtOHnc1	EtOHnc2	Galnc1	Galnc2
13	SLG1	YOR008C	YPDcl2	YPDcl3	EtOHcl1	EtOHcl2	Galcl1	YPDnc1	YPDnc2	YPDnc3	YPDnc4	EtOHnc1	EtOHnc2	Galnc1	Galnc2
13	SHE9	YDR393W	YPDcl2	YPDcl3	EtOHcl1	EtOHcl2	Galcl1	YPDnc1	YPDnc2	YPDnc3	YPDnc4	EtOHnc1	EtOHnc2	Galnc1	Galnc2
13		YEL050W-A	YPDcl2	YPDcl3	EtOHcl1	EtOHcl2	Galcl1	YPDnc1	YPDnc2	YPDnc3	YPDnc4	EtOHnc1	EtOHnc2	Galnc1	Galnc2
13	VAM10	YOR068C	YPDcl2	YPDcl3	EtOHcl1	EtOHcl2	Galcl1	YPDnc1	YPDnc2	YPDnc3	YPDnc4	EtOHnc1	EtOHnc2	Galnc1	Galnc2
13	MDN1	YLR106C	YPDcl2	YPDcl3	EtOHcl1	EtOHcl2	Galcl1	YPDnc1	YPDnc2	YPDnc3	YPDnc4	EtOHnc1	EtOHnc2	Galnc1	Galnc2
13		YEL025C	YPDcl2	YPDcl3	EtOHcl1	EtOHcl2	Galcl1	YPDnc1	YPDnc2	YPDnc3	YPDnc4	EtOHnc1	EtOHnc2	Galnc1	Galnc2
13	PES4	YFR023W	YPDcl2	YPDcl3	EtOHcl1	EtOHcl2	Galcl1	YPDnc1	YPDnc2	YPDnc3	YPDnc4	EtOHnc1	EtOHnc2	Galnc1	Galnc2
13	GIS2	YNL255C	YPDcl2	YPDcl3	EtOHcl1	EtOHcl2	Galcl1	YPDnc1	YPDnc2	YPDnc3	YPDnc4	EtOHnc1	EtOHnc2	Galnc1	Galnc2
13	MBB1	YJL199C	YPDcl2	YPDcl3	EtOHcl1	EtOHcl2	Galcl1	YPDnc1	YPDnc2	YPDnc3	YPDnc4	EtOHnc1	EtOHnc2	Galnc1	Galnc2
13	MYO3	YKL129C	YPDcl2	YPDcl3	EtOHcl1	EtOHcl2	Galcl1	YPDnc1	YPDnc2	YPDnc3	YPDnc4	EtOHnc1	EtOHnc2	Galnc1	Galnc2
13	RBK1	YCR036W	YPDcl2	YPDcl3	EtOHcl1	EtOHcl2	Galcl1	YPDnc1	YPDnc2	YPDnc3	YPDnc4	EtOHnc1	EtOHnc2	Galnc1	Galnc2
13	ATX1	YNL259C	YPDcl2	YPDcl3	EtOHcl1	EtOHcl2	Galcl1	YPDnc1	YPDnc2	YPDnc3	YPDnc4	EtOHnc1	EtOHnc2	Galnc1	Galnc2

13	OMA1	YKR087C	YPDcl2	YPDcl3	EtOHcl1	EtOHcl2	Galcl1	YPDnc1	YPDnc2	YPDnc3	YPDnc4	EtOHnc1	EtOHnc2	Galnc1	Galnc2
13	ARG3	YJL088W	YPDcl2	YPDcl3	EtOHcl1	EtOHcl2	Galcl1	YPDnc1	YPDnc2	YPDnc3	YPDnc4	EtOHnc1	EtOHnc2	Galnc1	Galnc2
13		YCR087W	YPDcl2	YPDcl3	EtOHcl1	EtOHcl2	Galcl1	YPDnc1	YPDnc2	YPDnc3	YPDnc4	EtOHnc1	EtOHnc2	Galnc1	Galnc2
13		YDL114W-A	YPDcl2	YPDcl3	EtOHcl1	EtOHcl2	Galcl1	YPDnc1	YPDnc2	YPDnc3	YPDnc4	EtOHnc1	EtOHnc2	Galnc1	Galnc2
13	DAT1	YML113W	YPDcl2	YPDcl3	EtOHcl1	EtOHcl2	Galcl1	YPDnc1	YPDnc2	YPDnc3	YPDnc4	EtOHnc1	EtOHnc2	Galnc1	Galnc2
13	RNQ1	YCL028W	YPDcl2	YPDcl3	EtOHcl1	EtOHcl2	Galcl1	YPDnc1	YPDnc2	YPDnc3	YPDnc4	EtOHnc1	EtOHnc2	Galnc1	Galnc2
13	CIR2	YOR356W	YPDcl2	YPDcl3	EtOHcl1	EtOHcl2	Galcl1	YPDnc1	YPDnc2	YPDnc3	YPDnc4	EtOHnc1	EtOHnc2	Galnc1	Galnc2
13	CBF5	YLR175W	YPDcl2	YPDcl3	EtOHcl1	EtOHcl2	Galcl1	YPDnc1	YPDnc2	YPDnc3	YPDnc4	EtOHnc1	EtOHnc2	Galnc1	Galnc2
13	RAD14	YMR201C	YPDcl2	YPDcl3	EtOHcl1	EtOHcl2	Galcl1	YPDnc1	YPDnc2	YPDnc3	YPDnc4	EtOHnc1	EtOHnc2	Galnc1	Galnc2
13	MET4	YNL103W	YPDcl2	YPDcl3	EtOHcl1	EtOHcl2	Galcl1	YPDnc1	YPDnc2	YPDnc3	YPDnc4	EtOHnc1	EtOHnc2	Galnc1	Galnc2
13		YNL285W	YPDcl2	YPDcl3	EtOHcl1	EtOHcl2	Galcl1	YPDnc1	YPDnc2	YPDnc3	YPDnc4	EtOHnc1	EtOHnc2	Galnc1	Galnc2
13	SRO9	YCL037C	YPDcl2	YPDcl3	EtOHcl1	EtOHcl2	Galcl1	YPDnc1	YPDnc2	YPDnc3	YPDnc4	EtOHnc1	EtOHnc2	Galnc1	Galnc2
13	HOS3	YPL116W	YPDcl2	YPDcl3	EtOHcl1	EtOHcl2	Galcl1	YPDnc1	YPDnc2	YPDnc3	YPDnc4	EtOHnc1	EtOHnc2	Galnc1	Galnc2
13	ROT2	YBR229C	YPDcl2	YPDcl3	EtOHcl1	EtOHcl2	Galcl1	YPDnc1	YPDnc2	YPDnc3	YPDnc4	EtOHnc1	EtOHnc2	Galnc1	Galnc2
13	ISW2	YOR304W	YPDcl2	YPDcl3	EtOHcl1	EtOHcl2	Galcl1	YPDnc1	YPDnc2	YPDnc3	YPDnc4	EtOHnc1	EtOHnc2	Galnc1	Galnc2
13	TOM71	YHR117W	YPDcl2	YPDcl3	EtOHcl1	EtOHcl2	Galcl1	YPDnc1	YPDnc2	YPDnc3	YPDnc4	EtOHnc1	EtOHnc2	Galnc1	Galnc2
13		YOR366W	YPDcl2	YPDcl3	EtOHcl1	EtOHcl2	Galcl1	YPDnc1	YPDnc2	YPDnc3	YPDnc4	EtOHnc1	EtOHnc2	Galnc1	Galnc2
13	DSD1	YGL196W	YPDcl2	YPDcl3	EtOHcl1	EtOHcl2	Galcl1	YPDnc1	YPDnc2	YPDnc3	YPDnc4	EtOHnc1	EtOHnc2	Galnc1	Galnc2
13	MIG1	YGL035C	YPDcl2	YPDcl3	EtOHcl1	EtOHcl2	Galcl1	YPDnc1	YPDnc2	YPDnc3	YPDnc4	EtOHnc1	EtOHnc2	Galnc1	Galnc2
13	LEO1	YOR123C	YPDcl2	YPDcl3	EtOHcl1	EtOHcl2	Galcl1	YPDnc1	YPDnc2	YPDnc3	YPDnc4	EtOHnc1	EtOHnc2	Galnc1	Galnc2
13	TAH18	YPR048W	YPDcl2	YPDcl3	EtOHcl1	EtOHcl2	Galcl1	YPDnc1	YPDnc2	YPDnc3	YPDnc4	EtOHnc1	EtOHnc2	Galnc1	Galnc2
13		YPL109C	YPDcl2	YPDcl3	EtOHcl1	EtOHcl2	Galcl1	YPDnc1	YPDnc2	YPDnc3	YPDnc4	EtOHnc1	EtOHnc2	Galnc1	Galnc2
13	SPP1	YPL138C	YPDcl2	YPDcl3	EtOHcl1	EtOHcl2	Galcl1	YPDnc1	YPDnc2	YPDnc3	YPDnc4	EtOHnc1	EtOHnc2	Galnc1	Galnc2
13	SBA1	YKL117W	YPDcl2	YPDcl3	EtOHcl1	EtOHcl2	Galcl1	YPDnc1	YPDnc2	YPDnc3	YPDnc4	EtOHnc1	EtOHnc2	Galnc1	Galnc2
13	TRM13	YOL125W	YPDcl2	YPDcl3	EtOHcl1	EtOHcl2	Galcl1	YPDnc1	YPDnc2	YPDnc3	YPDnc4	EtOHnc1	EtOHnc2	Galnc1	Galnc2
13	CUP5	YEL027W	YPDcl2	YPDcl3	EtOHcl1	EtOHcl2	Galcl1	YPDnc1	YPDnc2	YPDnc3	YPDnc4	EtOHnc1	EtOHnc2	Galnc1	Galnc2
13	HAL5	YJL165C	YPDcl2	YPDcl3	EtOHcl1	EtOHcl2	Galcl1	YPDnc1	YPDnc2	YPDnc3	YPDnc4	EtOHnc1	EtOHnc2	Galnc1	Galnc2
13		YOR300W	YPDcl2	YPDcl3	EtOHcl1	EtOHcl2	Galcl1	YPDnc1	YPDnc2	YPDnc3	YPDnc4	EtOHnc1	EtOHnc2	Galnc1	Galnc2
13	MIG2	YGL209W	YPDcl2	YPDcl3	EtOHcl1	EtOHcl2	Galcl1	YPDnc1	YPDnc2	YPDnc3	YPDnc4	EtOHnc1	EtOHnc2	Galnc1	Galnc2
13	RPL32	YBL092W	YPDcl2	YPDcl3	EtOHcl1	EtOHcl2	Galcl1	YPDnc1	YPDnc2	YPDnc3	YPDnc4	EtOHnc1	EtOHnc2	Galnc1	Galnc2
13	ADH5	YBR145W	YPDcl2	YPDcl3	EtOHcl1	EtOHcl2	Galcl1	YPDnc1	YPDnc2	YPDnc3	YPDnc4	EtOHnc1	EtOHnc2	Galnc1	Galnc2
13	BUD6	YLR319C	YPDcl2	YPDcl3	EtOHcl1	EtOHcl2	Galcl1	YPDnc1	YPDnc2	YPDnc3	YPDnc4	EtOHnc1	EtOHnc2	Galnc1	Galnc2
13	PBP2	YBR233W	YPDcl2	YPDcl3	EtOHcl1	EtOHcl2	Galcl1	YPDnc1	YPDnc2	YPDnc3	YPDnc4	EtOHnc1	EtOHnc2	Galnc1	Galnc2
13	RPC11	YDR045C	YPDcl2	YPDcl3	EtOHcl1	EtOHcl2	Galcl1	YPDnc1	YPDnc2	YPDnc3	YPDnc4	EtOHnc1	EtOHnc2	Galnc1	Galnc2

13	ERP5	YHR110W	YPDcl2	YPDcl3	EtOHcl1	EtOHcl2	Galcl1	YPDnc1	YPDnc2	YPDnc3	YPDnc4	EtOHnc1	EtOHnc2	Galnc1	Galnc2
13	MET14	YKL001C	YPDcl2	YPDcl3	EtOHcl1	EtOHcl2	Galcl1	YPDnc1	YPDnc2	YPDnc3	YPDnc4	EtOHnc1	EtOHnc2	Galnc1	Galnc2
13	CHC1	YGL206C	YPDcl2	YPDcl3	EtOHcl1	EtOHcl2	Galcl1	YPDnc1	YPDnc2	YPDnc3	YPDnc4	EtOHnc1	EtOHnc2	Galnc1	Galnc2
13	GPX2	YBR244W	YPDcl2	YPDcl3	EtOHcl1	EtOHcl2	Galcl1	YPDnc1	YPDnc2	YPDnc3	YPDnc4	EtOHnc1	EtOHnc2	Galnc1	Galnc2
13	STE2	YFL026W	YPDcl2	YPDcl3	EtOHcl1	EtOHcl2	Galcl1	YPDnc1	YPDnc2	YPDnc3	YPDnc4	EtOHnc1	EtOHnc2	Galnc1	Galnc2
13		YLR236C	YPDcl2	YPDcl3	EtOHcl1	EtOHcl2	Galcl1	YPDnc1	YPDnc2	YPDnc3	YPDnc4	EtOHnc1	EtOHnc2	Galnc1	Galnc2
13	BNI5	YNL166C	YPDcl2	YPDcl3	EtOHcl1	EtOHcl2	Galcl1	YPDnc1	YPDnc2	YPDnc3	YPDnc4	EtOHnc1	EtOHnc2	Galnc1	Galnc2
13	INP2	YMR163C	YPDcl2	YPDcl3	EtOHcl1	EtOHcl2	Galcl1	YPDnc1	YPDnc2	YPDnc3	YPDnc4	EtOHnc1	EtOHnc2	Galnc1	Galnc2
13	PDR1	YGL013C	YPDcl2	YPDcl3	EtOHcl1	EtOHcl2	Galcl1	YPDnc1	YPDnc2	YPDnc3	YPDnc4	EtOHnc1	EtOHnc2	Galnc1	Galnc2
13	PIH1	YHR034C	YPDcl2	YPDcl3	EtOHcl1	EtOHcl2	Galcl1	YPDnc1	YPDnc2	YPDnc3	YPDnc4	EtOHnc1	EtOHnc2	Galnc1	Galnc2
13		YOR008C-A	YPDcl2	YPDcl3	EtOHcl1	EtOHcl2	Galcl1	YPDnc1	YPDnc2	YPDnc3	YPDnc4	EtOHnc1	EtOHnc2	Galnc1	Galnc2
13		YFL041W-A	YPDcl2	YPDcl3	EtOHcl1	EtOHcl2	Galcl1	YPDnc1	YPDnc2	YPDnc3	YPDnc4	EtOHnc1	EtOHnc2	Galnc1	Galnc2
13	GIS4	YML006C	YPDcl2	YPDcl3	EtOHcl1	EtOHcl2	Galcl1	YPDnc1	YPDnc2	YPDnc3	YPDnc4	EtOHnc1	EtOHnc2	Galnc1	Galnc2
13	BNS1	YGR230W	YPDcl2	YPDcl3	EtOHcl1	EtOHcl2	Galcl1	YPDnc1	YPDnc2	YPDnc3	YPDnc4	EtOHnc1	EtOHnc2	Galnc1	Galnc2
13		YIL066W-A	YPDcl2	YPDcl3	EtOHcl1	EtOHcl2	Galcl1	YPDnc1	YPDnc2	YPDnc3	YPDnc4	EtOHnc1	EtOHnc2	Galnc1	Galnc2
13		YKR017C	YPDcl2	YPDcl3	EtOHcl1	EtOHcl2	Galcl1	YPDnc1	YPDnc2	YPDnc3	YPDnc4	EtOHnc1	EtOHnc2	Galnc1	Galnc2
13	TAF2	YCR042C	YPDcl2	YPDcl3	EtOHcl1	EtOHcl2	Galcl1	YPDnc1	YPDnc2	YPDnc3	YPDnc4	EtOHnc1	EtOHnc2	Galnc1	Galnc2
13	EAF6	YJR082C	YPDcl2	YPDcl3	EtOHcl1	EtOHcl2	Galcl1	YPDnc1	YPDnc2	YPDnc3	YPDnc4	EtOHnc1	EtOHnc2	Galnc1	Galnc2
13	LEE1	YPL054W	YPDcl2	YPDcl3	EtOHcl1	EtOHcl2	Galcl1	YPDnc1	YPDnc2	YPDnc3	YPDnc4	EtOHnc1	EtOHnc2	Galnc1	Galnc2
13	AEP2	YMR282C	YPDcl2	YPDcl3	EtOHcl1	EtOHcl2	Galcl1	YPDnc1	YPDnc2	YPDnc3	YPDnc4	EtOHnc1	EtOHnc2	Galnc1	Galnc2
13	CIR1	YGR207C	YPDcl2	YPDcl3	EtOHcl1	EtOHcl2	Galcl1	YPDnc1	YPDnc2	YPDnc3	YPDnc4	EtOHnc1	EtOHnc2	Galnc1	Galnc2
13		YOR225W	YPDcl2	YPDcl3	EtOHcl1	EtOHcl2	Galcl1	YPDnc1	YPDnc2	YPDnc3	YPDnc4	EtOHnc1	EtOHnc2	Galnc1	Galnc2
13		YJL062W-A	YPDcl2	YPDcl3	EtOHcl1	EtOHcl2	Galcl1	YPDnc1	YPDnc2	YPDnc3	YPDnc4	EtOHnc1	EtOHnc2	Galnc1	Galnc2
13	CUE5	YOR042W	YPDcl2	YPDcl3	EtOHcl1	EtOHcl2	Galcl1	YPDnc1	YPDnc2	YPDnc3	YPDnc4	EtOHnc1	EtOHnc2	Galnc1	Galnc2
13		YHR131W-	YPDcl2	YPDcl3	EtOHcl1	EtOHcl2	Galcl1	YPDnc1	YPDnc2	YPDnc3	YPDnc4	EtOHnc1	EtOHnc2	Galnc1	Galnc2
13	GUD1	YDL238C	YPDcl2	YPDcl3	EtOHcl1	EtOHcl2	Galcl1	YPDnc1	YPDnc2	YPDnc3	YPDnc4	EtOHnc1	EtOHnc2	Galnc1	Galnc2
13	PFD1	YJL179W	YPDcl2	YPDcl3	EtOHcl1	EtOHcl2	Galcl1	YPDnc1	YPDnc2	YPDnc3	YPDnc4	EtOHnc1	EtOHnc2	Galnc1	Galnc2
13		YJR111C	YPDcl2	YPDcl3	EtOHcl1	EtOHcl2	Galcl1	YPDnc1	YPDnc2	YPDnc3	YPDnc4	EtOHnc1	EtOHnc2	Galnc1	Galnc2
13	ABP1	YCR088W	YPDcl2	YPDcl3	EtOHcl1	EtOHcl2	Galcl1	YPDnc1	YPDnc2	YPDnc3	YPDnc4	EtOHnc1	EtOHnc2	Galnc1	Galnc2
13	DAL2	YIR029W	YPDcl2	YPDcl3	EtOHcl1	EtOHcl2	Galcl1	YPDnc1	YPDnc2	YPDnc3	YPDnc4	EtOHnc1	EtOHnc2	Galnc1	Galnc2
13	TRM10	YOL093W	YPDcl2	YPDcl3	EtOHcl1	EtOHcl2	Galcl1	YPDnc1	YPDnc2	YPDnc3	YPDnc4	EtOHnc1	EtOHnc2	Galnc1	Galnc2
13		YJL144W	YPDcl2	YPDcl3	EtOHcl1	EtOHcl2	Galcl1	YPDnc1	YPDnc2	YPDnc3	YPDnc4	EtOHnc1	EtOHnc2	Galnc1	Galnc2
13	NUP159	YIL115C	YPDcl2	YPDcl3	EtOHcl1	EtOHcl2	Galcl1	YPDnc1	YPDnc2	YPDnc3	YPDnc4	EtOHnc1	EtOHnc2	Galnc1	Galnc2
13	CTF13	YMR094W	YPDcl2	YPDcl3	EtOHcl1	EtOHcl2	Galcl1	YPDnc1	YPDnc2	YPDnc3	YPDnc4	EtOHnc1	EtOHnc2	Galnc1	Galnc2

13		YLR217W	YPDcl2	YPDcl3	EtOHcl1	EtOHcl2	Galcl1	YPDnc1	YPDnc2	YPDnc3	YPDnc4	EtOHnc1	EtOHnc2	Galnc1	Galnc2
13	PML39	YML107C	YPDcl2	YPDcl3	EtOHcl1	EtOHcl2	Galcl1	YPDnc1	YPDnc2	YPDnc3	YPDnc4	EtOHnc1	EtOHnc2	Galnc1	Galnc2
13	SYG1	YIL047C	YPDcl2	YPDcl3	EtOHcl1	EtOHcl2	Galcl1	YPDnc1	YPDnc2	YPDnc3	YPDnc4	EtOHnc1	EtOHnc2	Galnc1	Galnc2
13		YHR130C	YPDcl2	YPDcl3	EtOHcl1	EtOHcl2	Galcl1	YPDnc1	YPDnc2	YPDnc3	YPDnc4	EtOHnc1	EtOHnc2	Galnc1	Galnc2
13	MLS1	YNL117W	YPDcl2	YPDcl3	EtOHcl1	EtOHcl2	Galcl1	YPDnc1	YPDnc2	YPDnc3	YPDnc4	EtOHnc1	EtOHnc2	Galnc1	Galnc2
13	PHO4	YFR034C	YPDcl2	YPDcl3	EtOHcl1	EtOHcl2	Galcl1	YPDnc1	YPDnc2	YPDnc3	YPDnc4	EtOHnc1	EtOHnc2	Galnc1	Galnc2
13		YDL172C	YPDcl2	YPDcl3	EtOHcl1	EtOHcl2	Galcl1	YPDnc1	YPDnc2	YPDnc3	YPDnc4	EtOHnc1	EtOHnc2	Galnc1	Galnc2
13		YPL066W	YPDcl2	YPDcl3	EtOHcl1	EtOHcl2	Galcl1	YPDnc1	YPDnc2	YPDnc3	YPDnc4	EtOHnc1	EtOHnc2	Galnc1	Galnc2
13	ALK1	YGL021W	YPDcl2	YPDcl3	EtOHcl1	EtOHcl2	Galcl1	YPDnc1	YPDnc2	YPDnc3	YPDnc4	EtOHnc1	EtOHnc2	Galnc1	Galnc2
13		YAL034C-B	YPDcl2	YPDcl3	EtOHcl1	EtOHcl2	Galcl1	YPDnc1	YPDnc2	YPDnc3	YPDnc4	EtOHnc1	EtOHnc2	Galnc1	Galnc2
13	YAK1	YJL141C	YPDcl2	YPDcl3	EtOHcl1	EtOHcl2	Galcl1	YPDnc1	YPDnc2	YPDnc3	YPDnc4	EtOHnc1	EtOHnc2	Galnc1	Galnc2
13	INP53	YOR109W	YPDcl2	YPDcl3	EtOHcl1	EtOHcl2	Galcl1	YPDnc1	YPDnc2	YPDnc3	YPDnc4	EtOHnc1	EtOHnc2	Galnc1	Galnc2
13	SDS22	YKL193C	YPDcl2	YPDcl3	EtOHcl1	EtOHcl2	Galcl1	YPDnc1	YPDnc2	YPDnc3	YPDnc4	EtOHnc1	EtOHnc2	Galnc1	Galnc2
13	PXL1	YKR090W	YPDcl2	YPDcl3	EtOHcl1	EtOHcl2	Galcl1	YPDnc1	YPDnc2	YPDnc3	YPDnc4	EtOHnc1	EtOHnc2	Galnc1	Galnc2
13	IML3	YBR107C	YPDcl2	YPDcl3	EtOHcl1	EtOHcl2	Galcl1	YPDnc1	YPDnc2	YPDnc3	YPDnc4	EtOHnc1	EtOHnc2	Galnc1	Galnc2
13	YRR1	YOR162C	YPDcl2	YPDcl3	EtOHcl1	EtOHcl2	Galcl1	YPDnc1	YPDnc2	YPDnc3	YPDnc4	EtOHnc1	EtOHnc2	Galnc1	Galnc2
13	NTG2	YOL043C	YPDcl2	YPDcl3	EtOHcl1	EtOHcl2	Galcl1	YPDnc1	YPDnc2	YPDnc3	YPDnc4	EtOHnc1	EtOHnc2	Galnc1	Galnc2
13	ARO4	YBR249C	YPDcl2	YPDcl3	EtOHcl1	EtOHcl2	Galcl1	YPDnc1	YPDnc2	YPDnc3	YPDnc4	EtOHnc1	EtOHnc2	Galnc1	Galnc2
13	SPO12	YHR152W	YPDcl2	YPDcl3	EtOHcl1	EtOHcl2	Galcl1	YPDnc1	YPDnc2	YPDnc3	YPDnc4	EtOHnc1	EtOHnc2	Galnc1	Galnc2
13	FET5	YFL041W	YPDcl2	YPDcl3	EtOHcl1	EtOHcl2	Galcl1	YPDnc1	YPDnc2	YPDnc3	YPDnc4	EtOHnc1	EtOHnc2	Galnc1	Galnc2
13	ATF1	YOR377W	YPDcl2	YPDcl3	EtOHcl1	EtOHcl2	Galcl1	YPDnc1	YPDnc2	YPDnc3	YPDnc4	EtOHnc1	EtOHnc2	Galnc1	Galnc2
13	FAT1	YBR041W	YPDcl2	YPDcl3	EtOHcl1	EtOHcl2	Galcl1	YPDnc1	YPDnc2	YPDnc3	YPDnc4	EtOHnc1	EtOHnc2	Galnc1	Galnc2
13		YNL028W	YPDcl2	YPDcl3	EtOHcl1	EtOHcl2	Galcl1	YPDnc1	YPDnc2	YPDnc3	YPDnc4	EtOHnc1	EtOHnc2	Galnc1	Galnc2
13	PGM3	YMR278W	YPDcl2	YPDcl3	EtOHcl1	EtOHcl2	Galcl1	YPDnc1	YPDnc2	YPDnc3	YPDnc4	EtOHnc1	EtOHnc2	Galnc1	Galnc2
13	NRD1	YNL251C	YPDcl2	YPDcl3	EtOHcl1	EtOHcl2	Galcl1	YPDnc1	YPDnc2	YPDnc3	YPDnc4	EtOHnc1	EtOHnc2	Galnc1	Galnc2
13		YMR310C	YPDcl2	YPDcl3	EtOHcl1	EtOHcl2	Galcl1	YPDnc1	YPDnc2	YPDnc3	YPDnc4	EtOHnc1	EtOHnc2	Galnc1	Galnc2
13		YNL058C	YPDcl2	YPDcl3	EtOHcl1	EtOHcl2	Galcl1	YPDnc1	YPDnc2	YPDnc3	YPDnc4	EtOHnc1	EtOHnc2	Galnc1	Galnc2
13	RTT105	YER104W	YPDcl2	YPDcl3	EtOHcl1	EtOHcl2	Galcl1	YPDnc1	YPDnc2	YPDnc3	YPDnc4	EtOHnc1	EtOHnc2	Galnc1	Galnc2
13	GPI18	YBR004C	YPDcl2	YPDcl3	EtOHcl1	EtOHcl2	Galcl1	YPDnc1	YPDnc2	YPDnc3	YPDnc4	EtOHnc1	EtOHnc2	Galnc1	Galnc2
13	FUM1	YPL262W	YPDcl2	YPDcl3	EtOHcl1	EtOHcl2	Galcl1	YPDnc1	YPDnc2	YPDnc3	YPDnc4	EtOHnc1	EtOHnc2	Galnc1	Galnc2
13		YIR020C-B	YPDcl2	YPDcl3	EtOHcl1	EtOHcl2	Galcl1	YPDnc1	YPDnc2	YPDnc3	YPDnc4	EtOHnc1	EtOHnc2	Galnc1	Galnc2
13	BOP2	YLR267W	YPDcl2	YPDcl3	EtOHcl1	EtOHcl2	Galcl1	YPDnc1	YPDnc2	YPDnc3	YPDnc4	EtOHnc1	EtOHnc2	Galnc1	Galnc2
13	SPO71	YDR104C	YPDcl2	YPDcl3	EtOHcl1	EtOHcl2	Galcl1	YPDnc1	YPDnc2	YPDnc3	YPDnc4	EtOHnc1	EtOHnc2	Galnc1	Galnc2
13		YKL106C-A	YPDcl2	YPDcl3	EtOHcl1	EtOHcl2	Galcl1	YPDnc1	YPDnc2	YPDnc3	YPDnc4	EtOHnc1	EtOHnc2	Galnc1	Galnc2

13	RAD18	YCR066W	YPDcl2	YPDcl3	EtOHcl1	EtOHcl2	Galcl1	YPDnc1	YPDnc2	YPDnc3	YPDnc4	EtOHnc1	EtOHnc2	Galnc1	Galnc2
13		YBL068W-A	YPDcl2	YPDcl3	EtOHcl1	EtOHcl2	Galcl1	YPDnc1	YPDnc2	YPDnc3	YPDnc4	EtOHnc1	EtOHnc2	Galnc1	Galnc2
13	SWD3	YBR175W	YPDcl2	YPDcl3	EtOHcl1	EtOHcl2	Galcl1	YPDnc1	YPDnc2	YPDnc3	YPDnc4	EtOHnc1	EtOHnc2	Galnc1	Galnc2
13	GPI1	YGR216C	YPDcl2	YPDcl3	EtOHcl1	EtOHcl2	Galcl1	YPDnc1	YPDnc2	YPDnc3	YPDnc4	EtOHnc1	EtOHnc2	Galnc1	Galnc2
13	VID22	YLR373C	YPDcl2	YPDcl3	EtOHcl1	EtOHcl2	Galcl1	YPDnc1	YPDnc2	YPDnc3	YPDnc4	EtOHnc1	EtOHnc2	Galnc1	Galnc2
13	AFG1	YEL052W	YPDcl2	YPDcl3	EtOHcl1	EtOHcl2	Galcl1	YPDnc1	YPDnc2	YPDnc3	YPDnc4	EtOHnc1	EtOHnc2	Galnc1	Galnc2
13	NOP14	YDL148C	YPDcl2	YPDcl3	EtOHcl1	EtOHcl2	Galcl1	YPDnc1	YPDnc2	YPDnc3	YPDnc4	EtOHnc1	EtOHnc2	Galnc1	Galnc2
13	MCK1	YNL307C	YPDcl2	YPDcl3	EtOHcl1	EtOHcl2	Galcl1	YPDnc1	YPDnc2	YPDnc3	YPDnc4	EtOHnc1	EtOHnc2	Galnc1	Galnc2
13	LSM6	YDR378C	YPDcl2	YPDcl3	EtOHcl1	EtOHcl2	Galcl1	YPDnc1	YPDnc2	YPDnc3	YPDnc4	EtOHnc1	EtOHnc2	Galnc1	Galnc2
13	IBI2	YMR295C	YPDcl2	YPDcl3	EtOHcl1	EtOHcl2	Galcl1	YPDnc1	YPDnc2	YPDnc3	YPDnc4	EtOHnc1	EtOHnc2	Galnc1	Galnc2
13	CCT2	YIL142W	YPDcl2	YPDcl3	EtOHcl1	EtOHcl2	Galcl1	YPDnc1	YPDnc2	YPDnc3	YPDnc4	EtOHnc1	EtOHnc2	Galnc1	Galnc2
13	MDM1	YML104C	YPDcl2	YPDcl3	EtOHcl1	EtOHcl2	Galcl1	YPDnc1	YPDnc2	YPDnc3	YPDnc4	EtOHnc1	EtOHnc2	Galnc1	Galnc2
13	CYC1	YJR048W	YPDcl2	YPDcl3	EtOHcl1	EtOHcl2	Galcl1	YPDnc1	YPDnc2	YPDnc3	YPDnc4	EtOHnc1	EtOHnc2	Galnc1	Galnc2
13	AIM45	YPR004C	YPDcl2	YPDcl3	EtOHcl1	EtOHcl2	Galcl1	YPDnc1	YPDnc2	YPDnc3	YPDnc4	EtOHnc1	EtOHnc2	Galnc1	Galnc2
13	COS12	YGL263W	YPDcl2	YPDcl3	EtOHcl1	EtOHcl2	Galcl1	YPDnc1	YPDnc2	YPDnc3	YPDnc4	EtOHnc1	EtOHnc2	Galnc1	Galnc2
13		YMR034C	YPDcl2	YPDcl3	EtOHcl1	EtOHcl2	Galcl1	YPDnc1	YPDnc2	YPDnc3	YPDnc4	EtOHnc1	EtOHnc2	Galnc1	Galnc2
13	SKI6	YGR195W	YPDcl2	YPDcl3	EtOHcl1	EtOHcl2	Galcl1	YPDnc1	YPDnc2	YPDnc3	YPDnc4	EtOHnc1	EtOHnc2	Galnc1	Galnc2
13	BUB2	YMR055C	YPDcl2	YPDcl3	EtOHcl1	EtOHcl2	Galcl1	YPDnc1	YPDnc2	YPDnc3	YPDnc4	EtOHnc1	EtOHnc2	Galnc1	Galnc2
13	GRX7	YBR014C	YPDcl2	YPDcl3	EtOHcl1	EtOHcl2	Galcl1	YPDnc1	YPDnc2	YPDnc3	YPDnc4	EtOHnc1	EtOHnc2	Galnc1	Galnc2
13	RPP2A	YOL039W	YPDcl2	YPDcl3	EtOHcl1	EtOHcl2	Galcl1	YPDnc1	YPDnc2	YPDnc3	YPDnc4	EtOHnc1	EtOHnc2	Galnc1	Galnc2
13	VMA6	YLR447C	YPDcl2	YPDcl3	EtOHcl1	EtOHcl2	Galcl1	YPDnc1	YPDnc2	YPDnc3	YPDnc4	EtOHnc1	EtOHnc2	Galnc1	Galnc2
13	HTZ1	YOL012C	YPDcl2	YPDcl3	EtOHcl1	EtOHcl2	Galcl1	YPDnc1	YPDnc2	YPDnc3	YPDnc4	EtOHnc1	EtOHnc2	Galnc1	Galnc2
13	PEX14	YGL153W	YPDcl2	YPDcl3	EtOHcl1	EtOHcl2	Galcl1	YPDnc1	YPDnc2	YPDnc3	YPDnc4	EtOHnc1	EtOHnc2	Galnc1	Galnc2
13	MEP3	YPR138C	YPDcl2	YPDcl3	EtOHcl1	EtOHcl2	Galcl1	YPDnc1	YPDnc2	YPDnc3	YPDnc4	EtOHnc1	EtOHnc2	Galnc1	Galnc2
13	MSM1	YGR171C	YPDcl2	YPDcl3	EtOHcl1	EtOHcl2	Galcl1	YPDnc1	YPDnc2	YPDnc3	YPDnc4	EtOHnc1	EtOHnc2	Galnc1	Galnc2
13	MDM10	YAL010C	YPDcl2	YPDcl3	EtOHcl1	EtOHcl2	Galcl1	YPDnc1	YPDnc2	YPDnc3	YPDnc4	EtOHnc1	EtOHnc2	Galnc1	Galnc2
13	TPK3	YKL166C	YPDcl2	YPDcl3	EtOHcl1	EtOHcl2	Galcl1	YPDnc1	YPDnc2	YPDnc3	YPDnc4	EtOHnc1	EtOHnc2	Galnc1	Galnc2
13	FPR3	YML074C	YPDcl2	YPDcl3	EtOHcl1	EtOHcl2	Galcl1	YPDnc1	YPDnc2	YPDnc3	YPDnc4	EtOHnc1	EtOHnc2	Galnc1	Galnc2
13		YJR151W-A	YPDcl2	YPDcl3	EtOHcl1	EtOHcl2	Galcl1	YPDnc1	YPDnc2	YPDnc3	YPDnc4	EtOHnc1	EtOHnc2	Galnc1	Galnc2
13	PRP2	YNR011C	YPDcl2	YPDcl3	EtOHcl1	EtOHcl2	Galcl1	YPDnc1	YPDnc2	YPDnc3	YPDnc4	EtOHnc1	EtOHnc2	Galnc1	Galnc2
13	SEM1	YDR363W-A	YPDcl2	YPDcl3	EtOHcl1	EtOHcl2	Galcl1	YPDnc1	YPDnc2	YPDnc3	YPDnc4	EtOHnc1	EtOHnc2	Galnc1	Galnc2
13	RTT109	YLL002W	YPDcl2	YPDcl3	EtOHcl1	EtOHcl2	Galcl1	YPDnc1	YPDnc2	YPDnc3	YPDnc4	EtOHnc1	EtOHnc2	Galnc1	Galnc2
13	PUF3	YLL013C	YPDcl2	YPDcl3	EtOHcl1	EtOHcl2	Galcl1	YPDnc1	YPDnc2	YPDnc3	YPDnc4	EtOHnc1	EtOHnc2	Galnc1	Galnc2
13	SUR4	YLR372W	YPDcl2	YPDcl3	EtOHcl1	EtOHcl2	Galcl1	YPDnc1	YPDnc2	YPDnc3	YPDnc4	EtOHnc1	EtOHnc2	Galnc1	Galnc2

13	CYC2	YOR037W	YPDcl2	YPDcl3	EtOHcl1	EtOHcl2	Galcl1	YPDnc1	YPDnc2	YPDnc3	YPDnc4	EtOHnc1	EtOHnc2	Galnc1	Galnc2
13	ARN2	YHL047C	YPDcl2	YPDcl3	EtOHcl1	EtOHcl2	Galcl1	YPDnc1	YPDnc2	YPDnc3	YPDnc4	EtOHnc1	EtOHnc2	Galnc1	Galnc2
13	NOP16	YER002W	YPDcl2	YPDcl3	EtOHcl1	EtOHcl2	Galcl1	YPDnc1	YPDnc2	YPDnc3	YPDnc4	EtOHnc1	EtOHnc2	Galnc1	Galnc2
13	PST2	YDR032C	YPDcl2	YPDcl3	EtOHcl1	EtOHcl2	Galcl1	YPDnc1	YPDnc2	YPDnc3	YPDnc4	EtOHnc1	EtOHnc2	Galnc1	Galnc2
13		YJL133C-A	YPDcl2	YPDcl3	EtOHcl1	EtOHcl2	Galcl1	YPDnc1	YPDnc2	YPDnc3	YPDnc4	EtOHnc1	EtOHnc2	Galnc1	Galnc2
13	ABM1	YJR108W	YPDcl2	YPDcl3	EtOHcl1	EtOHcl2	Galcl1	YPDnc1	YPDnc2	YPDnc3	YPDnc4	EtOHnc1	EtOHnc2	Galnc1	Galnc2
13	SNT1	YCR033W	YPDcl2	YPDcl3	EtOHcl1	EtOHcl2	Galcl1	YPDnc1	YPDnc2	YPDnc3	YPDnc4	EtOHnc1	EtOHnc2	Galnc1	Galnc2
13	CCT3	YJL014W	YPDcl2	YPDcl3	EtOHcl1	EtOHcl2	Galcl1	YPDnc1	YPDnc2	YPDnc3	YPDnc4	EtOHnc1	EtOHnc2	Galnc1	Galnc2
13	IAH1	YOR126C	YPDcl2	YPDcl3	EtOHcl1	EtOHcl2	Galcl1	YPDnc1	YPDnc2	YPDnc3	YPDnc4	EtOHnc1	EtOHnc2	Galnc1	Galnc2
13	RTT101	YJL047C	YPDcl2	YPDcl3	EtOHcl1	EtOHcl2	Galcl1	YPDnc1	YPDnc2	YPDnc3	YPDnc4	EtOHnc1	EtOHnc2	Galnc1	Galnc2
13		YDL118W	YPDcl2	YPDcl3	EtOHcl1	EtOHcl2	Galcl1	YPDnc1	YPDnc2	YPDnc3	YPDnc4	EtOHnc1	EtOHnc2	Galnc1	Galnc2
13		YER133W-/	YPDcl2	YPDcl3	EtOHcl1	EtOHcl2	Galcl1	YPDnc1	YPDnc2	YPDnc3	YPDnc4	EtOHnc1	EtOHnc2	Galnc1	Galnc2
13	UPS1	YLR193C	YPDcl2	YPDcl3	EtOHcl1	EtOHcl2	Galcl1	YPDnc1	YPDnc2	YPDnc3	YPDnc4	EtOHnc1	EtOHnc2	Galnc1	Galnc2
13	MCM3	YEL032W	YPDcl2	YPDcl3	EtOHcl1	EtOHcl2	Galcl1	YPDnc1	YPDnc2	YPDnc3	YPDnc4	EtOHnc1	EtOHnc2	Galnc1	Galnc2
13	FUS3	YBL016W	YPDcl2	YPDcl3	EtOHcl1	EtOHcl2	Galcl1	YPDnc1	YPDnc2	YPDnc3	YPDnc4	EtOHnc1	EtOHnc2	Galnc1	Galnc2
13	ERP1	YAR002C-A	YPDcl2	YPDcl3	EtOHcl1	EtOHcl2	Galcl1	YPDnc1	YPDnc2	YPDnc3	YPDnc4	EtOHnc1	EtOHnc2	Galnc1	Galnc2
13	OPY1	YBR129C	YPDcl2	YPDcl3	EtOHcl1	EtOHcl2	Galcl1	YPDnc1	YPDnc2	YPDnc3	YPDnc4	EtOHnc1	EtOHnc2	Galnc1	Galnc2
13	OGG1	YML060W	YPDcl2	YPDcl3	EtOHcl1	EtOHcl2	Galcl1	YPDnc1	YPDnc2	YPDnc3	YPDnc4	EtOHnc1	EtOHnc2	Galnc1	Galnc2
13		YLR218C	YPDcl2	YPDcl3	EtOHcl1	EtOHcl2	Galcl1	YPDnc1	YPDnc2	YPDnc3	YPDnc4	EtOHnc1	EtOHnc2	Galnc1	Galnc2
13	MSF1	YPR047W	YPDcl2	YPDcl3	EtOHcl1	EtOHcl2	Galcl1	YPDnc1	YPDnc2	YPDnc3	YPDnc4	EtOHnc1	EtOHnc2	Galnc1	Galnc2
13	SLP1	YOR154W	YPDcl2	YPDcl3	EtOHcl1	EtOHcl2	Galcl1	YPDnc1	YPDnc2	YPDnc3	YPDnc4	EtOHnc1	EtOHnc2	Galnc1	Galnc2
13		YPR170W-/	YPDcl2	YPDcl3	EtOHcl1	EtOHcl2	Galcl1	YPDnc1	YPDnc2	YPDnc3	YPDnc4	EtOHnc1	EtOHnc2	Galnc1	Galnc2
13		YKL123W	YPDcl2	YPDcl3	EtOHcl1	EtOHcl2	Galcl1	YPDnc1	YPDnc2	YPDnc3	YPDnc4	EtOHnc1	EtOHnc2	Galnc1	Galnc2
13	SEH1	YGL100W	YPDcl2	YPDcl3	EtOHcl1	EtOHcl2	Galcl1	YPDnc1	YPDnc2	YPDnc3	YPDnc4	EtOHnc1	EtOHnc2	Galnc1	Galnc2
13		YDR510C-A	YPDcl2	YPDcl3	EtOHcl1	EtOHcl2	Galcl1	YPDnc1	YPDnc2	YPDnc3	YPDnc4	EtOHnc1	EtOHnc2	Galnc1	Galnc2
13	MSS51	YLR203C	YPDcl2	YPDcl3	EtOHcl1	EtOHcl2	Galcl1	YPDnc1	YPDnc2	YPDnc3	YPDnc4	EtOHnc1	EtOHnc2	Galnc1	Galnc2
13	CDC123	YLR215C	YPDcl2	YPDcl3	EtOHcl1	EtOHcl2	Galcl1	YPDnc1	YPDnc2	YPDnc3	YPDnc4	EtOHnc1	EtOHnc2	Galnc1	Galnc2
13	GDH1	YOR375C	YPDcl2	YPDcl3	EtOHcl1	EtOHcl2	Galcl1	YPDnc1	YPDnc2	YPDnc3	YPDnc4	EtOHnc1	EtOHnc2	Galnc1	Galnc2
13	SSF1	YHR066W	YPDcl2	YPDcl3	EtOHcl1	EtOHcl2	Galcl1	YPDnc1	YPDnc2	YPDnc3	YPDnc4	EtOHnc1	EtOHnc2	Galnc1	Galnc2
13		YLR312C	YPDcl2	YPDcl3	EtOHcl1	EtOHcl2	Galcl1	YPDnc1	YPDnc2	YPDnc3	YPDnc4	EtOHnc1	EtOHnc2	Galnc1	Galnc2
13		YHR122W	YPDcl2	YPDcl3	EtOHcl1	EtOHcl2	Galcl1	YPDnc1	YPDnc2	YPDnc3	YPDnc4	EtOHnc1	EtOHnc2	Galnc1	Galnc2
13	GLT1	YDL171C	YPDcl2	YPDcl3	EtOHcl1	EtOHcl2	Galcl1	YPDnc1	YPDnc2	YPDnc3	YPDnc4	EtOHnc1	EtOHnc2	Galnc1	Galnc2
13		YEL068C	YPDcl2	YPDcl3	EtOHcl1	EtOHcl2	Galcl1	YPDnc1	YPDnc2	YPDnc3	YPDnc4	EtOHnc1	EtOHnc2	Galnc1	Galnc2
13	GIM5	YML094W	YPDcl2	YPDcl3	EtOHcl1	EtOHcl2	Galcl1	YPDnc1	YPDnc2	YPDnc3	YPDnc4	EtOHnc1	EtOHnc2	Galnc1	Galnc2

13	SKT5	YBL061C	YPDcl2	YPDcl3	EtOHcl1	EtOHcl2	Galcl1	YPDnc1	YPDnc2	YPDnc3	YPDnc4	EtOHnc1	EtOHnc2	Galnc1	Galnc2
13	FAP1	YNL023C	YPDcl2	YPDcl3	EtOHcl1	EtOHcl2	Galcl1	YPDnc1	YPDnc2	YPDnc3	YPDnc4	EtOHnc1	EtOHnc2	Galnc1	Galnc2
13	BOR1	YNL275W	YPDcl2	YPDcl3	EtOHcl1	EtOHcl2	Galcl1	YPDnc1	YPDnc2	YPDnc3	YPDnc4	EtOHnc1	EtOHnc2	Galnc1	Galnc2
13		YGR016W	YPDcl2	YPDcl3	EtOHcl1	EtOHcl2	Galcl1	YPDnc1	YPDnc2	YPDnc3	YPDnc4	EtOHnc1	EtOHnc2	Galnc1	Galnc2
13		YKR005C	YPDcl2	YPDcl3	EtOHcl1	EtOHcl2	Galcl1	YPDnc1	YPDnc2	YPDnc3	YPDnc4	EtOHnc1	EtOHnc2	Galnc1	Galnc2
13		YJL206C	YPDcl2	YPDcl3	EtOHcl1	EtOHcl2	Galcl1	YPDnc1	YPDnc2	YPDnc3	YPDnc4	EtOHnc1	EtOHnc2	Galnc1	Galnc2
13	ACP1	YKL192C	YPDcl2	YPDcl3	EtOHcl1	EtOHcl2	Galcl1	YPDnc1	YPDnc2	YPDnc3	YPDnc4	EtOHnc1	EtOHnc2	Galnc1	Galnc2
13	DDI1	YER143W	YPDcl2	YPDcl3	EtOHcl1	EtOHcl2	Galcl1	YPDnc1	YPDnc2	YPDnc3	YPDnc4	EtOHnc1	EtOHnc2	Galnc1	Galnc2
13	SPT14	YPL175W	YPDcl2	YPDcl3	EtOHcl1	EtOHcl2	Galcl1	YPDnc1	YPDnc2	YPDnc3	YPDnc4	EtOHnc1	EtOHnc2	Galnc1	Galnc2
13		YOR329W-	YPDcl2	YPDcl3	EtOHcl1	EtOHcl2	Galcl1	YPDnc1	YPDnc2	YPDnc3	YPDnc4	EtOHnc1	EtOHnc2	Galnc1	Galnc2
13	MRPS5	YBR251W	YPDcl2	YPDcl3	EtOHcl1	EtOHcl2	Galcl1	YPDnc1	YPDnc2	YPDnc3	YPDnc4	EtOHnc1	EtOHnc2	Galnc1	Galnc2
13	QCR8	YJL166W	YPDcl2	YPDcl3	EtOHcl1	EtOHcl2	Galcl1	YPDnc1	YPDnc2	YPDnc3	YPDnc4	EtOHnc1	EtOHnc2	Galnc1	Galnc2
13	MSK1	YNL073W	YPDcl2	YPDcl3	EtOHcl1	EtOHcl2	Galcl1	YPDnc1	YPDnc2	YPDnc3	YPDnc4	EtOHnc1	EtOHnc2	Galnc1	Galnc2
13	ANT1	YPR128C	YPDcl2	YPDcl3	EtOHcl1	EtOHcl2	Galcl1	YPDnc1	YPDnc2	YPDnc3	YPDnc4	EtOHnc1	EtOHnc2	Galnc1	Galnc2
13	MPS2	YGL075C	YPDcl2	YPDcl3	EtOHcl1	EtOHcl2	Galcl1	YPDnc1	YPDnc2	YPDnc3	YPDnc4	EtOHnc1	EtOHnc2	Galnc1	Galnc2
13	EEB1	YPL095C	YPDcl2	YPDcl3	EtOHcl1	EtOHcl2	Galcl1	YPDnc1	YPDnc2	YPDnc3	YPDnc4	EtOHnc1	EtOHnc2	Galnc1	Galnc2
13		YKL162C-A	YPDcl2	YPDcl3	EtOHcl1	EtOHcl2	Galcl1	YPDnc1	YPDnc2	YPDnc3	YPDnc4	EtOHnc1	EtOHnc2	Galnc1	Galnc2
13		YPL038W-A	YPDcl2	YPDcl3	EtOHcl1	EtOHcl2	Galcl1	YPDnc1	YPDnc2	YPDnc3	YPDnc4	EtOHnc1	EtOHnc2	Galnc1	Galnc2
13	CDC21	YOR074C	YPDcl2	YPDcl3	EtOHcl1	EtOHcl2	Galcl1	YPDnc1	YPDnc2	YPDnc3	YPDnc4	EtOHnc1	EtOHnc2	Galnc1	Galnc2
13	RPA12	YJR063W	YPDcl2	YPDcl3	EtOHcl1	EtOHcl2	Galcl1	YPDnc1	YPDnc2	YPDnc3	YPDnc4	EtOHnc1	EtOHnc2	Galnc1	Galnc2
13	VHR1	YIL056W	YPDcl2	YPDcl3	EtOHcl1	EtOHcl2	Galcl1	YPDnc1	YPDnc2	YPDnc3	YPDnc4	EtOHnc1	EtOHnc2	Galnc1	Galnc2
13	LPP1	YDR503C	YPDcl2	YPDcl3	EtOHcl1	EtOHcl2	Galcl1	YPDnc1	YPDnc2	YPDnc3	YPDnc4	EtOHnc1	EtOHnc2	Galnc1	Galnc2
13	DUS1	YML080W	YPDcl2	YPDcl3	EtOHcl1	EtOHcl2	Galcl1	YPDnc1	YPDnc2	YPDnc3	YPDnc4	EtOHnc1	EtOHnc2	Galnc1	Galnc2
13	CTL1	YMR180C	YPDcl2	YPDcl3	EtOHcl1	EtOHcl2	Galcl1	YPDnc1	YPDnc2	YPDnc3	YPDnc4	EtOHnc1	EtOHnc2	Galnc1	Galnc2
13	FRE1	YLR214W	YPDcl2	YPDcl3	EtOHcl1	EtOHcl2	Galcl1	YPDnc1	YPDnc2	YPDnc3	YPDnc4	EtOHnc1	EtOHnc2	Galnc1	Galnc2
13	SNF5	YBR289W	YPDcl2	YPDcl3	EtOHcl1	EtOHcl2	Galcl1	YPDnc1	YPDnc2	YPDnc3	YPDnc4	EtOHnc1	EtOHnc2	Galnc1	Galnc2
13	DID2	YKR035W-A	YPDcl2	YPDcl3	EtOHcl1	EtOHcl2	Galcl1	YPDnc1	YPDnc2	YPDnc3	YPDnc4	EtOHnc1	EtOHnc2	Galnc1	Galnc2
13	IPL1	YPL209C	YPDcl2	YPDcl3	EtOHcl1	EtOHcl2	Galcl1	YPDnc1	YPDnc2	YPDnc3	YPDnc4	EtOHnc1	EtOHnc2	Galnc1	Galnc2
13	NIP100	YPL174C	YPDcl2	YPDcl3	EtOHcl1	EtOHcl2	Galcl1	YPDnc1	YPDnc2	YPDnc3	YPDnc4	EtOHnc1	EtOHnc2	Galnc1	Galnc2
13	TUP1	YCR084C	YPDcl2	YPDcl3	EtOHcl1	EtOHcl2	Galcl1	YPDnc1	YPDnc2	YPDnc3	YPDnc4	EtOHnc1	EtOHnc2	Galnc1	Galnc2
13	CDC40	YDR364C	YPDcl2	YPDcl3	EtOHcl1	EtOHcl2	Galcl1	YPDnc1	YPDnc2	YPDnc3	YPDnc4	EtOHnc1	EtOHnc2	Galnc1	Galnc2
13		YLR257W	YPDcl2	YPDcl3	EtOHcl1	EtOHcl2	Galcl1	YPDnc1	YPDnc2	YPDnc3	YPDnc4	EtOHnc1	EtOHnc2	Galnc1	Galnc2
13	PHM6	YDR281C	YPDcl2	YPDcl3	EtOHcl1	EtOHcl2	Galcl1	YPDnc1	YPDnc2	YPDnc3	YPDnc4	EtOHnc1	EtOHnc2	Galnc1	Galnc2
13	GPB1	YOR371C	YPDcl2	YPDcl3	EtOHcl1	EtOHcl2	Galcl1	YPDnc1	YPDnc2	YPDnc3	YPDnc4	EtOHnc1	EtOHnc2	Galnc1	Galnc2

13	APM4	YOL062C	YPDcl2	YPDcl3	EtOHcl1	EtOHcl2	Galcl1	YPDnc1	YPDnc2	YPDnc3	YPDnc4	EtOHnc1	EtOHnc2	Galnc1	Galnc2
13	NIT2	YJL126W	YPDcl2	YPDcl3	EtOHcl1	EtOHcl2	Galcl1	YPDnc1	YPDnc2	YPDnc3	YPDnc4	EtOHnc1	EtOHnc2	Galnc1	Galnc2
13	NSP1	YJL041W	YPDcl2	YPDcl3	EtOHcl1	EtOHcl2	Galcl1	YPDnc1	YPDnc2	YPDnc3	YPDnc4	EtOHnc1	EtOHnc2	Galnc1	Galnc2
13	SBH2	YER019C-A	YPDcl2	YPDcl3	EtOHcl1	EtOHcl2	Galcl1	YPDnc1	YPDnc2	YPDnc3	YPDnc4	EtOHnc1	EtOHnc2	Galnc1	Galnc2
13	SMF1	YOL122C	YPDcl2	YPDcl3	EtOHcl1	EtOHcl2	Galcl1	YPDnc1	YPDnc2	YPDnc3	YPDnc4	EtOHnc1	EtOHnc2	Galnc1	Galnc2
13	OCH1	YGL038C	YPDcl2	YPDcl3	EtOHcl1	EtOHcl2	Galcl1	YPDnc1	YPDnc2	YPDnc3	YPDnc4	EtOHnc1	EtOHnc2	Galnc1	Galnc2
13	MYO1	YHR023W	YPDcl2	YPDcl3	EtOHcl1	EtOHcl2	Galcl1	YPDnc1	YPDnc2	YPDnc3	YPDnc4	EtOHnc1	EtOHnc2	Galnc1	Galnc2
13	TDH1	YJL052W	YPDcl2	YPDcl3	EtOHcl1	EtOHcl2	Galcl1	YPDnc1	YPDnc2	YPDnc3	YPDnc4	EtOHnc1	EtOHnc2	Galnc1	Galnc2
13	TFA2	YKR062W	YPDcl2	YPDcl3	EtOHcl1	EtOHcl2	Galcl1	YPDnc1	YPDnc2	YPDnc3	YPDnc4	EtOHnc1	EtOHnc2	Galnc1	Galnc2
13	MGA2	YIR033W	YPDcl2	YPDcl3	EtOHcl1	EtOHcl2	Galcl1	YPDnc1	YPDnc2	YPDnc3	YPDnc4	EtOHnc1	EtOHnc2	Galnc1	Galnc2
13	STR3	YGL184C	YPDcl2	YPDcl3	EtOHcl1	EtOHcl2	Galcl1	YPDnc1	YPDnc2	YPDnc3	YPDnc4	EtOHnc1	EtOHnc2	Galnc1	Galnc2
13	RMP1	YLR145W	YPDcl2	YPDcl3	EtOHcl1	EtOHcl2	Galcl1	YPDnc1	YPDnc2	YPDnc3	YPDnc4	EtOHnc1	EtOHnc2	Galnc1	Galnc2
13	HMI1	YOL095C	YPDcl2	YPDcl3	EtOHcl1	EtOHcl2	Galcl1	YPDnc1	YPDnc2	YPDnc3	YPDnc4	EtOHnc1	EtOHnc2	Galnc1	Galnc2
13	YET2	YMR040W	YPDcl2	YPDcl3	EtOHcl1	EtOHcl2	Galcl1	YPDnc1	YPDnc2	YPDnc3	YPDnc4	EtOHnc1	EtOHnc2	Galnc1	Galnc2
13	TYE7	YOR344C	YPDcl2	YPDcl3	EtOHcl1	EtOHcl2	Galcl1	YPDnc1	YPDnc2	YPDnc3	YPDnc4	EtOHnc1	EtOHnc2	Galnc1	Galnc2
13	ATP18	YML081C-A	YPDcl2	YPDcl3	EtOHcl1	EtOHcl2	Galcl1	YPDnc1	YPDnc2	YPDnc3	YPDnc4	EtOHnc1	EtOHnc2	Galnc1	Galnc2
13	NIT3	YLR351C	YPDcl2	YPDcl3	EtOHcl1	EtOHcl2	Galcl1	YPDnc1	YPDnc2	YPDnc3	YPDnc4	EtOHnc1	EtOHnc2	Galnc1	Galnc2
13	GGC1	YDL198C	YPDcl2	YPDcl3	EtOHcl1	EtOHcl2	Galcl1	YPDnc1	YPDnc2	YPDnc3	YPDnc4	EtOHnc1	EtOHnc2	Galnc1	Galnc2
13	DRS2	YAL026C	YPDcl2	YPDcl3	EtOHcl1	EtOHcl2	Galcl1	YPDnc1	YPDnc2	YPDnc3	YPDnc4	EtOHnc1	EtOHnc2	Galnc1	Galnc2
13	FYV7	YLR068W	YPDcl2	YPDcl3	EtOHcl1	EtOHcl2	Galcl1	YPDnc1	YPDnc2	YPDnc3	YPDnc4	EtOHnc1	EtOHnc2	Galnc1	Galnc2
13		YPL185W	YPDcl2	YPDcl3	EtOHcl1	EtOHcl2	Galcl1	YPDnc1	YPDnc2	YPDnc3	YPDnc4	EtOHnc1	EtOHnc2	Galnc1	Galnc2
13	HTS1	YPR033C	YPDcl2	YPDcl3	EtOHcl1	EtOHcl2	Galcl1	YPDnc1	YPDnc2	YPDnc3	YPDnc4	EtOHnc1	EtOHnc2	Galnc1	Galnc2
13	ERO1	YML130C	YPDcl2	YPDcl3	EtOHcl1	EtOHcl2	Galcl1	YPDnc1	YPDnc2	YPDnc3	YPDnc4	EtOHnc1	EtOHnc2	Galnc1	Galnc2
13	PHO86	YJL117W	YPDcl2	YPDcl3	EtOHcl1	EtOHcl2	Galcl1	YPDnc1	YPDnc2	YPDnc3	YPDnc4	EtOHnc1	EtOHnc2	Galnc1	Galnc2
13		YJR087W	YPDcl2	YPDcl3	EtOHcl1	EtOHcl2	Galcl1	YPDnc1	YPDnc2	YPDnc3	YPDnc4	EtOHnc1	EtOHnc2	Galnc1	Galnc2
13	ARO80	YDR421W	YPDcl2	YPDcl3	EtOHcl1	EtOHcl2	Galcl1	YPDnc1	YPDnc2	YPDnc3	YPDnc4	EtOHnc1	EtOHnc2	Galnc1	Galnc2
13	GCV1	YDR019C	YPDcl2	YPDcl3	EtOHcl1	EtOHcl2	Galcl1	YPDnc1	YPDnc2	YPDnc3	YPDnc4	EtOHnc1	EtOHnc2	Galnc1	Galnc2
13	TFC7	YOR110W	YPDcl2	YPDcl3	EtOHcl1	EtOHcl2	Galcl1	YPDnc1	YPDnc2	YPDnc3	YPDnc4	EtOHnc1	EtOHnc2	Galnc1	Galnc2
13	WHI2	YOR043W	YPDcl2	YPDcl3	EtOHcl1	EtOHcl2	Galcl1	YPDnc1	YPDnc2	YPDnc3	YPDnc4	EtOHnc1	EtOHnc2	Galnc1	Galnc2
13	END3	YNL084C	YPDcl2	YPDcl3	EtOHcl1	EtOHcl2	Galcl1	YPDnc1	YPDnc2	YPDnc3	YPDnc4	EtOHnc1	EtOHnc2	Galnc1	Galnc2
13	TPI1	YDR050C	YPDcl2	YPDcl3	EtOHcl1	EtOHcl2	Galcl1	YPDnc1	YPDnc2	YPDnc3	YPDnc4	EtOHnc1	EtOHnc2	Galnc1	Galnc2
13	RPT5	YOR117W	YPDcl2	YPDcl3	EtOHcl1	EtOHcl2	Galcl1	YPDnc1	YPDnc2	YPDnc3	YPDnc4	EtOHnc1	EtOHnc2	Galnc1	Galnc2
13		YOR072W	YPDcl2	YPDcl3	EtOHcl1	EtOHcl2	Galcl1	YPDnc1	YPDnc2	YPDnc3	YPDnc4	EtOHnc1	EtOHnc2	Galnc1	Galnc2
13	PHD1	YKL043W	YPDcl2	YPDcl3	EtOHcl1	EtOHcl2	Galcl1	YPDnc1	YPDnc2	YPDnc3	YPDnc4	EtOHnc1	EtOHnc2	Galnc1	Galnc2

13	NMT1	YLR195C	YPDcl2	YPDcl3	EtOHcl1	EtOHcl2	Galcl1	YPDnc1	YPDnc2	YPDnc3	YPDnc4	EtOHnc1	EtOHnc2	Galnc1	Galnc2
13	ITT1	YML068W	YPDcl2	YPDcl3	EtOHcl1	EtOHcl2	Galcl1	YPDnc1	YPDnc2	YPDnc3	YPDnc4	EtOHnc1	EtOHnc2	Galnc1	Galnc2
13	CMK1	YFR014C	YPDcl2	YPDcl3	EtOHcl1	EtOHcl2	Galcl1	YPDnc1	YPDnc2	YPDnc3	YPDnc4	EtOHnc1	EtOHnc2	Galnc1	Galnc2
13	IPK1	YDR315C	YPDcl2	YPDcl3	EtOHcl1	EtOHcl2	Galcl1	YPDnc1	YPDnc2	YPDnc3	YPDnc4	EtOHnc1	EtOHnc2	Galnc1	Galnc2
13	MCH1	YDL054C	YPDcl2	YPDcl3	EtOHcl1	EtOHcl2	Galcl1	YPDnc1	YPDnc2	YPDnc3	YPDnc4	EtOHnc1	EtOHnc2	Galnc1	Galnc2
13	RDS2	YPL133C	YPDcl2	YPDcl3	EtOHcl1	EtOHcl2	Galcl1	YPDnc1	YPDnc2	YPDnc3	YPDnc4	EtOHnc1	EtOHnc2	Galnc1	Galnc2
13	GRS2	YPR081C	YPDcl2	YPDcl3	EtOHcl1	EtOHcl2	Galcl1	YPDnc1	YPDnc2	YPDnc3	YPDnc4	EtOHnc1	EtOHnc2	Galnc1	Galnc2
13	IRC23	YOR044W	YPDcl2	YPDcl3	EtOHcl1	EtOHcl2	Galcl1	YPDnc1	YPDnc2	YPDnc3	YPDnc4	EtOHnc1	EtOHnc2	Galnc1	Galnc2
13	DSE2	YHR143W	YPDcl2	YPDcl3	EtOHcl1	EtOHcl2	Galcl1	YPDnc1	YPDnc2	YPDnc3	YPDnc4	EtOHnc1	EtOHnc2	Galnc1	Galnc2
13	AAD6	YFL056C	YPDcl2	YPDcl3	EtOHcl1	EtOHcl2	Galcl1	YPDnc1	YPDnc2	YPDnc3	YPDnc4	EtOHnc1	EtOHnc2	Galnc1	Galnc2
13	VPS54	YDR027C	YPDcl2	YPDcl3	EtOHcl1	EtOHcl2	Galcl1	YPDnc1	YPDnc2	YPDnc3	YPDnc4	EtOHnc1	EtOHnc2	Galnc1	Galnc2
13		YHR035W	YPDcl2	YPDcl3	EtOHcl1	EtOHcl2	Galcl1	YPDnc1	YPDnc2	YPDnc3	YPDnc4	EtOHnc1	EtOHnc2	Galnc1	Galnc2
13		YLR140W	YPDcl2	YPDcl3	EtOHcl1	EtOHcl2	Galcl1	YPDnc1	YPDnc2	YPDnc3	YPDnc4	EtOHnc1	EtOHnc2	Galnc1	Galnc2
13	GCD2	YGR083C	YPDcl2	YPDcl3	EtOHcl1	EtOHcl2	Galcl1	YPDnc1	YPDnc2	YPDnc3	YPDnc4	EtOHnc1	EtOHnc2	Galnc1	Galnc2
13	TSR4	YOL022C	YPDcl2	YPDcl3	EtOHcl1	EtOHcl2	Galcl1	YPDnc1	YPDnc2	YPDnc3	YPDnc4	EtOHnc1	EtOHnc2	Galnc1	Galnc2
13	LIF1	YGL090W	YPDcl2	YPDcl3	EtOHcl1	EtOHcl2	Galcl1	YPDnc1	YPDnc2	YPDnc3	YPDnc4	EtOHnc1	EtOHnc2	Galnc1	Galnc2
13	UTR1	YJR049C	YPDcl2	YPDcl3	EtOHcl1	EtOHcl2	Galcl1	YPDnc1	YPDnc2	YPDnc3	YPDnc4	EtOHnc1	EtOHnc2	Galnc1	Galnc2
13		YJR071W	YPDcl2	YPDcl3	EtOHcl1	EtOHcl2	Galcl1	YPDnc1	YPDnc2	YPDnc3	YPDnc4	EtOHnc1	EtOHnc2	Galnc1	Galnc2
13	GYP7	YDL234C	YPDcl2	YPDcl3	EtOHcl1	EtOHcl2	Galcl1	YPDnc1	YPDnc2	YPDnc3	YPDnc4	EtOHnc1	EtOHnc2	Galnc1	Galnc2
13	GIS3	YLR094C	YPDcl2	YPDcl3	EtOHcl1	EtOHcl2	Galcl1	YPDnc1	YPDnc2	YPDnc3	YPDnc4	EtOHnc1	EtOHnc2	Galnc1	Galnc2
13	NGL3	YML118W	YPDcl2	YPDcl3	EtOHcl1	EtOHcl2	Galcl1	YPDnc1	YPDnc2	YPDnc3	YPDnc4	EtOHnc1	EtOHnc2	Galnc1	Galnc2
13	RPL14B	YHL001W	YPDcl2	YPDcl3	EtOHcl1	EtOHcl2	Galcl1	YPDnc1	YPDnc2	YPDnc3	YPDnc4	EtOHnc1	EtOHnc2	Galnc1	Galnc2
13	YPR1	YDR368W	YPDcl2	YPDcl3	EtOHcl1	EtOHcl2	Galcl1	YPDnc1	YPDnc2	YPDnc3	YPDnc4	EtOHnc1	EtOHnc2	Galnc1	Galnc2
13		YPR014C	YPDcl2	YPDcl3	EtOHcl1	EtOHcl2	Galcl1	YPDnc1	YPDnc2	YPDnc3	YPDnc4	EtOHnc1	EtOHnc2	Galnc1	Galnc2
13	RPC17	YJL011C	YPDcl2	YPDcl3	EtOHcl1	EtOHcl2	Galcl1	YPDnc1	YPDnc2	YPDnc3	YPDnc4	EtOHnc1	EtOHnc2	Galnc1	Galnc2
13	MCT1	YOR221C	YPDcl2	YPDcl3	EtOHcl1	EtOHcl2	Galcl1	YPDnc1	YPDnc2	YPDnc3	YPDnc4	EtOHnc1	EtOHnc2	Galnc1	Galnc2
13		YKL177W	YPDcl2	YPDcl3	EtOHcl1	EtOHcl2	Galcl1	YPDnc1	YPDnc2	YPDnc3	YPDnc4	EtOHnc1	EtOHnc2	Galnc1	Galnc2
13	RFA1	YAR007C	YPDcl2	YPDcl3	EtOHcl1	EtOHcl2	Galcl1	YPDnc1	YPDnc2	YPDnc3	YPDnc4	EtOHnc1	EtOHnc2	Galnc1	Galnc2
13	AML1	YGR001C	YPDcl2	YPDcl3	EtOHcl1	EtOHcl2	Galcl1	YPDnc1	YPDnc2	YPDnc3	YPDnc4	EtOHnc1	EtOHnc2	Galnc1	Galnc2
13	SEC13	YLR208W	YPDcl2	YPDcl3	EtOHcl1	EtOHcl2	Galcl1	YPDnc1	YPDnc2	YPDnc3	YPDnc4	EtOHnc1	EtOHnc2	Galnc1	Galnc2
13	POA1	YBR022W	YPDcl2	YPDcl3	EtOHcl1	EtOHcl2	Galcl1	YPDnc1	YPDnc2	YPDnc3	YPDnc4	EtOHnc1	EtOHnc2	Galnc1	Galnc2
13	GNT1	YOR320C	YPDcl2	YPDcl3	EtOHcl1	EtOHcl2	Galcl1	YPDnc1	YPDnc2	YPDnc3	YPDnc4	EtOHnc1	EtOHnc2	Galnc1	Galnc2
13	MAL13	YGR288W	YPDcl2	YPDcl3	EtOHcl1	EtOHcl2	Galcl1	YPDnc1	YPDnc2	YPDnc3	YPDnc4	EtOHnc1	EtOHnc2	Galnc1	Galnc2
13		YOR385W	YPDcl2	YPDcl3	EtOHcl1	EtOHcl2	Galcl1	YPDnc1	YPDnc2	YPDnc3	YPDnc4	EtOHnc1	EtOHnc2	Galnc1	Galnc2

13	ADY3	YDL239C	YPDcl2	YPDcl3	EtOHcl1	EtOHcl2	Galcl1	YPDnc1	YPDnc2	YPDnc3	YPDnc4	EtOHnc1	EtOHnc2	Galnc1	Galnc2
13	MTC5	YDR128W	YPDcl2	YPDcl3	EtOHcl1	EtOHcl2	Galcl1	YPDnc1	YPDnc2	YPDnc3	YPDnc4	EtOHnc1	EtOHnc2	Galnc1	Galnc2
13	TUB1	YML085C	YPDcl2	YPDcl3	EtOHcl1	EtOHcl2	Galcl1	YPDnc1	YPDnc2	YPDnc3	YPDnc4	EtOHnc1	EtOHnc2	Galnc1	Galnc2
13	OYE2	YHR179W	YPDcl2	YPDcl3	EtOHcl1	EtOHcl2	Galcl1	YPDnc1	YPDnc2	YPDnc3	YPDnc4	EtOHnc1	EtOHnc2	Galnc1	Galnc2
13	SPC24	YMR117C	YPDcl2	YPDcl3	EtOHcl1	EtOHcl2	Galcl1	YPDnc1	YPDnc2	YPDnc3	YPDnc4	EtOHnc1	EtOHnc2	Galnc1	Galnc2
13	DPB2	YPR175W	YPDcl2	YPDcl3	EtOHcl1	EtOHcl2	Galcl1	YPDnc1	YPDnc2	YPDnc3	YPDnc4	EtOHnc1	EtOHnc2	Galnc1	Galnc2
13	SEC2	YNL272C	YPDcl2	YPDcl3	EtOHcl1	EtOHcl2	Galcl1	YPDnc1	YPDnc2	YPDnc3	YPDnc4	EtOHnc1	EtOHnc2	Galnc1	Galnc2
13		YJR038C	YPDcl2	YPDcl3	EtOHcl1	EtOHcl2	Galcl1	YPDnc1	YPDnc2	YPDnc3	YPDnc4	EtOHnc1	EtOHnc2	Galnc1	Galnc2
13	RPO31	YOR116C	YPDcl2	YPDcl3	EtOHcl1	EtOHcl2	Galcl1	YPDnc1	YPDnc2	YPDnc3	YPDnc4	EtOHnc1	EtOHnc2	Galnc1	Galnc2
13	MBR1	YKL093W	YPDcl2	YPDcl3	EtOHcl1	EtOHcl2	Galcl1	YPDnc1	YPDnc2	YPDnc3	YPDnc4	EtOHnc1	EtOHnc2	Galnc1	Galnc2
13	UFO1	YML088W	YPDcl2	YPDcl3	EtOHcl1	EtOHcl2	Galcl1	YPDnc1	YPDnc2	YPDnc3	YPDnc4	EtOHnc1	EtOHnc2	Galnc1	Galnc2
13	SEC3	YER008C	YPDcl2	YPDcl3	EtOHcl1	EtOHcl2	Galcl1	YPDnc1	YPDnc2	YPDnc3	YPDnc4	EtOHnc1	EtOHnc2	Galnc1	Galnc2
13	UBC5	YDR059C	YPDcl2	YPDcl3	EtOHcl1	EtOHcl2	Galcl1	YPDnc1	YPDnc2	YPDnc3	YPDnc4	EtOHnc1	EtOHnc2	Galnc1	Galnc2
13	ADY4	YLR227C	YPDcl2	YPDcl3	EtOHcl1	EtOHcl2	Galcl1	YPDnc1	YPDnc2	YPDnc3	YPDnc4	EtOHnc1	EtOHnc2	Galnc1	Galnc2
13	TRM11	YOL124C	YPDcl2	YPDcl3	EtOHcl1	EtOHcl2	Galcl1	YPDnc1	YPDnc2	YPDnc3	YPDnc4	EtOHnc1	EtOHnc2	Galnc1	Galnc2
13	COQ2	YNR041C	YPDcl2	YPDcl3	EtOHcl1	EtOHcl2	Galcl1	YPDnc1	YPDnc2	YPDnc3	YPDnc4	EtOHnc1	EtOHnc2	Galnc1	Galnc2
13		YJL052C-A	YPDcl2	YPDcl3	EtOHcl1	EtOHcl2	Galcl1	YPDnc1	YPDnc2	YPDnc3	YPDnc4	EtOHnc1	EtOHnc2	Galnc1	Galnc2
13	MNI1	YIL110W	YPDcl2	YPDcl3	EtOHcl1	EtOHcl2	Galcl1	YPDnc1	YPDnc2	YPDnc3	YPDnc4	EtOHnc1	EtOHnc2	Galnc1	Galnc2
13	BDS1	YOL164W	YPDcl2	YPDcl3	EtOHcl1	EtOHcl2	Galcl1	YPDnc1	YPDnc2	YPDnc3	YPDnc4	EtOHnc1	EtOHnc2	Galnc1	Galnc2
13	YCS4	YLR272C	YPDcl2	YPDcl3	EtOHcl1	EtOHcl2	Galcl1	YPDnc1	YPDnc2	YPDnc3	YPDnc4	EtOHnc1	EtOHnc2	Galnc1	Galnc2
13	DSF1	YEL070W	YPDcl2	YPDcl3	EtOHcl1	EtOHcl2	Galcl1	YPDnc1	YPDnc2	YPDnc3	YPDnc4	EtOHnc1	EtOHnc2	Galnc1	Galnc2
13	RTA1	YGR213C	YPDcl2	YPDcl3	EtOHcl1	EtOHcl2	Galcl1	YPDnc1	YPDnc2	YPDnc3	YPDnc4	EtOHnc1	EtOHnc2	Galnc1	Galnc2
13	HOS1	YPR068C	YPDcl2	YPDcl3	EtOHcl1	EtOHcl2	Galcl1	YPDnc1	YPDnc2	YPDnc3	YPDnc4	EtOHnc1	EtOHnc2	Galnc1	Galnc2
13	PET20	YPL159C	YPDcl2	YPDcl3	EtOHcl1	EtOHcl2	Galcl1	YPDnc1	YPDnc2	YPDnc3	YPDnc4	EtOHnc1	EtOHnc2	Galnc1	Galnc2
13	BCK2	YER167W	YPDcl2	YPDcl3	EtOHcl1	EtOHcl2	Galcl1	YPDnc1	YPDnc2	YPDnc3	YPDnc4	EtOHnc1	EtOHnc2	Galnc1	Galnc2
13	IWR1	YDL115C	YPDcl2	YPDcl3	EtOHcl1	EtOHcl2	Galcl1	YPDnc1	YPDnc2	YPDnc3	YPDnc4	EtOHnc1	EtOHnc2	Galnc1	Galnc2
13	ADH4	YGL256W	YPDcl2	YPDcl3	EtOHcl1	EtOHcl2	Galcl1	YPDnc1	YPDnc2	YPDnc3	YPDnc4	EtOHnc1	EtOHnc2	Galnc1	Galnc2
13		YMR147W	YPDcl2	YPDcl3	EtOHcl1	EtOHcl2	Galcl1	YPDnc1	YPDnc2	YPDnc3	YPDnc4	EtOHnc1	EtOHnc2	Galnc1	Galnc2
13	RBS1	YDL189W	YPDcl2	YPDcl3	EtOHcl1	EtOHcl2	Galcl1	YPDnc1	YPDnc2	YPDnc3	YPDnc4	EtOHnc1	EtOHnc2	Galnc1	Galnc2
13	FAS2	YPL231W	YPDcl2	YPDcl3	EtOHcl1	EtOHcl2	Galcl1	YPDnc1	YPDnc2	YPDnc3	YPDnc4	EtOHnc1	EtOHnc2	Galnc1	Galnc2
13		YPR045C	YPDcl2	YPDcl3	EtOHcl1	EtOHcl2	Galcl1	YPDnc1	YPDnc2	YPDnc3	YPDnc4	EtOHnc1	EtOHnc2	Galnc1	Galnc2
13	PPX1	YHR201C	YPDcl2	YPDcl3	EtOHcl1	EtOHcl2	Galcl1	YPDnc1	YPDnc2	YPDnc3	YPDnc4	EtOHnc1	EtOHnc2	Galnc1	Galnc2
13	ALG11	YNL048W	YPDcl2	YPDcl3	EtOHcl1	EtOHcl2	Galcl1	YPDnc1	YPDnc2	YPDnc3	YPDnc4	EtOHnc1	EtOHnc2	Galnc1	Galnc2
13	APS2	YJR058C	YPDcl2	YPDcl3	EtOHcl1	EtOHcl2	Galcl1	YPDnc1	YPDnc2	YPDnc3	YPDnc4	EtOHnc1	EtOHnc2	Galnc1	Galnc2

13	RPL8B	YLL045C	YPDcl2	YPDcl3	EtOHcl1	EtOHcl2	Galcl1	YPDnc1	YPDnc2	YPDnc3	YPDnc4	EtOHnc1	EtOHnc2	Galnc1	Galnc2
13	FAD1	YDL045C	YPDcl2	YPDcl3	EtOHcl1	EtOHcl2	Galcl1	YPDnc1	YPDnc2	YPDnc3	YPDnc4	EtOHnc1	EtOHnc2	Galnc1	Galnc2
13	UTP11	YKL099C	YPDcl2	YPDcl3	EtOHcl1	EtOHcl2	Galcl1	YPDnc1	YPDnc2	YPDnc3	YPDnc4	EtOHnc1	EtOHnc2	Galnc1	Galnc2
13	RRP1	YDR087C	YPDcl2	YPDcl3	EtOHcl1	EtOHcl2	Galcl1	YPDnc1	YPDnc2	YPDnc3	YPDnc4	EtOHnc1	EtOHnc2	Galnc1	Galnc2
13	ICL2	YPR006C	YPDcl2	YPDcl3	EtOHcl1	EtOHcl2	Galcl1	YPDnc1	YPDnc2	YPDnc3	YPDnc4	EtOHnc1	EtOHnc2	Galnc1	Galnc2
13	PMT4	YJR143C	YPDcl2	YPDcl3	EtOHcl1	EtOHcl2	Galcl1	YPDnc1	YPDnc2	YPDnc3	YPDnc4	EtOHnc1	EtOHnc2	Galnc1	Galnc2
13		YMR001C-A	YPDcl2	YPDcl3	EtOHcl1	EtOHcl2	Galcl1	YPDnc1	YPDnc2	YPDnc3	YPDnc4	EtOHnc1	EtOHnc2	Galnc1	Galnc2
13	SRP72	YPL210C	YPDcl2	YPDcl3	EtOHcl1	EtOHcl2	Galcl1	YPDnc1	YPDnc2	YPDnc3	YPDnc4	EtOHnc1	EtOHnc2	Galnc1	Galnc2
13	NDT80	YHR124W	YPDcl2	YPDcl3	EtOHcl1	EtOHcl2	Galcl1	YPDnc1	YPDnc2	YPDnc3	YPDnc4	EtOHnc1	EtOHnc2	Galnc1	Galnc2
13		YGR130C	YPDcl2	YPDcl3	EtOHcl1	EtOHcl2	Galcl1	YPDnc1	YPDnc2	YPDnc3	YPDnc4	EtOHnc1	EtOHnc2	Galnc1	Galnc2
13	SNF6	YHL025W	YPDcl2	YPDcl3	EtOHcl1	EtOHcl2	Galcl1	YPDnc1	YPDnc2	YPDnc3	YPDnc4	EtOHnc1	EtOHnc2	Galnc1	Galnc2
13	SSA4	YER103W	YPDcl2	YPDcl3	EtOHcl1	EtOHcl2	Galcl1	YPDnc1	YPDnc2	YPDnc3	YPDnc4	EtOHnc1	EtOHnc2	Galnc1	Galnc2
13	SED4	YCR067C	YPDcl2	YPDcl3	EtOHcl1	EtOHcl2	Galcl1	YPDnc1	YPDnc2	YPDnc3	YPDnc4	EtOHnc1	EtOHnc2	Galnc1	Galnc2
13	TSC11	YER093C	YPDcl2	YPDcl3	EtOHcl1	EtOHcl2	Galcl1	YPDnc1	YPDnc2	YPDnc3	YPDnc4	EtOHnc1	EtOHnc2	Galnc1	Galnc2
13	MED7	YOL135C	YPDcl2	YPDcl3	EtOHcl1	EtOHcl2	Galcl1	YPDnc1	YPDnc2	YPDnc3	YPDnc4	EtOHnc1	EtOHnc2	Galnc1	Galnc2
13	RSC1	YGR056W	YPDcl2	YPDcl3	EtOHcl1	EtOHcl2	Galcl1	YPDnc1	YPDnc2	YPDnc3	YPDnc4	EtOHnc1	EtOHnc2	Galnc1	Galnc2
13		YGR131W	YPDcl2	YPDcl3	EtOHcl1	EtOHcl2	Galcl1	YPDnc1	YPDnc2	YPDnc3	YPDnc4	EtOHnc1	EtOHnc2	Galnc1	Galnc2
13	ADE2	YOR128C	YPDcl2	YPDcl3	EtOHcl1	EtOHcl2	Galcl1	YPDnc1	YPDnc2	YPDnc3	YPDnc4	EtOHnc1	EtOHnc2	Galnc1	Galnc2
13	ARP3	YJR065C	YPDcl2	YPDcl3	EtOHcl1	EtOHcl2	Galcl1	YPDnc1	YPDnc2	YPDnc3	YPDnc4	EtOHnc1	EtOHnc2	Galnc1	Galnc2
13	GET1	YGL020C	YPDcl2	YPDcl3	EtOHcl1	EtOHcl2	Galcl1	YPDnc1	YPDnc2	YPDnc3	YPDnc4	EtOHnc1	EtOHnc2	Galnc1	Galnc2
13		YLR222C-A	YPDcl2	YPDcl3	EtOHcl1	EtOHcl2	Galcl1	YPDnc1	YPDnc2	YPDnc3	YPDnc4	EtOHnc1	EtOHnc2	Galnc1	Galnc2
13	PRP42	YDR235W	YPDcl2	YPDcl3	EtOHcl1	EtOHcl2	Galcl1	YPDnc1	YPDnc2	YPDnc3	YPDnc4	EtOHnc1	EtOHnc2	Galnc1	Galnc2
13	SOG2	YOR353C	YPDcl2	YPDcl3	EtOHcl1	EtOHcl2	Galcl1	YPDnc1	YPDnc2	YPDnc3	YPDnc4	EtOHnc1	EtOHnc2	Galnc1	Galnc2
13	CRN1	YLR429W	YPDcl2	YPDcl3	EtOHcl1	EtOHcl2	Galcl1	YPDnc1	YPDnc2	YPDnc3	YPDnc4	EtOHnc1	EtOHnc2	Galnc1	Galnc2
13		YGL242C	YPDcl2	YPDcl3	EtOHcl1	EtOHcl2	Galcl1	YPDnc1	YPDnc2	YPDnc3	YPDnc4	EtOHnc1	EtOHnc2	Galnc1	Galnc2
13		YDR539W	YPDcl2	YPDcl3	EtOHcl1	EtOHcl2	Galcl1	YPDnc1	YPDnc2	YPDnc3	YPDnc4	EtOHnc1	EtOHnc2	Galnc1	Galnc2
13	ELF1	YKL160W	YPDcl2	YPDcl3	EtOHcl1	EtOHcl2	Galcl1	YPDnc1	YPDnc2	YPDnc3	YPDnc4	EtOHnc1	EtOHnc2	Galnc1	Galnc2
13	UBP11	YKR098C	YPDcl2	YPDcl3	EtOHcl1	EtOHcl2	Galcl1	YPDnc1	YPDnc2	YPDnc3	YPDnc4	EtOHnc1	EtOHnc2	Galnc1	Galnc2
13	OSH7	YHR001W	YPDcl2	YPDcl3	EtOHcl1	EtOHcl2	Galcl1	YPDnc1	YPDnc2	YPDnc3	YPDnc4	EtOHnc1	EtOHnc2	Galnc1	Galnc2
13	CLB5	YPR120C	YPDcl2	YPDcl3	EtOHcl1	EtOHcl2	Galcl1	YPDnc1	YPDnc2	YPDnc3	YPDnc4	EtOHnc1	EtOHnc2	Galnc1	Galnc2
13	CIN5	YOR028C	YPDcl2	YPDcl3	EtOHcl1	EtOHcl2	Galcl1	YPDnc1	YPDnc2	YPDnc3	YPDnc4	EtOHnc1	EtOHnc2	Galnc1	Galnc2
13	SRY1	YKL218C	YPDcl2	YPDcl3	EtOHcl1	EtOHcl2	Galcl1	YPDnc1	YPDnc2	YPDnc3	YPDnc4	EtOHnc1	EtOHnc2	Galnc1	Galnc2
13		YBL065W	YPDcl2	YPDcl3	EtOHcl1	EtOHcl2	Galcl1	YPDnc1	YPDnc2	YPDnc3	YPDnc4	EtOHnc1	EtOHnc2	Galnc1	Galnc2
13	RPS11B	YBR048W	YPDcl2	YPDcl3	EtOHcl1	EtOHcl2	Galcl1	YPDnc1	YPDnc2	YPDnc3	YPDnc4	EtOHnc1	EtOHnc2	Galnc1	Galnc2

13	SUL1	YBR294W	YPDcl2	YPDcl3	EtOHcl1	EtOHcl2	Galcl1	YPDnc1	YPDnc2	YPDnc3	YPDnc4	EtOHnc1	EtOHnc2	Galnc1	Galnc2
13		YLR407W	YPDcl2	YPDcl3	EtOHcl1	EtOHcl2	Galcl1	YPDnc1	YPDnc2	YPDnc3	YPDnc4	EtOHnc1	EtOHnc2	Galnc1	Galnc2
13		YIL068W-A	YPDcl2	YPDcl3	EtOHcl1	EtOHcl2	Galcl1	YPDnc1	YPDnc2	YPDnc3	YPDnc4	EtOHnc1	EtOHnc2	Galnc1	Galnc2
13		YBR174C	YPDcl2	YPDcl3	EtOHcl1	EtOHcl2	Galcl1	YPDnc1	YPDnc2	YPDnc3	YPDnc4	EtOHnc1	EtOHnc2	Galnc1	Galnc2
13		YLR271W	YPDcl2	YPDcl3	EtOHcl1	EtOHcl2	Galcl1	YPDnc1	YPDnc2	YPDnc3	YPDnc4	EtOHnc1	EtOHnc2	Galnc1	Galnc2
13	PSD1	YNL169C	YPDcl2	YPDcl3	EtOHcl1	EtOHcl2	Galcl1	YPDnc1	YPDnc2	YPDnc3	YPDnc4	EtOHnc1	EtOHnc2	Galnc1	Galnc2
13		YKL063C	YPDcl2	YPDcl3	EtOHcl1	EtOHcl2	Galcl1	YPDnc1	YPDnc2	YPDnc3	YPDnc4	EtOHnc1	EtOHnc2	Galnc1	Galnc2
13		YIL152W	YPDcl2	YPDcl3	EtOHcl1	EtOHcl2	Galcl1	YPDnc1	YPDnc2	YPDnc3	YPDnc4	EtOHnc1	EtOHnc2	Galnc1	Galnc2
13	UTP18	YJL069C	YPDcl2	YPDcl3	EtOHcl1	EtOHcl2	Galcl1	YPDnc1	YPDnc2	YPDnc3	YPDnc4	EtOHnc1	EtOHnc2	Galnc1	Galnc2
13	GON7	YJL184W	YPDcl2	YPDcl3	EtOHcl1	EtOHcl2	Galcl1	YPDnc1	YPDnc2	YPDnc3	YPDnc4	EtOHnc1	EtOHnc2	Galnc1	Galnc2
13	GCA1	YPR115W	YPDcl2	YPDcl3	EtOHcl1	EtOHcl2	Galcl1	YPDnc1	YPDnc2	YPDnc3	YPDnc4	EtOHnc1	EtOHnc2	Galnc1	Galnc2
13	EAR1	YMR171C	YPDcl2	YPDcl3	EtOHcl1	EtOHcl2	Galcl1	YPDnc1	YPDnc2	YPDnc3	YPDnc4	EtOHnc1	EtOHnc2	Galnc1	Galnc2
13	APA2	YDR530C	YPDcl2	YPDcl3	EtOHcl1	EtOHcl2	Galcl1	YPDnc1	YPDnc2	YPDnc3	YPDnc4	EtOHnc1	EtOHnc2	Galnc1	Galnc2
13	SPC34	YKR037C	YPDcl2	YPDcl3	EtOHcl1	EtOHcl2	Galcl1	YPDnc1	YPDnc2	YPDnc3	YPDnc4	EtOHnc1	EtOHnc2	Galnc1	Galnc2
13	ATP14	YLR295C	YPDcl2	YPDcl3	EtOHcl1	EtOHcl2	Galcl1	YPDnc1	YPDnc2	YPDnc3	YPDnc4	EtOHnc1	EtOHnc2	Galnc1	Galnc2
13	KHA1	YJL094C	YPDcl2	YPDcl3	EtOHcl1	EtOHcl2	Galcl1	YPDnc1	YPDnc2	YPDnc3	YPDnc4	EtOHnc1	EtOHnc2	Galnc1	Galnc2
13	SNU13	YEL026W	YPDcl2	YPDcl3	EtOHcl1	EtOHcl2	Galcl1	YPDnc1	YPDnc2	YPDnc3	YPDnc4	EtOHnc1	EtOHnc2	Galnc1	Galnc2
13	MSS2	YDL107W	YPDcl2	YPDcl3	EtOHcl1	EtOHcl2	Galcl1	YPDnc1	YPDnc2	YPDnc3	YPDnc4	EtOHnc1	EtOHnc2	Galnc1	Galnc2
13	MAK16	YAL025C	YPDcl2	YPDcl3	EtOHcl1	EtOHcl2	Galcl1	YPDnc1	YPDnc2	YPDnc3	YPDnc4	EtOHnc1	EtOHnc2	Galnc1	Galnc2
13	PMT2	YAL023C	YPDcl2	YPDcl3	EtOHcl1	EtOHcl2	Galcl1	YPDnc1	YPDnc2	YPDnc3	YPDnc4	EtOHnc1	EtOHnc2	Galnc1	Galnc2
13	PEX17	YNL214W	YPDcl2	YPDcl3	EtOHcl1	EtOHcl2	Galcl1	YPDnc1	YPDnc2	YPDnc3	YPDnc4	EtOHnc1	EtOHnc2	Galnc1	Galnc2
13	ATG4	YNL223W	YPDcl2	YPDcl3	EtOHcl1	EtOHcl2	Galcl1	YPDnc1	YPDnc2	YPDnc3	YPDnc4	EtOHnc1	EtOHnc2	Galnc1	Galnc2
13	RPL7A	YGL076C	YPDcl2	YPDcl3	EtOHcl1	EtOHcl2	Galcl1	YPDnc1	YPDnc2	YPDnc3	YPDnc4	EtOHnc1	EtOHnc2	Galnc1	Galnc2
13		YIL100W	YPDcl2	YPDcl3	EtOHcl1	EtOHcl2	Galcl1	YPDnc1	YPDnc2	YPDnc3	YPDnc4	EtOHnc1	EtOHnc2	Galnc1	Galnc2
13	CNE1	YAL058W	YPDcl2	YPDcl3	EtOHcl1	EtOHcl2	Galcl1	YPDnc1	YPDnc2	YPDnc3	YPDnc4	EtOHnc1	EtOHnc2	Galnc1	Galnc2
13	MRPL40	YPL173W	YPDcl2	YPDcl3	EtOHcl1	EtOHcl2	Galcl1	YPDnc1	YPDnc2	YPDnc3	YPDnc4	EtOHnc1	EtOHnc2	Galnc1	Galnc2
13	PGU1	YJR153W	YPDcl2	YPDcl3	EtOHcl1	EtOHcl2	Galcl1	YPDnc1	YPDnc2	YPDnc3	YPDnc4	EtOHnc1	EtOHnc2	Galnc1	Galnc2
13	ERV41	YML067C	YPDcl2	YPDcl3	EtOHcl1	EtOHcl2	Galcl1	YPDnc1	YPDnc2	YPDnc3	YPDnc4	EtOHnc1	EtOHnc2	Galnc1	Galnc2
13	GPM1	YKL152C	YPDcl2	YPDcl3	EtOHcl1	EtOHcl2	Galcl1	YPDnc1	YPDnc2	YPDnc3	YPDnc4	EtOHnc1	EtOHnc2	Galnc1	Galnc2
13		YDR464C-A	YPDcl2	YPDcl3	EtOHcl1	EtOHcl2	Galcl1	YPDnc1	YPDnc2	YPDnc3	YPDnc4	EtOHnc1	EtOHnc2	Galnc1	Galnc2
13	ABC1	YGL119W	YPDcl2	YPDcl3	EtOHcl1	EtOHcl2	Galcl1	YPDnc1	YPDnc2	YPDnc3	YPDnc4	EtOHnc1	EtOHnc2	Galnc1	Galnc2
13	SIN4	YNL236W	YPDcl2	YPDcl3	EtOHcl1	EtOHcl2	Galcl1	YPDnc1	YPDnc2	YPDnc3	YPDnc4	EtOHnc1	EtOHnc2	Galnc1	Galnc2
13	GIM4	YEL003W	YPDcl2	YPDcl3	EtOHcl1	EtOHcl2	Galcl1	YPDnc1	YPDnc2	YPDnc3	YPDnc4	EtOHnc1	EtOHnc2	Galnc1	Galnc2
13	PHB1	YGR132C	YPDcl2	YPDcl3	EtOHcl1	EtOHcl2	Galcl1	YPDnc1	YPDnc2	YPDnc3	YPDnc4	EtOHnc1	EtOHnc2	Galnc1	Galnc2

13		YHR193C-A	YPDcl2	YPDcl3	EtOHcl1	EtOHcl2	Galcl1	YPDnc1	YPDnc2	YPDnc3	YPDnc4	EtOHnc1	EtOHnc2	Galnc1	Galnc2
13	MRT4	YKL009W	YPDcl2	YPDcl3	EtOHcl1	EtOHcl2	Galcl1	YPDnc1	YPDnc2	YPDnc3	YPDnc4	EtOHnc1	EtOHnc2	Galnc1	Galnc2
13	ATP2	YJR121W	YPDcl2	YPDcl3	EtOHcl1	EtOHcl2	Galcl1	YPDnc1	YPDnc2	YPDnc3	YPDnc4	EtOHnc1	EtOHnc2	Galnc1	Galnc2
13	ADE1	YAR015W	YPDcl2	YPDcl3	EtOHcl1	EtOHcl2	Galcl1	YPDnc1	YPDnc2	YPDnc3	YPDnc4	EtOHnc1	EtOHnc2	Galnc1	Galnc2
13	RRT7	YLL030C	YPDcl2	YPDcl3	EtOHcl1	EtOHcl2	Galcl1	YPDnc1	YPDnc2	YPDnc3	YPDnc4	EtOHnc1	EtOHnc2	Galnc1	Galnc2
13	NOP10	YHR072W-/	YPDcl2	YPDcl3	EtOHcl1	EtOHcl2	Galcl1	YPDnc1	YPDnc2	YPDnc3	YPDnc4	EtOHnc1	EtOHnc2	Galnc1	Galnc2
13	MRPS28	YDR337W	YPDcl2	YPDcl3	EtOHcl1	EtOHcl2	Galcl1	YPDnc1	YPDnc2	YPDnc3	YPDnc4	EtOHnc1	EtOHnc2	Galnc1	Galnc2
13	CBF2	YGR140W	YPDcl2	YPDcl3	EtOHcl1	EtOHcl2	Galcl1	YPDnc1	YPDnc2	YPDnc3	YPDnc4	EtOHnc1	EtOHnc2	Galnc1	Galnc2
13	PGA2	YNL149C	YPDcl2	YPDcl3	EtOHcl1	EtOHcl2	Galcl1	YPDnc1	YPDnc2	YPDnc3	YPDnc4	EtOHnc1	EtOHnc2	Galnc1	Galnc2
13	HOM2	YDR158W	YPDcl2	YPDcl3	EtOHcl1	EtOHcl2	Galcl1	YPDnc1	YPDnc2	YPDnc3	YPDnc4	EtOHnc1	EtOHnc2	Galnc1	Galnc2
13	APT1	YML022W	YPDcl2	YPDcl3	EtOHcl1	EtOHcl2	Galcl1	YPDnc1	YPDnc2	YPDnc3	YPDnc4	EtOHnc1	EtOHnc2	Galnc1	Galnc2
13	SMC4	YLR086W	YPDcl2	YPDcl3	EtOHcl1	EtOHcl2	Galcl1	YPDnc1	YPDnc2	YPDnc3	YPDnc4	EtOHnc1	EtOHnc2	Galnc1	Galnc2
13	TFP3	YPL234C	YPDcl2	YPDcl3	EtOHcl1	EtOHcl2	Galcl1	YPDnc1	YPDnc2	YPDnc3	YPDnc4	EtOHnc1	EtOHnc2	Galnc1	Galnc2
13	DEG1	YFL001W	YPDcl2	YPDcl3	EtOHcl1	EtOHcl2	Galcl1	YPDnc1	YPDnc2	YPDnc3	YPDnc4	EtOHnc1	EtOHnc2	Galnc1	Galnc2
13	MAC1	YMR021C	YPDcl2	YPDcl3	EtOHcl1	EtOHcl2	Galcl1	YPDnc1	YPDnc2	YPDnc3	YPDnc4	EtOHnc1	EtOHnc2	Galnc1	Galnc2
13	STU1	YBL034C	YPDcl2	YPDcl3	EtOHcl1	EtOHcl2	Galcl1	YPDnc1	YPDnc2	YPDnc3	YPDnc4	EtOHnc1	EtOHnc2	Galnc1	Galnc2
13		YOR032W-/	YPDcl2	YPDcl3	EtOHcl1	EtOHcl2	Galcl1	YPDnc1	YPDnc2	YPDnc3	YPDnc4	EtOHnc1	EtOHnc2	Galnc1	Galnc2
13		YIR007W	YPDcl2	YPDcl3	EtOHcl1	EtOHcl2	Galcl1	YPDnc1	YPDnc2	YPDnc3	YPDnc4	EtOHnc1	EtOHnc2	Galnc1	Galnc2
13		YDR003W-/	YPDcl2	YPDcl3	EtOHcl1	EtOHcl2	Galcl1	YPDnc1	YPDnc2	YPDnc3	YPDnc4	EtOHnc1	EtOHnc2	Galnc1	Galnc2
13	SMY1	YKL079W	YPDcl2	YPDcl3	EtOHcl1	EtOHcl2	Galcl1	YPDnc1	YPDnc2	YPDnc3	YPDnc4	EtOHnc1	EtOHnc2	Galnc1	Galnc2
13	BPT1	YLL015W	YPDcl2	YPDcl3	EtOHcl1	EtOHcl2	Galcl1	YPDnc1	YPDnc2	YPDnc3	YPDnc4	EtOHnc1	EtOHnc2	Galnc1	Galnc2
13	MCR1	YKL150W	YPDcl2	YPDcl3	EtOHcl1	EtOHcl2	Galcl1	YPDnc1	YPDnc2	YPDnc3	YPDnc4	EtOHnc1	EtOHnc2	Galnc1	Galnc2
13	SIR4	YDR227W	YPDcl2	YPDcl3	EtOHcl1	EtOHcl2	Galcl1	YPDnc1	YPDnc2	YPDnc3	YPDnc4	EtOHnc1	EtOHnc2	Galnc1	Galnc2
13	SSS1	YDR086C	YPDcl2	YPDcl3	EtOHcl1	EtOHcl2	Galcl1	YPDnc1	YPDnc2	YPDnc3	YPDnc4	EtOHnc1	EtOHnc2	Galnc1	Galnc2
13	CRD1	YDL142C	YPDcl2	YPDcl3	EtOHcl1	EtOHcl2	Galcl1	YPDnc1	YPDnc2	YPDnc3	YPDnc4	EtOHnc1	EtOHnc2	Galnc1	Galnc2
13	ASI2	YNL159C	YPDcl2	YPDcl3	EtOHcl1	EtOHcl2	Galcl1	YPDnc1	YPDnc2	YPDnc3	YPDnc4	EtOHnc1	EtOHnc2	Galnc1	Galnc2
13	MTC4	YBR255W	YPDcl2	YPDcl3	EtOHcl1	EtOHcl2	Galcl1	YPDnc1	YPDnc2	YPDnc3	YPDnc4	EtOHnc1	EtOHnc2	Galnc1	Galnc2
13	SPI1	YER150W	YPDcl2	YPDcl3	EtOHcl1	EtOHcl2	Galcl1	YPDnc1	YPDnc2	YPDnc3	YPDnc4	EtOHnc1	EtOHnc2	Galnc1	Galnc2
13	EBP2	YKL172W	YPDcl2	YPDcl3	EtOHcl1	EtOHcl2	Galcl1	YPDnc1	YPDnc2	YPDnc3	YPDnc4	EtOHnc1	EtOHnc2	Galnc1	Galnc2
13	RGD2	YFL047W	YPDcl2	YPDcl3	EtOHcl1	EtOHcl2	Galcl1	YPDnc1	YPDnc2	YPDnc3	YPDnc4	EtOHnc1	EtOHnc2	Galnc1	Galnc2
13		YDR327W	YPDcl2	YPDcl3	EtOHcl1	EtOHcl2	Galcl1	YPDnc1	YPDnc2	YPDnc3	YPDnc4	EtOHnc1	EtOHnc2	Galnc1	Galnc2
13		YPL071C	YPDcl2	YPDcl3	EtOHcl1	EtOHcl2	Galcl1	YPDnc1	YPDnc2	YPDnc3	YPDnc4	EtOHnc1	EtOHnc2	Galnc1	Galnc2
13	TAF13	YML098W	YPDcl2	YPDcl3	EtOHcl1	EtOHcl2	Galcl1	YPDnc1	YPDnc2	YPDnc3	YPDnc4	EtOHnc1	EtOHnc2	Galnc1	Galnc2
13	HYR1	YIR037W	YPDcl2	YPDcl3	EtOHcl1	EtOHcl2	Galcl1	YPDnc1	YPDnc2	YPDnc3	YPDnc4	EtOHnc1	EtOHnc2	Galnc1	Galnc2

13	TMS1	YDR105C	YPDcl2	YPDcl3	EtOHcl1	EtOHcl2	Galcl1	YPDnc1	YPDnc2	YPDnc3	YPDnc4	EtOHnc1	EtOHnc2	Galnc1	Galnc2
13		YLR050C	YPDcl2	YPDcl3	EtOHcl1	EtOHcl2	Galcl1	YPDnc1	YPDnc2	YPDnc3	YPDnc4	EtOHnc1	EtOHnc2	Galnc1	Galnc2
13	HPA2	YPR193C	YPDcl2	YPDcl3	EtOHcl1	EtOHcl2	Galcl1	YPDnc1	YPDnc2	YPDnc3	YPDnc4	EtOHnc1	EtOHnc2	Galnc1	Galnc2
13	MSS1	YMR023C	YPDcl2	YPDcl3	EtOHcl1	EtOHcl2	Galcl1	YPDnc1	YPDnc2	YPDnc3	YPDnc4	EtOHnc1	EtOHnc2	Galnc1	Galnc2
13	RRN11	YML043C	YPDcl2	YPDcl3	EtOHcl1	EtOHcl2	Galcl1	YPDnc1	YPDnc2	YPDnc3	YPDnc4	EtOHnc1	EtOHnc2	Galnc1	Galnc2
13	ASH1	YKL185W	YPDcl2	YPDcl3	EtOHcl1	EtOHcl2	Galcl1	YPDnc1	YPDnc2	YPDnc3	YPDnc4	EtOHnc1	EtOHnc2	Galnc1	Galnc2
13	RPS29B	YDL061C	YPDcl2	YPDcl3	EtOHcl1	EtOHcl2	Galcl1	YPDnc1	YPDnc2	YPDnc3	YPDnc4	EtOHnc1	EtOHnc2	Galnc1	Galnc2
13	MET12	YPL023C	YPDcl2	YPDcl3	EtOHcl1	EtOHcl2	Galcl1	YPDnc1	YPDnc2	YPDnc3	YPDnc4	EtOHnc1	EtOHnc2	Galnc1	Galnc2
13	SGO1	YOR073W	YPDcl2	YPDcl3	EtOHcl1	EtOHcl2	Galcl1	YPDnc1	YPDnc2	YPDnc3	YPDnc4	EtOHnc1	EtOHnc2	Galnc1	Galnc2
13		YDR026C	YPDcl2	YPDcl3	EtOHcl1	EtOHcl2	Galcl1	YPDnc1	YPDnc2	YPDnc3	YPDnc4	EtOHnc1	EtOHnc2	Galnc1	Galnc2
13	SWP1	YMR149W	YPDcl2	YPDcl3	EtOHcl1	EtOHcl2	Galcl1	YPDnc1	YPDnc2	YPDnc3	YPDnc4	EtOHnc1	EtOHnc2	Galnc1	Galnc2
13	MRC1	YCL061C	YPDcl2	YPDcl3	EtOHcl1	EtOHcl2	Galcl1	YPDnc1	YPDnc2	YPDnc3	YPDnc4	EtOHnc1	EtOHnc2	Galnc1	Galnc2
13		YIL001W	YPDcl2	YPDcl3	EtOHcl1	EtOHcl2	Galcl1	YPDnc1	YPDnc2	YPDnc3	YPDnc4	EtOHnc1	EtOHnc2	Galnc1	Galnc2
13		YDL027C	YPDcl2	YPDcl3	EtOHcl1	EtOHcl2	Galcl1	YPDnc1	YPDnc2	YPDnc3	YPDnc4	EtOHnc1	EtOHnc2	Galnc1	Galnc2
13	CRC1	YOR100C	YPDcl2	YPDcl3	EtOHcl1	EtOHcl2	Galcl1	YPDnc1	YPDnc2	YPDnc3	YPDnc4	EtOHnc1	EtOHnc2	Galnc1	Galnc2
13	IRC24	YIR036C	YPDcl2	YPDcl3	EtOHcl1	EtOHcl2	Galcl1	YPDnc1	YPDnc2	YPDnc3	YPDnc4	EtOHnc1	EtOHnc2	Galnc1	Galnc2
13		YBL107C	YPDcl2	YPDcl3	EtOHcl1	EtOHcl2	Galcl1	YPDnc1	YPDnc2	YPDnc3	YPDnc4	EtOHnc1	EtOHnc2	Galnc1	Galnc2
13	RRT1	YBL048W	YPDcl2	YPDcl3	EtOHcl1	EtOHcl2	Galcl1	YPDnc1	YPDnc2	YPDnc3	YPDnc4	EtOHnc1	EtOHnc2	Galnc1	Galnc2
13	MNL1	YHR204W	YPDcl2	YPDcl3	EtOHcl1	EtOHcl2	Galcl1	YPDnc1	YPDnc2	YPDnc3	YPDnc4	EtOHnc1	EtOHnc2	Galnc1	Galnc2
13	ROG1	YGL144C	YPDcl2	YPDcl3	EtOHcl1	EtOHcl2	Galcl1	YPDnc1	YPDnc2	YPDnc3	YPDnc4	EtOHnc1	EtOHnc2	Galnc1	Galnc2
13		YFL042C	YPDcl2	YPDcl3	EtOHcl1	EtOHcl2	Galcl1	YPDnc1	YPDnc2	YPDnc3	YPDnc4	EtOHnc1	EtOHnc2	Galnc1	Galnc2
13	TOS6	YNL300W	YPDcl2	YPDcl3	EtOHcl1	EtOHcl2	Galcl1	YPDnc1	YPDnc2	YPDnc3	YPDnc4	EtOHnc1	EtOHnc2	Galnc1	Galnc2
13	AIM44	YPL158C	YPDcl2	YPDcl3	EtOHcl1	EtOHcl2	Galcl1	YPDnc1	YPDnc2	YPDnc3	YPDnc4	EtOHnc1	EtOHnc2	Galnc1	Galnc2
13		YFR009W-A	YPDcl2	YPDcl3	EtOHcl1	EtOHcl2	Galcl1	YPDnc1	YPDnc2	YPDnc3	YPDnc4	EtOHnc1	EtOHnc2	Galnc1	Galnc2
13		YKL105C	YPDcl2	YPDcl3	EtOHcl1	EtOHcl2	Galcl1	YPDnc1	YPDnc2	YPDnc3	YPDnc4	EtOHnc1	EtOHnc2	Galnc1	Galnc2
13	OAZ1	YPL052W	YPDcl2	YPDcl3	EtOHcl1	EtOHcl2	Galcl1	YPDnc1	YPDnc2	YPDnc3	YPDnc4	EtOHnc1	EtOHnc2	Galnc1	Galnc2
13		YBR241C	YPDcl2	YPDcl3	EtOHcl1	EtOHcl2	Galcl1	YPDnc1	YPDnc2	YPDnc3	YPDnc4	EtOHnc1	EtOHnc2	Galnc1	Galnc2
13	NUP116	YMR047C	YPDcl2	YPDcl3	EtOHcl1	EtOHcl2	Galcl1	YPDnc1	YPDnc2	YPDnc3	YPDnc4	EtOHnc1	EtOHnc2	Galnc1	Galnc2
13		YGR102C	YPDcl2	YPDcl3	EtOHcl1	EtOHcl2	Galcl1	YPDnc1	YPDnc2	YPDnc3	YPDnc4	EtOHnc1	EtOHnc2	Galnc1	Galnc2
13	RPS26B	YER131W	YPDcl2	YPDcl3	EtOHcl1	EtOHcl2	Galcl1	YPDnc1	YPDnc2	YPDnc3	YPDnc4	EtOHnc1	EtOHnc2	Galnc1	Galnc2
13	MSB3	YNL293W	YPDcl2	YPDcl3	EtOHcl1	EtOHcl2	Galcl1	YPDnc1	YPDnc2	YPDnc3	YPDnc4	EtOHnc1	EtOHnc2	Galnc1	Galnc2
13	TRM112	YNR046W	YPDcl2	YPDcl3	EtOHcl1	EtOHcl2	Galcl1	YPDnc1	YPDnc2	YPDnc3	YPDnc4	EtOHnc1	EtOHnc2	Galnc1	Galnc2
13	CDC3	YLR314C	YPDcl2	YPDcl3	EtOHcl1	EtOHcl2	Galcl1	YPDnc1	YPDnc2	YPDnc3	YPDnc4	EtOHnc1	EtOHnc2	Galnc1	Galnc2
13	ARC19	YKL013C	YPDcl2	YPDcl3	EtOHcl1	EtOHcl2	Galcl1	YPDnc1	YPDnc2	YPDnc3	YPDnc4	EtOHnc1	EtOHnc2	Galnc1	Galnc2

13	PET122	YER153C	YPDcl2	YPDcl3	EtOHcl1	EtOHcl2	Galcl1	YPDnc1	YPDnc2	YPDnc3	YPDnc4	EtOHnc1	EtOHnc2	Galnc1	Galnc2
13		YLL044W	YPDcl2	YPDcl3	EtOHcl1	EtOHcl2	Galcl1	YPDnc1	YPDnc2	YPDnc3	YPDnc4	EtOHnc1	EtOHnc2	Galnc1	Galnc2
13		YAR035C-A	YPDcl2	YPDcl3	EtOHcl1	EtOHcl2	Galcl1	YPDnc1	YPDnc2	YPDnc3	YPDnc4	EtOHnc1	EtOHnc2	Galnc1	Galnc2
13	DBP9	YLR276C	YPDcl2	YPDcl3	EtOHcl1	EtOHcl2	Galcl1	YPDnc1	YPDnc2	YPDnc3	YPDnc4	EtOHnc1	EtOHnc2	Galnc1	Galnc2
13	HOL1	YNR055C	YPDcl2	YPDcl3	EtOHcl1	EtOHcl2	Galcl1	YPDnc1	YPDnc2	YPDnc3	YPDnc4	EtOHnc1	EtOHnc2	Galnc1	Galnc2
13	CLB3	YDL155W	YPDcl2	YPDcl3	EtOHcl1	EtOHcl2	Galcl1	YPDnc1	YPDnc2	YPDnc3	YPDnc4	EtOHnc1	EtOHnc2	Galnc1	Galnc2
13	VPS28	YPL065W	YPDcl2	YPDcl3	EtOHcl1	EtOHcl2	Galcl1	YPDnc1	YPDnc2	YPDnc3	YPDnc4	EtOHnc1	EtOHnc2	Galnc1	Galnc2
13	VMA21	YGR105W	YPDcl2	YPDcl3	EtOHcl1	EtOHcl2	Galcl1	YPDnc1	YPDnc2	YPDnc3	YPDnc4	EtOHnc1	EtOHnc2	Galnc1	Galnc2
13	NUP82	YJL061W	YPDcl2	YPDcl3	EtOHcl1	EtOHcl2	Galcl1	YPDnc1	YPDnc2	YPDnc3	YPDnc4	EtOHnc1	EtOHnc2	Galnc1	Galnc2
13	PCP1	YGR101W	YPDcl2	YPDcl3	EtOHcl1	EtOHcl2	Galcl1	YPDnc1	YPDnc2	YPDnc3	YPDnc4	EtOHnc1	EtOHnc2	Galnc1	Galnc2
13	SPO1	YNL012W	YPDcl2	YPDcl3	EtOHcl1	EtOHcl2	Galcl1	YPDnc1	YPDnc2	YPDnc3	YPDnc4	EtOHnc1	EtOHnc2	Galnc1	Galnc2
13	FAU1	YER183C	YPDcl2	YPDcl3	EtOHcl1	EtOHcl2	Galcl1	YPDnc1	YPDnc2	YPDnc3	YPDnc4	EtOHnc1	EtOHnc2	Galnc1	Galnc2
13	SEN2	YLR105C	YPDcl2	YPDcl3	EtOHcl1	EtOHcl2	Galcl1	YPDnc1	YPDnc2	YPDnc3	YPDnc4	EtOHnc1	EtOHnc2	Galnc1	Galnc2
13	CTK2	YJL006C	YPDcl2	YPDcl3	EtOHcl1	EtOHcl2	Galcl1	YPDnc1	YPDnc2	YPDnc3	YPDnc4	EtOHnc1	EtOHnc2	Galnc1	Galnc2
13		YGR250C	YPDcl2	YPDcl3	EtOHcl1	EtOHcl2	Galcl1	YPDnc1	YPDnc2	YPDnc3	YPDnc4	EtOHnc1	EtOHnc2	Galnc1	Galnc2
13	ACN9	YDR511W	YPDcl2	YPDcl3	EtOHcl1	EtOHcl2	Galcl1	YPDnc1	YPDnc2	YPDnc3	YPDnc4	EtOHnc1	EtOHnc2	Galnc1	Galnc2
13		YDL032W	YPDcl2	YPDcl3	EtOHcl1	EtOHcl2	Galcl1	YPDnc1	YPDnc2	YPDnc3	YPDnc4	EtOHnc1	EtOHnc2	Galnc1	Galnc2
13	NOG2	YNR053C	YPDcl2	YPDcl3	EtOHcl1	EtOHcl2	Galcl1	YPDnc1	YPDnc2	YPDnc3	YPDnc4	EtOHnc1	EtOHnc2	Galnc1	Galnc2
13	BNA6	YFR047C	YPDcl2	YPDcl3	EtOHcl1	EtOHcl2	Galcl1	YPDnc1	YPDnc2	YPDnc3	YPDnc4	EtOHnc1	EtOHnc2	Galnc1	Galnc2
13	PAT1	YCR077C	YPDcl2	YPDcl3	EtOHcl1	EtOHcl2	Galcl1	YPDnc1	YPDnc2	YPDnc3	YPDnc4	EtOHnc1	EtOHnc2	Galnc1	Galnc2
13	RRI2	YOL117W	YPDcl2	YPDcl3	EtOHcl1	EtOHcl2	Galcl1	YPDnc1	YPDnc2	YPDnc3	YPDnc4	EtOHnc1	EtOHnc2	Galnc1	Galnc2
13	CUE2	YKL090W	YPDcl2	YPDcl3	EtOHcl1	EtOHcl2	Galcl1	YPDnc1	YPDnc2	YPDnc3	YPDnc4	EtOHnc1	EtOHnc2	Galnc1	Galnc2
13		YGL081W	YPDcl2	YPDcl3	EtOHcl1	EtOHcl2	Galcl1	YPDnc1	YPDnc2	YPDnc3	YPDnc4	EtOHnc1	EtOHnc2	Galnc1	Galnc2
13	MMP1	YLL061W	YPDcl2	YPDcl3	EtOHcl1	EtOHcl2	Galcl1	YPDnc1	YPDnc2	YPDnc3	YPDnc4	EtOHnc1	EtOHnc2	Galnc1	Galnc2
13	ERG12	YMR208W	YPDcl2	YPDcl3	EtOHcl1	EtOHcl2	Galcl1	YPDnc1	YPDnc2	YPDnc3	YPDnc4	EtOHnc1	EtOHnc2	Galnc1	Galnc2
13	HRD3	YLR207W	YPDcl2	YPDcl3	EtOHcl1	EtOHcl2	Galcl1	YPDnc1	YPDnc2	YPDnc3	YPDnc4	EtOHnc1	EtOHnc2	Galnc1	Galnc2
13	ARE2	YNR019W	YPDcl2	YPDcl3	EtOHcl1	EtOHcl2	Galcl1	YPDnc1	YPDnc2	YPDnc3	YPDnc4	EtOHnc1	EtOHnc2	Galnc1	Galnc2
13	RVB2	YPL235W	YPDcl2	YPDcl3	EtOHcl1	EtOHcl2	Galcl1	YPDnc1	YPDnc2	YPDnc3	YPDnc4	EtOHnc1	EtOHnc2	Galnc1	Galnc2
13	MUD1	YBR119W	YPDcl2	YPDcl3	EtOHcl1	EtOHcl2	Galcl1	YPDnc1	YPDnc2	YPDnc3	YPDnc4	EtOHnc1	EtOHnc2	Galnc1	Galnc2
13	WWM1	YFL010C	YPDcl2	YPDcl3	EtOHcl1	EtOHcl2	Galcl1	YPDnc1	YPDnc2	YPDnc3	YPDnc4	EtOHnc1	EtOHnc2	Galnc1	Galnc2
13	ATG19	YOL082W	YPDcl2	YPDcl3	EtOHcl1	EtOHcl2	Galcl1	YPDnc1	YPDnc2	YPDnc3	YPDnc4	EtOHnc1	EtOHnc2	Galnc1	Galnc2
13	SDC1	YDR469W	YPDcl2	YPDcl3	EtOHcl1	EtOHcl2	Galcl1	YPDnc1	YPDnc2	YPDnc3	YPDnc4	EtOHnc1	EtOHnc2	Galnc1	Galnc2
13	SPO7	YAL009W	YPDcl2	YPDcl3	EtOHcl1	EtOHcl2	Galcl1	YPDnc1	YPDnc2	YPDnc3	YPDnc4	EtOHnc1	EtOHnc2	Galnc1	Galnc2
13	YAE1	YJR067C	YPDcl2	YPDcl3	EtOHcl1	EtOHcl2	Galcl1	YPDnc1	YPDnc2	YPDnc3	YPDnc4	EtOHnc1	EtOHnc2	Galnc1	Galnc2

13	NEW1	YPL226W	YPDcl2	YPDcl3	EtOHcl1	EtOHcl2	Galcl1	YPDnc1	YPDnc2	YPDnc3	YPDnc4	EtOHnc1	EtOHnc2	Galnc1	Galnc2
13	DYN2	YDR424C	YPDcl2	YPDcl3	EtOHcl1	EtOHcl2	Galcl1	YPDnc1	YPDnc2	YPDnc3	YPDnc4	EtOHnc1	EtOHnc2	Galnc1	Galnc2
13	NIC96	YFR002W	YPDcl2	YPDcl3	EtOHcl1	EtOHcl2	Galcl1	YPDnc1	YPDnc2	YPDnc3	YPDnc4	EtOHnc1	EtOHnc2	Galnc1	Galnc2
13		YFR017C	YPDcl2	YPDcl3	EtOHcl1	EtOHcl2	Galcl1	YPDnc1	YPDnc2	YPDnc3	YPDnc4	EtOHnc1	EtOHnc2	Galnc1	Galnc2
13		YAR023C	YPDcl2	YPDcl3	EtOHcl1	EtOHcl2	Galcl1	YPDnc1	YPDnc2	YPDnc3	YPDnc4	EtOHnc1	EtOHnc2	Galnc1	Galnc2
13	CRP1	YHR146W	YPDcl2	YPDcl3	EtOHcl1	EtOHcl2	Galcl1	YPDnc1	YPDnc2	YPDnc3	YPDnc4	EtOHnc1	EtOHnc2	Galnc1	Galnc2
13	TFB2	YPL122C	YPDcl2	YPDcl3	EtOHcl1	EtOHcl2	Galcl1	YPDnc1	YPDnc2	YPDnc3	YPDnc4	EtOHnc1	EtOHnc2	Galnc1	Galnc2
13		YHL030W-A	YPDcl2	YPDcl3	EtOHcl1	EtOHcl2	Galcl1	YPDnc1	YPDnc2	YPDnc3	YPDnc4	EtOHnc1	EtOHnc2	Galnc1	Galnc2
13		YPL222C-A	YPDcl2	YPDcl3	EtOHcl1	EtOHcl2	Galcl1	YPDnc1	YPDnc2	YPDnc3	YPDnc4	EtOHnc1	EtOHnc2	Galnc1	Galnc2
13		YER186C	YPDcl2	YPDcl3	EtOHcl1	EtOHcl2	Galcl1	YPDnc1	YPDnc2	YPDnc3	YPDnc4	EtOHnc1	EtOHnc2	Galnc1	Galnc2
13	GPG1	YGL121C	YPDcl2	YPDcl3	EtOHcl1	EtOHcl2	Galcl1	YPDnc1	YPDnc2	YPDnc3	YPDnc4	EtOHnc1	EtOHnc2	Galnc1	Galnc2
13	HIR2	YOR038C	YPDcl2	YPDcl3	EtOHcl1	EtOHcl2	Galcl1	YPDnc1	YPDnc2	YPDnc3	YPDnc4	EtOHnc1	EtOHnc2	Galnc1	Galnc2
13	BUD23	YCR047C	YPDcl2	YPDcl3	EtOHcl1	EtOHcl2	Galcl1	YPDnc1	YPDnc2	YPDnc3	YPDnc4	EtOHnc1	EtOHnc2	Galnc1	Galnc2
13	ESS1	YJR017C	YPDcl2	YPDcl3	EtOHcl1	EtOHcl2	Galcl1	YPDnc1	YPDnc2	YPDnc3	YPDnc4	EtOHnc1	EtOHnc2	Galnc1	Galnc2
13	NAP1	YKR048C	YPDcl2	YPDcl3	EtOHcl1	EtOHcl2	Galcl1	YPDnc1	YPDnc2	YPDnc3	YPDnc4	EtOHnc1	EtOHnc2	Galnc1	Galnc2
13	PIS1	YPR113W	YPDcl2	YPDcl3	EtOHcl1	EtOHcl2	Galcl1	YPDnc1	YPDnc2	YPDnc3	YPDnc4	EtOHnc1	EtOHnc2	Galnc1	Galnc2
13		YOL085W-A	YPDcl2	YPDcl3	EtOHcl1	EtOHcl2	Galcl1	YPDnc1	YPDnc2	YPDnc3	YPDnc4	EtOHnc1	EtOHnc2	Galnc1	Galnc2
13		YMR099C	YPDcl2	YPDcl3	EtOHcl1	EtOHcl2	Galcl1	YPDnc1	YPDnc2	YPDnc3	YPDnc4	EtOHnc1	EtOHnc2	Galnc1	Galnc2
13	COG2	YGR120C	YPDcl2	YPDcl3	EtOHcl1	EtOHcl2	Galcl1	YPDnc1	YPDnc2	YPDnc3	YPDnc4	EtOHnc1	EtOHnc2	Galnc1	Galnc2
13	CAD1	YDR423C	YPDcl2	YPDcl3	EtOHcl1	EtOHcl2	Galcl1	YPDnc1	YPDnc2	YPDnc3	YPDnc4	EtOHnc1	EtOHnc2	Galnc1	Galnc2
13	MMR1	YLR190W	YPDcl2	YPDcl3	EtOHcl1	EtOHcl2	Galcl1	YPDnc1	YPDnc2	YPDnc3	YPDnc4	EtOHnc1	EtOHnc2	Galnc1	Galnc2
13	TAF3	YPL011C	YPDcl2	YPDcl3	EtOHcl1	EtOHcl2	Galcl1	YPDnc1	YPDnc2	YPDnc3	YPDnc4	EtOHnc1	EtOHnc2	Galnc1	Galnc2
13	AMS1	YGL156W	YPDcl2	YPDcl3	EtOHcl1	EtOHcl2	Galcl1	YPDnc1	YPDnc2	YPDnc3	YPDnc4	EtOHnc1	EtOHnc2	Galnc1	Galnc2
13	SAC1	YKL212W	YPDcl2	YPDcl3	EtOHcl1	EtOHcl2	Galcl1	YPDnc1	YPDnc2	YPDnc3	YPDnc4	EtOHnc1	EtOHnc2	Galnc1	Galnc2
13	MOT1	YPL082C	YPDcl2	YPDcl3	EtOHcl1	EtOHcl2	Galcl1	YPDnc1	YPDnc2	YPDnc3	YPDnc4	EtOHnc1	EtOHnc2	Galnc1	Galnc2
13	ECM29	YHL030W	YPDcl2	YPDcl3	EtOHcl1	EtOHcl2	Galcl1	YPDnc1	YPDnc2	YPDnc3	YPDnc4	EtOHnc1	EtOHnc2	Galnc1	Galnc2
13	MIH1	YMR036C	YPDcl2	YPDcl3	EtOHcl1	EtOHcl2	Galcl1	YPDnc1	YPDnc2	YPDnc3	YPDnc4	EtOHnc1	EtOHnc2	Galnc1	Galnc2
13		YDR352W	YPDcl2	YPDcl3	EtOHcl1	EtOHcl2	Galcl1	YPDnc1	YPDnc2	YPDnc3	YPDnc4	EtOHnc1	EtOHnc2	Galnc1	Galnc2
13		YCR025C	YPDcl2	YPDcl3	EtOHcl1	EtOHcl2	Galcl1	YPDnc1	YPDnc2	YPDnc3	YPDnc4	EtOHnc1	EtOHnc2	Galnc1	Galnc2
13	PTC5	YOR090C	YPDcl2	YPDcl3	EtOHcl1	EtOHcl2	Galcl1	YPDnc1	YPDnc2	YPDnc3	YPDnc4	EtOHnc1	EtOHnc2	Galnc1	Galnc2
13	NPR1	YNL183C	YPDcl2	YPDcl3	EtOHcl1	EtOHcl2	Galcl1	YPDnc1	YPDnc2	YPDnc3	YPDnc4	EtOHnc1	EtOHnc2	Galnc1	Galnc2
13	SIP1	YDR422C	YPDcl2	YPDcl3	EtOHcl1	EtOHcl2	Galcl1	YPDnc1	YPDnc2	YPDnc3	YPDnc4	EtOHnc1	EtOHnc2	Galnc1	Galnc2
13	RIM1	YCR028C-A	YPDcl2	YPDcl3	EtOHcl1	EtOHcl2	Galcl1	YPDnc1	YPDnc2	YPDnc3	YPDnc4	EtOHnc1	EtOHnc2	Galnc1	Galnc2
13	MNT3	YIL014W	YPDcl2	YPDcl3	EtOHcl1	EtOHcl2	Galcl1	YPDnc1	YPDnc2	YPDnc3	YPDnc4	EtOHnc1	EtOHnc2	Galnc1	Galnc2

13	PEX30	YLR324W	YPDcl2	YPDcl3	EtOHcl1	EtOHcl2	Galcl1	YPDnc1	YPDnc2	YPDnc3	YPDnc4	EtOHnc1	EtOHnc2	Galnc1	Galnc2
13	STE23	YLR389C	YPDcl2	YPDcl3	EtOHcl1	EtOHcl2	Galcl1	YPDnc1	YPDnc2	YPDnc3	YPDnc4	EtOHnc1	EtOHnc2	Galnc1	Galnc2
13	YIF1	YNL263C	YPDcl2	YPDcl3	EtOHcl1	EtOHcl2	Galcl1	YPDnc1	YPDnc2	YPDnc3	YPDnc4	EtOHnc1	EtOHnc2	Galnc1	Galnc2
13	CSR2	YPR030W	YPDcl2	YPDcl3	EtOHcl1	EtOHcl2	Galcl1	YPDnc1	YPDnc2	YPDnc3	YPDnc4	EtOHnc1	EtOHnc2	Galnc1	Galnc2
13	SPO22	YIL073C	YPDcl2	YPDcl3	EtOHcl1	EtOHcl2	Galcl1	YPDnc1	YPDnc2	YPDnc3	YPDnc4	EtOHnc1	EtOHnc2	Galnc1	Galnc2
13	SDH1	YKL148C	YPDcl2	YPDcl3	EtOHcl1	EtOHcl2	Galcl1	YPDnc1	YPDnc2	YPDnc3	YPDnc4	EtOHnc1	EtOHnc2	Galnc1	Galnc2
13	TPN1	YGL186C	YPDcl2	YPDcl3	EtOHcl1	EtOHcl2	Galcl1	YPDnc1	YPDnc2	YPDnc3	YPDnc4	EtOHnc1	EtOHnc2	Galnc1	Galnc2
13	RAD4	YER162C	YPDcl2	YPDcl3	EtOHcl1	EtOHcl2	Galcl1	YPDnc1	YPDnc2	YPDnc3	YPDnc4	EtOHnc1	EtOHnc2	Galnc1	Galnc2
13	FIN1	YDR130C	YPDcl2	YPDcl3	EtOHcl1	EtOHcl2	Galcl1	YPDnc1	YPDnc2	YPDnc3	YPDnc4	EtOHnc1	EtOHnc2	Galnc1	Galnc2
13	RCR1	YBR005W	YPDcl2	YPDcl3	EtOHcl1	EtOHcl2	Galcl1	YPDnc1	YPDnc2	YPDnc3	YPDnc4	EtOHnc1	EtOHnc2	Galnc1	Galnc2
13	TOA2	YKL058W	YPDcl2	YPDcl3	EtOHcl1	EtOHcl2	Galcl1	YPDnc1	YPDnc2	YPDnc3	YPDnc4	EtOHnc1	EtOHnc2	Galnc1	Galnc2
13		YAL019W-A	YPDcl2	YPDcl3	EtOHcl1	EtOHcl2	Galcl1	YPDnc1	YPDnc2	YPDnc3	YPDnc4	EtOHnc1	EtOHnc2	Galnc1	Galnc2
13	APL1	YJR005W	YPDcl2	YPDcl3	EtOHcl1	EtOHcl2	Galcl1	YPDnc1	YPDnc2	YPDnc3	YPDnc4	EtOHnc1	EtOHnc2	Galnc1	Galnc2
13	EAF3	YPR023C	YPDcl2	YPDcl3	EtOHcl1	EtOHcl2	Galcl1	YPDnc1	YPDnc2	YPDnc3	YPDnc4	EtOHnc1	EtOHnc2	Galnc1	Galnc2
13	POP8	YBL018C	YPDcl2	YPDcl3	EtOHcl1	EtOHcl2	Galcl1	YPDnc1	YPDnc2	YPDnc3	YPDnc4	EtOHnc1	EtOHnc2	Galnc1	Galnc2
13	GUT2	YIL155C	YPDcl2	YPDcl3	EtOHcl1	EtOHcl2	Galcl1	YPDnc1	YPDnc2	YPDnc3	YPDnc4	EtOHnc1	EtOHnc2	Galnc1	Galnc2
13	PMT3	YOR321W	YPDcl2	YPDcl3	EtOHcl1	EtOHcl2	Galcl1	YPDnc1	YPDnc2	YPDnc3	YPDnc4	EtOHnc1	EtOHnc2	Galnc1	Galnc2
13	RPS4B	YHR203C	YPDcl2	YPDcl3	EtOHcl1	EtOHcl2	Galcl1	YPDnc1	YPDnc2	YPDnc3	YPDnc4	EtOHnc1	EtOHnc2	Galnc1	Galnc2
13	TDA7	YNL176C	YPDcl2	YPDcl3	EtOHcl1	EtOHcl2	Galcl1	YPDnc1	YPDnc2	YPDnc3	YPDnc4	EtOHnc1	EtOHnc2	Galnc1	Galnc2
13		YIL096C	YPDcl2	YPDcl3	EtOHcl1	EtOHcl2	Galcl1	YPDnc1	YPDnc2	YPDnc3	YPDnc4	EtOHnc1	EtOHnc2	Galnc1	Galnc2
13	TFC6	YDR362C	YPDcl2	YPDcl3	EtOHcl1	EtOHcl2	Galcl1	YPDnc1	YPDnc2	YPDnc3	YPDnc4	EtOHnc1	EtOHnc2	Galnc1	Galnc2
13	SWE1	YJL187C	YPDcl2	YPDcl3	EtOHcl1	EtOHcl2	Galcl1	YPDnc1	YPDnc2	YPDnc3	YPDnc4	EtOHnc1	EtOHnc2	Galnc1	Galnc2
13	PCT1	YGR202C	YPDcl2	YPDcl3	EtOHcl1	EtOHcl2	Galcl1	YPDnc1	YPDnc2	YPDnc3	YPDnc4	EtOHnc1	EtOHnc2	Galnc1	Galnc2
13		YDR056C	YPDcl2	YPDcl3	EtOHcl1	EtOHcl2	Galcl1	YPDnc1	YPDnc2	YPDnc3	YPDnc4	EtOHnc1	EtOHnc2	Galnc1	Galnc2
13	OSH2	YDL019C	YPDcl2	YPDcl3	EtOHcl1	EtOHcl2	Galcl1	YPDnc1	YPDnc2	YPDnc3	YPDnc4	EtOHnc1	EtOHnc2	Galnc1	Galnc2
13		YNL144W-A	YPDcl2	YPDcl3	EtOHcl1	EtOHcl2	Galcl1	YPDnc1	YPDnc2	YPDnc3	YPDnc4	EtOHnc1	EtOHnc2	Galnc1	Galnc2
13	MIM1	YOL026C	YPDcl2	YPDcl3	EtOHcl1	EtOHcl2	Galcl1	YPDnc1	YPDnc2	YPDnc3	YPDnc4	EtOHnc1	EtOHnc2	Galnc1	Galnc2
13	RPS19A	YOL121C	YPDcl2	YPDcl3	EtOHcl1	EtOHcl2	Galcl1	YPDnc1	YPDnc2	YPDnc3	YPDnc4	EtOHnc1	EtOHnc2	Galnc1	Galnc2
13	GEP4	YHR100C	YPDcl2	YPDcl3	EtOHcl1	EtOHcl2	Galcl1	YPDnc1	YPDnc2	YPDnc3	YPDnc4	EtOHnc1	EtOHnc2	Galnc1	Galnc2
13		YOR055W	YPDcl2	YPDcl3	EtOHcl1	EtOHcl2	Galcl1	YPDnc1	YPDnc2	YPDnc3	YPDnc4	EtOHnc1	EtOHnc2	Galnc1	Galnc2
13	MCM16	YPR046W	YPDcl2	YPDcl3	EtOHcl1	EtOHcl2	Galcl1	YPDnc1	YPDnc2	YPDnc3	YPDnc4	EtOHnc1	EtOHnc2	Galnc1	Galnc2
13		YOR102W	YPDcl2	YPDcl3	EtOHcl1	EtOHcl2	Galcl1	YPDnc1	YPDnc2	YPDnc3	YPDnc4	EtOHnc1	EtOHnc2	Galnc1	Galnc2
13	VPS15	YBR097W	YPDcl2	YPDcl3	EtOHcl1	EtOHcl2	Galcl1	YPDnc1	YPDnc2	YPDnc3	YPDnc4	EtOHnc1	EtOHnc2	Galnc1	Galnc2
13	CPR8	YNR028W	YPDcl2	YPDcl3	EtOHcl1	EtOHcl2	Galcl1	YPDnc1	YPDnc2	YPDnc3	YPDnc4	EtOHnc1	EtOHnc2	Galnc1	Galnc2

13	YHC1	YLR298C	YPDcl2	YPDcl3	EtOHcl1	EtOHcl2	Galcl1	YPDnc1	YPDnc2	YPDnc3	YPDnc4	EtOHnc1	EtOHnc2	Galnc1	Galnc2
13	AQY2	YLL052C	YPDcl2	YPDcl3	EtOHcl1	EtOHcl2	Galcl1	YPDnc1	YPDnc2	YPDnc3	YPDnc4	EtOHnc1	EtOHnc2	Galnc1	Galnc2
13		YOL098C	YPDcl2	YPDcl3	EtOHcl1	EtOHcl2	Galcl1	YPDnc1	YPDnc2	YPDnc3	YPDnc4	EtOHnc1	EtOHnc2	Galnc1	Galnc2
13	FSF1	YOR271C	YPDcl2	YPDcl3	EtOHcl1	EtOHcl2	Galcl1	YPDnc1	YPDnc2	YPDnc3	YPDnc4	EtOHnc1	EtOHnc2	Galnc1	Galnc2
13	SMP3	YOR149C	YPDcl2	YPDcl3	EtOHcl1	EtOHcl2	Galcl1	YPDnc1	YPDnc2	YPDnc3	YPDnc4	EtOHnc1	EtOHnc2	Galnc1	Galnc2
13	MMM1	YLL006W	YPDcl2	YPDcl3	EtOHcl1	EtOHcl2	Galcl1	YPDnc1	YPDnc2	YPDnc3	YPDnc4	EtOHnc1	EtOHnc2	Galnc1	Galnc2
13	SEC5	YDR166C	YPDcl2	YPDcl3	EtOHcl1	EtOHcl2	Galcl1	YPDnc1	YPDnc2	YPDnc3	YPDnc4	EtOHnc1	EtOHnc2	Galnc1	Galnc2
13		YIL028W	YPDcl2	YPDcl3	EtOHcl1	EtOHcl2	Galcl1	YPDnc1	YPDnc2	YPDnc3	YPDnc4	EtOHnc1	EtOHnc2	Galnc1	Galnc2
13	YFT2	YDR319C	YPDcl2	YPDcl3	EtOHcl1	EtOHcl2	Galcl1	YPDnc1	YPDnc2	YPDnc3	YPDnc4	EtOHnc1	EtOHnc2	Galnc1	Galnc2
13		YKL133C	YPDcl2	YPDcl3	EtOHcl1	EtOHcl2	Galcl1	YPDnc1	YPDnc2	YPDnc3	YPDnc4	EtOHnc1	EtOHnc2	Galnc1	Galnc2
13	NAM9	YNL137C	YPDcl2	YPDcl3	EtOHcl1	EtOHcl2	Galcl1	YPDnc1	YPDnc2	YPDnc3	YPDnc4	EtOHnc1	EtOHnc2	Galnc1	Galnc2
13	SNF3	YDL194W	YPDcl2	YPDcl3	EtOHcl1	EtOHcl2	Galcl1	YPDnc1	YPDnc2	YPDnc3	YPDnc4	EtOHnc1	EtOHnc2	Galnc1	Galnc2
13	YPT52	YKR014C	YPDcl2	YPDcl3	EtOHcl1	EtOHcl2	Galcl1	YPDnc1	YPDnc2	YPDnc3	YPDnc4	EtOHnc1	EtOHnc2	Galnc1	Galnc2
13		YDL176W	YPDcl2	YPDcl3	EtOHcl1	EtOHcl2	Galcl1	YPDnc1	YPDnc2	YPDnc3	YPDnc4	EtOHnc1	EtOHnc2	Galnc1	Galnc2
13	RIB3	YDR487C	YPDcl2	YPDcl3	EtOHcl1	EtOHcl2	Galcl1	YPDnc1	YPDnc2	YPDnc3	YPDnc4	EtOHnc1	EtOHnc2	Galnc1	Galnc2
13	LPX1	YOR084W	YPDcl2	YPDcl3	EtOHcl1	EtOHcl2	Galcl1	YPDnc1	YPDnc2	YPDnc3	YPDnc4	EtOHnc1	EtOHnc2	Galnc1	Galnc2
13	VHS2	YIL135C	YPDcl2	YPDcl3	EtOHcl1	EtOHcl2	Galcl1	YPDnc1	YPDnc2	YPDnc3	YPDnc4	EtOHnc1	EtOHnc2	Galnc1	Galnc2
13	PNP1	YLR209C	YPDcl2	YPDcl3	EtOHcl1	EtOHcl2	Galcl1	YPDnc1	YPDnc2	YPDnc3	YPDnc4	EtOHnc1	EtOHnc2	Galnc1	Galnc2
13	YOP1	YPR028W	YPDcl2	YPDcl3	EtOHcl1	EtOHcl2	Galcl1	YPDnc1	YPDnc2	YPDnc3	YPDnc4	EtOHnc1	EtOHnc2	Galnc1	Galnc2
13		YIL115W-A	YPDcl2	YPDcl3	EtOHcl1	EtOHcl2	Galcl1	YPDnc1	YPDnc2	YPDnc3	YPDnc4	EtOHnc1	EtOHnc2	Galnc1	Galnc2
13	FKS1	YLR342W	YPDcl2	YPDcl3	EtOHcl1	EtOHcl2	Galcl1	YPDnc1	YPDnc2	YPDnc3	YPDnc4	EtOHnc1	EtOHnc2	Galnc1	Galnc2
13	NDL1	YLR254C	YPDcl2	YPDcl3	EtOHcl1	EtOHcl2	Galcl1	YPDnc1	YPDnc2	YPDnc3	YPDnc4	EtOHnc1	EtOHnc2	Galnc1	Galnc2
13	CSE4	YKL049C	YPDcl2	YPDcl3	EtOHcl1	EtOHcl2	Galcl1	YPDnc1	YPDnc2	YPDnc3	YPDnc4	EtOHnc1	EtOHnc2	Galnc1	Galnc2
13	CDC23	YHR166C	YPDcl2	YPDcl3	EtOHcl1	EtOHcl2	Galcl1	YPDnc1	YPDnc2	YPDnc3	YPDnc4	EtOHnc1	EtOHnc2	Galnc1	Galnc2
13	YSH1	YLR277C	YPDcl2	YPDcl3	EtOHcl1	EtOHcl2	Galcl1	YPDnc1	YPDnc2	YPDnc3	YPDnc4	EtOHnc1	EtOHnc2	Galnc1	Galnc2
13	RLM1	YPL089C	YPDcl2	YPDcl3	EtOHcl1	EtOHcl2	Galcl1	YPDnc1	YPDnc2	YPDnc3	YPDnc4	EtOHnc1	EtOHnc2	Galnc1	Galnc2
13	LST8	YNL006W	YPDcl2	YPDcl3	EtOHcl1	EtOHcl2	Galcl1	YPDnc1	YPDnc2	YPDnc3	YPDnc4	EtOHnc1	EtOHnc2	Galnc1	Galnc2
13	SKO1	YNL167C	YPDcl2	YPDcl3	EtOHcl1	EtOHcl2	Galcl1	YPDnc1	YPDnc2	YPDnc3	YPDnc4	EtOHnc1	EtOHnc2	Galnc1	Galnc2
13		YGR203W	YPDcl2	YPDcl3	EtOHcl1	EtOHcl2	Galcl1	YPDnc1	YPDnc2	YPDnc3	YPDnc4	EtOHnc1	EtOHnc2	Galnc1	Galnc2
13		YJR005C-A	YPDcl2	YPDcl3	EtOHcl1	EtOHcl2	Galcl1	YPDnc1	YPDnc2	YPDnc3	YPDnc4	EtOHnc1	EtOHnc2	Galnc1	Galnc2
13	PDE1	YGL248W	YPDcl2	YPDcl3	EtOHcl1	EtOHcl2	Galcl1	YPDnc1	YPDnc2	YPDnc3	YPDnc4	EtOHnc1	EtOHnc2	Galnc1	Galnc2
13	SPO75	YLL005C	YPDcl2	YPDcl3	EtOHcl1	EtOHcl2	Galcl1	YPDnc1	YPDnc2	YPDnc3	YPDnc4	EtOHnc1	EtOHnc2	Galnc1	Galnc2
13	SHE10	YGL228W	YPDcl2	YPDcl3	EtOHcl1	EtOHcl2	Galcl1	YPDnc1	YPDnc2	YPDnc3	YPDnc4	EtOHnc1	EtOHnc2	Galnc1	Galnc2
13	ISU2	YOR226C	YPDcl2	YPDcl3	EtOHcl1	EtOHcl2	Galcl1	YPDnc1	YPDnc2	YPDnc3	YPDnc4	EtOHnc1	EtOHnc2	Galnc1	Galnc2

13	ERG5	YMR015C	YPDcl2	YPDcl3	EtOHcl1	EtOHcl2	Galcl1	YPDnc1	YPDnc2	YPDnc3	YPDnc4	EtOHnc1	EtOHnc2	Galnc1	Galnc2
13		YJL169W	YPDcl2	YPDcl3	EtOHcl1	EtOHcl2	Galcl1	YPDnc1	YPDnc2	YPDnc3	YPDnc4	EtOHnc1	EtOHnc2	Galnc1	Galnc2
13	CAK1	YFL029C	YPDcl2	YPDcl3	EtOHcl1	EtOHcl2	Galcl1	YPDnc1	YPDnc2	YPDnc3	YPDnc4	EtOHnc1	EtOHnc2	Galnc1	Galnc2
13	PEX27	YOR193W	YPDcl2	YPDcl3	EtOHcl1	EtOHcl2	Galcl1	YPDnc1	YPDnc2	YPDnc3	YPDnc4	EtOHnc1	EtOHnc2	Galnc1	Galnc2
13		YGL194C-A	YPDcl2	YPDcl3	EtOHcl1	EtOHcl2	Galcl1	YPDnc1	YPDnc2	YPDnc3	YPDnc4	EtOHnc1	EtOHnc2	Galnc1	Galnc2
13		YKR075W-/	YPDcl2	YPDcl3	EtOHcl1	EtOHcl2	Galcl1	YPDnc1	YPDnc2	YPDnc3	YPDnc4	EtOHnc1	EtOHnc2	Galnc1	Galnc2
13	IRC8	YJL051W	YPDcl2	YPDcl3	EtOHcl1	EtOHcl2	Galcl1	YPDnc1	YPDnc2	YPDnc3	YPDnc4	EtOHnc1	EtOHnc2	Galnc1	Galnc2
13	ASF2	YDL197C	YPDcl2	YPDcl3	EtOHcl1	EtOHcl2	Galcl1	YPDnc1	YPDnc2	YPDnc3	YPDnc4	EtOHnc1	EtOHnc2	Galnc1	Galnc2
13		YNL089C	YPDcl2	YPDcl3	EtOHcl1	EtOHcl2	Galcl1	YPDnc1	YPDnc2	YPDnc3	YPDnc4	EtOHnc1	EtOHnc2	Galnc1	Galnc2
13	ILV1	YER086W	YPDcl2	YPDcl3	EtOHcl1	EtOHcl2	Galcl1	YPDnc1	YPDnc2	YPDnc3	YPDnc4	EtOHnc1	EtOHnc2	Galnc1	Galnc2
13	QCR7	YDR529C	YPDcl2	YPDcl3	EtOHcl1	EtOHcl2	Galcl1	YPDnc1	YPDnc2	YPDnc3	YPDnc4	EtOHnc1	EtOHnc2	Galnc1	Galnc2
13	MSD1	YPL104W	YPDcl2	YPDcl3	EtOHcl1	EtOHcl2	Galcl1	YPDnc1	YPDnc2	YPDnc3	YPDnc4	EtOHnc1	EtOHnc2	Galnc1	Galnc2
13		YDR018C	YPDcl2	YPDcl3	EtOHcl1	EtOHcl2	Galcl1	YPDnc1	YPDnc2	YPDnc3	YPDnc4	EtOHnc1	EtOHnc2	Galnc1	Galnc2
13	TAF7	YMR227C	YPDcl2	YPDcl3	EtOHcl1	EtOHcl2	Galcl1	YPDnc1	YPDnc2	YPDnc3	YPDnc4	EtOHnc1	EtOHnc2	Galnc1	Galnc2
13	GPX1	YKL026C	YPDcl2	YPDcl3	EtOHcl1	EtOHcl2	Galcl1	YPDnc1	YPDnc2	YPDnc3	YPDnc4	EtOHnc1	EtOHnc2	Galnc1	Galnc2
13	COG8	YML071C	YPDcl2	YPDcl3	EtOHcl1	EtOHcl2	Galcl1	YPDnc1	YPDnc2	YPDnc3	YPDnc4	EtOHnc1	EtOHnc2	Galnc1	Galnc2
13		YOL097W-/	YPDcl2	YPDcl3	EtOHcl1	EtOHcl2	Galcl1	YPDnc1	YPDnc2	YPDnc3	YPDnc4	EtOHnc1	EtOHnc2	Galnc1	Galnc2
13	TRS130	YMR218C	YPDcl2	YPDcl3	EtOHcl1	EtOHcl2	Galcl1	YPDnc1	YPDnc2	YPDnc3	YPDnc4	EtOHnc1	EtOHnc2	Galnc1	Galnc2
13	GRE1	YPL223C	YPDcl2	YPDcl3	EtOHcl1	EtOHcl2	Galcl1	YPDnc1	YPDnc2	YPDnc3	YPDnc4	EtOHnc1	EtOHnc2	Galnc1	Galnc2
13		YKL223W	YPDcl2	YPDcl3	EtOHcl1	EtOHcl2	Galcl1	YPDnc1	YPDnc2	YPDnc3	YPDnc4	EtOHnc1	EtOHnc2	Galnc1	Galnc2
13	TAN1	YGL232W	YPDcl2	YPDcl3	EtOHcl1	EtOHcl2	Galcl1	YPDnc1	YPDnc2	YPDnc3	YPDnc4	EtOHnc1	EtOHnc2	Galnc1	Galnc2
13	SKI8	YGL213C	YPDcl2	YPDcl3	EtOHcl1	EtOHcl2	Galcl1	YPDnc1	YPDnc2	YPDnc3	YPDnc4	EtOHnc1	EtOHnc2	Galnc1	Galnc2
13		YGL101W	YPDcl2	YPDcl3	EtOHcl1	EtOHcl2	Galcl1	YPDnc1	YPDnc2	YPDnc3	YPDnc4	EtOHnc1	EtOHnc2	Galnc1	Galnc2
13	HCS1	YKL017C	YPDcl2	YPDcl3	EtOHcl1	EtOHcl2	Galcl1	YPDnc1	YPDnc2	YPDnc3	YPDnc4	EtOHnc1	EtOHnc2	Galnc1	Galnc2
13	SAL1	YNL083W	YPDcl2	YPDcl3	EtOHcl1	EtOHcl2	Galcl1	YPDnc1	YPDnc2	YPDnc3	YPDnc4	EtOHnc1	EtOHnc2	Galnc1	Galnc2
13	ICS2	YBR157C	YPDcl2	YPDcl3	EtOHcl1	EtOHcl2	Galcl1	YPDnc1	YPDnc2	YPDnc3	YPDnc4	EtOHnc1	EtOHnc2	Galnc1	Galnc2
13	RNA14	YMR061W	YPDcl2	YPDcl3	EtOHcl1	EtOHcl2	Galcl1	YPDnc1	YPDnc2	YPDnc3	YPDnc4	EtOHnc1	EtOHnc2	Galnc1	Galnc2
13		YDR157W	YPDcl2	YPDcl3	EtOHcl1	EtOHcl2	Galcl1	YPDnc1	YPDnc2	YPDnc3	YPDnc4	EtOHnc1	EtOHnc2	Galnc1	Galnc2
13	RRP7	YCL031C	YPDcl2	YPDcl3	EtOHcl1	EtOHcl2	Galcl1	YPDnc1	YPDnc2	YPDnc3	YPDnc4	EtOHnc1	EtOHnc2	Galnc1	Galnc2
13	YVH1	YIR026C	YPDcl2	YPDcl3	EtOHcl1	EtOHcl2	Galcl1	YPDnc1	YPDnc2	YPDnc3	YPDnc4	EtOHnc1	EtOHnc2	Galnc1	Galnc2
13	NUT1	YGL151W	YPDcl2	YPDcl3	EtOHcl1	EtOHcl2	Galcl1	YPDnc1	YPDnc2	YPDnc3	YPDnc4	EtOHnc1	EtOHnc2	Galnc1	Galnc2
13	VPS45	YGL095C	YPDcl2	YPDcl3	EtOHcl1	EtOHcl2	Galcl1	YPDnc1	YPDnc2	YPDnc3	YPDnc4	EtOHnc1	EtOHnc2	Galnc1	Galnc2
13	PAC11	YDR488C	YPDcl2	YPDcl3	EtOHcl1	EtOHcl2	Galcl1	YPDnc1	YPDnc2	YPDnc3	YPDnc4	EtOHnc1	EtOHnc2	Galnc1	Galnc2
13	JIP5	YPR169W	YPDcl2	YPDcl3	EtOHcl1	EtOHcl2	Galcl1	YPDnc1	YPDnc2	YPDnc3	YPDnc4	EtOHnc1	EtOHnc2	Galnc1	Galnc2

13	APL5	YPL195W	YPDcl2	YPDcl3	EtOHcl1	EtOHcl2	Galcl1	YPDnc1	YPDnc2	YPDnc3	YPDnc4	EtOHnc1	EtOHnc2	Galnc1	Galnc2
13	SCS22	YBL091C-A	YPDcl2	YPDcl3	EtOHcl1	EtOHcl2	Galcl1	YPDnc1	YPDnc2	YPDnc3	YPDnc4	EtOHnc1	EtOHnc2	Galnc1	Galnc2
13		YPR010C-A	YPDcl2	YPDcl3	EtOHcl1	EtOHcl2	Galcl1	YPDnc1	YPDnc2	YPDnc3	YPDnc4	EtOHnc1	EtOHnc2	Galnc1	Galnc2
13	PAH1	YMR165C	YPDcl2	YPDcl3	EtOHcl1	EtOHcl2	Galcl1	YPDnc1	YPDnc2	YPDnc3	YPDnc4	EtOHnc1	EtOHnc2	Galnc1	Galnc2
13		YGR015C	YPDcl2	YPDcl3	EtOHcl1	EtOHcl2	Galcl1	YPDnc1	YPDnc2	YPDnc3	YPDnc4	EtOHnc1	EtOHnc2	Galnc1	Galnc2
13	MCM7	YBR202W	YPDcl2	YPDcl3	EtOHcl1	EtOHcl2	Galcl1	YPDnc1	YPDnc2	YPDnc3	YPDnc4	EtOHnc1	EtOHnc2	Galnc1	Galnc2
13	ASK1	YKL052C	YPDcl2	YPDcl3	EtOHcl1	EtOHcl2	Galcl1	YPDnc1	YPDnc2	YPDnc3	YPDnc4	EtOHnc1	EtOHnc2	Galnc1	Galnc2
13	APM1	YPL259C	YPDcl2	YPDcl3	EtOHcl1	EtOHcl2	Galcl1	YPDnc1	YPDnc2	YPDnc3	YPDnc4	EtOHnc1	EtOHnc2	Galnc1	Galnc2
13		YIR020C	YPDcl2	YPDcl3	EtOHcl1	EtOHcl2	Galcl1	YPDnc1	YPDnc2	YPDnc3	YPDnc4	EtOHnc1	EtOHnc2	Galnc1	Galnc2
13	EFR3	YMR212C	YPDcl2	YPDcl3	EtOHcl1	EtOHcl2	Galcl1	YPDnc1	YPDnc2	YPDnc3	YPDnc4	EtOHnc1	EtOHnc2	Galnc1	Galnc2
13	ALR2	YFL050C	YPDcl2	YPDcl3	EtOHcl1	EtOHcl2	Galcl1	YPDnc1	YPDnc2	YPDnc3	YPDnc4	EtOHnc1	EtOHnc2	Galnc1	Galnc2
13		YER087C-A	YPDcl2	YPDcl3	EtOHcl1	EtOHcl2	Galcl1	YPDnc1	YPDnc2	YPDnc3	YPDnc4	EtOHnc1	EtOHnc2	Galnc1	Galnc2
13	TOF2	YKR010C	YPDcl2	YPDcl3	EtOHcl1	EtOHcl2	Galcl1	YPDnc1	YPDnc2	YPDnc3	YPDnc4	EtOHnc1	EtOHnc2	Galnc1	Galnc2
13	RPL6B	YLR448W	YPDcl2	YPDcl3	EtOHcl1	EtOHcl2	Galcl1	YPDnc1	YPDnc2	YPDnc3	YPDnc4	EtOHnc1	EtOHnc2	Galnc1	Galnc2
13	RET2	YFR051C	YPDcl2	YPDcl3	EtOHcl1	EtOHcl2	Galcl1	YPDnc1	YPDnc2	YPDnc3	YPDnc4	EtOHnc1	EtOHnc2	Galnc1	Galnc2
13	AYT1	YLL063C	YPDcl2	YPDcl3	EtOHcl1	EtOHcl2	Galcl1	YPDnc1	YPDnc2	YPDnc3	YPDnc4	EtOHnc1	EtOHnc2	Galnc1	Galnc2
13	SSL1	YLR005W	YPDcl2	YPDcl3	EtOHcl1	EtOHcl2	Galcl1	YPDnc1	YPDnc2	YPDnc3	YPDnc4	EtOHnc1	EtOHnc2	Galnc1	Galnc2
13	ATG3	YNR007C	YPDcl2	YPDcl3	EtOHcl1	EtOHcl2	Galcl1	YPDnc1	YPDnc2	YPDnc3	YPDnc4	EtOHnc1	EtOHnc2	Galnc1	Galnc2
13	IZH4	YOL101C	YPDcl2	YPDcl3	EtOHcl1	EtOHcl2	Galcl1	YPDnc1	YPDnc2	YPDnc3	YPDnc4	EtOHnc1	EtOHnc2	Galnc1	Galnc2
13	YAT2	YER024W	YPDcl2	YPDcl3	EtOHcl1	EtOHcl2	Galcl1	YPDnc1	YPDnc2	YPDnc3	YPDnc4	EtOHnc1	EtOHnc2	Galnc1	Galnc2
13	GPD1	YDL022W	YPDcl2	YPDcl3	EtOHcl1	EtOHcl2	Galcl1	YPDnc1	YPDnc2	YPDnc3	YPDnc4	EtOHnc1	EtOHnc2	Galnc1	Galnc2
13	MSE1	YOL033W	YPDcl2	YPDcl3	EtOHcl1	EtOHcl2	Galcl1	YPDnc1	YPDnc2	YPDnc3	YPDnc4	EtOHnc1	EtOHnc2	Galnc1	Galnc2
13	ENT3	YJR125C	YPDcl2	YPDcl3	EtOHcl1	EtOHcl2	Galcl1	YPDnc1	YPDnc2	YPDnc3	YPDnc4	EtOHnc1	EtOHnc2	Galnc1	Galnc2
13	GSY2	YLR258W	YPDcl2	YPDcl3	EtOHcl1	EtOHcl2	Galcl1	YPDnc1	YPDnc2	YPDnc3	YPDnc4	EtOHnc1	EtOHnc2	Galnc1	Galnc2
13	BOI1	YBL085W	YPDcl2	YPDcl3	EtOHcl1	EtOHcl2	Galcl1	YPDnc1	YPDnc2	YPDnc3	YPDnc4	EtOHnc1	EtOHnc2	Galnc1	Galnc2
13	HSH155	YMR288W	YPDcl2	YPDcl3	EtOHcl1	EtOHcl2	Galcl1	YPDnc1	YPDnc2	YPDnc3	YPDnc4	EtOHnc1	EtOHnc2	Galnc1	Galnc2
13	FMO1	YHR176W	YPDcl2	YPDcl3	EtOHcl1	EtOHcl2	Galcl1	YPDnc1	YPDnc2	YPDnc3	YPDnc4	EtOHnc1	EtOHnc2	Galnc1	Galnc2
13	SNC2	YOR327C	YPDcl2	YPDcl3	EtOHcl1	EtOHcl2	Galcl1	YPDnc1	YPDnc2	YPDnc3	YPDnc4	EtOHnc1	EtOHnc2	Galnc1	Galnc2
13	BUD17	YNR027W	YPDcl2	YPDcl3	EtOHcl1	EtOHcl2	Galcl1	YPDnc1	YPDnc2	YPDnc3	YPDnc4	EtOHnc1	EtOHnc2	Galnc1	Galnc2
13	TAH1	YCR060W	YPDcl2	YPDcl3	EtOHcl1	EtOHcl2	Galcl1	YPDnc1	YPDnc2	YPDnc3	YPDnc4	EtOHnc1	EtOHnc2	Galnc1	Galnc2
13	ZRT2	YLR130C	YPDcl2	YPDcl3	EtOHcl1	EtOHcl2	Galcl1	YPDnc1	YPDnc2	YPDnc3	YPDnc4	EtOHnc1	EtOHnc2	Galnc1	Galnc2
13	LYS4	YDR234W	YPDcl2	YPDcl3	EtOHcl1	EtOHcl2	Galcl1	YPDnc1	YPDnc2	YPDnc3	YPDnc4	EtOHnc1	EtOHnc2	Galnc1	Galnc2
13	GET4	YOR164C	YPDcl2	YPDcl3	EtOHcl1	EtOHcl2	Galcl1	YPDnc1	YPDnc2	YPDnc3	YPDnc4	EtOHnc1	EtOHnc2	Galnc1	Galnc2
13	GDH3	YAL062W	YPDcl2	YPDcl3	EtOHcl1	EtOHcl2	Galcl1	YPDnc1	YPDnc2	YPDnc3	YPDnc4	EtOHnc1	EtOHnc2	Galnc1	Galnc2

13		YOR376W-	YPDcl2	YPDcl3	EtOHcl1	EtOHcl2	Galcl1	YPDnc1	YPDnc2	YPDnc3	YPDnc4	EtOHnc1	EtOHnc2	Galnc1	Galnc2
13	RRN6	YBL014C	YPDcl2	YPDcl3	EtOHcl1	EtOHcl2	Galcl1	YPDnc1	YPDnc2	YPDnc3	YPDnc4	EtOHnc1	EtOHnc2	Galnc1	Galnc2
13	MAF1	YDR005C	YPDcl2	YPDcl3	EtOHcl1	EtOHcl2	Galcl1	YPDnc1	YPDnc2	YPDnc3	YPDnc4	EtOHnc1	EtOHnc2	Galnc1	Galnc2
13	AIM28	YKR016W	YPDcl2	YPDcl3	EtOHcl1	EtOHcl2	Galcl1	YPDnc1	YPDnc2	YPDnc3	YPDnc4	EtOHnc1	EtOHnc2	Galnc1	Galnc2
13		YDL163W	YPDcl2	YPDcl3	EtOHcl1	EtOHcl2	Galcl1	YPDnc1	YPDnc2	YPDnc3	YPDnc4	EtOHnc1	EtOHnc2	Galnc1	Galnc2
13	CWC25	YNL245C	YPDcl2	YPDcl3	EtOHcl1	EtOHcl2	Galcl1	YPDnc1	YPDnc2	YPDnc3	YPDnc4	EtOHnc1	EtOHnc2	Galnc1	Galnc2
13	CBP3	YPL215W	YPDcl2	YPDcl3	EtOHcl1	EtOHcl2	Galcl1	YPDnc1	YPDnc2	YPDnc3	YPDnc4	EtOHnc1	EtOHnc2	Galnc1	Galnc2
13	QNS1	YHR074W	YPDcl2	YPDcl3	EtOHcl1	EtOHcl2	Galcl1	YPDnc1	YPDnc2	YPDnc3	YPDnc4	EtOHnc1	EtOHnc2	Galnc1	Galnc2
13	DAL4	YIR028W	YPDcl2	YPDcl3	EtOHcl1	EtOHcl2	Galcl1	YPDnc1	YPDnc2	YPDnc3	YPDnc4	EtOHnc1	EtOHnc2	Galnc1	Galnc2
13		YPR170C	YPDcl2	YPDcl3	EtOHcl1	EtOHcl2	Galcl1	YPDnc1	YPDnc2	YPDnc3	YPDnc4	EtOHnc1	EtOHnc2	Galnc1	Galnc2
13	KRR1	YCL059C	YPDcl2	YPDcl3	EtOHcl1	EtOHcl2	Galcl1	YPDnc1	YPDnc2	YPDnc3	YPDnc4	EtOHnc1	EtOHnc2	Galnc1	Galnc2
13		YAL037W	YPDcl2	YPDcl3	EtOHcl1	EtOHcl2	Galcl1	YPDnc1	YPDnc2	YPDnc3	YPDnc4	EtOHnc1	EtOHnc2	Galnc1	Galnc2
13	PMI40	YER003C	YPDcl2	YPDcl3	EtOHcl1	EtOHcl2	Galcl1	YPDnc1	YPDnc2	YPDnc3	YPDnc4	EtOHnc1	EtOHnc2	Galnc1	Galnc2
13		YJL119C	YPDcl2	YPDcl3	EtOHcl1	EtOHcl2	Galcl1	YPDnc1	YPDnc2	YPDnc3	YPDnc4	EtOHnc1	EtOHnc2	Galnc1	Galnc2
13		YOL050C	YPDcl2	YPDcl3	EtOHcl1	EtOHcl2	Galcl1	YPDnc1	YPDnc2	YPDnc3	YPDnc4	EtOHnc1	EtOHnc2	Galnc1	Galnc2
13		YMR085W	YPDcl2	YPDcl3	EtOHcl1	EtOHcl2	Galcl1	YPDnc1	YPDnc2	YPDnc3	YPDnc4	EtOHnc1	EtOHnc2	Galnc1	Galnc2
13	LSB5	YCL034W	YPDcl2	YPDcl3	EtOHcl1	EtOHcl2	Galcl1	YPDnc1	YPDnc2	YPDnc3	YPDnc4	EtOHnc1	EtOHnc2	Galnc1	Galnc2
13	GPI16	YHR188C	YPDcl2	YPDcl3	EtOHcl1	EtOHcl2	Galcl1	YPDnc1	YPDnc2	YPDnc3	YPDnc4	EtOHnc1	EtOHnc2	Galnc1	Galnc2
13	REX4	YOL080C	YPDcl2	YPDcl3	EtOHcl1	EtOHcl2	Galcl1	YPDnc1	YPDnc2	YPDnc3	YPDnc4	EtOHnc1	EtOHnc2	Galnc1	Galnc2
13	LCP5	YER127W	YPDcl2	YPDcl3	EtOHcl1	EtOHcl2	Galcl1	YPDnc1	YPDnc2	YPDnc3	YPDnc4	EtOHnc1	EtOHnc2	Galnc1	Galnc2
13	GRR1	YJR090C	YPDcl2	YPDcl3	EtOHcl1	EtOHcl2	Galcl1	YPDnc1	YPDnc2	YPDnc3	YPDnc4	EtOHnc1	EtOHnc2	Galnc1	Galnc2
13	PHS1	YJL097W	YPDcl2	YPDcl3	EtOHcl1	EtOHcl2	Galcl1	YPDnc1	YPDnc2	YPDnc3	YPDnc4	EtOHnc1	EtOHnc2	Galnc1	Galnc2
13	GRX5	YPL059W	YPDcl2	YPDcl3	EtOHcl1	EtOHcl2	Galcl1	YPDnc1	YPDnc2	YPDnc3	YPDnc4	EtOHnc1	EtOHnc2	Galnc1	Galnc2
13	TYW3	YGL050W	YPDcl2	YPDcl3	EtOHcl1	EtOHcl2	Galcl1	YPDnc1	YPDnc2	YPDnc3	YPDnc4	EtOHnc1	EtOHnc2	Galnc1	Galnc2
13	PMT5	YDL093W	YPDcl2	YPDcl3	EtOHcl1	EtOHcl2	Galcl1	YPDnc1	YPDnc2	YPDnc3	YPDnc4	EtOHnc1	EtOHnc2	Galnc1	Galnc2
13	PEX22	YAL055W	YPDcl2	YPDcl3	EtOHcl1	EtOHcl2	Galcl1	YPDnc1	YPDnc2	YPDnc3	YPDnc4	EtOHnc1	EtOHnc2	Galnc1	Galnc2
13	DLD1	YDL174C	YPDcl2	YPDcl3	EtOHcl1	EtOHcl2	Galcl1	YPDnc1	YPDnc2	YPDnc3	YPDnc4	EtOHnc1	EtOHnc2	Galnc1	Galnc2
13	UBC7	YMR022W	YPDcl2	YPDcl3	EtOHcl1	EtOHcl2	Galcl1	YPDnc1	YPDnc2	YPDnc3	YPDnc4	EtOHnc1	EtOHnc2	Galnc1	Galnc2
13	TAE1	YBR261C	YPDcl2	YPDcl3	EtOHcl1	EtOHcl2	Galcl1	YPDnc1	YPDnc2	YPDnc3	YPDnc4	EtOHnc1	EtOHnc2	Galnc1	Galnc2
13	YCK1	YHR135C	YPDcl2	YPDcl3	EtOHcl1	EtOHcl2	Galcl1	YPDnc1	YPDnc2	YPDnc3	YPDnc4	EtOHnc1	EtOHnc2	Galnc1	Galnc2
13	CTK3	YML112W	YPDcl2	YPDcl3	EtOHcl1	EtOHcl2	Galcl1	YPDnc1	YPDnc2	YPDnc3	YPDnc4	EtOHnc1	EtOHnc2	Galnc1	Galnc2
13	BUG1	YDL099W	YPDcl2	YPDcl3	EtOHcl1	EtOHcl2	Galcl1	YPDnc1	YPDnc2	YPDnc3	YPDnc4	EtOHnc1	EtOHnc2	Galnc1	Galnc2
13	ZUO1	YGR285C	YPDcl2	YPDcl3	EtOHcl1	EtOHcl2	Galcl1	YPDnc1	YPDnc2	YPDnc3	YPDnc4	EtOHnc1	EtOHnc2	Galnc1	Galnc2
13	ENV7	YPL236C	YPDcl2	YPDcl3	EtOHcl1	EtOHcl2	Galcl1	YPDnc1	YPDnc2	YPDnc3	YPDnc4	EtOHnc1	EtOHnc2	Galnc1	Galnc2

13	REG1	YDR028C	YPDcl2	YPDcl3	EtOHcl1	EtOHcl2	Galcl1	YPDnc1	YPDnc2	YPDnc3	YPDnc4	EtOHnc1	EtOHnc2	Galnc1	Galnc2
13	BST1	YFL025C	YPDcl2	YPDcl3	EtOHcl1	EtOHcl2	Galcl1	YPDnc1	YPDnc2	YPDnc3	YPDnc4	EtOHnc1	EtOHnc2	Galnc1	Galnc2
13	CWC15	YDR163W	YPDcl2	YPDcl3	EtOHcl1	EtOHcl2	Galcl1	YPDnc1	YPDnc2	YPDnc3	YPDnc4	EtOHnc1	EtOHnc2	Galnc1	Galnc2
13	XPT1	YJR133W	YPDcl2	YPDcl3	EtOHcl1	EtOHcl2	Galcl1	YPDnc1	YPDnc2	YPDnc3	YPDnc4	EtOHnc1	EtOHnc2	Galnc1	Galnc2
13		YKL171W	YPDcl2	YPDcl3	EtOHcl1	EtOHcl2	Galcl1	YPDnc1	YPDnc2	YPDnc3	YPDnc4	EtOHnc1	EtOHnc2	Galnc1	Galnc2
13	OPI3	YJR073C	YPDcl2	YPDcl3	EtOHcl1	EtOHcl2	Galcl1	YPDnc1	YPDnc2	YPDnc3	YPDnc4	EtOHnc1	EtOHnc2	Galnc1	Galnc2
13	ELP4	YPL101W	YPDcl2	YPDcl3	EtOHcl1	EtOHcl2	Galcl1	YPDnc1	YPDnc2	YPDnc3	YPDnc4	EtOHnc1	EtOHnc2	Galnc1	Galnc2
13	CAF130	YGR134W	YPDcl2	YPDcl3	EtOHcl1	EtOHcl2	Galcl1	YPDnc1	YPDnc2	YPDnc3	YPDnc4	EtOHnc1	EtOHnc2	Galnc1	Galnc2
13		YKL075C	YPDcl2	YPDcl3	EtOHcl1	EtOHcl2	Galcl1	YPDnc1	YPDnc2	YPDnc3	YPDnc4	EtOHnc1	EtOHnc2	Galnc1	Galnc2
13	PKC1	YBL105C	YPDcl2	YPDcl3	EtOHcl1	EtOHcl2	Galcl1	YPDnc1	YPDnc2	YPDnc3	YPDnc4	EtOHnc1	EtOHnc2	Galnc1	Galnc2
13		YLR198C	YPDcl2	YPDcl3	EtOHcl1	EtOHcl2	Galcl1	YPDnc1	YPDnc2	YPDnc3	YPDnc4	EtOHnc1	EtOHnc2	Galnc1	Galnc2
13		YJR107W	YPDcl2	YPDcl3	EtOHcl1	EtOHcl2	Galcl1	YPDnc1	YPDnc2	YPDnc3	YPDnc4	EtOHnc1	EtOHnc2	Galnc1	Galnc2
13	GPI17	YDR434W	YPDcl2	YPDcl3	EtOHcl1	EtOHcl2	Galcl1	YPDnc1	YPDnc2	YPDnc3	YPDnc4	EtOHnc1	EtOHnc2	Galnc1	Galnc2
13	PFA3	YNL326C	YPDcl2	YPDcl3	EtOHcl1	EtOHcl2	Galcl1	YPDnc1	YPDnc2	YPDnc3	YPDnc4	EtOHnc1	EtOHnc2	Galnc1	Galnc2
13	BYE1	YKL005C	YPDcl2	YPDcl3	EtOHcl1	EtOHcl2	Galcl1	YPDnc1	YPDnc2	YPDnc3	YPDnc4	EtOHnc1	EtOHnc2	Galnc1	Galnc2
13		YOR021C	YPDcl2	YPDcl3	EtOHcl1	EtOHcl2	Galcl1	YPDnc1	YPDnc2	YPDnc3	YPDnc4	EtOHnc1	EtOHnc2	Galnc1	Galnc2
13	AIR1	YIL079C	YPDcl2	YPDcl3	EtOHcl1	EtOHcl2	Galcl1	YPDnc1	YPDnc2	YPDnc3	YPDnc4	EtOHnc1	EtOHnc2	Galnc1	Galnc2
13		YDL041W	YPDcl2	YPDcl3	EtOHcl1	EtOHcl2	Galcl1	YPDnc1	YPDnc2	YPDnc3	YPDnc4	EtOHnc1	EtOHnc2	Galnc1	Galnc2
13	TLG2	YOL018C	YPDcl2	YPDcl3	EtOHcl1	EtOHcl2	Galcl1	YPDnc1	YPDnc2	YPDnc3	YPDnc4	EtOHnc1	EtOHnc2	Galnc1	Galnc2
13		YNR029C	YPDcl2	YPDcl3	EtOHcl1	EtOHcl2	Galcl1	YPDnc1	YPDnc2	YPDnc3	YPDnc4	EtOHnc1	EtOHnc2	Galnc1	Galnc2
13	YPC1	YBR183W	YPDcl2	YPDcl3	EtOHcl1	EtOHcl2	Galcl1	YPDnc1	YPDnc2	YPDnc3	YPDnc4	EtOHnc1	EtOHnc2	Galnc1	Galnc2
13	NUP60	YAR002W	YPDcl2	YPDcl3	EtOHcl1	EtOHcl2	Galcl1	YPDnc1	YPDnc2	YPDnc3	YPDnc4	EtOHnc1	EtOHnc2	Galnc1	Galnc2
13		YNL296W	YPDcl2	YPDcl3	EtOHcl1	EtOHcl2	Galcl1	YPDnc1	YPDnc2	YPDnc3	YPDnc4	EtOHnc1	EtOHnc2	Galnc1	Galnc2
13	YRB2	YIL063C	YPDcl2	YPDcl3	EtOHcl1	EtOHcl2	Galcl1	YPDnc1	YPDnc2	YPDnc3	YPDnc4	EtOHnc1	EtOHnc2	Galnc1	Galnc2
13	RET3	YPL010W	YPDcl2	YPDcl3	EtOHcl1	EtOHcl2	Galcl1	YPDnc1	YPDnc2	YPDnc3	YPDnc4	EtOHnc1	EtOHnc2	Galnc1	Galnc2
13	PZF1	YPR186C	YPDcl2	YPDcl3	EtOHcl1	EtOHcl2	Galcl1	YPDnc1	YPDnc2	YPDnc3	YPDnc4	EtOHnc1	EtOHnc2	Galnc1	Galnc2
13		YBR131C-A	YPDcl2	YPDcl3	EtOHcl1	EtOHcl2	Galcl1	YPDnc1	YPDnc2	YPDnc3	YPDnc4	EtOHnc1	EtOHnc2	Galnc1	Galnc2
13	THP2	YHR167W	YPDcl2	YPDcl3	EtOHcl1	EtOHcl2	Galcl1	YPDnc1	YPDnc2	YPDnc3	YPDnc4	EtOHnc1	EtOHnc2	Galnc1	Galnc2
13	HPA3	YEL066W	YPDcl2	YPDcl3	EtOHcl1	EtOHcl2	Galcl1	YPDnc1	YPDnc2	YPDnc3	YPDnc4	EtOHnc1	EtOHnc2	Galnc1	Galnc2
13	NOB1	YOR056C	YPDcl2	YPDcl3	EtOHcl1	EtOHcl2	Galcl1	YPDnc1	YPDnc2	YPDnc3	YPDnc4	EtOHnc1	EtOHnc2	Galnc1	Galnc2
13	MAP2	YBL091C	YPDcl2	YPDcl3	EtOHcl1	EtOHcl2	Galcl1	YPDnc1	YPDnc2	YPDnc3	YPDnc4	EtOHnc1	EtOHnc2	Galnc1	Galnc2
13	QCR6	YFR033C	YPDcl2	YPDcl3	EtOHcl1	EtOHcl2	Galcl1	YPDnc1	YPDnc2	YPDnc3	YPDnc4	EtOHnc1	EtOHnc2	Galnc1	Galnc2
13	MSS116	YDR194C	YPDcl2	YPDcl3	EtOHcl1	EtOHcl2	Galcl1	YPDnc1	YPDnc2	YPDnc3	YPDnc4	EtOHnc1	EtOHnc2	Galnc1	Galnc2
13		YHR022C-A	YPDcl2	YPDcl3	EtOHcl1	EtOHcl2	Galcl1	YPDnc1	YPDnc2	YPDnc3	YPDnc4	EtOHnc1	EtOHnc2	Galnc1	Galnc2

13		YHR165W-/	YPDcl2	YPDcl3	EtOHcl1	EtOHcl2	Galcl1	YPDnc1	YPDnc2	YPDnc3	YPDnc4	EtOHnc1	EtOHnc2	Galnc1	Galnc2
13	ATP22	YDR350C	YPDcl2	YPDcl3	EtOHcl1	EtOHcl2	Galcl1	YPDnc1	YPDnc2	YPDnc3	YPDnc4	EtOHnc1	EtOHnc2	Galnc1	Galnc2
13	SPO20	YMR017W	YPDcl2	YPDcl3	EtOHcl1	EtOHcl2	Galcl1	YPDnc1	YPDnc2	YPDnc3	YPDnc4	EtOHnc1	EtOHnc2	Galnc1	Galnc2
13	ARP8	YOR141C	YPDcl2	YPDcl3	EtOHcl1	EtOHcl2	Galcl1	YPDnc1	YPDnc2	YPDnc3	YPDnc4	EtOHnc1	EtOHnc2	Galnc1	Galnc2
13	PRP3	YDR473C	YPDcl2	YPDcl3	EtOHcl1	EtOHcl2	Galcl1	YPDnc1	YPDnc2	YPDnc3	YPDnc4	EtOHnc1	EtOHnc2	Galnc1	Galnc2
13		YDL057W	YPDcl2	YPDcl3	EtOHcl1	EtOHcl2	Galcl1	YPDnc1	YPDnc2	YPDnc3	YPDnc4	EtOHnc1	EtOHnc2	Galnc1	Galnc2
13	DAL7	YIR031C	YPDcl2	YPDcl3	EtOHcl1	EtOHcl2	Galcl1	YPDnc1	YPDnc2	YPDnc3	YPDnc4	EtOHnc1	EtOHnc2	Galnc1	Galnc2
13	STE3	YKL178C	YPDcl2	YPDcl3	EtOHcl1	EtOHcl2	Galcl1	YPDnc1	YPDnc2	YPDnc3	YPDnc4	EtOHnc1	EtOHnc2	Galnc1	Galnc2
13	VAM6	YDL077C	YPDcl2	YPDcl3	EtOHcl1	EtOHcl2	Galcl1	YPDnc1	YPDnc2	YPDnc3	YPDnc4	EtOHnc1	EtOHnc2	Galnc1	Galnc2
13	TOS8	YGL096W	YPDcl2	YPDcl3	EtOHcl1	EtOHcl2	Galcl1	YPDnc1	YPDnc2	YPDnc3	YPDnc4	EtOHnc1	EtOHnc2	Galnc1	Galnc2
13	PRP24	YMR268C	YPDcl2	YPDcl3	EtOHcl1	EtOHcl2	Galcl1	YPDnc1	YPDnc2	YPDnc3	YPDnc4	EtOHnc1	EtOHnc2	Galnc1	Galnc2
13	RET1	YOR207C	YPDcl2	YPDcl3	EtOHcl1	EtOHcl2	Galcl1	YPDnc1	YPDnc2	YPDnc3	YPDnc4	EtOHnc1	EtOHnc2	Galnc1	Galnc2
13	RVS161	YCR009C	YPDcl2	YPDcl3	EtOHcl1	EtOHcl2	Galcl1	YPDnc1	YPDnc2	YPDnc3	YPDnc4	EtOHnc1	EtOHnc2	Galnc1	Galnc2
13		YLR053C	YPDcl2	YPDcl3	EtOHcl1	EtOHcl2	Galcl1	YPDnc1	YPDnc2	YPDnc3	YPDnc4	EtOHnc1	EtOHnc2	Galnc1	Galnc2
13	IGO1	YNL157W	YPDcl2	YPDcl3	EtOHcl1	EtOHcl2	Galcl1	YPDnc1	YPDnc2	YPDnc3	YPDnc4	EtOHnc1	EtOHnc2	Galnc1	Galnc2
13	NFU1	YKL040C	YPDcl2	YPDcl3	EtOHcl1	EtOHcl2	Galcl1	YPDnc1	YPDnc2	YPDnc3	YPDnc4	EtOHnc1	EtOHnc2	Galnc1	Galnc2
13	NUP120	YKL057C	YPDcl2	YPDcl3	EtOHcl1	EtOHcl2	Galcl1	YPDnc1	YPDnc2	YPDnc3	YPDnc4	EtOHnc1	EtOHnc2	Galnc1	Galnc2
13		YLR211C	YPDcl2	YPDcl3	EtOHcl1	EtOHcl2	Galcl1	YPDnc1	YPDnc2	YPDnc3	YPDnc4	EtOHnc1	EtOHnc2	Galnc1	Galnc2
13	GEP5	YLR091W	YPDcl2	YPDcl3	EtOHcl1	EtOHcl2	Galcl1	YPDnc1	YPDnc2	YPDnc3	YPDnc4	EtOHnc1	EtOHnc2	Galnc1	Galnc2
13	WAR1	YML076C	YPDcl2	YPDcl3	EtOHcl1	EtOHcl2	Galcl1	YPDnc1	YPDnc2	YPDnc3	YPDnc4	EtOHnc1	EtOHnc2	Galnc1	Galnc2
13	BRE5	YNR051C	YPDcl2	YPDcl3	EtOHcl1	EtOHcl2	Galcl1	YPDnc1	YPDnc2	YPDnc3	YPDnc4	EtOHnc1	EtOHnc2	Galnc1	Galnc2
13	PAD1	YDR538W	YPDcl2	YPDcl3	EtOHcl1	EtOHcl2	Galcl1	YPDnc1	YPDnc2	YPDnc3	YPDnc4	EtOHnc1	EtOHnc2	Galnc1	Galnc2
13	ERV29	YGR284C	YPDcl2	YPDcl3	EtOHcl1	EtOHcl2	Galcl1	YPDnc1	YPDnc2	YPDnc3	YPDnc4	EtOHnc1	EtOHnc2	Galnc1	Galnc2
13	GLC8	YMR311C	YPDcl2	YPDcl3	EtOHcl1	EtOHcl2	Galcl1	YPDnc1	YPDnc2	YPDnc3	YPDnc4	EtOHnc1	EtOHnc2	Galnc1	Galnc2
13	SLF1	YDR515W	YPDcl2	YPDcl3	EtOHcl1	EtOHcl2	Galcl1	YPDnc1	YPDnc2	YPDnc3	YPDnc4	EtOHnc1	EtOHnc2	Galnc1	Galnc2
13	PRM1	YNL279W	YPDcl2	YPDcl3	EtOHcl1	EtOHcl2	Galcl1	YPDnc1	YPDnc2	YPDnc3	YPDnc4	EtOHnc1	EtOHnc2	Galnc1	Galnc2
13	MRPL10	YNL284C	YPDcl2	YPDcl3	EtOHcl1	EtOHcl2	Galcl1	YPDnc1	YPDnc2	YPDnc3	YPDnc4	EtOHnc1	EtOHnc2	Galnc1	Galnc2
13	ABD1	YBR236C	YPDcl2	YPDcl3	EtOHcl1	EtOHcl2	Galcl1	YPDnc1	YPDnc2	YPDnc3	YPDnc4	EtOHnc1	EtOHnc2	Galnc1	Galnc2
13	GPT2	YKR067W	YPDcl2	YPDcl3	EtOHcl1	EtOHcl2	Galcl1	YPDnc1	YPDnc2	YPDnc3	YPDnc4	EtOHnc1	EtOHnc2	Galnc1	Galnc2
13	LAG2	YOL025W	YPDcl2	YPDcl3	EtOHcl1	EtOHcl2	Galcl1	YPDnc1	YPDnc2	YPDnc3	YPDnc4	EtOHnc1	EtOHnc2	Galnc1	Galnc2
13		YJR011C	YPDcl2	YPDcl3	EtOHcl1	EtOHcl2	Galcl1	YPDnc1	YPDnc2	YPDnc3	YPDnc4	EtOHnc1	EtOHnc2	Galnc1	Galnc2
13	SNM1	YDR478W	YPDcl2	YPDcl3	EtOHcl1	EtOHcl2	Galcl1	YPDnc1	YPDnc2	YPDnc3	YPDnc4	EtOHnc1	EtOHnc2	Galnc1	Galnc2
13	BUD32	YGR262C	YPDcl2	YPDcl3	EtOHcl1	EtOHcl2	Galcl1	YPDnc1	YPDnc2	YPDnc3	YPDnc4	EtOHnc1	EtOHnc2	Galnc1	Galnc2
13	DAL3	YIR032C	YPDcl2	YPDcl3	EtOHcl1	EtOHcl2	Galcl1	YPDnc1	YPDnc2	YPDnc3	YPDnc4	EtOHnc1	EtOHnc2	Galnc1	Galnc2

13	ATP12	YJL180C	YPDcl2	YPDcl3	EtOHcl1	EtOHcl2	Galcl1	YPDnc1	YPDnc2	YPDnc3	YPDnc4	EtOHnc1	EtOHnc2	Galnc1	Galnc2
13	RRP40	YOL142W	YPDcl2	YPDcl3	EtOHcl1	EtOHcl2	Galcl1	YPDnc1	YPDnc2	YPDnc3	YPDnc4	EtOHnc1	EtOHnc2	Galnc1	Galnc2
13	MRK1	YDL079C	YPDcl2	YPDcl3	EtOHcl1	EtOHcl2	Galcl1	YPDnc1	YPDnc2	YPDnc3	YPDnc4	EtOHnc1	EtOHnc2	Galnc1	Galnc2
13	SOM1	YEL059C-A	YPDcl2	YPDcl3	EtOHcl1	EtOHcl2	Galcl1	YPDnc1	YPDnc2	YPDnc3	YPDnc4	EtOHnc1	EtOHnc2	Galnc1	Galnc2
13	DBP7	YKR024C	YPDcl2	YPDcl3	EtOHcl1	EtOHcl2	Galcl1	YPDnc1	YPDnc2	YPDnc3	YPDnc4	EtOHnc1	EtOHnc2	Galnc1	Galnc2
13		YGR259C	YPDcl2	YPDcl3	EtOHcl1	EtOHcl2	Galcl1	YPDnc1	YPDnc2	YPDnc3	YPDnc4	EtOHnc1	EtOHnc2	Galnc1	Galnc2
13	MAK5	YBR142W	YPDcl2	YPDcl3	EtOHcl1	EtOHcl2	Galcl1	YPDnc1	YPDnc2	YPDnc3	YPDnc4	EtOHnc1	EtOHnc2	Galnc1	Galnc2
13	ICS3	YJL077C	YPDcl2	YPDcl3	EtOHcl1	EtOHcl2	Galcl1	YPDnc1	YPDnc2	YPDnc3	YPDnc4	EtOHnc1	EtOHnc2	Galnc1	Galnc2
13	CMS1	YLR003C	YPDcl2	YPDcl3	EtOHcl1	EtOHcl2	Galcl1	YPDnc1	YPDnc2	YPDnc3	YPDnc4	EtOHnc1	EtOHnc2	Galnc1	Galnc2
13	EOS1	YNL080C	YPDcl2	YPDcl3	EtOHcl1	EtOHcl2	Galcl1	YPDnc1	YPDnc2	YPDnc3	YPDnc4	EtOHnc1	EtOHnc2	Galnc1	Galnc2
13		YOL150C	YPDcl2	YPDcl3	EtOHcl1	EtOHcl2	Galcl1	YPDnc1	YPDnc2	YPDnc3	YPDnc4	EtOHnc1	EtOHnc2	Galnc1	Galnc2
13	FAR11	YNL127W	YPDcl2	YPDcl3	EtOHcl1	EtOHcl2	Galcl1	YPDnc1	YPDnc2	YPDnc3	YPDnc4	EtOHnc1	EtOHnc2	Galnc1	Galnc2
13	LRE1	YCL051W	YPDcl2	YPDcl3	EtOHcl1	EtOHcl2	Galcl1	YPDnc1	YPDnc2	YPDnc3	YPDnc4	EtOHnc1	EtOHnc2	Galnc1	Galnc2
13	MMT1	YMR177W	YPDcl2	YPDcl3	EtOHcl1	EtOHcl2	Galcl1	YPDnc1	YPDnc2	YPDnc3	YPDnc4	EtOHnc1	EtOHnc2	Galnc1	Galnc2
13	YPK9	YOR291W	YPDcl2	YPDcl3	EtOHcl1	EtOHcl2	Galcl1	YPDnc1	YPDnc2	YPDnc3	YPDnc4	EtOHnc1	EtOHnc2	Galnc1	Galnc2
13	FLC2	YAL053W	YPDcl2	YPDcl3	EtOHcl1	EtOHcl2	Galcl1	YPDnc1	YPDnc2	YPDnc3	YPDnc4	EtOHnc1	EtOHnc2	Galnc1	Galnc2
13	RPL41B	YDL133C-A	YPDcl2	YPDcl3	EtOHcl1	EtOHcl2	Galcl1	YPDnc1	YPDnc2	YPDnc3	YPDnc4	EtOHnc1	EtOHnc2	Galnc1	Galnc2
13	FBA1	YKL060C	YPDcl2	YPDcl3	EtOHcl1	EtOHcl2	Galcl1	YPDnc1	YPDnc2	YPDnc3	YPDnc4	EtOHnc1	EtOHnc2	Galnc1	Galnc2
13	TAF6	YGL112C	YPDcl2	YPDcl3	EtOHcl1	EtOHcl2	Galcl1	YPDnc1	YPDnc2	YPDnc3	YPDnc4	EtOHnc1	EtOHnc2	Galnc1	Galnc2
13	MBA1	YBR185C	YPDcl2	YPDcl3	EtOHcl1	EtOHcl2	Galcl1	YPDnc1	YPDnc2	YPDnc3	YPDnc4	EtOHnc1	EtOHnc2	Galnc1	Galnc2
13	CKB1	YGL019W	YPDcl2	YPDcl3	EtOHcl1	EtOHcl2	Galcl1	YPDnc1	YPDnc2	YPDnc3	YPDnc4	EtOHnc1	EtOHnc2	Galnc1	Galnc2
13	SLI15	YBR156C	YPDcl2	YPDcl3	EtOHcl1	EtOHcl2	Galcl1	YPDnc1	YPDnc2	YPDnc3	YPDnc4	EtOHnc1	EtOHnc2	Galnc1	Galnc2
13		YMR272W-	YPDcl2	YPDcl3	EtOHcl1	EtOHcl2	Galcl1	YPDnc1	YPDnc2	YPDnc3	YPDnc4	EtOHnc1	EtOHnc2	Galnc1	Galnc2
13	RAD2	YGR258C	YPDcl2	YPDcl3	EtOHcl1	EtOHcl2	Galcl1	YPDnc1	YPDnc2	YPDnc3	YPDnc4	EtOHnc1	EtOHnc2	Galnc1	Galnc2
13	STF2	YGR008C	YPDcl2	YPDcl3	EtOHcl1	EtOHcl2	Galcl1	YPDnc1	YPDnc2	YPDnc3	YPDnc4	EtOHnc1	EtOHnc2	Galnc1	Galnc2
13	NCR1	YPL006W	YPDcl2	YPDcl3	EtOHcl1	EtOHcl2	Galcl1	YPDnc1	YPDnc2	YPDnc3	YPDnc4	EtOHnc1	EtOHnc2	Galnc1	Galnc2
13		YFR054C	YPDcl2	YPDcl3	EtOHcl1	EtOHcl2	Galcl1	YPDnc1	YPDnc2	YPDnc3	YPDnc4	EtOHnc1	EtOHnc2	Galnc1	Galnc2
13	MRP17	YKL003C	YPDcl2	YPDcl3	EtOHcl1	EtOHcl2	Galcl1	YPDnc1	YPDnc2	YPDnc3	YPDnc4	EtOHnc1	EtOHnc2	Galnc1	Galnc2
13	DUG3	YNL191W	YPDcl2	YPDcl3	EtOHcl1	EtOHcl2	Galcl1	YPDnc1	YPDnc2	YPDnc3	YPDnc4	EtOHnc1	EtOHnc2	Galnc1	Galnc2
13	RTG1	YOL067C	YPDcl2	YPDcl3	EtOHcl1	EtOHcl2	Galcl1	YPDnc1	YPDnc2	YPDnc3	YPDnc4	EtOHnc1	EtOHnc2	Galnc1	Galnc2
13		YLL058W	YPDcl2	YPDcl3	EtOHcl1	EtOHcl2	Galcl1	YPDnc1	YPDnc2	YPDnc3	YPDnc4	EtOHnc1	EtOHnc2	Galnc1	Galnc2
13	ARC18	YLR370C	YPDcl2	YPDcl3	EtOHcl1	EtOHcl2	Galcl1	YPDnc1	YPDnc2	YPDnc3	YPDnc4	EtOHnc1	EtOHnc2	Galnc1	Galnc2
13		YDR526C	YPDcl2	YPDcl3	EtOHcl1	EtOHcl2	Galcl1	YPDnc1	YPDnc2	YPDnc3	YPDnc4	EtOHnc1	EtOHnc2	Galnc1	Galnc2
13		YDL121C	YPDcl2	YPDcl3	EtOHcl1	EtOHcl2	Galcl1	YPDnc1	YPDnc2	YPDnc3	YPDnc4	EtOHnc1	EtOHnc2	Galnc1	Galnc2

13	COX5B	YIL111W	YPDcl2	YPDcl3	EtOHcl1	EtOHcl2	Galcl1	YPDnc1	YPDnc2	YPDnc3	YPDnc4	EtOHnc1	EtOHnc2	Galnc1	Galnc2
13	ATP25	YMR098C	YPDcl2	YPDcl3	EtOHcl1	EtOHcl2	Galcl1	YPDnc1	YPDnc2	YPDnc3	YPDnc4	EtOHnc1	EtOHnc2	Galnc1	Galnc2
13	CPR4	YCR069W	YPDcl2	YPDcl3	EtOHcl1	EtOHcl2	Galcl1	YPDnc1	YPDnc2	YPDnc3	YPDnc4	EtOHnc1	EtOHnc2	Galnc1	Galnc2
13		YKL169C	YPDcl2	YPDcl3	EtOHcl1	EtOHcl2	Galcl1	YPDnc1	YPDnc2	YPDnc3	YPDnc4	EtOHnc1	EtOHnc2	Galnc1	Galnc2
13	SIT4	YDL047W	YPDcl2	YPDcl3	EtOHcl1	EtOHcl2	Galcl1	YPDnc1	YPDnc2	YPDnc3	YPDnc4	EtOHnc1	EtOHnc2	Galnc1	Galnc2
13	AUR1	YKL004W	YPDcl2	YPDcl3	EtOHcl1	EtOHcl2	Galcl1	YPDnc1	YPDnc2	YPDnc3	YPDnc4	EtOHnc1	EtOHnc2	Galnc1	Galnc2
13		YLR241W	YPDcl2	YPDcl3	EtOHcl1	EtOHcl2	Galcl1	YPDnc1	YPDnc2	YPDnc3	YPDnc4	EtOHnc1	EtOHnc2	Galnc1	Galnc2
13	SIL1	YOL031C	YPDcl2	YPDcl3	EtOHcl1	EtOHcl2	Galcl1	YPDnc1	YPDnc2	YPDnc3	YPDnc4	EtOHnc1	EtOHnc2	Galnc1	Galnc2
13	PAN1	YIR006C	YPDcl2	YPDcl3	EtOHcl1	EtOHcl2	Galcl1	YPDnc1	YPDnc2	YPDnc3	YPDnc4	EtOHnc1	EtOHnc2	Galnc1	Galnc2
13	RPL25	YOL127W	YPDcl2	YPDcl3	EtOHcl1	EtOHcl2	Galcl1	YPDnc1	YPDnc2	YPDnc3	YPDnc4	EtOHnc1	EtOHnc2	Galnc1	Galnc2
13	MKS1	YNL076W	YPDcl2	YPDcl3	EtOHcl1	EtOHcl2	Galcl1	YPDnc1	YPDnc2	YPDnc3	YPDnc4	EtOHnc1	EtOHnc2	Galnc1	Galnc2
13	SRP101	YDR292C	YPDcl2	YPDcl3	EtOHcl1	EtOHcl2	Galcl1	YPDnc1	YPDnc2	YPDnc3	YPDnc4	EtOHnc1	EtOHnc2	Galnc1	Galnc2
13		YLR101C	YPDcl2	YPDcl3	EtOHcl1	EtOHcl2	Galcl1	YPDnc1	YPDnc2	YPDnc3	YPDnc4	EtOHnc1	EtOHnc2	Galnc1	Galnc2
13		YJR114W	YPDcl2	YPDcl3	EtOHcl1	EtOHcl2	Galcl1	YPDnc1	YPDnc2	YPDnc3	YPDnc4	EtOHnc1	EtOHnc2	Galnc1	Galnc2
13	AIM27	YKL207W	YPDcl2	YPDcl3	EtOHcl1	EtOHcl2	Galcl1	YPDnc1	YPDnc2	YPDnc3	YPDnc4	EtOHnc1	EtOHnc2	Galnc1	Galnc2
13	SSC1	YJR045C	YPDcl2	YPDcl3	EtOHcl1	EtOHcl2	Galcl1	YPDnc1	YPDnc2	YPDnc3	YPDnc4	EtOHnc1	EtOHnc2	Galnc1	Galnc2
13	MCM2	YBL023C	YPDcl2	YPDcl3	EtOHcl1	EtOHcl2	Galcl1	YPDnc1	YPDnc2	YPDnc3	YPDnc4	EtOHnc1	EtOHnc2	Galnc1	Galnc2
13	MTC6	YHR151C	YPDcl2	YPDcl3	EtOHcl1	EtOHcl2	Galcl1	YPDnc1	YPDnc2	YPDnc3	YPDnc4	EtOHnc1	EtOHnc2	Galnc1	Galnc2
13		YEL009C-A	YPDcl2	YPDcl3	EtOHcl1	EtOHcl2	Galcl1	YPDnc1	YPDnc2	YPDnc3	YPDnc4	EtOHnc1	EtOHnc2	Galnc1	Galnc2
13	PHO13	YDL236W	YPDcl2	YPDcl3	EtOHcl1	EtOHcl2	Galcl1	YPDnc1	YPDnc2	YPDnc3	YPDnc4	EtOHnc1	EtOHnc2	Galnc1	Galnc2
13		YCL065W	YPDcl2	YPDcl3	EtOHcl1	EtOHcl2	Galcl1	YPDnc1	YPDnc2	YPDnc3	YPDnc4	EtOHnc1	EtOHnc2	Galnc1	Galnc2
13	JHD1	YER051W	YPDcl2	YPDcl3	EtOHcl1	EtOHcl2	Galcl1	YPDnc1	YPDnc2	YPDnc3	YPDnc4	EtOHnc1	EtOHnc2	Galnc1	Galnc2
13	MHP1	YJL042W	YPDcl2	YPDcl3	EtOHcl1	EtOHcl2	Galcl1	YPDnc1	YPDnc2	YPDnc3	YPDnc4	EtOHnc1	EtOHnc2	Galnc1	Galnc2
13	TDA9	YML081W	YPDcl2	YPDcl3	EtOHcl1	EtOHcl2	Galcl1	YPDnc1	YPDnc2	YPDnc3	YPDnc4	EtOHnc1	EtOHnc2	Galnc1	Galnc2
13		YEL057C	YPDcl2	YPDcl3	EtOHcl1	EtOHcl2	Galcl1	YPDnc1	YPDnc2	YPDnc3	YPDnc4	EtOHnc1	EtOHnc2	Galnc1	Galnc2
13	GVP36	YIL041W	YPDcl2	YPDcl3	EtOHcl1	EtOHcl2	Galcl1	YPDnc1	YPDnc2	YPDnc3	YPDnc4	EtOHnc1	EtOHnc2	Galnc1	Galnc2
13	AIM41	YOR215C	YPDcl2	YPDcl3	EtOHcl1	EtOHcl2	Galcl1	YPDnc1	YPDnc2	YPDnc3	YPDnc4	EtOHnc1	EtOHnc2	Galnc1	Galnc2
13	KTR4	YBR199W	YPDcl2	YPDcl3	EtOHcl1	EtOHcl2	Galcl1	YPDnc1	YPDnc2	YPDnc3	YPDnc4	EtOHnc1	EtOHnc2	Galnc1	Galnc2
13		YGL041W-	YPDcl2	YPDcl3	EtOHcl1	EtOHcl2	Galcl1	YPDnc1	YPDnc2	YPDnc3	YPDnc4	EtOHnc1	EtOHnc2	Galnc1	Galnc2
13	ARG5,6	YER069W	YPDcl2	YPDcl3	EtOHcl1	EtOHcl2	Galcl1	YPDnc1	YPDnc2	YPDnc3	YPDnc4	EtOHnc1	EtOHnc2	Galnc1	Galnc2
13	SPE2	YOL052C	YPDcl2	YPDcl3	EtOHcl1	EtOHcl2	Galcl1	YPDnc1	YPDnc2	YPDnc3	YPDnc4	EtOHnc1	EtOHnc2	Galnc1	Galnc2
13		YNL086W	YPDcl2	YPDcl3	EtOHcl1	EtOHcl2	Galcl1	YPDnc1	YPDnc2	YPDnc3	YPDnc4	EtOHnc1	EtOHnc2	Galnc1	Galnc2
13		YBL010C	YPDcl2	YPDcl3	EtOHcl1	EtOHcl2	Galcl1	YPDnc1	YPDnc2	YPDnc3	YPDnc4	EtOHnc1	EtOHnc2	Galnc1	Galnc2
13		YER148W-	YPDcl2	YPDcl3	EtOHcl1	EtOHcl2	Galcl1	YPDnc1	YPDnc2	YPDnc3	YPDnc4	EtOHnc1	EtOHnc2	Galnc1	Galnc2

13	RMD1	YDL001W	YPDcl2	YPDcl3	EtOHcl1	EtOHcl2	Galcl1	YPDnc1	YPDnc2	YPDnc3	YPDnc4	EtOHnc1	EtOHnc2	Galnc1	Galnc2
13		YBR056W	YPDcl2	YPDcl3	EtOHcl1	EtOHcl2	Galcl1	YPDnc1	YPDnc2	YPDnc3	YPDnc4	EtOHnc1	EtOHnc2	Galnc1	Galnc2
13	SCW10	YMR305C	YPDcl2	YPDcl3	EtOHcl1	EtOHcl2	Galcl1	YPDnc1	YPDnc2	YPDnc3	YPDnc4	EtOHnc1	EtOHnc2	Galnc1	Galnc2
13	NOP58	YOR310C	YPDcl2	YPDcl3	EtOHcl1	EtOHcl2	Galcl1	YPDnc1	YPDnc2	YPDnc3	YPDnc4	EtOHnc1	EtOHnc2	Galnc1	Galnc2
13	CCS1	YMR038C	YPDcl2	YPDcl3	EtOHcl1	EtOHcl2	Galcl1	YPDnc1	YPDnc2	YPDnc3	YPDnc4	EtOHnc1	EtOHnc2	Galnc1	Galnc2
13	ERG4	YGL012W	YPDcl2	YPDcl3	EtOHcl1	EtOHcl2	Galcl1	YPDnc1	YPDnc2	YPDnc3	YPDnc4	EtOHnc1	EtOHnc2	Galnc1	Galnc2
13		YJL020W-A	YPDcl2	YPDcl3	EtOHcl1	EtOHcl2	Galcl1	YPDnc1	YPDnc2	YPDnc3	YPDnc4	EtOHnc1	EtOHnc2	Galnc1	Galnc2
13	YSR3	YKR053C	YPDcl2	YPDcl3	EtOHcl1	EtOHcl2	Galcl1	YPDnc1	YPDnc2	YPDnc3	YPDnc4	EtOHnc1	EtOHnc2	Galnc1	Galnc2
13		YJL007C	YPDcl2	YPDcl3	EtOHcl1	EtOHcl2	Galcl1	YPDnc1	YPDnc2	YPDnc3	YPDnc4	EtOHnc1	EtOHnc2	Galnc1	Galnc2
13		YPR002C-A	YPDcl2	YPDcl3	EtOHcl1	EtOHcl2	Galcl1	YPDnc1	YPDnc2	YPDnc3	YPDnc4	EtOHnc1	EtOHnc2	Galnc1	Galnc2
13	SWP82	YFL049W	YPDcl2	YPDcl3	EtOHcl1	EtOHcl2	Galcl1	YPDnc1	YPDnc2	YPDnc3	YPDnc4	EtOHnc1	EtOHnc2	Galnc1	Galnc2
13	CIT2	YCR005C	YPDcl2	YPDcl3	EtOHcl1	EtOHcl2	Galcl1	YPDnc1	YPDnc2	YPDnc3	YPDnc4	EtOHnc1	EtOHnc2	Galnc1	Galnc2
13	TSA1	YML028W	YPDcl2	YPDcl3	EtOHcl1	EtOHcl2	Galcl1	YPDnc1	YPDnc2	YPDnc3	YPDnc4	EtOHnc1	EtOHnc2	Galnc1	Galnc2
13	ILS1	YBL076C	YPDcl2	YPDcl3	EtOHcl1	EtOHcl2	Galcl1	YPDnc1	YPDnc2	YPDnc3	YPDnc4	EtOHnc1	EtOHnc2	Galnc1	Galnc2
13		YGR064W	YPDcl2	YPDcl3	EtOHcl1	EtOHcl2	Galcl1	YPDnc1	YPDnc2	YPDnc3	YPDnc4	EtOHnc1	EtOHnc2	Galnc1	Galnc2
13		YEL074W	YPDcl2	YPDcl3	EtOHcl1	EtOHcl2	Galcl1	YPDnc1	YPDnc2	YPDnc3	YPDnc4	EtOHnc1	EtOHnc2	Galnc1	Galnc2
13		YHR175W-/	YPDcl2	YPDcl3	EtOHcl1	EtOHcl2	Galcl1	YPDnc1	YPDnc2	YPDnc3	YPDnc4	EtOHnc1	EtOHnc2	Galnc1	Galnc2
13	DMA2	YNL116W	YPDcl2	YPDcl3	EtOHcl1	EtOHcl2	Galcl1	YPDnc1	YPDnc2	YPDnc3	YPDnc4	EtOHnc1	EtOHnc2	Galnc1	Galnc2
13	MAM3	YOL060C	YPDcl2	YPDcl3	EtOHcl1	EtOHcl2	Galcl1	YPDnc1	YPDnc2	YPDnc3	YPDnc4	EtOHnc1	EtOHnc2	Galnc1	Galnc2
13	DOT5	YIL010W	YPDcl2	YPDcl3	EtOHcl1	EtOHcl2	Galcl1	YPDnc1	YPDnc2	YPDnc3	YPDnc4	EtOHnc1	EtOHnc2	Galnc1	Galnc2
13	PSH1	YOL054W	YPDcl2	YPDcl3	EtOHcl1	EtOHcl2	Galcl1	YPDnc1	YPDnc2	YPDnc3	YPDnc4	EtOHnc1	EtOHnc2	Galnc1	Galnc2
13		YML094C-/	YPDcl2	YPDcl3	EtOHcl1	EtOHcl2	Galcl1	YPDnc1	YPDnc2	YPDnc3	YPDnc4	EtOHnc1	EtOHnc2	Galnc1	Galnc2
13	LYS5	YGL154C	YPDcl2	YPDcl3	EtOHcl1	EtOHcl2	Galcl1	YPDnc1	YPDnc2	YPDnc3	YPDnc4	EtOHnc1	EtOHnc2	Galnc1	Galnc2
13	MDM38	YOL027C	YPDcl2	YPDcl3	EtOHcl1	EtOHcl2	Galcl1	YPDnc1	YPDnc2	YPDnc3	YPDnc4	EtOHnc1	EtOHnc2	Galnc1	Galnc2
13	UTP15	YMR093W	YPDcl2	YPDcl3	EtOHcl1	EtOHcl2	Galcl1	YPDnc1	YPDnc2	YPDnc3	YPDnc4	EtOHnc1	EtOHnc2	Galnc1	Galnc2
13	HXT8	YJL214W	YPDcl2	YPDcl3	EtOHcl1	EtOHcl2	Galcl1	YPDnc1	YPDnc2	YPDnc3	YPDnc4	EtOHnc1	EtOHnc2	Galnc1	Galnc2
13	NUC1	YJL208C	YPDcl2	YPDcl3	EtOHcl1	EtOHcl2	Galcl1	YPDnc1	YPDnc2	YPDnc3	YPDnc4	EtOHnc1	EtOHnc2	Galnc1	Galnc2
13		YIL161W	YPDcl2	YPDcl3	EtOHcl1	EtOHcl2	Galcl1	YPDnc1	YPDnc2	YPDnc3	YPDnc4	EtOHnc1	EtOHnc2	Galnc1	Galnc2
13	BMH1	YER177W	YPDcl2	YPDcl3	EtOHcl1	EtOHcl2	Galcl1	YPDnc1	YPDnc2	YPDnc3	YPDnc4	EtOHnc1	EtOHnc2	Galnc1	Galnc2
13	MYO4	YAL029C	YPDcl2	YPDcl3	EtOHcl1	EtOHcl2	Galcl1	YPDnc1	YPDnc2	YPDnc3	YPDnc4	EtOHnc1	EtOHnc2	Galnc1	Galnc2
13		YHR028W-/	YPDcl2	YPDcl3	EtOHcl1	EtOHcl2	Galcl1	YPDnc1	YPDnc2	YPDnc3	YPDnc4	EtOHnc1	EtOHnc2	Galnc1	Galnc2
13	SAM50	YNL026W	YPDcl2	YPDcl3	EtOHcl1	EtOHcl2	Galcl1	YPDnc1	YPDnc2	YPDnc3	YPDnc4	EtOHnc1	EtOHnc2	Galnc1	Galnc2
13	HDA3	YPR179C	YPDcl2	YPDcl3	EtOHcl1	EtOHcl2	Galcl1	YPDnc1	YPDnc2	YPDnc3	YPDnc4	EtOHnc1	EtOHnc2	Galnc1	Galnc2
13	ISC1	YER019W	YPDcl2	YPDcl3	EtOHcl1	EtOHcl2	Galcl1	YPDnc1	YPDnc2	YPDnc3	YPDnc4	EtOHnc1	EtOHnc2	Galnc1	Galnc2

13	BRE2	YLR015W	YPDcl2	YPDcl3	EtOHcl1	EtOHcl2	Galcl1	YPDnc1	YPDnc2	YPDnc3	YPDnc4	EtOHnc1	EtOHnc2	Galnc1	Galnc2
13		YDL009C	YPDcl2	YPDcl3	EtOHcl1	EtOHcl2	Galcl1	YPDnc1	YPDnc2	YPDnc3	YPDnc4	EtOHnc1	EtOHnc2	Galnc1	Galnc2
13		YIL091C	YPDcl2	YPDcl3	EtOHcl1	EtOHcl2	Galcl1	YPDnc1	YPDnc2	YPDnc3	YPDnc4	EtOHnc1	EtOHnc2	Galnc1	Galnc2
13		YGL230C	YPDcl2	YPDcl3	EtOHcl1	EtOHcl2	Galcl1	YPDnc1	YPDnc2	YPDnc3	YPDnc4	EtOHnc1	EtOHnc2	Galnc1	Galnc2
13		YLR311C	YPDcl2	YPDcl3	EtOHcl1	EtOHcl2	Galcl1	YPDnc1	YPDnc2	YPDnc3	YPDnc4	EtOHnc1	EtOHnc2	Galnc1	Galnc2
13	PRS3	YHL011C	YPDcl2	YPDcl3	EtOHcl1	EtOHcl2	Galcl1	YPDnc1	YPDnc2	YPDnc3	YPDnc4	EtOHnc1	EtOHnc2	Galnc1	Galnc2
13	DET1	YDR051C	YPDcl2	YPDcl3	EtOHcl1	EtOHcl2	Galcl1	YPDnc1	YPDnc2	YPDnc3	YPDnc4	EtOHnc1	EtOHnc2	Galnc1	Galnc2
13	MRS6	YOR370C	YPDcl2	YPDcl3	EtOHcl1	EtOHcl2	Galcl1	YPDnc1	YPDnc2	YPDnc3	YPDnc4	EtOHnc1	EtOHnc2	Galnc1	Galnc2
13	NCB2	YDR397C	YPDcl2	YPDcl3	EtOHcl1	EtOHcl2	Galcl1	YPDnc1	YPDnc2	YPDnc3	YPDnc4	EtOHnc1	EtOHnc2	Galnc1	Galnc2
13	CBP6	YBR120C	YPDcl2	YPDcl3	EtOHcl1	EtOHcl2	Galcl1	YPDnc1	YPDnc2	YPDnc3	YPDnc4	EtOHnc1	EtOHnc2	Galnc1	Galnc2
13		YMR084W	YPDcl2	YPDcl3	EtOHcl1	EtOHcl2	Galcl1	YPDnc1	YPDnc2	YPDnc3	YPDnc4	EtOHnc1	EtOHnc2	Galnc1	Galnc2
13		YHR210C	YPDcl2	YPDcl3	EtOHcl1	EtOHcl2	Galcl1	YPDnc1	YPDnc2	YPDnc3	YPDnc4	EtOHnc1	EtOHnc2	Galnc1	Galnc2
13	TAF1	YGR274C	YPDcl2	YPDcl3	EtOHcl1	EtOHcl2	Galcl1	YPDnc1	YPDnc2	YPDnc3	YPDnc4	EtOHnc1	EtOHnc2	Galnc1	Galnc2
13	BLM10	YFL007W	YPDcl2	YPDcl3	EtOHcl1	EtOHcl2	Galcl1	YPDnc1	YPDnc2	YPDnc3	YPDnc4	EtOHnc1	EtOHnc2	Galnc1	Galnc2
13	ATG13	YPR185W	YPDcl2	YPDcl3	EtOHcl1	EtOHcl2	Galcl1	YPDnc1	YPDnc2	YPDnc3	YPDnc4	EtOHnc1	EtOHnc2	Galnc1	Galnc2
13	PXR1	YGR280C	YPDcl2	YPDcl3	EtOHcl1	EtOHcl2	Galcl1	YPDnc1	YPDnc2	YPDnc3	YPDnc4	EtOHnc1	EtOHnc2	Galnc1	Galnc2
13	TAF8	YML114C	YPDcl2	YPDcl3	EtOHcl1	EtOHcl2	Galcl1	YPDnc1	YPDnc2	YPDnc3	YPDnc4	EtOHnc1	EtOHnc2	Galnc1	Galnc2
13	MAP1	YLR244C	YPDcl2	YPDcl3	EtOHcl1	EtOHcl2	Galcl1	YPDnc1	YPDnc2	YPDnc3	YPDnc4	EtOHnc1	EtOHnc2	Galnc1	Galnc2
13	REC102	YLR329W	YPDcl2	YPDcl3	EtOHcl1	EtOHcl2	Galcl1	YPDnc1	YPDnc2	YPDnc3	YPDnc4	EtOHnc1	EtOHnc2	Galnc1	Galnc2
13		YFR056C	YPDcl2	YPDcl3	EtOHcl1	EtOHcl2	Galcl1	YPDnc1	YPDnc2	YPDnc3	YPDnc4	EtOHnc1	EtOHnc2	Galnc1	Galnc2
13	SGN1	YIR001C	YPDcl2	YPDcl3	EtOHcl1	EtOHcl2	Galcl1	YPDnc1	YPDnc2	YPDnc3	YPDnc4	EtOHnc1	EtOHnc2	Galnc1	Galnc2
13	ASP1	YDR321W	YPDcl2	YPDcl3	EtOHcl1	EtOHcl2	Galcl1	YPDnc1	YPDnc2	YPDnc3	YPDnc4	EtOHnc1	EtOHnc2	Galnc1	Galnc2
13	SPO21	YOL091W	YPDcl2	YPDcl3	EtOHcl1	EtOHcl2	Galcl1	YPDnc1	YPDnc2	YPDnc3	YPDnc4	EtOHnc1	EtOHnc2	Galnc1	Galnc2
13		YNL170W	YPDcl2	YPDcl3	EtOHcl1	EtOHcl2	Galcl1	YPDnc1	YPDnc2	YPDnc3	YPDnc4	EtOHnc1	EtOHnc2	Galnc1	Galnc2
13	TUB2	YFL037W	YPDcl2	YPDcl3	EtOHcl1	EtOHcl2	Galcl1	YPDnc1	YPDnc2	YPDnc3	YPDnc4	EtOHnc1	EtOHnc2	Galnc1	Galnc2
13	UTH1	YKR042W	YPDcl2	YPDcl3	EtOHcl1	EtOHcl2	Galcl1	YPDnc1	YPDnc2	YPDnc3	YPDnc4	EtOHnc1	EtOHnc2	Galnc1	Galnc2
13		YGL123C-A	YPDcl2	YPDcl3	EtOHcl1	EtOHcl2	Galcl1	YPDnc1	YPDnc2	YPDnc3	YPDnc4	EtOHnc1	EtOHnc2	Galnc1	Galnc2
13	ERF2	YLR246W	YPDcl2	YPDcl3	EtOHcl1	EtOHcl2	Galcl1	YPDnc1	YPDnc2	YPDnc3	YPDnc4	EtOHnc1	EtOHnc2	Galnc1	Galnc2
13		YDR169C-A	YPDcl2	YPDcl3	EtOHcl1	EtOHcl2	Galcl1	YPDnc1	YPDnc2	YPDnc3	YPDnc4	EtOHnc1	EtOHnc2	Galnc1	Galnc2
13	PUP1	YOR157C	YPDcl2	YPDcl3	EtOHcl1	EtOHcl2	Galcl1	YPDnc1	YPDnc2	YPDnc3	YPDnc4	EtOHnc1	EtOHnc2	Galnc1	Galnc2
13	OST6	YML019W	YPDcl2	YPDcl3	EtOHcl1	EtOHcl2	Galcl1	YPDnc1	YPDnc2	YPDnc3	YPDnc4	EtOHnc1	EtOHnc2	Galnc1	Galnc2
13	TCA17	YEL048C	YPDcl2	YPDcl3	EtOHcl1	EtOHcl2	Galcl1	YPDnc1	YPDnc2	YPDnc3	YPDnc4	EtOHnc1	EtOHnc2	Galnc1	Galnc2
13	PET112	YBL080C	YPDcl2	YPDcl3	EtOHcl1	EtOHcl2	Galcl1	YPDnc1	YPDnc2	YPDnc3	YPDnc4	EtOHnc1	EtOHnc2	Galnc1	Galnc2
13		YMR182W-	YPDcl2	YPDcl3	EtOHcl1	EtOHcl2	Galcl1	YPDnc1	YPDnc2	YPDnc3	YPDnc4	EtOHnc1	EtOHnc2	Galnc1	Galnc2

13	GAL4	YPL248C	YPDcl2	YPDcl3	EtOHcl1	EtOHcl2	Galcl1	YPDnc1	YPDnc2	YPDnc3	YPDnc4	EtOHnc1	EtOHnc2	Galnc1	Galnc2
13	CHZ1	YER030W	YPDcl2	YPDcl3	EtOHcl1	EtOHcl2	Galcl1	YPDnc1	YPDnc2	YPDnc3	YPDnc4	EtOHnc1	EtOHnc2	Galnc1	Galnc2
13	GAL83	YER027C	YPDcl2	YPDcl3	EtOHcl1	EtOHcl2	Galcl1	YPDnc1	YPDnc2	YPDnc3	YPDnc4	EtOHnc1	EtOHnc2	Galnc1	Galnc2
13		YER175W-/	YPDcl2	YPDcl3	EtOHcl1	EtOHcl2	Galcl1	YPDnc1	YPDnc2	YPDnc3	YPDnc4	EtOHnc1	EtOHnc2	Galnc1	Galnc2
13	LOC1	YFR001W	YPDcl2	YPDcl3	EtOHcl1	EtOHcl2	Galcl1	YPDnc1	YPDnc2	YPDnc3	YPDnc4	EtOHnc1	EtOHnc2	Galnc1	Galnc2
13		YDL242W	YPDcl2	YPDcl3	EtOHcl1	EtOHcl2	Galcl1	YPDnc1	YPDnc2	YPDnc3	YPDnc4	EtOHnc1	EtOHnc2	Galnc1	Galnc2
13	RPN2	YIL075C	YPDcl2	YPDcl3	EtOHcl1	EtOHcl2	Galcl1	YPDnc1	YPDnc2	YPDnc3	YPDnc4	EtOHnc1	EtOHnc2	Galnc1	Galnc2
13	PAM16	YJL104W	YPDcl2	YPDcl3	EtOHcl1	EtOHcl2	Galcl1	YPDnc1	YPDnc2	YPDnc3	YPDnc4	EtOHnc1	EtOHnc2	Galnc1	Galnc2
13	MCM6	YGL201C	YPDcl2	YPDcl3	EtOHcl1	EtOHcl2	Galcl1	YPDnc1	YPDnc2	YPDnc3	YPDnc4	EtOHnc1	EtOHnc2	Galnc1	Galnc2
13	MRPS35	YGR165W	YPDcl2	YPDcl3	EtOHcl1	EtOHcl2	Galcl1	YPDnc1	YPDnc2	YPDnc3	YPDnc4	EtOHnc1	EtOHnc2	Galnc1	Galnc2
13	RPL31A	YDL075W	YPDcl2	YPDcl3	EtOHcl1	EtOHcl2	Galcl1	YPDnc1	YPDnc2	YPDnc3	YPDnc4	EtOHnc1	EtOHnc2	Galnc1	Galnc2
13	TLG1	YDR468C	YPDcl2	YPDcl3	EtOHcl1	EtOHcl2	Galcl1	YPDnc1	YPDnc2	YPDnc3	YPDnc4	EtOHnc1	EtOHnc2	Galnc1	Galnc2
13	IMG1	YCR046C	YPDcl2	YPDcl3	EtOHcl1	EtOHcl2	Galcl1	YPDnc1	YPDnc2	YPDnc3	YPDnc4	EtOHnc1	EtOHnc2	Galnc1	Galnc2
13	CYC7	YEL039C	YPDcl2	YPDcl3	EtOHcl1	EtOHcl2	Galcl1	YPDnc1	YPDnc2	YPDnc3	YPDnc4	EtOHnc1	EtOHnc2	Galnc1	Galnc2
13		YDL144C	YPDcl2	YPDcl3	EtOHcl1	EtOHcl2	Galcl1	YPDnc1	YPDnc2	YPDnc3	YPDnc4	EtOHnc1	EtOHnc2	Galnc1	Galnc2
13	BRR6	YGL247W	YPDcl2	YPDcl3	EtOHcl1	EtOHcl2	Galcl1	YPDnc1	YPDnc2	YPDnc3	YPDnc4	EtOHnc1	EtOHnc2	Galnc1	Galnc2
13	VPS27	YNR006W	YPDcl2	YPDcl3	EtOHcl1	EtOHcl2	Galcl1	YPDnc1	YPDnc2	YPDnc3	YPDnc4	EtOHnc1	EtOHnc2	Galnc1	Galnc2
13	RPL16A	YIL133C	YPDcl2	YPDcl3	EtOHcl1	EtOHcl2	Galcl1	YPDnc1	YPDnc2	YPDnc3	YPDnc4	EtOHnc1	EtOHnc2	Galnc1	Galnc2
13	NAF1	YNL124W	YPDcl2	YPDcl3	EtOHcl1	EtOHcl2	Galcl1	YPDnc1	YPDnc2	YPDnc3	YPDnc4	EtOHnc1	EtOHnc2	Galnc1	Galnc2
13	TTI2	YJR136C	YPDcl2	YPDcl3	EtOHcl1	EtOHcl2	Galcl1	YPDnc1	YPDnc2	YPDnc3	YPDnc4	EtOHnc1	EtOHnc2	Galnc1	Galnc2
13		YJR079W	YPDcl2	YPDcl3	EtOHcl1	EtOHcl2	Galcl1	YPDnc1	YPDnc2	YPDnc3	YPDnc4	EtOHnc1	EtOHnc2	Galnc1	Galnc2
13		YDL158C	YPDcl2	YPDcl3	EtOHcl1	EtOHcl2	Galcl1	YPDnc1	YPDnc2	YPDnc3	YPDnc4	EtOHnc1	EtOHnc2	Galnc1	Galnc2
13	FPK1	YNR047W	YPDcl2	YPDcl3	EtOHcl1	EtOHcl2	Galcl1	YPDnc1	YPDnc2	YPDnc3	YPDnc4	EtOHnc1	EtOHnc2	Galnc1	Galnc2
13	EPT1	YHR123W	YPDcl2	YPDcl3	EtOHcl1	EtOHcl2	Galcl1	YPDnc1	YPDnc2	YPDnc3	YPDnc4	EtOHnc1	EtOHnc2	Galnc1	Galnc2
13		YIL092W	YPDcl2	YPDcl3	EtOHcl1	EtOHcl2	Galcl1	YPDnc1	YPDnc2	YPDnc3	YPDnc4	EtOHnc1	EtOHnc2	Galnc1	Galnc2
13	BNA1	YJR025C	YPDcl2	YPDcl3	EtOHcl1	EtOHcl2	Galcl1	YPDnc1	YPDnc2	YPDnc3	YPDnc4	EtOHnc1	EtOHnc2	Galnc1	Galnc2
13	HTB2	YBL002W	YPDcl2	YPDcl3	EtOHcl1	EtOHcl2	Galcl1	YPDnc1	YPDnc2	YPDnc3	YPDnc4	EtOHnc1	EtOHnc2	Galnc1	Galnc2
13	SWI1	YPL016W	YPDcl2	YPDcl3	EtOHcl1	EtOHcl2	Galcl1	YPDnc1	YPDnc2	YPDnc3	YPDnc4	EtOHnc1	EtOHnc2	Galnc1	Galnc2
13	ETP1	YHL010C	YPDcl2	YPDcl3	EtOHcl1	EtOHcl2	Galcl1	YPDnc1	YPDnc2	YPDnc3	YPDnc4	EtOHnc1	EtOHnc2	Galnc1	Galnc2
13	MED4	YOR174W	YPDcl2	YPDcl3	EtOHcl1	EtOHcl2	Galcl1	YPDnc1	YPDnc2	YPDnc3	YPDnc4	EtOHnc1	EtOHnc2	Galnc1	Galnc2
13	THI80	YOR143C	YPDcl2	YPDcl3	EtOHcl1	EtOHcl2	Galcl1	YPDnc1	YPDnc2	YPDnc3	YPDnc4	EtOHnc1	EtOHnc2	Galnc1	Galnc2
13	GLO3	YER122C	YPDcl2	YPDcl3	EtOHcl1	EtOHcl2	Galcl1	YPDnc1	YPDnc2	YPDnc3	YPDnc4	EtOHnc1	EtOHnc2	Galnc1	Galnc2
13		YLL032C	YPDcl2	YPDcl3	EtOHcl1	EtOHcl2	Galcl1	YPDnc1	YPDnc2	YPDnc3	YPDnc4	EtOHnc1	EtOHnc2	Galnc1	Galnc2
13	ATC1	YDR184C	YPDcl2	YPDcl3	EtOHcl1	EtOHcl2	Galcl1	YPDnc1	YPDnc2	YPDnc3	YPDnc4	EtOHnc1	EtOHnc2	Galnc1	Galnc2

13	ERS1	YCR075C	YPDcl2	YPDcl3	EtOHcl1	EtOHcl2	Galcl1	YPDnc1	YPDnc2	YPDnc3	YPDnc4	EtOHnc1	EtOHnc2	Galnc1	Galnc2
13	MRPL6	YHR147C	YPDcl2	YPDcl3	EtOHcl1	EtOHcl2	Galcl1	YPDnc1	YPDnc2	YPDnc3	YPDnc4	EtOHnc1	EtOHnc2	Galnc1	Galnc2
13		YPR012W	YPDcl2	YPDcl3	EtOHcl1	EtOHcl2	Galcl1	YPDnc1	YPDnc2	YPDnc3	YPDnc4	EtOHnc1	EtOHnc2	Galnc1	Galnc2
13	MOG1	YJR074W	YPDcl2	YPDcl3	EtOHcl1	EtOHcl2	Galcl1	YPDnc1	YPDnc2	YPDnc3	YPDnc4	EtOHnc1	EtOHnc2	Galnc1	Galnc2
13		YPL162C	YPDcl2	YPDcl3	EtOHcl1	EtOHcl2	Galcl1	YPDnc1	YPDnc2	YPDnc3	YPDnc4	EtOHnc1	EtOHnc2	Galnc1	Galnc2
13	RAT1	YOR048C	YPDcl2	YPDcl3	EtOHcl1	EtOHcl2	Galcl1	YPDnc1	YPDnc2	YPDnc3	YPDnc4	EtOHnc1	EtOHnc2	Galnc1	Galnc2
13		YNL295W	YPDcl2	YPDcl3	EtOHcl1	EtOHcl2	Galcl1	YPDnc1	YPDnc2	YPDnc3	YPDnc4	EtOHnc1	EtOHnc2	Galnc1	Galnc2
13		YHL041W	YPDcl2	YPDcl3	EtOHcl1	EtOHcl2	Galcl1	YPDnc1	YPDnc2	YPDnc3	YPDnc4	EtOHnc1	EtOHnc2	Galnc1	Galnc2
13	QRI5	YLR204W	YPDcl2	YPDcl3	EtOHcl1	EtOHcl2	Galcl1	YPDnc1	YPDnc2	YPDnc3	YPDnc4	EtOHnc1	EtOHnc2	Galnc1	Galnc2
13	THR1	YHR025W	YPDcl2	YPDcl3	EtOHcl1	EtOHcl2	Galcl1	YPDnc1	YPDnc2	YPDnc3	YPDnc4	EtOHnc1	EtOHnc2	Galnc1	Galnc2
13	RTC4	YNL254C	YPDcl2	YPDcl3	EtOHcl1	EtOHcl2	Galcl1	YPDnc1	YPDnc2	YPDnc3	YPDnc4	EtOHnc1	EtOHnc2	Galnc1	Galnc2
13	AGP2	YBR132C	YPDcl2	YPDcl3	EtOHcl1	EtOHcl2	Galcl1	YPDnc1	YPDnc2	YPDnc3	YPDnc4	EtOHnc1	EtOHnc2	Galnc1	Galnc2
13	CEF1	YMR213W	YPDcl2	YPDcl3	EtOHcl1	EtOHcl2	Galcl1	YPDnc1	YPDnc2	YPDnc3	YPDnc4	EtOHnc1	EtOHnc2	Galnc1	Galnc2
13	GCD6	YDR211W	YPDcl2	YPDcl3	EtOHcl1	EtOHcl2	Galcl1	YPDnc1	YPDnc2	YPDnc3	YPDnc4	EtOHnc1	EtOHnc2	Galnc1	Galnc2
13	HSF1	YGL073W	YPDcl2	YPDcl3	EtOHcl1	EtOHcl2	Galcl1	YPDnc1	YPDnc2	YPDnc3	YPDnc4	EtOHnc1	EtOHnc2	Galnc1	Galnc2
13	MRPL13	YKR006C	YPDcl2	YPDcl3	EtOHcl1	EtOHcl2	Galcl1	YPDnc1	YPDnc2	YPDnc3	YPDnc4	EtOHnc1	EtOHnc2	Galnc1	Galnc2
13	ATP15	YPL271W	YPDcl2	YPDcl3	EtOHcl1	EtOHcl2	Galcl1	YPDnc1	YPDnc2	YPDnc3	YPDnc4	EtOHnc1	EtOHnc2	Galnc1	Galnc2
13		YDR370C	YPDcl2	YPDcl3	EtOHcl1	EtOHcl2	Galcl1	YPDnc1	YPDnc2	YPDnc3	YPDnc4	EtOHnc1	EtOHnc2	Galnc1	Galnc2
13	FAA2	YER015W	YPDcl2	YPDcl3	EtOHcl1	EtOHcl2	Galcl1	YPDnc1	YPDnc2	YPDnc3	YPDnc4	EtOHnc1	EtOHnc2	Galnc1	Galnc2
13	LGE1	YPL055C	YPDcl2	YPDcl3	EtOHcl1	EtOHcl2	Galcl1	YPDnc1	YPDnc2	YPDnc3	YPDnc4	EtOHnc1	EtOHnc2	Galnc1	Galnc2
13	GPI8	YDR331W	YPDcl2	YPDcl3	EtOHcl1	EtOHcl2	Galcl1	YPDnc1	YPDnc2	YPDnc3	YPDnc4	EtOHnc1	EtOHnc2	Galnc1	Galnc2
13	SNU56	YDR240C	YPDcl2	YPDcl3	EtOHcl1	EtOHcl2	Galcl1	YPDnc1	YPDnc2	YPDnc3	YPDnc4	EtOHnc1	EtOHnc2	Galnc1	Galnc2
13		YCL042W	YPDcl2	YPDcl3	EtOHcl1	EtOHcl2	Galcl1	YPDnc1	YPDnc2	YPDnc3	YPDnc4	EtOHnc1	EtOHnc2	Galnc1	Galnc2
13	AIM6	YDL237W	YPDcl2	YPDcl3	EtOHcl1	EtOHcl2	Galcl1	YPDnc1	YPDnc2	YPDnc3	YPDnc4	EtOHnc1	EtOHnc2	Galnc1	Galnc2
13	FRA1	YLL029W	YPDcl2	YPDcl3	EtOHcl1	EtOHcl2	Galcl1	YPDnc1	YPDnc2	YPDnc3	YPDnc4	EtOHnc1	EtOHnc2	Galnc1	Galnc2
13		YJL163C	YPDcl2	YPDcl3	EtOHcl1	EtOHcl2	Galcl1	YPDnc1	YPDnc2	YPDnc3	YPDnc4	EtOHnc1	EtOHnc2	Galnc1	Galnc2
13	TRS20	YBR254C	YPDcl2	YPDcl3	EtOHcl1	EtOHcl2	Galcl1	YPDnc1	YPDnc2	YPDnc3	YPDnc4	EtOHnc1	EtOHnc2	Galnc1	Galnc2
13	RPS11A	YDR025W	YPDcl2	YPDcl3	EtOHcl1	EtOHcl2	Galcl1	YPDnc1	YPDnc2	YPDnc3	YPDnc4	EtOHnc1	EtOHnc2	Galnc1	Galnc2
13	POR1	YNL055C	YPDcl2	YPDcl3	EtOHcl1	EtOHcl2	Galcl1	YPDnc1	YPDnc2	YPDnc3	YPDnc4	EtOHnc1	EtOHnc2	Galnc1	Galnc2
13	ARP9	YMR033W	YPDcl2	YPDcl3	EtOHcl1	EtOHcl2	Galcl1	YPDnc1	YPDnc2	YPDnc3	YPDnc4	EtOHnc1	EtOHnc2	Galnc1	Galnc2
13	FIG1	YBR040W	YPDcl2	YPDcl3	EtOHcl1	EtOHcl2	Galcl1	YPDnc1	YPDnc2	YPDnc3	YPDnc4	EtOHnc1	EtOHnc2	Galnc1	Galnc2
13		YLR063W	YPDcl2	YPDcl3	EtOHcl1	EtOHcl2	Galcl1	YPDnc1	YPDnc2	YPDnc3	YPDnc4	EtOHnc1	EtOHnc2	Galnc1	Galnc2
13		YJL132W	YPDcl2	YPDcl3	EtOHcl1	EtOHcl2	Galcl1	YPDnc1	YPDnc2	YPDnc3	YPDnc4	EtOHnc1	EtOHnc2	Galnc1	Galnc2
13		YPR022C	YPDcl2	YPDcl3	EtOHcl1	EtOHcl2	Galcl1	YPDnc1	YPDnc2	YPDnc3	YPDnc4	EtOHnc1	EtOHnc2	Galnc1	Galnc2

13	SEC26	YDR238C	YPDcl2	YPDcl3	EtOHcl1	EtOHcl2	Galcl1	YPDnc1	YPDnc2	YPDnc3	YPDnc4	EtOHnc1	EtOHnc2	Galnc1	Galnc2
13	RPH1	YER169W	YPDcl2	YPDcl3	EtOHcl1	EtOHcl2	Galcl1	YPDnc1	YPDnc2	YPDnc3	YPDnc4	EtOHnc1	EtOHnc2	Galnc1	Galnc2
13	SEC11	YIR022W	YPDcl2	YPDcl3	EtOHcl1	EtOHcl2	Galcl1	YPDnc1	YPDnc2	YPDnc3	YPDnc4	EtOHnc1	EtOHnc2	Galnc1	Galnc2
13	KEL1	YHR158C	YPDcl2	YPDcl3	EtOHcl1	EtOHcl2	Galcl1	YPDnc1	YPDnc2	YPDnc3	YPDnc4	EtOHnc1	EtOHnc2	Galnc1	Galnc2
13		YNL042W-I	YPDcl2	YPDcl3	EtOHcl1	EtOHcl2	Galcl1	YPDnc1	YPDnc2	YPDnc3	YPDnc4	EtOHnc1	EtOHnc2	Galnc1	Galnc2
13		YOR029W	YPDcl2	YPDcl3	EtOHcl1	EtOHcl2	Galcl1	YPDnc1	YPDnc2	YPDnc3	YPDnc4	EtOHnc1	EtOHnc2	Galnc1	Galnc2
13	YKT6	YKL196C	YPDcl2	YPDcl3	EtOHcl1	EtOHcl2	Galcl1	YPDnc1	YPDnc2	YPDnc3	YPDnc4	EtOHnc1	EtOHnc2	Galnc1	Galnc2
13	BET3	YKR068C	YPDcl2	YPDcl3	EtOHcl1	EtOHcl2	Galcl1	YPDnc1	YPDnc2	YPDnc3	YPDnc4	EtOHnc1	EtOHnc2	Galnc1	Galnc2
13	ECM1	YAL059W	YPDcl2	YPDcl3	EtOHcl1	EtOHcl2	Galcl1	YPDnc1	YPDnc2	YPDnc3	YPDnc4	EtOHnc1	EtOHnc2	Galnc1	Galnc2
13	PEX32	YBR168W	YPDcl2	YPDcl3	EtOHcl1	EtOHcl2	Galcl1	YPDnc1	YPDnc2	YPDnc3	YPDnc4	EtOHnc1	EtOHnc2	Galnc1	Galnc2
13	NUM1	YDR150W	YPDcl2	YPDcl3	EtOHcl1	EtOHcl2	Galcl1	YPDnc1	YPDnc2	YPDnc3	YPDnc4	EtOHnc1	EtOHnc2	Galnc1	Galnc2
13		YIL105W-A	YPDcl2	YPDcl3	EtOHcl1	EtOHcl2	Galcl1	YPDnc1	YPDnc2	YPDnc3	YPDnc4	EtOHnc1	EtOHnc2	Galnc1	Galnc2
13		YIL134C-A	YPDcl2	YPDcl3	EtOHcl1	EtOHcl2	Galcl1	YPDnc1	YPDnc2	YPDnc3	YPDnc4	EtOHnc1	EtOHnc2	Galnc1	Galnc2
13		YJL135W	YPDcl2	YPDcl3	EtOHcl1	EtOHcl2	Galcl1	YPDnc1	YPDnc2	YPDnc3	YPDnc4	EtOHnc1	EtOHnc2	Galnc1	Galnc2
13	CLB2	YPR119W	YPDcl2	YPDcl3	EtOHcl1	EtOHcl2	Galcl1	YPDnc1	YPDnc2	YPDnc3	YPDnc4	EtOHnc1	EtOHnc2	Galnc1	Galnc2
13	UGA2	YBR006W	YPDcl2	YPDcl3	EtOHcl1	EtOHcl2	Galcl1	YPDnc1	YPDnc2	YPDnc3	YPDnc4	EtOHnc1	EtOHnc2	Galnc1	Galnc2
13	MRI1	YPR118W	YPDcl2	YPDcl3	EtOHcl1	EtOHcl2	Galcl1	YPDnc1	YPDnc2	YPDnc3	YPDnc4	EtOHnc1	EtOHnc2	Galnc1	Galnc2
13	SCT1	YBL011W	YPDcl2	YPDcl3	EtOHcl1	EtOHcl2	Galcl1	YPDnc1	YPDnc2	YPDnc3	YPDnc4	EtOHnc1	EtOHnc2	Galnc1	Galnc2
13	IES4	YOR189W	YPDcl2	YPDcl3	EtOHcl1	EtOHcl2	Galcl1	YPDnc1	YPDnc2	YPDnc3	YPDnc4	EtOHnc1	EtOHnc2	Galnc1	Galnc2
13	ESL2	YKR096W	YPDcl2	YPDcl3	EtOHcl1	EtOHcl2	Galcl1	YPDnc1	YPDnc2	YPDnc3	YPDnc4	EtOHnc1	EtOHnc2	Galnc1	Galnc2
13		YDL062W	YPDcl2	YPDcl3	EtOHcl1	EtOHcl2	Galcl1	YPDnc1	YPDnc2	YPDnc3	YPDnc4	EtOHnc1	EtOHnc2	Galnc1	Galnc2
13		YOR015W	YPDcl2	YPDcl3	EtOHcl1	EtOHcl2	Galcl1	YPDnc1	YPDnc2	YPDnc3	YPDnc4	EtOHnc1	EtOHnc2	Galnc1	Galnc2
13	DSS1	YMR287C	YPDcl2	YPDcl3	EtOHcl1	EtOHcl2	Galcl1	YPDnc1	YPDnc2	YPDnc3	YPDnc4	EtOHnc1	EtOHnc2	Galnc1	Galnc2
13	GLN3	YER040W	YPDcl2	YPDcl3	EtOHcl1	EtOHcl2	Galcl1	YPDnc1	YPDnc2	YPDnc3	YPDnc4	EtOHnc1	EtOHnc2	Galnc1	Galnc2
13		YMR153C-/	YPDcl2	YPDcl3	EtOHcl1	EtOHcl2	Galcl1	YPDnc1	YPDnc2	YPDnc3	YPDnc4	EtOHnc1	EtOHnc2	Galnc1	Galnc2
13	IFH1	YLR223C	YPDcl2	YPDcl3	EtOHcl1	EtOHcl2	Galcl1	YPDnc1	YPDnc2	YPDnc3	YPDnc4	EtOHnc1	EtOHnc2	Galnc1	Galnc2
13	SRC1	YML034W	YPDcl2	YPDcl3	EtOHcl1	EtOHcl2	Galcl1	YPDnc1	YPDnc2	YPDnc3	YPDnc4	EtOHnc1	EtOHnc2	Galnc1	Galnc2
13	CLN3	YAL040C	YPDcl2	YPDcl3	EtOHcl1	EtOHcl2	Galcl1	YPDnc1	YPDnc2	YPDnc3	YPDnc4	EtOHnc1	EtOHnc2	Galnc1	Galnc2
13		YHL002C-A	YPDcl2	YPDcl3	EtOHcl1	EtOHcl2	Galcl1	YPDnc1	YPDnc2	YPDnc3	YPDnc4	EtOHnc1	EtOHnc2	Galnc1	Galnc2
13	FCP1	YMR277W	YPDcl2	YPDcl3	EtOHcl1	EtOHcl2	Galcl1	YPDnc1	YPDnc2	YPDnc3	YPDnc4	EtOHnc1	EtOHnc2	Galnc1	Galnc2
13	ZIM17	YNL310C	YPDcl2	YPDcl3	EtOHcl1	EtOHcl2	Galcl1	YPDnc1	YPDnc2	YPDnc3	YPDnc4	EtOHnc1	EtOHnc2	Galnc1	Galnc2
13	PSY3	YLR376C	YPDcl2	YPDcl3	EtOHcl1	EtOHcl2	Galcl1	YPDnc1	YPDnc2	YPDnc3	YPDnc4	EtOHnc1	EtOHnc2	Galnc1	Galnc2
13	YNG1	YOR064C	YPDcl2	YPDcl3	EtOHcl1	EtOHcl2	Galcl1	YPDnc1	YPDnc2	YPDnc3	YPDnc4	EtOHnc1	EtOHnc2	Galnc1	Galnc2
13	SOD1	YJR104C	YPDcl2	YPDcl3	EtOHcl1	EtOHcl2	Galcl1	YPDnc1	YPDnc2	YPDnc3	YPDnc4	EtOHnc1	EtOHnc2	Galnc1	Galnc2

13	SAM1	YLR180W	YPDcl2	YPDcl3	EtOHcl1	EtOHcl2	Galcl1	YPDnc1	YPDnc2	YPDnc3	YPDnc4	EtOHnc1	EtOHnc2	Galnc1	Galnc2
13	RNA15	YGL044C	YPDcl2	YPDcl3	EtOHcl1	EtOHcl2	Galcl1	YPDnc1	YPDnc2	YPDnc3	YPDnc4	EtOHnc1	EtOHnc2	Galnc1	Galnc2
13		YNL276C	YPDcl2	YPDcl3	EtOHcl1	EtOHcl2	Galcl1	YPDnc1	YPDnc2	YPDnc3	YPDnc4	EtOHnc1	EtOHnc2	Galnc1	Galnc2
13	PPH22	YDL188C	YPDcl2	YPDcl3	EtOHcl1	EtOHcl2	Galcl1	YPDnc1	YPDnc2	YPDnc3	YPDnc4	EtOHnc1	EtOHnc2	Galnc1	Galnc2
13		YHR113W	YPDcl2	YPDcl3	EtOHcl1	EtOHcl2	Galcl1	YPDnc1	YPDnc2	YPDnc3	YPDnc4	EtOHnc1	EtOHnc2	Galnc1	Galnc2
13	CDC33	YOL139C	YPDcl2	YPDcl3	EtOHcl1	EtOHcl2	Galcl1	YPDnc1	YPDnc2	YPDnc3	YPDnc4	EtOHnc1	EtOHnc2	Galnc1	Galnc2
13		YHL018W	YPDcl2	YPDcl3	EtOHcl1	EtOHcl2	Galcl1	YPDnc1	YPDnc2	YPDnc3	YPDnc4	EtOHnc1	EtOHnc2	Galnc1	Galnc2
13	IRC3	YDR332W	YPDcl2	YPDcl3	EtOHcl1	EtOHcl2	Galcl1	YPDnc1	YPDnc2	YPDnc3	YPDnc4	EtOHnc1	EtOHnc2	Galnc1	Galnc2
13	DPL1	YDR294C	YPDcl2	YPDcl3	EtOHcl1	EtOHcl2	Galcl1	YPDnc1	YPDnc2	YPDnc3	YPDnc4	EtOHnc1	EtOHnc2	Galnc1	Galnc2
13	UME6	YDR207C	YPDcl2	YPDcl3	EtOHcl1	EtOHcl2	Galcl1	YPDnc1	YPDnc2	YPDnc3	YPDnc4	EtOHnc1	EtOHnc2	Galnc1	Galnc2
13	YPI1	YFR003C	YPDcl2	YPDcl3	EtOHcl1	EtOHcl2	Galcl1	YPDnc1	YPDnc2	YPDnc3	YPDnc4	EtOHnc1	EtOHnc2	Galnc1	Galnc2
13	PRE4	YFR050C	YPDcl2	YPDcl3	EtOHcl1	EtOHcl2	Galcl1	YPDnc1	YPDnc2	YPDnc3	YPDnc4	EtOHnc1	EtOHnc2	Galnc1	Galnc2
13	ERG7	YHR072W	YPDcl2	YPDcl3	EtOHcl1	EtOHcl2	Galcl1	YPDnc1	YPDnc2	YPDnc3	YPDnc4	EtOHnc1	EtOHnc2	Galnc1	Galnc2
13	BIR1	YJR089W	YPDcl2	YPDcl3	EtOHcl1	EtOHcl2	Galcl1	YPDnc1	YPDnc2	YPDnc3	YPDnc4	EtOHnc1	EtOHnc2	Galnc1	Galnc2
13	ISY1	YJR050W	YPDcl2	YPDcl3	EtOHcl1	EtOHcl2	Galcl1	YPDnc1	YPDnc2	YPDnc3	YPDnc4	EtOHnc1	EtOHnc2	Galnc1	Galnc2
13	RIB2	YOL066C	YPDcl2	YPDcl3	EtOHcl1	EtOHcl2	Galcl1	YPDnc1	YPDnc2	YPDnc3	YPDnc4	EtOHnc1	EtOHnc2	Galnc1	Galnc2
13		YLL006W-A	YPDcl2	YPDcl3	EtOHcl1	EtOHcl2	Galcl1	YPDnc1	YPDnc2	YPDnc3	YPDnc4	EtOHnc1	EtOHnc2	Galnc1	Galnc2
13	HHF2	YNL030W	YPDcl2	YPDcl3	EtOHcl1	EtOHcl2	Galcl1	YPDnc1	YPDnc2	YPDnc3	YPDnc4	EtOHnc1	EtOHnc2	Galnc1	Galnc2
13	RPS19B	YNL302C	YPDcl2	YPDcl3	EtOHcl1	EtOHcl2	Galcl1	YPDnc1	YPDnc2	YPDnc3	YPDnc4	EtOHnc1	EtOHnc2	Galnc1	Galnc2
13	COX20	YDR231C	YPDcl2	YPDcl3	EtOHcl1	EtOHcl2	Galcl1	YPDnc1	YPDnc2	YPDnc3	YPDnc4	EtOHnc1	EtOHnc2	Galnc1	Galnc2
13		YDR374C	YPDcl2	YPDcl3	EtOHcl1	EtOHcl2	Galcl1	YPDnc1	YPDnc2	YPDnc3	YPDnc4	EtOHnc1	EtOHnc2	Galnc1	Galnc2
13	PEP12	YOR036W	YPDcl2	YPDcl3	EtOHcl1	EtOHcl2	Galcl1	YPDnc1	YPDnc2	YPDnc3	YPDnc4	EtOHnc1	EtOHnc2	Galnc1	Galnc2
13	SWF1	YDR126W	YPDcl2	YPDcl3	EtOHcl1	EtOHcl2	Galcl1	YPDnc1	YPDnc2	YPDnc3	YPDnc4	EtOHnc1	EtOHnc2	Galnc1	Galnc2
13	WRS1	YOL097C	YPDcl2	YPDcl3	EtOHcl1	EtOHcl2	Galcl1	YPDnc1	YPDnc2	YPDnc3	YPDnc4	EtOHnc1	EtOHnc2	Galnc1	Galnc2
13	NEM1	YHR004C	YPDcl2	YPDcl3	EtOHcl1	EtOHcl2	Galcl1	YPDnc1	YPDnc2	YPDnc3	YPDnc4	EtOHnc1	EtOHnc2	Galnc1	Galnc2
13	WHI3	YNL197C	YPDcl2	YPDcl3	EtOHcl1	EtOHcl2	Galcl1	YPDnc1	YPDnc2	YPDnc3	YPDnc4	EtOHnc1	EtOHnc2	Galnc1	Galnc2
13	BTT1	YDR252W	YPDcl2	YPDcl3	EtOHcl1	EtOHcl2	Galcl1	YPDnc1	YPDnc2	YPDnc3	YPDnc4	EtOHnc1	EtOHnc2	Galnc1	Galnc2
13		YDR306C	YPDcl2	YPDcl3	EtOHcl1	EtOHcl2	Galcl1	YPDnc1	YPDnc2	YPDnc3	YPDnc4	EtOHnc1	EtOHnc2	Galnc1	Galnc2
13	KRE9	YJL174W	YPDcl2	YPDcl3	EtOHcl1	EtOHcl2	Galcl1	YPDnc1	YPDnc2	YPDnc3	YPDnc4	EtOHnc1	EtOHnc2	Galnc1	Galnc2
13	ORM2	YLR350W	YPDcl2	YPDcl3	EtOHcl1	EtOHcl2	Galcl1	YPDnc1	YPDnc2	YPDnc3	YPDnc4	EtOHnc1	EtOHnc2	Galnc1	Galnc2
13	HST3	YOR025W	YPDcl2	YPDcl3	EtOHcl1	EtOHcl2	Galcl1	YPDnc1	YPDnc2	YPDnc3	YPDnc4	EtOHnc1	EtOHnc2	Galnc1	Galnc2
13	RUB1	YDR139C	YPDcl2	YPDcl3	EtOHcl1	EtOHcl2	Galcl1	YPDnc1	YPDnc2	YPDnc3	YPDnc4	EtOHnc1	EtOHnc2	Galnc1	Galnc2
13	ATF2	YGR177C	YPDcl2	YPDcl3	EtOHcl1	EtOHcl2	Galcl1	YPDnc1	YPDnc2	YPDnc3	YPDnc4	EtOHnc1	EtOHnc2	Galnc1	Galnc2
13		YER134C	YPDcl2	YPDcl3	EtOHcl1	EtOHcl2	Galcl1	YPDnc1	YPDnc2	YPDnc3	YPDnc4	EtOHnc1	EtOHnc2	Galnc1	Galnc2

13	SSK22	YCR073C	YPDcl2	YPDcl3	EtOHcl1	EtOHcl2	Galcl1	YPDnc1	YPDnc2	YPDnc3	YPDnc4	EtOHnc1	EtOHnc2	Galnc1	Galnc2
13	PBN1	YCL052C	YPDcl2	YPDcl3	EtOHcl1	EtOHcl2	Galcl1	YPDnc1	YPDnc2	YPDnc3	YPDnc4	EtOHnc1	EtOHnc2	Galnc1	Galnc2
13	RMD5	YDR255C	YPDcl2	YPDcl3	EtOHcl1	EtOHcl2	Galcl1	YPDnc1	YPDnc2	YPDnc3	YPDnc4	EtOHnc1	EtOHnc2	Galnc1	Galnc2
13		YOR379C	YPDcl2	YPDcl3	EtOHcl1	EtOHcl2	Galcl1	YPDnc1	YPDnc2	YPDnc3	YPDnc4	EtOHnc1	EtOHnc2	Galnc1	Galnc2
13	VNX1	YNL321W	YPDcl2	YPDcl3	EtOHcl1	EtOHcl2	Galcl1	YPDnc1	YPDnc2	YPDnc3	YPDnc4	EtOHnc1	EtOHnc2	Galnc1	Galnc2
13	VPS24	YKL041W	YPDcl2	YPDcl3	EtOHcl1	EtOHcl2	Galcl1	YPDnc1	YPDnc2	YPDnc3	YPDnc4	EtOHnc1	EtOHnc2	Galnc1	Galnc2
13		YNR042W	YPDcl2	YPDcl3	EtOHcl1	EtOHcl2	Galcl1	YPDnc1	YPDnc2	YPDnc3	YPDnc4	EtOHnc1	EtOHnc2	Galnc1	Galnc2
13		YER163C	YPDcl2	YPDcl3	EtOHcl1	EtOHcl2	Galcl1	YPDnc1	YPDnc2	YPDnc3	YPDnc4	EtOHnc1	EtOHnc2	Galnc1	Galnc2
13		YIL025C	YPDcl2	YPDcl3	EtOHcl1	EtOHcl2	Galcl1	YPDnc1	YPDnc2	YPDnc3	YPDnc4	EtOHnc1	EtOHnc2	Galnc1	Galnc2
13		YML009W-	YPDcl2	YPDcl3	EtOHcl1	EtOHcl2	Galcl1	YPDnc1	YPDnc2	YPDnc3	YPDnc4	EtOHnc1	EtOHnc2	Galnc1	Galnc2
13	OM14	YBR230C	YPDcl2	YPDcl3	EtOHcl1	EtOHcl2	Galcl1	YPDnc1	YPDnc2	YPDnc3	YPDnc4	EtOHnc1	EtOHnc2	Galnc1	Galnc2
13	DCN1	YLR128W	YPDcl2	YPDcl3	EtOHcl1	EtOHcl2	Galcl1	YPDnc1	YPDnc2	YPDnc3	YPDnc4	EtOHnc1	EtOHnc2	Galnc1	Galnc2
13	RAD34	YDR314C	YPDcl2	YPDcl3	EtOHcl1	EtOHcl2	Galcl1	YPDnc1	YPDnc2	YPDnc3	YPDnc4	EtOHnc1	EtOHnc2	Galnc1	Galnc2
12	SYF2	YGR129W	YPDcl2	YPDcl3	EtOHcl1	EtOHcl2	Galcl1	YPDnc1	YPDnc2	YPDnc3	YPDnc4	EtOHnc1	-	Galnc1	Galnc2
12	HAA1	YPR008W	YPDcl2	YPDcl3	EtOHcl1	-	Galcl1	YPDnc1	YPDnc2	YPDnc3	YPDnc4	EtOHnc1	EtOHnc2	Galnc1	Galnc2
12	ARF3	YOR094W	YPDcl2	YPDcl3	EtOHcl1	-	Galcl1	YPDnc1	YPDnc2	YPDnc3	YPDnc4	EtOHnc1	EtOHnc2	Galnc1	Galnc2
12	MRP51	YPL118W	YPDcl2	YPDcl3	EtOHcl1	EtOHcl2	Galcl1	-	YPDnc2	YPDnc3	YPDnc4	EtOHnc1	EtOHnc2	Galnc1	Galnc2
12		YBR271W	YPDcl2	YPDcl3	EtOHcl1	-	Galcl1	YPDnc1	YPDnc2	YPDnc3	YPDnc4	EtOHnc1	EtOHnc2	Galnc1	Galnc2
12	OCA4	YCR095C	YPDcl2	YPDcl3	EtOHcl1	EtOHcl2	Galcl1	-	YPDnc2	YPDnc3	YPDnc4	EtOHnc1	EtOHnc2	Galnc1	Galnc2
12	FMP45	YDL222C	YPDcl2	YPDcl3	EtOHcl1	EtOHcl2	Galcl1	YPDnc1	YPDnc2	YPDnc3	YPDnc4	-	EtOHnc2	Galnc1	Galnc2
12	HEM1	YDR232W	YPDcl2	YPDcl3	EtOHcl1	-	Galcl1	YPDnc1	YPDnc2	YPDnc3	YPDnc4	EtOHnc1	EtOHnc2	Galnc1	Galnc2
12	PIB1	YDR313C	YPDcl2	YPDcl3	-	EtOHcl2	Galcl1	YPDnc1	YPDnc2	YPDnc3	YPDnc4	EtOHnc1	EtOHnc2	Galnc1	Galnc2
12		YKL183C-A	YPDcl2	YPDcl3	EtOHcl1	EtOHcl2	Galcl1	YPDnc1	YPDnc2	-	YPDnc4	EtOHnc1	EtOHnc2	Galnc1	Galnc2
12	FRT1	YOR324C	YPDcl2	YPDcl3	EtOHcl1	EtOHcl2	Galcl1	YPDnc1	-	YPDnc3	YPDnc4	EtOHnc1	EtOHnc2	Galnc1	Galnc2
12		YNL134C	YPDcl2	YPDcl3	EtOHcl1	-	Galcl1	YPDnc1	YPDnc2	YPDnc3	YPDnc4	EtOHnc1	EtOHnc2	Galnc1	Galnc2
12	PCS60	YBR222C	YPDcl2	YPDcl3	EtOHcl1	EtOHcl2	Galcl1	YPDnc1	YPDnc2	YPDnc3	YPDnc4	EtOHnc1	-	Galnc1	Galnc2
12	DYN1	YKR054C	YPDcl2	YPDcl3	EtOHcl1	EtOHcl2	Galcl1	YPDnc1	YPDnc2	YPDnc3	YPDnc4	EtOHnc1	-	Galnc1	Galnc2
12		YKL187C	YPDcl2	YPDcl3	EtOHcl1	-	Galcl1	YPDnc1	YPDnc2	YPDnc3	YPDnc4	EtOHnc1	EtOHnc2	Galnc1	Galnc2
12	NKP1	YDR383C	-	YPDcl3	EtOHcl1	EtOHcl2	Galcl1	YPDnc1	YPDnc2	YPDnc3	YPDnc4	EtOHnc1	EtOHnc2	Galnc1	Galnc2
12	SIP3	YNL257C	YPDcl2	YPDcl3	EtOHcl1	-	Galcl1	YPDnc1	YPDnc2	YPDnc3	YPDnc4	EtOHnc1	EtOHnc2	Galnc1	Galnc2
12		YDR262W	YPDcl2	YPDcl3	EtOHcl1	-	Galcl1	YPDnc1	YPDnc2	YPDnc3	YPDnc4	EtOHnc1	EtOHnc2	Galnc1	Galnc2
12	CSN9	YDR179C	YPDcl2	YPDcl3	EtOHcl1	EtOHcl2	Galcl1	YPDnc1	YPDnc2	YPDnc3	YPDnc4	EtOHnc1	EtOHnc2	Galnc1	-
12	HSP78	YDR258C	YPDcl2	YPDcl3	EtOHcl1	EtOHcl2	Galcl1	YPDnc1	YPDnc2	YPDnc3	YPDnc4	EtOHnc1	-	Galnc1	Galnc2
12	MDM34	YGL219C	YPDcl2	YPDcl3	EtOHcl1	EtOHcl2	Galcl1	YPDnc1	YPDnc2	YPDnc3	-	EtOHnc1	EtOHnc2	Galnc1	Galnc2

12	TFS1	YLR178C	-	YPDcl3	EtOHcl1	EtOHcl2	Galcl1	YPDnc1	YPDnc2	YPDnc3	YPDnc4	EtOHnc1	EtOHnc2	Galnc1	Galnc2
12	SER1	YOR184W	YPDcl2	YPDcl3	EtOHcl1	-	Galcl1	YPDnc1	YPDnc2	YPDnc3	YPDnc4	EtOHnc1	EtOHnc2	Galnc1	Galnc2
12	COQ10	YOL008W	YPDcl2	-	EtOHcl1	EtOHcl2	Galcl1	YPDnc1	YPDnc2	YPDnc3	YPDnc4	EtOHnc1	EtOHnc2	Galnc1	Galnc2
12		YGL015C	YPDcl2	YPDcl3	EtOHcl1	EtOHcl2	Galcl1	-	YPDnc2	YPDnc3	YPDnc4	EtOHnc1	EtOHnc2	Galnc1	Galnc2
12	PEP3	YLR148W	YPDcl2	YPDcl3	EtOHcl1	-	Galcl1	YPDnc1	YPDnc2	YPDnc3	YPDnc4	EtOHnc1	EtOHnc2	Galnc1	Galnc2
12		YPR097W	YPDcl2	YPDcl3	-	EtOHcl2	Galcl1	YPDnc1	YPDnc2	YPDnc3	YPDnc4	EtOHnc1	EtOHnc2	Galnc1	Galnc2
12		YDR379C-A	YPDcl2	YPDcl3	EtOHcl1	-	Galcl1	YPDnc1	YPDnc2	YPDnc3	YPDnc4	EtOHnc1	EtOHnc2	Galnc1	Galnc2
12		YOL159C	YPDcl2	YPDcl3	EtOHcl1	-	Galcl1	YPDnc1	YPDnc2	YPDnc3	YPDnc4	EtOHnc1	EtOHnc2	Galnc1	Galnc2
12	DTD1	YDL219W	YPDcl2	YPDcl3	-	EtOHcl2	Galcl1	YPDnc1	YPDnc2	YPDnc3	YPDnc4	EtOHnc1	EtOHnc2	Galnc1	Galnc2
12	ALD6	YPL061W	YPDcl2	YPDcl3	EtOHcl1	EtOHcl2	Galcl1	YPDnc1	YPDnc2	YPDnc3	YPDnc4	-	EtOHnc2	Galnc1	Galnc2
12	BNA3	YJL060W	YPDcl2	YPDcl3	EtOHcl1	-	Galcl1	YPDnc1	YPDnc2	YPDnc3	YPDnc4	EtOHnc1	EtOHnc2	Galnc1	Galnc2
12	GRX4	YER174C	-	YPDcl3	EtOHcl1	EtOHcl2	Galcl1	YPDnc1	YPDnc2	YPDnc3	YPDnc4	EtOHnc1	EtOHnc2	Galnc1	Galnc2
12	CRF1	YDR223W	YPDcl2	YPDcl3	EtOHcl1	-	Galcl1	YPDnc1	YPDnc2	YPDnc3	YPDnc4	EtOHnc1	EtOHnc2	Galnc1	Galnc2
12	TIF34	YMR146C	YPDcl2	YPDcl3	EtOHcl1	EtOHcl2	Galcl1	YPDnc1	YPDnc2	YPDnc3	YPDnc4	EtOHnc1	EtOHnc2	Galnc1	-
12	PYC1	YGL062W	YPDcl2	YPDcl3	-	EtOHcl2	Galcl1	YPDnc1	YPDnc2	YPDnc3	YPDnc4	EtOHnc1	EtOHnc2	Galnc1	Galnc2
12		YIL012W	YPDcl2	-	EtOHcl1	EtOHcl2	Galcl1	YPDnc1	YPDnc2	YPDnc3	YPDnc4	EtOHnc1	EtOHnc2	Galnc1	Galnc2
12	RSM7	YJR113C	YPDcl2	YPDcl3	EtOHcl1	EtOHcl2	-	YPDnc1	YPDnc2	YPDnc3	YPDnc4	EtOHnc1	EtOHnc2	Galnc1	Galnc2
12		YOL106W	-	YPDcl3	EtOHcl1	EtOHcl2	Galcl1	YPDnc1	YPDnc2	YPDnc3	YPDnc4	EtOHnc1	EtOHnc2	Galnc1	Galnc2
12		YPR077C	YPDcl2	YPDcl3	EtOHcl1	-	Galcl1	YPDnc1	YPDnc2	YPDnc3	YPDnc4	EtOHnc1	EtOHnc2	Galnc1	Galnc2
12		YOR365C	YPDcl2	YPDcl3	EtOHcl1	EtOHcl2	Galcl1	-	YPDnc2	YPDnc3	YPDnc4	EtOHnc1	EtOHnc2	Galnc1	Galnc2
12	DCS2	YOR173W	YPDcl2	YPDcl3	EtOHcl1	EtOHcl2	Galcl1	YPDnc1	YPDnc2	YPDnc3	YPDnc4	EtOHnc1	-	Galnc1	Galnc2
12	TEA1	YOR337W	-	YPDcl3	EtOHcl1	EtOHcl2	Galcl1	YPDnc1	YPDnc2	YPDnc3	YPDnc4	EtOHnc1	EtOHnc2	Galnc1	Galnc2
12	RLP7	YNL002C	YPDcl2	YPDcl3	EtOHcl1	-	Galcl1	YPDnc1	YPDnc2	YPDnc3	YPDnc4	EtOHnc1	EtOHnc2	Galnc1	Galnc2
12	OSW2	YLR054C	YPDcl2	-	EtOHcl1	EtOHcl2	Galcl1	YPDnc1	YPDnc2	YPDnc3	YPDnc4	EtOHnc1	EtOHnc2	Galnc1	Galnc2
12	DNL4	YOR005C	YPDcl2	YPDcl3	EtOHcl1	EtOHcl2	Galcl1	YPDnc1	YPDnc2	YPDnc3	YPDnc4	EtOHnc1	EtOHnc2	Galnc1	-
12		YLR458W	YPDcl2	YPDcl3	EtOHcl1	-	Galcl1	YPDnc1	YPDnc2	YPDnc3	YPDnc4	EtOHnc1	EtOHnc2	Galnc1	Galnc2
12	ERD2	YBL040C	-	YPDcl3	EtOHcl1	EtOHcl2	Galcl1	YPDnc1	YPDnc2	YPDnc3	YPDnc4	EtOHnc1	EtOHnc2	Galnc1	Galnc2
12	RSF2	YJR127C	YPDcl2	YPDcl3	EtOHcl1	EtOHcl2	Galcl1	YPDnc1	-	YPDnc3	YPDnc4	EtOHnc1	EtOHnc2	Galnc1	Galnc2
12	RIF2	YLR453C	YPDcl2	YPDcl3	EtOHcl1	EtOHcl2	Galcl1	YPDnc1	YPDnc2	YPDnc3	YPDnc4	EtOHnc1	-	Galnc1	Galnc2
12		YCR007C	-	YPDcl3	EtOHcl1	EtOHcl2	Galcl1	YPDnc1	YPDnc2	YPDnc3	YPDnc4	EtOHnc1	EtOHnc2	Galnc1	Galnc2
12	TIM18	YOR297C	YPDcl2	YPDcl3	-	EtOHcl2	Galcl1	YPDnc1	YPDnc2	YPDnc3	YPDnc4	EtOHnc1	EtOHnc2	Galnc1	Galnc2
12	ATP20	YPR020W	YPDcl2	YPDcl3	EtOHcl1	-	Galcl1	YPDnc1	YPDnc2	YPDnc3	YPDnc4	EtOHnc1	EtOHnc2	Galnc1	Galnc2
12	UBI4	YLL039C	YPDcl2	YPDcl3	EtOHcl1	EtOHcl2	Galcl1	YPDnc1	-	YPDnc3	YPDnc4	EtOHnc1	EtOHnc2	Galnc1	Galnc2
12	HSC82	YMR186W	YPDcl2	YPDcl3	EtOHcl1	-	Galcl1	YPDnc1	YPDnc2	YPDnc3	YPDnc4	EtOHnc1	EtOHnc2	Galnc1	Galnc2

12		YHL042W	YPDcl2	YPDcl3	EtOHcl1	-	Galcl1	YPDnc1	YPDnc2	YPDnc3	YPDnc4	EtOHnc1	EtOHnc2	Galnc1	Galnc2
12		YOR011W-	YPDcl2	YPDcl3	EtOHcl1	EtOHcl2	Galcl1	YPDnc1	YPDnc2	YPDnc3	YPDnc4	-	EtOHnc2	Galnc1	Galnc2
12	TIF1	YKR059W	YPDcl2	YPDcl3	EtOHcl1	EtOHcl2	Galcl1	-	YPDnc2	YPDnc3	YPDnc4	EtOHnc1	EtOHnc2	Galnc1	Galnc2
12	BAP3	YDR046C	YPDcl2	-	EtOHcl1	EtOHcl2	Galcl1	YPDnc1	YPDnc2	YPDnc3	YPDnc4	EtOHnc1	EtOHnc2	Galnc1	Galnc2
12		YML018C	YPDcl2	YPDcl3	-	EtOHcl2	Galcl1	YPDnc1	YPDnc2	YPDnc3	YPDnc4	EtOHnc1	EtOHnc2	Galnc1	Galnc2
12	RPS4A	YJR145C	-	YPDcl3	EtOHcl1	EtOHcl2	Galcl1	YPDnc1	YPDnc2	YPDnc3	YPDnc4	EtOHnc1	EtOHnc2	Galnc1	Galnc2
12	NHA1	YLR138W	-	YPDcl3	EtOHcl1	EtOHcl2	Galcl1	YPDnc1	YPDnc2	YPDnc3	YPDnc4	EtOHnc1	EtOHnc2	Galnc1	Galnc2
12	RPL14A	YKL006W	YPDcl2	-	EtOHcl1	EtOHcl2	Galcl1	YPDnc1	YPDnc2	YPDnc3	YPDnc4	EtOHnc1	EtOHnc2	Galnc1	Galnc2
12	ADA2	YDR448W	YPDcl2	YPDcl3	EtOHcl1	EtOHcl2	Galcl1	YPDnc1	YPDnc2	YPDnc3	YPDnc4	EtOHnc1	-	Galnc1	Galnc2
12	MDM30	YLR368W	YPDcl2	YPDcl3	EtOHcl1	-	Galcl1	YPDnc1	YPDnc2	YPDnc3	YPDnc4	EtOHnc1	EtOHnc2	Galnc1	Galnc2
12	ERG25	YGR060W	YPDcl2	YPDcl3	EtOHcl1	EtOHcl2	-	YPDnc1	YPDnc2	YPDnc3	YPDnc4	EtOHnc1	EtOHnc2	Galnc1	Galnc2
12		YML079W	-	YPDcl3	EtOHcl1	EtOHcl2	Galcl1	YPDnc1	YPDnc2	YPDnc3	YPDnc4	EtOHnc1	EtOHnc2	Galnc1	Galnc2
12		YPL152W-A	YPDcl2	YPDcl3	EtOHcl1	EtOHcl2	-	YPDnc1	YPDnc2	YPDnc3	YPDnc4	EtOHnc1	EtOHnc2	Galnc1	Galnc2
12	RHB1	YCR027C	YPDcl2	YPDcl3	EtOHcl1	-	Galcl1	YPDnc1	YPDnc2	YPDnc3	YPDnc4	EtOHnc1	EtOHnc2	Galnc1	Galnc2
12	RSP5	YER125W	YPDcl2	YPDcl3	EtOHcl1	-	Galcl1	YPDnc1	YPDnc2	YPDnc3	YPDnc4	EtOHnc1	EtOHnc2	Galnc1	Galnc2
12	NUP42	YDR192C	YPDcl2	-	EtOHcl1	EtOHcl2	Galcl1	YPDnc1	YPDnc2	YPDnc3	YPDnc4	EtOHnc1	EtOHnc2	Galnc1	Galnc2
12	HOP2	YGL033W	YPDcl2	YPDcl3	EtOHcl1	EtOHcl2	-	YPDnc1	YPDnc2	YPDnc3	YPDnc4	EtOHnc1	EtOHnc2	Galnc1	Galnc2
12	DML1	YMR211W	YPDcl2	YPDcl3	EtOHcl1	-	Galcl1	YPDnc1	YPDnc2	YPDnc3	YPDnc4	EtOHnc1	EtOHnc2	Galnc1	Galnc2
12	VHT1	YGR065C	YPDcl2	-	EtOHcl1	EtOHcl2	Galcl1	YPDnc1	YPDnc2	YPDnc3	YPDnc4	EtOHnc1	EtOHnc2	Galnc1	Galnc2
12	GCD1	YOR260W	YPDcl2	YPDcl3	EtOHcl1	EtOHcl2	Galcl1	-	YPDnc2	YPDnc3	YPDnc4	EtOHnc1	EtOHnc2	Galnc1	Galnc2
12	SPO13	YHR014W	YPDcl2	YPDcl3	EtOHcl1	-	Galcl1	YPDnc1	YPDnc2	YPDnc3	YPDnc4	EtOHnc1	EtOHnc2	Galnc1	Galnc2
12	MEF2	YJL102W	-	YPDcl3	EtOHcl1	EtOHcl2	Galcl1	YPDnc1	YPDnc2	YPDnc3	YPDnc4	EtOHnc1	EtOHnc2	Galnc1	Galnc2
12	TSC13	YDL015C	YPDcl2	-	EtOHcl1	EtOHcl2	Galcl1	YPDnc1	YPDnc2	YPDnc3	YPDnc4	EtOHnc1	EtOHnc2	Galnc1	Galnc2
12	HSP31	YDR533C	YPDcl2	YPDcl3	EtOHcl1	EtOHcl2	Galcl1	YPDnc1	YPDnc2	YPDnc3	YPDnc4	EtOHnc1	-	Galnc1	Galnc2
12	SWC7	YLR385C	YPDcl2	YPDcl3	EtOHcl1	-	Galcl1	YPDnc1	YPDnc2	YPDnc3	YPDnc4	EtOHnc1	EtOHnc2	Galnc1	Galnc2
12	GTO1	YGR154C	YPDcl2	-	EtOHcl1	EtOHcl2	Galcl1	YPDnc1	YPDnc2	YPDnc3	YPDnc4	EtOHnc1	EtOHnc2	Galnc1	Galnc2
12	MET2	YNL277W	YPDcl2	-	EtOHcl1	EtOHcl2	Galcl1	YPDnc1	YPDnc2	YPDnc3	YPDnc4	EtOHnc1	EtOHnc2	Galnc1	Galnc2
12	PRS5	YOL061W	YPDcl2	YPDcl3	-	EtOHcl2	Galcl1	YPDnc1	YPDnc2	YPDnc3	YPDnc4	EtOHnc1	EtOHnc2	Galnc1	Galnc2
12	HCR1	YLR192C	YPDcl2	YPDcl3	EtOHcl1	EtOHcl2	Galcl1	YPDnc1	YPDnc2	YPDnc3	YPDnc4	-	EtOHnc2	Galnc1	Galnc2
12	RBL2	YOR265W	YPDcl2	YPDcl3	EtOHcl1	-	Galcl1	YPDnc1	YPDnc2	YPDnc3	YPDnc4	EtOHnc1	EtOHnc2	Galnc1	Galnc2
12		YOR333C	YPDcl2	YPDcl3	EtOHcl1	-	Galcl1	YPDnc1	YPDnc2	YPDnc3	YPDnc4	EtOHnc1	EtOHnc2	Galnc1	Galnc2
12	PKH1	YDR490C	YPDcl2	YPDcl3	EtOHcl1	EtOHcl2	Galcl1	YPDnc1	YPDnc2	YPDnc3	YPDnc4	EtOHnc1	EtOHnc2	Galnc1	-
12	VPS38	YLR360W	YPDcl2	YPDcl3	EtOHcl1	EtOHcl2	Galcl1	YPDnc1	YPDnc2	-	YPDnc4	EtOHnc1	EtOHnc2	Galnc1	Galnc2
12	LSC1	YOR142W	YPDcl2	YPDcl3	EtOHcl1	-	Galcl1	YPDnc1	YPDnc2	YPDnc3	YPDnc4	EtOHnc1	EtOHnc2	Galnc1	Galnc2

12	CPR5	YDR304C	YPDcl2	YPDcl3	EtOHcl1	EtOHcl2	Galcl1	YPDnc1	YPDnc2	-	YPDnc4	EtOHnc1	EtOHnc2	Galnc1	Galnc2
12	PUS5	YLR165C	YPDcl2	YPDcl3	EtOHcl1	-	Galcl1	YPDnc1	YPDnc2	YPDnc3	YPDnc4	EtOHnc1	EtOHnc2	Galnc1	Galnc2
12	ERM6	YJR054W	YPDcl2	YPDcl3	EtOHcl1	EtOHcl2	Galcl1	YPDnc1	YPDnc2	YPDnc3	YPDnc4	-	EtOHnc2	Galnc1	Galnc2
12	PEP4	YPL154C	-	YPDcl3	EtOHcl1	EtOHcl2	Galcl1	YPDnc1	YPDnc2	YPDnc3	YPDnc4	EtOHnc1	EtOHnc2	Galnc1	Galnc2
12	SIC1	YLR079W	YPDcl2	YPDcl3	EtOHcl1	EtOHcl2	Galcl1	YPDnc1	YPDnc2	YPDnc3	-	EtOHnc1	EtOHnc2	Galnc1	Galnc2
12	RPO21	YDL140C	-	YPDcl3	EtOHcl1	EtOHcl2	Galcl1	YPDnc1	YPDnc2	YPDnc3	YPDnc4	EtOHnc1	EtOHnc2	Galnc1	Galnc2
12	PPA1	YHR026W	YPDcl2	YPDcl3	EtOHcl1	EtOHcl2	Galcl1	YPDnc1	-	YPDnc3	YPDnc4	EtOHnc1	EtOHnc2	Galnc1	Galnc2
12	PRR1	YKL116C	YPDcl2	-	EtOHcl1	EtOHcl2	Galcl1	YPDnc1	YPDnc2	YPDnc3	YPDnc4	EtOHnc1	EtOHnc2	Galnc1	Galnc2
12	ECM30	YLR436C	YPDcl2	YPDcl3	EtOHcl1	EtOHcl2	Galcl1	YPDnc1	YPDnc2	YPDnc3	YPDnc4	EtOHnc1	EtOHnc2	-	Galnc2
12		YPR160C-A	YPDcl2	YPDcl3	EtOHcl1	-	Galcl1	YPDnc1	YPDnc2	YPDnc3	YPDnc4	EtOHnc1	EtOHnc2	Galnc1	Galnc2
12	UBC8	YEL012W	YPDcl2	YPDcl3	EtOHcl1	EtOHcl2	Galcl1	YPDnc1	YPDnc2	YPDnc3	YPDnc4	EtOHnc1	-	Galnc1	Galnc2
12	THI72	YOR192C	-	YPDcl3	EtOHcl1	EtOHcl2	Galcl1	YPDnc1	YPDnc2	YPDnc3	YPDnc4	EtOHnc1	EtOHnc2	Galnc1	Galnc2
12	OPT2	YPR194C	YPDcl2	YPDcl3	EtOHcl1	-	Galcl1	YPDnc1	YPDnc2	YPDnc3	YPDnc4	EtOHnc1	EtOHnc2	Galnc1	Galnc2
12	PAU13	YHL046C	YPDcl2	YPDcl3	EtOHcl1	-	Galcl1	YPDnc1	YPDnc2	YPDnc3	YPDnc4	EtOHnc1	EtOHnc2	Galnc1	Galnc2
12	LAS21	YJL062W	YPDcl2	YPDcl3	EtOHcl1	EtOHcl2	Galcl1	-	YPDnc2	YPDnc3	YPDnc4	EtOHnc1	EtOHnc2	Galnc1	Galnc2
12	RPS0B	YLR048W	-	YPDcl3	EtOHcl1	EtOHcl2	Galcl1	YPDnc1	YPDnc2	YPDnc3	YPDnc4	EtOHnc1	EtOHnc2	Galnc1	Galnc2
12	NTC20	YBR188C	YPDcl2	YPDcl3	EtOHcl1	EtOHcl2	Galcl1	-	YPDnc2	YPDnc3	YPDnc4	EtOHnc1	EtOHnc2	Galnc1	Galnc2
12	EUG1	YDR518W	YPDcl2	YPDcl3	EtOHcl1	EtOHcl2	Galcl1	YPDnc1	YPDnc2	YPDnc3	YPDnc4	-	EtOHnc2	Galnc1	Galnc2
12		YJL120W	YPDcl2	YPDcl3	EtOHcl1	-	Galcl1	YPDnc1	YPDnc2	YPDnc3	YPDnc4	EtOHnc1	EtOHnc2	Galnc1	Galnc2
12	RAD6	YGL058W	YPDcl2	YPDcl3	EtOHcl1	EtOHcl2	-	YPDnc1	YPDnc2	YPDnc3	YPDnc4	EtOHnc1	EtOHnc2	Galnc1	Galnc2
12	SEC4	YFL005W	YPDcl2	-	EtOHcl1	EtOHcl2	Galcl1	YPDnc1	YPDnc2	YPDnc3	YPDnc4	EtOHnc1	EtOHnc2	Galnc1	Galnc2
12	YUR1	YJL139C	YPDcl2	-	EtOHcl1	EtOHcl2	Galcl1	YPDnc1	YPDnc2	YPDnc3	YPDnc4	EtOHnc1	EtOHnc2	Galnc1	Galnc2
12		YDL129W	-	YPDcl3	EtOHcl1	EtOHcl2	Galcl1	YPDnc1	YPDnc2	YPDnc3	YPDnc4	EtOHnc1	EtOHnc2	Galnc1	Galnc2
12	POG1	YIL122W	YPDcl2	YPDcl3	EtOHcl1	-	Galcl1	YPDnc1	YPDnc2	YPDnc3	YPDnc4	EtOHnc1	EtOHnc2	Galnc1	Galnc2
12	ERV2	YPR037C	YPDcl2	-	EtOHcl1	EtOHcl2	Galcl1	YPDnc1	YPDnc2	YPDnc3	YPDnc4	EtOHnc1	EtOHnc2	Galnc1	Galnc2
12	EMP46	YLR080W	YPDcl2	YPDcl3	EtOHcl1	EtOHcl2	-	YPDnc1	YPDnc2	YPDnc3	YPDnc4	EtOHnc1	EtOHnc2	Galnc1	Galnc2
12	SGE1	YPR198W	YPDcl2	YPDcl3	EtOHcl1	EtOHcl2	Galcl1	YPDnc1	-	YPDnc3	YPDnc4	EtOHnc1	EtOHnc2	Galnc1	Galnc2
12	PRE5	YMR314W	YPDcl2	YPDcl3	EtOHcl1	EtOHcl2	Galcl1	YPDnc1	-	YPDnc3	YPDnc4	EtOHnc1	EtOHnc2	Galnc1	Galnc2
12		YNL247W	YPDcl2	YPDcl3	EtOHcl1	-	Galcl1	YPDnc1	YPDnc2	YPDnc3	YPDnc4	EtOHnc1	EtOHnc2	Galnc1	Galnc2
12	PRP40	YKL012W	YPDcl2	YPDcl3	EtOHcl1	EtOHcl2	Galcl1	YPDnc1	YPDnc2	YPDnc3	YPDnc4	EtOHnc1	EtOHnc2	Galnc1	-
12		YOR020W-.	YPDcl2	-	EtOHcl1	EtOHcl2	Galcl1	YPDnc1	YPDnc2	YPDnc3	YPDnc4	EtOHnc1	EtOHnc2	Galnc1	Galnc2
12	SWA2	YDR320C	YPDcl2	YPDcl3	-	EtOHcl2	Galcl1	YPDnc1	YPDnc2	YPDnc3	YPDnc4	EtOHnc1	EtOHnc2	Galnc1	Galnc2
12	TPD3	YAL016W	-	YPDcl3	EtOHcl1	EtOHcl2	Galcl1	YPDnc1	YPDnc2	YPDnc3	YPDnc4	EtOHnc1	EtOHnc2	Galnc1	Galnc2
12	ICT1	YLR099C	YPDcl2	YPDcl3	EtOHcl1	EtOHcl2	-	YPDnc1	YPDnc2	YPDnc3	YPDnc4	EtOHnc1	EtOHnc2	Galnc1	Galnc2

12		YNL277W-/	YPDcl2	YPDcl3	EtOHcl1	EtOHcl2	Galcl1	YPDnc1	YPDnc2	-	YPDnc4	EtOHnc1	EtOHnc2	Galnc1	Galnc2
12	SPC2	YML055W	YPDcl2	YPDcl3	EtOHcl1	-	Galcl1	YPDnc1	YPDnc2	YPDnc3	YPDnc4	EtOHnc1	EtOHnc2	Galnc1	Galnc2
12		YFL052W	YPDcl2	YPDcl3	EtOHcl1	-	Galcl1	YPDnc1	YPDnc2	YPDnc3	YPDnc4	EtOHnc1	EtOHnc2	Galnc1	Galnc2
12	THI21	YPL258C	YPDcl2	YPDcl3	EtOHcl1	-	Galcl1	YPDnc1	YPDnc2	YPDnc3	YPDnc4	EtOHnc1	EtOHnc2	Galnc1	Galnc2
12	CDA1	YLR307W	YPDcl2	YPDcl3	EtOHcl1	-	Galcl1	YPDnc1	YPDnc2	YPDnc3	YPDnc4	EtOHnc1	EtOHnc2	Galnc1	Galnc2
12		YHR003C	YPDcl2	YPDcl3	EtOHcl1	EtOHcl2	Galcl1	YPDnc1	YPDnc2	YPDnc3	YPDnc4	EtOHnc1	EtOHnc2	Galnc1	-
12		YPL025C	YPDcl2	YPDcl3	EtOHcl1	-	Galcl1	YPDnc1	YPDnc2	YPDnc3	YPDnc4	EtOHnc1	EtOHnc2	Galnc1	Galnc2
12	SLX8	YER116C	YPDcl2	YPDcl3	EtOHcl1	EtOHcl2	Galcl1	YPDnc1	YPDnc2	YPDnc3	YPDnc4	-	EtOHnc2	Galnc1	Galnc2
12	HIS3	YOR202W	YPDcl2	YPDcl3	EtOHcl1	EtOHcl2	Galcl1	YPDnc1	YPDnc2	YPDnc3	YPDnc4	EtOHnc1	-	Galnc1	Galnc2
12		YGR137W	-	YPDcl3	EtOHcl1	EtOHcl2	Galcl1	YPDnc1	YPDnc2	YPDnc3	YPDnc4	EtOHnc1	EtOHnc2	Galnc1	Galnc2
12	PUF2	YPR042C	YPDcl2	YPDcl3	EtOHcl1	EtOHcl2	Galcl1	YPDnc1	YPDnc2	YPDnc3	YPDnc4	EtOHnc1	EtOHnc2	Galnc1	-
12	AAC3	YBR085W	YPDcl2	YPDcl3	EtOHcl1	-	Galcl1	YPDnc1	YPDnc2	YPDnc3	YPDnc4	EtOHnc1	EtOHnc2	Galnc1	Galnc2
12		YDR053W	YPDcl2	YPDcl3	-	EtOHcl2	Galcl1	YPDnc1	YPDnc2	YPDnc3	YPDnc4	EtOHnc1	EtOHnc2	Galnc1	Galnc2
12		YPL264C	-	YPDcl3	EtOHcl1	EtOHcl2	Galcl1	YPDnc1	YPDnc2	YPDnc3	YPDnc4	EtOHnc1	EtOHnc2	Galnc1	Galnc2
12	FIT2	YOR382W	YPDcl2	YPDcl3	EtOHcl1	-	Galcl1	YPDnc1	YPDnc2	YPDnc3	YPDnc4	EtOHnc1	EtOHnc2	Galnc1	Galnc2
12	PKP2	YGL059W	YPDcl2	YPDcl3	EtOHcl1	EtOHcl2	Galcl1	YPDnc1	YPDnc2	YPDnc3	YPDnc4	EtOHnc1	EtOHnc2	-	Galnc2
12	PRI2	YKL045W	YPDcl2	YPDcl3	EtOHcl1	EtOHcl2	Galcl1	YPDnc1	YPDnc2	YPDnc3	YPDnc4	EtOHnc1	-	Galnc1	Galnc2
12	MEU1	YLR017W	YPDcl2	YPDcl3	EtOHcl1	-	Galcl1	YPDnc1	YPDnc2	YPDnc3	YPDnc4	EtOHnc1	EtOHnc2	Galnc1	Galnc2
12		YNL165W	YPDcl2	-	EtOHcl1	EtOHcl2	Galcl1	YPDnc1	YPDnc2	YPDnc3	YPDnc4	EtOHnc1	EtOHnc2	Galnc1	Galnc2
12	FET4	YMR319C	YPDcl2	YPDcl3	EtOHcl1	-	Galcl1	YPDnc1	YPDnc2	YPDnc3	YPDnc4	EtOHnc1	EtOHnc2	Galnc1	Galnc2
12		YOR152C	YPDcl2	YPDcl3	EtOHcl1	EtOHcl2	Galcl1	YPDnc1	YPDnc2	YPDnc3	YPDnc4	EtOHnc1	-	Galnc1	Galnc2
12	BAP2	YBR068C	-	YPDcl3	EtOHcl1	EtOHcl2	Galcl1	YPDnc1	YPDnc2	YPDnc3	YPDnc4	EtOHnc1	EtOHnc2	Galnc1	Galnc2
12		YER138W-/	YPDcl2	YPDcl3	EtOHcl1	-	Galcl1	YPDnc1	YPDnc2	YPDnc3	YPDnc4	EtOHnc1	EtOHnc2	Galnc1	Galnc2
12	FRE3	YOR381W	YPDcl2	YPDcl3	EtOHcl1	-	Galcl1	YPDnc1	YPDnc2	YPDnc3	YPDnc4	EtOHnc1	EtOHnc2	Galnc1	Galnc2
12	MNE1	YOR350C	YPDcl2	-	EtOHcl1	EtOHcl2	Galcl1	YPDnc1	YPDnc2	YPDnc3	YPDnc4	EtOHnc1	EtOHnc2	Galnc1	Galnc2
12	SFP1	YLR403W	YPDcl2	YPDcl3	EtOHcl1	-	Galcl1	YPDnc1	YPDnc2	YPDnc3	YPDnc4	EtOHnc1	EtOHnc2	Galnc1	Galnc2
12	KGD2	YDR148C	YPDcl2	YPDcl3	EtOHcl1	-	Galcl1	YPDnc1	YPDnc2	YPDnc3	YPDnc4	EtOHnc1	EtOHnc2	Galnc1	Galnc2
12		YER067C-A	YPDcl2	-	EtOHcl1	EtOHcl2	Galcl1	YPDnc1	YPDnc2	YPDnc3	YPDnc4	EtOHnc1	EtOHnc2	Galnc1	Galnc2
12	ERG2	YMR202W	-	YPDcl3	EtOHcl1	EtOHcl2	Galcl1	YPDnc1	YPDnc2	YPDnc3	YPDnc4	EtOHnc1	EtOHnc2	Galnc1	Galnc2
12	SFL1	YOR140W	YPDcl2	YPDcl3	-	EtOHcl2	Galcl1	YPDnc1	YPDnc2	YPDnc3	YPDnc4	EtOHnc1	EtOHnc2	Galnc1	Galnc2
12	SSP120	YLR250W	YPDcl2	YPDcl3	EtOHcl1	EtOHcl2	Galcl1	YPDnc1	YPDnc2	-	YPDnc4	EtOHnc1	EtOHnc2	Galnc1	Galnc2
12	SUV3	YPL029W	-	YPDcl3	EtOHcl1	EtOHcl2	Galcl1	YPDnc1	YPDnc2	YPDnc3	YPDnc4	EtOHnc1	EtOHnc2	Galnc1	Galnc2
12		YOL013W-/	YPDcl2	YPDcl3	EtOHcl1	-	Galcl1	YPDnc1	YPDnc2	YPDnc3	YPDnc4	EtOHnc1	EtOHnc2	Galnc1	Galnc2
12	RSM24	YDR175C	YPDcl2	YPDcl3	EtOHcl1	-	Galcl1	YPDnc1	YPDnc2	YPDnc3	YPDnc4	EtOHnc1	EtOHnc2	Galnc1	Galnc2

12	GPI11	YDR302W	YPDcl2	YPDcl3	EtOHcl1	-	Galcl1	YPDnc1	YPDnc2	YPDnc3	YPDnc4	EtOHnc1	EtOHnc2	Galnc1	Galnc2
12	VPS75	YNL246W	-	YPDcl3	EtOHcl1	EtOHcl2	Galcl1	YPDnc1	YPDnc2	YPDnc3	YPDnc4	EtOHnc1	EtOHnc2	Galnc1	Galnc2
12		YOR231C-A	YPDcl2	YPDcl3	-	EtOHcl2	Galcl1	YPDnc1	YPDnc2	YPDnc3	YPDnc4	EtOHnc1	EtOHnc2	Galnc1	Galnc2
12	AFT2	YPL202C	-	YPDcl3	EtOHcl1	EtOHcl2	Galcl1	YPDnc1	YPDnc2	YPDnc3	YPDnc4	EtOHnc1	EtOHnc2	Galnc1	Galnc2
12	GWT1	YJL091C	YPDcl2	YPDcl3	EtOHcl1	-	Galcl1	YPDnc1	YPDnc2	YPDnc3	YPDnc4	EtOHnc1	EtOHnc2	Galnc1	Galnc2
12		YLR283W	YPDcl2	YPDcl3	EtOHcl1	-	Galcl1	YPDnc1	YPDnc2	YPDnc3	YPDnc4	EtOHnc1	EtOHnc2	Galnc1	Galnc2
12	PMT6	YGR199W	YPDcl2	-	EtOHcl1	EtOHcl2	Galcl1	YPDnc1	YPDnc2	YPDnc3	YPDnc4	EtOHnc1	EtOHnc2	Galnc1	Galnc2
12		YML122C	YPDcl2	-	EtOHcl1	EtOHcl2	Galcl1	YPDnc1	YPDnc2	YPDnc3	YPDnc4	EtOHnc1	EtOHnc2	Galnc1	Galnc2
12	PCL7	YIL050W	YPDcl2	YPDcl3	EtOHcl1	-	Galcl1	YPDnc1	YPDnc2	YPDnc3	YPDnc4	EtOHnc1	EtOHnc2	Galnc1	Galnc2
12	NRM1	YNR009W	YPDcl2	YPDcl3	EtOHcl1	-	Galcl1	YPDnc1	YPDnc2	YPDnc3	YPDnc4	EtOHnc1	EtOHnc2	Galnc1	Galnc2
12	HOS2	YGL194C	YPDcl2	YPDcl3	EtOHcl1	EtOHcl2	Galcl1	YPDnc1	YPDnc2	YPDnc3	YPDnc4	EtOHnc1	EtOHnc2	Galnc1	-
12	VAM3	YOR106W	YPDcl2	YPDcl3	EtOHcl1	-	Galcl1	YPDnc1	YPDnc2	YPDnc3	YPDnc4	EtOHnc1	EtOHnc2	Galnc1	Galnc2
12	HEM13	YDR044W	YPDcl2	-	EtOHcl1	EtOHcl2	Galcl1	YPDnc1	YPDnc2	YPDnc3	YPDnc4	EtOHnc1	EtOHnc2	Galnc1	Galnc2
12		YDR514C	YPDcl2	YPDcl3	EtOHcl1	EtOHcl2	Galcl1	-	YPDnc2	YPDnc3	YPDnc4	EtOHnc1	EtOHnc2	Galnc1	Galnc2
12		YEL045C	YPDcl2	YPDcl3	EtOHcl1	-	Galcl1	YPDnc1	YPDnc2	YPDnc3	YPDnc4	EtOHnc1	EtOHnc2	Galnc1	Galnc2
12		YML116W-	YPDcl2	YPDcl3	EtOHcl1	EtOHcl2	Galcl1	YPDnc1	YPDnc2	-	YPDnc4	EtOHnc1	EtOHnc2	Galnc1	Galnc2
12		YFL031C-A	YPDcl2	YPDcl3	EtOHcl1	EtOHcl2	Galcl1	YPDnc1	YPDnc2	YPDnc3	YPDnc4	EtOHnc1	-	Galnc1	Galnc2
12	SOP4	YJL192C	YPDcl2	YPDcl3	EtOHcl1	-	Galcl1	YPDnc1	YPDnc2	YPDnc3	YPDnc4	EtOHnc1	EtOHnc2	Galnc1	Galnc2
12		YAL067W-A	YPDcl2	YPDcl3	EtOHcl1	-	Galcl1	YPDnc1	YPDnc2	YPDnc3	YPDnc4	EtOHnc1	EtOHnc2	Galnc1	Galnc2
12		YHR180W	YPDcl2	YPDcl3	EtOHcl1	EtOHcl2	Galcl1	YPDnc1	YPDnc2	YPDnc3	YPDnc4	EtOHnc1	EtOHnc2	Galnc1	-
12	SEC27	YGL137W	YPDcl2	-	EtOHcl1	EtOHcl2	Galcl1	YPDnc1	YPDnc2	YPDnc3	YPDnc4	EtOHnc1	EtOHnc2	Galnc1	Galnc2
12	TIM10	YHR005C-A	YPDcl2	YPDcl3	EtOHcl1	EtOHcl2	Galcl1	YPDnc1	-	YPDnc3	YPDnc4	EtOHnc1	EtOHnc2	Galnc1	Galnc2
12	VAM7	YGL212W	-	YPDcl3	EtOHcl1	EtOHcl2	Galcl1	YPDnc1	YPDnc2	YPDnc3	YPDnc4	EtOHnc1	EtOHnc2	Galnc1	Galnc2
12		YJL156W-A	-	YPDcl3	EtOHcl1	EtOHcl2	Galcl1	YPDnc1	YPDnc2	YPDnc3	YPDnc4	EtOHnc1	EtOHnc2	Galnc1	Galnc2
12	MNR2	YKL064W	YPDcl2	YPDcl3	EtOHcl1	EtOHcl2	Galcl1	YPDnc1	YPDnc2	YPDnc3	-	EtOHnc1	EtOHnc2	Galnc1	Galnc2
12	VIK1	YPL253C	YPDcl2	YPDcl3	EtOHcl1	EtOHcl2	-	YPDnc1	YPDnc2	YPDnc3	YPDnc4	EtOHnc1	EtOHnc2	Galnc1	Galnc2
12	CCW14	YLR390W-A	YPDcl2	YPDcl3	EtOHcl1	-	Galcl1	YPDnc1	YPDnc2	YPDnc3	YPDnc4	EtOHnc1	EtOHnc2	Galnc1	Galnc2
12		YPR146C	-	YPDcl3	EtOHcl1	EtOHcl2	Galcl1	YPDnc1	YPDnc2	YPDnc3	YPDnc4	EtOHnc1	EtOHnc2	Galnc1	Galnc2
12	NOP4	YPL043W	YPDcl2	YPDcl3	EtOHcl1	-	Galcl1	YPDnc1	YPDnc2	YPDnc3	YPDnc4	EtOHnc1	EtOHnc2	Galnc1	Galnc2
12	TFG1	YGR186W	YPDcl2	YPDcl3	EtOHcl1	EtOHcl2	Galcl1	-	YPDnc2	YPDnc3	YPDnc4	EtOHnc1	EtOHnc2	Galnc1	Galnc2
12	MRP4	YHL004W	YPDcl2	YPDcl3	EtOHcl1	EtOHcl2	Galcl1	YPDnc1	-	YPDnc3	YPDnc4	EtOHnc1	EtOHnc2	Galnc1	Galnc2
12	HPC2	YBR215W	YPDcl2	YPDcl3	EtOHcl1	EtOHcl2	Galcl1	YPDnc1	YPDnc2	YPDnc3	YPDnc4	EtOHnc1	EtOHnc2	Galnc1	-
12	SLC1	YDL052C	-	YPDcl3	EtOHcl1	EtOHcl2	Galcl1	YPDnc1	YPDnc2	YPDnc3	YPDnc4	EtOHnc1	EtOHnc2	Galnc1	Galnc2
12	HOR2	YER062C	YPDcl2	-	EtOHcl1	EtOHcl2	Galcl1	YPDnc1	YPDnc2	YPDnc3	YPDnc4	EtOHnc1	EtOHnc2	Galnc1	Galnc2

12	CDC50	YCR094W	YPDcl2	YPDcl3	EtOHcl1	EtOHcl2	Galcl1	-	YPDnc2	YPDnc3	YPDnc4	EtOHnc1	EtOHnc2	Galnc1	Galnc2
12	VTC4	YJL012C	YPDcl2	YPDcl3	EtOHcl1	EtOHcl2	Galcl1	YPDnc1	YPDnc2	YPDnc3	YPDnc4	EtOHnc1	-	Galnc1	Galnc2
12	FAA4	YMR246W	YPDcl2	YPDcl3	EtOHcl1	EtOHcl2	Galcl1	YPDnc1	YPDnc2	YPDnc3	YPDnc4	EtOHnc1	-	Galnc1	Galnc2
12		YAR047C	YPDcl2	YPDcl3	EtOHcl1	-	Galcl1	YPDnc1	YPDnc2	YPDnc3	YPDnc4	EtOHnc1	EtOHnc2	Galnc1	Galnc2
12	SWC5	YBR231C	YPDcl2	-	EtOHcl1	EtOHcl2	Galcl1	YPDnc1	YPDnc2	YPDnc3	YPDnc4	EtOHnc1	EtOHnc2	Galnc1	Galnc2
12	PCD1	YLR151C	YPDcl2	YPDcl3	EtOHcl1	-	Galcl1	YPDnc1	YPDnc2	YPDnc3	YPDnc4	EtOHnc1	EtOHnc2	Galnc1	Galnc2
12	PDC6	YGR087C	YPDcl2	YPDcl3	EtOHcl1	EtOHcl2	Galcl1	YPDnc1	YPDnc2	YPDnc3	YPDnc4	EtOHnc1	-	Galnc1	Galnc2
12		YMR262W	YPDcl2	-	EtOHcl1	EtOHcl2	Galcl1	YPDnc1	YPDnc2	YPDnc3	YPDnc4	EtOHnc1	EtOHnc2	Galnc1	Galnc2
12	REC8	YPR007C	YPDcl2	YPDcl3	EtOHcl1	-	Galcl1	YPDnc1	YPDnc2	YPDnc3	YPDnc4	EtOHnc1	EtOHnc2	Galnc1	Galnc2
12	SIP2	YGL208W	YPDcl2	YPDcl3	EtOHcl1	EtOHcl2	Galcl1	YPDnc1	YPDnc2	YPDnc3	YPDnc4	EtOHnc1	-	Galnc1	Galnc2
12		YGR226C	-	YPDcl3	EtOHcl1	EtOHcl2	Galcl1	YPDnc1	YPDnc2	YPDnc3	YPDnc4	EtOHnc1	EtOHnc2	Galnc1	Galnc2
12	UFD1	YGR048W	YPDcl2	YPDcl3	EtOHcl1	-	Galcl1	YPDnc1	YPDnc2	YPDnc3	YPDnc4	EtOHnc1	EtOHnc2	Galnc1	Galnc2
12	MCX1	YBR227C	YPDcl2	YPDcl3	EtOHcl1	EtOHcl2	Galcl1	-	YPDnc2	YPDnc3	YPDnc4	EtOHnc1	EtOHnc2	Galnc1	Galnc2
12	BIM1	YER016W	YPDcl2	YPDcl3	EtOHcl1	EtOHcl2	Galcl1	YPDnc1	YPDnc2	YPDnc3	-	EtOHnc1	EtOHnc2	Galnc1	Galnc2
12		YLR099W-A	YPDcl2	YPDcl3	EtOHcl1	EtOHcl2	-	YPDnc1	YPDnc2	YPDnc3	YPDnc4	EtOHnc1	EtOHnc2	Galnc1	Galnc2
12		YAL018C	YPDcl2	YPDcl3	EtOHcl1	-	Galcl1	YPDnc1	YPDnc2	YPDnc3	YPDnc4	EtOHnc1	EtOHnc2	Galnc1	Galnc2
12	LCL2	YLR104W	YPDcl2	YPDcl3	EtOHcl1	-	Galcl1	YPDnc1	YPDnc2	YPDnc3	YPDnc4	EtOHnc1	EtOHnc2	Galnc1	Galnc2
12	BER1	YLR412W	-	YPDcl3	EtOHcl1	EtOHcl2	Galcl1	YPDnc1	YPDnc2	YPDnc3	YPDnc4	EtOHnc1	EtOHnc2	Galnc1	Galnc2
12	YIP3	YNL044W	YPDcl2	YPDcl3	EtOHcl1	EtOHcl2	Galcl1	-	YPDnc2	YPDnc3	YPDnc4	EtOHnc1	EtOHnc2	Galnc1	Galnc2
12	RAD24	YER173W	YPDcl2	YPDcl3	EtOHcl1	-	Galcl1	YPDnc1	YPDnc2	YPDnc3	YPDnc4	EtOHnc1	EtOHnc2	Galnc1	Galnc2
12	STI1	YOR027W	YPDcl2	YPDcl3	EtOHcl1	EtOHcl2	Galcl1	YPDnc1	YPDnc2	YPDnc3	YPDnc4	EtOHnc1	-	Galnc1	Galnc2
12	CWC22	YGR278W	-	YPDcl3	EtOHcl1	EtOHcl2	Galcl1	YPDnc1	YPDnc2	YPDnc3	YPDnc4	EtOHnc1	EtOHnc2	Galnc1	Galnc2
12		YEL007W	YPDcl2	YPDcl3	EtOHcl1	-	Galcl1	YPDnc1	YPDnc2	YPDnc3	YPDnc4	EtOHnc1	EtOHnc2	Galnc1	Galnc2
12	YMC2	YBR104W	YPDcl2	YPDcl3	EtOHcl1	-	Galcl1	YPDnc1	YPDnc2	YPDnc3	YPDnc4	EtOHnc1	EtOHnc2	Galnc1	Galnc2
12	CAB3	YKL088W	-	YPDcl3	EtOHcl1	EtOHcl2	Galcl1	YPDnc1	YPDnc2	YPDnc3	YPDnc4	EtOHnc1	EtOHnc2	Galnc1	Galnc2
12		YNR004W	YPDcl2	-	EtOHcl1	EtOHcl2	Galcl1	YPDnc1	YPDnc2	YPDnc3	YPDnc4	EtOHnc1	EtOHnc2	Galnc1	Galnc2
12		YNL194C	YPDcl2	YPDcl3	EtOHcl1	-	Galcl1	YPDnc1	YPDnc2	YPDnc3	YPDnc4	EtOHnc1	EtOHnc2	Galnc1	Galnc2
12	SWM1	YDR260C	-	YPDcl3	EtOHcl1	EtOHcl2	Galcl1	YPDnc1	YPDnc2	YPDnc3	YPDnc4	EtOHnc1	EtOHnc2	Galnc1	Galnc2
12	DAM1	YGR113W	YPDcl2	YPDcl3	EtOHcl1	-	Galcl1	YPDnc1	YPDnc2	YPDnc3	YPDnc4	EtOHnc1	EtOHnc2	Galnc1	Galnc2
12		YDR340W	YPDcl2	YPDcl3	EtOHcl1	-	Galcl1	YPDnc1	YPDnc2	YPDnc3	YPDnc4	EtOHnc1	EtOHnc2	Galnc1	Galnc2
12	ESC1	YMR219W	YPDcl2	YPDcl3	EtOHcl1	-	Galcl1	YPDnc1	YPDnc2	YPDnc3	YPDnc4	EtOHnc1	EtOHnc2	Galnc1	Galnc2
12	REV7	YIL139C	YPDcl2	YPDcl3	EtOHcl1	-	Galcl1	YPDnc1	YPDnc2	YPDnc3	YPDnc4	EtOHnc1	EtOHnc2	Galnc1	Galnc2
12	PXA2	YKL188C	YPDcl2	YPDcl3	EtOHcl1	-	Galcl1	YPDnc1	YPDnc2	YPDnc3	YPDnc4	EtOHnc1	EtOHnc2	Galnc1	Galnc2
12		YDL183C	YPDcl2	YPDcl3	EtOHcl1	EtOHcl2	Galcl1	YPDnc1	YPDnc2	YPDnc3	YPDnc4	EtOHnc1	-	Galnc1	Galnc2

12		YER140W	YPDcl2	YPDcl3	EtOHcl1	EtOHcl2	Galcl1	YPDnc1	-	YPDnc3	YPDnc4	EtOHnc1	EtOHnc2	Galnc1	Galnc2
12	PYK2	YOR347C	YPDcl2	YPDcl3	EtOHcl1	EtOHcl2	Galcl1	YPDnc1	YPDnc2	YPDnc3	-	EtOHnc1	EtOHnc2	Galnc1	Galnc2
12	AAT2	YLR027C	YPDcl2	YPDcl3	EtOHcl1	-	Galcl1	YPDnc1	YPDnc2	YPDnc3	YPDnc4	EtOHnc1	EtOHnc2	Galnc1	Galnc2
12	CSI2	YOL007C	YPDcl2	-	EtOHcl1	EtOHcl2	Galcl1	YPDnc1	YPDnc2	YPDnc3	YPDnc4	EtOHnc1	EtOHnc2	Galnc1	Galnc2
12		YML047W-	YPDcl2	YPDcl3	EtOHcl1	EtOHcl2	Galcl1	YPDnc1	YPDnc2	YPDnc3	YPDnc4	-	EtOHnc2	Galnc1	Galnc2
12	NOP12	YOL041C	YPDcl2	-	EtOHcl1	EtOHcl2	Galcl1	YPDnc1	YPDnc2	YPDnc3	YPDnc4	EtOHnc1	EtOHnc2	Galnc1	Galnc2
12		YMR122C	YPDcl2	YPDcl3	EtOHcl1	-	Galcl1	YPDnc1	YPDnc2	YPDnc3	YPDnc4	EtOHnc1	EtOHnc2	Galnc1	Galnc2
12	GLY1	YEL046C	YPDcl2	-	EtOHcl1	EtOHcl2	Galcl1	YPDnc1	YPDnc2	YPDnc3	YPDnc4	EtOHnc1	EtOHnc2	Galnc1	Galnc2
12		YMR307C-/	-	YPDcl3	EtOHcl1	EtOHcl2	Galcl1	YPDnc1	YPDnc2	YPDnc3	YPDnc4	EtOHnc1	EtOHnc2	Galnc1	Galnc2
12		YLR392C	YPDcl2	YPDcl3	EtOHcl1	-	Galcl1	YPDnc1	YPDnc2	YPDnc3	YPDnc4	EtOHnc1	EtOHnc2	Galnc1	Galnc2
12	PRP38	YGR075C	YPDcl2	YPDcl3	EtOHcl1	EtOHcl2	Galcl1	-	YPDnc2	YPDnc3	YPDnc4	EtOHnc1	EtOHnc2	Galnc1	Galnc2
12	PAC2	YER007W	YPDcl2	YPDcl3	EtOHcl1	EtOHcl2	Galcl1	YPDnc1	YPDnc2	YPDnc3	YPDnc4	-	EtOHnc2	Galnc1	Galnc2
12	NUR1	YDL089W	YPDcl2	YPDcl3	EtOHcl1	EtOHcl2	Galcl1	YPDnc1	YPDnc2	YPDnc3	-	EtOHnc1	EtOHnc2	Galnc1	Galnc2
12	MAD1	YGL086W	YPDcl2	YPDcl3	EtOHcl1	-	Galcl1	YPDnc1	YPDnc2	YPDnc3	YPDnc4	EtOHnc1	EtOHnc2	Galnc1	Galnc2
12		YMR074C	YPDcl2	-	EtOHcl1	EtOHcl2	Galcl1	YPDnc1	YPDnc2	YPDnc3	YPDnc4	EtOHnc1	EtOHnc2	Galnc1	Galnc2
12		YDR341C	YPDcl2	YPDcl3	EtOHcl1	-	Galcl1	YPDnc1	YPDnc2	YPDnc3	YPDnc4	EtOHnc1	EtOHnc2	Galnc1	Galnc2
12	TMA23	YMR269W	YPDcl2	-	EtOHcl1	EtOHcl2	Galcl1	YPDnc1	YPDnc2	YPDnc3	YPDnc4	EtOHnc1	EtOHnc2	Galnc1	Galnc2
12	OPI1	YHL020C	YPDcl2	YPDcl3	EtOHcl1	-	Galcl1	YPDnc1	YPDnc2	YPDnc3	YPDnc4	EtOHnc1	EtOHnc2	Galnc1	Galnc2
12	SST2	YLR452C	YPDcl2	YPDcl3	EtOHcl1	EtOHcl2	Galcl1	YPDnc1	YPDnc2	YPDnc3	-	EtOHnc1	EtOHnc2	Galnc1	Galnc2
12	TAF4	YMR005W	YPDcl2	YPDcl3	EtOHcl1	-	Galcl1	YPDnc1	YPDnc2	YPDnc3	YPDnc4	EtOHnc1	EtOHnc2	Galnc1	Galnc2
12		YCR015C	YPDcl2	YPDcl3	EtOHcl1	EtOHcl2	Galcl1	-	YPDnc2	YPDnc3	YPDnc4	EtOHnc1	EtOHnc2	Galnc1	Galnc2
12		YMR135W-	-	YPDcl3	EtOHcl1	EtOHcl2	Galcl1	YPDnc1	YPDnc2	YPDnc3	YPDnc4	EtOHnc1	EtOHnc2	Galnc1	Galnc2
12	OPI6	YDL096C	-	YPDcl3	EtOHcl1	EtOHcl2	Galcl1	YPDnc1	YPDnc2	YPDnc3	YPDnc4	EtOHnc1	EtOHnc2	Galnc1	Galnc2
12		YGR149W	YPDcl2	YPDcl3	EtOHcl1	EtOHcl2	Galcl1	YPDnc1	YPDnc2	YPDnc3	YPDnc4	EtOHnc1	EtOHnc2	-	Galnc2
12		YGL258W-/	YPDcl2	YPDcl3	EtOHcl1	-	Galcl1	YPDnc1	YPDnc2	YPDnc3	YPDnc4	EtOHnc1	EtOHnc2	Galnc1	Galnc2
12	EAF5	YEL018W	YPDcl2	YPDcl3	EtOHcl1	EtOHcl2	-	YPDnc1	YPDnc2	YPDnc3	YPDnc4	EtOHnc1	EtOHnc2	Galnc1	Galnc2
12	GPM2	YDL021W	YPDcl2	YPDcl3	EtOHcl1	EtOHcl2	Galcl1	YPDnc1	YPDnc2	YPDnc3	YPDnc4	EtOHnc1	-	Galnc1	Galnc2
12		YBR074W	YPDcl2	YPDcl3	-	EtOHcl2	Galcl1	YPDnc1	YPDnc2	YPDnc3	YPDnc4	EtOHnc1	EtOHnc2	Galnc1	Galnc2
12		YKR015C	YPDcl2	YPDcl3	EtOHcl1	-	Galcl1	YPDnc1	YPDnc2	YPDnc3	YPDnc4	EtOHnc1	EtOHnc2	Galnc1	Galnc2
12	MEC1	YBR136W	YPDcl2	-	EtOHcl1	EtOHcl2	Galcl1	YPDnc1	YPDnc2	YPDnc3	YPDnc4	EtOHnc1	EtOHnc2	Galnc1	Galnc2
12		YGR018C	-	YPDcl3	EtOHcl1	EtOHcl2	Galcl1	YPDnc1	YPDnc2	YPDnc3	YPDnc4	EtOHnc1	EtOHnc2	Galnc1	Galnc2
12	RCY1	YJL204C	YPDcl2	-	EtOHcl1	EtOHcl2	Galcl1	YPDnc1	YPDnc2	YPDnc3	YPDnc4	EtOHnc1	EtOHnc2	Galnc1	Galnc2
12		YMR294W-	YPDcl2	YPDcl3	EtOHcl1	EtOHcl2	Galcl1	YPDnc1	YPDnc2	YPDnc3	-	EtOHnc1	EtOHnc2	Galnc1	Galnc2
12	RIM2	YBR192W	YPDcl2	-	EtOHcl1	EtOHcl2	Galcl1	YPDnc1	YPDnc2	YPDnc3	YPDnc4	EtOHnc1	EtOHnc2	Galnc1	Galnc2

12		YGR160W	YPDcl2	YPDcl3	EtOHcl1	-	Galcl1	YPDnc1	YPDnc2	YPDnc3	YPDnc4	EtOHnc1	EtOHnc2	Galnc1	Galnc2
12		YGR017W	-	YPDcl3	EtOHcl1	EtOHcl2	Galcl1	YPDnc1	YPDnc2	YPDnc3	YPDnc4	EtOHnc1	EtOHnc2	Galnc1	Galnc2
12	AYR1	YIL124W	-	YPDcl3	EtOHcl1	EtOHcl2	Galcl1	YPDnc1	YPDnc2	YPDnc3	YPDnc4	EtOHnc1	EtOHnc2	Galnc1	Galnc2
12	RRP9	YPR137W	YPDcl2	-	EtOHcl1	EtOHcl2	Galcl1	YPDnc1	YPDnc2	YPDnc3	YPDnc4	EtOHnc1	EtOHnc2	Galnc1	Galnc2
12	RPL9B	YNL067W	-	YPDcl3	EtOHcl1	EtOHcl2	Galcl1	YPDnc1	YPDnc2	YPDnc3	YPDnc4	EtOHnc1	EtOHnc2	Galnc1	Galnc2
12	LPE10	YPL060W	YPDcl2	YPDcl3	-	EtOHcl2	Galcl1	YPDnc1	YPDnc2	YPDnc3	YPDnc4	EtOHnc1	EtOHnc2	Galnc1	Galnc2
12	TTI1	YKL033W	YPDcl2	-	EtOHcl1	EtOHcl2	Galcl1	YPDnc1	YPDnc2	YPDnc3	YPDnc4	EtOHnc1	EtOHnc2	Galnc1	Galnc2
12		YLR049C	-	YPDcl3	EtOHcl1	EtOHcl2	Galcl1	YPDnc1	YPDnc2	YPDnc3	YPDnc4	EtOHnc1	EtOHnc2	Galnc1	Galnc2
12	SPT16	YGL207W	YPDcl2	YPDcl3	-	EtOHcl2	Galcl1	YPDnc1	YPDnc2	YPDnc3	YPDnc4	EtOHnc1	EtOHnc2	Galnc1	Galnc2
12	NKP2	YLR315W	-	YPDcl3	EtOHcl1	EtOHcl2	Galcl1	YPDnc1	YPDnc2	YPDnc3	YPDnc4	EtOHnc1	EtOHnc2	Galnc1	Galnc2
12	KEG1	YFR042W	YPDcl2	YPDcl3	EtOHcl1	EtOHcl2	Galcl1	YPDnc1	YPDnc2	YPDnc3	YPDnc4	EtOHnc1	-	Galnc1	Galnc2
12	SRP68	YPL243W	YPDcl2	YPDcl3	-	EtOHcl2	Galcl1	YPDnc1	YPDnc2	YPDnc3	YPDnc4	EtOHnc1	EtOHnc2	Galnc1	Galnc2
12	GAL7	YBR018C	YPDcl2	YPDcl3	EtOHcl1	EtOHcl2	-	YPDnc1	YPDnc2	YPDnc3	YPDnc4	EtOHnc1	EtOHnc2	Galnc1	Galnc2
12	MSN2	YMR037C	YPDcl2	-	EtOHcl1	EtOHcl2	Galcl1	YPDnc1	YPDnc2	YPDnc3	YPDnc4	EtOHnc1	EtOHnc2	Galnc1	Galnc2
12	MSA1	YOR066W	-	YPDcl3	EtOHcl1	EtOHcl2	Galcl1	YPDnc1	YPDnc2	YPDnc3	YPDnc4	EtOHnc1	EtOHnc2	Galnc1	Galnc2
12	HMS2	YJR147W	-	YPDcl3	EtOHcl1	EtOHcl2	Galcl1	YPDnc1	YPDnc2	YPDnc3	YPDnc4	EtOHnc1	EtOHnc2	Galnc1	Galnc2
12	NUP192	YJL039C	YPDcl2	YPDcl3	EtOHcl1	EtOHcl2	Galcl1	YPDnc1	YPDnc2	YPDnc3	YPDnc4	-	EtOHnc2	Galnc1	Galnc2
12		YNL200C	-	YPDcl3	EtOHcl1	EtOHcl2	Galcl1	YPDnc1	YPDnc2	YPDnc3	YPDnc4	EtOHnc1	EtOHnc2	Galnc1	Galnc2
12	THI4	YGR144W	YPDcl2	YPDcl3	-	EtOHcl2	Galcl1	YPDnc1	YPDnc2	YPDnc3	YPDnc4	EtOHnc1	EtOHnc2	Galnc1	Galnc2
12	PAC1	YOR269W	YPDcl2	YPDcl3	EtOHcl1	EtOHcl2	Galcl1	YPDnc1	YPDnc2	YPDnc3	YPDnc4	EtOHnc1	-	Galnc1	Galnc2
12	SVS1	YPL163C	YPDcl2	YPDcl3	-	EtOHcl2	Galcl1	YPDnc1	YPDnc2	YPDnc3	YPDnc4	EtOHnc1	EtOHnc2	Galnc1	Galnc2
12	RPL37B	YDR500C	YPDcl2	YPDcl3	EtOHcl1	EtOHcl2	Galcl1	YPDnc1	YPDnc2	YPDnc3	YPDnc4	EtOHnc1	-	Galnc1	Galnc2
12		YMR258C	YPDcl2	YPDcl3	EtOHcl1	EtOHcl2	Galcl1	YPDnc1	YPDnc2	YPDnc3	YPDnc4	EtOHnc1	EtOHnc2	Galnc1	-
12	POB3	YML069W	YPDcl2	YPDcl3	EtOHcl1	EtOHcl2	Galcl1	YPDnc1	YPDnc2	YPDnc3	YPDnc4	-	EtOHnc2	Galnc1	Galnc2
12		YNL266W	YPDcl2	-	EtOHcl1	EtOHcl2	Galcl1	YPDnc1	YPDnc2	YPDnc3	YPDnc4	EtOHnc1	EtOHnc2	Galnc1	Galnc2
12	ADE6	YGR061C	YPDcl2	YPDcl3	EtOHcl1	-	Galcl1	YPDnc1	YPDnc2	YPDnc3	YPDnc4	EtOHnc1	EtOHnc2	Galnc1	Galnc2
12	TUB3	YML124C	YPDcl2	YPDcl3	EtOHcl1	EtOHcl2	Galcl1	YPDnc1	YPDnc2	YPDnc3	YPDnc4	EtOHnc1	-	Galnc1	Galnc2
12	DAD4	YDR320C-A	YPDcl2	YPDcl3	-	EtOHcl2	Galcl1	YPDnc1	YPDnc2	YPDnc3	YPDnc4	EtOHnc1	EtOHnc2	Galnc1	Galnc2
12	PEX25	YPL112C	-	YPDcl3	EtOHcl1	EtOHcl2	Galcl1	YPDnc1	YPDnc2	YPDnc3	YPDnc4	EtOHnc1	EtOHnc2	Galnc1	Galnc2
12	YAP3	YHL009C	YPDcl2	YPDcl3	EtOHcl1	EtOHcl2	-	YPDnc1	YPDnc2	YPDnc3	YPDnc4	EtOHnc1	EtOHnc2	Galnc1	Galnc2
12		YOL036W	-	YPDcl3	EtOHcl1	EtOHcl2	Galcl1	YPDnc1	YPDnc2	YPDnc3	YPDnc4	EtOHnc1	EtOHnc2	Galnc1	Galnc2
12	ABZ1	YNR033W	YPDcl2	YPDcl3	EtOHcl1	-	Galcl1	YPDnc1	YPDnc2	YPDnc3	YPDnc4	EtOHnc1	EtOHnc2	Galnc1	Galnc2
12	ROX3	YBL093C	YPDcl2	YPDcl3	EtOHcl1	EtOHcl2	Galcl1	YPDnc1	YPDnc2	YPDnc3	-	EtOHnc1	EtOHnc2	Galnc1	Galnc2
12	MDM12	YOL009C	YPDcl2	YPDcl3	EtOHcl1	-	Galcl1	YPDnc1	YPDnc2	YPDnc3	YPDnc4	EtOHnc1	EtOHnc2	Galnc1	Galnc2

12	SWT21	YNL187W	YPDcl2	YPDcl3	EtOHcl1	-	Galcl1	YPDnc1	YPDnc2	YPDnc3	YPDnc4	EtOHnc1	EtOHnc2	Galnc1	Galnc2
12		YEL067C	YPDcl2	YPDcl3	EtOHcl1	-	Galcl1	YPDnc1	YPDnc2	YPDnc3	YPDnc4	EtOHnc1	EtOHnc2	Galnc1	Galnc2
12		YAL056C-A	YPDcl2	YPDcl3	-	EtOHcl2	Galcl1	YPDnc1	YPDnc2	YPDnc3	YPDnc4	EtOHnc1	EtOHnc2	Galnc1	Galnc2
12	SEC61	YLR378C	YPDcl2	YPDcl3	EtOHcl1	-	Galcl1	YPDnc1	YPDnc2	YPDnc3	YPDnc4	EtOHnc1	EtOHnc2	Galnc1	Galnc2
12	POL4	YCR014C	YPDcl2	YPDcl3	EtOHcl1	EtOHcl2	Galcl1	-	YPDnc2	YPDnc3	YPDnc4	EtOHnc1	EtOHnc2	Galnc1	Galnc2
12	MRPL19	YNL185C	YPDcl2	YPDcl3	EtOHcl1	EtOHcl2	Galcl1	YPDnc1	YPDnc2	YPDnc3	YPDnc4	EtOHnc1	EtOHnc2	Galnc1	-
12	CRH1	YGR189C	YPDcl2	YPDcl3	EtOHcl1	EtOHcl2	Galcl1	-	YPDnc2	YPDnc3	YPDnc4	EtOHnc1	EtOHnc2	Galnc1	Galnc2
12	HXT7	YDR342C	YPDcl2	YPDcl3	EtOHcl1	-	Galcl1	YPDnc1	YPDnc2	YPDnc3	YPDnc4	EtOHnc1	EtOHnc2	Galnc1	Galnc2
12	FOL2	YGR267C	YPDcl2	YPDcl3	EtOHcl1	EtOHcl2	Galcl1	YPDnc1	YPDnc2	YPDnc3	YPDnc4	EtOHnc1	EtOHnc2	-	Galnc2
12	JLP2	YMR132C	YPDcl2	YPDcl3	EtOHcl1	-	Galcl1	YPDnc1	YPDnc2	YPDnc3	YPDnc4	EtOHnc1	EtOHnc2	Galnc1	Galnc2
12	MTL1	YGR023W	-	YPDcl3	EtOHcl1	EtOHcl2	Galcl1	YPDnc1	YPDnc2	YPDnc3	YPDnc4	EtOHnc1	EtOHnc2	Galnc1	Galnc2
12	DAP1	YPL170W	-	YPDcl3	EtOHcl1	EtOHcl2	Galcl1	YPDnc1	YPDnc2	YPDnc3	YPDnc4	EtOHnc1	EtOHnc2	Galnc1	Galnc2
12	HIS7	YBR248C	-	YPDcl3	EtOHcl1	EtOHcl2	Galcl1	YPDnc1	YPDnc2	YPDnc3	YPDnc4	EtOHnc1	EtOHnc2	Galnc1	Galnc2
12	MSC2	YDR205W	-	YPDcl3	EtOHcl1	EtOHcl2	Galcl1	YPDnc1	YPDnc2	YPDnc3	YPDnc4	EtOHnc1	EtOHnc2	Galnc1	Galnc2
12	PDX1	YGR193C	YPDcl2	YPDcl3	EtOHcl1	EtOHcl2	Galcl1	YPDnc1	YPDnc2	YPDnc3	YPDnc4	EtOHnc1	EtOHnc2	Galnc1	-
12		YOL029C	-	YPDcl3	EtOHcl1	EtOHcl2	Galcl1	YPDnc1	YPDnc2	YPDnc3	YPDnc4	EtOHnc1	EtOHnc2	Galnc1	Galnc2
12	VPS71	YML041C	YPDcl2	YPDcl3	EtOHcl1	EtOHcl2	Galcl1	YPDnc1	YPDnc2	YPDnc3	YPDnc4	EtOHnc1	EtOHnc2	-	Galnc2
12	NVJ1	YHR195W	YPDcl2	YPDcl3	-	EtOHcl2	Galcl1	YPDnc1	YPDnc2	YPDnc3	YPDnc4	EtOHnc1	EtOHnc2	Galnc1	Galnc2
12		YKL044W	YPDcl2	YPDcl3	EtOHcl1	EtOHcl2	Galcl1	YPDnc1	YPDnc2	YPDnc3	YPDnc4	EtOHnc1	-	Galnc1	Galnc2
12		YOR296W	YPDcl2	YPDcl3	EtOHcl1	EtOHcl2	Galcl1	-	YPDnc2	YPDnc3	YPDnc4	EtOHnc1	EtOHnc2	Galnc1	Galnc2
12	RTC5	YOR118W	YPDcl2	YPDcl3	EtOHcl1	EtOHcl2	-	YPDnc1	YPDnc2	YPDnc3	YPDnc4	EtOHnc1	EtOHnc2	Galnc1	Galnc2
12	GAL10	YBR019C	YPDcl2	YPDcl3	EtOHcl1	EtOHcl2	-	YPDnc1	YPDnc2	YPDnc3	YPDnc4	EtOHnc1	EtOHnc2	Galnc1	Galnc2
12	ABP140	YOR239W	-	YPDcl3	EtOHcl1	EtOHcl2	Galcl1	YPDnc1	YPDnc2	YPDnc3	YPDnc4	EtOHnc1	EtOHnc2	Galnc1	Galnc2
12		YNL103W-/	-	YPDcl3	EtOHcl1	EtOHcl2	Galcl1	YPDnc1	YPDnc2	YPDnc3	YPDnc4	EtOHnc1	EtOHnc2	Galnc1	Galnc2
12	PLB2	YMR006C	YPDcl2	YPDcl3	EtOHcl1	-	Galcl1	YPDnc1	YPDnc2	YPDnc3	YPDnc4	EtOHnc1	EtOHnc2	Galnc1	Galnc2
12	PAI3	YMR174C	YPDcl2	YPDcl3	EtOHcl1	EtOHcl2	Galcl1	YPDnc1	YPDnc2	YPDnc3	YPDnc4	EtOHnc1	-	Galnc1	Galnc2
12	PAP1	YKR002W	YPDcl2	YPDcl3	-	EtOHcl2	Galcl1	YPDnc1	YPDnc2	YPDnc3	YPDnc4	EtOHnc1	EtOHnc2	Galnc1	Galnc2
12	TVP38	YKR088C	YPDcl2	YPDcl3	EtOHcl1	EtOHcl2	Galcl1	YPDnc1	YPDnc2	YPDnc3	YPDnc4	EtOHnc1	-	Galnc1	Galnc2
12	UIP5	YKR044W	-	YPDcl3	EtOHcl1	EtOHcl2	Galcl1	YPDnc1	YPDnc2	YPDnc3	YPDnc4	EtOHnc1	EtOHnc2	Galnc1	Galnc2
12	ADE12	YNL220W	YPDcl2	YPDcl3	-	EtOHcl2	Galcl1	YPDnc1	YPDnc2	YPDnc3	YPDnc4	EtOHnc1	EtOHnc2	Galnc1	Galnc2
12	GGA2	YHR108W	YPDcl2	YPDcl3	EtOHcl1	-	Galcl1	YPDnc1	YPDnc2	YPDnc3	YPDnc4	EtOHnc1	EtOHnc2	Galnc1	Galnc2
12	DYN3	YMR299C	YPDcl2	YPDcl3	EtOHcl1	EtOHcl2	Galcl1	YPDnc1	YPDnc2	-	YPDnc4	EtOHnc1	EtOHnc2	Galnc1	Galnc2
12	BCK1	YJL095W	YPDcl2	YPDcl3	-	EtOHcl2	Galcl1	YPDnc1	YPDnc2	YPDnc3	YPDnc4	EtOHnc1	EtOHnc2	Galnc1	Galnc2
12		YML053C	YPDcl2	YPDcl3	EtOHcl1	EtOHcl2	-	YPDnc1	YPDnc2	YPDnc3	YPDnc4	EtOHnc1	EtOHnc2	Galnc1	Galnc2

12	MRPL32	YCR003W	YPDcl2	YPDcl3	EtOHcl1	EtOHcl2	Galcl1	YPDnc1	YPDnc2	YPDnc3	YPDnc4	EtOHnc1	-	Galnc1	Galnc2
12	RAD50	YNL250W	YPDcl2	-	EtOHcl1	EtOHcl2	Galcl1	YPDnc1	YPDnc2	YPDnc3	YPDnc4	EtOHnc1	EtOHnc2	Galnc1	Galnc2
12		YDR269C	YPDcl2	YPDcl3	EtOHcl1	EtOHcl2	-	YPDnc1	YPDnc2	YPDnc3	YPDnc4	EtOHnc1	EtOHnc2	Galnc1	Galnc2
12	MRPL15	YLR312W-A	YPDcl2	YPDcl3	EtOHcl1	-	Galcl1	YPDnc1	YPDnc2	YPDnc3	YPDnc4	EtOHnc1	EtOHnc2	Galnc1	Galnc2
12	GAA1	YLR088W	YPDcl2	YPDcl3	EtOHcl1	EtOHcl2	Galcl1	YPDnc1	YPDnc2	YPDnc3	-	EtOHnc1	EtOHnc2	Galnc1	Galnc2
12	SIM1	YIL123W	YPDcl2	YPDcl3	EtOHcl1	-	Galcl1	YPDnc1	YPDnc2	YPDnc3	YPDnc4	EtOHnc1	EtOHnc2	Galnc1	Galnc2
12		YER090C-A	-	YPDcl3	EtOHcl1	EtOHcl2	Galcl1	YPDnc1	YPDnc2	YPDnc3	YPDnc4	EtOHnc1	EtOHnc2	Galnc1	Galnc2
12		YGR121W-A	YPDcl2	YPDcl3	EtOHcl1	EtOHcl2	Galcl1	YPDnc1	YPDnc2	YPDnc3	YPDnc4	EtOHnc1	-	Galnc1	Galnc2
12	POM152	YMR129W	YPDcl2	YPDcl3	EtOHcl1	EtOHcl2	-	YPDnc1	YPDnc2	YPDnc3	YPDnc4	EtOHnc1	EtOHnc2	Galnc1	Galnc2
12	ACS2	YLR153C	YPDcl2	YPDcl3	EtOHcl1	-	Galcl1	YPDnc1	YPDnc2	YPDnc3	YPDnc4	EtOHnc1	EtOHnc2	Galnc1	Galnc2
12	YGK3	YOL128C	YPDcl2	-	EtOHcl1	EtOHcl2	Galcl1	YPDnc1	YPDnc2	YPDnc3	YPDnc4	EtOHnc1	EtOHnc2	Galnc1	Galnc2
12	BUD20	YLR074C	YPDcl2	YPDcl3	EtOHcl1	EtOHcl2	Galcl1	-	YPDnc2	YPDnc3	YPDnc4	EtOHnc1	EtOHnc2	Galnc1	Galnc2
12		YJL016W	YPDcl2	-	EtOHcl1	EtOHcl2	Galcl1	YPDnc1	YPDnc2	YPDnc3	YPDnc4	EtOHnc1	EtOHnc2	Galnc1	Galnc2
12	HRR25	YPL204W	YPDcl2	YPDcl3	EtOHcl1	EtOHcl2	Galcl1	YPDnc1	YPDnc2	YPDnc3	YPDnc4	EtOHnc1	-	Galnc1	Galnc2
12		YLR122C	YPDcl2	YPDcl3	EtOHcl1	-	Galcl1	YPDnc1	YPDnc2	YPDnc3	YPDnc4	EtOHnc1	EtOHnc2	Galnc1	Galnc2
12	RPL23A	YBL087C	YPDcl2	YPDcl3	EtOHcl1	-	Galcl1	YPDnc1	YPDnc2	YPDnc3	YPDnc4	EtOHnc1	EtOHnc2	Galnc1	Galnc2
12	IMP2	YMR035W	YPDcl2	-	EtOHcl1	EtOHcl2	Galcl1	YPDnc1	YPDnc2	YPDnc3	YPDnc4	EtOHnc1	EtOHnc2	Galnc1	Galnc2
12		YIL142C-A	-	YPDcl3	EtOHcl1	EtOHcl2	Galcl1	YPDnc1	YPDnc2	YPDnc3	YPDnc4	EtOHnc1	EtOHnc2	Galnc1	Galnc2
12	MGM1	YOR211C	YPDcl2	-	EtOHcl1	EtOHcl2	Galcl1	YPDnc1	YPDnc2	YPDnc3	YPDnc4	EtOHnc1	EtOHnc2	Galnc1	Galnc2
12	LOT5	YKL183W	YPDcl2	YPDcl3	EtOHcl1	EtOHcl2	Galcl1	YPDnc1	YPDnc2	YPDnc3	-	EtOHnc1	EtOHnc2	Galnc1	Galnc2
12	RPS23A	YGR118W	-	YPDcl3	EtOHcl1	EtOHcl2	Galcl1	YPDnc1	YPDnc2	YPDnc3	YPDnc4	EtOHnc1	EtOHnc2	Galnc1	Galnc2
12	ARO1	YDR127W	YPDcl2	YPDcl3	EtOHcl1	EtOHcl2	Galcl1	YPDnc1	YPDnc2	YPDnc3	-	EtOHnc1	EtOHnc2	Galnc1	Galnc2
12	MTG1	YMR097C	YPDcl2	YPDcl3	EtOHcl1	EtOHcl2	Galcl1	YPDnc1	YPDnc2	YPDnc3	YPDnc4	EtOHnc1	-	Galnc1	Galnc2
12		YBR051W	YPDcl2	YPDcl3	EtOHcl1	-	Galcl1	YPDnc1	YPDnc2	YPDnc3	YPDnc4	EtOHnc1	EtOHnc2	Galnc1	Galnc2
12	RSC58	YLR033W	YPDcl2	YPDcl3	EtOHcl1	-	Galcl1	YPDnc1	YPDnc2	YPDnc3	YPDnc4	EtOHnc1	EtOHnc2	Galnc1	Galnc2
12	ARN1	YHL040C	YPDcl2	YPDcl3	EtOHcl1	EtOHcl2	Galcl1	YPDnc1	YPDnc2	YPDnc3	-	EtOHnc1	EtOHnc2	Galnc1	Galnc2
12	RPL24B	YGR148C	YPDcl2	YPDcl3	EtOHcl1	EtOHcl2	Galcl1	YPDnc1	YPDnc2	YPDnc3	YPDnc4	EtOHnc1	EtOHnc2	Galnc1	-
12		YGR242W	YPDcl2	YPDcl3	EtOHcl1	EtOHcl2	Galcl1	YPDnc1	YPDnc2	YPDnc3	-	EtOHnc1	EtOHnc2	Galnc1	Galnc2
12		YBR182C-A	YPDcl2	YPDcl3	EtOHcl1	-	Galcl1	YPDnc1	YPDnc2	YPDnc3	YPDnc4	EtOHnc1	EtOHnc2	Galnc1	Galnc2
12	MET8	YBR213W	YPDcl2	YPDcl3	EtOHcl1	EtOHcl2	Galcl1	YPDnc1	-	YPDnc3	YPDnc4	EtOHnc1	EtOHnc2	Galnc1	Galnc2
12		YPL205C	YPDcl2	YPDcl3	EtOHcl1	EtOHcl2	Galcl1	YPDnc1	YPDnc2	YPDnc3	YPDnc4	EtOHnc1	-	Galnc1	Galnc2
12	CAT8	YMR280C	YPDcl2	-	EtOHcl1	EtOHcl2	Galcl1	YPDnc1	YPDnc2	YPDnc3	YPDnc4	EtOHnc1	EtOHnc2	Galnc1	Galnc2
12	MDH1	YKL085W	YPDcl2	YPDcl3	EtOHcl1	EtOHcl2	Galcl1	YPDnc1	YPDnc2	YPDnc3	YPDnc4	EtOHnc1	-	Galnc1	Galnc2
12		YLR036C	YPDcl2	YPDcl3	EtOHcl1	EtOHcl2	Galcl1	YPDnc1	YPDnc2	YPDnc3	YPDnc4	-	EtOHnc2	Galnc1	Galnc2

12	SRP1	YNL189W	YPDcl2	YPDcl3	EtOHcl1	EtOHcl2	Galcl1	-	YPDnc2	YPDnc3	YPDnc4	EtOHnc1	EtOHnc2	Galnc1	Galnc2
12	KIN2	YLR096W	YPDcl2	-	EtOHcl1	EtOHcl2	Galcl1	YPDnc1	YPDnc2	YPDnc3	YPDnc4	EtOHnc1	EtOHnc2	Galnc1	Galnc2
12	RPT3	YDR394W	YPDcl2	YPDcl3	-	EtOHcl2	Galcl1	YPDnc1	YPDnc2	YPDnc3	YPDnc4	EtOHnc1	EtOHnc2	Galnc1	Galnc2
12	PCL5	YHR071W	YPDcl2	YPDcl3	EtOHcl1	EtOHcl2	Galcl1	YPDnc1	YPDnc2	YPDnc3	YPDnc4	EtOHnc1	EtOHnc2	Galnc1	-
12	ROT1	YMR200W	YPDcl2	YPDcl3	EtOHcl1	-	Galcl1	YPDnc1	YPDnc2	YPDnc3	YPDnc4	EtOHnc1	EtOHnc2	Galnc1	Galnc2
12	RRT13	YER066W	YPDcl2	YPDcl3	EtOHcl1	EtOHcl2	Galcl1	YPDnc1	YPDnc2	YPDnc3	YPDnc4	EtOHnc1	-	Galnc1	Galnc2
12	EKI1	YDR147W	YPDcl2	YPDcl3	EtOHcl1	-	Galcl1	YPDnc1	YPDnc2	YPDnc3	YPDnc4	EtOHnc1	EtOHnc2	Galnc1	Galnc2
12	RIF1	YBR275C	YPDcl2	YPDcl3	EtOHcl1	EtOHcl2	Galcl1	YPDnc1	YPDnc2	YPDnc3	YPDnc4	EtOHnc1	-	Galnc1	Galnc2
12	DCP2	YNL118C	YPDcl2	YPDcl3	EtOHcl1	-	Galcl1	YPDnc1	YPDnc2	YPDnc3	YPDnc4	EtOHnc1	EtOHnc2	Galnc1	Galnc2
12	LYS9	YNR050C	YPDcl2	-	EtOHcl1	EtOHcl2	Galcl1	YPDnc1	YPDnc2	YPDnc3	YPDnc4	EtOHnc1	EtOHnc2	Galnc1	Galnc2
12		YKL023C-A	YPDcl2	YPDcl3	EtOHcl1	EtOHcl2	-	YPDnc1	YPDnc2	YPDnc3	YPDnc4	EtOHnc1	EtOHnc2	Galnc1	Galnc2
12	RAD26	YJR035W	YPDcl2	YPDcl3	EtOHcl1	EtOHcl2	-	YPDnc1	YPDnc2	YPDnc3	YPDnc4	EtOHnc1	EtOHnc2	Galnc1	Galnc2
12	RPS6B	YBR181C	-	YPDcl3	EtOHcl1	EtOHcl2	Galcl1	YPDnc1	YPDnc2	YPDnc3	YPDnc4	EtOHnc1	EtOHnc2	Galnc1	Galnc2
12	PDR16	YNL231C	YPDcl2	YPDcl3	EtOHcl1	EtOHcl2	Galcl1	YPDnc1	YPDnc2	YPDnc3	-	EtOHnc1	EtOHnc2	Galnc1	Galnc2
12	GCD14	YJL125C	YPDcl2	-	EtOHcl1	EtOHcl2	Galcl1	YPDnc1	YPDnc2	YPDnc3	YPDnc4	EtOHnc1	EtOHnc2	Galnc1	Galnc2
12	SNU71	YGR013W	YPDcl2	YPDcl3	EtOHcl1	EtOHcl2	-	YPDnc1	YPDnc2	YPDnc3	YPDnc4	EtOHnc1	EtOHnc2	Galnc1	Galnc2
12	ARG80	YMR042W	YPDcl2	YPDcl3	EtOHcl1	EtOHcl2	Galcl1	YPDnc1	YPDnc2	YPDnc3	-	EtOHnc1	EtOHnc2	Galnc1	Galnc2
12	TES1	YJR019C	YPDcl2	-	EtOHcl1	EtOHcl2	Galcl1	YPDnc1	YPDnc2	YPDnc3	YPDnc4	EtOHnc1	EtOHnc2	Galnc1	Galnc2
12		YOR293C-A	YPDcl2	-	EtOHcl1	EtOHcl2	Galcl1	YPDnc1	YPDnc2	YPDnc3	YPDnc4	EtOHnc1	EtOHnc2	Galnc1	Galnc2
12	CAB1	YDR531W	YPDcl2	YPDcl3	EtOHcl1	EtOHcl2	Galcl1	YPDnc1	YPDnc2	YPDnc3	YPDnc4	EtOHnc1	EtOHnc2	Galnc1	-
12		YOL019W-A	YPDcl2	YPDcl3	EtOHcl1	EtOHcl2	Galcl1	YPDnc1	YPDnc2	YPDnc3	-	EtOHnc1	EtOHnc2	Galnc1	Galnc2
12		YDL068W	-	YPDcl3	EtOHcl1	EtOHcl2	Galcl1	YPDnc1	YPDnc2	YPDnc3	YPDnc4	EtOHnc1	EtOHnc2	Galnc1	Galnc2
12	MFB1	YDR219C	YPDcl2	YPDcl3	EtOHcl1	EtOHcl2	-	YPDnc1	YPDnc2	YPDnc3	YPDnc4	EtOHnc1	EtOHnc2	Galnc1	Galnc2
12		YIL141W	-	YPDcl3	EtOHcl1	EtOHcl2	Galcl1	YPDnc1	YPDnc2	YPDnc3	YPDnc4	EtOHnc1	EtOHnc2	Galnc1	Galnc2
12	BAT2	YJR148W	YPDcl2	-	EtOHcl1	EtOHcl2	Galcl1	YPDnc1	YPDnc2	YPDnc3	YPDnc4	EtOHnc1	EtOHnc2	Galnc1	Galnc2
12		YIL102C-A	YPDcl2	YPDcl3	EtOHcl1	-	Galcl1	YPDnc1	YPDnc2	YPDnc3	YPDnc4	EtOHnc1	EtOHnc2	Galnc1	Galnc2
12	ATG5	YPL149W	YPDcl2	-	EtOHcl1	EtOHcl2	Galcl1	YPDnc1	YPDnc2	YPDnc3	YPDnc4	EtOHnc1	EtOHnc2	Galnc1	Galnc2
12		YFL021C-A	YPDcl2	YPDcl3	-	EtOHcl2	Galcl1	YPDnc1	YPDnc2	YPDnc3	YPDnc4	EtOHnc1	EtOHnc2	Galnc1	Galnc2
12	GRS1	YBR121C	YPDcl2	-	EtOHcl1	EtOHcl2	Galcl1	YPDnc1	YPDnc2	YPDnc3	YPDnc4	EtOHnc1	EtOHnc2	Galnc1	Galnc2
12	GPB2	YAL056W	YPDcl2	YPDcl3	EtOHcl1	EtOHcl2	Galcl1	YPDnc1	YPDnc2	-	YPDnc4	EtOHnc1	EtOHnc2	Galnc1	Galnc2
12	TIR3	YIL011W	YPDcl2	YPDcl3	EtOHcl1	-	Galcl1	YPDnc1	YPDnc2	YPDnc3	YPDnc4	EtOHnc1	EtOHnc2	Galnc1	Galnc2
12	SOV1	YMR066W	-	YPDcl3	EtOHcl1	EtOHcl2	Galcl1	YPDnc1	YPDnc2	YPDnc3	YPDnc4	EtOHnc1	EtOHnc2	Galnc1	Galnc2
12	XDJ1	YLR090W	YPDcl2	YPDcl3	EtOHcl1	EtOHcl2	-	YPDnc1	YPDnc2	YPDnc3	YPDnc4	EtOHnc1	EtOHnc2	Galnc1	Galnc2
12	IES3	YLR052W	YPDcl2	YPDcl3	EtOHcl1	-	Galcl1	YPDnc1	YPDnc2	YPDnc3	YPDnc4	EtOHnc1	EtOHnc2	Galnc1	Galnc2

12	RAD57	YDR004W	YPDcl2	YPDcl3	EtOHcl1	EtOHcl2	Galcl1	YPDnc1	YPDnc2	-	YPDnc4	EtOHnc1	EtOHnc2	Galnc1	Galnc2
12	ERV15	YBR210W	YPDcl2	YPDcl3	EtOHcl1	EtOHcl2	Galcl1	YPDnc1	YPDnc2	YPDnc3	YPDnc4	EtOHnc1	EtOHnc2	Galnc1	-
12	FMP52	YER004W	-	YPDcl3	EtOHcl1	EtOHcl2	Galcl1	YPDnc1	YPDnc2	YPDnc3	YPDnc4	EtOHnc1	EtOHnc2	Galnc1	Galnc2
12	HOR7	YMR251W-	YPDcl2	YPDcl3	EtOHcl1	-	Galcl1	YPDnc1	YPDnc2	YPDnc3	YPDnc4	EtOHnc1	EtOHnc2	Galnc1	Galnc2
12	RPS3	YNL178W	YPDcl2	YPDcl3	EtOHcl1	-	Galcl1	YPDnc1	YPDnc2	YPDnc3	YPDnc4	EtOHnc1	EtOHnc2	Galnc1	Galnc2
12	KRE2	YDR483W	YPDcl2	YPDcl3	EtOHcl1	EtOHcl2	Galcl1	-	YPDnc2	YPDnc3	YPDnc4	EtOHnc1	EtOHnc2	Galnc1	Galnc2
12	NPP1	YCR026C	YPDcl2	YPDcl3	EtOHcl1	-	Galcl1	YPDnc1	YPDnc2	YPDnc3	YPDnc4	EtOHnc1	EtOHnc2	Galnc1	Galnc2
12		YGL218W	YPDcl2	YPDcl3	EtOHcl1	EtOHcl2	Galcl1	YPDnc1	YPDnc2	YPDnc3	-	EtOHnc1	EtOHnc2	Galnc1	Galnc2
12		YKR012C	YPDcl2	YPDcl3	EtOHcl1	EtOHcl2	-	YPDnc1	YPDnc2	YPDnc3	YPDnc4	EtOHnc1	EtOHnc2	Galnc1	Galnc2
12	NHP6A	YPR052C	-	YPDcl3	EtOHcl1	EtOHcl2	Galcl1	YPDnc1	YPDnc2	YPDnc3	YPDnc4	EtOHnc1	EtOHnc2	Galnc1	Galnc2
12		YGR219W	YPDcl2	-	EtOHcl1	EtOHcl2	Galcl1	YPDnc1	YPDnc2	YPDnc3	YPDnc4	EtOHnc1	EtOHnc2	Galnc1	Galnc2
12	SPC98	YNL126W	YPDcl2	YPDcl3	EtOHcl1	-	Galcl1	YPDnc1	YPDnc2	YPDnc3	YPDnc4	EtOHnc1	EtOHnc2	Galnc1	Galnc2
12	YAR1	YPL239W	-	YPDcl3	EtOHcl1	EtOHcl2	Galcl1	YPDnc1	YPDnc2	YPDnc3	YPDnc4	EtOHnc1	EtOHnc2	Galnc1	Galnc2
12	URA6	YKL024C	YPDcl2	YPDcl3	EtOHcl1	EtOHcl2	-	YPDnc1	YPDnc2	YPDnc3	YPDnc4	EtOHnc1	EtOHnc2	Galnc1	Galnc2
12	SPS22	YCL048W	YPDcl2	YPDcl3	EtOHcl1	-	Galcl1	YPDnc1	YPDnc2	YPDnc3	YPDnc4	EtOHnc1	EtOHnc2	Galnc1	Galnc2
12	COS9	YKL219W	YPDcl2	YPDcl3	EtOHcl1	-	Galcl1	YPDnc1	YPDnc2	YPDnc3	YPDnc4	EtOHnc1	EtOHnc2	Galnc1	Galnc2
12	CDC5	YMR001C	YPDcl2	YPDcl3	-	EtOHcl2	Galcl1	YPDnc1	YPDnc2	YPDnc3	YPDnc4	EtOHnc1	EtOHnc2	Galnc1	Galnc2
12	GDT1	YBR187W	YPDcl2	YPDcl3	EtOHcl1	EtOHcl2	Galcl1	-	YPDnc2	YPDnc3	YPDnc4	EtOHnc1	EtOHnc2	Galnc1	Galnc2
12		YCR097W-/	YPDcl2	-	EtOHcl1	EtOHcl2	Galcl1	YPDnc1	YPDnc2	YPDnc3	YPDnc4	EtOHnc1	EtOHnc2	Galnc1	Galnc2
12	HMO1	YDR174W	YPDcl2	YPDcl3	EtOHcl1	-	Galcl1	YPDnc1	YPDnc2	YPDnc3	YPDnc4	EtOHnc1	EtOHnc2	Galnc1	Galnc2
12	CGI121	YML036W	-	YPDcl3	EtOHcl1	EtOHcl2	Galcl1	YPDnc1	YPDnc2	YPDnc3	YPDnc4	EtOHnc1	EtOHnc2	Galnc1	Galnc2
12		YLR177W	YPDcl2	YPDcl3	-	EtOHcl2	Galcl1	YPDnc1	YPDnc2	YPDnc3	YPDnc4	EtOHnc1	EtOHnc2	Galnc1	Galnc2
12	PAP2	YOL115W	YPDcl2	YPDcl3	EtOHcl1	EtOHcl2	Galcl1	YPDnc1	YPDnc2	-	YPDnc4	EtOHnc1	EtOHnc2	Galnc1	Galnc2
12	TRE1	YPL176C	YPDcl2	YPDcl3	EtOHcl1	-	Galcl1	YPDnc1	YPDnc2	YPDnc3	YPDnc4	EtOHnc1	EtOHnc2	Galnc1	Galnc2
12	TRM82	YDR165W	YPDcl2	YPDcl3	EtOHcl1	EtOHcl2	Galcl1	YPDnc1	YPDnc2	YPDnc3	YPDnc4	-	EtOHnc2	Galnc1	Galnc2
12		YDR344C	YPDcl2	YPDcl3	EtOHcl1	EtOHcl2	Galcl1	-	YPDnc2	YPDnc3	YPDnc4	EtOHnc1	EtOHnc2	Galnc1	Galnc2
12	RMD9	YGL107C	-	YPDcl3	EtOHcl1	EtOHcl2	Galcl1	YPDnc1	YPDnc2	YPDnc3	YPDnc4	EtOHnc1	EtOHnc2	Galnc1	Galnc2
12	ATP23	YNR020C	-	YPDcl3	EtOHcl1	EtOHcl2	Galcl1	YPDnc1	YPDnc2	YPDnc3	YPDnc4	EtOHnc1	EtOHnc2	Galnc1	Galnc2
12		YFR016C	YPDcl2	YPDcl3	EtOHcl1	-	Galcl1	YPDnc1	YPDnc2	YPDnc3	YPDnc4	EtOHnc1	EtOHnc2	Galnc1	Galnc2
12	ADY2	YCR010C	-	YPDcl3	EtOHcl1	EtOHcl2	Galcl1	YPDnc1	YPDnc2	YPDnc3	YPDnc4	EtOHnc1	EtOHnc2	Galnc1	Galnc2
12	MSC6	YOR354C	YPDcl2	YPDcl3	EtOHcl1	EtOHcl2	-	YPDnc1	YPDnc2	YPDnc3	YPDnc4	EtOHnc1	EtOHnc2	Galnc1	Galnc2
12	ERG1	YGR175C	YPDcl2	YPDcl3	EtOHcl1	-	Galcl1	YPDnc1	YPDnc2	YPDnc3	YPDnc4	EtOHnc1	EtOHnc2	Galnc1	Galnc2
12	MCM5	YLR274W	YPDcl2	-	EtOHcl1	EtOHcl2	Galcl1	YPDnc1	YPDnc2	YPDnc3	YPDnc4	EtOHnc1	EtOHnc2	Galnc1	Galnc2
12	LAG1	YHL003C	YPDcl2	YPDcl3	EtOHcl1	EtOHcl2	Galcl1	YPDnc1	-	YPDnc3	YPDnc4	EtOHnc1	EtOHnc2	Galnc1	Galnc2

12	SDO1	YLR022C	YPDcl2	YPDcl3	EtOHcl1	-	Galcl1	YPDnc1	YPDnc2	YPDnc3	YPDnc4	EtOHnc1	EtOHnc2	Galnc1	Galnc2
12	NGL1	YOL042W	YPDcl2	-	EtOHcl1	EtOHcl2	Galcl1	YPDnc1	YPDnc2	YPDnc3	YPDnc4	EtOHnc1	EtOHnc2	Galnc1	Galnc2
12		YKL151C	YPDcl2	YPDcl3	EtOHcl1	EtOHcl2	Galcl1	YPDnc1	YPDnc2	YPDnc3	YPDnc4	EtOHnc1	-	Galnc1	Galnc2
12		YLR255C	YPDcl2	-	EtOHcl1	EtOHcl2	Galcl1	YPDnc1	YPDnc2	YPDnc3	YPDnc4	EtOHnc1	EtOHnc2	Galnc1	Galnc2
12	APS1	YLR170C	YPDcl2	-	EtOHcl1	EtOHcl2	Galcl1	YPDnc1	YPDnc2	YPDnc3	YPDnc4	EtOHnc1	EtOHnc2	Galnc1	Galnc2
12		YBR287W	YPDcl2	YPDcl3	EtOHcl1	EtOHcl2	Galcl1	-	YPDnc2	YPDnc3	YPDnc4	EtOHnc1	EtOHnc2	Galnc1	Galnc2
12	SLM3	YDL033C	YPDcl2	-	EtOHcl1	EtOHcl2	Galcl1	YPDnc1	YPDnc2	YPDnc3	YPDnc4	EtOHnc1	EtOHnc2	Galnc1	Galnc2
12		YML057C-A	-	YPDcl3	EtOHcl1	EtOHcl2	Galcl1	YPDnc1	YPDnc2	YPDnc3	YPDnc4	EtOHnc1	EtOHnc2	Galnc1	Galnc2
12		YGR153W	YPDcl2	-	EtOHcl1	EtOHcl2	Galcl1	YPDnc1	YPDnc2	YPDnc3	YPDnc4	EtOHnc1	EtOHnc2	Galnc1	Galnc2
12		YPL062W	YPDcl2	YPDcl3	EtOHcl1	EtOHcl2	Galcl1	YPDnc1	YPDnc2	YPDnc3	YPDnc4	EtOHnc1	-	Galnc1	Galnc2
12	MNN4	YKL201C	YPDcl2	YPDcl3	EtOHcl1	-	Galcl1	YPDnc1	YPDnc2	YPDnc3	YPDnc4	EtOHnc1	EtOHnc2	Galnc1	Galnc2
12		YNL198C	YPDcl2	YPDcl3	EtOHcl1	-	Galcl1	YPDnc1	YPDnc2	YPDnc3	YPDnc4	EtOHnc1	EtOHnc2	Galnc1	Galnc2
12	HFI1	YPL254W	YPDcl2	YPDcl3	EtOHcl1	EtOHcl2	-	YPDnc1	YPDnc2	YPDnc3	YPDnc4	EtOHnc1	EtOHnc2	Galnc1	Galnc2
12	AIM37	YNL100W	YPDcl2	YPDcl3	EtOHcl1	EtOHcl2	Galcl1	YPDnc1	YPDnc2	YPDnc3	YPDnc4	EtOHnc1	-	Galnc1	Galnc2
12		YOL114C	-	YPDcl3	EtOHcl1	EtOHcl2	Galcl1	YPDnc1	YPDnc2	YPDnc3	YPDnc4	EtOHnc1	EtOHnc2	Galnc1	Galnc2
12	SKN1	YGR143W	YPDcl2	-	EtOHcl1	EtOHcl2	Galcl1	YPDnc1	YPDnc2	YPDnc3	YPDnc4	EtOHnc1	EtOHnc2	Galnc1	Galnc2
12	WTM2	YOR229W	-	YPDcl3	EtOHcl1	EtOHcl2	Galcl1	YPDnc1	YPDnc2	YPDnc3	YPDnc4	EtOHnc1	EtOHnc2	Galnc1	Galnc2
12	PUF6	YDR496C	YPDcl2	YPDcl3	EtOHcl1	-	Galcl1	YPDnc1	YPDnc2	YPDnc3	YPDnc4	EtOHnc1	EtOHnc2	Galnc1	Galnc2
12		YBL070C	YPDcl2	YPDcl3	EtOHcl1	EtOHcl2	-	YPDnc1	YPDnc2	YPDnc3	YPDnc4	EtOHnc1	EtOHnc2	Galnc1	Galnc2
12		YOR161W-	YPDcl2	-	EtOHcl1	EtOHcl2	Galcl1	YPDnc1	YPDnc2	YPDnc3	YPDnc4	EtOHnc1	EtOHnc2	Galnc1	Galnc2
12	ALT2	YDR111C	YPDcl2	-	EtOHcl1	EtOHcl2	Galcl1	YPDnc1	YPDnc2	YPDnc3	YPDnc4	EtOHnc1	EtOHnc2	Galnc1	Galnc2
12	MRPL16	YBL038W	YPDcl2	YPDcl3	-	EtOHcl2	Galcl1	YPDnc1	YPDnc2	YPDnc3	YPDnc4	EtOHnc1	EtOHnc2	Galnc1	Galnc2
12		YLR422W	YPDcl2	-	EtOHcl1	EtOHcl2	Galcl1	YPDnc1	YPDnc2	YPDnc3	YPDnc4	EtOHnc1	EtOHnc2	Galnc1	Galnc2
12	PAU2	YEL049W	YPDcl2	YPDcl3	EtOHcl1	EtOHcl2	Galcl1	YPDnc1	YPDnc2	YPDnc3	YPDnc4	EtOHnc1	EtOHnc2	Galnc1	-
12	REV3	YPL167C	YPDcl2	YPDcl3	EtOHcl1	-	Galcl1	YPDnc1	YPDnc2	YPDnc3	YPDnc4	EtOHnc1	EtOHnc2	Galnc1	Galnc2
12	EDS1	YBR033W	YPDcl2	YPDcl3	EtOHcl1	EtOHcl2	Galcl1	YPDnc1	YPDnc2	YPDnc3	-	EtOHnc1	EtOHnc2	Galnc1	Galnc2
12	UIP4	YPL186C	YPDcl2	YPDcl3	EtOHcl1	-	Galcl1	YPDnc1	YPDnc2	YPDnc3	YPDnc4	EtOHnc1	EtOHnc2	Galnc1	Galnc2
12	PHO84	YML123C	-	YPDcl3	EtOHcl1	EtOHcl2	Galcl1	YPDnc1	YPDnc2	YPDnc3	YPDnc4	EtOHnc1	EtOHnc2	Galnc1	Galnc2
12		YDL206W	YPDcl2	YPDcl3	-	EtOHcl2	Galcl1	YPDnc1	YPDnc2	YPDnc3	YPDnc4	EtOHnc1	EtOHnc2	Galnc1	Galnc2
12	UBP1	YDL122W	YPDcl2	YPDcl3	EtOHcl1	EtOHcl2	Galcl1	YPDnc1	YPDnc2	YPDnc3	YPDnc4	EtOHnc1	EtOHnc2	Galnc1	-
12	RTC3	YHR087W	-	YPDcl3	EtOHcl1	EtOHcl2	Galcl1	YPDnc1	YPDnc2	YPDnc3	YPDnc4	EtOHnc1	EtOHnc2	Galnc1	Galnc2
12	CTH1	YDR151C	YPDcl2	YPDcl3	EtOHcl1	EtOHcl2	Galcl1	YPDnc1	-	YPDnc3	YPDnc4	EtOHnc1	EtOHnc2	Galnc1	Galnc2
12	TDA5	YLR426W	YPDcl2	YPDcl3	EtOHcl1	-	Galcl1	YPDnc1	YPDnc2	YPDnc3	YPDnc4	EtOHnc1	EtOHnc2	Galnc1	Galnc2
12	NUP2	YLR335W	YPDcl2	YPDcl3	-	EtOHcl2	Galcl1	YPDnc1	YPDnc2	YPDnc3	YPDnc4	EtOHnc1	EtOHnc2	Galnc1	Galnc2

12	HSP104	YLL026W	YPDcl2	YPDcl3	EtOHcl1	EtOHcl2	Galcl1	YPDnc1	-	YPDnc3	YPDnc4	EtOHnc1	EtOHnc2	Galnc1	Galnc2
12	RIT1	YMR283C	YPDcl2	YPDcl3	EtOHcl1	EtOHcl2	Galcl1	-	YPDnc2	YPDnc3	YPDnc4	EtOHnc1	EtOHnc2	Galnc1	Galnc2
12		YBR137W	YPDcl2	YPDcl3	EtOHcl1	EtOHcl2	Galcl1	YPDnc1	YPDnc2	YPDnc3	YPDnc4	EtOHnc1	-	Galnc1	Galnc2
12		YML100W-	YPDcl2	YPDcl3	EtOHcl1	EtOHcl2	Galcl1	YPDnc1	YPDnc2	YPDnc3	YPDnc4	EtOHnc1	-	Galnc1	Galnc2
12	COX17	YLL009C	YPDcl2	-	EtOHcl1	EtOHcl2	Galcl1	YPDnc1	YPDnc2	YPDnc3	YPDnc4	EtOHnc1	EtOHnc2	Galnc1	Galnc2
12	TMN2	YDR107C	YPDcl2	YPDcl3	EtOHcl1	-	Galcl1	YPDnc1	YPDnc2	YPDnc3	YPDnc4	EtOHnc1	EtOHnc2	Galnc1	Galnc2
12	MRH4	YGL064C	YPDcl2	-	EtOHcl1	EtOHcl2	Galcl1	YPDnc1	YPDnc2	YPDnc3	YPDnc4	EtOHnc1	EtOHnc2	Galnc1	Galnc2
12	RKI1	YOR095C	YPDcl2	YPDcl3	EtOHcl1	-	Galcl1	YPDnc1	YPDnc2	YPDnc3	YPDnc4	EtOHnc1	EtOHnc2	Galnc1	Galnc2
12	HXT9	YJL219W	YPDcl2	-	EtOHcl1	EtOHcl2	Galcl1	YPDnc1	YPDnc2	YPDnc3	YPDnc4	EtOHnc1	EtOHnc2	Galnc1	Galnc2
12		YJL193W	YPDcl2	YPDcl3	EtOHcl1	-	Galcl1	YPDnc1	YPDnc2	YPDnc3	YPDnc4	EtOHnc1	EtOHnc2	Galnc1	Galnc2
12		YAL063C-A	YPDcl2	YPDcl3	EtOHcl1	EtOHcl2	Galcl1	YPDnc1	YPDnc2	YPDnc3	YPDnc4	-	EtOHnc2	Galnc1	Galnc2
12	SAC7	YDR389W	YPDcl2	YPDcl3	EtOHcl1	-	Galcl1	YPDnc1	YPDnc2	YPDnc3	YPDnc4	EtOHnc1	EtOHnc2	Galnc1	Galnc2
12	SNF1	YDR477W	YPDcl2	-	EtOHcl1	EtOHcl2	Galcl1	YPDnc1	YPDnc2	YPDnc3	YPDnc4	EtOHnc1	EtOHnc2	Galnc1	Galnc2
12		YER076W-/	YPDcl2	YPDcl3	EtOHcl1	EtOHcl2	Galcl1	YPDnc1	YPDnc2	YPDnc3	YPDnc4	-	EtOHnc2	Galnc1	Galnc2
12	DDC1	YPL194W	YPDcl2	YPDcl3	EtOHcl1	EtOHcl2	Galcl1	YPDnc1	YPDnc2	YPDnc3	YPDnc4	-	EtOHnc2	Galnc1	Galnc2
12		YDL026W	YPDcl2	YPDcl3	EtOHcl1	EtOHcl2	Galcl1	YPDnc1	YPDnc2	YPDnc3	YPDnc4	EtOHnc1	-	Galnc1	Galnc2
12		YMR245W	YPDcl2	YPDcl3	EtOHcl1	EtOHcl2	-	YPDnc1	YPDnc2	YPDnc3	YPDnc4	EtOHnc1	EtOHnc2	Galnc1	Galnc2
12		YPL068C	YPDcl2	YPDcl3	EtOHcl1	-	Galcl1	YPDnc1	YPDnc2	YPDnc3	YPDnc4	EtOHnc1	EtOHnc2	Galnc1	Galnc2
12	HUL4	YJR036C	YPDcl2	-	EtOHcl1	EtOHcl2	Galcl1	YPDnc1	YPDnc2	YPDnc3	YPDnc4	EtOHnc1	EtOHnc2	Galnc1	Galnc2
12	HPR1	YDR138W	YPDcl2	YPDcl3	EtOHcl1	EtOHcl2	Galcl1	-	YPDnc2	YPDnc3	YPDnc4	EtOHnc1	EtOHnc2	Galnc1	Galnc2
12		YNL179C	YPDcl2	YPDcl3	EtOHcl1	EtOHcl2	-	YPDnc1	YPDnc2	YPDnc3	YPDnc4	EtOHnc1	EtOHnc2	Galnc1	Galnc2
12	TEX1	YNL253W	-	YPDcl3	EtOHcl1	EtOHcl2	Galcl1	YPDnc1	YPDnc2	YPDnc3	YPDnc4	EtOHnc1	EtOHnc2	Galnc1	Galnc2
12	HIR3	YJR140C	YPDcl2	YPDcl3	EtOHcl1	EtOHcl2	Galcl1	-	YPDnc2	YPDnc3	YPDnc4	EtOHnc1	EtOHnc2	Galnc1	Galnc2
12	ROD1	YOR018W	YPDcl2	YPDcl3	EtOHcl1	EtOHcl2	Galcl1	YPDnc1	YPDnc2	YPDnc3	YPDnc4	EtOHnc1	-	Galnc1	Galnc2
12	CIN1	YOR349W	YPDcl2	YPDcl3	EtOHcl1	-	Galcl1	YPDnc1	YPDnc2	YPDnc3	YPDnc4	EtOHnc1	EtOHnc2	Galnc1	Galnc2
12	PEX7	YDR142C	-	YPDcl3	EtOHcl1	EtOHcl2	Galcl1	YPDnc1	YPDnc2	YPDnc3	YPDnc4	EtOHnc1	EtOHnc2	Galnc1	Galnc2
12	EHD3	YDR036C	-	YPDcl3	EtOHcl1	EtOHcl2	Galcl1	YPDnc1	YPDnc2	YPDnc3	YPDnc4	EtOHnc1	EtOHnc2	Galnc1	Galnc2
12	ECM27	YJR106W	-	YPDcl3	EtOHcl1	EtOHcl2	Galcl1	YPDnc1	YPDnc2	YPDnc3	YPDnc4	EtOHnc1	EtOHnc2	Galnc1	Galnc2
12	OAC1	YKL120W	YPDcl2	YPDcl3	EtOHcl1	EtOHcl2	-	YPDnc1	YPDnc2	YPDnc3	YPDnc4	EtOHnc1	EtOHnc2	Galnc1	Galnc2
12	SEC16	YPL085W	YPDcl2	YPDcl3	EtOHcl1	EtOHcl2	Galcl1	-	YPDnc2	YPDnc3	YPDnc4	EtOHnc1	EtOHnc2	Galnc1	Galnc2
12	MET3	YJR010W	YPDcl2	YPDcl3	-	EtOHcl2	Galcl1	YPDnc1	YPDnc2	YPDnc3	YPDnc4	EtOHnc1	EtOHnc2	Galnc1	Galnc2
12		YPR053C	YPDcl2	YPDcl3	EtOHcl1	-	Galcl1	YPDnc1	YPDnc2	YPDnc3	YPDnc4	EtOHnc1	EtOHnc2	Galnc1	Galnc2
12	DOM34	YNL001W	YPDcl2	YPDcl3	EtOHcl1	-	Galcl1	YPDnc1	YPDnc2	YPDnc3	YPDnc4	EtOHnc1	EtOHnc2	Galnc1	Galnc2
12	STE20	YHL007C	YPDcl2	YPDcl3	EtOHcl1	-	Galcl1	YPDnc1	YPDnc2	YPDnc3	YPDnc4	EtOHnc1	EtOHnc2	Galnc1	Galnc2

12	TPS3	YMR261C	YPDcl2	-	EtOHcl1	EtOHcl2	Galcl1	YPDnc1	YPDnc2	YPDnc3	YPDnc4	EtOHnc1	EtOHnc2	Galnc1	Galnc2
12	CHD1	YER164W	-	YPDcl3	EtOHcl1	EtOHcl2	Galcl1	YPDnc1	YPDnc2	YPDnc3	YPDnc4	EtOHnc1	EtOHnc2	Galnc1	Galnc2
12	MCH5	YOR306C	YPDcl2	YPDcl3	EtOHcl1	EtOHcl2	Galcl1	YPDnc1	YPDnc2	YPDnc3	-	EtOHnc1	EtOHnc2	Galnc1	Galnc2
12	CRR1	YLR213C	YPDcl2	YPDcl3	EtOHcl1	-	Galcl1	YPDnc1	YPDnc2	YPDnc3	YPDnc4	EtOHnc1	EtOHnc2	Galnc1	Galnc2
12	SPC1	YJR010C-A	YPDcl2	YPDcl3	EtOHcl1	EtOHcl2	Galcl1	-	YPDnc2	YPDnc3	YPDnc4	EtOHnc1	EtOHnc2	Galnc1	Galnc2
12	ISA2	YPR067W	YPDcl2	YPDcl3	EtOHcl1	EtOHcl2	Galcl1	-	YPDnc2	YPDnc3	YPDnc4	EtOHnc1	EtOHnc2	Galnc1	Galnc2
12	YRM1	YOR172W	YPDcl2	YPDcl3	EtOHcl1	EtOHcl2	Galcl1	YPDnc1	YPDnc2	YPDnc3	YPDnc4	EtOHnc1	-	Galnc1	Galnc2
12		YOL160W	YPDcl2	-	EtOHcl1	EtOHcl2	Galcl1	YPDnc1	YPDnc2	YPDnc3	YPDnc4	EtOHnc1	EtOHnc2	Galnc1	Galnc2
12		YKR075C	-	YPDcl3	EtOHcl1	EtOHcl2	Galcl1	YPDnc1	YPDnc2	YPDnc3	YPDnc4	EtOHnc1	EtOHnc2	Galnc1	Galnc2
12	MFT1	YML062C	YPDcl2	YPDcl3	-	EtOHcl2	Galcl1	YPDnc1	YPDnc2	YPDnc3	YPDnc4	EtOHnc1	EtOHnc2	Galnc1	Galnc2
12	GSH1	YJL101C	-	YPDcl3	EtOHcl1	EtOHcl2	Galcl1	YPDnc1	YPDnc2	YPDnc3	YPDnc4	EtOHnc1	EtOHnc2	Galnc1	Galnc2
12	SWI5	YDR146C	YPDcl2	YPDcl3	EtOHcl1	EtOHcl2	Galcl1	YPDnc1	YPDnc2	YPDnc3	YPDnc4	EtOHnc1	-	Galnc1	Galnc2
12		YJR037W	YPDcl2	-	EtOHcl1	EtOHcl2	Galcl1	YPDnc1	YPDnc2	YPDnc3	YPDnc4	EtOHnc1	EtOHnc2	Galnc1	Galnc2
12	CPD1	YGR247W	YPDcl2	YPDcl3	EtOHcl1	EtOHcl2	Galcl1	YPDnc1	YPDnc2	YPDnc3	-	EtOHnc1	EtOHnc2	Galnc1	Galnc2
12	SBE2	YDR351W	YPDcl2	YPDcl3	EtOHcl1	EtOHcl2	Galcl1	YPDnc1	YPDnc2	YPDnc3	YPDnc4	EtOHnc1	-	Galnc1	Galnc2
12		YHL008C	YPDcl2	YPDcl3	EtOHcl1	EtOHcl2	-	YPDnc1	YPDnc2	YPDnc3	YPDnc4	EtOHnc1	EtOHnc2	Galnc1	Galnc2
12	AVT3	YKL146W	YPDcl2	-	EtOHcl1	EtOHcl2	Galcl1	YPDnc1	YPDnc2	YPDnc3	YPDnc4	EtOHnc1	EtOHnc2	Galnc1	Galnc2
12	NFI1	YOR156C	YPDcl2	YPDcl3	EtOHcl1	-	Galcl1	YPDnc1	YPDnc2	YPDnc3	YPDnc4	EtOHnc1	EtOHnc2	Galnc1	Galnc2
12	CTF3	YLR381W	YPDcl2	YPDcl3	EtOHcl1	-	Galcl1	YPDnc1	YPDnc2	YPDnc3	YPDnc4	EtOHnc1	EtOHnc2	Galnc1	Galnc2
12		YBL028C	YPDcl2	YPDcl3	EtOHcl1	EtOHcl2	Galcl1	-	YPDnc2	YPDnc3	YPDnc4	EtOHnc1	EtOHnc2	Galnc1	Galnc2
12	CDC73	YLR418C	YPDcl2	YPDcl3	-	EtOHcl2	Galcl1	YPDnc1	YPDnc2	YPDnc3	YPDnc4	EtOHnc1	EtOHnc2	Galnc1	Galnc2
12	PFK2	YMR205C	YPDcl2	YPDcl3	EtOHcl1	EtOHcl2	-	YPDnc1	YPDnc2	YPDnc3	YPDnc4	EtOHnc1	EtOHnc2	Galnc1	Galnc2
12		YJL118W	-	YPDcl3	EtOHcl1	EtOHcl2	Galcl1	YPDnc1	YPDnc2	YPDnc3	YPDnc4	EtOHnc1	EtOHnc2	Galnc1	Galnc2
12	GSP2	YOR185C	YPDcl2	YPDcl3	EtOHcl1	-	Galcl1	YPDnc1	YPDnc2	YPDnc3	YPDnc4	EtOHnc1	EtOHnc2	Galnc1	Galnc2
12	RPE1	YJL121C	YPDcl2	YPDcl3	EtOHcl1	-	Galcl1	YPDnc1	YPDnc2	YPDnc3	YPDnc4	EtOHnc1	EtOHnc2	Galnc1	Galnc2
12		YGL114W	YPDcl2	YPDcl3	-	EtOHcl2	Galcl1	YPDnc1	YPDnc2	YPDnc3	YPDnc4	EtOHnc1	EtOHnc2	Galnc1	Galnc2
12	EMC4	YGL231C	YPDcl2	YPDcl3	EtOHcl1	EtOHcl2	Galcl1	YPDnc1	YPDnc2	YPDnc3	YPDnc4	-	EtOHnc2	Galnc1	Galnc2
12		YDL133W	YPDcl2	-	EtOHcl1	EtOHcl2	Galcl1	YPDnc1	YPDnc2	YPDnc3	YPDnc4	EtOHnc1	EtOHnc2	Galnc1	Galnc2
12	GPA1	YHR005C	YPDcl2	YPDcl3	EtOHcl1	EtOHcl2	Galcl1	YPDnc1	-	YPDnc3	YPDnc4	EtOHnc1	EtOHnc2	Galnc1	Galnc2
12		YLR281C	YPDcl2	YPDcl3	EtOHcl1	-	Galcl1	YPDnc1	YPDnc2	YPDnc3	YPDnc4	EtOHnc1	EtOHnc2	Galnc1	Galnc2
12	PRO3	YER023W	YPDcl2	YPDcl3	EtOHcl1	EtOHcl2	Galcl1	YPDnc1	YPDnc2	YPDnc3	YPDnc4	EtOHnc1	EtOHnc2	Galnc1	-
12		YGR039W	YPDcl2	YPDcl3	EtOHcl1	-	Galcl1	YPDnc1	YPDnc2	YPDnc3	YPDnc4	EtOHnc1	EtOHnc2	Galnc1	Galnc2
12		YOR161W-	YPDcl2	-	EtOHcl1	EtOHcl2	Galcl1	YPDnc1	YPDnc2	YPDnc3	YPDnc4	EtOHnc1	EtOHnc2	Galnc1	Galnc2
12	SNL1	YIL016W	-	YPDcl3	EtOHcl1	EtOHcl2	Galcl1	YPDnc1	YPDnc2	YPDnc3	YPDnc4	EtOHnc1	EtOHnc2	Galnc1	Galnc2

12	RSC9	YML127W	-	YPDcl3	EtOHcl1	EtOHcl2	Galcl1	YPDnc1	YPDnc2	YPDnc3	YPDnc4	EtOHnc1	EtOHnc2	Galnc1	Galnc2
12	POP1	YNL221C	-	YPDcl3	EtOHcl1	EtOHcl2	Galcl1	YPDnc1	YPDnc2	YPDnc3	YPDnc4	EtOHnc1	EtOHnc2	Galnc1	Galnc2
12		YJR098C	YPDcl2	YPDcl3	-	EtOHcl2	Galcl1	YPDnc1	YPDnc2	YPDnc3	YPDnc4	EtOHnc1	EtOHnc2	Galnc1	Galnc2
12	JSN1	YJR091C	-	YPDcl3	EtOHcl1	EtOHcl2	Galcl1	YPDnc1	YPDnc2	YPDnc3	YPDnc4	EtOHnc1	EtOHnc2	Galnc1	Galnc2
12		YML007C-A	YPDcl2	YPDcl3	-	EtOHcl2	Galcl1	YPDnc1	YPDnc2	YPDnc3	YPDnc4	EtOHnc1	EtOHnc2	Galnc1	Galnc2
12		YNL140C	YPDcl2	-	EtOHcl1	EtOHcl2	Galcl1	YPDnc1	YPDnc2	YPDnc3	YPDnc4	EtOHnc1	EtOHnc2	Galnc1	Galnc2
12		YMR160W	-	YPDcl3	EtOHcl1	EtOHcl2	Galcl1	YPDnc1	YPDnc2	YPDnc3	YPDnc4	EtOHnc1	EtOHnc2	Galnc1	Galnc2
12	MDR1	YGR100W	YPDcl2	YPDcl3	EtOHcl1	-	Galcl1	YPDnc1	YPDnc2	YPDnc3	YPDnc4	EtOHnc1	EtOHnc2	Galnc1	Galnc2
12	MXR1	YER042W	YPDcl2	YPDcl3	EtOHcl1	EtOHcl2	Galcl1	YPDnc1	-	YPDnc3	YPDnc4	EtOHnc1	EtOHnc2	Galnc1	Galnc2
12	GDE1	YPL110C	YPDcl2	YPDcl3	EtOHcl1	EtOHcl2	Galcl1	YPDnc1	YPDnc2	-	YPDnc4	EtOHnc1	EtOHnc2	Galnc1	Galnc2
12	FPS1	YLL043W	YPDcl2	YPDcl3	EtOHcl1	-	Galcl1	YPDnc1	YPDnc2	YPDnc3	YPDnc4	EtOHnc1	EtOHnc2	Galnc1	Galnc2
12		YPR195C	YPDcl2	YPDcl3	EtOHcl1	-	Galcl1	YPDnc1	YPDnc2	YPDnc3	YPDnc4	EtOHnc1	EtOHnc2	Galnc1	Galnc2
12	BIT61	YJL058C	YPDcl2	YPDcl3	EtOHcl1	-	Galcl1	YPDnc1	YPDnc2	YPDnc3	YPDnc4	EtOHnc1	EtOHnc2	Galnc1	Galnc2
12	SWR1	YDR334W	YPDcl2	YPDcl3	EtOHcl1	-	Galcl1	YPDnc1	YPDnc2	YPDnc3	YPDnc4	EtOHnc1	EtOHnc2	Galnc1	Galnc2
12	DIS3	YOL021C	YPDcl2	YPDcl3	EtOHcl1	EtOHcl2	Galcl1	YPDnc1	YPDnc2	YPDnc3	-	EtOHnc1	EtOHnc2	Galnc1	Galnc2
12	SPO77	YLR341W	-	YPDcl3	EtOHcl1	EtOHcl2	Galcl1	YPDnc1	YPDnc2	YPDnc3	YPDnc4	EtOHnc1	EtOHnc2	Galnc1	Galnc2
12	DEM1	YBR163W	YPDcl2	YPDcl3	EtOHcl1	EtOHcl2	Galcl1	YPDnc1	YPDnc2	YPDnc3	-	EtOHnc1	EtOHnc2	Galnc1	Galnc2
12	FIG2	YCR089W	YPDcl2	YPDcl3	EtOHcl1	EtOHcl2	Galcl1	YPDnc1	YPDnc2	YPDnc3	YPDnc4	EtOHnc1	-	Galnc1	Galnc2
12		YKL065W-A	YPDcl2	YPDcl3	EtOHcl1	EtOHcl2	Galcl1	-	YPDnc2	YPDnc3	YPDnc4	EtOHnc1	EtOHnc2	Galnc1	Galnc2
12	PPS1	YBR276C	YPDcl2	YPDcl3	EtOHcl1	EtOHcl2	-	YPDnc1	YPDnc2	YPDnc3	YPDnc4	EtOHnc1	EtOHnc2	Galnc1	Galnc2
12	PEX15	YOL044W	YPDcl2	YPDcl3	EtOHcl1	EtOHcl2	Galcl1	YPDnc1	-	YPDnc3	YPDnc4	EtOHnc1	EtOHnc2	Galnc1	Galnc2
12	CCR4	YAL021C	-	YPDcl3	EtOHcl1	EtOHcl2	Galcl1	YPDnc1	YPDnc2	YPDnc3	YPDnc4	EtOHnc1	EtOHnc2	Galnc1	Galnc2
12	SLY1	YDR189W	-	YPDcl3	EtOHcl1	EtOHcl2	Galcl1	YPDnc1	YPDnc2	YPDnc3	YPDnc4	EtOHnc1	EtOHnc2	Galnc1	Galnc2
12	DRN1	YGR093W	-	YPDcl3	EtOHcl1	EtOHcl2	Galcl1	YPDnc1	YPDnc2	YPDnc3	YPDnc4	EtOHnc1	EtOHnc2	Galnc1	Galnc2
12	TOM5	YPR133W-A	YPDcl2	YPDcl3	EtOHcl1	EtOHcl2	Galcl1	YPDnc1	YPDnc2	YPDnc3	YPDnc4	-	EtOHnc2	Galnc1	Galnc2
12		YDR008C	YPDcl2	YPDcl3	EtOHcl1	-	Galcl1	YPDnc1	YPDnc2	YPDnc3	YPDnc4	EtOHnc1	EtOHnc2	Galnc1	Galnc2
12	IDS2	YJL146W	YPDcl2	YPDcl3	EtOHcl1	EtOHcl2	Galcl1	YPDnc1	-	YPDnc3	YPDnc4	EtOHnc1	EtOHnc2	Galnc1	Galnc2
12		YOR012W	YPDcl2	YPDcl3	-	EtOHcl2	Galcl1	YPDnc1	YPDnc2	YPDnc3	YPDnc4	EtOHnc1	EtOHnc2	Galnc1	Galnc2
12		YMR244C-A	YPDcl2	YPDcl3	EtOHcl1	EtOHcl2	-	YPDnc1	YPDnc2	YPDnc3	YPDnc4	EtOHnc1	EtOHnc2	Galnc1	Galnc2
12	SIP18	YMR175W	YPDcl2	YPDcl3	EtOHcl1	-	Galcl1	YPDnc1	YPDnc2	YPDnc3	YPDnc4	EtOHnc1	EtOHnc2	Galnc1	Galnc2
12	NMD3	YHR170W	YPDcl2	YPDcl3	EtOHcl1	-	Galcl1	YPDnc1	YPDnc2	YPDnc3	YPDnc4	EtOHnc1	EtOHnc2	Galnc1	Galnc2
12	HBT1	YDL223C	YPDcl2	YPDcl3	EtOHcl1	EtOHcl2	Galcl1	YPDnc1	YPDnc2	YPDnc3	YPDnc4	EtOHnc1	-	Galnc1	Galnc2
12		YDR338C	YPDcl2	-	EtOHcl1	EtOHcl2	Galcl1	YPDnc1	YPDnc2	YPDnc3	YPDnc4	EtOHnc1	EtOHnc2	Galnc1	Galnc2
12	MLP1	YKR095W	YPDcl2	YPDcl3	-	EtOHcl2	Galcl1	YPDnc1	YPDnc2	YPDnc3	YPDnc4	EtOHnc1	EtOHnc2	Galnc1	Galnc2

12	STM1	YLR150W	YPDcl2	YPDcl3	EtOHcl1	-	Galcl1	YPDnc1	YPDnc2	YPDnc3	YPDnc4	EtOHnc1	EtOHnc2	Galnc1	Galnc2
12	REI1	YBR267W	YPDcl2	YPDcl3	EtOHcl1	-	Galcl1	YPDnc1	YPDnc2	YPDnc3	YPDnc4	EtOHnc1	EtOHnc2	Galnc1	Galnc2
12	LCL1	YPL056C	YPDcl2	YPDcl3	EtOHcl1	EtOHcl2	Galcl1	YPDnc1	-	YPDnc3	YPDnc4	EtOHnc1	EtOHnc2	Galnc1	Galnc2
12	TCO89	YPL180W	YPDcl2	YPDcl3	EtOHcl1	-	Galcl1	YPDnc1	YPDnc2	YPDnc3	YPDnc4	EtOHnc1	EtOHnc2	Galnc1	Galnc2
12	MPD1	YOR288C	YPDcl2	YPDcl3	-	EtOHcl2	Galcl1	YPDnc1	YPDnc2	YPDnc3	YPDnc4	EtOHnc1	EtOHnc2	Galnc1	Galnc2
12		YPR148C	YPDcl2	-	EtOHcl1	EtOHcl2	Galcl1	YPDnc1	YPDnc2	YPDnc3	YPDnc4	EtOHnc1	EtOHnc2	Galnc1	Galnc2
12	MYO5	YMR109W	YPDcl2	YPDcl3	EtOHcl1	EtOHcl2	Galcl1	YPDnc1	YPDnc2	YPDnc3	YPDnc4	EtOHnc1	-	Galnc1	Galnc2
12	GRE2	YOL151W	YPDcl2	YPDcl3	EtOHcl1	EtOHcl2	Galcl1	YPDnc1	YPDnc2	YPDnc3	YPDnc4	EtOHnc1	-	Galnc1	Galnc2
12	FPR2	YDR519W	-	YPDcl3	EtOHcl1	EtOHcl2	Galcl1	YPDnc1	YPDnc2	YPDnc3	YPDnc4	EtOHnc1	EtOHnc2	Galnc1	Galnc2
12	POL32	YJR043C	YPDcl2	YPDcl3	EtOHcl1	EtOHcl2	Galcl1	YPDnc1	YPDnc2	YPDnc3	YPDnc4	EtOHnc1	-	Galnc1	Galnc2
12	TRS31	YDR472W	YPDcl2	YPDcl3	EtOHcl1	-	Galcl1	YPDnc1	YPDnc2	YPDnc3	YPDnc4	EtOHnc1	EtOHnc2	Galnc1	Galnc2
12	GCR2	YNL199C	YPDcl2	YPDcl3	EtOHcl1	EtOHcl2	Galcl1	YPDnc1	YPDnc2	YPDnc3	-	EtOHnc1	EtOHnc2	Galnc1	Galnc2
12		YPL260W	YPDcl2	YPDcl3	EtOHcl1	-	Galcl1	YPDnc1	YPDnc2	YPDnc3	YPDnc4	EtOHnc1	EtOHnc2	Galnc1	Galnc2
12	STB4	YMR019W	YPDcl2	YPDcl3	EtOHcl1	-	Galcl1	YPDnc1	YPDnc2	YPDnc3	YPDnc4	EtOHnc1	EtOHnc2	Galnc1	Galnc2
12	DTR1	YBR180W	-	YPDcl3	EtOHcl1	EtOHcl2	Galcl1	YPDnc1	YPDnc2	YPDnc3	YPDnc4	EtOHnc1	EtOHnc2	Galnc1	Galnc2
12		YER187W	YPDcl2	YPDcl3	EtOHcl1	EtOHcl2	-	YPDnc1	YPDnc2	YPDnc3	YPDnc4	EtOHnc1	EtOHnc2	Galnc1	Galnc2
12	OXA1	YER154W	-	YPDcl3	EtOHcl1	EtOHcl2	Galcl1	YPDnc1	YPDnc2	YPDnc3	YPDnc4	EtOHnc1	EtOHnc2	Galnc1	Galnc2
12	DFR1	YOR236W	-	YPDcl3	EtOHcl1	EtOHcl2	Galcl1	YPDnc1	YPDnc2	YPDnc3	YPDnc4	EtOHnc1	EtOHnc2	Galnc1	Galnc2
12	TOF1	YNL273W	-	YPDcl3	EtOHcl1	EtOHcl2	Galcl1	YPDnc1	YPDnc2	YPDnc3	YPDnc4	EtOHnc1	EtOHnc2	Galnc1	Galnc2
12	PTC7	YHR076W	YPDcl2	YPDcl3	EtOHcl1	EtOHcl2	Galcl1	YPDnc1	YPDnc2	YPDnc3	-	EtOHnc1	EtOHnc2	Galnc1	Galnc2
12	NRT1	YOR071C	-	YPDcl3	EtOHcl1	EtOHcl2	Galcl1	YPDnc1	YPDnc2	YPDnc3	YPDnc4	EtOHnc1	EtOHnc2	Galnc1	Galnc2
12	GCN5	YGR252W	YPDcl2	YPDcl3	EtOHcl1	EtOHcl2	Galcl1	YPDnc1	YPDnc2	YPDnc3	YPDnc4	-	EtOHnc2	Galnc1	Galnc2
12	LTP1	YPR073C	-	YPDcl3	EtOHcl1	EtOHcl2	Galcl1	YPDnc1	YPDnc2	YPDnc3	YPDnc4	EtOHnc1	EtOHnc2	Galnc1	Galnc2
12	TAF5	YBR198C	YPDcl2	YPDcl3	EtOHcl1	-	Galcl1	YPDnc1	YPDnc2	YPDnc3	YPDnc4	EtOHnc1	EtOHnc2	Galnc1	Galnc2
12		YPL034W	YPDcl2	YPDcl3	EtOHcl1	-	Galcl1	YPDnc1	YPDnc2	YPDnc3	YPDnc4	EtOHnc1	EtOHnc2	Galnc1	Galnc2
12	UBA1	YKL210W	-	YPDcl3	EtOHcl1	EtOHcl2	Galcl1	YPDnc1	YPDnc2	YPDnc3	YPDnc4	EtOHnc1	EtOHnc2	Galnc1	Galnc2
12	ZRG8	YER033C	YPDcl2	YPDcl3	EtOHcl1	EtOHcl2	Galcl1	YPDnc1	YPDnc2	YPDnc3	YPDnc4	EtOHnc1	EtOHnc2	-	Galnc2
12	ELP2	YGR200C	YPDcl2	YPDcl3	EtOHcl1	-	Galcl1	YPDnc1	YPDnc2	YPDnc3	YPDnc4	EtOHnc1	EtOHnc2	Galnc1	Galnc2
12	GUF1	YLR289W	YPDcl2	-	EtOHcl1	EtOHcl2	Galcl1	YPDnc1	YPDnc2	YPDnc3	YPDnc4	EtOHnc1	EtOHnc2	Galnc1	Galnc2
12	SSY1	YDR160W	-	YPDcl3	EtOHcl1	EtOHcl2	Galcl1	YPDnc1	YPDnc2	YPDnc3	YPDnc4	EtOHnc1	EtOHnc2	Galnc1	Galnc2
12	LOT6	YLR011W	YPDcl2	YPDcl3	EtOHcl1	EtOHcl2	Galcl1	-	YPDnc2	YPDnc3	YPDnc4	EtOHnc1	EtOHnc2	Galnc1	Galnc2
12		YMR279C	YPDcl2	YPDcl3	EtOHcl1	EtOHcl2	Galcl1	YPDnc1	YPDnc2	YPDnc3	YPDnc4	EtOHnc1	-	Galnc1	Galnc2
12	RPN11	YFR004W	-	YPDcl3	EtOHcl1	EtOHcl2	Galcl1	YPDnc1	YPDnc2	YPDnc3	YPDnc4	EtOHnc1	EtOHnc2	Galnc1	Galnc2
12		YBR064W	YPDcl2	YPDcl3	EtOHcl1	EtOHcl2	Galcl1	YPDnc1	YPDnc2	YPDnc3	YPDnc4	EtOHnc1	EtOHnc2	Galnc1	-

12		YKL147C	YPDcl2	YPDcl3	-	EtOHcl2	Galcl1	YPDnc1	YPDnc2	YPDnc3	YPDnc4	EtOHnc1	EtOHnc2	Galnc1	Galnc2
12		YKL069W	YPDcl2	YPDcl3	EtOHcl1	EtOHcl2	Galcl1	YPDnc1	YPDnc2	YPDnc3	YPDnc4	-	EtOHnc2	Galnc1	Galnc2
12	YSP2	YDR326C	YPDcl2	YPDcl3	EtOHcl1	EtOHcl2	Galcl1	YPDnc1	YPDnc2	-	YPDnc4	EtOHnc1	EtOHnc2	Galnc1	Galnc2
12		YOR381W-	YPDcl2	YPDcl3	EtOHcl1	-	Galcl1	YPDnc1	YPDnc2	YPDnc3	YPDnc4	EtOHnc1	EtOHnc2	Galnc1	Galnc2
12		YPR197C	YPDcl2	YPDcl3	EtOHcl1	-	Galcl1	YPDnc1	YPDnc2	YPDnc3	YPDnc4	EtOHnc1	EtOHnc2	Galnc1	Galnc2
12	RPL1A	YPL220W	YPDcl2	-	EtOHcl1	EtOHcl2	Galcl1	YPDnc1	YPDnc2	YPDnc3	YPDnc4	EtOHnc1	EtOHnc2	Galnc1	Galnc2
12	TFB3	YDR460W	YPDcl2	YPDcl3	EtOHcl1	EtOHcl2	Galcl1	-	YPDnc2	YPDnc3	YPDnc4	EtOHnc1	EtOHnc2	Galnc1	Galnc2
12		YGL007C-A	-	YPDcl3	EtOHcl1	EtOHcl2	Galcl1	YPDnc1	YPDnc2	YPDnc3	YPDnc4	EtOHnc1	EtOHnc2	Galnc1	Galnc2
12		YMR173W-	YPDcl2	YPDcl3	EtOHcl1	EtOHcl2	Galcl1	YPDnc1	YPDnc2	YPDnc3	YPDnc4	EtOHnc1	-	Galnc1	Galnc2
12	YTP1	YNL237W	YPDcl2	YPDcl3	EtOHcl1	EtOHcl2	Galcl1	-	YPDnc2	YPDnc3	YPDnc4	EtOHnc1	EtOHnc2	Galnc1	Galnc2
12	DOA1	YKL213C	YPDcl2	YPDcl3	EtOHcl1	-	Galcl1	YPDnc1	YPDnc2	YPDnc3	YPDnc4	EtOHnc1	EtOHnc2	Galnc1	Galnc2
12	NNF1	YJR112W	YPDcl2	YPDcl3	EtOHcl1	EtOHcl2	Galcl1	YPDnc1	YPDnc2	YPDnc3	-	EtOHnc1	EtOHnc2	Galnc1	Galnc2
12	SNA4	YDL123W	YPDcl2	YPDcl3	EtOHcl1	EtOHcl2	Galcl1	YPDnc1	YPDnc2	YPDnc3	-	EtOHnc1	EtOHnc2	Galnc1	Galnc2
12	PBP4	YDL053C	-	YPDcl3	EtOHcl1	EtOHcl2	Galcl1	YPDnc1	YPDnc2	YPDnc3	YPDnc4	EtOHnc1	EtOHnc2	Galnc1	Galnc2
12	MPA43	YNL249C	YPDcl2	-	EtOHcl1	EtOHcl2	Galcl1	YPDnc1	YPDnc2	YPDnc3	YPDnc4	EtOHnc1	EtOHnc2	Galnc1	Galnc2
12	KEI1	YDR367W	YPDcl2	YPDcl3	EtOHcl1	-	Galcl1	YPDnc1	YPDnc2	YPDnc3	YPDnc4	EtOHnc1	EtOHnc2	Galnc1	Galnc2
12	SDH3	YKL141W	YPDcl2	YPDcl3	EtOHcl1	EtOHcl2	Galcl1	YPDnc1	YPDnc2	YPDnc3	YPDnc4	EtOHnc1	-	Galnc1	Galnc2
12	RNH201	YNL072W	YPDcl2	YPDcl3	EtOHcl1	-	Galcl1	YPDnc1	YPDnc2	YPDnc3	YPDnc4	EtOHnc1	EtOHnc2	Galnc1	Galnc2
12	IRS4	YKR019C	YPDcl2	YPDcl3	EtOHcl1	-	Galcl1	YPDnc1	YPDnc2	YPDnc3	YPDnc4	EtOHnc1	EtOHnc2	Galnc1	Galnc2
12		YOR062C	YPDcl2	YPDcl3	EtOHcl1	-	Galcl1	YPDnc1	YPDnc2	YPDnc3	YPDnc4	EtOHnc1	EtOHnc2	Galnc1	Galnc2
12	PBA1	YLR199C	YPDcl2	-	EtOHcl1	EtOHcl2	Galcl1	YPDnc1	YPDnc2	YPDnc3	YPDnc4	EtOHnc1	EtOHnc2	Galnc1	Galnc2
12	TMA22	YJR014W	-	YPDcl3	EtOHcl1	EtOHcl2	Galcl1	YPDnc1	YPDnc2	YPDnc3	YPDnc4	EtOHnc1	EtOHnc2	Galnc1	Galnc2
12		YDL050C	YPDcl2	YPDcl3	EtOHcl1	EtOHcl2	-	YPDnc1	YPDnc2	YPDnc3	YPDnc4	EtOHnc1	EtOHnc2	Galnc1	Galnc2
12		YDL086C-A	-	YPDcl3	EtOHcl1	EtOHcl2	Galcl1	YPDnc1	YPDnc2	YPDnc3	YPDnc4	EtOHnc1	EtOHnc2	Galnc1	Galnc2
12		YDR187C	-	YPDcl3	EtOHcl1	EtOHcl2	Galcl1	YPDnc1	YPDnc2	YPDnc3	YPDnc4	EtOHnc1	EtOHnc2	Galnc1	Galnc2
12		YDR336W	YPDcl2	-	EtOHcl1	EtOHcl2	Galcl1	YPDnc1	YPDnc2	YPDnc3	YPDnc4	EtOHnc1	EtOHnc2	Galnc1	Galnc2
12	SDS23	YGL056C	YPDcl2	YPDcl3	EtOHcl1	EtOHcl2	Galcl1	YPDnc1	YPDnc2	YPDnc3	YPDnc4	EtOHnc1	EtOHnc2	-	Galnc2
12		YBR071W	YPDcl2	YPDcl3	EtOHcl1	EtOHcl2	Galcl1	YPDnc1	YPDnc2	YPDnc3	YPDnc4	EtOHnc1	-	Galnc1	Galnc2
12	ECM33	YBR078W	-	YPDcl3	EtOHcl1	EtOHcl2	Galcl1	YPDnc1	YPDnc2	YPDnc3	YPDnc4	EtOHnc1	EtOHnc2	Galnc1	Galnc2
12	BCY1	YIL033C	YPDcl2	YPDcl3	EtOHcl1	-	Galcl1	YPDnc1	YPDnc2	YPDnc3	YPDnc4	EtOHnc1	EtOHnc2	Galnc1	Galnc2
12	IST3	YIR005W	-	YPDcl3	EtOHcl1	EtOHcl2	Galcl1	YPDnc1	YPDnc2	YPDnc3	YPDnc4	EtOHnc1	EtOHnc2	Galnc1	Galnc2
12	HEM3	YDL205C	YPDcl2	YPDcl3	-	EtOHcl2	Galcl1	YPDnc1	YPDnc2	YPDnc3	YPDnc4	EtOHnc1	EtOHnc2	Galnc1	Galnc2
12	TKL2	YBR117C	YPDcl2	YPDcl3	EtOHcl1	-	Galcl1	YPDnc1	YPDnc2	YPDnc3	YPDnc4	EtOHnc1	EtOHnc2	Galnc1	Galnc2
12		YFR006W	YPDcl2	YPDcl3	EtOHcl1	-	Galcl1	YPDnc1	YPDnc2	YPDnc3	YPDnc4	EtOHnc1	EtOHnc2	Galnc1	Galnc2

12	LOS1	YKL205W	-	YPDcl3	EtOHcl1	EtOHcl2	Galcl1	YPDnc1	YPDnc2	YPDnc3	YPDnc4	EtOHnc1	EtOHnc2	Galnc1	Galnc2
12	CMK2	YOL016C	-	YPDcl3	EtOHcl1	EtOHcl2	Galcl1	YPDnc1	YPDnc2	YPDnc3	YPDnc4	EtOHnc1	EtOHnc2	Galnc1	Galnc2
12	CUP9	YPL177C	-	YPDcl3	EtOHcl1	EtOHcl2	Galcl1	YPDnc1	YPDnc2	YPDnc3	YPDnc4	EtOHnc1	EtOHnc2	Galnc1	Galnc2
12		YJL136W-A	YPDcl2	YPDcl3	EtOHcl1	EtOHcl2	Galcl1	YPDnc1	YPDnc2	YPDnc3	YPDnc4	-	EtOHnc2	Galnc1	Galnc2
12		YNR066C	-	YPDcl3	EtOHcl1	EtOHcl2	Galcl1	YPDnc1	YPDnc2	YPDnc3	YPDnc4	EtOHnc1	EtOHnc2	Galnc1	Galnc2
12		YDR455C	YPDcl2	-	EtOHcl1	EtOHcl2	Galcl1	YPDnc1	YPDnc2	YPDnc3	YPDnc4	EtOHnc1	EtOHnc2	Galnc1	Galnc2
12	UAF30	YOR295W	YPDcl2	YPDcl3	EtOHcl1	EtOHcl2	Galcl1	YPDnc1	-	YPDnc3	YPDnc4	EtOHnc1	EtOHnc2	Galnc1	Galnc2
12		YGR066C	YPDcl2	YPDcl3	EtOHcl1	-	Galcl1	YPDnc1	YPDnc2	YPDnc3	YPDnc4	EtOHnc1	EtOHnc2	Galnc1	Galnc2
12	OPT1	YJL212C	YPDcl2	YPDcl3	EtOHcl1	EtOHcl2	Galcl1	YPDnc1	YPDnc2	-	YPDnc4	EtOHnc1	EtOHnc2	Galnc1	Galnc2
12		YER172C-A	-	YPDcl3	EtOHcl1	EtOHcl2	Galcl1	YPDnc1	YPDnc2	YPDnc3	YPDnc4	EtOHnc1	EtOHnc2	Galnc1	Galnc2
12	ARA2	YMR041C	YPDcl2	-	EtOHcl1	EtOHcl2	Galcl1	YPDnc1	YPDnc2	YPDnc3	YPDnc4	EtOHnc1	EtOHnc2	Galnc1	Galnc2
12	THS1	YIL078W	YPDcl2	YPDcl3	EtOHcl1	EtOHcl2	Galcl1	YPDnc1	YPDnc2	YPDnc3	YPDnc4	EtOHnc1	EtOHnc2	Galnc1	-
12	PRP11	YDL043C	YPDcl2	YPDcl3	EtOHcl1	EtOHcl2	Galcl1	YPDnc1	YPDnc2	YPDnc3	YPDnc4	-	EtOHnc2	Galnc1	Galnc2
12	PDR10	YOR328W	YPDcl2	YPDcl3	EtOHcl1	EtOHcl2	Galcl1	YPDnc1	YPDnc2	YPDnc3	YPDnc4	EtOHnc1	-	Galnc1	Galnc2
12		YLR041W	YPDcl2	YPDcl3	EtOHcl1	EtOHcl2	Galcl1	YPDnc1	YPDnc2	YPDnc3	YPDnc4	EtOHnc1	-	Galnc1	Galnc2
12	DIF1	YLR437C	YPDcl2	YPDcl3	EtOHcl1	EtOHcl2	Galcl1	YPDnc1	YPDnc2	YPDnc3	YPDnc4	EtOHnc1	EtOHnc2	-	Galnc2
12	AUS1	YOR011W	YPDcl2	YPDcl3	EtOHcl1	EtOHcl2	Galcl1	YPDnc1	YPDnc2	YPDnc3	YPDnc4	-	EtOHnc2	Galnc1	Galnc2
12	DPH1	YIL103W	YPDcl2	YPDcl3	EtOHcl1	-	Galcl1	YPDnc1	YPDnc2	YPDnc3	YPDnc4	EtOHnc1	EtOHnc2	Galnc1	Galnc2
12	LDB7	YBL006C	-	YPDcl3	EtOHcl1	EtOHcl2	Galcl1	YPDnc1	YPDnc2	YPDnc3	YPDnc4	EtOHnc1	EtOHnc2	Galnc1	Galnc2
12	ACF2	YLR144C	YPDcl2	YPDcl3	-	EtOHcl2	Galcl1	YPDnc1	YPDnc2	YPDnc3	YPDnc4	EtOHnc1	EtOHnc2	Galnc1	Galnc2
12	PIN3	YPR154W	YPDcl2	YPDcl3	EtOHcl1	-	Galcl1	YPDnc1	YPDnc2	YPDnc3	YPDnc4	EtOHnc1	EtOHnc2	Galnc1	Galnc2
12	RSC3	YDR303C	YPDcl2	YPDcl3	EtOHcl1	-	Galcl1	YPDnc1	YPDnc2	YPDnc3	YPDnc4	EtOHnc1	EtOHnc2	Galnc1	Galnc2
12		YDR154C	YPDcl2	YPDcl3	EtOHcl1	EtOHcl2	Galcl1	YPDnc1	YPDnc2	YPDnc3	-	EtOHnc1	EtOHnc2	Galnc1	Galnc2
12	SCC4	YER147C	YPDcl2	YPDcl3	EtOHcl1	-	Galcl1	YPDnc1	YPDnc2	YPDnc3	YPDnc4	EtOHnc1	EtOHnc2	Galnc1	Galnc2
12	ICE2	YIL090W	YPDcl2	YPDcl3	EtOHcl1	EtOHcl2	Galcl1	-	YPDnc2	YPDnc3	YPDnc4	EtOHnc1	EtOHnc2	Galnc1	Galnc2
12	CPA2	YJR109C	YPDcl2	YPDcl3	EtOHcl1	EtOHcl2	Galcl1	YPDnc1	YPDnc2	YPDnc3	-	EtOHnc1	EtOHnc2	Galnc1	Galnc2
12	NUP100	YKL068W	YPDcl2	YPDcl3	EtOHcl1	-	Galcl1	YPDnc1	YPDnc2	YPDnc3	YPDnc4	EtOHnc1	EtOHnc2	Galnc1	Galnc2
12	ADE8	YDR408C	-	YPDcl3	EtOHcl1	EtOHcl2	Galcl1	YPDnc1	YPDnc2	YPDnc3	YPDnc4	EtOHnc1	EtOHnc2	Galnc1	Galnc2
12	PCC1	YKR095W-/	YPDcl2	YPDcl3	EtOHcl1	EtOHcl2	Galcl1	YPDnc1	YPDnc2	YPDnc3	YPDnc4	EtOHnc1	-	Galnc1	Galnc2
12	DOT1	YDR440W	YPDcl2	YPDcl3	EtOHcl1	-	Galcl1	YPDnc1	YPDnc2	YPDnc3	YPDnc4	EtOHnc1	EtOHnc2	Galnc1	Galnc2
12	RPC31	YNL151C	YPDcl2	YPDcl3	EtOHcl1	-	Galcl1	YPDnc1	YPDnc2	YPDnc3	YPDnc4	EtOHnc1	EtOHnc2	Galnc1	Galnc2
12	NCS6	YGL211W	YPDcl2	YPDcl3	-	EtOHcl2	Galcl1	YPDnc1	YPDnc2	YPDnc3	YPDnc4	EtOHnc1	EtOHnc2	Galnc1	Galnc2
12		YMR304C-/	-	YPDcl3	EtOHcl1	EtOHcl2	Galcl1	YPDnc1	YPDnc2	YPDnc3	YPDnc4	EtOHnc1	EtOHnc2	Galnc1	Galnc2
12	GLN1	YPR035W	YPDcl2	YPDcl3	EtOHcl1	EtOHcl2	Galcl1	YPDnc1	-	YPDnc3	YPDnc4	EtOHnc1	EtOHnc2	Galnc1	Galnc2

12	DHR2	YKL078W	-	YPDcl3	EtOHcl1	EtOHcl2	Galcl1	YPDnc1	YPDnc2	YPDnc3	YPDnc4	EtOHnc1	EtOHnc2	Galnc1	Galnc2
12	CPR1	YDR155C	YPDcl2	YPDcl3	EtOHcl1	EtOHcl2	Galcl1	YPDnc1	YPDnc2	YPDnc3	-	EtOHnc1	EtOHnc2	Galnc1	Galnc2
12	YOS1	YER074W-/	YPDcl2	YPDcl3	EtOHcl1	EtOHcl2	Galcl1	YPDnc1	YPDnc2	YPDnc3	YPDnc4	EtOHnc1	-	Galnc1	Galnc2
12		YGR201C	-	YPDcl3	EtOHcl1	EtOHcl2	Galcl1	YPDnc1	YPDnc2	YPDnc3	YPDnc4	EtOHnc1	EtOHnc2	Galnc1	Galnc2
12		YIL163C	YPDcl2	YPDcl3	EtOHcl1	-	Galcl1	YPDnc1	YPDnc2	YPDnc3	YPDnc4	EtOHnc1	EtOHnc2	Galnc1	Galnc2
12	HKR1	YDR420W	YPDcl2	YPDcl3	-	EtOHcl2	Galcl1	YPDnc1	YPDnc2	YPDnc3	YPDnc4	EtOHnc1	EtOHnc2	Galnc1	Galnc2
12		YDR186C	YPDcl2	YPDcl3	EtOHcl1	EtOHcl2	Galcl1	YPDnc1	YPDnc2	YPDnc3	-	EtOHnc1	EtOHnc2	Galnc1	Galnc2
12	GPI15	YNL038W	YPDcl2	YPDcl3	EtOHcl1	-	Galcl1	YPDnc1	YPDnc2	YPDnc3	YPDnc4	EtOHnc1	EtOHnc2	Galnc1	Galnc2
12	YIM1	YMR152W	YPDcl2	YPDcl3	EtOHcl1	EtOHcl2	Galcl1	YPDnc1	YPDnc2	YPDnc3	-	EtOHnc1	EtOHnc2	Galnc1	Galnc2
12	IDI1	YPL117C	YPDcl2	YPDcl3	-	EtOHcl2	Galcl1	YPDnc1	YPDnc2	YPDnc3	YPDnc4	EtOHnc1	EtOHnc2	Galnc1	Galnc2
12	DGK1	YOR311C	YPDcl2	-	EtOHcl1	EtOHcl2	Galcl1	YPDnc1	YPDnc2	YPDnc3	YPDnc4	EtOHnc1	EtOHnc2	Galnc1	Galnc2
12	YAP6	YDR259C	-	YPDcl3	EtOHcl1	EtOHcl2	Galcl1	YPDnc1	YPDnc2	YPDnc3	YPDnc4	EtOHnc1	EtOHnc2	Galnc1	Galnc2
12	HXT5	YHR096C	YPDcl2	YPDcl3	EtOHcl1	EtOHcl2	Galcl1	YPDnc1	YPDnc2	YPDnc3	YPDnc4	EtOHnc1	-	Galnc1	Galnc2
12	FIP1	YJR093C	YPDcl2	-	EtOHcl1	EtOHcl2	Galcl1	YPDnc1	YPDnc2	YPDnc3	YPDnc4	EtOHnc1	EtOHnc2	Galnc1	Galnc2
12	YKU80	YMR106C	YPDcl2	YPDcl3	EtOHcl1	EtOHcl2	Galcl1	YPDnc1	YPDnc2	YPDnc3	YPDnc4	EtOHnc1	-	Galnc1	Galnc2
12	NQM1	YGR043C	YPDcl2	YPDcl3	EtOHcl1	-	Galcl1	YPDnc1	YPDnc2	YPDnc3	YPDnc4	EtOHnc1	EtOHnc2	Galnc1	Galnc2
12	MNN11	YJL183W	YPDcl2	YPDcl3	EtOHcl1	EtOHcl2	Galcl1	YPDnc1	YPDnc2	YPDnc3	YPDnc4	-	EtOHnc2	Galnc1	Galnc2
12	ERG13	YML126C	-	YPDcl3	EtOHcl1	EtOHcl2	Galcl1	YPDnc1	YPDnc2	YPDnc3	YPDnc4	EtOHnc1	EtOHnc2	Galnc1	Galnc2
12	ECM21	YBL101C	YPDcl2	YPDcl3	EtOHcl1	EtOHcl2	Galcl1	YPDnc1	YPDnc2	YPDnc3	YPDnc4	-	EtOHnc2	Galnc1	Galnc2
12	AIM17	YHL021C	YPDcl2	YPDcl3	EtOHcl1	-	Galcl1	YPDnc1	YPDnc2	YPDnc3	YPDnc4	EtOHnc1	EtOHnc2	Galnc1	Galnc2
12	SSO1	YPL232W	-	YPDcl3	EtOHcl1	EtOHcl2	Galcl1	YPDnc1	YPDnc2	YPDnc3	YPDnc4	EtOHnc1	EtOHnc2	Galnc1	Galnc2
12	ACT1	YFL039C	YPDcl2	YPDcl3	EtOHcl1	EtOHcl2	Galcl1	YPDnc1	YPDnc2	YPDnc3	-	EtOHnc1	EtOHnc2	Galnc1	Galnc2
12		YLR164W	YPDcl2	YPDcl3	EtOHcl1	-	Galcl1	YPDnc1	YPDnc2	YPDnc3	YPDnc4	EtOHnc1	EtOHnc2	Galnc1	Galnc2
12		YKR047W	-	YPDcl3	EtOHcl1	EtOHcl2	Galcl1	YPDnc1	YPDnc2	YPDnc3	YPDnc4	EtOHnc1	EtOHnc2	Galnc1	Galnc2
12		YOR263C	YPDcl2	YPDcl3	EtOHcl1	EtOHcl2	-	YPDnc1	YPDnc2	YPDnc3	YPDnc4	EtOHnc1	EtOHnc2	Galnc1	Galnc2
12	RPL21B	YPL079W	YPDcl2	YPDcl3	EtOHcl1	-	Galcl1	YPDnc1	YPDnc2	YPDnc3	YPDnc4	EtOHnc1	EtOHnc2	Galnc1	Galnc2
12		YHR182W	YPDcl2	YPDcl3	EtOHcl1	EtOHcl2	-	YPDnc1	YPDnc2	YPDnc3	YPDnc4	EtOHnc1	EtOHnc2	Galnc1	Galnc2
12		YER137C	YPDcl2	YPDcl3	EtOHcl1	EtOHcl2	Galcl1	YPDnc1	YPDnc2	YPDnc3	YPDnc4	EtOHnc1	-	Galnc1	Galnc2
12	CHA4	YLR098C	-	YPDcl3	EtOHcl1	EtOHcl2	Galcl1	YPDnc1	YPDnc2	YPDnc3	YPDnc4	EtOHnc1	EtOHnc2	Galnc1	Galnc2
12	HAT2	YEL056W	YPDcl2	-	EtOHcl1	EtOHcl2	Galcl1	YPDnc1	YPDnc2	YPDnc3	YPDnc4	EtOHnc1	EtOHnc2	Galnc1	Galnc2
12	AVT7	YIL088C	YPDcl2	YPDcl3	EtOHcl1	-	Galcl1	YPDnc1	YPDnc2	YPDnc3	YPDnc4	EtOHnc1	EtOHnc2	Galnc1	Galnc2
12	AGP1	YCL025C	YPDcl2	YPDcl3	EtOHcl1	-	Galcl1	YPDnc1	YPDnc2	YPDnc3	YPDnc4	EtOHnc1	EtOHnc2	Galnc1	Galnc2
12	BNI1	YNL271C	YPDcl2	YPDcl3	EtOHcl1	-	Galcl1	YPDnc1	YPDnc2	YPDnc3	YPDnc4	EtOHnc1	EtOHnc2	Galnc1	Galnc2
12	RIP1	YEL024W	YPDcl2	YPDcl3	EtOHcl1	EtOHcl2	Galcl1	YPDnc1	YPDnc2	YPDnc3	YPDnc4	EtOHnc1	EtOHnc2	-	Galnc2

12	HXT2	YMR011W	YPDcl2	YPDcl3	-	EtOHcl2	Galcl1	YPDnc1	YPDnc2	YPDnc3	YPDnc4	EtOHnc1	EtOHnc2	Galnc1	Galnc2
12		YJL218W	YPDcl2	-	EtOHcl1	EtOHcl2	Galcl1	YPDnc1	YPDnc2	YPDnc3	YPDnc4	EtOHnc1	EtOHnc2	Galnc1	Galnc2
12	RPS8A	YBL072C	YPDcl2	YPDcl3	EtOHcl1	EtOHcl2	Galcl1	YPDnc1	YPDnc2	YPDnc3	YPDnc4	EtOHnc1	EtOHnc2	-	Galnc2
12	RSC6	YCR052W	YPDcl2	YPDcl3	EtOHcl1	EtOHcl2	-	YPDnc1	YPDnc2	YPDnc3	YPDnc4	EtOHnc1	EtOHnc2	Galnc1	Galnc2
12	PDR11	YIL013C	YPDcl2	-	EtOHcl1	EtOHcl2	Galcl1	YPDnc1	YPDnc2	YPDnc3	YPDnc4	EtOHnc1	EtOHnc2	Galnc1	Galnc2
12	NCA3	YJL116C	YPDcl2	YPDcl3	EtOHcl1	-	Galcl1	YPDnc1	YPDnc2	YPDnc3	YPDnc4	EtOHnc1	EtOHnc2	Galnc1	Galnc2
12		YMR187C	-	YPDcl3	EtOHcl1	EtOHcl2	Galcl1	YPDnc1	YPDnc2	YPDnc3	YPDnc4	EtOHnc1	EtOHnc2	Galnc1	Galnc2
12	PIC2	YER053C	YPDcl2	YPDcl3	EtOHcl1	EtOHcl2	Galcl1	YPDnc1	YPDnc2	YPDnc3	YPDnc4	EtOHnc1	-	Galnc1	Galnc2
12	RRS1	YOR294W	YPDcl2	YPDcl3	EtOHcl1	EtOHcl2	Galcl1	YPDnc1	YPDnc2	YPDnc3	YPDnc4	EtOHnc1	-	Galnc1	Galnc2
12	TPO4	YOR273C	YPDcl2	YPDcl3	EtOHcl1	EtOHcl2	Galcl1	YPDnc1	YPDnc2	YPDnc3	YPDnc4	EtOHnc1	-	Galnc1	Galnc2
12	EFT1	YOR133W	YPDcl2	YPDcl3	EtOHcl1	-	Galcl1	YPDnc1	YPDnc2	YPDnc3	YPDnc4	EtOHnc1	EtOHnc2	Galnc1	Galnc2
12	HSK3	YKL138C-A	YPDcl2	YPDcl3	EtOHcl1	EtOHcl2	Galcl1	-	YPDnc2	YPDnc3	YPDnc4	EtOHnc1	EtOHnc2	Galnc1	Galnc2
12	ALG14	YBR070C	YPDcl2	YPDcl3	EtOHcl1	-	Galcl1	YPDnc1	YPDnc2	YPDnc3	YPDnc4	EtOHnc1	EtOHnc2	Galnc1	Galnc2
12	RPB8	YOR224C	YPDcl2	YPDcl3	EtOHcl1	EtOHcl2	Galcl1	YPDnc1	YPDnc2	YPDnc3	YPDnc4	EtOHnc1	EtOHnc2	Galnc1	-
12		YNR005C	-	YPDcl3	EtOHcl1	EtOHcl2	Galcl1	YPDnc1	YPDnc2	YPDnc3	YPDnc4	EtOHnc1	EtOHnc2	Galnc1	Galnc2
12	HSE1	YHL002W	YPDcl2	-	EtOHcl1	EtOHcl2	Galcl1	YPDnc1	YPDnc2	YPDnc3	YPDnc4	EtOHnc1	EtOHnc2	Galnc1	Galnc2
12	DBP10	YDL031W	YPDcl2	YPDcl3	EtOHcl1	-	Galcl1	YPDnc1	YPDnc2	YPDnc3	YPDnc4	EtOHnc1	EtOHnc2	Galnc1	Galnc2
12	YSW1	YBR148W	YPDcl2	YPDcl3	EtOHcl1	EtOHcl2	Galcl1	YPDnc1	YPDnc2	YPDnc3	YPDnc4	EtOHnc1	-	Galnc1	Galnc2
12		YIL071W-A	YPDcl2	YPDcl3	EtOHcl1	EtOHcl2	Galcl1	-	YPDnc2	YPDnc3	YPDnc4	EtOHnc1	EtOHnc2	Galnc1	Galnc2
12		YLR347W-A	-	YPDcl3	EtOHcl1	EtOHcl2	Galcl1	YPDnc1	YPDnc2	YPDnc3	YPDnc4	EtOHnc1	EtOHnc2	Galnc1	Galnc2
12	AVT1	YJR001W	-	YPDcl3	EtOHcl1	EtOHcl2	Galcl1	YPDnc1	YPDnc2	YPDnc3	YPDnc4	EtOHnc1	EtOHnc2	Galnc1	Galnc2
12	URK1	YNR012W	-	YPDcl3	EtOHcl1	EtOHcl2	Galcl1	YPDnc1	YPDnc2	YPDnc3	YPDnc4	EtOHnc1	EtOHnc2	Galnc1	Galnc2
12		YHL012W	-	YPDcl3	EtOHcl1	EtOHcl2	Galcl1	YPDnc1	YPDnc2	YPDnc3	YPDnc4	EtOHnc1	EtOHnc2	Galnc1	Galnc2
12		YOR268C	YPDcl2	YPDcl3	EtOHcl1	-	Galcl1	YPDnc1	YPDnc2	YPDnc3	YPDnc4	EtOHnc1	EtOHnc2	Galnc1	Galnc2
12	BIO2	YGR286C	YPDcl2	-	EtOHcl1	EtOHcl2	Galcl1	YPDnc1	YPDnc2	YPDnc3	YPDnc4	EtOHnc1	EtOHnc2	Galnc1	Galnc2
12	TOM6	YOR045W	YPDcl2	YPDcl3	EtOHcl1	EtOHcl2	Galcl1	YPDnc1	YPDnc2	-	YPDnc4	EtOHnc1	EtOHnc2	Galnc1	Galnc2
12	ECM18	YDR125C	YPDcl2	YPDcl3	EtOHcl1	EtOHcl2	Galcl1	-	YPDnc2	YPDnc3	YPDnc4	EtOHnc1	EtOHnc2	Galnc1	Galnc2
12	MEX67	YPL169C	YPDcl2	YPDcl3	EtOHcl1	EtOHcl2	Galcl1	YPDnc1	YPDnc2	YPDnc3	YPDnc4	EtOHnc1	-	Galnc1	Galnc2
12	TRP4	YDR354W	YPDcl2	-	EtOHcl1	EtOHcl2	Galcl1	YPDnc1	YPDnc2	YPDnc3	YPDnc4	EtOHnc1	EtOHnc2	Galnc1	Galnc2
12	PTR3	YFR029W	-	YPDcl3	EtOHcl1	EtOHcl2	Galcl1	YPDnc1	YPDnc2	YPDnc3	YPDnc4	EtOHnc1	EtOHnc2	Galnc1	Galnc2
12	DBF2	YGR092W	-	YPDcl3	EtOHcl1	EtOHcl2	Galcl1	YPDnc1	YPDnc2	YPDnc3	YPDnc4	EtOHnc1	EtOHnc2	Galnc1	Galnc2
12	MMS21	YEL019C	YPDcl2	YPDcl3	EtOHcl1	EtOHcl2	Galcl1	-	YPDnc2	YPDnc3	YPDnc4	EtOHnc1	EtOHnc2	Galnc1	Galnc2
12	RPN6	YDL097C	YPDcl2	-	EtOHcl1	EtOHcl2	Galcl1	YPDnc1	YPDnc2	YPDnc3	YPDnc4	EtOHnc1	EtOHnc2	Galnc1	Galnc2
12	CAB4	YGR277C	YPDcl2	-	EtOHcl1	EtOHcl2	Galcl1	YPDnc1	YPDnc2	YPDnc3	YPDnc4	EtOHnc1	EtOHnc2	Galnc1	Galnc2

12	IME1	YJR094C	YPDcl2	YPDcl3	EtOHcl1	-	Galcl1	YPDnc1	YPDnc2	YPDnc3	YPDnc4	EtOHnc1	EtOHnc2	Galnc1	Galnc2
12		YDR355C	YPDcl2	YPDcl3	EtOHcl1	EtOHcl2	Galcl1	YPDnc1	YPDnc2	YPDnc3	YPDnc4	EtOHnc1	EtOHnc2	Galnc1	-
12	LSP1	YPL004C	YPDcl2	YPDcl3	EtOHcl1	EtOHcl2	Galcl1	YPDnc1	YPDnc2	YPDnc3	YPDnc4	EtOHnc1	-	Galnc1	Galnc2
12		YOR082C	YPDcl2	YPDcl3	EtOHcl1	EtOHcl2	-	YPDnc1	YPDnc2	YPDnc3	YPDnc4	EtOHnc1	EtOHnc2	Galnc1	Galnc2
12	NSE5	YML023C	YPDcl2	-	EtOHcl1	EtOHcl2	Galcl1	YPDnc1	YPDnc2	YPDnc3	YPDnc4	EtOHnc1	EtOHnc2	Galnc1	Galnc2
12		YLR400W	YPDcl2	YPDcl3	EtOHcl1	EtOHcl2	Galcl1	YPDnc1	-	YPDnc3	YPDnc4	EtOHnc1	EtOHnc2	Galnc1	Galnc2
12	PCL9	YDL179W	YPDcl2	YPDcl3	-	EtOHcl2	Galcl1	YPDnc1	YPDnc2	YPDnc3	YPDnc4	EtOHnc1	EtOHnc2	Galnc1	Galnc2
12		YOR364W	YPDcl2	YPDcl3	EtOHcl1	-	Galcl1	YPDnc1	YPDnc2	YPDnc3	YPDnc4	EtOHnc1	EtOHnc2	Galnc1	Galnc2
12	BUD22	YMR014W	YPDcl2	YPDcl3	-	EtOHcl2	Galcl1	YPDnc1	YPDnc2	YPDnc3	YPDnc4	EtOHnc1	EtOHnc2	Galnc1	Galnc2
12	GMH1	YKR030W	YPDcl2	YPDcl3	-	EtOHcl2	Galcl1	YPDnc1	YPDnc2	YPDnc3	YPDnc4	EtOHnc1	EtOHnc2	Galnc1	Galnc2
12	SDS24	YBR214W	YPDcl2	YPDcl3	EtOHcl1	EtOHcl2	Galcl1	YPDnc1	YPDnc2	YPDnc3	-	EtOHnc1	EtOHnc2	Galnc1	Galnc2
11		YEL008C-A	YPDcl2	YPDcl3	EtOHcl1	-	Galcl1	YPDnc1	YPDnc2	YPDnc3	-	EtOHnc1	EtOHnc2	Galnc1	Galnc2
11	YBP2	YGL060W	YPDcl2	YPDcl3	EtOHcl1	EtOHcl2	Galcl1	YPDnc1	YPDnc2	YPDnc3	YPDnc4	-	EtOHnc2	Galnc1	-
11	MRPL11	YDL202W	YPDcl2	YPDcl3	EtOHcl1	-	-	YPDnc1	YPDnc2	YPDnc3	YPDnc4	EtOHnc1	EtOHnc2	Galnc1	Galnc2
11	CTR1	YPR124W	YPDcl2	-	EtOHcl1	EtOHcl2	Galcl1	YPDnc1	-	YPDnc3	YPDnc4	EtOHnc1	EtOHnc2	Galnc1	Galnc2
11	PMR1	YGL167C	YPDcl2	YPDcl3	EtOHcl1	EtOHcl2	Galcl1	YPDnc1	-	YPDnc3	-	EtOHnc1	EtOHnc2	Galnc1	Galnc2
11	DCS1	YLR270W	YPDcl2	YPDcl3	EtOHcl1	-	Galcl1	YPDnc1	-	YPDnc3	YPDnc4	EtOHnc1	EtOHnc2	Galnc1	Galnc2
11	LCB1	YMR296C	YPDcl2	YPDcl3	-	-	Galcl1	YPDnc1	YPDnc2	YPDnc3	YPDnc4	EtOHnc1	EtOHnc2	Galnc1	Galnc2
11	COX23	YHR116W	-	-	EtOHcl1	EtOHcl2	Galcl1	YPDnc1	YPDnc2	YPDnc3	YPDnc4	EtOHnc1	EtOHnc2	Galnc1	Galnc2
11	ECM23	YPL021W	YPDcl2	YPDcl3	EtOHcl1	EtOHcl2	Galcl1	-	YPDnc2	YPDnc3	YPDnc4	EtOHnc1	EtOHnc2	Galnc1	-
11	TAX4	YJL083W	-	YPDcl3	EtOHcl1	-	Galcl1	YPDnc1	YPDnc2	YPDnc3	YPDnc4	EtOHnc1	EtOHnc2	Galnc1	Galnc2
11		YMR315W	YPDcl2	YPDcl3	EtOHcl1	EtOHcl2	Galcl1	-	-	YPDnc3	YPDnc4	EtOHnc1	EtOHnc2	Galnc1	Galnc2
11		YDR467C	YPDcl2	YPDcl3	EtOHcl1	-	Galcl1	-	YPDnc2	YPDnc3	YPDnc4	EtOHnc1	EtOHnc2	Galnc1	Galnc2
11	CTR3	YLR411W	-	YPDcl3	EtOHcl1	EtOHcl2	-	YPDnc1	YPDnc2	YPDnc3	YPDnc4	EtOHnc1	EtOHnc2	Galnc1	Galnc2
11	SUB1	YMR039C	YPDcl2	YPDcl3	EtOHcl1	-	Galcl1	YPDnc1	-	YPDnc3	YPDnc4	EtOHnc1	EtOHnc2	Galnc1	Galnc2
11	ATH1	YPR026W	YPDcl2	YPDcl3	EtOHcl1	EtOHcl2	Galcl1	YPDnc1	-	-	YPDnc4	EtOHnc1	EtOHnc2	Galnc1	Galnc2
11	CWP2	YKL096W-A	YPDcl2	-	EtOHcl1	EtOHcl2	Galcl1	YPDnc1	YPDnc2	YPDnc3	-	EtOHnc1	EtOHnc2	Galnc1	Galnc2
11	VPS74	YDR372C	YPDcl2	YPDcl3	EtOHcl1	EtOHcl2	Galcl1	YPDnc1	-	-	YPDnc4	EtOHnc1	EtOHnc2	Galnc1	Galnc2
11	FLR1	YBR008C	YPDcl2	YPDcl3	EtOHcl1	EtOHcl2	Galcl1	-	YPDnc2	YPDnc3	YPDnc4	EtOHnc1	EtOHnc2	Galnc1	-
11	CLB4	YLR210W	YPDcl2	YPDcl3	EtOHcl1	EtOHcl2	Galcl1	-	YPDnc2	YPDnc3	YPDnc4	-	EtOHnc2	Galnc1	Galnc2
11		YDL025C	YPDcl2	-	EtOHcl1	EtOHcl2	Galcl1	-	YPDnc2	YPDnc3	YPDnc4	EtOHnc1	EtOHnc2	Galnc1	Galnc2
11	AAD4	YDL243C	-	-	EtOHcl1	EtOHcl2	Galcl1	YPDnc1	YPDnc2	YPDnc3	YPDnc4	EtOHnc1	EtOHnc2	Galnc1	Galnc2
11	ATG26	YLR189C	-	-	EtOHcl1	EtOHcl2	Galcl1	YPDnc1	YPDnc2	YPDnc3	YPDnc4	EtOHnc1	EtOHnc2	Galnc1	Galnc2
11		YLR416C	-	YPDcl3	EtOHcl1	EtOHcl2	Galcl1	YPDnc1	-	YPDnc3	YPDnc4	EtOHnc1	EtOHnc2	Galnc1	Galnc2

11	RHO5	YNL180C	YPDcl2	YPDcl3	EtOHcl1	-	-	YPDnc1	YPDnc2	YPDnc3	YPDnc4	EtOHnc1	EtOHnc2	Galnc1	Galnc2
11	SLS1	YLR139C	-	YPDcl3	EtOHcl1	-	Galcl1	YPDnc1	YPDnc2	YPDnc3	YPDnc4	EtOHnc1	EtOHnc2	Galnc1	Galnc2
11	LEU4	YNL104C	-	YPDcl3	EtOHcl1	-	Galcl1	YPDnc1	YPDnc2	YPDnc3	YPDnc4	EtOHnc1	EtOHnc2	Galnc1	Galnc2
11	MST1	YKL194C	YPDcl2	YPDcl3	-	EtOHcl2	Galcl1	YPDnc1	YPDnc2	YPDnc3	YPDnc4	EtOHnc1	EtOHnc2	-	Galnc2
11	YEF3	YLR249W	YPDcl2	YPDcl3	-	EtOHcl2	Galcl1	YPDnc1	YPDnc2	YPDnc3	YPDnc4	EtOHnc1	-	Galnc1	Galnc2
11	TMA46	YOR091W	YPDcl2	-	EtOHcl1	EtOHcl2	Galcl1	YPDnc1	YPDnc2	YPDnc3	YPDnc4	EtOHnc1	-	Galnc1	Galnc2
11	GFA1	YKL104C	-	YPDcl3	EtOHcl1	-	Galcl1	YPDnc1	YPDnc2	YPDnc3	YPDnc4	EtOHnc1	EtOHnc2	Galnc1	Galnc2
11	MPM1	YJL066C	YPDcl2	-	EtOHcl1	-	Galcl1	YPDnc1	YPDnc2	YPDnc3	YPDnc4	EtOHnc1	EtOHnc2	Galnc1	Galnc2
11	CLA4	YNL298W	YPDcl2	-	EtOHcl1	-	Galcl1	YPDnc1	YPDnc2	YPDnc3	YPDnc4	EtOHnc1	EtOHnc2	Galnc1	Galnc2
11	PEX13	YLR191W	-	YPDcl3	EtOHcl1	EtOHcl2	Galcl1	YPDnc1	YPDnc2	YPDnc3	-	EtOHnc1	EtOHnc2	Galnc1	Galnc2
11	PRP4	YPR178W	YPDcl2	YPDcl3	EtOHcl1	EtOHcl2	Galcl1	-	YPDnc2	-	YPDnc4	EtOHnc1	EtOHnc2	Galnc1	Galnc2
11	RHO3	YIL118W	-	YPDcl3	EtOHcl1	-	Galcl1	YPDnc1	YPDnc2	YPDnc3	YPDnc4	EtOHnc1	EtOHnc2	Galnc1	Galnc2
11	SEC21	YNL287W	YPDcl2	YPDcl3	-	EtOHcl2	Galcl1	YPDnc1	YPDnc2	YPDnc3	YPDnc4	EtOHnc1	-	Galnc1	Galnc2
11		YLR269C	-	YPDcl3	EtOHcl1	EtOHcl2	Galcl1	YPDnc1	YPDnc2	YPDnc3	YPDnc4	EtOHnc1	-	Galnc1	Galnc2
11	BRP1	YGL007W	-	-	EtOHcl1	EtOHcl2	Galcl1	YPDnc1	YPDnc2	YPDnc3	YPDnc4	EtOHnc1	EtOHnc2	Galnc1	Galnc2
11	STT3	YGL022W	YPDcl2	-	EtOHcl1	-	Galcl1	YPDnc1	YPDnc2	YPDnc3	YPDnc4	EtOHnc1	EtOHnc2	Galnc1	Galnc2
11	HST4	YDR191W	-	-	EtOHcl1	EtOHcl2	Galcl1	YPDnc1	YPDnc2	YPDnc3	YPDnc4	EtOHnc1	EtOHnc2	Galnc1	Galnc2
11	SBE22	YHR103W	YPDcl2	YPDcl3	-	EtOHcl2	Galcl1	YPDnc1	YPDnc2	-	YPDnc4	EtOHnc1	EtOHnc2	Galnc1	Galnc2
11	TPO2	YGR138C	-	YPDcl3	EtOHcl1	EtOHcl2	Galcl1	YPDnc1	YPDnc2	-	YPDnc4	EtOHnc1	EtOHnc2	Galnc1	Galnc2
11	TEM1	YML064C	YPDcl2	YPDcl3	EtOHcl1	EtOHcl2	-	YPDnc1	YPDnc2	YPDnc3	YPDnc4	EtOHnc1	EtOHnc2	-	Galnc2
11	BET2	YPR176C	YPDcl2	YPDcl3	EtOHcl1	EtOHcl2	Galcl1	YPDnc1	YPDnc2	YPDnc3	YPDnc4	-	EtOHnc2	Galnc1	-
11		YAL037C-B	YPDcl2	YPDcl3	EtOHcl1	EtOHcl2	Galcl1	YPDnc1	YPDnc2	YPDnc3	YPDnc4	-	EtOHnc2	Galnc1	-
11	IRC25	YLR021W	YPDcl2	YPDcl3	EtOHcl1	EtOHcl2	Galcl1	-	YPDnc2	YPDnc3	YPDnc4	-	EtOHnc2	Galnc1	Galnc2
11		YKL036C	YPDcl2	YPDcl3	-	EtOHcl2	Galcl1	YPDnc1	YPDnc2	YPDnc3	YPDnc4	EtOHnc1	-	Galnc1	Galnc2
11	FMP40	YPL222W	YPDcl2	YPDcl3	EtOHcl1	EtOHcl2	Galcl1	YPDnc1	-	-	YPDnc4	EtOHnc1	EtOHnc2	Galnc1	Galnc2
11		YLR455W	YPDcl2	-	EtOHcl1	EtOHcl2	Galcl1	YPDnc1	YPDnc2	YPDnc3	YPDnc4	EtOHnc1	EtOHnc2	Galnc1	-
11		YER097W	YPDcl2	-	EtOHcl1	EtOHcl2	-	YPDnc1	YPDnc2	YPDnc3	YPDnc4	EtOHnc1	EtOHnc2	Galnc1	Galnc2
11	TAT1	YBR069C	-	YPDcl3	EtOHcl1	-	Galcl1	YPDnc1	YPDnc2	YPDnc3	YPDnc4	EtOHnc1	EtOHnc2	Galnc1	Galnc2
11	AMD1	YML035C	-	YPDcl3	EtOHcl1	-	Galcl1	YPDnc1	YPDnc2	YPDnc3	YPDnc4	EtOHnc1	EtOHnc2	Galnc1	Galnc2
11		YDR357C	YPDcl2	YPDcl3	EtOHcl1	-	-	YPDnc1	YPDnc2	YPDnc3	YPDnc4	EtOHnc1	EtOHnc2	Galnc1	Galnc2
11	RIX1	YHR197W	-	YPDcl3	EtOHcl1	EtOHcl2	-	YPDnc1	YPDnc2	YPDnc3	YPDnc4	EtOHnc1	EtOHnc2	Galnc1	Galnc2
11	CST6	YIL036W	-	YPDcl3	EtOHcl1	-	Galcl1	YPDnc1	YPDnc2	YPDnc3	YPDnc4	EtOHnc1	EtOHnc2	Galnc1	Galnc2
11		YOL086W-/	YPDcl2	YPDcl3	EtOHcl1	EtOHcl2	Galcl1	YPDnc1	YPDnc2	YPDnc3	YPDnc4	-	-	Galnc1	Galnc2
11	BTN2	YGR142W	YPDcl2	YPDcl3	EtOHcl1	-	Galcl1	YPDnc1	-	YPDnc3	YPDnc4	EtOHnc1	EtOHnc2	Galnc1	Galnc2

11	APT2	YDR441C	YPDcl2	YPDcl3	EtOHcl1	-	Galcl1	YPDnc1	YPDnc2	YPDnc3	YPDnc4	-	EtOHnc2	Galnc1	Galnc2
11	RPL17B	YJL177W	-	-	EtOHcl1	EtOHcl2	Galcl1	YPDnc1	YPDnc2	YPDnc3	YPDnc4	EtOHnc1	EtOHnc2	Galnc1	Galnc2
11	CDC8	YJR057W	YPDcl2	YPDcl3	EtOHcl1	EtOHcl2	Galcl1	YPDnc1	YPDnc2	-	YPDnc4	-	EtOHnc2	Galnc1	Galnc2
11	MSB2	YGR014W	YPDcl2	-	EtOHcl1	EtOHcl2	Galcl1	YPDnc1	YPDnc2	YPDnc3	YPDnc4	-	EtOHnc2	Galnc1	Galnc2
11	SOD2	YHR008C	-	-	EtOHcl1	EtOHcl2	Galcl1	YPDnc1	YPDnc2	YPDnc3	YPDnc4	EtOHnc1	EtOHnc2	Galnc1	Galnc2
11	SMI1	YGR229C	-	YPDcl3	-	EtOHcl2	Galcl1	YPDnc1	YPDnc2	YPDnc3	YPDnc4	EtOHnc1	EtOHnc2	Galnc1	Galnc2
11	KNH1	YDL049C	YPDcl2	YPDcl3	EtOHcl1	EtOHcl2	Galcl1	YPDnc1	-	YPDnc3	YPDnc4	EtOHnc1	-	Galnc1	Galnc2
11		YJL022W	-	YPDcl3	-	EtOHcl2	Galcl1	YPDnc1	YPDnc2	YPDnc3	YPDnc4	EtOHnc1	EtOHnc2	Galnc1	Galnc2
11	ZPR1	YGR211W	-	YPDcl3	EtOHcl1	-	Galcl1	YPDnc1	YPDnc2	YPDnc3	YPDnc4	EtOHnc1	EtOHnc2	Galnc1	Galnc2
11		YCL076W	YPDcl2	YPDcl3	-	EtOHcl2	Galcl1	YPDnc1	YPDnc2	YPDnc3	YPDnc4	EtOHnc1	-	Galnc1	Galnc2
11	SLA1	YBL007C	YPDcl2	YPDcl3	EtOHcl1	EtOHcl2	Galcl1	-	-	YPDnc3	YPDnc4	EtOHnc1	EtOHnc2	Galnc1	Galnc2
11	TGL2	YDR058C	YPDcl2	YPDcl3	-	EtOHcl2	Galcl1	YPDnc1	YPDnc2	YPDnc3	-	EtOHnc1	EtOHnc2	Galnc1	Galnc2
11	CFD1	YIL003W	YPDcl2	YPDcl3	-	-	Galcl1	YPDnc1	YPDnc2	YPDnc3	YPDnc4	EtOHnc1	EtOHnc2	Galnc1	Galnc2
11	ROM1	YGR070W	YPDcl2	-	EtOHcl1	EtOHcl2	Galcl1	YPDnc1	YPDnc2	YPDnc3	YPDnc4	EtOHnc1	EtOHnc2	-	Galnc2
11		YGL102C	YPDcl2	YPDcl3	EtOHcl1	EtOHcl2	Galcl1	-	YPDnc2	YPDnc3	YPDnc4	EtOHnc1	-	Galnc1	Galnc2
11	CBS2	YDR197W	-	YPDcl3	-	EtOHcl2	Galcl1	YPDnc1	YPDnc2	YPDnc3	YPDnc4	EtOHnc1	EtOHnc2	Galnc1	Galnc2
11	FAS1	YKL182W	YPDcl2	YPDcl3	EtOHcl1	EtOHcl2	Galcl1	YPDnc1	-	YPDnc3	-	EtOHnc1	EtOHnc2	Galnc1	Galnc2
11	STN1	YDR082W	-	YPDcl3	EtOHcl1	-	Galcl1	YPDnc1	YPDnc2	YPDnc3	YPDnc4	EtOHnc1	EtOHnc2	Galnc1	Galnc2
11	BUD27	YFL023W	-	YPDcl3	EtOHcl1	EtOHcl2	Galcl1	YPDnc1	YPDnc2	YPDnc3	-	EtOHnc1	EtOHnc2	Galnc1	Galnc2
11	ENO2	YHR174W	YPDcl2	YPDcl3	EtOHcl1	-	Galcl1	YPDnc1	YPDnc2	-	YPDnc4	EtOHnc1	EtOHnc2	Galnc1	Galnc2
11		YDR061W	-	-	EtOHcl1	EtOHcl2	Galcl1	YPDnc1	YPDnc2	YPDnc3	YPDnc4	EtOHnc1	EtOHnc2	Galnc1	Galnc2
11	MIS1	YBR084W	YPDcl2	-	EtOHcl1	EtOHcl2	Galcl1	YPDnc1	-	YPDnc3	YPDnc4	EtOHnc1	EtOHnc2	Galnc1	Galnc2
11		YKL111C	-	YPDcl3	EtOHcl1	-	Galcl1	YPDnc1	YPDnc2	YPDnc3	YPDnc4	EtOHnc1	EtOHnc2	Galnc1	Galnc2
11	PDA1	YER178W	YPDcl2	YPDcl3	EtOHcl1	-	Galcl1	YPDnc1	YPDnc2	YPDnc3	YPDnc4	EtOHnc1	EtOHnc2	Galnc1	-
11	KTR2	YKR061W	YPDcl2	YPDcl3	EtOHcl1	EtOHcl2	Galcl1	YPDnc1	-	YPDnc3	-	EtOHnc1	EtOHnc2	Galnc1	Galnc2
11	ACM1	YPL267W	YPDcl2	-	EtOHcl1	-	Galcl1	YPDnc1	YPDnc2	YPDnc3	YPDnc4	EtOHnc1	EtOHnc2	Galnc1	Galnc2
11	LAP2	YNL045W	YPDcl2	YPDcl3	EtOHcl1	EtOHcl2	Galcl1	YPDnc1	YPDnc2	YPDnc3	YPDnc4	-	EtOHnc2	-	Galnc2
11	SHQ1	YIL104C	YPDcl2	-	EtOHcl1	-	Galcl1	YPDnc1	YPDnc2	YPDnc3	YPDnc4	EtOHnc1	EtOHnc2	Galnc1	Galnc2
11	FMP27	YLR454W	YPDcl2	-	EtOHcl1	EtOHcl2	Galcl1	YPDnc1	YPDnc2	YPDnc3	YPDnc4	EtOHnc1	EtOHnc2	Galnc1	-
11	DOG2	YHR043C	YPDcl2	YPDcl3	EtOHcl1	EtOHcl2	Galcl1	YPDnc1	YPDnc2	-	YPDnc4	-	EtOHnc2	Galnc1	Galnc2
11		YHR070C-A	YPDcl2	YPDcl3	EtOHcl1	EtOHcl2	Galcl1	-	YPDnc2	YPDnc3	YPDnc4	EtOHnc1	EtOHnc2	Galnc1	-
11		YMR144W	YPDcl2	-	EtOHcl1	EtOHcl2	-	YPDnc1	YPDnc2	YPDnc3	YPDnc4	EtOHnc1	EtOHnc2	Galnc1	Galnc2
11	ATG15	YCR068W	YPDcl2	YPDcl3	-	EtOHcl2	Galcl1	YPDnc1	YPDnc2	-	YPDnc4	EtOHnc1	EtOHnc2	Galnc1	Galnc2
11		YGR151C	YPDcl2	YPDcl3	EtOHcl1	EtOHcl2	Galcl1	YPDnc1	YPDnc2	-	YPDnc4	EtOHnc1	-	Galnc1	Galnc2

11		YNL033W	YPDcl2	YPDcl3	EtOHcl1	EtOHcl2	Galcl1	YPDnc1	YPDnc2	-	-	EtOHnc1	EtOHnc2	Galnc1	Galnc2
11		YLL019W-A	-	YPDcl3	EtOHcl1	-	Galcl1	YPDnc1	YPDnc2	YPDnc3	YPDnc4	EtOHnc1	EtOHnc2	Galnc1	Galnc2
11	ECM12	YHR021W-	YPDcl2	YPDcl3	-	EtOHcl2	Galcl1	YPDnc1	YPDnc2	YPDnc3	-	EtOHnc1	EtOHnc2	Galnc1	Galnc2
11	APN2	YBL019W	YPDcl2	YPDcl3	-	EtOHcl2	Galcl1	YPDnc1	YPDnc2	YPDnc3	YPDnc4	EtOHnc1	-	Galnc1	Galnc2
11		YIL067C	-	-	EtOHcl1	EtOHcl2	Galcl1	YPDnc1	YPDnc2	YPDnc3	YPDnc4	EtOHnc1	EtOHnc2	Galnc1	Galnc2
11	YEF1	YEL041W	-	YPDcl3	EtOHcl1	EtOHcl2	Galcl1	YPDnc1	YPDnc2	YPDnc3	-	EtOHnc1	EtOHnc2	Galnc1	Galnc2
11	SEC66	YBR171W	-	YPDcl3	EtOHcl1	-	Galcl1	YPDnc1	YPDnc2	YPDnc3	YPDnc4	EtOHnc1	EtOHnc2	Galnc1	Galnc2
11		YLR317W	YPDcl2	YPDcl3	EtOHcl1	-	Galcl1	YPDnc1	YPDnc2	YPDnc3	YPDnc4	EtOHnc1	-	Galnc1	Galnc2
11	PRM7	YDL039C	YPDcl2	-	EtOHcl1	-	Galcl1	YPDnc1	YPDnc2	YPDnc3	YPDnc4	EtOHnc1	EtOHnc2	Galnc1	Galnc2
11	AVO1	YOL078W	YPDcl2	-	EtOHcl1	-	Galcl1	YPDnc1	YPDnc2	YPDnc3	YPDnc4	EtOHnc1	EtOHnc2	Galnc1	Galnc2
11	FUR4	YBR021W	YPDcl2	YPDcl3	EtOHcl1	EtOHcl2	Galcl1	YPDnc1	YPDnc2	YPDnc3	YPDnc4	EtOHnc1	-	-	Galnc2
11		YHR007C-A	-	YPDcl3	EtOHcl1	EtOHcl2	Galcl1	YPDnc1	YPDnc2	YPDnc3	YPDnc4	EtOHnc1	EtOHnc2	Galnc1	-
11	NOT5	YPR072W	-	-	EtOHcl1	EtOHcl2	Galcl1	YPDnc1	YPDnc2	YPDnc3	YPDnc4	EtOHnc1	EtOHnc2	Galnc1	Galnc2
11		YFR012W-	-	YPDcl3	EtOHcl1	-	Galcl1	YPDnc1	YPDnc2	YPDnc3	YPDnc4	EtOHnc1	EtOHnc2	Galnc1	Galnc2
11	SHU1	YHL006C	YPDcl2	YPDcl3	EtOHcl1	EtOHcl2	Galcl1	YPDnc1	YPDnc2	YPDnc3	-	EtOHnc1	-	Galnc1	Galnc2
11	HUA1	YGR268C	YPDcl2	YPDcl3	-	-	Galcl1	YPDnc1	YPDnc2	YPDnc3	YPDnc4	EtOHnc1	EtOHnc2	Galnc1	Galnc2
11	STR2	YJR130C	YPDcl2	YPDcl3	-	EtOHcl2	Galcl1	YPDnc1	YPDnc2	YPDnc3	YPDnc4	EtOHnc1	-	Galnc1	Galnc2
11	ELM1	YKL048C	YPDcl2	YPDcl3	EtOHcl1	EtOHcl2	Galcl1	YPDnc1	YPDnc2	YPDnc3	YPDnc4	EtOHnc1	EtOHnc2	-	-
11	SYM1	YLR251W	YPDcl2	YPDcl3	EtOHcl1	EtOHcl2	Galcl1	-	YPDnc2	-	YPDnc4	EtOHnc1	EtOHnc2	Galnc1	Galnc2
11	VMA22	YHR060W	YPDcl2	-	EtOHcl1	-	Galcl1	YPDnc1	YPDnc2	YPDnc3	YPDnc4	EtOHnc1	EtOHnc2	Galnc1	Galnc2
11	MUK1	YPL070W	YPDcl2	YPDcl3	EtOHcl1	EtOHcl2	Galcl1	YPDnc1	YPDnc2	YPDnc3	YPDnc4	-	EtOHnc2	-	Galnc2
11	CIS3	YJL158C	-	YPDcl3	EtOHcl1	-	Galcl1	YPDnc1	YPDnc2	YPDnc3	YPDnc4	EtOHnc1	EtOHnc2	Galnc1	Galnc2
11	MRP1	YDR347W	YPDcl2	YPDcl3	-	-	Galcl1	YPDnc1	YPDnc2	YPDnc3	YPDnc4	EtOHnc1	EtOHnc2	Galnc1	Galnc2
11	SER2	YGR208W	YPDcl2	-	EtOHcl1	EtOHcl2	-	YPDnc1	YPDnc2	YPDnc3	YPDnc4	EtOHnc1	EtOHnc2	Galnc1	Galnc2
11	GUS1	YGL245W	YPDcl2	-	EtOHcl1	EtOHcl2	Galcl1	YPDnc1	YPDnc2	YPDnc3	YPDnc4	EtOHnc1	-	Galnc1	Galnc2
11	YTA7	YGR270W	YPDcl2	YPDcl3	EtOHcl1	EtOHcl2	Galcl1	YPDnc1	YPDnc2	YPDnc3	YPDnc4	-	EtOHnc2	Galnc1	-
11	MRPL37	YBR268W	YPDcl2	YPDcl3	EtOHcl1	EtOHcl2	Galcl1	YPDnc1	-	-	YPDnc4	EtOHnc1	EtOHnc2	Galnc1	Galnc2
11		YJR112W-A	-	YPDcl3	EtOHcl1	EtOHcl2	Galcl1	YPDnc1	YPDnc2	YPDnc3	-	EtOHnc1	EtOHnc2	Galnc1	Galnc2
11	GAT1	YFL021W	YPDcl2	YPDcl3	-	EtOHcl2	Galcl1	YPDnc1	YPDnc2	YPDnc3	YPDnc4	EtOHnc1	EtOHnc2	Galnc1	-
11		YBL039C-A	YPDcl2	-	EtOHcl1	EtOHcl2	Galcl1	YPDnc1	-	YPDnc3	YPDnc4	EtOHnc1	EtOHnc2	Galnc1	Galnc2
11	MRPL31	YKL138C	YPDcl2	YPDcl3	EtOHcl1	EtOHcl2	Galcl1	-	YPDnc2	YPDnc3	YPDnc4	EtOHnc1	EtOHnc2	-	Galnc2
11	ELA1	YNL230C	YPDcl2	YPDcl3	EtOHcl1	EtOHcl2	Galcl1	YPDnc1	YPDnc2	-	YPDnc4	EtOHnc1	EtOHnc2	Galnc1	-
11		YPL088W	YPDcl2	YPDcl3	EtOHcl1	-	Galcl1	YPDnc1	YPDnc2	YPDnc3	YPDnc4	EtOHnc1	-	Galnc1	Galnc2
11	LIA1	YJR070C	YPDcl2	-	EtOHcl1	-	Galcl1	YPDnc1	YPDnc2	YPDnc3	YPDnc4	EtOHnc1	EtOHnc2	Galnc1	Galnc2

11	CYS4	YGR155W	-	YPDcl3	EtOHcl1	EtOHcl2	Galcl1	YPDnc1	-	YPDnc3	YPDnc4	EtOHnc1	EtOHnc2	Galnc1	Galnc2
11	BDP1	YNL039W	YPDcl2	YPDcl3	EtOHcl1	EtOHcl2	Galcl1	-	YPDnc2	YPDnc3	YPDnc4	EtOHnc1	-	Galnc1	Galnc2
11		YHL034W-A	-	-	EtOHcl1	EtOHcl2	Galcl1	YPDnc1	YPDnc2	YPDnc3	YPDnc4	EtOHnc1	EtOHnc2	Galnc1	Galnc2
11		YLR243W	-	-	EtOHcl1	EtOHcl2	Galcl1	YPDnc1	YPDnc2	YPDnc3	YPDnc4	EtOHnc1	EtOHnc2	Galnc1	Galnc2
11	HAM1	YJR069C	-	-	EtOHcl1	EtOHcl2	Galcl1	YPDnc1	YPDnc2	YPDnc3	YPDnc4	EtOHnc1	EtOHnc2	Galnc1	Galnc2
11	RNR3	YIL066C	-	-	EtOHcl1	EtOHcl2	Galcl1	YPDnc1	YPDnc2	YPDnc3	YPDnc4	EtOHnc1	EtOHnc2	Galnc1	Galnc2
11	CTR86	YCR054C	YPDcl2	-	EtOHcl1	-	Galcl1	YPDnc1	YPDnc2	YPDnc3	YPDnc4	EtOHnc1	EtOHnc2	Galnc1	Galnc2
11	TAD1	YGL243W	YPDcl2	YPDcl3	EtOHcl1	-	Galcl1	YPDnc1	YPDnc2	YPDnc3	YPDnc4	EtOHnc1	-	Galnc1	Galnc2
11	SEC39	YLR440C	YPDcl2	YPDcl3	-	EtOHcl2	Galcl1	YPDnc1	YPDnc2	YPDnc3	YPDnc4	-	EtOHnc2	Galnc1	Galnc2
11	SIZ1	YDR409W	-	YPDcl3	EtOHcl1	-	Galcl1	YPDnc1	YPDnc2	YPDnc3	YPDnc4	EtOHnc1	EtOHnc2	Galnc1	Galnc2
11		YKL118W	YPDcl2	YPDcl3	EtOHcl1	EtOHcl2	Galcl1	YPDnc1	-	-	YPDnc4	EtOHnc1	EtOHnc2	Galnc1	Galnc2
11	CDC48	YDL126C	-	YPDcl3	EtOHcl1	EtOHcl2	Galcl1	YPDnc1	YPDnc2	YPDnc3	YPDnc4	EtOHnc1	-	Galnc1	Galnc2
11	UBX6	YJL048C	-	YPDcl3	EtOHcl1	-	Galcl1	YPDnc1	YPDnc2	YPDnc3	YPDnc4	EtOHnc1	EtOHnc2	Galnc1	Galnc2
11	MED8	YBR193C	YPDcl2	-	EtOHcl1	-	Galcl1	YPDnc1	YPDnc2	YPDnc3	YPDnc4	EtOHnc1	EtOHnc2	Galnc1	Galnc2
11	NSL1	YPL233W	-	YPDcl3	EtOHcl1	-	Galcl1	YPDnc1	YPDnc2	YPDnc3	YPDnc4	EtOHnc1	EtOHnc2	Galnc1	Galnc2
11		YPR015C	YPDcl2	YPDcl3	EtOHcl1	EtOHcl2	Galcl1	YPDnc1	-	YPDnc3	-	EtOHnc1	EtOHnc2	Galnc1	Galnc2
11	MBF1	YOR298C-A	YPDcl2	YPDcl3	EtOHcl1	EtOHcl2	Galcl1	YPDnc1	-	YPDnc3	-	EtOHnc1	EtOHnc2	Galnc1	Galnc2
11	COX18	YGR062C	-	-	EtOHcl1	EtOHcl2	Galcl1	YPDnc1	YPDnc2	YPDnc3	YPDnc4	EtOHnc1	EtOHnc2	Galnc1	Galnc2
11	IRC9	YJL142C	YPDcl2	YPDcl3	-	EtOHcl2	Galcl1	YPDnc1	YPDnc2	-	YPDnc4	EtOHnc1	EtOHnc2	Galnc1	Galnc2
11	GDS1	YOR355W	-	YPDcl3	EtOHcl1	EtOHcl2	-	YPDnc1	YPDnc2	YPDnc3	YPDnc4	EtOHnc1	EtOHnc2	Galnc1	Galnc2
11		YNL013C	YPDcl2	YPDcl3	EtOHcl1	EtOHcl2	Galcl1	YPDnc1	YPDnc2	YPDnc3	YPDnc4	-	-	Galnc1	Galnc2
11	VCX1	YDL128W	-	YPDcl3	EtOHcl1	EtOHcl2	-	YPDnc1	YPDnc2	YPDnc3	YPDnc4	EtOHnc1	EtOHnc2	Galnc1	Galnc2
11		YBR056C-B	YPDcl2	-	EtOHcl1	EtOHcl2	Galcl1	YPDnc1	YPDnc2	YPDnc3	YPDnc4	EtOHnc1	EtOHnc2	-	Galnc2
11		YOR262W	-	-	EtOHcl1	EtOHcl2	Galcl1	YPDnc1	YPDnc2	YPDnc3	YPDnc4	EtOHnc1	EtOHnc2	Galnc1	Galnc2
11	MRP10	YDL045W-A	YPDcl2	YPDcl3	EtOHcl1	-	Galcl1	YPDnc1	YPDnc2	YPDnc3	YPDnc4	-	EtOHnc2	Galnc1	Galnc2
11		YLL059C	YPDcl2	YPDcl3	EtOHcl1	EtOHcl2	Galcl1	YPDnc1	YPDnc2	YPDnc3	-	EtOHnc1	-	Galnc1	Galnc2
11	RPI1	YIL119C	YPDcl2	YPDcl3	EtOHcl1	-	Galcl1	YPDnc1	YPDnc2	YPDnc3	-	EtOHnc1	EtOHnc2	Galnc1	Galnc2
11		YMR290W-	-	YPDcl3	EtOHcl1	-	Galcl1	YPDnc1	YPDnc2	YPDnc3	YPDnc4	EtOHnc1	EtOHnc2	Galnc1	Galnc2
11	GAS2	YLR343W	YPDcl2	YPDcl3	-	EtOHcl2	Galcl1	YPDnc1	YPDnc2	YPDnc3	YPDnc4	EtOHnc1	EtOHnc2	Galnc1	-
11	SRX1	YKL086W	YPDcl2	YPDcl3	EtOHcl1	-	-	YPDnc1	YPDnc2	YPDnc3	YPDnc4	EtOHnc1	EtOHnc2	Galnc1	Galnc2
11		YGL010W	YPDcl2	YPDcl3	EtOHcl1	EtOHcl2	Galcl1	YPDnc1	-	YPDnc3	-	EtOHnc1	EtOHnc2	Galnc1	Galnc2
11	RPB2	YOR151C	YPDcl2	YPDcl3	EtOHcl1	EtOHcl2	Galcl1	YPDnc1	YPDnc2	YPDnc3	-	EtOHnc1	-	Galnc1	Galnc2
11	YPS3	YLR121C	YPDcl2	YPDcl3	EtOHcl1	-	Galcl1	YPDnc1	YPDnc2	YPDnc3	-	EtOHnc1	EtOHnc2	Galnc1	Galnc2
11	DOS2	YDR068W	YPDcl2	YPDcl3	EtOHcl1	-	Galcl1	YPDnc1	YPDnc2	YPDnc3	YPDnc4	EtOHnc1	-	Galnc1	Galnc2

11	ARP10	YDR106W	-	-	EtOHcl1	EtOHcl2	Galcl1	YPDnc1	YPDnc2	YPDnc3	YPDnc4	EtOHnc1	EtOHnc2	Galnc1	Galnc2
11	REH1	YLR387C	YPDcl2	YPDcl3	EtOHcl1	EtOHcl2	Galcl1	YPDnc1	-	YPDnc3	YPDnc4	EtOHnc1	EtOHnc2	Galnc1	-
11	SPC29	YPL124W	YPDcl2	YPDcl3	-	-	Galcl1	YPDnc1	YPDnc2	YPDnc3	YPDnc4	EtOHnc1	EtOHnc2	Galnc1	Galnc2
11		YER152C	YPDcl2	-	EtOHcl1	-	Galcl1	YPDnc1	YPDnc2	YPDnc3	YPDnc4	EtOHnc1	EtOHnc2	Galnc1	Galnc2
11	WSC3	YOL105C	YPDcl2	-	-	EtOHcl2	Galcl1	YPDnc1	YPDnc2	YPDnc3	YPDnc4	EtOHnc1	EtOHnc2	Galnc1	Galnc2
11		YLR334C	-	YPDcl3	EtOHcl1	EtOHcl2	Galcl1	YPDnc1	YPDnc2	YPDnc3	-	EtOHnc1	EtOHnc2	Galnc1	Galnc2
11	RLP24	YLR009W	YPDcl2	-	EtOHcl1	EtOHcl2	Galcl1	YPDnc1	YPDnc2	YPDnc3	YPDnc4	-	EtOHnc2	Galnc1	Galnc2
11	OST3	YOR085W	YPDcl2	-	-	EtOHcl2	Galcl1	YPDnc1	YPDnc2	YPDnc3	YPDnc4	EtOHnc1	EtOHnc2	Galnc1	Galnc2
11	NST1	YNL091W	-	YPDcl3	EtOHcl1	EtOHcl2	Galcl1	YPDnc1	YPDnc2	YPDnc3	YPDnc4	EtOHnc1	EtOHnc2	-	Galnc2
11	TAT2	YOL020W	-	YPDcl3	EtOHcl1	EtOHcl2	Galcl1	YPDnc1	YPDnc2	YPDnc3	-	EtOHnc1	EtOHnc2	Galnc1	Galnc2
11	ECM8	YBR076W	YPDcl2	YPDcl3	EtOHcl1	-	-	YPDnc1	YPDnc2	YPDnc3	YPDnc4	EtOHnc1	EtOHnc2	Galnc1	Galnc2
11	THG1	YGR024C	-	YPDcl3	EtOHcl1	-	Galcl1	YPDnc1	YPDnc2	YPDnc3	YPDnc4	EtOHnc1	EtOHnc2	Galnc1	Galnc2
11	RAV1	YJR033C	YPDcl2	YPDcl3	EtOHcl1	EtOHcl2	Galcl1	YPDnc1	YPDnc2	YPDnc3	YPDnc4	EtOHnc1	EtOHnc2	-	-
11		YMR253C	YPDcl2	YPDcl3	EtOHcl1	-	Galcl1	YPDnc1	YPDnc2	YPDnc3	YPDnc4	EtOHnc1	-	Galnc1	Galnc2
11	REV1	YOR346W	YPDcl2	YPDcl3	-	EtOHcl2	Galcl1	YPDnc1	YPDnc2	YPDnc3	YPDnc4	EtOHnc1	-	Galnc1	Galnc2
11	SRL3	YKR091W	YPDcl2	YPDcl3	EtOHcl1	EtOHcl2	Galcl1	YPDnc1	YPDnc2	YPDnc3	YPDnc4	-	-	Galnc1	Galnc2
11	RIC1	YLR039C	YPDcl2	-	EtOHcl1	-	Galcl1	YPDnc1	YPDnc2	YPDnc3	YPDnc4	EtOHnc1	EtOHnc2	Galnc1	Galnc2
11	GUT1	YHL032C	YPDcl2	YPDcl3	EtOHcl1	-	-	YPDnc1	YPDnc2	YPDnc3	YPDnc4	EtOHnc1	EtOHnc2	Galnc1	Galnc2
11	HEM14	YER014W	YPDcl2	YPDcl3	EtOHcl1	EtOHcl2	Galcl1	-	-	YPDnc3	YPDnc4	EtOHnc1	EtOHnc2	Galnc1	Galnc2
11	PRY1	YJL079C	-	YPDcl3	EtOHcl1	EtOHcl2	-	YPDnc1	YPDnc2	YPDnc3	YPDnc4	EtOHnc1	EtOHnc2	Galnc1	Galnc2
11	PRB1	YEL060C	-	YPDcl3	EtOHcl1	-	Galcl1	YPDnc1	YPDnc2	YPDnc3	YPDnc4	EtOHnc1	EtOHnc2	Galnc1	Galnc2
11	RPL26A	YLR344W	YPDcl2	YPDcl3	-	EtOHcl2	Galcl1	YPDnc1	YPDnc2	YPDnc3	YPDnc4	EtOHnc1	-	Galnc1	Galnc2
11	ARL3	YPL051W	YPDcl2	-	EtOHcl1	EtOHcl2	Galcl1	YPDnc1	YPDnc2	-	YPDnc4	EtOHnc1	EtOHnc2	Galnc1	Galnc2
11	YBT1	YLL048C	YPDcl2	YPDcl3	-	EtOHcl2	Galcl1	YPDnc1	YPDnc2	YPDnc3	-	EtOHnc1	EtOHnc2	Galnc1	Galnc2
11	PPG1	YNR032W	YPDcl2	YPDcl3	-	EtOHcl2	Galcl1	YPDnc1	YPDnc2	-	YPDnc4	EtOHnc1	EtOHnc2	Galnc1	Galnc2
11	SPS4	YOR313C	YPDcl2	-	EtOHcl1	-	Galcl1	YPDnc1	YPDnc2	YPDnc3	YPDnc4	EtOHnc1	EtOHnc2	Galnc1	Galnc2
11	NUF2	YOL069W	-	YPDcl3	EtOHcl1	EtOHcl2	-	YPDnc1	YPDnc2	YPDnc3	YPDnc4	EtOHnc1	EtOHnc2	Galnc1	Galnc2
11	CYB5	YNL111C	YPDcl2	YPDcl3	EtOHcl1	EtOHcl2	Galcl1	YPDnc1	YPDnc2	YPDnc3	YPDnc4	EtOHnc1	EtOHnc2	-	-
11		YOR008W-	-	YPDcl3	EtOHcl1	-	Galcl1	YPDnc1	YPDnc2	YPDnc3	YPDnc4	EtOHnc1	EtOHnc2	Galnc1	Galnc2
11		YDR415C	YPDcl2	-	EtOHcl1	-	Galcl1	YPDnc1	YPDnc2	YPDnc3	YPDnc4	EtOHnc1	EtOHnc2	Galnc1	Galnc2
11	SED1	YDR077W	YPDcl2	YPDcl3	EtOHcl1	EtOHcl2	Galcl1	-	YPDnc2	YPDnc3	YPDnc4	EtOHnc1	EtOHnc2	Galnc1	-
11	TDA10	YGR205W	YPDcl2	YPDcl3	EtOHcl1	EtOHcl2	Galcl1	-	YPDnc2	YPDnc3	YPDnc4	-	EtOHnc2	Galnc1	Galnc2
11		YOR186C-A	YPDcl2	YPDcl3	-	-	Galcl1	YPDnc1	YPDnc2	YPDnc3	YPDnc4	EtOHnc1	EtOHnc2	Galnc1	Galnc2
11	COX7	YMR256C	YPDcl2	YPDcl3	EtOHcl1	-	Galcl1	YPDnc1	YPDnc2	YPDnc3	YPDnc4	-	EtOHnc2	Galnc1	Galnc2

11	NPY1	YGL067W	YPDcl2	YPDcl3	EtOHcl1	EtOHcl2	Galcl1	-	YPDnc2	YPDnc3	YPDnc4	EtOHnc1	EtOHnc2	-	Galnc2
11	NCS2	YNL119W	YPDcl2	YPDcl3	EtOHcl1	EtOHcl2	Galcl1	YPDnc1	YPDnc2	YPDnc3	YPDnc4	EtOHnc1	EtOHnc2	-	-
11	YML6	YML025C	YPDcl2	YPDcl3	EtOHcl1	-	Galcl1	-	YPDnc2	YPDnc3	YPDnc4	EtOHnc1	EtOHnc2	Galnc1	Galnc2
11	MTC1	YJL123C	-	-	EtOHcl1	EtOHcl2	Galcl1	YPDnc1	YPDnc2	YPDnc3	YPDnc4	EtOHnc1	EtOHnc2	Galnc1	Galnc2
11		YPR027C	YPDcl2	YPDcl3	EtOHcl1	EtOHcl2	Galcl1	YPDnc1	YPDnc2	-	-	EtOHnc1	EtOHnc2	Galnc1	Galnc2
11	NAM7	YMR080C	YPDcl2	YPDcl3	EtOHcl1	EtOHcl2	Galcl1	YPDnc1	YPDnc2	-	YPDnc4	EtOHnc1	-	Galnc1	Galnc2
11	HRT3	YLR097C	-	-	EtOHcl1	EtOHcl2	Galcl1	YPDnc1	YPDnc2	YPDnc3	YPDnc4	EtOHnc1	EtOHnc2	Galnc1	Galnc2
11	SPT3	YDR392W	YPDcl2	-	EtOHcl1	-	Galcl1	YPDnc1	YPDnc2	YPDnc3	YPDnc4	EtOHnc1	EtOHnc2	Galnc1	Galnc2
11		YGR176W	-	-	EtOHcl1	EtOHcl2	Galcl1	YPDnc1	YPDnc2	YPDnc3	YPDnc4	EtOHnc1	EtOHnc2	Galnc1	Galnc2
11		YJR146W	-	YPDcl3	EtOHcl1	EtOHcl2	-	YPDnc1	YPDnc2	YPDnc3	YPDnc4	EtOHnc1	EtOHnc2	Galnc1	Galnc2
11	SER33	YIL074C	YPDcl2	-	EtOHcl1	-	Galcl1	YPDnc1	YPDnc2	YPDnc3	YPDnc4	EtOHnc1	EtOHnc2	Galnc1	Galnc2
11	GPI14	YJR013W	-	-	EtOHcl1	EtOHcl2	Galcl1	YPDnc1	YPDnc2	YPDnc3	YPDnc4	EtOHnc1	EtOHnc2	Galnc1	Galnc2
11	FBP1	YLR377C	-	YPDcl3	EtOHcl1	EtOHcl2	Galcl1	YPDnc1	YPDnc2	YPDnc3	YPDnc4	EtOHnc1	-	Galnc1	Galnc2
11	ANB1	YJR047C	YPDcl2	YPDcl3	EtOHcl1	-	-	YPDnc1	YPDnc2	YPDnc3	YPDnc4	EtOHnc1	EtOHnc2	Galnc1	Galnc2
11		YPR126C	YPDcl2	YPDcl3	EtOHcl1	-	Galcl1	YPDnc1	-	YPDnc3	YPDnc4	EtOHnc1	EtOHnc2	Galnc1	Galnc2
11	NCE101	YJL205C	YPDcl2	YPDcl3	EtOHcl1	EtOHcl2	Galcl1	YPDnc1	YPDnc2	-	YPDnc4	EtOHnc1	-	Galnc1	Galnc2
11	TPS1	YBR126C	YPDcl2	YPDcl3	EtOHcl1	EtOHcl2	Galcl1	YPDnc1	YPDnc2	YPDnc3	YPDnc4	-	EtOHnc2	-	Galnc2
11	ARP4	YJL081C	-	YPDcl3	EtOHcl1	EtOHcl2	Galcl1	YPDnc1	-	YPDnc3	YPDnc4	EtOHnc1	EtOHnc2	Galnc1	Galnc2
11	CDC53	YDL132W	YPDcl2	YPDcl3	EtOHcl1	EtOHcl2	Galcl1	-	YPDnc2	YPDnc3	YPDnc4	-	EtOHnc2	Galnc1	Galnc2
11	GAS4	YOL132W	YPDcl2	YPDcl3	-	EtOHcl2	Galcl1	YPDnc1	YPDnc2	YPDnc3	YPDnc4	EtOHnc1	-	Galnc1	Galnc2
11	DAS2	YDR020C	YPDcl2	YPDcl3	EtOHcl1	-	Galcl1	YPDnc1	YPDnc2	YPDnc3	-	EtOHnc1	EtOHnc2	Galnc1	Galnc2
11	RSM28	YDR494W	YPDcl2	YPDcl3	EtOHcl1	-	Galcl1	-	YPDnc2	YPDnc3	YPDnc4	EtOHnc1	EtOHnc2	Galnc1	Galnc2
11	DBP5	YOR046C	YPDcl2	-	EtOHcl1	EtOHcl2	Galcl1	YPDnc1	YPDnc2	-	YPDnc4	EtOHnc1	EtOHnc2	Galnc1	Galnc2
11		YGR067C	-	YPDcl3	EtOHcl1	EtOHcl2	Galcl1	YPDnc1	YPDnc2	YPDnc3	-	EtOHnc1	EtOHnc2	Galnc1	Galnc2
11		YBL012C	-	YPDcl3	EtOHcl1	EtOHcl2	Galcl1	YPDnc1	YPDnc2	YPDnc3	-	EtOHnc1	EtOHnc2	Galnc1	Galnc2
11		YJL160C	YPDcl2	YPDcl3	EtOHcl1	EtOHcl2	-	YPDnc1	YPDnc2	-	YPDnc4	EtOHnc1	EtOHnc2	Galnc1	Galnc2
11	TSR3	YOR006C	YPDcl2	YPDcl3	EtOHcl1	-	Galcl1	YPDnc1	YPDnc2	YPDnc3	YPDnc4	EtOHnc1	-	Galnc1	Galnc2
11	RCL1	YOL010W	YPDcl2	YPDcl3	EtOHcl1	EtOHcl2	Galcl1	-	YPDnc2	YPDnc3	YPDnc4	-	EtOHnc2	Galnc1	Galnc2
11	AIM29	YKR074W	-	-	EtOHcl1	EtOHcl2	Galcl1	YPDnc1	YPDnc2	YPDnc3	YPDnc4	EtOHnc1	EtOHnc2	Galnc1	Galnc2
11	KIC1	YHR102W	-	YPDcl3	EtOHcl1	EtOHcl2	Galcl1	YPDnc1	-	YPDnc3	YPDnc4	EtOHnc1	EtOHnc2	Galnc1	Galnc2
11	TAZ1	YPR140W	-	YPDcl3	-	EtOHcl2	Galcl1	YPDnc1	YPDnc2	YPDnc3	YPDnc4	EtOHnc1	EtOHnc2	Galnc1	Galnc2
11	EMC6	YLL014W	YPDcl2	-	EtOHcl1	-	Galcl1	YPDnc1	YPDnc2	YPDnc3	YPDnc4	EtOHnc1	EtOHnc2	Galnc1	Galnc2
11	YTM1	YOR272W	-	YPDcl3	EtOHcl1	EtOHcl2	Galcl1	YPDnc1	YPDnc2	YPDnc3	YPDnc4	EtOHnc1	-	Galnc1	Galnc2
11		YGL006W-/	YPDcl2	YPDcl3	-	EtOHcl2	Galcl1	YPDnc1	YPDnc2	YPDnc3	-	EtOHnc1	EtOHnc2	Galnc1	Galnc2

11		YJR129C	YPDcl2	-	EtOHcl1	-	Galcl1	YPDnc1	YPDnc2	YPDnc3	YPDnc4	EtOHnc1	EtOHnc2	Galnc1	Galnc2
11	TVP15	YDR100W	-	-	EtOHcl1	EtOHcl2	Galcl1	YPDnc1	YPDnc2	YPDnc3	YPDnc4	EtOHnc1	EtOHnc2	Galnc1	Galnc2
11	SUP35	YDR172W	YPDcl2	YPDcl3	EtOHcl1	-	Galcl1	YPDnc1	YPDnc2	YPDnc3	-	EtOHnc1	EtOHnc2	Galnc1	Galnc2
11	AHT1	YHR093W	-	YPDcl3	EtOHcl1	EtOHcl2	Galcl1	YPDnc1	YPDnc2	YPDnc3	YPDnc4	EtOHnc1	EtOHnc2	Galnc1	-
11	MST27	YGL051W	YPDcl2	-	EtOHcl1	EtOHcl2	Galcl1	YPDnc1	YPDnc2	YPDnc3	YPDnc4	EtOHnc1	-	Galnc1	Galnc2
11	ATG29	YPL166W	YPDcl2	YPDcl3	-	-	Galcl1	YPDnc1	YPDnc2	YPDnc3	YPDnc4	EtOHnc1	EtOHnc2	Galnc1	Galnc2
11	CHS6	YJL099W	-	YPDcl3	EtOHcl1	EtOHcl2	-	YPDnc1	YPDnc2	YPDnc3	YPDnc4	EtOHnc1	EtOHnc2	Galnc1	Galnc2
11	PRE2	YPR103W	YPDcl2	YPDcl3	-	EtOHcl2	Galcl1	YPDnc1	YPDnc2	YPDnc3	-	EtOHnc1	EtOHnc2	Galnc1	Galnc2
11	IBA57	YJR122W	YPDcl2	YPDcl3	EtOHcl1	-	Galcl1	YPDnc1	YPDnc2	YPDnc3	YPDnc4	EtOHnc1	EtOHnc2	-	Galnc2
11	ROK1	YGL171W	YPDcl2	YPDcl3	-	-	Galcl1	YPDnc1	YPDnc2	YPDnc3	YPDnc4	EtOHnc1	EtOHnc2	Galnc1	Galnc2
11	NUD1	YOR373W	YPDcl2	YPDcl3	EtOHcl1	-	-	YPDnc1	YPDnc2	YPDnc3	YPDnc4	EtOHnc1	EtOHnc2	Galnc1	Galnc2
11		YLR406C-A	-	YPDcl3	EtOHcl1	EtOHcl2	Galcl1	YPDnc1	YPDnc2	YPDnc3	-	EtOHnc1	EtOHnc2	Galnc1	Galnc2
11	CTR2	YHR175W	YPDcl2	YPDcl3	EtOHcl1	-	Galcl1	YPDnc1	YPDnc2	YPDnc3	YPDnc4	EtOHnc1	-	Galnc1	Galnc2
11	RRT2	YBR246W	YPDcl2	YPDcl3	EtOHcl1	EtOHcl2	Galcl1	YPDnc1	YPDnc2	-	-	EtOHnc1	EtOHnc2	Galnc1	Galnc2
11	RPL17A	YKL180W	YPDcl2	YPDcl3	EtOHcl1	EtOHcl2	Galcl1	-	YPDnc2	YPDnc3	YPDnc4	-	EtOHnc2	Galnc1	Galnc2
11	UBP7	YIL156W	-	YPDcl3	EtOHcl1	EtOHcl2	-	YPDnc1	YPDnc2	YPDnc3	YPDnc4	EtOHnc1	EtOHnc2	Galnc1	Galnc2
11	TYR1	YBR166C	YPDcl2	YPDcl3	EtOHcl1	EtOHcl2	Galcl1	-	YPDnc2	YPDnc3	YPDnc4	-	EtOHnc2	Galnc1	Galnc2
11		YDR271C	YPDcl2	YPDcl3	EtOHcl1	-	Galcl1	YPDnc1	YPDnc2	YPDnc3	YPDnc4	EtOHnc1	-	Galnc1	Galnc2
11		YDR433W	YPDcl2	-	EtOHcl1	EtOHcl2	Galcl1	-	YPDnc2	YPDnc3	YPDnc4	EtOHnc1	EtOHnc2	Galnc1	Galnc2
11	LIN1	YHR156C	YPDcl2	YPDcl3	EtOHcl1	EtOHcl2	Galcl1	-	YPDnc2	YPDnc3	YPDnc4	-	EtOHnc2	Galnc1	Galnc2
11		YMR230W-	YPDcl2	-	EtOHcl1	-	Galcl1	YPDnc1	YPDnc2	YPDnc3	YPDnc4	EtOHnc1	EtOHnc2	Galnc1	Galnc2
11	PTK2	YJR059W	-	YPDcl3	EtOHcl1	EtOHcl2	-	YPDnc1	YPDnc2	YPDnc3	YPDnc4	EtOHnc1	EtOHnc2	Galnc1	Galnc2
11	DBP2	YNL112W	-	YPDcl3	EtOHcl1	EtOHcl2	Galcl1	YPDnc1	YPDnc2	YPDnc3	YPDnc4	-	EtOHnc2	Galnc1	Galnc2
11	SMY2	YBR172C	-	YPDcl3	EtOHcl1	-	Galcl1	YPDnc1	YPDnc2	YPDnc3	YPDnc4	EtOHnc1	EtOHnc2	Galnc1	Galnc2
11	SCP160	YJL080C	-	YPDcl3	EtOHcl1	-	Galcl1	YPDnc1	YPDnc2	YPDnc3	YPDnc4	EtOHnc1	EtOHnc2	Galnc1	Galnc2
11	STE13	YOR219C	YPDcl2	YPDcl3	EtOHcl1	EtOHcl2	Galcl1	YPDnc1	YPDnc2	-	YPDnc4	EtOHnc1	-	Galnc1	Galnc2
11	RAD61	YDR014W	YPDcl2	-	EtOHcl1	-	Galcl1	YPDnc1	YPDnc2	YPDnc3	YPDnc4	EtOHnc1	EtOHnc2	Galnc1	Galnc2
11	NBP1	YLR457C	YPDcl2	-	EtOHcl1	-	Galcl1	YPDnc1	YPDnc2	YPDnc3	YPDnc4	EtOHnc1	EtOHnc2	Galnc1	Galnc2
11	GIT1	YCR098C	YPDcl2	-	EtOHcl1	-	Galcl1	YPDnc1	YPDnc2	YPDnc3	YPDnc4	EtOHnc1	EtOHnc2	Galnc1	Galnc2
11	PHO8	YDR481C	YPDcl2	YPDcl3	-	EtOHcl2	Galcl1	YPDnc1	YPDnc2	YPDnc3	-	EtOHnc1	EtOHnc2	Galnc1	Galnc2
11		YNL150W	-	YPDcl3	EtOHcl1	EtOHcl2	Galcl1	YPDnc1	YPDnc2	-	YPDnc4	EtOHnc1	EtOHnc2	Galnc1	Galnc2
11	BUD8	YLR353W	-	-	EtOHcl1	EtOHcl2	Galcl1	YPDnc1	YPDnc2	YPDnc3	YPDnc4	EtOHnc1	EtOHnc2	Galnc1	Galnc2
11	BAT1	YHR208W	YPDcl2	YPDcl3	EtOHcl1	-	Galcl1	YPDnc1	YPDnc2	YPDnc3	-	EtOHnc1	EtOHnc2	Galnc1	Galnc2
11	PRE7	YBL041W	-	YPDcl3	EtOHcl1	EtOHcl2	Galcl1	-	YPDnc2	YPDnc3	YPDnc4	EtOHnc1	EtOHnc2	Galnc1	Galnc2

11	TOM7	YNL070W	YPDcl2	-	EtOHcl1	EtOHcl2	Galcl1	YPDnc1	YPDnc2	-	YPDnc4	EtOHnc1	EtOHnc2	Galnc1	Galnc2
11	RPS7A	YOR096W	YPDcl2	-	EtOHcl1	-	Galcl1	YPDnc1	YPDnc2	YPDnc3	YPDnc4	EtOHnc1	EtOHnc2	Galnc1	Galnc2
11	ZTA1	YBR046C	YPDcl2	YPDcl3	EtOHcl1	EtOHcl2	Galcl1	-	YPDnc2	YPDnc3	YPDnc4	EtOHnc1	-	Galnc1	Galnc2
11	KGD1	YIL125W	YPDcl2	YPDcl3	-	EtOHcl2	Galcl1	YPDnc1	YPDnc2	-	YPDnc4	EtOHnc1	EtOHnc2	Galnc1	Galnc2
11	TOM22	YNL131W	YPDcl2	-	EtOHcl1	EtOHcl2	Galcl1	YPDnc1	YPDnc2	-	YPDnc4	EtOHnc1	EtOHnc2	Galnc1	Galnc2
11	GOS1	YHL031C	-	YPDcl3	-	EtOHcl2	Galcl1	YPDnc1	YPDnc2	YPDnc3	YPDnc4	EtOHnc1	EtOHnc2	Galnc1	Galnc2
11	HLR1	YDR528W	YPDcl2	YPDcl3	EtOHcl1	-	Galcl1	YPDnc1	YPDnc2	YPDnc3	YPDnc4	EtOHnc1	-	Galnc1	Galnc2
11	POL2	YNL262W	-	-	EtOHcl1	EtOHcl2	Galcl1	YPDnc1	YPDnc2	YPDnc3	YPDnc4	EtOHnc1	EtOHnc2	Galnc1	Galnc2
11	BRL1	YHR036W	YPDcl2	-	-	EtOHcl2	Galcl1	YPDnc1	YPDnc2	YPDnc3	YPDnc4	EtOHnc1	EtOHnc2	Galnc1	Galnc2
11	MRPL4	YLR439W	-	YPDcl3	EtOHcl1	-	Galcl1	YPDnc1	YPDnc2	YPDnc3	YPDnc4	EtOHnc1	EtOHnc2	Galnc1	Galnc2
11		YJL220W	YPDcl2	-	EtOHcl1	-	Galcl1	YPDnc1	YPDnc2	YPDnc3	YPDnc4	EtOHnc1	EtOHnc2	Galnc1	Galnc2
11		YEL020C-B	YPDcl2	YPDcl3	EtOHcl1	EtOHcl2	Galcl1	-	YPDnc2	YPDnc3	YPDnc4	EtOHnc1	EtOHnc2	-	Galnc2
11	RPS21A	YKR057W	-	YPDcl3	EtOHcl1	-	Galcl1	YPDnc1	YPDnc2	YPDnc3	YPDnc4	EtOHnc1	EtOHnc2	Galnc1	Galnc2
11		YDL023C	YPDcl2	-	-	EtOHcl2	Galcl1	YPDnc1	YPDnc2	YPDnc3	YPDnc4	EtOHnc1	EtOHnc2	Galnc1	Galnc2
11	APS3	YJL024C	YPDcl2	YPDcl3	EtOHcl1	-	Galcl1	YPDnc1	YPDnc2	YPDnc3	YPDnc4	EtOHnc1	-	Galnc1	Galnc2
11		YPR160W-/	YPDcl2	YPDcl3	-	-	Galcl1	YPDnc1	YPDnc2	YPDnc3	YPDnc4	EtOHnc1	EtOHnc2	Galnc1	Galnc2
11		YOL155W-/	-	YPDcl3	-	EtOHcl2	Galcl1	YPDnc1	YPDnc2	YPDnc3	YPDnc4	EtOHnc1	EtOHnc2	Galnc1	Galnc2
11	MUB1	YMR100W	YPDcl2	YPDcl3	-	EtOHcl2	-	YPDnc1	YPDnc2	YPDnc3	YPDnc4	EtOHnc1	EtOHnc2	Galnc1	Galnc2
11	TRP1	YDR007W	YPDcl2	YPDcl3	EtOHcl1	-	Galcl1	YPDnc1	YPDnc2	YPDnc3	YPDnc4	-	EtOHnc2	Galnc1	Galnc2
11	INP54	YOL065C	YPDcl2	YPDcl3	EtOHcl1	EtOHcl2	Galcl1	-	-	YPDnc3	YPDnc4	EtOHnc1	EtOHnc2	Galnc1	Galnc2
11	DNF2	YDR093W	YPDcl2	YPDcl3	EtOHcl1	EtOHcl2	Galcl1	YPDnc1	YPDnc2	YPDnc3	YPDnc4	EtOHnc1	-	Galnc1	-
11	ADH1	YOL086C	YPDcl2	YPDcl3	EtOHcl1	EtOHcl2	Galcl1	YPDnc1	YPDnc2	YPDnc3	YPDnc4	-	-	Galnc1	Galnc2
11		YBR032W	-	YPDcl3	EtOHcl1	-	Galcl1	YPDnc1	YPDnc2	YPDnc3	YPDnc4	EtOHnc1	EtOHnc2	Galnc1	Galnc2
11		YPL135C-A	YPDcl2	YPDcl3	EtOHcl1	-	-	YPDnc1	YPDnc2	YPDnc3	YPDnc4	EtOHnc1	EtOHnc2	Galnc1	Galnc2
11	MSN5	YDR335W	YPDcl2	-	EtOHcl1	EtOHcl2	Galcl1	YPDnc1	YPDnc2	YPDnc3	YPDnc4	-	EtOHnc2	Galnc1	Galnc2
11	BAG7	YOR134W	YPDcl2	YPDcl3	EtOHcl1	-	-	YPDnc1	YPDnc2	YPDnc3	YPDnc4	EtOHnc1	EtOHnc2	Galnc1	Galnc2
11		YGR022C	-	YPDcl3	EtOHcl1	EtOHcl2	Galcl1	YPDnc1	YPDnc2	YPDnc3	YPDnc4	EtOHnc1	EtOHnc2	-	Galnc2
11	BSC1	YDL037C	YPDcl2	-	EtOHcl1	-	Galcl1	YPDnc1	YPDnc2	YPDnc3	YPDnc4	EtOHnc1	EtOHnc2	Galnc1	Galnc2
11	FAP7	YDL166C	YPDcl2	-	EtOHcl1	EtOHcl2	Galcl1	YPDnc1	YPDnc2	YPDnc3	YPDnc4	EtOHnc1	-	Galnc1	Galnc2
11	STE11	YLR362W	-	YPDcl3	EtOHcl1	EtOHcl2	Galcl1	YPDnc1	YPDnc2	YPDnc3	YPDnc4	EtOHnc1	-	Galnc1	Galnc2
11	GUA1	YMR217W	-	-	EtOHcl1	EtOHcl2	Galcl1	YPDnc1	YPDnc2	YPDnc3	YPDnc4	EtOHnc1	EtOHnc2	Galnc1	Galnc2
11	ACA1	YER045C	-	YPDcl3	EtOHcl1	EtOHcl2	Galcl1	YPDnc1	YPDnc2	YPDnc3	YPDnc4	EtOHnc1	-	Galnc1	Galnc2
11	RPP0	YLR340W	-	-	EtOHcl1	EtOHcl2	Galcl1	YPDnc1	YPDnc2	YPDnc3	YPDnc4	EtOHnc1	EtOHnc2	Galnc1	Galnc2
11	PPQ1	YPL179W	YPDcl2	YPDcl3	EtOHcl1	EtOHcl2	-	YPDnc1	YPDnc2	-	YPDnc4	EtOHnc1	EtOHnc2	Galnc1	Galnc2

11		YER034W	YPDcl2	YPDcl3	EtOHcl1	EtOHcl2	Galcl1	YPDnc1	-	-	YPDnc4	EtOHnc1	EtOHnc2	Galnc1	Galnc2
11	RVS167	YDR388W	-	YPDcl3	EtOHcl1	-	Galcl1	YPDnc1	YPDnc2	YPDnc3	YPDnc4	EtOHnc1	EtOHnc2	Galnc1	Galnc2
11		YPR059C	YPDcl2	-	EtOHcl1	EtOHcl2	Galcl1	YPDnc1	YPDnc2	-	YPDnc4	EtOHnc1	EtOHnc2	Galnc1	Galnc2
11		YPL142C	-	-	EtOHcl1	EtOHcl2	Galcl1	YPDnc1	YPDnc2	YPDnc3	YPDnc4	EtOHnc1	EtOHnc2	Galnc1	Galnc2
11	RNR2	YJL026W	YPDcl2	YPDcl3	EtOHcl1	EtOHcl2	Galcl1	YPDnc1	YPDnc2	-	-	EtOHnc1	EtOHnc2	Galnc1	Galnc2
11		YGL074C	-	YPDcl3	EtOHcl1	-	Galcl1	YPDnc1	YPDnc2	YPDnc3	YPDnc4	EtOHnc1	EtOHnc2	Galnc1	Galnc2
11	SPO74	YGL170C	YPDcl2	-	EtOHcl1	-	Galcl1	YPDnc1	YPDnc2	YPDnc3	YPDnc4	EtOHnc1	EtOHnc2	Galnc1	Galnc2
11	SDT1	YGL224C	YPDcl2	YPDcl3	EtOHcl1	EtOHcl2	Galcl1	-	YPDnc2	YPDnc3	-	EtOHnc1	EtOHnc2	Galnc1	Galnc2
11		YPL182C	YPDcl2	-	EtOHcl1	EtOHcl2	Galcl1	YPDnc1	YPDnc2	YPDnc3	YPDnc4	EtOHnc1	EtOHnc2	-	Galnc2
11		YPL250W-A	YPDcl2	-	-	EtOHcl2	Galcl1	YPDnc1	YPDnc2	YPDnc3	YPDnc4	EtOHnc1	EtOHnc2	Galnc1	Galnc2
11	GEM1	YAL048C	YPDcl2	YPDcl3	EtOHcl1	EtOHcl2	-	YPDnc1	YPDnc2	YPDnc3	YPDnc4	EtOHnc1	EtOHnc2	Galnc1	-
11		YKR033C	YPDcl2	YPDcl3	-	-	Galcl1	YPDnc1	YPDnc2	YPDnc3	YPDnc4	EtOHnc1	EtOHnc2	Galnc1	Galnc2
11	DNF3	YMR162C	YPDcl2	YPDcl3	-	-	Galcl1	YPDnc1	YPDnc2	YPDnc3	YPDnc4	EtOHnc1	EtOHnc2	Galnc1	Galnc2
11	SSF2	YDR312W	-	YPDcl3	-	EtOHcl2	Galcl1	YPDnc1	YPDnc2	YPDnc3	YPDnc4	EtOHnc1	EtOHnc2	Galnc1	Galnc2
11		YGR054W	YPDcl2	-	EtOHcl1	-	Galcl1	YPDnc1	YPDnc2	YPDnc3	YPDnc4	EtOHnc1	EtOHnc2	Galnc1	Galnc2
11		YNR001W-A	YPDcl2	YPDcl3	EtOHcl1	-	Galcl1	YPDnc1	YPDnc2	YPDnc3	YPDnc4	EtOHnc1	-	Galnc1	Galnc2
11	ERG26	YGL001C	YPDcl2	YPDcl3	EtOHcl1	EtOHcl2	Galcl1	-	YPDnc2	-	YPDnc4	EtOHnc1	EtOHnc2	Galnc1	Galnc2
11		YOR378W	YPDcl2	YPDcl3	EtOHcl1	EtOHcl2	Galcl1	YPDnc1	YPDnc2	YPDnc3	-	EtOHnc1	-	Galnc1	Galnc2
11	VPS61	YDR136C	-	YPDcl3	EtOHcl1	EtOHcl2	Galcl1	YPDnc1	YPDnc2	YPDnc3	YPDnc4	EtOHnc1	-	Galnc1	Galnc2
11	KTI12	YKL110C	-	YPDcl3	EtOHcl1	-	Galcl1	YPDnc1	YPDnc2	YPDnc3	YPDnc4	EtOHnc1	EtOHnc2	Galnc1	Galnc2
11	HRT1	YOL133W	YPDcl2	-	EtOHcl1	-	Galcl1	YPDnc1	YPDnc2	YPDnc3	YPDnc4	EtOHnc1	EtOHnc2	Galnc1	Galnc2
11	CBP1	YJL209W	YPDcl2	-	EtOHcl1	-	Galcl1	YPDnc1	YPDnc2	YPDnc3	YPDnc4	EtOHnc1	EtOHnc2	Galnc1	Galnc2
11		YMR118C	-	YPDcl3	EtOHcl1	-	Galcl1	YPDnc1	YPDnc2	YPDnc3	YPDnc4	EtOHnc1	EtOHnc2	Galnc1	Galnc2
11	MTO1	YGL236C	-	-	EtOHcl1	EtOHcl2	Galcl1	YPDnc1	YPDnc2	YPDnc3	YPDnc4	EtOHnc1	EtOHnc2	Galnc1	Galnc2
11	RNH203	YLR154C	YPDcl2	YPDcl3	-	-	Galcl1	YPDnc1	YPDnc2	YPDnc3	YPDnc4	EtOHnc1	EtOHnc2	Galnc1	Galnc2
11		YGR269W	YPDcl2	YPDcl3	-	-	Galcl1	YPDnc1	YPDnc2	YPDnc3	YPDnc4	EtOHnc1	EtOHnc2	Galnc1	Galnc2
11	MTH1	YDR277C	YPDcl2	YPDcl3	EtOHcl1	-	Galcl1	YPDnc1	YPDnc2	-	YPDnc4	EtOHnc1	EtOHnc2	Galnc1	Galnc2
11	CCC1	YLR220W	YPDcl2	-	EtOHcl1	EtOHcl2	Galcl1	YPDnc1	YPDnc2	YPDnc3	-	EtOHnc1	EtOHnc2	Galnc1	Galnc2
11	FRS2	YFL022C	YPDcl2	YPDcl3	EtOHcl1	EtOHcl2	Galcl1	YPDnc1	-	YPDnc3	YPDnc4	EtOHnc1	-	Galnc1	Galnc2
11	RPS28A	YOR167C	YPDcl2	YPDcl3	EtOHcl1	EtOHcl2	Galcl1	-	YPDnc2	-	YPDnc4	EtOHnc1	EtOHnc2	Galnc1	Galnc2
11	PET9	YBL030C	YPDcl2	YPDcl3	EtOHcl1	-	Galcl1	YPDnc1	YPDnc2	YPDnc3	YPDnc4	-	EtOHnc2	Galnc1	Galnc2
11	ALG8	YOR067C	-	YPDcl3	-	EtOHcl2	Galcl1	YPDnc1	YPDnc2	YPDnc3	YPDnc4	EtOHnc1	EtOHnc2	Galnc1	Galnc2
11		YGL072C	YPDcl2	YPDcl3	EtOHcl1	-	-	YPDnc1	YPDnc2	YPDnc3	YPDnc4	EtOHnc1	EtOHnc2	Galnc1	Galnc2
11	PIG2	YIL045W	YPDcl2	YPDcl3	EtOHcl1	EtOHcl2	Galcl1	YPDnc1	-	YPDnc3	-	EtOHnc1	EtOHnc2	Galnc1	Galnc2

11	SAS5	YOR213C	YPDcl2	YPDcl3	EtOHcl1	-	Galcl1	YPDnc1	YPDnc2	YPDnc3	YPDnc4	-	EtOHnc2	Galnc1	Galnc2
11		YGR146C-A	-	YPDcl3	EtOHcl1	EtOHcl2	Galcl1	YPDnc1	YPDnc2	-	YPDnc4	EtOHnc1	EtOHnc2	Galnc1	Galnc2
11		YDR535C	-	YPDcl3	EtOHcl1	EtOHcl2	Galcl1	YPDnc1	YPDnc2	YPDnc3	YPDnc4	EtOHnc1	-	Galnc1	Galnc2
11	SCP1	YOR367W	YPDcl2	-	EtOHcl1	-	Galcl1	YPDnc1	YPDnc2	YPDnc3	YPDnc4	EtOHnc1	EtOHnc2	Galnc1	Galnc2
11	YUH1	YJR099W	YPDcl2	YPDcl3	EtOHcl1	-	Galcl1	YPDnc1	YPDnc2	YPDnc3	-	EtOHnc1	EtOHnc2	Galnc1	Galnc2
11	KAP122	YGL016W	YPDcl2	YPDcl3	EtOHcl1	EtOHcl2	Galcl1	-	YPDnc2	YPDnc3	YPDnc4	-	EtOHnc2	Galnc1	Galnc2
11		YGR045C	YPDcl2	YPDcl3	EtOHcl1	EtOHcl2	Galcl1	YPDnc1	-	YPDnc3	YPDnc4	EtOHnc1	-	Galnc1	Galnc2
11	GUK1	YDR454C	-	-	EtOHcl1	EtOHcl2	Galcl1	YPDnc1	YPDnc2	YPDnc3	YPDnc4	EtOHnc1	EtOHnc2	Galnc1	Galnc2
11		YLR346C	YPDcl2	-	EtOHcl1	-	Galcl1	YPDnc1	YPDnc2	YPDnc3	YPDnc4	EtOHnc1	EtOHnc2	Galnc1	Galnc2
11	SWT1	YOR166C	YPDcl2	-	EtOHcl1	EtOHcl2	Galcl1	YPDnc1	YPDnc2	YPDnc3	YPDnc4	EtOHnc1	-	Galnc1	Galnc2
11	RXT3	YDL076C	YPDcl2	YPDcl3	EtOHcl1	-	Galcl1	-	YPDnc2	YPDnc3	YPDnc4	EtOHnc1	EtOHnc2	Galnc1	Galnc2
11	ATX2	YOR079C	-	-	EtOHcl1	EtOHcl2	Galcl1	YPDnc1	YPDnc2	YPDnc3	YPDnc4	EtOHnc1	EtOHnc2	Galnc1	Galnc2
11		YNL155W	YPDcl2	-	EtOHcl1	-	Galcl1	YPDnc1	YPDnc2	YPDnc3	YPDnc4	EtOHnc1	EtOHnc2	Galnc1	Galnc2
11		YBR285W	YPDcl2	YPDcl3	EtOHcl1	-	Galcl1	YPDnc1	YPDnc2	YPDnc3	YPDnc4	EtOHnc1	-	Galnc1	Galnc2
11	SMD2	YLR275W	YPDcl2	-	EtOHcl1	-	Galcl1	YPDnc1	YPDnc2	YPDnc3	YPDnc4	EtOHnc1	EtOHnc2	Galnc1	Galnc2
11	AIM25	YJR100C	YPDcl2	YPDcl3	EtOHcl1	EtOHcl2	-	YPDnc1	YPDnc2	YPDnc3	-	EtOHnc1	EtOHnc2	Galnc1	Galnc2
11	RRT5	YFR032C	YPDcl2	YPDcl3	EtOHcl1	EtOHcl2	Galcl1	YPDnc1	-	YPDnc3	-	EtOHnc1	EtOHnc2	Galnc1	Galnc2
11	SPN1	YPR133C	YPDcl2	YPDcl3	EtOHcl1	EtOHcl2	Galcl1	YPDnc1	-	YPDnc3	-	EtOHnc1	EtOHnc2	Galnc1	Galnc2
11	PRP39	YML046W	YPDcl2	YPDcl3	EtOHcl1	EtOHcl2	Galcl1	YPDnc1	YPDnc2	YPDnc3	YPDnc4	EtOHnc1	-	-	Galnc2
11	RRN3	YKL125W	-	YPDcl3	EtOHcl1	EtOHcl2	Galcl1	YPDnc1	YPDnc2	-	YPDnc4	EtOHnc1	EtOHnc2	Galnc1	Galnc2
11	PEX8	YGR077C	-	YPDcl3	EtOHcl1	EtOHcl2	Galcl1	-	YPDnc2	YPDnc3	YPDnc4	EtOHnc1	EtOHnc2	Galnc1	Galnc2
11	UBC1	YDR177W	YPDcl2	YPDcl3	EtOHcl1	-	Galcl1	YPDnc1	YPDnc2	YPDnc3	YPDnc4	-	EtOHnc2	Galnc1	Galnc2
11	MON1	YGL124C	YPDcl2	-	EtOHcl1	EtOHcl2	Galcl1	YPDnc1	-	YPDnc3	YPDnc4	EtOHnc1	EtOHnc2	Galnc1	Galnc2
11		YDR417C	YPDcl2	-	EtOHcl1	EtOHcl2	Galcl1	YPDnc1	YPDnc2	YPDnc3	YPDnc4	EtOHnc1	EtOHnc2	Galnc1	-
11	ISF1	YMR081C	YPDcl2	-	EtOHcl1	-	Galcl1	YPDnc1	YPDnc2	YPDnc3	YPDnc4	EtOHnc1	EtOHnc2	Galnc1	Galnc2
11	INO80	YGL150C	YPDcl2	-	EtOHcl1	-	Galcl1	YPDnc1	YPDnc2	YPDnc3	YPDnc4	EtOHnc1	EtOHnc2	Galnc1	Galnc2
11	MEH1	YKR007W	-	YPDcl3	EtOHcl1	-	Galcl1	YPDnc1	YPDnc2	YPDnc3	YPDnc4	EtOHnc1	EtOHnc2	Galnc1	Galnc2
11		YKL131W	-	-	EtOHcl1	EtOHcl2	Galcl1	YPDnc1	YPDnc2	YPDnc3	YPDnc4	EtOHnc1	EtOHnc2	Galnc1	Galnc2
11	SPO19	YPL130W	YPDcl2	-	EtOHcl1	-	Galcl1	YPDnc1	YPDnc2	YPDnc3	YPDnc4	EtOHnc1	EtOHnc2	Galnc1	Galnc2
11	FIT1	YDR534C	YPDcl2	YPDcl3	EtOHcl1	EtOHcl2	Galcl1	YPDnc1	YPDnc2	YPDnc3	YPDnc4	EtOHnc1	-	Galnc1	-
11	RCN2	YOR220W	-	YPDcl3	EtOHcl1	EtOHcl2	Galcl1	YPDnc1	YPDnc2	YPDnc3	YPDnc4	EtOHnc1	-	Galnc1	Galnc2
11	RIO1	YOR119C	-	YPDcl3	EtOHcl1	EtOHcl2	-	YPDnc1	YPDnc2	YPDnc3	YPDnc4	EtOHnc1	EtOHnc2	Galnc1	Galnc2
11		YPR177C	-	YPDcl3	EtOHcl1	-	Galcl1	YPDnc1	YPDnc2	YPDnc3	YPDnc4	EtOHnc1	EtOHnc2	Galnc1	Galnc2
11		YJR120W	YPDcl2	-	EtOHcl1	-	Galcl1	YPDnc1	YPDnc2	YPDnc3	YPDnc4	EtOHnc1	EtOHnc2	Galnc1	Galnc2

11	AGA2	YGL032C	-	-	EtOHcl1	EtOHcl2	Galcl1	YPDnc1	YPDnc2	YPDnc3	YPDnc4	EtOHnc1	EtOHnc2	Galnc1	Galnc2
11	SAM3	YPL274W	YPDcl2	YPDcl3	EtOHcl1	EtOHcl2	Galcl1	YPDnc1	YPDnc2	YPDnc3	-	EtOHnc1	EtOHnc2	-	Galnc2
11	COS8	YHL048W	-	-	EtOHcl1	EtOHcl2	Galcl1	YPDnc1	YPDnc2	YPDnc3	YPDnc4	EtOHnc1	EtOHnc2	Galnc1	Galnc2
11		YDL007C-A	YPDcl2	YPDcl3	EtOHcl1	-	Galcl1	YPDnc1	YPDnc2	YPDnc3	-	EtOHnc1	EtOHnc2	Galnc1	Galnc2
11		YGR228W	-	YPDcl3	-	EtOHcl2	Galcl1	YPDnc1	YPDnc2	YPDnc3	YPDnc4	EtOHnc1	EtOHnc2	Galnc1	Galnc2
11		YDR250C	-	YPDcl3	EtOHcl1	EtOHcl2	Galcl1	YPDnc1	-	YPDnc3	YPDnc4	EtOHnc1	EtOHnc2	Galnc1	Galnc2
11	MIG3	YER028C	YPDcl2	-	EtOHcl1	EtOHcl2	Galcl1	-	YPDnc2	YPDnc3	YPDnc4	EtOHnc1	EtOHnc2	Galnc1	Galnc2
11	CSM4	YPL200W	YPDcl2	YPDcl3	EtOHcl1	EtOHcl2	Galcl1	YPDnc1	-	-	YPDnc4	EtOHnc1	EtOHnc2	Galnc1	Galnc2
11	TEF4	YKL081W	YPDcl2	-	EtOHcl1	EtOHcl2	Galcl1	YPDnc1	-	YPDnc3	YPDnc4	EtOHnc1	EtOHnc2	Galnc1	Galnc2
11	SRL2	YLR082C	-	YPDcl3	EtOHcl1	EtOHcl2	Galcl1	YPDnc1	YPDnc2	YPDnc3	-	EtOHnc1	EtOHnc2	Galnc1	Galnc2
11	RPL24A	YGL031C	-	-	EtOHcl1	EtOHcl2	Galcl1	YPDnc1	YPDnc2	YPDnc3	YPDnc4	EtOHnc1	EtOHnc2	Galnc1	Galnc2
11		YMR272W-	YPDcl2	YPDcl3	EtOHcl1	-	Galcl1	YPDnc1	YPDnc2	YPDnc3	YPDnc4	EtOHnc1	-	Galnc1	Galnc2
11	GPA2	YER020W	YPDcl2	YPDcl3	EtOHcl1	EtOHcl2	Galcl1	YPDnc1	YPDnc2	-	YPDnc4	EtOHnc1	-	Galnc1	Galnc2
11	NRG2	YBR066C	YPDcl2	-	EtOHcl1	-	Galcl1	YPDnc1	YPDnc2	YPDnc3	YPDnc4	EtOHnc1	EtOHnc2	Galnc1	Galnc2
11		YNR003W-	YPDcl2	YPDcl3	EtOHcl1	-	Galcl1	YPDnc1	YPDnc2	YPDnc3	YPDnc4	EtOHnc1	-	Galnc1	Galnc2
11	YTH1	YPR107C	-	YPDcl3	EtOHcl1	EtOHcl2	Galcl1	YPDnc1	YPDnc2	-	YPDnc4	EtOHnc1	EtOHnc2	Galnc1	Galnc2
11	POP4	YBR257W	YPDcl2	YPDcl3	EtOHcl1	EtOHcl2	Galcl1	-	YPDnc2	YPDnc3	YPDnc4	EtOHnc1	EtOHnc2	Galnc1	-
11		YDL199C	-	YPDcl3	EtOHcl1	EtOHcl2	Galcl1	YPDnc1	YPDnc2	-	YPDnc4	EtOHnc1	EtOHnc2	Galnc1	Galnc2
10	DLS1	YJL065C	YPDcl2	-	EtOHcl1	-	Galcl1	YPDnc1	YPDnc2	YPDnc3	YPDnc4	EtOHnc1	EtOHnc2	-	Galnc2
10	PXA1	YPL147W	YPDcl2	YPDcl3	EtOHcl1	EtOHcl2	Galcl1	YPDnc1	YPDnc2	YPDnc3	YPDnc4	-	EtOHnc2	-	-
10		YLR299C-A	-	YPDcl3	EtOHcl1	-	Galcl1	YPDnc1	YPDnc2	YPDnc3	YPDnc4	EtOHnc1	-	Galnc1	Galnc2
10	PRO2	YOR323C	-	YPDcl3	EtOHcl1	EtOHcl2	Galcl1	-	YPDnc2	YPDnc3	YPDnc4	EtOHnc1	-	Galnc1	Galnc2
10	TIM50	YPL063W	YPDcl2	YPDcl3	EtOHcl1	EtOHcl2	Galcl1	YPDnc1	YPDnc2	-	-	EtOHnc1	-	Galnc1	Galnc2
10		YGR115C	YPDcl2	YPDcl3	-	EtOHcl2	Galcl1	YPDnc1	YPDnc2	-	YPDnc4	EtOHnc1	-	Galnc1	Galnc2
10	CET1	YPL228W	YPDcl2	YPDcl3	EtOHcl1	EtOHcl2	Galcl1	-	YPDnc2	YPDnc3	YPDnc4	-	-	Galnc1	Galnc2
10	IPT1	YDR072C	YPDcl2	YPDcl3	EtOHcl1	-	Galcl1	YPDnc1	YPDnc2	-	YPDnc4	EtOHnc1	-	Galnc1	Galnc2
10	RPL9A	YGL147C	YPDcl2	YPDcl3	-	-	-	YPDnc1	YPDnc2	YPDnc3	YPDnc4	EtOHnc1	EtOHnc2	Galnc1	Galnc2
10		YDR049W	-	-	EtOHcl1	EtOHcl2	Galcl1	YPDnc1	YPDnc2	YPDnc3	YPDnc4	EtOHnc1	-	Galnc1	Galnc2
10	BSP1	YPR171W	-	YPDcl3	EtOHcl1	-	-	YPDnc1	YPDnc2	YPDnc3	YPDnc4	EtOHnc1	EtOHnc2	Galnc1	Galnc2
10	DAP2	YHR028C	YPDcl2	YPDcl3	EtOHcl1	EtOHcl2	Galcl1	-	YPDnc2	YPDnc3	YPDnc4	-	EtOHnc2	Galnc1	-
10		YPL107W	YPDcl2	YPDcl3	EtOHcl1	EtOHcl2	Galcl1	YPDnc1	-	-	YPDnc4	EtOHnc1	EtOHnc2	-	Galnc2
10	STP4	YDL048C	YPDcl2	YPDcl3	EtOHcl1	EtOHcl2	-	YPDnc1	-	YPDnc3	-	EtOHnc1	EtOHnc2	Galnc1	Galnc2
10	HXT4	YHR092C	-	-	EtOHcl1	-	Galcl1	YPDnc1	YPDnc2	YPDnc3	YPDnc4	EtOHnc1	EtOHnc2	Galnc1	Galnc2
10	NAT3	YPR131C	YPDcl2	YPDcl3	EtOHcl1	EtOHcl2	Galcl1	YPDnc1	YPDnc2	-	YPDnc4	EtOHnc1	EtOHnc2	-	-

10	GLO2	YDR272W	-	YPDcl3	EtOHcl1	-	Galcl1	YPDnc1	YPDnc2	YPDnc3	-	EtOHnc1	EtOHnc2	Galnc1	Galnc2
10		YOR238W	-	-	-	EtOHcl2	Galcl1	YPDnc1	YPDnc2	YPDnc3	YPDnc4	EtOHnc1	EtOHnc2	Galnc1	Galnc2
10		YGR210C	YPDcl2	YPDcl3	EtOHcl1	EtOHcl2	Galcl1	YPDnc1	-	-	-	EtOHnc1	EtOHnc2	Galnc1	Galnc2
10		YKL107W	YPDcl2	-	EtOHcl1	-	Galcl1	YPDnc1	YPDnc2	YPDnc3	YPDnc4	EtOHnc1	EtOHnc2	-	Galnc2
10	FDH1	YOR388C	YPDcl2	YPDcl3	-	-	-	YPDnc1	YPDnc2	YPDnc3	YPDnc4	EtOHnc1	EtOHnc2	Galnc1	Galnc2
10	NOC3	YLR002C	YPDcl2	YPDcl3	EtOHcl1	EtOHcl2	Galcl1	YPDnc1	YPDnc2	YPDnc3	-	-	EtOHnc2	-	Galnc2
10	ARK1	YNL020C	YPDcl2	YPDcl3	EtOHcl1	EtOHcl2	Galcl1	-	-	-	YPDnc4	EtOHnc1	EtOHnc2	Galnc1	Galnc2
10	FKH2	YNL068C	YPDcl2	YPDcl3	-	EtOHcl2	Galcl1	YPDnc1	YPDnc2	-	-	EtOHnc1	EtOHnc2	Galnc1	Galnc2
10		YJL049W	-	YPDcl3	EtOHcl1	-	Galcl1	YPDnc1	YPDnc2	YPDnc3	YPDnc4	EtOHnc1	-	Galnc1	Galnc2
10	BRN1	YBL097W	YPDcl2	-	EtOHcl1	-	Galcl1	YPDnc1	YPDnc2	YPDnc3	YPDnc4	EtOHnc1	EtOHnc2	-	Galnc2
10		YAR070C	YPDcl2	-	EtOHcl1	EtOHcl2	Galcl1	YPDnc1	YPDnc2	YPDnc3	YPDnc4	-	EtOHnc2	Galnc1	-
10	SRS2	YJL092W	YPDcl2	YPDcl3	EtOHcl1	EtOHcl2	Galcl1	-	YPDnc2	YPDnc3	YPDnc4	-	EtOHnc2	Galnc1	-
10	INP52	YNL106C	-	YPDcl3	EtOHcl1	EtOHcl2	Galcl1	YPDnc1	YPDnc2	YPDnc3	YPDnc4	-	EtOHnc2	Galnc1	-
10	SNX41	YDR425W	-	-	EtOHcl1	EtOHcl2	Galcl1	YPDnc1	YPDnc2	YPDnc3	YPDnc4	EtOHnc1	-	Galnc1	Galnc2
10	DIP2	YLR129W	YPDcl2	-	EtOHcl1	-	Galcl1	YPDnc1	YPDnc2	YPDnc3	-	EtOHnc1	EtOHnc2	Galnc1	Galnc2
10	SGF73	YGL066W	YPDcl2	YPDcl3	EtOHcl1	EtOHcl2	Galcl1	YPDnc1	YPDnc2	-	YPDnc4	EtOHnc1	-	Galnc1	-
10	PRY3	YJL078C	-	-	EtOHcl1	EtOHcl2	-	YPDnc1	YPDnc2	YPDnc3	YPDnc4	EtOHnc1	EtOHnc2	Galnc1	Galnc2
10	GPR1	YDL035C	-	-	EtOHcl1	EtOHcl2	Galcl1	YPDnc1	YPDnc2	YPDnc3	YPDnc4	EtOHnc1	EtOHnc2	Galnc1	-
10		YML012C-A	YPDcl2	-	EtOHcl1	EtOHcl2	Galcl1	YPDnc1	YPDnc2	YPDnc3	YPDnc4	-	EtOHnc2	Galnc1	-
10		YCR018C-A	YPDcl2	YPDcl3	-	-	Galcl1	YPDnc1	YPDnc2	YPDnc3	YPDnc4	EtOHnc1	-	Galnc1	Galnc2
10	IOC3	YFR013W	-	YPDcl3	-	-	Galcl1	YPDnc1	YPDnc2	YPDnc3	YPDnc4	EtOHnc1	EtOHnc2	Galnc1	Galnc2
10	YPK2	YMR104C	YPDcl2	YPDcl3	EtOHcl1	-	Galcl1	YPDnc1	-	-	YPDnc4	EtOHnc1	EtOHnc2	Galnc1	Galnc2
10	CTK1	YKL139W	YPDcl2	YPDcl3	EtOHcl1	EtOHcl2	Galcl1	-	YPDnc2	YPDnc3	YPDnc4	-	EtOHnc2	Galnc1	-
10	VPS17	YOR132W	-	YPDcl3	EtOHcl1	-	Galcl1	YPDnc1	YPDnc2	YPDnc3	YPDnc4	EtOHnc1	EtOHnc2	Galnc1	-
10	RMA1	YKL132C	YPDcl2	YPDcl3	EtOHcl1	EtOHcl2	Galcl1	-	-	-	YPDnc4	EtOHnc1	EtOHnc2	Galnc1	Galnc2
10		YMR158C-A	YPDcl2	-	EtOHcl1	-	Galcl1	YPDnc1	YPDnc2	YPDnc3	YPDnc4	EtOHnc1	-	Galnc1	Galnc2
10	SRB7	YDR308C	-	YPDcl3	EtOHcl1	EtOHcl2	Galcl1	YPDnc1	-	-	YPDnc4	EtOHnc1	EtOHnc2	Galnc1	Galnc2
10		YDR215C	-	YPDcl3	-	-	Galcl1	YPDnc1	YPDnc2	YPDnc3	YPDnc4	EtOHnc1	EtOHnc2	Galnc1	Galnc2
10	TMA7	YLR262C-A	-	-	EtOHcl1	EtOHcl2	Galcl1	YPDnc1	YPDnc2	YPDnc3	-	EtOHnc1	EtOHnc2	Galnc1	Galnc2
10	UBX2	YML013W	YPDcl2	-	EtOHcl1	EtOHcl2	Galcl1	YPDnc1	YPDnc2	YPDnc3	YPDnc4	-	EtOHnc2	Galnc1	-
10	CCH1	YGR217W	YPDcl2	YPDcl3	EtOHcl1	-	Galcl1	YPDnc1	YPDnc2	-	-	EtOHnc1	EtOHnc2	Galnc1	Galnc2
10		YEL014C	-	-	EtOHcl1	EtOHcl2	Galcl1	YPDnc1	YPDnc2	YPDnc3	YPDnc4	EtOHnc1	-	Galnc1	Galnc2
10	PCL2	YDL127W	YPDcl2	YPDcl3	EtOHcl1	EtOHcl2	Galcl1	YPDnc1	-	YPDnc3	-	EtOHnc1	-	Galnc1	Galnc2
10		YHR139C-A	YPDcl2	YPDcl3	EtOHcl1	-	Galcl1	YPDnc1	-	-	YPDnc4	EtOHnc1	EtOHnc2	Galnc1	Galnc2

10	GAS5	YOL030W	-	YPDcl3	EtOHcl1	EtOHcl2	-	YPDnc1	-	YPDnc3	YPDnc4	EtOHnc1	EtOHnc2	Galnc1	Galnc2
10	VPS66	YPR139C	YPDcl2	-	-	EtOHcl2	Galcl1	YPDnc1	YPDnc2	YPDnc3	YPDnc4	EtOHnc1	-	Galnc1	Galnc2
10		YDL221W	-	YPDcl3	EtOHcl1	EtOHcl2	Galcl1	YPDnc1	YPDnc2	YPDnc3	-	EtOHnc1	-	Galnc1	Galnc2
10	TBS1	YBR150C	-	YPDcl3	EtOHcl1	EtOHcl2	Galcl1	YPDnc1	YPDnc2	-	-	EtOHnc1	EtOHnc2	Galnc1	Galnc2
10	HAL9	YOL089C	YPDcl2	YPDcl3	EtOHcl1	-	Galcl1	-	YPDnc2	YPDnc3	YPDnc4	EtOHnc1	EtOHnc2	-	Galnc2
10	CRS5	YOR031W	YPDcl2	YPDcl3	EtOHcl1	-	-	YPDnc1	YPDnc2	YPDnc3	YPDnc4	-	EtOHnc2	Galnc1	Galnc2
10		YLR118C	YPDcl2	-	EtOHcl1	-	Galcl1	YPDnc1	YPDnc2	YPDnc3	YPDnc4	EtOHnc1	-	Galnc1	Galnc2
10	ALR1	YOL130W	YPDcl2	-	EtOHcl1	-	-	YPDnc1	YPDnc2	YPDnc3	YPDnc4	EtOHnc1	EtOHnc2	Galnc1	Galnc2
10	SPG4	YMR107W	-	-	EtOHcl1	-	Galcl1	YPDnc1	YPDnc2	YPDnc3	YPDnc4	EtOHnc1	EtOHnc2	Galnc1	Galnc2
10	GAL11	YOL051W	YPDcl2	YPDcl3	EtOHcl1	EtOHcl2	Galcl1	-	YPDnc2	YPDnc3	YPDnc4	-	EtOHnc2	-	Galnc2
10		YBL096C	-	YPDcl3	EtOHcl1	-	Galcl1	YPDnc1	YPDnc2	YPDnc3	YPDnc4	EtOHnc1	EtOHnc2	Galnc1	-
10	DMR1	YGR150C	YPDcl2	YPDcl3	EtOHcl1	EtOHcl2	Galcl1	-	YPDnc2	YPDnc3	YPDnc4	-	EtOHnc2	-	Galnc2
10	SCD5	YOR329C	YPDcl2	YPDcl3	EtOHcl1	EtOHcl2	Galcl1	-	YPDnc2	YPDnc3	YPDnc4	-	EtOHnc2	-	Galnc2
10	SMC2	YFR031C	-	-	EtOHcl1	EtOHcl2	-	YPDnc1	YPDnc2	YPDnc3	YPDnc4	EtOHnc1	EtOHnc2	Galnc1	Galnc2
10	MSR1	YHR091C	-	YPDcl3	EtOHcl1	EtOHcl2	Galcl1	YPDnc1	YPDnc2	YPDnc3	-	EtOHnc1	EtOHnc2	-	Galnc2
10	CUE4	YML101C	-	YPDcl3	EtOHcl1	EtOHcl2	Galcl1	YPDnc1	YPDnc2	YPDnc3	YPDnc4	-	-	Galnc1	Galnc2
10	PGD1	YGL025C	-	YPDcl3	-	EtOHcl2	Galcl1	-	YPDnc2	YPDnc3	YPDnc4	EtOHnc1	EtOHnc2	Galnc1	Galnc2
10	FET3	YMR058W	-	YPDcl3	EtOHcl1	EtOHcl2	-	YPDnc1	YPDnc2	YPDnc3	-	EtOHnc1	EtOHnc2	Galnc1	Galnc2
10	RBG2	YGR173W	YPDcl2	YPDcl3	EtOHcl1	-	Galcl1	-	YPDnc2	YPDnc3	YPDnc4	EtOHnc1	EtOHnc2	-	Galnc2
10	ATO2	YNR002C	YPDcl2	YPDcl3	EtOHcl1	-	Galcl1	YPDnc1	YPDnc2	YPDnc3	YPDnc4	EtOHnc1	-	-	Galnc2
10		YPR150W	YPDcl2	YPDcl3	-	-	Galcl1	YPDnc1	YPDnc2	YPDnc3	YPDnc4	EtOHnc1	-	Galnc1	Galnc2
10	TFB1	YDR311W	-	YPDcl3	EtOHcl1	-	Galcl1	YPDnc1	YPDnc2	YPDnc3	YPDnc4	-	EtOHnc2	Galnc1	Galnc2
10	ECM22	YLR228C	YPDcl2	-	-	-	Galcl1	YPDnc1	YPDnc2	YPDnc3	YPDnc4	EtOHnc1	EtOHnc2	Galnc1	Galnc2
10	TMA19	YKL056C	YPDcl2	-	EtOHcl1	EtOHcl2	-	YPDnc1	-	YPDnc3	YPDnc4	EtOHnc1	EtOHnc2	Galnc1	Galnc2
10	EMP24	YGL200C	YPDcl2	-	EtOHcl1	EtOHcl2	Galcl1	YPDnc1	-	-	YPDnc4	EtOHnc1	EtOHnc2	Galnc1	Galnc2
10	AEP3	YPL005W	YPDcl2	YPDcl3	EtOHcl1	-	Galcl1	YPDnc1	YPDnc2	YPDnc3	YPDnc4	-	-	Galnc1	Galnc2
10		YCR064C	-	YPDcl3	EtOHcl1	-	-	YPDnc1	YPDnc2	YPDnc3	YPDnc4	EtOHnc1	EtOHnc2	Galnc1	Galnc2
10	HPT1	YDR399W	-	-	EtOHcl1	-	Galcl1	YPDnc1	YPDnc2	YPDnc3	YPDnc4	EtOHnc1	EtOHnc2	Galnc1	Galnc2
10	YLH47	YPR125W	YPDcl2	YPDcl3	EtOHcl1	-	Galcl1	YPDnc1	-	YPDnc3	YPDnc4	EtOHnc1	-	Galnc1	Galnc2
10	IML2	YJL082W	-	-	-	EtOHcl2	Galcl1	YPDnc1	YPDnc2	YPDnc3	YPDnc4	EtOHnc1	EtOHnc2	Galnc1	Galnc2
10	RRP46	YGR095C	YPDcl2	-	-	EtOHcl2	Galcl1	YPDnc1	-	YPDnc3	YPDnc4	EtOHnc1	EtOHnc2	Galnc1	Galnc2
10	SPS2	YDR522C	YPDcl2	-	-	-	Galcl1	YPDnc1	YPDnc2	YPDnc3	YPDnc4	EtOHnc1	EtOHnc2	Galnc1	Galnc2
10	MAM1	YER106W	YPDcl2	-	EtOHcl1	-	-	YPDnc1	YPDnc2	YPDnc3	YPDnc4	EtOHnc1	EtOHnc2	Galnc1	Galnc2
10	ERP6	YGL002W	-	-	EtOHcl1	EtOHcl2	Galcl1	YPDnc1	YPDnc2	YPDnc3	YPDnc4	-	EtOHnc2	Galnc1	Galnc2

10	MRPL3	YMR024W	YPDcl2	-	EtOHcl1	EtOHcl2	Galcl1	YPDnc1	YPDnc2	YPDnc3	-	EtOHnc1	-	Galnc1	Galnc2
10		YOR316C-A	-	-	EtOHcl1	-	Galcl1	YPDnc1	YPDnc2	YPDnc3	YPDnc4	EtOHnc1	EtOHnc2	Galnc1	Galnc2
10	REC114	YMR133W	YPDcl2	YPDcl3	EtOHcl1	-	Galcl1	YPDnc1	YPDnc2	YPDnc3	-	EtOHnc1	EtOHnc2	Galnc1	-
10	LEU1	YGL009C	YPDcl2	YPDcl3	EtOHcl1	EtOHcl2	Galcl1	-	-	YPDnc3	YPDnc4	-	EtOHnc2	Galnc1	Galnc2
10	DIA3	YDL024C	-	YPDcl3	EtOHcl1	EtOHcl2	Galcl1	-	YPDnc2	YPDnc3	-	EtOHnc1	EtOHnc2	Galnc1	Galnc2
10	VIP1	YLR410W	-	YPDcl3	EtOHcl1	-	-	YPDnc1	YPDnc2	YPDnc3	YPDnc4	EtOHnc1	EtOHnc2	Galnc1	Galnc2
10		YPL261C	-	YPDcl3	-	EtOHcl2	Galcl1	YPDnc1	YPDnc2	-	YPDnc4	EtOHnc1	EtOHnc2	Galnc1	Galnc2
10	HIS1	YER055C	YPDcl2	-	EtOHcl1	EtOHcl2	Galcl1	YPDnc1	-	-	YPDnc4	EtOHnc1	EtOHnc2	Galnc1	Galnc2
10	RRP3	YHR065C	YPDcl2	-	EtOHcl1	-	Galcl1	YPDnc1	YPDnc2	YPDnc3	YPDnc4	EtOHnc1	EtOHnc2	Galnc1	-
10		YDR426C	-	-	EtOHcl1	EtOHcl2	Galcl1	YPDnc1	YPDnc2	YPDnc3	YPDnc4	EtOHnc1	-	Galnc1	Galnc2
10	MPP10	YJR002W	YPDcl2	YPDcl3	-	-	Galcl1	YPDnc1	YPDnc2	YPDnc3	YPDnc4	EtOHnc1	-	Galnc1	Galnc2
10	YJU3	YKL094W	-	YPDcl3	EtOHcl1	-	Galcl1	YPDnc1	YPDnc2	YPDnc3	-	EtOHnc1	EtOHnc2	Galnc1	Galnc2
10	PAU20	YOL161C	-	-	EtOHcl1	-	Galcl1	YPDnc1	YPDnc2	YPDnc3	YPDnc4	EtOHnc1	EtOHnc2	Galnc1	Galnc2
10		YLR152C	-	-	EtOHcl1	EtOHcl2	-	YPDnc1	YPDnc2	YPDnc3	YPDnc4	EtOHnc1	EtOHnc2	Galnc1	Galnc2
10	RPN5	YDL147W	-	YPDcl3	EtOHcl1	-	Galcl1	YPDnc1	YPDnc2	YPDnc3	YPDnc4	-	EtOHnc2	Galnc1	Galnc2
10	HOF1	YMR032W	YPDcl2	YPDcl3	EtOHcl1	EtOHcl2	Galcl1	YPDnc1	YPDnc2	YPDnc3	YPDnc4	-	EtOHnc2	-	-
10	BSC4	YNL269W	YPDcl2	-	EtOHcl1	-	Galcl1	YPDnc1	-	YPDnc3	YPDnc4	EtOHnc1	EtOHnc2	Galnc1	Galnc2
10	SFG1	YOR315W	-	-	EtOHcl1	EtOHcl2	-	YPDnc1	YPDnc2	YPDnc3	YPDnc4	EtOHnc1	EtOHnc2	Galnc1	Galnc2
10	AXL1	YPR122W	YPDcl2	YPDcl3	EtOHcl1	-	Galcl1	YPDnc1	YPDnc2	YPDnc3	YPDnc4	-	-	Galnc1	Galnc2
10	SAT4	YCR008W	YPDcl2	YPDcl3	EtOHcl1	EtOHcl2	Galcl1	YPDnc1	-	-	YPDnc4	EtOHnc1	-	Galnc1	Galnc2
10		YHR020W	YPDcl2	YPDcl3	EtOHcl1	EtOHcl2	-	-	YPDnc2	YPDnc3	YPDnc4	EtOHnc1	-	Galnc1	Galnc2
10	RPL27B	YDR471W	YPDcl2	-	EtOHcl1	EtOHcl2	Galcl1	YPDnc1	-	-	YPDnc4	EtOHnc1	EtOHnc2	Galnc1	Galnc2
10	MRPS8	YMR158W	YPDcl2	-	EtOHcl1	-	Galcl1	YPDnc1	YPDnc2	YPDnc3	YPDnc4	EtOHnc1	-	Galnc1	Galnc2
10	GGA1	YDR358W	-	-	EtOHcl1	EtOHcl2	-	YPDnc1	YPDnc2	YPDnc3	YPDnc4	EtOHnc1	EtOHnc2	Galnc1	Galnc2
10	YIM2	YMR151W	YPDcl2	YPDcl3	EtOHcl1	-	Galcl1	-	YPDnc2	YPDnc3	YPDnc4	-	EtOHnc2	Galnc1	Galnc2
10		YHR032C-A	YPDcl2	-	EtOHcl1	EtOHcl2	-	YPDnc1	YPDnc2	YPDnc3	YPDnc4	EtOHnc1	-	Galnc1	Galnc2
10	NAS6	YGR232W	-	-	EtOHcl1	EtOHcl2	-	YPDnc1	YPDnc2	YPDnc3	YPDnc4	EtOHnc1	EtOHnc2	Galnc1	Galnc2
10	AIM39	YOL053W	YPDcl2	YPDcl3	EtOHcl1	-	Galcl1	YPDnc1	YPDnc2	YPDnc3	YPDnc4	-	-	Galnc1	Galnc2
10	UTP14	YML093W	YPDcl2	YPDcl3	EtOHcl1	EtOHcl2	Galcl1	YPDnc1	-	YPDnc3	-	EtOHnc1	EtOHnc2	Galnc1	-
10	SEC22	YLR268W	YPDcl2	YPDcl3	EtOHcl1	-	-	YPDnc1	YPDnc2	YPDnc3	YPDnc4	EtOHnc1	EtOHnc2	Galnc1	-
10	POL5	YEL055C	-	-	EtOHcl1	-	Galcl1	YPDnc1	YPDnc2	YPDnc3	YPDnc4	EtOHnc1	EtOHnc2	Galnc1	Galnc2
10	ALD3	YMR169C	YPDcl2	YPDcl3	-	EtOHcl2	Galcl1	YPDnc1	YPDnc2	YPDnc3	YPDnc4	-	-	Galnc1	Galnc2
10		YPL251W	-	YPDcl3	EtOHcl1	-	Galcl1	YPDnc1	YPDnc2	-	YPDnc4	EtOHnc1	EtOHnc2	Galnc1	Galnc2
10		YDL124W	YPDcl2	YPDcl3	-	EtOHcl2	Galcl1	YPDnc1	YPDnc2	YPDnc3	-	EtOHnc1	-	Galnc1	Galnc2

10		YDR194W-[illegible]	-	YPDcl3	-	EtOHcl2	Galcl1	YPDnc1	YPDnc2	YPDnc3	YPDnc4	EtOHnc1	-	Galnc1	Galnc2
10	VTC1	YER072W	-	YPDcl3	-	EtOHcl2	Galcl1	YPDnc1	-	YPDnc3	YPDnc4	EtOHnc1	EtOHnc2	Galnc1	Galnc2
10		YLR437C-A	-	YPDcl3	-	EtOHcl2	-	YPDnc1	YPDnc2	YPDnc3	YPDnc4	EtOHnc1	EtOHnc2	Galnc1	Galnc2
10		YHR045W	YPDcl2	-	EtOHcl1	EtOHcl2	Galcl1	YPDnc1	-	-	YPDnc4	EtOHnc1	EtOHnc2	Galnc1	Galnc2
10	AIM24	YJR080C	YPDcl2	YPDcl3	EtOHcl1	-	Galcl1	YPDnc1	YPDnc2	YPDnc3	YPDnc4	EtOHnc1	EtOHnc2	-	-
10		YER158C	-	YPDcl3	EtOHcl1	EtOHcl2	Galcl1	YPDnc1	YPDnc2	-	YPDnc4	EtOHnc1	-	Galnc1	Galnc2
10		YDR210W	-	-	EtOHcl1	-	Galcl1	YPDnc1	YPDnc2	YPDnc3	YPDnc4	EtOHnc1	EtOHnc2	Galnc1	Galnc2
10	RSC2	YLR357W	YPDcl2	YPDcl3	EtOHcl1	-	Galcl1	YPDnc1	YPDnc2	-	YPDnc4	EtOHnc1	EtOHnc2	Galnc1	-
10	CUR1	YPR158W	YPDcl2	YPDcl3	-	-	-	YPDnc1	YPDnc2	YPDnc3	YPDnc4	EtOHnc1	EtOHnc2	Galnc1	Galnc2
10	HSP150	YJL159W	-	-	EtOHcl1	-	Galcl1	YPDnc1	YPDnc2	YPDnc3	YPDnc4	EtOHnc1	EtOHnc2	Galnc1	Galnc2
10	UBP14	YBR058C	YPDcl2	YPDcl3	EtOHcl1	EtOHcl2	Galcl1	-	YPDnc2	YPDnc3	YPDnc4	-	EtOHnc2	-	Galnc2
10	PHO11	YAR071W	YPDcl2	-	EtOHcl1	EtOHcl2	Galcl1	YPDnc1	YPDnc2	YPDnc3	YPDnc4	-	EtOHnc2	Galnc1	-
10	ATP5	YDR298C	YPDcl2	YPDcl3	-	-	Galcl1	YPDnc1	YPDnc2	YPDnc3	YPDnc4	EtOHnc1	-	Galnc1	Galnc2
10	SPG5	YMR191W	YPDcl2	YPDcl3	EtOHcl1	-	Galcl1	YPDnc1	YPDnc2	-	-	EtOHnc1	EtOHnc2	Galnc1	Galnc2
10	COX11	YPL132W	YPDcl2	-	-	-	Galcl1	YPDnc1	YPDnc2	YPDnc3	YPDnc4	EtOHnc1	EtOHnc2	Galnc1	Galnc2
10	MPC54	YOR177C	YPDcl2	YPDcl3	EtOHcl1	-	-	YPDnc1	YPDnc2	YPDnc3	YPDnc4	EtOHnc1	-	Galnc1	Galnc2
10		YMR102C	YPDcl2	YPDcl3	EtOHcl1	EtOHcl2	Galcl1	-	YPDnc2	-	YPDnc4	EtOHnc1	-	Galnc1	Galnc2
10		YOR387C	YPDcl2	YPDcl3	-	-	-	YPDnc1	YPDnc2	YPDnc3	YPDnc4	EtOHnc1	EtOHnc2	Galnc1	Galnc2
10		YER076C	YPDcl2	YPDcl3	EtOHcl1	EtOHcl2	Galcl1	YPDnc1	YPDnc2	YPDnc3	YPDnc4	-	-	Galnc1	-
10	SSB2	YNL209W	-	-	EtOHcl1	EtOHcl2	Galcl1	YPDnc1	YPDnc2	YPDnc3	YPDnc4	EtOHnc1	-	Galnc1	Galnc2
10	COT1	YOR316C	-	-	EtOHcl1	-	Galcl1	YPDnc1	YPDnc2	YPDnc3	YPDnc4	EtOHnc1	EtOHnc2	Galnc1	Galnc2
10		YLR326W	YPDcl2	YPDcl3	EtOHcl1	-	Galcl1	YPDnc1	YPDnc2	YPDnc3	YPDnc4	-	-	Galnc1	Galnc2
10	MET17	YLR303W	YPDcl2	-	EtOHcl1	-	Galcl1	YPDnc1	YPDnc2	YPDnc3	YPDnc4	EtOHnc1	-	Galnc1	Galnc2
10	MSN1	YOL116W	YPDcl2	-	EtOHcl1	EtOHcl2	Galcl1	YPDnc1	YPDnc2	-	-	EtOHnc1	EtOHnc2	Galnc1	Galnc2
10	KIP2	YPL155C	-	YPDcl3	-	EtOHcl2	Galcl1	-	YPDnc2	YPDnc3	YPDnc4	EtOHnc1	EtOHnc2	Galnc1	Galnc2
10	CDC13	YDL220C	YPDcl2	YPDcl3	-	EtOHcl2	Galcl1	YPDnc1	YPDnc2	-	YPDnc4	-	EtOHnc2	Galnc1	Galnc2
10		YNL260C	-	-	EtOHcl1	EtOHcl2	Galcl1	YPDnc1	YPDnc2	YPDnc3	YPDnc4	EtOHnc1	EtOHnc2	Galnc1	-
10	MRPL35	YDR322W	YPDcl2	-	EtOHcl1	-	-	YPDnc1	YPDnc2	YPDnc3	YPDnc4	EtOHnc1	EtOHnc2	Galnc1	Galnc2
10	RPS27B	YHR021C	-	YPDcl3	EtOHcl1	-	Galcl1	-	YPDnc2	YPDnc3	YPDnc4	EtOHnc1	EtOHnc2	Galnc1	Galnc2
10		YGL193C	YPDcl2	YPDcl3	EtOHcl1	-	Galcl1	YPDnc1	YPDnc2	YPDnc3	-	EtOHnc1	-	Galnc1	Galnc2
10	SLZ1	YNL196C	-	YPDcl3	-	EtOHcl2	Galcl1	YPDnc1	YPDnc2	YPDnc3	YPDnc4	EtOHnc1	-	Galnc1	Galnc2
10	PIR1	YKL164C	-	-	EtOHcl1	EtOHcl2	-	YPDnc1	YPDnc2	YPDnc3	YPDnc4	EtOHnc1	EtOHnc2	Galnc1	Galnc2
10	USV1	YPL230W	YPDcl2	YPDcl3	EtOHcl1	EtOHcl2	Galcl1	-	-	YPDnc3	-	EtOHnc1	EtOHnc2	Galnc1	Galnc2
10	ELG1	YOR144C	YPDcl2	-	EtOHcl1	EtOHcl2	Galcl1	YPDnc1	YPDnc2	YPDnc3	YPDnc4	-	EtOHnc2	Galnc1	-

10	SPC110	YDR356W	YPDcl2	-	EtOHcl1	-	Galcl1	YPDnc1	YPDnc2	YPDnc3	YPDnc4	EtOHnc1	-	Galnc1	Galnc2
10		YER077C	-	YPDcl3	EtOHcl1	-	Galcl1	-	YPDnc2	YPDnc3	YPDnc4	EtOHnc1	EtOHnc2	Galnc1	Galnc2
10	DBP3	YGL078C	YPDcl2	-	-	EtOHcl2	Galcl1	YPDnc1	YPDnc2	YPDnc3	YPDnc4	EtOHnc1	EtOHnc2	Galnc1	-
10	OCA1	YNL099C	YPDcl2	YPDcl3	-	EtOHcl2	Galcl1	YPDnc1	YPDnc2	YPDnc3	-	EtOHnc1	-	Galnc1	Galnc2
10	HXT1	YHR094C	-	YPDcl3	EtOHcl1	EtOHcl2	Galcl1	YPDnc1	-	-	YPDnc4	EtOHnc1	EtOHnc2	Galnc1	Galnc2
10		YIL102C	-	YPDcl3	EtOHcl1	EtOHcl2	-	YPDnc1	YPDnc2	YPDnc3	-	EtOHnc1	EtOHnc2	Galnc1	Galnc2
10	POL31	YJR006W	YPDcl2	-	EtOHcl1	EtOHcl2	Galcl1	-	YPDnc2	YPDnc3	YPDnc4	EtOHnc1	EtOHnc2	Galnc1	-
10		YNL195C	YPDcl2	YPDcl3	EtOHcl1	-	Galcl1	YPDnc1	YPDnc2	YPDnc3	YPDnc4	-	-	Galnc1	Galnc2
10	YTA6	YPL074W	YPDcl2	YPDcl3	EtOHcl1	EtOHcl2	Galcl1	-	YPDnc2	YPDnc3	YPDnc4	-	EtOHnc2	-	Galnc2
10	IMP1	YMR150C	-	-	EtOHcl1	EtOHcl2	Galcl1	YPDnc1	YPDnc2	YPDnc3	-	EtOHnc1	EtOHnc2	Galnc1	Galnc2
10	PIN2	YOR104W	YPDcl2	YPDcl3	EtOHcl1	-	Galcl1	-	YPDnc2	YPDnc3	YPDnc4	-	EtOHnc2	Galnc1	Galnc2
10	MRPL23	YOR150W	YPDcl2	YPDcl3	EtOHcl1	EtOHcl2	Galcl1	YPDnc1	-	-	-	EtOHnc1	EtOHnc2	Galnc1	Galnc2
10		YPR153W	YPDcl2	YPDcl3	EtOHcl1	EtOHcl2	Galcl1	-	-	-	YPDnc4	EtOHnc1	EtOHnc2	Galnc1	Galnc2
10	STH1	YIL126W	-	YPDcl3	-	EtOHcl2	Galcl1	YPDnc1	YPDnc2	YPDnc3	YPDnc4	EtOHnc1	-	Galnc1	Galnc2
10		YKL061W	YPDcl2	YPDcl3	EtOHcl1	EtOHcl2	-	YPDnc1	-	-	YPDnc4	EtOHnc1	EtOHnc2	Galnc1	Galnc2
10		YDR278C	YPDcl2	YPDcl3	EtOHcl1	-	Galcl1	-	YPDnc2	-	YPDnc4	EtOHnc1	EtOHnc2	Galnc1	Galnc2
10	NDE2	YDL085W	YPDcl2	YPDcl3	EtOHcl1	EtOHcl2	Galcl1	YPDnc1	-	-	-	EtOHnc1	EtOHnc2	Galnc1	Galnc2
10		YAR028W	-	-	EtOHcl1	-	Galcl1	YPDnc1	YPDnc2	YPDnc3	YPDnc4	EtOHnc1	EtOHnc2	Galnc1	Galnc2
10	PAN5	YHR063C	YPDcl2	-	EtOHcl1	-	Galcl1	YPDnc1	YPDnc2	YPDnc3	YPDnc4	EtOHnc1	-	Galnc1	Galnc2
10		YDL218W	YPDcl2	-	EtOHcl1	EtOHcl2	Galcl1	YPDnc1	YPDnc2	-	YPDnc4	EtOHnc1	EtOHnc2	Galnc1	-
10	MSL1	YIR009W	-	-	EtOHcl1	-	Galcl1	YPDnc1	YPDnc2	YPDnc3	YPDnc4	EtOHnc1	EtOHnc2	Galnc1	Galnc2
10	MRPS16	YPL013C	-	YPDcl3	EtOHcl1	EtOHcl2	Galcl1	YPDnc1	-	-	YPDnc4	EtOHnc1	EtOHnc2	Galnc1	Galnc2
10	MNN1	YER001W	YPDcl2	YPDcl3	EtOHcl1	EtOHcl2	Galcl1	YPDnc1	YPDnc2	-	YPDnc4	EtOHnc1	-	Galnc1	-
10	TPO1	YLL028W	-	YPDcl3	EtOHcl1	EtOHcl2	Galcl1	YPDnc1	-	-	YPDnc4	EtOHnc1	EtOHnc2	Galnc1	Galnc2
10	NSR1	YGR159C	YPDcl2	YPDcl3	EtOHcl1	-	Galcl1	-	YPDnc2	YPDnc3	YPDnc4	EtOHnc1	EtOHnc2	-	Galnc2
10	HUG1	YML058W-	-	-	EtOHcl1	EtOHcl2	Galcl1	YPDnc1	YPDnc2	YPDnc3	YPDnc4	EtOHnc1	EtOHnc2	-	Galnc2
10	PFA4	YOL003C	YPDcl2	YPDcl3	EtOHcl1	EtOHcl2	Galcl1	-	YPDnc2	-	YPDnc4	-	EtOHnc2	Galnc1	Galnc2
10	PEX10	YDR265W	-	-	EtOHcl1	EtOHcl2	-	YPDnc1	YPDnc2	YPDnc3	YPDnc4	EtOHnc1	EtOHnc2	Galnc1	Galnc2
10		YFL015C	YPDcl2	YPDcl3	-	EtOHcl2	Galcl1	YPDnc1	YPDnc2	YPDnc3	-	EtOHnc1	-	Galnc1	Galnc2
10	PSY4	YBL046W	YPDcl2	-	EtOHcl1	EtOHcl2	-	YPDnc1	YPDnc2	YPDnc3	YPDnc4	EtOHnc1	-	Galnc1	Galnc2
10	PHO23	YNL097C	-	YPDcl3	EtOHcl1	EtOHcl2	Galcl1	-	YPDnc2	YPDnc3	YPDnc4	EtOHnc1	EtOHnc2	Galnc1	-
10		YMR259C	YPDcl2	YPDcl3	EtOHcl1	EtOHcl2	Galcl1	-	YPDnc2	YPDnc3	-	EtOHnc1	EtOHnc2	Galnc1	-
10	VPS62	YGR141W	YPDcl2	YPDcl3	EtOHcl1	EtOHcl2	Galcl1	YPDnc1	-	-	-	EtOHnc1	EtOHnc2	Galnc1	Galnc2
10		YNL205C	-	-	EtOHcl1	-	Galcl1	YPDnc1	YPDnc2	YPDnc3	YPDnc4	EtOHnc1	EtOHnc2	Galnc1	Galnc2

10	RAD10	YML095C	YPDcl2	YPDcl3	EtOHcl1	EtOHcl2	Galcl1	-	YPDnc2	YPDnc3	YPDnc4	-	EtOHnc2	Galnc1	-
10	ARG82	YDR173C	YPDcl2	YPDcl3	EtOHcl1	-	Galcl1	YPDnc1	YPDnc2	YPDnc3	-	-	EtOHnc2	Galnc1	Galnc2
10	IMD3	YLR432W	-	-	EtOHcl1	EtOHcl2	Galcl1	YPDnc1	YPDnc2	YPDnc3	YPDnc4	EtOHnc1	-	Galnc1	Galnc2
10	FMP37	YGL080W	YPDcl2	YPDcl3	EtOHcl1	EtOHcl2	Galcl1	-	YPDnc2	YPDnc3	-	EtOHnc1	EtOHnc2	Galnc1	-
10	RGA2	YDR379W	YPDcl2	YPDcl3	EtOHcl1	-	Galcl1	YPDnc1	YPDnc2	YPDnc3	YPDnc4	-	EtOHnc2	-	Galnc2
10	FLC1	YPL221W	YPDcl2	-	EtOHcl1	-	Galcl1	YPDnc1	YPDnc2	YPDnc3	YPDnc4	EtOHnc1	EtOHnc2	Galnc1	-
10	ZDS2	YML109W	YPDcl2	YPDcl3	EtOHcl1	EtOHcl2	Galcl1	-	-	YPDnc3	YPDnc4	EtOHnc1	-	Galnc1	Galnc2
10	MSS4	YDR208W	YPDcl2	YPDcl3	EtOHcl1	EtOHcl2	-	YPDnc1	YPDnc2	YPDnc3	-	-	EtOHnc2	Galnc1	Galnc2
10	ENT1	YDL161W	YPDcl2	-	EtOHcl1	-	Galcl1	YPDnc1	-	YPDnc3	YPDnc4	EtOHnc1	EtOHnc2	Galnc1	Galnc2
10	OTU2	YHL013C	YPDcl2	YPDcl3	EtOHcl1	EtOHcl2	Galcl1	-	YPDnc2	YPDnc3	YPDnc4	-	EtOHnc2	Galnc1	-
10		YIL030W-A	YPDcl2	-	EtOHcl1	EtOHcl2	-	YPDnc1	YPDnc2	-	YPDnc4	EtOHnc1	EtOHnc2	Galnc1	Galnc2
10		YJL077W-B	-	-	EtOHcl1	EtOHcl2	-	YPDnc1	YPDnc2	YPDnc3	YPDnc4	EtOHnc1	EtOHnc2	Galnc1	Galnc2
10		YLR057W	-	YPDcl3	-	-	Galcl1	YPDnc1	YPDnc2	YPDnc3	YPDnc4	EtOHnc1	EtOHnc2	Galnc1	Galnc2
10	RUD3	YOR216C	-	-	EtOHcl1	EtOHcl2	Galcl1	YPDnc1	YPDnc2	YPDnc3	-	EtOHnc1	EtOHnc2	Galnc1	Galnc2
10	RRB1	YMR131C	YPDcl2	-	-	EtOHcl2	Galcl1	YPDnc1	YPDnc2	YPDnc3	YPDnc4	EtOHnc1	-	Galnc1	Galnc2
10	HAP5	YOR358W	-	-	EtOHcl1	EtOHcl2	-	YPDnc1	YPDnc2	YPDnc3	YPDnc4	EtOHnc1	EtOHnc2	Galnc1	Galnc2
10		YBR296C-A	YPDcl2	YPDcl3	EtOHcl1	-	-	YPDnc1	YPDnc2	YPDnc3	YPDnc4	EtOHnc1	-	Galnc1	Galnc2
10	MAL33	YBR297W	YPDcl2	YPDcl3	-	-	-	YPDnc1	YPDnc2	YPDnc3	YPDnc4	EtOHnc1	EtOHnc2	Galnc1	Galnc2
10	MCM1	YMR043W	YPDcl2	YPDcl3	-	EtOHcl2	Galcl1	YPDnc1	-	YPDnc3	YPDnc4	EtOHnc1	-	Galnc1	Galnc2
10		YER091C-A	-	-	EtOHcl1	-	Galcl1	YPDnc1	YPDnc2	YPDnc3	YPDnc4	EtOHnc1	EtOHnc2	Galnc1	Galnc2
10	COX5A	YNL052W	YPDcl2	YPDcl3	EtOHcl1	EtOHcl2	Galcl1	-	YPDnc2	YPDnc3	YPDnc4	-	EtOHnc2	Galnc1	-
10		YIR035C	-	-	EtOHcl1	EtOHcl2	-	YPDnc1	YPDnc2	YPDnc3	YPDnc4	EtOHnc1	EtOHnc2	Galnc1	Galnc2
10	TSR2	YLR435W	-	-	EtOHcl1	EtOHcl2	-	YPDnc1	YPDnc2	YPDnc3	YPDnc4	EtOHnc1	EtOHnc2	Galnc1	Galnc2
10		YLR108C	YPDcl2	YPDcl3	EtOHcl1	-	Galcl1	YPDnc1	-	-	YPDnc4	EtOHnc1	EtOHnc2	Galnc1	Galnc2
10	RPS13	YDR064W	YPDcl2	-	EtOHcl1	-	Galcl1	YPDnc1	YPDnc2	YPDnc3	YPDnc4	EtOHnc1	-	Galnc1	Galnc2
10		YKL071W	-	YPDcl3	EtOHcl1	EtOHcl2	Galcl1	YPDnc1	YPDnc2	YPDnc3	-	EtOHnc1	-	Galnc1	Galnc2
10	DAL80	YKR034W	YPDcl2	YPDcl3	-	-	Galcl1	YPDnc1	YPDnc2	YPDnc3	YPDnc4	EtOHnc1	-	Galnc1	Galnc2
10		YCR013C	YPDcl2	YPDcl3	EtOHcl1	EtOHcl2	Galcl1	YPDnc1	YPDnc2	YPDnc3	-	-	EtOHnc2	-	Galnc2
10	ECM32	YER176W	-	YPDcl3	EtOHcl1	EtOHcl2	Galcl1	YPDnc1	-	-	YPDnc4	EtOHnc1	EtOHnc2	Galnc1	Galnc2
10	LCB5	YLR260W	-	-	EtOHcl1	-	Galcl1	YPDnc1	YPDnc2	YPDnc3	YPDnc4	EtOHnc1	EtOHnc2	Galnc1	Galnc2
10	TCB1	YOR086C	YPDcl2	-	-	-	Galcl1	YPDnc1	YPDnc2	YPDnc3	YPDnc4	EtOHnc1	EtOHnc2	Galnc1	Galnc2
10	RRP15	YPR143W	-	-	EtOHcl1	-	Galcl1	YPDnc1	YPDnc2	YPDnc3	YPDnc4	EtOHnc1	EtOHnc2	Galnc1	Galnc2
10	COG3	YER157W	-	YPDcl3	EtOHcl1	EtOHcl2	Galcl1	YPDnc1	YPDnc2	-	YPDnc4	EtOHnc1	-	Galnc1	Galnc2
10	MSB4	YOL112W	YPDcl2	-	EtOHcl1	EtOHcl2	Galcl1	YPDnc1	YPDnc2	YPDnc3	-	EtOHnc1	-	Galnc1	Galnc2

10	ERG29	YMR134W	YPDcl2	-	-	-	Galcl1	YPDnc1	YPDnc2	YPDnc3	YPDnc4	EtOHnc1	EtOHnc2	Galnc1	Galnc2
10	PHO87	YCR037C	-	YPDcl3	EtOHcl1	EtOHcl2	Galcl1	YPDnc1	-	-	YPDnc4	EtOHnc1	EtOHnc2	Galnc1	Galnc2
10	ECM31	YBR176W	YPDcl2	-	EtOHcl1	-	-	YPDnc1	YPDnc2	YPDnc3	YPDnc4	EtOHnc1	EtOHnc2	Galnc1	Galnc2
10		YPR076W	-	-	EtOHcl1	EtOHcl2	Galcl1	YPDnc1	YPDnc2	-	YPDnc4	EtOHnc1	EtOHnc2	Galnc1	Galnc2
10		YMR057C	-	YPDcl3	EtOHcl1	-	-	YPDnc1	YPDnc2	YPDnc3	YPDnc4	EtOHnc1	EtOHnc2	Galnc1	Galnc2
10	YNK1	YKL067W	YPDcl2	YPDcl3	-	-	Galcl1	YPDnc1	YPDnc2	YPDnc3	YPDnc4	EtOHnc1	-	Galnc1	Galnc2
10	RPS9A	YPL081W	-	-	EtOHcl1	EtOHcl2	-	YPDnc1	YPDnc2	YPDnc3	YPDnc4	EtOHnc1	EtOHnc2	Galnc1	Galnc2
10	EXG2	YDR261C	-	-	-	EtOHcl2	Galcl1	YPDnc1	YPDnc2	YPDnc3	YPDnc4	EtOHnc1	EtOHnc2	Galnc1	Galnc2
10	NSE3	YDR288W	-	-	EtOHcl1	-	Galcl1	YPDnc1	YPDnc2	YPDnc3	YPDnc4	EtOHnc1	EtOHnc2	Galnc1	Galnc2
10	NFT1	YKR103W	-	-	EtOHcl1	-	Galcl1	YPDnc1	YPDnc2	YPDnc3	YPDnc4	EtOHnc1	EtOHnc2	Galnc1	Galnc2
10		YJL032W	YPDcl2	YPDcl3	EtOHcl1	EtOHcl2	Galcl1	-	YPDnc2	-	YPDnc4	EtOHnc1	EtOHnc2	Galnc1	-
10	SMA1	YPL027W	-	-	EtOHcl1	-	Galcl1	YPDnc1	YPDnc2	YPDnc3	YPDnc4	EtOHnc1	EtOHnc2	Galnc1	Galnc2
10	NCA2	YPR155C	-	YPDcl3	EtOHcl1	-	Galcl1	YPDnc1	YPDnc2	-	YPDnc4	EtOHnc1	EtOHnc2	Galnc1	Galnc2
10	PET8	YNL003C	YPDcl2	YPDcl3	EtOHcl1	EtOHcl2	Galcl1	-	YPDnc2	YPDnc3	-	EtOHnc1	EtOHnc2	-	Galnc2
10	GET3	YDL100C	YPDcl2	YPDcl3	EtOHcl1	EtOHcl2	Galcl1	YPDnc1	YPDnc2	-	-	EtOHnc1	-	Galnc1	Galnc2
10	PAA1	YDR071C	YPDcl2	-	EtOHcl1	EtOHcl2	Galcl1	YPDnc1	YPDnc2	YPDnc3	-	EtOHnc1	-	Galnc1	Galnc2
10		YFR034W-A	-	YPDcl3	-	EtOHcl2	Galcl1	YPDnc1	YPDnc2	-	YPDnc4	EtOHnc1	EtOHnc2	Galnc1	Galnc2
10		YDL022C-A	-	YPDcl3	EtOHcl1	EtOHcl2	Galcl1	-	YPDnc2	YPDnc3	-	EtOHnc1	EtOHnc2	Galnc1	Galnc2
10	SUR2	YDR297W	YPDcl2	YPDcl3	-	-	Galcl1	YPDnc1	YPDnc2	YPDnc3	YPDnc4	EtOHnc1	-	Galnc1	Galnc2
10		YCR001W	YPDcl2	-	EtOHcl1	-	Galcl1	-	YPDnc2	YPDnc3	YPDnc4	EtOHnc1	EtOHnc2	Galnc1	Galnc2
10	YEN1	YER041W	YPDcl2	YPDcl3	EtOHcl1	EtOHcl2	Galcl1	-	YPDnc2	YPDnc3	YPDnc4	-	EtOHnc2	-	Galnc2
10	STE24	YJR117W	YPDcl2	-	EtOHcl1	EtOHcl2	Galcl1	-	YPDnc2	YPDnc3	-	EtOHnc1	EtOHnc2	Galnc1	Galnc2
10	RTT103	YDR289C	YPDcl2	YPDcl3	EtOHcl1	-	Galcl1	-	YPDnc2	YPDnc3	YPDnc4	-	EtOHnc2	Galnc1	Galnc2
10	MPS1	YDL028C	YPDcl2	YPDcl3	-	EtOHcl2	Galcl1	-	YPDnc2	YPDnc3	YPDnc4	EtOHnc1	EtOHnc2	Galnc1	-
10	CIN8	YEL061C	-	YPDcl3	EtOHcl1	-	-	YPDnc1	YPDnc2	YPDnc3	YPDnc4	EtOHnc1	EtOHnc2	Galnc1	Galnc2
10	RRT15	YLR162W-A	YPDcl2	YPDcl3	EtOHcl1	EtOHcl2	Galcl1	YPDnc1	-	YPDnc3	-	EtOHnc1	EtOHnc2	Galnc1	-
10	HSP26	YBR072W	YPDcl2	YPDcl3	-	EtOHcl2	Galcl1	YPDnc1	YPDnc2	YPDnc3	YPDnc4	-	-	Galnc1	Galnc2
10		YHR180C-B	YPDcl2	YPDcl3	EtOHcl1	EtOHcl2	Galcl1	-	YPDnc2	-	YPDnc4	EtOHnc1	EtOHnc2	-	Galnc2
10	TGL1	YKL140W	YPDcl2	YPDcl3	EtOHcl1	EtOHcl2	Galcl1	YPDnc1	-	-	YPDnc4	EtOHnc1	-	Galnc1	Galnc2
10		YLR402W	YPDcl2	-	EtOHcl1	EtOHcl2	Galcl1	YPDnc1	YPDnc2	-	YPDnc4	EtOHnc1	-	Galnc1	Galnc2
10	SMX3	YPR182W	-	YPDcl3	EtOHcl1	EtOHcl2	Galcl1	YPDnc1	-	YPDnc3	-	EtOHnc1	EtOHnc2	Galnc1	Galnc2
10	RRN10	YBL025W	-	-	-	EtOHcl2	Galcl1	YPDnc1	YPDnc2	YPDnc3	YPDnc4	EtOHnc1	EtOHnc2	Galnc1	Galnc2
10	SHE1	YBL031W	-	YPDcl3	EtOHcl1	-	-	YPDnc1	YPDnc2	YPDnc3	YPDnc4	EtOHnc1	EtOHnc2	Galnc1	Galnc2
10	ALP1	YNL270C	-	-	EtOHcl1	-	Galcl1	YPDnc1	YPDnc2	YPDnc3	YPDnc4	EtOHnc1	EtOHnc2	Galnc1	Galnc2

10	NUP133	YKR082W	YPDcl2	YPDcl3	EtOHcl1	EtOHcl2	Galcl1	-	YPDnc2	YPDnc3	YPDnc4	-	EtOHnc2	Galnc1	-
10	MND1	YGL183C	YPDcl2	YPDcl3	-	EtOHcl2	Galcl1	YPDnc1	YPDnc2	YPDnc3	YPDnc4	EtOHnc1	-	Galnc1	-
10		YMR103C	YPDcl2	YPDcl3	-	-	Galcl1	YPDnc1	YPDnc2	-	YPDnc4	EtOHnc1	EtOHnc2	Galnc1	Galnc2
10	NGR1	YBR212W	YPDcl2	-	-	-	Galcl1	YPDnc1	YPDnc2	YPDnc3	YPDnc4	EtOHnc1	EtOHnc2	Galnc1	Galnc2
10	MLC1	YGL106W	YPDcl2	YPDcl3	EtOHcl1	EtOHcl2	-	YPDnc1	YPDnc2	YPDnc3	-	EtOHnc1	-	Galnc1	Galnc2
10	CDC31	YOR257W	YPDcl2	YPDcl3	EtOHcl1	EtOHcl2	Galcl1	-	YPDnc2	YPDnc3	YPDnc4	-	EtOHnc2	Galnc1	-
10	OPY2	YPR075C	-	-	EtOHcl1	EtOHcl2	Galcl1	YPDnc1	YPDnc2	-	YPDnc4	EtOHnc1	EtOHnc2	Galnc1	Galnc2
10	TAF12	YDR145W	YPDcl2	YPDcl3	EtOHcl1	EtOHcl2	Galcl1	YPDnc1	-	YPDnc3	YPDnc4	EtOHnc1	-	Galnc1	-
10		YLR412C-A	-	-	EtOHcl1	EtOHcl2	Galcl1	YPDnc1	-	YPDnc3	YPDnc4	EtOHnc1	EtOHnc2	Galnc1	Galnc2
10	PHO81	YGR233C	-	YPDcl3	EtOHcl1	-	-	YPDnc1	YPDnc2	YPDnc3	YPDnc4	EtOHnc1	EtOHnc2	Galnc1	Galnc2
10	HXT10	YFL011W	YPDcl2	YPDcl3	EtOHcl1	EtOHcl2	Galcl1	-	YPDnc2	YPDnc3	YPDnc4	-	EtOHnc2	Galnc1	-
10	YKE2	YLR200W	-	YPDcl3	-	-	Galcl1	YPDnc1	YPDnc2	YPDnc3	YPDnc4	EtOHnc1	EtOHnc2	Galnc1	Galnc2
10	YOX1	YML027W	YPDcl2	-	-	EtOHcl2	-	YPDnc1	YPDnc2	YPDnc3	YPDnc4	EtOHnc1	EtOHnc2	Galnc1	Galnc2
10		YPL114W	-	YPDcl3	EtOHcl1	-	Galcl1	YPDnc1	YPDnc2	YPDnc3	-	EtOHnc1	EtOHnc2	Galnc1	Galnc2
10		YBR226C	-	YPDcl3	EtOHcl1	EtOHcl2	Galcl1	YPDnc1	-	-	YPDnc4	EtOHnc1	EtOHnc2	Galnc1	Galnc2
10		YOR292C	-	-	EtOHcl1	-	Galcl1	YPDnc1	YPDnc2	YPDnc3	YPDnc4	EtOHnc1	EtOHnc2	Galnc1	Galnc2
10	IQG1	YPL242C	YPDcl2	YPDcl3	-	-	Galcl1	YPDnc1	YPDnc2	YPDnc3	YPDnc4	-	EtOHnc2	Galnc1	Galnc2
10	VPH2	YKL119C	YPDcl2	YPDcl3	EtOHcl1	EtOHcl2	Galcl1	-	-	-	YPDnc4	EtOHnc1	EtOHnc2	Galnc1	Galnc2
10	FAL1	YDR021W	YPDcl2	YPDcl3	EtOHcl1	EtOHcl2	Galcl1	-	YPDnc2	YPDnc3	YPDnc4	-	EtOHnc2	Galnc1	-
10	RAD5	YLR032W	-	-	-	EtOHcl2	Galcl1	YPDnc1	YPDnc2	YPDnc3	YPDnc4	EtOHnc1	EtOHnc2	Galnc1	Galnc2
10	HSP10	YOR020C	YPDcl2	-	-	EtOHcl2	Galcl1	YPDnc1	YPDnc2	YPDnc3	-	EtOHnc1	EtOHnc2	Galnc1	Galnc2
9		YCR061W	-	-	EtOHcl1	-	-	YPDnc1	YPDnc2	YPDnc3	YPDnc4	EtOHnc1	EtOHnc2	Galnc1	Galnc2
9	TIP1	YBR067C	-	-	EtOHcl1	EtOHcl2	-	YPDnc1	YPDnc2	YPDnc3	YPDnc4	EtOHnc1	-	Galnc1	Galnc2
9	BUB3	YOR026W	-	YPDcl3	EtOHcl1	EtOHcl2	Galcl1	YPDnc1	-	-	-	EtOHnc1	EtOHnc2	Galnc1	Galnc2
9	OAR1	YKL055C	-	YPDcl3	EtOHcl1	-	-	YPDnc1	YPDnc2	YPDnc3	-	EtOHnc1	EtOHnc2	Galnc1	Galnc2
9	MCM4	YPR019W	YPDcl2	-	-	-	Galcl1	YPDnc1	YPDnc2	YPDnc3	YPDnc4	EtOHnc1	EtOHnc2	Galnc1	-
9		YPL014W	-	YPDcl3	-	-	Galcl1	YPDnc1	-	YPDnc3	YPDnc4	EtOHnc1	EtOHnc2	Galnc1	Galnc2
9		YER119C-A	YPDcl2	-	EtOHcl1	-	-	YPDnc1	YPDnc2	YPDnc3	YPDnc4	EtOHnc1	-	Galnc1	Galnc2
9	TOM40	YMR203W	YPDcl2	YPDcl3	EtOHcl1	EtOHcl2	Galcl1	-	-	-	YPDnc4	EtOHnc1	EtOHnc2	-	Galnc2
9	TRL1	YJL087C	YPDcl2	YPDcl3	EtOHcl1	EtOHcl2	Galcl1	-	YPDnc2	YPDnc3	YPDnc4	-	EtOHnc2	-	-
9	PRE8	YML092C	-	YPDcl3	EtOHcl1	EtOHcl2	Galcl1	YPDnc1	YPDnc2	YPDnc3	YPDnc4	EtOHnc1	-	-	-
9	GNA1	YFL017C	YPDcl2	YPDcl3	EtOHcl1	EtOHcl2	Galcl1	-	-	-	-	EtOHnc1	EtOHnc2	Galnc1	Galnc2
9		YMR086C-A	YPDcl2	-	EtOHcl1	-	Galcl1	YPDnc1	YPDnc2	YPDnc3	YPDnc4	-	-	Galnc1	Galnc2
9	RGS2	YOR107W	-	-	-	EtOHcl2	Galcl1	YPDnc1	YPDnc2	YPDnc3	-	EtOHnc1	EtOHnc2	Galnc1	Galnc2

9		YLR012C	YPDcl2	YPDcl3	-	-	Galcl1	-	YPDnc2	YPDnc3	YPDnc4	EtOHnc1	EtOHnc2	Galnc1	-
9	IES1	YFL013C	YPDcl2	YPDcl3	EtOHcl1	EtOHcl2	Galcl1	-	YPDnc2	YPDnc3	YPDnc4	-	EtOHnc2	-	-
9	ECM13	YBL043W	-	-	-	EtOHcl2	Galcl1	YPDnc1	YPDnc2	YPDnc3	YPDnc4	EtOHnc1	EtOHnc2	Galnc1	-
9	HRK1	YOR267C	-	YPDcl3	EtOHcl1	-	Galcl1	YPDnc1	YPDnc2	-	YPDnc4	EtOHnc1	-	Galnc1	Galnc2
9	DIA1	YMR316W	-	-	EtOHcl1	-	Galcl1	YPDnc1	YPDnc2	YPDnc3	-	EtOHnc1	EtOHnc2	Galnc1	Galnc2
9	UPF3	YGR072W	-	-	EtOHcl1	EtOHcl2	Galcl1	YPDnc1	YPDnc2	YPDnc3	YPDnc4	-	EtOHnc2	-	Galnc2
9	MDE1	YJR024C	YPDcl2	YPDcl3	-	-	Galcl1	YPDnc1	-	-	YPDnc4	EtOHnc1	EtOHnc2	Galnc1	Galnc2
9	FEN2	YCR028C	YPDcl2	-	EtOHcl1	-	Galcl1	YPDnc1	YPDnc2	YPDnc3	YPDnc4	-	EtOHnc2	-	Galnc2
9	KAR2	YJL034W	-	-	-	EtOHcl2	Galcl1	YPDnc1	YPDnc2	YPDnc3	-	EtOHnc1	EtOHnc2	Galnc1	Galnc2
9	SRP40	YKR092C	YPDcl2	-	EtOHcl1	-	Galcl1	YPDnc1	YPDnc2	-	YPDnc4	EtOHnc1	-	Galnc1	Galnc2
9	FUN14	YAL008W	YPDcl2	YPDcl3	EtOHcl1	EtOHcl2	Galcl1	YPDnc1	-	-	-	EtOHnc1	EtOHnc2	Galnc1	-
9	ULP1	YPL020C	YPDcl2	YPDcl3	EtOHcl1	-	-	YPDnc1	-	-	YPDnc4	EtOHnc1	EtOHnc2	Galnc1	Galnc2
9	MSP1	YGR028W	YPDcl2	YPDcl3	-	EtOHcl2	Galcl1	YPDnc1	-	-	YPDnc4	EtOHnc1	EtOHnc2	-	Galnc2
9	ARP1	YHR129C	YPDcl2	YPDcl3	EtOHcl1	EtOHcl2	Galcl1	-	YPDnc2	YPDnc3	YPDnc4	-	EtOHnc2	-	-
9	LYP1	YNL268W	-	-	EtOHcl1	EtOHcl2	Galcl1	YPDnc1	-	YPDnc3	-	EtOHnc1	EtOHnc2	Galnc1	Galnc2
9		YOR345C	-	-	EtOHcl1	-	Galcl1	YPDnc1	YPDnc2	YPDnc3	-	EtOHnc1	EtOHnc2	Galnc1	Galnc2
9	FYV6	YNL133C	-	-	EtOHcl1	EtOHcl2	Galcl1	YPDnc1	-	YPDnc3	-	EtOHnc1	EtOHnc2	Galnc1	Galnc2
9	SES1	YDR023W	-	-	EtOHcl1	-	-	YPDnc1	YPDnc2	YPDnc3	YPDnc4	EtOHnc1	EtOHnc2	Galnc1	Galnc2
9	SPG3	YDR504C	-	-	EtOHcl1	-	Galcl1	YPDnc1	YPDnc2	-	YPDnc4	EtOHnc1	EtOHnc2	Galnc1	Galnc2
9	PSY1	YKL076C	-	-	EtOHcl1	-	-	YPDnc1	YPDnc2	YPDnc3	YPDnc4	EtOHnc1	EtOHnc2	Galnc1	Galnc2
9		YMR119W-	-	YPDcl3	EtOHcl1	EtOHcl2	Galcl1	YPDnc1	-	-	-	EtOHnc1	EtOHnc2	Galnc1	Galnc2
9		YFR035C	YPDcl2	YPDcl3	-	-	-	YPDnc1	YPDnc2	-	YPDnc4	EtOHnc1	EtOHnc2	Galnc1	Galnc2
9	MRM2	YGL136C	YPDcl2	YPDcl3	-	EtOHcl2	-	YPDnc1	YPDnc2	-	YPDnc4	EtOHnc1	EtOHnc2	-	Galnc2
9	MGM101	YJR144W	-	-	EtOHcl1	EtOHcl2	-	YPDnc1	-	YPDnc3	YPDnc4	EtOHnc1	EtOHnc2	Galnc1	Galnc2
9	ENP2	YGR145W	YPDcl2	-	-	-	Galcl1	-	YPDnc2	YPDnc3	YPDnc4	EtOHnc1	EtOHnc2	Galnc1	Galnc2
9		YCR095W-/	YPDcl2	-	EtOHcl1	-	-	-	YPDnc2	YPDnc3	YPDnc4	EtOHnc1	EtOHnc2	Galnc1	Galnc2
9		YMR210W	-	-	EtOHcl1	-	Galcl1	YPDnc1	YPDnc2	YPDnc3	-	EtOHnc1	EtOHnc2	Galnc1	Galnc2
9	DIC1	YLR348C	-	YPDcl3	EtOHcl1	EtOHcl2	Galcl1	YPDnc1	-	YPDnc3	-	EtOHnc1	EtOHnc2	-	Galnc2
9	RCK1	YGL158W	-	-	EtOHcl1	EtOHcl2	Galcl1	YPDnc1	YPDnc2	-	-	EtOHnc1	EtOHnc2	Galnc1	Galnc2
9	RPC19	YNL113W	-	-	EtOHcl1	-	Galcl1	YPDnc1	YPDnc2	YPDnc3	-	EtOHnc1	EtOHnc2	Galnc1	Galnc2
9	PDR12	YPL058C	-	-	EtOHcl1	-	Galcl1	YPDnc1	YPDnc2	YPDnc3	-	EtOHnc1	EtOHnc2	Galnc1	Galnc2
9	PAB1	YER165W	-	YPDcl3	EtOHcl1	EtOHcl2	Galcl1	-	YPDnc2	YPDnc3	YPDnc4	-	EtOHnc2	Galnc1	-
9	TMA10	YLR327C	-	-	EtOHcl1	-	Galcl1	YPDnc1	YPDnc2	YPDnc3	YPDnc4	EtOHnc1	-	Galnc1	Galnc2
9		YOR139C	-	-	EtOHcl1	-	-	YPDnc1	YPDnc2	YPDnc3	YPDnc4	EtOHnc1	EtOHnc2	Galnc1	Galnc2

9	DSE4	YNR067C	-	-	EtOHcl1	EtOHcl2	Galcl1	-	YPDnc2	-	YPDnc4	EtOHnc1	EtOHnc2	Galnc1	Galnc2
9	SBP1	YHL034C	-	-	EtOHcl1	-	Galcl1	YPDnc1	YPDnc2	YPDnc3	YPDnc4	EtOHnc1	EtOHnc2	Galnc1	-
9		YGL063C-A	YPDcl2	YPDcl3	EtOHcl1	-	-	-	YPDnc2	YPDnc3	YPDnc4	EtOHnc1	-	Galnc1	Galnc2
9	AQR1	YNL065W	YPDcl2	-	EtOHcl1	EtOHcl2	Galcl1	YPDnc1	-	-	-	EtOHnc1	EtOHnc2	Galnc1	Galnc2
9	ERG28	YER044C	YPDcl2	YPDcl3	EtOHcl1	EtOHcl2	Galcl1	-	YPDnc2	-	YPDnc4	-	EtOHnc2	-	Galnc2
9		YJL202C	YPDcl2	YPDcl3	-	EtOHcl2	Galcl1	-	YPDnc2	YPDnc3	YPDnc4	EtOHnc1	-	Galnc1	-
9		YLR365W	-	-	EtOHcl1	-	Galcl1	YPDnc1	YPDnc2	YPDnc3	-	EtOHnc1	EtOHnc2	Galnc1	Galnc2
9		YHR127W	-	-	-	-	Galcl1	YPDnc1	YPDnc2	YPDnc3	YPDnc4	EtOHnc1	EtOHnc2	Galnc1	Galnc2
9		YNL144C	-	YPDcl3	EtOHcl1	EtOHcl2	Galcl1	YPDnc1	-	-	-	EtOHnc1	EtOHnc2	Galnc1	Galnc2
9	CYM1	YDR430C	-	YPDcl3	-	EtOHcl2	Galcl1	YPDnc1	-	-	YPDnc4	EtOHnc1	EtOHnc2	Galnc1	Galnc2
9		YDR521W	YPDcl2	YPDcl3	EtOHcl1	EtOHcl2	Galcl1	-	YPDnc2	YPDnc3	YPDnc4	-	-	Galnc1	-
9		YLR179C	-	-	EtOHcl1	-	-	YPDnc1	YPDnc2	YPDnc3	YPDnc4	EtOHnc1	EtOHnc2	Galnc1	Galnc2
9	MUM3	YOR298W	YPDcl2	YPDcl3	EtOHcl1	EtOHcl2	Galcl1	-	-	-	-	EtOHnc1	EtOHnc2	Galnc1	Galnc2
9	TPK2	YPL203W	-	-	EtOHcl1	EtOHcl2	Galcl1	YPDnc1	-	-	YPDnc4	EtOHnc1	EtOHnc2	Galnc1	Galnc2
9	MSI1	YBR195C	-	YPDcl3	-	EtOHcl2	-	YPDnc1	YPDnc2	YPDnc3	YPDnc4	-	EtOHnc2	Galnc1	Galnc2
9	GAL3	YDR009W	YPDcl2	-	EtOHcl1	EtOHcl2	Galcl1	YPDnc1	-	-	YPDnc4	EtOHnc1	-	Galnc1	Galnc2
9	RIA1	YNL163C	YPDcl2	YPDcl3	EtOHcl1	EtOHcl2	Galcl1	-	YPDnc2	YPDnc3	YPDnc4	-	EtOHnc2	-	-
9	SCS2	YER120W	YPDcl2	-	EtOHcl1	-	-	YPDnc1	YPDnc2	YPDnc3	YPDnc4	EtOHnc1	-	Galnc1	Galnc2
9	YHM2	YMR241W	YPDcl2	-	EtOHcl1	EtOHcl2	-	YPDnc1	-	YPDnc3	-	EtOHnc1	EtOHnc2	Galnc1	Galnc2
9	RPS22B	YLR367W	YPDcl2	YPDcl3	-	EtOHcl2	Galcl1	YPDnc1	-	YPDnc3	-	EtOHnc1	-	Galnc1	Galnc2
9	SNZ1	YMR096W	YPDcl2	YPDcl3	-	EtOHcl2	Galcl1	-	YPDnc2	YPDnc3	YPDnc4	EtOHnc1	-	Galnc1	-
9	RMR1	YGL250W	YPDcl2	-	EtOHcl1	EtOHcl2	Galcl1	YPDnc1	-	YPDnc3	YPDnc4	EtOHnc1	EtOHnc2	-	-
9	NTH1	YDR001C	YPDcl2	YPDcl3	-	EtOHcl2	Galcl1	YPDnc1	-	-	-	EtOHnc1	EtOHnc2	Galnc1	Galnc2
9		YKR070W	YPDcl2	YPDcl3	EtOHcl1	EtOHcl2	Galcl1	-	-	-	YPDnc4	EtOHnc1	-	Galnc1	Galnc2
9	REE1	YJL217W	-	-	EtOHcl1	-	Galcl1	YPDnc1	YPDnc2	YPDnc3	-	EtOHnc1	EtOHnc2	Galnc1	Galnc2
9	MTC2	YKL098W	-	YPDcl3	EtOHcl1	EtOHcl2	Galcl1	YPDnc1	YPDnc2	YPDnc3	-	EtOHnc1	EtOHnc2	-	-
9		YHR069C-A	-	YPDcl3	EtOHcl1	EtOHcl2	Galcl1	YPDnc1	-	-	-	EtOHnc1	EtOHnc2	Galnc1	Galnc2
9	UGO1	YDR470C	YPDcl2	-	EtOHcl1	EtOHcl2	Galcl1	-	-	-	YPDnc4	EtOHnc1	EtOHnc2	Galnc1	Galnc2
9	RAD53	YPL153C	-	-	-	-	Galcl1	YPDnc1	YPDnc2	YPDnc3	YPDnc4	EtOHnc1	EtOHnc2	Galnc1	Galnc2
9	ATP10	YLR393W	-	-	-	-	Galcl1	YPDnc1	YPDnc2	YPDnc3	YPDnc4	EtOHnc1	EtOHnc2	Galnc1	Galnc2
9		YDR431W	-	YPDcl3	-	EtOHcl2	Galcl1	YPDnc1	-	-	YPDnc4	EtOHnc1	EtOHnc2	Galnc1	Galnc2
9	EFG1	YGR271C-A	-	YPDcl3	EtOHcl1	-	Galcl1	YPDnc1	YPDnc2	YPDnc3	YPDnc4	-	-	Galnc1	Galnc2
9	MVB12	YGR206W	YPDcl2	YPDcl3	-	EtOHcl2	Galcl1	-	YPDnc2	-	YPDnc4	EtOHnc1	EtOHnc2	-	Galnc2
9	ARF1	YDL192W	YPDcl2	-	EtOHcl1	-	Galcl1	YPDnc1	YPDnc2	-	YPDnc4	EtOHnc1	EtOHnc2	Galnc1	-

9	RPS22A	YJL190C	-	YPDcl3	EtOHcl1	EtOHcl2	-	-	YPDnc2	YPDnc3	-	EtOHnc1	EtOHnc2	Galnc1	Galnc2
9	YPT35	YHR105W	-	YPDcl3	EtOHcl1	-	Galcl1	YPDnc1	YPDnc2	YPDnc3	YPDnc4	-	EtOHnc2	Galnc1	-
9	THR4	YCR053W	-	YPDcl3	EtOHcl1	-	-	YPDnc1	-	YPDnc3	YPDnc4	EtOHnc1	EtOHnc2	Galnc1	Galnc2
9	DOA4	YDR069C	YPDcl2	YPDcl3	EtOHcl1	-	Galcl1	YPDnc1	-	YPDnc3	YPDnc4	-	-	Galnc1	Galnc2
9	HOS4	YIL112W	-	-	EtOHcl1	-	Galcl1	-	YPDnc2	YPDnc3	YPDnc4	EtOHnc1	EtOHnc2	Galnc1	Galnc2
9	LRG1	YDL240W	-	YPDcl3	-	-	Galcl1	YPDnc1	-	YPDnc3	YPDnc4	EtOHnc1	EtOHnc2	Galnc1	Galnc2
9	TAD2	YJL035C	-	-	EtOHcl1	EtOHcl2	Galcl1	YPDnc1	-	-	YPDnc4	EtOHnc1	EtOHnc2	Galnc1	Galnc2
9		YGL039W	-	-	-	-	Galcl1	YPDnc1	YPDnc2	YPDnc3	YPDnc4	EtOHnc1	EtOHnc2	Galnc1	Galnc2
9	SUB2	YDL084W	YPDcl2	YPDcl3	-	-	Galcl1	-	YPDnc2	YPDnc3	YPDnc4	EtOHnc1	EtOHnc2	-	Galnc2
9		YAR029W	YPDcl2	YPDcl3	EtOHcl1	EtOHcl2	Galcl1	-	YPDnc2	YPDnc3	YPDnc4	-	-	Galnc1	-
9		YGR240C-A	YPDcl2	-	EtOHcl1	-	Galcl1	YPDnc1	-	YPDnc3	-	EtOHnc1	EtOHnc2	Galnc1	Galnc2
9		YGR182C	-	-	EtOHcl1	-	Galcl1	YPDnc1	YPDnc2	YPDnc3	YPDnc4	EtOHnc1	-	Galnc1	Galnc2
9		YKL215C	YPDcl2	-	EtOHcl1	-	Galcl1	YPDnc1	YPDnc2	-	YPDnc4	EtOHnc1	-	Galnc1	Galnc2
9		YNL067W-/	-	YPDcl3	-	EtOHcl2	Galcl1	YPDnc1	YPDnc2	-	-	EtOHnc1	EtOHnc2	Galnc1	Galnc2
9		YER006C-A	YPDcl2	YPDcl3	EtOHcl1	EtOHcl2	Galcl1	-	-	-	-	EtOHnc1	EtOHnc2	Galnc1	Galnc2
9		YBR062C	-	YPDcl3	EtOHcl1	-	Galcl1	-	YPDnc2	YPDnc3	YPDnc4	EtOHnc1	EtOHnc2	Galnc1	-
9	TIF6	YPR016C	YPDcl2	-	-	-	Galcl1	-	YPDnc2	YPDnc3	YPDnc4	EtOHnc1	EtOHnc2	Galnc1	Galnc2
9		YPL067C	YPDcl2	YPDcl3	EtOHcl1	-	Galcl1	YPDnc1	-	YPDnc3	-	EtOHnc1	-	Galnc1	Galnc2
9	DFG5	YMR238W	-	-	EtOHcl1	EtOHcl2	Galcl1	-	YPDnc2	YPDnc3	-	EtOHnc1	EtOHnc2	Galnc1	Galnc2
9	RMD8	YFR048W	YPDcl2	YPDcl3	EtOHcl1	EtOHcl2	Galcl1	YPDnc1	-	-	-	EtOHnc1	-	Galnc1	Galnc2
9	PMP3	YDR276C	YPDcl2	YPDcl3	EtOHcl1	EtOHcl2	-	YPDnc1	-	-	YPDnc4	EtOHnc1	-	Galnc1	Galnc2
9	RPP2B	YDR382W	-	-	EtOHcl1	-	Galcl1	YPDnc1	YPDnc2	-	YPDnc4	EtOHnc1	EtOHnc2	Galnc1	Galnc2
9	CKI1	YLR133W	YPDcl2	YPDcl3	EtOHcl1	-	Galcl1	YPDnc1	YPDnc2	-	YPDnc4	EtOHnc1	-	-	Galnc2
9	CDC27	YBL084C	-	-	EtOHcl1	-	Galcl1	YPDnc1	YPDnc2	YPDnc3	YPDnc4	EtOHnc1	EtOHnc2	Galnc1	-
9	RRP45	YDR280W	-	-	EtOHcl1	EtOHcl2	-	YPDnc1	YPDnc2	YPDnc3	YPDnc4	-	EtOHnc2	Galnc1	Galnc2
9	PYC2	YBR218C	YPDcl2	YPDcl3	EtOHcl1	-	-	YPDnc1	YPDnc2	YPDnc3	YPDnc4	-	EtOHnc2	-	Galnc2
9	MST28	YAR033W	-	-	EtOHcl1	EtOHcl2	-	YPDnc1	YPDnc2	YPDnc3	-	EtOHnc1	EtOHnc2	Galnc1	Galnc2
9		YBR284W	YPDcl2	YPDcl3	-	-	Galcl1	YPDnc1	YPDnc2	YPDnc3	YPDnc4	-	-	Galnc1	Galnc2
9		YDR183C-A	YPDcl2	-	EtOHcl1	EtOHcl2	Galcl1	-	YPDnc2	-	-	EtOHnc1	EtOHnc2	Galnc1	Galnc2
9		YER079W	-	-	EtOHcl1	EtOHcl2	-	YPDnc1	YPDnc2	YPDnc3	-	EtOHnc1	EtOHnc2	Galnc1	Galnc2
9	SHS1	YDL225W	YPDcl2	YPDcl3	EtOHcl1	-	-	YPDnc1	-	YPDnc3	-	EtOHnc1	EtOHnc2	Galnc1	Galnc2
9	POP6	YGR030C	YPDcl2	YPDcl3	EtOHcl1	EtOHcl2	Galcl1	YPDnc1	-	-	YPDnc4	EtOHnc1	-	-	Galnc2
9	ADK1	YDR226W	YPDcl2	-	-	EtOHcl2	-	YPDnc1	YPDnc2	YPDnc3	YPDnc4	EtOHnc1	-	Galnc1	Galnc2
9		YLR399W-/	-	YPDcl3	EtOHcl1	-	-	YPDnc1	YPDnc2	YPDnc3	-	EtOHnc1	EtOHnc2	Galnc1	Galnc2

9	ATG12	YBR217W	-	YPDcl3	-	-	Galcl1	YPDnc1	YPDnc2	YPDnc3	YPDnc4	EtOHnc1	-	Galnc1	Galnc2
9	MER1	YNL210W	YPDcl2	YPDcl3	EtOHcl1	EtOHcl2	Galcl1	-	YPDnc2	YPDnc3	YPDnc4	-	-	Galnc1	-
9	GLC7	YER133W	YPDcl2	-	EtOHcl1	-	Galcl1	YPDnc1	-	YPDnc3	-	EtOHnc1	EtOHnc2	Galnc1	Galnc2
9		YHL044W	-	-	EtOHcl1	-	-	YPDnc1	YPDnc2	YPDnc3	YPDnc4	EtOHnc1	EtOHnc2	Galnc1	Galnc2
9	HSP30	YCR021C	-	YPDcl3	EtOHcl1	-	Galcl1	YPDnc1	-	-	YPDnc4	EtOHnc1	EtOHnc2	Galnc1	Galnc2
9	CHS2	YBR038W	YPDcl2	-	-	-	Galcl1	YPDnc1	YPDnc2	YPDnc3	YPDnc4	EtOHnc1	-	Galnc1	Galnc2
9	EFT2	YDR385W	-	YPDcl3	EtOHcl1	-	Galcl1	YPDnc1	-	-	YPDnc4	EtOHnc1	EtOHnc2	Galnc1	Galnc2
9	RPL11A	YPR102C	YPDcl2	YPDcl3	EtOHcl1	-	Galcl1	YPDnc1	-	-	-	EtOHnc1	EtOHnc2	Galnc1	Galnc2
9	GCD11	YER025W	YPDcl2	YPDcl3	EtOHcl1	EtOHcl2	Galcl1	YPDnc1	YPDnc2	-	YPDnc4	-	-	Galnc1	-
9	MTR10	YOR160W	YPDcl2	YPDcl3	EtOHcl1	EtOHcl2	Galcl1	-	YPDnc2	YPDnc3	-	-	EtOHnc2	-	Galnc2
9		YGR026W	YPDcl2	YPDcl3	-	EtOHcl2	-	YPDnc1	YPDnc2	YPDnc3	-	EtOHnc1	EtOHnc2	Galnc1	-
9	RPL4A	YBR031W	YPDcl2	YPDcl3	EtOHcl1	-	Galcl1	YPDnc1	YPDnc2	YPDnc3	YPDnc4	-	EtOHnc2	-	-
9	KRE11	YGR166W	YPDcl2	-	EtOHcl1	-	Galcl1	YPDnc1	YPDnc2	-	YPDnc4	EtOHnc1	-	Galnc1	Galnc2
9	AFG3	YER017C	-	YPDcl3	EtOHcl1	EtOHcl2	-	YPDnc1	YPDnc2	YPDnc3	-	-	EtOHnc2	Galnc1	Galnc2
9	YMR31	YFR049W	YPDcl2	YPDcl3	EtOHcl1	-	Galcl1	YPDnc1	-	YPDnc3	-	EtOHnc1	-	Galnc1	Galnc2
9		YPL113C	-	YPDcl3	EtOHcl1	EtOHcl2	Galcl1	YPDnc1	-	YPDnc3	-	EtOHnc1	-	Galnc1	Galnc2
9	IRA1	YBR140C	-	YPDcl3	-	EtOHcl2	Galcl1	YPDnc1	-	YPDnc3	YPDnc4	-	EtOHnc2	Galnc1	Galnc2
9	UTP13	YLR222C	YPDcl2	YPDcl3	EtOHcl1	EtOHcl2	Galcl1	-	YPDnc2	YPDnc3	YPDnc4	-	EtOHnc2	-	-
9		YMR316C-A	-	-	EtOHcl1	-	Galcl1	YPDnc1	YPDnc2	YPDnc3	-	EtOHnc1	EtOHnc2	Galnc1	Galnc2
9	PUB1	YNL016W	YPDcl2	-	EtOHcl1	-	Galcl1	YPDnc1	YPDnc2	YPDnc3	-	EtOHnc1	-	Galnc1	Galnc2
9	CWH43	YCR017C	YPDcl2	-	EtOHcl1	EtOHcl2	Galcl1	-	-	-	YPDnc4	EtOHnc1	EtOHnc2	Galnc1	Galnc2
9	DJP1	YIR004W	YPDcl2	-	EtOHcl1	EtOHcl2	Galcl1	-	-	YPDnc3	-	EtOHnc1	EtOHnc2	Galnc1	Galnc2
9	RPL11B	YGR085C	YPDcl2	YPDcl3	EtOHcl1	EtOHcl2	Galcl1	YPDnc1	YPDnc2	-	-	-	-	Galnc1	Galnc2
9	NUS1	YDL193W	YPDcl2	-	EtOHcl1	-	Galcl1	YPDnc1	YPDnc2	-	YPDnc4	EtOHnc1	EtOHnc2	Galnc1	-
9		YGR127W	-	-	-	EtOHcl2	Galcl1	YPDnc1	YPDnc2	YPDnc3	-	EtOHnc1	EtOHnc2	Galnc1	Galnc2
9		YOR199W	-	-	EtOHcl1	-	-	YPDnc1	YPDnc2	YPDnc3	YPDnc4	EtOHnc1	EtOHnc2	Galnc1	Galnc2
9	IRC10	YOL015W	-	YPDcl3	EtOHcl1	-	Galcl1	-	-	YPDnc3	YPDnc4	EtOHnc1	EtOHnc2	Galnc1	Galnc2
9	NDJ1	YOL104C	-	YPDcl3	-	-	Galcl1	YPDnc1	YPDnc2	YPDnc3	YPDnc4	EtOHnc1	-	Galnc1	Galnc2
9		YBR224W	-	-	-	EtOHcl2	Galcl1	YPDnc1	YPDnc2	YPDnc3	-	EtOHnc1	EtOHnc2	Galnc1	Galnc2
9		YCL022C	-	-	EtOHcl1	-	-	YPDnc1	YPDnc2	YPDnc3	YPDnc4	EtOHnc1	EtOHnc2	Galnc1	Galnc2
9	ERG10	YPL028W	-	YPDcl3	EtOHcl1	-	Galcl1	-	YPDnc2	YPDnc3	YPDnc4	-	EtOHnc2	Galnc1	Galnc2
9		YBL044W	-	-	EtOHcl1	-	-	YPDnc1	YPDnc2	YPDnc3	YPDnc4	EtOHnc1	EtOHnc2	Galnc1	Galnc2
9	LEU3	YLR451W	-	-	EtOHcl1	-	Galcl1	YPDnc1	YPDnc2	YPDnc3	-	EtOHnc1	EtOHnc2	Galnc1	Galnc2
9		YFL013W-A	YPDcl2	YPDcl3	-	EtOHcl2	Galcl1	YPDnc1	-	-	-	EtOHnc1	EtOHnc2	Galnc1	Galnc2

9	URE2	YNL229C	YPDcl2	-	EtOHcl1	-	Galcl1	YPDnc1	YPDnc2	-	YPDnc4	EtOHnc1	EtOHnc2	Galnc1	-
9	AAR2	YBL074C	-	-	EtOHcl1	-	Galcl1	-	YPDnc2	YPDnc3	YPDnc4	EtOHnc1	EtOHnc2	Galnc1	Galnc2
9		YLR413W	YPDcl2	YPDcl3	EtOHcl1	EtOHcl2	-	YPDnc1	-	-	-	EtOHnc1	EtOHnc2	Galnc1	Galnc2
9	IRC5	YFR038W	-	YPDcl3	-	EtOHcl2	Galcl1	YPDnc1	-	-	YPDnc4	EtOHnc1	EtOHnc2	Galnc1	Galnc2
9	NTE1	YML059C	-	-	EtOHcl1	EtOHcl2	Galcl1	YPDnc1	YPDnc2	YPDnc3	YPDnc4	-	EtOHnc2	-	Galnc2
9	RPS16B	YDL083C	YPDcl2	YPDcl3	-	-	Galcl1	-	YPDnc2	YPDnc3	YPDnc4	EtOHnc1	EtOHnc2	-	Galnc2
9	PDH1	YPR002W	YPDcl2	-	EtOHcl1	-	Galcl1	YPDnc1	YPDnc2	YPDnc3	YPDnc4	-	EtOHnc2	-	Galnc2
9	IRC13	YOR235W	-	-	EtOHcl1	-	Galcl1	YPDnc1	YPDnc2	YPDnc3	-	EtOHnc1	EtOHnc2	Galnc1	Galnc2
9		YMR315W-	YPDcl2	YPDcl3	EtOHcl1	EtOHcl2	Galcl1	-	-	-	YPDnc4	EtOHnc1	-	Galnc1	Galnc2
9	CYT1	YOR065W	-	-	EtOHcl1	-	-	YPDnc1	YPDnc2	YPDnc3	YPDnc4	EtOHnc1	EtOHnc2	Galnc1	Galnc2
9		YBL029W	YPDcl2	-	EtOHcl1	-	Galcl1	-	YPDnc2	YPDnc3	YPDnc4	-	EtOHnc2	Galnc1	Galnc2
9	RME1	YGR044C	-	-	EtOHcl1	-	Galcl1	YPDnc1	YPDnc2	YPDnc3	YPDnc4	EtOHnc1	-	Galnc1	Galnc2
9	ZIP1	YDR285W	-	YPDcl3	EtOHcl1	-	Galcl1	YPDnc1	YPDnc2	YPDnc3	YPDnc4	-	EtOHnc2	Galnc1	-
9	POX1	YGL205W	YPDcl2	YPDcl3	EtOHcl1	EtOHcl2	Galcl1	-	YPDnc2	YPDnc3	YPDnc4	-	-	-	Galnc2
9	EXG1	YLR300W	-	YPDcl3	EtOHcl1	-	-	YPDnc1	YPDnc2	YPDnc3	YPDnc4	EtOHnc1	-	Galnc1	Galnc2
9	RAD28	YDR030C	YPDcl2	YPDcl3	EtOHcl1	EtOHcl2	Galcl1	-	YPDnc2	YPDnc3	YPDnc4	-	EtOHnc2	-	-
9	RPS23B	YPR132W	-	-	EtOHcl1	EtOHcl2	Galcl1	YPDnc1	-	YPDnc3	-	EtOHnc1	EtOHnc2	Galnc1	Galnc2
9		YIL100C-A	-	YPDcl3	EtOHcl1	-	-	-	YPDnc2	YPDnc3	YPDnc4	EtOHnc1	EtOHnc2	Galnc1	Galnc2
9	YMC1	YPR058W	YPDcl2	-	EtOHcl1	EtOHcl2	Galcl1	YPDnc1	YPDnc2	-	YPDnc4	-	EtOHnc2	Galnc1	-
9	HOT13	YKL084W	YPDcl2	YPDcl3	EtOHcl1	EtOHcl2	Galcl1	-	YPDnc2	-	YPDnc4	EtOHnc1	-	-	Galnc2
9		YHR063W-	YPDcl2	-	EtOHcl1	EtOHcl2	Galcl1	YPDnc1	-	-	-	EtOHnc1	EtOHnc2	Galnc1	Galnc2
9	ERG27	YLR100W	-	-	EtOHcl1	EtOHcl2	-	YPDnc1	YPDnc2	YPDnc3	YPDnc4	-	EtOHnc2	Galnc1	Galnc2
9	CMP2	YML057W	YPDcl2	YPDcl3	EtOHcl1	EtOHcl2	Galcl1	-	YPDnc2	-	YPDnc4	EtOHnc1	EtOHnc2	-	-
9	DBF4	YDR052C	YPDcl2	YPDcl3	-	EtOHcl2	Galcl1	-	YPDnc2	YPDnc3	YPDnc4	-	EtOHnc2	Galnc1	-
9	CYT2	YKL087C	-	YPDcl3	EtOHcl1	EtOHcl2	Galcl1	-	YPDnc2	YPDnc3	YPDnc4	-	EtOHnc2	-	Galnc2
9		YML101C-	-	-	EtOHcl1	EtOHcl2	Galcl1	YPDnc1	YPDnc2	YPDnc3	-	-	EtOHnc2	Galnc1	Galnc2
9	IMD4	YML056C	YPDcl2	YPDcl3	EtOHcl1	EtOHcl2	Galcl1	-	YPDnc2	YPDnc3	YPDnc4	-	EtOHnc2	-	-
9	HIP1	YGR191W	YPDcl2	-	EtOHcl1	EtOHcl2	Galcl1	YPDnc1	-	-	-	EtOHnc1	EtOHnc2	Galnc1	Galnc2
9	PTP2	YOR208W	YPDcl2	YPDcl3	EtOHcl1	EtOHcl2	Galcl1	YPDnc1	-	-	-	EtOHnc1	-	Galnc1	Galnc2
9	NOP9	YJL010C	-	-	EtOHcl1	-	-	YPDnc1	YPDnc2	YPDnc3	YPDnc4	EtOHnc1	EtOHnc2	Galnc1	Galnc2
9	PLC1	YPL268W	YPDcl2	YPDcl3	-	-	Galcl1	-	YPDnc2	-	YPDnc4	EtOHnc1	EtOHnc2	Galnc1	Galnc2
9		YLL053C	-	-	EtOHcl1	-	Galcl1	-	YPDnc2	YPDnc3	YPDnc4	EtOHnc1	EtOHnc2	Galnc1	Galnc2
8	RTT10	YPL183C	-	-	-	EtOHcl2	Galcl1	-	YPDnc2	YPDnc3	YPDnc4	EtOHnc1	-	Galnc1	Galnc2
8		YNL228W	-	-	-	-	-	YPDnc1	YPDnc2	YPDnc3	YPDnc4	EtOHnc1	EtOHnc2	Galnc1	Galnc2

8		YLR434C	YPDcl2	-	-	-	Galcl1	YPDnc1	-	-	YPDnc4	EtOHnc1	EtOHnc2	Galnc1	Galnc2
8	VBA4	YDR119W	YPDcl2	YPDcl3	-	-	Galcl1	-	YPDnc2	YPDnc3	YPDnc4	EtOHnc1	-	-	Galnc2
8	CEM1	YER061C	YPDcl2	YPDcl3	-	EtOHcl2	Galcl1	YPDnc1	-	-	-	EtOHnc1	EtOHnc2	-	Galnc2
8	HNT1	YDL125C	-	YPDcl3	-	EtOHcl2	Galcl1	YPDnc1	-	YPDnc3	-	EtOHnc1	-	Galnc1	Galnc2
8		YOL166C	-	YPDcl3	EtOHcl1	-	Galcl1	YPDnc1	YPDnc2	-	YPDnc4	-	-	Galnc1	Galnc2
8	TGL5	YOR081C	-	-	-	EtOHcl2	-	-	YPDnc2	YPDnc3	YPDnc4	EtOHnc1	EtOHnc2	Galnc1	Galnc2
8		YGL079W	YPDcl2	-	-	EtOHcl2	Galcl1	YPDnc1	-	-	-	EtOHnc1	EtOHnc2	Galnc1	Galnc2
8	VPS73	YGL104C	YPDcl2	YPDcl3	EtOHcl1	EtOHcl2	Galcl1	-	YPDnc2	-	YPDnc4	-	EtOHnc2	-	-
8		YLR264C-A	YPDcl2	YPDcl3	-	-	Galcl1	-	YPDnc2	YPDnc3	YPDnc4	EtOHnc1	-	-	Galnc2
8		YLR445W	YPDcl2	-	-	-	Galcl1	YPDnc1	YPDnc2	YPDnc3	YPDnc4	EtOHnc1	-	Galnc1	-
8	MSH5	YDL154W	-	YPDcl3	-	-	Galcl1	YPDnc1	-	YPDnc3	YPDnc4	EtOHnc1	-	Galnc1	Galnc2
8	CTA1	YDR256C	YPDcl2	-	EtOHcl1	-	Galcl1	-	-	YPDnc3	-	EtOHnc1	EtOHnc2	Galnc1	Galnc2
8	PTI1	YGR156W	-	YPDcl3	EtOHcl1	-	Galcl1	YPDnc1	YPDnc2	YPDnc3	-	EtOHnc1	-	Galnc1	-
8	TOP3	YLR234W	-	YPDcl3	-	-	Galcl1	-	YPDnc2	YPDnc3	YPDnc4	EtOHnc1	EtOHnc2	-	Galnc2
8		YOR053W	YPDcl2	-	-	-	-	YPDnc1	YPDnc2	YPDnc3	-	EtOHnc1	EtOHnc2	Galnc1	Galnc2
8		YER084W-/	YPDcl2	-	EtOHcl1	-	Galcl1	-	YPDnc2	YPDnc3	YPDnc4	-	EtOHnc2	Galnc1	-
8		YMR141W-	YPDcl2	-	EtOHcl1	-	Galcl1	YPDnc1	YPDnc2	YPDnc3	-	-	EtOHnc2	Galnc1	-
8		YOR192C-C	-	-	-	-	Galcl1	YPDnc1	YPDnc2	-	YPDnc4	EtOHnc1	EtOHnc2	Galnc1	Galnc2
8	POP2	YNR052C	-	YPDcl3	EtOHcl1	EtOHcl2	Galcl1	YPDnc1	-	-	YPDnc4	EtOHnc1	-	-	Galnc2
8	CDC24	YAL041W	-	-	EtOHcl1	-	-	YPDnc1	YPDnc2	YPDnc3	-	EtOHnc1	EtOHnc2	Galnc1	Galnc2
8	INO1	YJL153C	-	-	EtOHcl1	-	Galcl1	YPDnc1	YPDnc2	YPDnc3	-	EtOHnc1	EtOHnc2	Galnc1	-
8	CDC10	YCR002C	YPDcl2	-	-	-	Galcl1	YPDnc1	-	-	YPDnc4	EtOHnc1	EtOHnc2	Galnc1	Galnc2
8	SPS19	YNL202W	-	-	EtOHcl1	-	Galcl1	YPDnc1	YPDnc2	-	-	EtOHnc1	EtOHnc2	Galnc1	Galnc2
8	YIG1	YPL201C	YPDcl2	-	EtOHcl1	EtOHcl2	Galcl1	-	YPDnc2	YPDnc3	-	EtOHnc1	-	Galnc1	-
8	ARB1	YER036C	-	YPDcl3	-	-	Galcl1	YPDnc1	-	-	YPDnc4	EtOHnc1	EtOHnc2	Galnc1	Galnc2
8	UTP5	YDR398W	-	YPDcl3	EtOHcl1	EtOHcl2	-	YPDnc1	-	-	-	EtOHnc1	EtOHnc2	Galnc1	Galnc2
8	ORM1	YGR038W	YPDcl2	YPDcl3	EtOHcl1	-	Galcl1	-	YPDnc2	-	YPDnc4	EtOHnc1	-	-	Galnc2
8		YNL017C	-	-	EtOHcl1	EtOHcl2	Galcl1	YPDnc1	-	-	-	EtOHnc1	EtOHnc2	Galnc1	Galnc2
8		YER078W-/	-	YPDcl3	-	EtOHcl2	-	YPDnc1	YPDnc2	-	YPDnc4	EtOHnc1	-	Galnc1	Galnc2
8	SEF1	YBL066C	YPDcl2	YPDcl3	EtOHcl1	EtOHcl2	Galcl1	-	YPDnc2	-	YPDnc4	-	-	Galnc1	-
8	VTS1	YOR359W	YPDcl2	-	EtOHcl1	EtOHcl2	Galcl1	YPDnc1	-	-	-	EtOHnc1	EtOHnc2	Galnc1	-
8	TIM13	YGR181W	-	-	EtOHcl1	-	-	YPDnc1	YPDnc2	YPDnc3	YPDnc4	EtOHnc1	-	Galnc1	Galnc2
8		YMR194C-/	-	YPDcl3	EtOHcl1	-	Galcl1	YPDnc1	-	YPDnc3	-	EtOHnc1	EtOHnc2	-	Galnc2
8		YDR065W	-	YPDcl3	EtOHcl1	-	Galcl1	-	YPDnc2	YPDnc3	YPDnc4	-	EtOHnc2	Galnc1	-

8	POP3	YNL282W	-	YPDcl3	EtOHcl1	EtOHcl2	Galcl1	YPDnc1	-	-	-	EtOHnc1	-	Galnc1	Galnc2
8		YLR278C	-	-	EtOHcl1	-	-	YPDnc1	YPDnc2	YPDnc3	-	EtOHnc1	EtOHnc2	Galnc1	Galnc2
8	BMH2	YDR099W	YPDcl2	YPDcl3	EtOHcl1	-	Galcl1	-	-	-	YPDnc4	EtOHnc1	-	Galnc1	Galnc2
8	CAF120	YNL278W	YPDcl2	-	EtOHcl1	EtOHcl2	Galcl1	YPDnc1	-	-	YPDnc4	-	EtOHnc2	Galnc1	-
8	YFH1	YDL120W	-	-	EtOHcl1	EtOHcl2	Galcl1	YPDnc1	YPDnc2	-	-	-	EtOHnc2	Galnc1	Galnc2
8	ILV2	YMR108W	-	-	-	-	Galcl1	YPDnc1	YPDnc2	YPDnc3	-	EtOHnc1	EtOHnc2	Galnc1	Galnc2
8	HHT2	YNL031C	-	-	EtOHcl1	EtOHcl2	-	YPDnc1	YPDnc2	YPDnc3	YPDnc4	-	-	Galnc1	Galnc2
8	HXT17	YNR072W	-	-	EtOHcl1	-	Galcl1	YPDnc1	-	YPDnc3	-	EtOHnc1	EtOHnc2	Galnc1	Galnc2
8	MSN4	YKL062W	-	YPDcl3	EtOHcl1	EtOHcl2	Galcl1	-	-	-	-	EtOHnc1	EtOHnc2	Galnc1	Galnc2
8		YLR111W	-	-	EtOHcl1	EtOHcl2	Galcl1	YPDnc1	-	YPDnc3	-	EtOHnc1	-	Galnc1	Galnc2
8	CCT4	YDL143W	YPDcl2	-	-	EtOHcl2	Galcl1	-	YPDnc2	YPDnc3	YPDnc4	-	-	Galnc1	Galnc2
8	KTI11	YBL071W-A	-	YPDcl3	-	-	-	YPDnc1	YPDnc2	YPDnc3	-	EtOHnc1	EtOHnc2	Galnc1	Galnc2
8	SEO1	YAL067C	YPDcl2	-	EtOHcl1	-	Galcl1	YPDnc1	-	-	-	EtOHnc1	EtOHnc2	Galnc1	Galnc2
8	MRPL27	YBR282W	-	YPDcl3	EtOHcl1	EtOHcl2	Galcl1	-	-	-	-	EtOHnc1	EtOHnc2	Galnc1	Galnc2
8	YCP4	YCR004C	YPDcl2	YPDcl3	-	-	Galcl1	YPDnc1	-	-	YPDnc4	EtOHnc1	EtOHnc2	Galnc1	-
8	YPT11	YNL304W	YPDcl2	YPDcl3	EtOHcl1	EtOHcl2	Galcl1	YPDnc1	YPDnc2	-	YPDnc4	-	-	-	-
8	SCS7	YMR272C	YPDcl2	-	EtOHcl1	-	Galcl1	-	YPDnc2	YPDnc3	YPDnc4	-	-	Galnc1	Galnc2
8		YKR041W	-	-	EtOHcl1	-	-	YPDnc1	YPDnc2	YPDnc3	YPDnc4	EtOHnc1	EtOHnc2	-	Galnc2
8		YGR146C	-	-	EtOHcl1	-	-	YPDnc1	YPDnc2	-	YPDnc4	EtOHnc1	EtOHnc2	Galnc1	Galnc2
8	GIP3	YPL137C	YPDcl2	-	EtOHcl1	-	Galcl1	YPDnc1	YPDnc2	-	YPDnc4	EtOHnc1	-	-	Galnc2
8	SYS1	YJL004C	-	-	-	-	Galcl1	YPDnc1	YPDnc2	-	YPDnc4	EtOHnc1	EtOHnc2	Galnc1	Galnc2
8	AIM19	YIL087C	YPDcl2	YPDcl3	EtOHcl1	-	Galcl1	-	YPDnc2	YPDnc3	YPDnc4	-	-	-	Galnc2
8	ENB1	YOL158C	-	-	EtOHcl1	-	-	-	YPDnc2	YPDnc3	YPDnc4	EtOHnc1	EtOHnc2	Galnc1	Galnc2
8		YOR318C	-	YPDcl3	EtOHcl1	EtOHcl2	Galcl1	-	-	YPDnc3	YPDnc4	-	EtOHnc2	-	Galnc2
8	ERG6	YML008C	-	YPDcl3	-	EtOHcl2	-	YPDnc1	-	YPDnc3	-	EtOHnc1	EtOHnc2	Galnc1	Galnc2
8		YBR196C-A	-	-	EtOHcl1	-	Galcl1	YPDnc1	YPDnc2	YPDnc3	-	EtOHnc1	EtOHnc2	-	Galnc2
8	SPE3	YPR069C	YPDcl2	YPDcl3	EtOHcl1	EtOHcl2	Galcl1	YPDnc1	-	YPDnc3	-	-	-	Galnc1	-
8	BFA1	YJR053W	-	-	-	-	Galcl1	YPDnc1	YPDnc2	YPDnc3	YPDnc4	EtOHnc1	-	Galnc1	Galnc2
8		YGR126W	-	-	-	-	Galcl1	YPDnc1	YPDnc2	YPDnc3	YPDnc4	EtOHnc1	-	Galnc1	Galnc2
8	SSZ1	YHR064C	YPDcl2	-	EtOHcl1	EtOHcl2	Galcl1	YPDnc1	-	-	-	-	EtOHnc2	Galnc1	Galnc2
8	MED1	YPR070W	-	YPDcl3	EtOHcl1	EtOHcl2	-	YPDnc1	YPDnc2	-	-	EtOHnc1	-	Galnc1	Galnc2
8	RSF1	YMR030W	YPDcl2	YPDcl3	-	EtOHcl2	Galcl1	YPDnc1	-	-	-	EtOHnc1	-	Galnc1	Galnc2
8		YHR078W	YPDcl2	-	-	EtOHcl2	Galcl1	YPDnc1	YPDnc2	-	-	EtOHnc1	-	Galnc1	Galnc2
8	RRT12	YCR045C	-	-	EtOHcl1	-	-	YPDnc1	YPDnc2	YPDnc3	-	EtOHnc1	EtOHnc2	Galnc1	Galnc2

8	ATO3	YDR384C	-	YPDcl3	-	-	Galcl1	YPDnc1	-	-	YPDnc4	EtOHnc1	EtOHnc2	Galnc1	Galnc2
8	FYV1	YDR024W	-	-	EtOHcl1	-	-	-	YPDnc2	YPDnc3	YPDnc4	EtOHnc1	EtOHnc2	Galnc1	Galnc2
8		YFL040W	-	-	EtOHcl1	-	-	YPDnc1	-	YPDnc3	YPDnc4	EtOHnc1	EtOHnc2	Galnc1	Galnc2
8	HAP4	YKL109W	-	-	EtOHcl1	-	Galcl1	YPDnc1	YPDnc2	YPDnc3	YPDnc4	-	-	Galnc1	Galnc2
8	BNA5	YLR231C	YPDcl2	-	EtOHcl1	-	Galcl1	YPDnc1	YPDnc2	-	YPDnc4	EtOHnc1	-	-	Galnc2
8	GRX3	YDR098C	YPDcl2	YPDcl3	EtOHcl1	EtOHcl2	Galcl1	-	-	-	-	EtOHnc1	-	Galnc1	Galnc2
8	QDR1	YIL120W	YPDcl2	-	EtOHcl1	-	-	YPDnc1	YPDnc2	YPDnc3	-	EtOHnc1	-	Galnc1	Galnc2
8	WTM1	YOR230W	-	YPDcl3	-	-	Galcl1	-	-	YPDnc3	YPDnc4	EtOHnc1	EtOHnc2	Galnc1	Galnc2
8	KIN28	YDL108W	YPDcl2	YPDcl3	EtOHcl1	EtOHcl2	Galcl1	-	YPDnc2	YPDnc3	YPDnc4	-	-	-	-
8		YPR159C-A	YPDcl2	-	EtOHcl1	-	-	YPDnc1	YPDnc2	YPDnc3	-	EtOHnc1	-	Galnc1	Galnc2
8	CLU1	YMR012W	YPDcl2	YPDcl3	EtOHcl1	-	Galcl1	-	-	-	YPDnc4	EtOHnc1	EtOHnc2	-	Galnc2
8		YBL071C	-	YPDcl3	-	EtOHcl2	-	YPDnc1	YPDnc2	-	YPDnc4	EtOHnc1	-	Galnc1	Galnc2
8		YHR086W-[illegible]	-	-	-	-	Galcl1	YPDnc1	-	YPDnc3	YPDnc4	EtOHnc1	EtOHnc2	Galnc1	Galnc2
8	TDA2	YER071C	-	-	-	EtOHcl2	-	YPDnc1	-	YPDnc3	YPDnc4	EtOHnc1	EtOHnc2	Galnc1	Galnc2
8	HRQ1	YDR291W	YPDcl2	YPDcl3	-	-	Galcl1	-	YPDnc2	YPDnc3	-	-	EtOHnc2	Galnc1	Galnc2
8		YOR203W	YPDcl2	YPDcl3	-	EtOHcl2	Galcl1	-	-	-	-	EtOHnc1	EtOHnc2	Galnc1	Galnc2
8	MET7	YOR241W	YPDcl2	-	EtOHcl1	-	Galcl1	-	YPDnc2	YPDnc3	-	-	EtOHnc2	Galnc1	Galnc2
8	TAH11	YJR046W	YPDcl2	YPDcl3	EtOHcl1	-	-	-	-	YPDnc3	YPDnc4	-	EtOHnc2	Galnc1	Galnc2
8		YPL136W	YPDcl2	-	EtOHcl1	-	Galcl1	YPDnc1	YPDnc2	-	YPDnc4	EtOHnc1	-	-	Galnc2
8		YGL036W	-	YPDcl3	EtOHcl1	EtOHcl2	-	YPDnc1	-	YPDnc3	-	EtOHnc1	-	Galnc1	Galnc2
8	AAD16	YFL057C	YPDcl2	YPDcl3	EtOHcl1	EtOHcl2	Galcl1	YPDnc1	-	-	-	EtOHnc1	-	Galnc1	-
8	ARA1	YBR149W	-	YPDcl3	EtOHcl1	EtOHcl2	Galcl1	YPDnc1	-	-	-	EtOHnc1	EtOHnc2	Galnc1	-
8	MPP6	YNR024W	YPDcl2	YPDcl3	EtOHcl1	EtOHcl2	Galcl1	-	-	YPDnc3	-	EtOHnc1	-	-	Galnc2
8	SPG1	YGR236C	YPDcl2	-	-	-	-	YPDnc1	YPDnc2	YPDnc3	-	EtOHnc1	EtOHnc2	Galnc1	Galnc2
8	SIW14	YNL032W	-	-	EtOHcl1	EtOHcl2	-	YPDnc1	YPDnc2	YPDnc3	YPDnc4	-	-	Galnc1	Galnc2
8	RPL30	YGL030W	YPDcl2	-	EtOHcl1	-	Galcl1	-	YPDnc2	YPDnc3	YPDnc4	-	EtOHnc2	-	Galnc2
8		YAL016C-B	-	YPDcl3	EtOHcl1	EtOHcl2	Galcl1	YPDnc1	-	YPDnc3	-	EtOHnc1	-	-	Galnc2
8	PHM8	YER037W	-	YPDcl3	-	EtOHcl2	Galcl1	-	YPDnc2	-	-	EtOHnc1	EtOHnc2	Galnc1	Galnc2
8		YML034C-[illegible]	-	-	EtOHcl1	-	-	YPDnc1	YPDnc2	YPDnc3	-	EtOHnc1	EtOHnc2	Galnc1	Galnc2
8	YOS9	YDR057W	YPDcl2	-	-	-	Galcl1	YPDnc1	-	YPDnc3	YPDnc4	EtOHnc1	EtOHnc2	Galnc1	-
8	RAV2	YDR202C	YPDcl2	-	-	EtOHcl2	Galcl1	-	YPDnc2	-	YPDnc4	EtOHnc1	EtOHnc2	-	Galnc2
8	BUD7	YOR299W	-	-	-	EtOHcl2	-	YPDnc1	-	YPDnc3	YPDnc4	EtOHnc1	EtOHnc2	Galnc1	Galnc2
8		YML002W	YPDcl2	-	-	EtOHcl2	Galcl1	YPDnc1	YPDnc2	-	YPDnc4	EtOHnc1	-	Galnc1	-
8		YPL150W	YPDcl2	YPDcl3	EtOHcl1	EtOHcl2	Galcl1	-	-	-	-	EtOHnc1	-	Galnc1	Galnc2

8		YPL199C	YPDcl2	YPDcl3	-	-	Galcl1	YPDnc1	-	-	YPDnc4	EtOHnc1	EtOHnc2	Galnc1	-
8	PDB1	YBR221C	-	-	EtOHcl1	-	Galcl1	YPDnc1	-	-	YPDnc4	EtOHnc1	EtOHnc2	Galnc1	Galnc2
8	LSM3	YLR438C-A	-	YPDcl3	-	-	Galcl1	YPDnc1	YPDnc2	-	-	EtOHnc1	EtOHnc2	Galnc1	Galnc2
8	PCL8	YPL219W	-	YPDcl3	EtOHcl1	EtOHcl2	Galcl1	YPDnc1	-	-	YPDnc4	EtOHnc1	-	Galnc1	-
8	CYB2	YML054C	YPDcl2	YPDcl3	EtOHcl1	-	Galcl1	YPDnc1	-	YPDnc3	-	-	-	Galnc1	Galnc2
8		YBL108W	YPDcl2	-	-	-	Galcl1	YPDnc1	YPDnc2	YPDnc3	-	EtOHnc1	-	Galnc1	Galnc2
8		YMR124W	YPDcl2	YPDcl3	EtOHcl1	-	Galcl1	-	-	-	YPDnc4	EtOHnc1	EtOHnc2	Galnc1	-
8		YNR063W	YPDcl2	-	EtOHcl1	EtOHcl2	-	YPDnc1	-	-	YPDnc4	-	EtOHnc2	Galnc1	Galnc2
8	YEL1	YBL060W	YPDcl2	YPDcl3	EtOHcl1	EtOHcl2	Galcl1	YPDnc1	-	-	YPDnc4	-	-	Galnc1	-
8	RFC5	YBR087W	-	YPDcl3	-	-	-	YPDnc1	YPDnc2	YPDnc3	YPDnc4	EtOHnc1	-	Galnc1	Galnc2
8	FMP23	YBR047W	YPDcl2	YPDcl3	EtOHcl1	EtOHcl2	Galcl1	YPDnc1	-	-	YPDnc4	-	-	Galnc1	-
8	API2	YDR525W	-	-	EtOHcl1	-	Galcl1	YPDnc1	YPDnc2	YPDnc3	-	EtOHnc1	-	Galnc1	Galnc2
8	OSH6	YKR003W	YPDcl2	YPDcl3	-	EtOHcl2	Galcl1	-	YPDnc2	-	YPDnc4	-	EtOHnc2	Galnc1	-
8	YRB1	YDR002W	YPDcl2	YPDcl3	EtOHcl1	-	Galcl1	YPDnc1	-	-	-	EtOHnc1	EtOHnc2	-	Galnc2
8	YPT7	YML001W	YPDcl2	-	-	EtOHcl2	Galcl1	YPDnc1	-	-	YPDnc4	EtOHnc1	EtOHnc2	Galnc1	-
8	ERP2	YAL007C	YPDcl2	YPDcl3	EtOHcl1	EtOHcl2	Galcl1	-	-	-	-	EtOHnc1	EtOHnc2	Galnc1	-
8	SPS18	YNL204C	-	-	EtOHcl1	-	-	YPDnc1	YPDnc2	YPDnc3	YPDnc4	EtOHnc1	EtOHnc2	Galnc1	-
8	CIS1	YDR022C	-	-	-	EtOHcl2	Galcl1	-	YPDnc2	-	YPDnc4	EtOHnc1	EtOHnc2	Galnc1	Galnc2
8	APD1	YBR151W	-	-	EtOHcl1	-	Galcl1	YPDnc1	YPDnc2	-	-	EtOHnc1	EtOHnc2	Galnc1	Galnc2
8		YOL163W	YPDcl2	YPDcl3	-	-	-	YPDnc1	-	YPDnc3	YPDnc4	EtOHnc1	-	Galnc1	Galnc2
8	TED1	YIL039W	YPDcl2	-	-	-	Galcl1	YPDnc1	-	-	YPDnc4	EtOHnc1	EtOHnc2	Galnc1	Galnc2
8	COR1	YBL045C	YPDcl2	-	EtOHcl1	-	-	YPDnc1	-	YPDnc3	YPDnc4	-	EtOHnc2	Galnc1	Galnc2
8	TVP18	YMR071C	YPDcl2	YPDcl3	EtOHcl1	-	Galcl1	-	YPDnc2	YPDnc3	YPDnc4	-	EtOHnc2	-	-
8	SIF2	YBR103W	YPDcl2	-	EtOHcl1	-	-	YPDnc1	YPDnc2	YPDnc3	YPDnc4	EtOHnc1	-	-	Galnc2
8	NYV1	YLR093C	-	YPDcl3	EtOHcl1	-	-	YPDnc1	YPDnc2	-	-	EtOHnc1	EtOHnc2	Galnc1	Galnc2
8	KCC4	YCL024W	-	-	EtOHcl1	-	-	YPDnc1	YPDnc2	YPDnc3	-	EtOHnc1	EtOHnc2	Galnc1	Galnc2
8	HAC1	YFL031W	YPDcl2	YPDcl3	-	EtOHcl2	-	YPDnc1	-	YPDnc3	YPDnc4	-	-	Galnc1	Galnc2
8		YDR094W	YPDcl2	YPDcl3	EtOHcl1	EtOHcl2	-	-	YPDnc2	YPDnc3	-	-	EtOHnc2	-	Galnc2
8	PDC5	YLR134W	-	-	-	-	Galcl1	YPDnc1	-	YPDnc3	YPDnc4	EtOHnc1	EtOHnc2	Galnc1	Galnc2
8		YGL069C	YPDcl2	YPDcl3	-	EtOHcl2	Galcl1	-	YPDnc2	YPDnc3	-	-	EtOHnc2	-	Galnc2
8		YGR107W	-	-	-	EtOHcl2	Galcl1	YPDnc1	YPDnc2	-	YPDnc4	EtOHnc1	-	Galnc1	Galnc2
8		YJL086C	YPDcl2	-	EtOHcl1	-	Galcl1	YPDnc1	YPDnc2	YPDnc3	YPDnc4	-	EtOHnc2	-	-
8	YHI9	YHR029C	YPDcl2	-	EtOHcl1	-	Galcl1	YPDnc1	-	YPDnc3	YPDnc4	EtOHnc1	-	-	Galnc2
8	RPL5	YPL131W	YPDcl2	-	EtOHcl1	-	Galcl1	-	YPDnc2	YPDnc3	YPDnc4	-	EtOHnc2	Galnc1	-

8		YPL073C	-	-	EtOHcl1	-	Galcl1	YPDnc1	-	YPDnc3	YPDnc4	EtOHnc1	EtOHnc2	Galnc1	-
8	APE3	YBR286W	YPDcl2	YPDcl3	EtOHcl1	-	Galcl1	-	-	-	YPDnc4	EtOHnc1	-	Galnc1	Galnc2
8		YPL238C	-	-	-	-	-	YPDnc1	YPDnc2	YPDnc3	YPDnc4	EtOHnc1	EtOHnc2	Galnc1	Galnc2
8	SSB1	YDL229W	-	-	-	-	-	YPDnc1	YPDnc2	YPDnc3	YPDnc4	EtOHnc1	EtOHnc2	Galnc1	Galnc2
8	PAU3	YCR104W	-	YPDcl3	EtOHcl1	-	-	YPDnc1	-	YPDnc3	YPDnc4	EtOHnc1	-	Galnc1	Galnc2
8	DGR2	YKL121W	-	-	EtOHcl1	-	-	YPDnc1	YPDnc2	YPDnc3	-	EtOHnc1	EtOHnc2	Galnc1	Galnc2
7		YPR098C	YPDcl2	YPDcl3	-	EtOHcl2	Galcl1	-	-	-	YPDnc4	-	EtOHnc2	Galnc1	-
7		YGR190C	-	YPDcl3	EtOHcl1	-	Galcl1	YPDnc1	-	-	-	EtOHnc1	-	Galnc1	Galnc2
7	TOM1	YDR457W	-	-	-	-	-	YPDnc1	YPDnc2	YPDnc3	-	EtOHnc1	EtOHnc2	Galnc1	Galnc2
7	MUP1	YGR055W	YPDcl2	-	-	-	Galcl1	YPDnc1	YPDnc2	-	-	EtOHnc1	-	Galnc1	Galnc2
7	POS5	YPL188W	YPDcl2	-	-	-	Galcl1	YPDnc1	YPDnc2	YPDnc3	YPDnc4	-	-	Galnc1	-
7	MCA1	YOR197W	-	-	EtOHcl1	-	-	YPDnc1	-	-	YPDnc4	EtOHnc1	EtOHnc2	Galnc1	Galnc2
7	RPS1B	YML063W	-	-	-	-	Galcl1	YPDnc1	YPDnc2	YPDnc3	YPDnc4	EtOHnc1	EtOHnc2	-	-
7		YKR040C	-	-	EtOHcl1	-	-	YPDnc1	-	YPDnc3	-	EtOHnc1	EtOHnc2	Galnc1	Galnc2
7	MRP8	YKL142W	YPDcl2	-	EtOHcl1	-	Galcl1	YPDnc1	-	-	-	EtOHnc1	-	Galnc1	Galnc2
7		YMR244W	-	YPDcl3	-	EtOHcl2	-	YPDnc1	-	-	YPDnc4	EtOHnc1	-	Galnc1	Galnc2
7		YDR246W-/	-	-	EtOHcl1	-	-	YPDnc1	YPDnc2	YPDnc3	YPDnc4	-	-	Galnc1	Galnc2
7	SPP41	YDR464W	YPDcl2	YPDcl3	EtOHcl1	EtOHcl2	Galcl1	-	-	YPDnc3	-	-	EtOHnc2	-	-
7	HTA1	YDR225W	-	-	EtOHcl1	-	-	YPDnc1	-	YPDnc3	YPDnc4	EtOHnc1	-	Galnc1	Galnc2
7	HMS1	YOR032C	-	-	EtOHcl1	-	-	YPDnc1	YPDnc2	YPDnc3	YPDnc4	-	-	Galnc1	Galnc2
7	KTR3	YBR205W	-	-	-	-	Galcl1	-	-	YPDnc3	YPDnc4	EtOHnc1	EtOHnc2	Galnc1	Galnc2
7		YGR069W	-	-	EtOHcl1	-	-	YPDnc1	YPDnc2	YPDnc3	-	EtOHnc1	-	Galnc1	Galnc2
7	ALD4	YOR374W	-	YPDcl3	-	EtOHcl2	-	YPDnc1	-	-	-	EtOHnc1	EtOHnc2	Galnc1	Galnc2
7		YLR345W	YPDcl2	-	EtOHcl1	-	Galcl1	-	-	-	YPDnc4	EtOHnc1	EtOHnc2	-	Galnc2
7		YOR072W-	YPDcl2	YPDcl3	-	-	Galcl1	YPDnc1	-	-	-	EtOHnc1	-	Galnc1	Galnc2
7	FCY2	YER056C	-	-	EtOHcl1	-	Galcl1	-	-	-	YPDnc4	EtOHnc1	EtOHnc2	Galnc1	Galnc2
7	RPS14B	YJL191W	-	YPDcl3	EtOHcl1	EtOHcl2	-	-	YPDnc2	YPDnc3	-	-	EtOHnc2	-	Galnc2
7	ARH1	YDR376W	-	-	EtOHcl1	-	Galcl1	YPDnc1	-	YPDnc3	-	EtOHnc1	-	Galnc1	Galnc2
7		YNR065C	YPDcl2	YPDcl3	-	EtOHcl2	Galcl1	YPDnc1	-	-	-	-	-	Galnc1	Galnc2
7	VHS3	YOR054C	YPDcl2	-	EtOHcl1	-	-	YPDnc1	YPDnc2	YPDnc3	YPDnc4	-	-	-	Galnc2
7		YLL065W	YPDcl2	YPDcl3	EtOHcl1	EtOHcl2	Galcl1	-	-	-	YPDnc4	-	EtOHnc2	-	-
7	RIB7	YBR153W	-	-	-	EtOHcl2	Galcl1	YPDnc1	-	YPDnc3	-	EtOHnc1	-	Galnc1	Galnc2
7	RPL15B	YMR121C	-	-	EtOHcl1	-	-	YPDnc1	YPDnc2	-	YPDnc4	EtOHnc1	-	Galnc1	Galnc2
7		YIL054W	-	-	EtOHcl1	EtOHcl2	Galcl1	YPDnc1	-	-	-	EtOHnc1	-	Galnc1	Galnc2

7	ARR3	YPR201W	YPDcl2	-	-	EtOHcl2	Galcl1	YPDnc1	-	-	YPDnc4	-	-	Galnc1	Galnc2
7	SLD2	YKL108W	-	-	EtOHcl1	-	Galcl1	YPDnc1	YPDnc2	-	-	EtOHnc1	-	Galnc1	Galnc2
7	DDR48	YMR173W	YPDcl2	YPDcl3	-	EtOHcl2	Galcl1	-	YPDnc2	YPDnc3	YPDnc4	-	-	-	-
7	DOT6	YER088C	-	-	EtOHcl1	EtOHcl2	-	YPDnc1	YPDnc2	YPDnc3	-	-	EtOHnc2	-	Galnc2
7	UTP6	YDR449C	YPDcl2	YPDcl3	EtOHcl1	EtOHcl2	Galcl1	-	YPDnc2	-	YPDnc4	-	-	-	-
7	RGP1	YDR137W	-	-	EtOHcl1	-	Galcl1	-	YPDnc2	YPDnc3	YPDnc4	-	-	Galnc1	Galnc2
7		YHR032W-	YPDcl2	-	EtOHcl1	-	-	YPDnc1	YPDnc2	YPDnc3	YPDnc4	EtOHnc1	-	-	-
7	RPB5	YBR154C	-	YPDcl3	-	EtOHcl2	Galcl1	-	-	-	-	EtOHnc1	EtOHnc2	Galnc1	Galnc2
7	BAR1	YIL015W	YPDcl2	YPDcl3	-	-	Galcl1	-	YPDnc2	-	YPDnc4	EtOHnc1	EtOHnc2	-	-
7	RPR2	YIR015W	-	YPDcl3	EtOHcl1	-	-	YPDnc1	YPDnc2	-	-	EtOHnc1	EtOHnc2	-	Galnc2
7	RPL20A	YMR242C	YPDcl2	-	EtOHcl1	EtOHcl2	-	-	-	YPDnc3	-	EtOHnc1	-	Galnc1	Galnc2
7	RPS24B	YIL069C	-	-	EtOHcl1	-	-	YPDnc1	YPDnc2	-	YPDnc4	-	EtOHnc2	Galnc1	Galnc2
7		YDR307W	-	-	EtOHcl1	EtOHcl2	Galcl1	-	-	-	YPDnc4	EtOHnc1	EtOHnc2	-	Galnc2
7	DPH2	YKL191W	-	-	EtOHcl1	EtOHcl2	Galcl1	-	-	-	YPDnc4	EtOHnc1	EtOHnc2	Galnc1	-
7		YPR123C	-	-	-	-	Galcl1	YPDnc1	YPDnc2	YPDnc3	-	EtOHnc1	-	Galnc1	Galnc2
7	SAH1	YER043C	YPDcl2	YPDcl3	EtOHcl1	EtOHcl2	Galcl1	-	-	-	-	-	-	Galnc1	Galnc2
7	PUP2	YGR253C	YPDcl2	-	EtOHcl1	EtOHcl2	Galcl1	YPDnc1	YPDnc2	YPDnc3	-	-	-	-	-
7	ROM2	YLR371W	-	-	EtOHcl1	-	Galcl1	-	-	YPDnc3	YPDnc4	EtOHnc1	-	Galnc1	Galnc2
7	VPS13	YLL040C	YPDcl2	-	EtOHcl1	EtOHcl2	Galcl1	YPDnc1	-	-	YPDnc4	-	-	-	Galnc2
7	SNF7	YLR025W	-	-	-	EtOHcl2	-	YPDnc1	YPDnc2	YPDnc3	-	EtOHnc1	-	Galnc1	Galnc2
7	EDC2	YER035W	-	YPDcl3	-	-	Galcl1	YPDnc1	-	-	-	EtOHnc1	EtOHnc2	Galnc1	Galnc2
7	MF(ALPHA	YPL187W	YPDcl2	-	-	-	-	YPDnc1	YPDnc2	YPDnc3	YPDnc4	-	-	Galnc1	Galnc2
7	TDA6	YPR157W	YPDcl2	YPDcl3	-	EtOHcl2	Galcl1	-	-	YPDnc3	-	-	EtOHnc2	Galnc1	-
7		YIL055C	-	YPDcl3	EtOHcl1	-	Galcl1	-	-	YPDnc3	YPDnc4	-	EtOHnc2	-	Galnc2
7		YMR320W	YPDcl2	-	-	-	-	YPDnc1	YPDnc2	YPDnc3	-	EtOHnc1	EtOHnc2	-	Galnc2
7	IDH1	YNL037C	YPDcl2	-	EtOHcl1	-	Galcl1	YPDnc1	YPDnc2	-	-	-	EtOHnc2	Galnc1	-
7	DSE3	YOR264W	YPDcl2	-	EtOHcl1	-	-	-	YPDnc2	YPDnc3	YPDnc4	EtOHnc1	-	Galnc1	-
7	MAM33	YIL070C	-	-	EtOHcl1	-	-	YPDnc1	YPDnc2	-	YPDnc4	-	EtOHnc2	Galnc1	Galnc2
7	CST9	YLR394W	YPDcl2	YPDcl3	EtOHcl1	-	Galcl1	-	YPDnc2	YPDnc3	YPDnc4	-	-	-	-
7	LYS20	YDL182W	YPDcl2	YPDcl3	EtOHcl1	-	Galcl1	YPDnc1	YPDnc2	-	-	-	-	-	Galnc2
7	RGT2	YDL138W	YPDcl2	-	EtOHcl1	EtOHcl2	Galcl1	-	-	YPDnc3	-	EtOHnc1	-	-	Galnc2
7	FRE8	YLR047C	-	YPDcl3	EtOHcl1	EtOHcl2	-	-	YPDnc2	YPDnc3	-	-	EtOHnc2	-	Galnc2
7	RPS31	YLR167W	-	YPDcl3	EtOHcl1	-	-	YPDnc1	YPDnc2	YPDnc3	YPDnc4	-	-	Galnc1	-
7	TDA11	YHR159W	-	YPDcl3	EtOHcl1	-	Galcl1	-	YPDnc2	-	YPDnc4	-	-	Galnc1	Galnc2

7		YNL226W	YPDcl2	-	EtOHcl1	EtOHcl2	Galcl1	-	-	-	-	-	EtOHnc2	Galnc1	Galnc2
7	RPL36B	YPL249C-A	-	-	-	-	Galcl1	-	YPDnc2	YPDnc3	-	EtOHnc1	EtOHnc2	Galnc1	Galnc2
7	GLG2	YJL137C	YPDcl2	YPDcl3	EtOHcl1	-	Galcl1	-	YPDnc2	YPDnc3	YPDnc4	-	-	-	-
7		YDL185C-A	-	-	-	EtOHcl2	Galcl1	YPDnc1	-	YPDnc3	-	EtOHnc1	EtOHnc2	Galnc1	-
7	RPS2	YGL123W	-	-	EtOHcl1	-	-	YPDnc1	YPDnc2	YPDnc3	-	EtOHnc1	EtOHnc2	Galnc1	-
7	STB1	YNL309W	-	YPDcl3	EtOHcl1	-	Galcl1	YPDnc1	-	-	-	EtOHnc1	EtOHnc2	Galnc1	-
7		YDR102C	-	YPDcl3	-	EtOHcl2	Galcl1	YPDnc1	-	-	-	EtOHnc1	-	Galnc1	Galnc2
7	DGR1	YNL130C-A	YPDcl2	YPDcl3	EtOHcl1	EtOHcl2	Galcl1	-	-	-	YPDnc4	-	EtOHnc2	-	-
7	PAU8	YAL068C	-	YPDcl3	-	-	Galcl1	YPDnc1	-	YPDnc3	-	EtOHnc1	-	Galnc1	Galnc2
7	HOP1	YIL072W	-	-	-	-	Galcl1	YPDnc1	YPDnc2	YPDnc3	YPDnc4	-	-	Galnc1	Galnc2
7	MYO2	YOR326W	YPDcl2	YPDcl3	EtOHcl1	-	Galcl1	-	-	-	-	EtOHnc1	EtOHnc2	-	Galnc2
7		YLR282C	-	-	-	-	-	YPDnc1	YPDnc2	YPDnc3	YPDnc4	EtOHnc1	EtOHnc2	-	Galnc2
7	FOB1	YDR110W	-	-	-	-	-	YPDnc1	YPDnc2	YPDnc3	-	EtOHnc1	EtOHnc2	Galnc1	Galnc2
7		YIR014W	-	YPDcl3	EtOHcl1	-	-	YPDnc1	-	-	-	EtOHnc1	EtOHnc2	Galnc1	Galnc2
7	SEC15	YGL233W	-	-	-	EtOHcl2	Galcl1	-	YPDnc2	-	-	EtOHnc1	EtOHnc2	Galnc1	Galnc2
7	RKM3	YBR030W	YPDcl2	YPDcl3	EtOHcl1	-	Galcl1	YPDnc1	YPDnc2	YPDnc3	-	-	-	-	-
7	NPT1	YOR209C	YPDcl2	YPDcl3	EtOHcl1	EtOHcl2	Galcl1	-	-	-	-	EtOHnc1	-	Galnc1	-
7	SSP1	YHR184W	YPDcl2	-	-	-	Galcl1	YPDnc1	YPDnc2	YPDnc3	-	EtOHnc1	-	-	Galnc2
7	FYV12	YOR183W	-	-	-	-	Galcl1	YPDnc1	-	YPDnc3	-	EtOHnc1	EtOHnc2	Galnc1	Galnc2
7	RTR2	YDR066C	YPDcl2	YPDcl3	EtOHcl1	EtOHcl2	Galcl1	-	-	-	YPDnc4	-	-	-	Galnc2
7		YBR089W	-	-	-	EtOHcl2	Galcl1	YPDnc1	YPDnc2	YPDnc3	-	EtOHnc1	-	Galnc1	-
7	RIM9	YMR063W	YPDcl2	-	-	-	Galcl1	YPDnc1	YPDnc2	-	-	EtOHnc1	EtOHnc2	Galnc1	-
7	NOP15	YNL110C	YPDcl2	-	EtOHcl1	-	Galcl1	YPDnc1	YPDnc2	YPDnc3	YPDnc4	-	-	-	-
7	SPE1	YKL184W	YPDcl2	-	EtOHcl1	EtOHcl2	Galcl1	-	-	-	YPDnc4	EtOHnc1	EtOHnc2	-	-
7	SUL2	YLR092W	-	YPDcl3	-	-	Galcl1	YPDnc1	-	-	YPDnc4	EtOHnc1	-	Galnc1	Galnc2
7	ERV1	YGR029W	YPDcl2	YPDcl3	-	EtOHcl2	Galcl1	YPDnc1	-	-	-	EtOHnc1	-	-	Galnc2
7		YDR274C	YPDcl2	-	EtOHcl1	-	-	YPDnc1	-	YPDnc3	-	EtOHnc1	-	Galnc1	Galnc2
7	BNI4	YNL233W	-	-	EtOHcl1	-	Galcl1	YPDnc1	-	-	YPDnc4	EtOHnc1	-	Galnc1	Galnc2
7		YAR030C	YPDcl2	YPDcl3	EtOHcl1	EtOHcl2	-	-	YPDnc2	YPDnc3	-	-	-	Galnc1	-
7		YLL020C	-	-	-	-	-	YPDnc1	YPDnc2	YPDnc3	YPDnc4	EtOHnc1	-	Galnc1	Galnc2
7	LYS21	YDL131W	YPDcl2	YPDcl3	EtOHcl1	-	Galcl1	YPDnc1	-	YPDnc3	YPDnc4	-	-	-	-
7	FTR1	YER145C	-	YPDcl3	EtOHcl1	-	-	YPDnc1	YPDnc2	-	-	EtOHnc1	-	Galnc1	Galnc2
7		YDR491C	-	-	-	-	Galcl1	YPDnc1	YPDnc2	YPDnc3	-	EtOHnc1	-	Galnc1	Galnc2
7	VTA1	YLR181C	-	-	-	-	-	YPDnc1	YPDnc2	YPDnc3	-	EtOHnc1	EtOHnc2	Galnc1	Galnc2

7	GSC2	YGR032W	YPDcl2	-	EtOHcl1	EtOHcl2	Galcl1	-	-	-	-	EtOHnc1	EtOHnc2	Galnc1	-
7	PGM2	YMR105C	YPDcl2	YPDcl3	EtOHcl1	-	Galcl1	-	-	-	YPDnc4	-	-	Galnc1	Galnc2
7		YCR085W	-	-	-	EtOHcl2	Galcl1	YPDnc1	-	-	YPDnc4	EtOHnc1	-	Galnc1	Galnc2
7		YMR031C	YPDcl2	YPDcl3	EtOHcl1	EtOHcl2	Galcl1	YPDnc1	-	-	-	-	-	-	Galnc2
7		YGL188C	YPDcl2	YPDcl3	EtOHcl1	-	-	-	-	-	-	EtOHnc1	EtOHnc2	Galnc1	Galnc2
7		YJL216C	-	-	EtOHcl1	-	Galcl1	-	-	YPDnc3	YPDnc4	EtOHnc1	EtOHnc2	Galnc1	-
7	HEM4	YOR278W	-	YPDcl3	-	-	-	YPDnc1	-	-	YPDnc4	EtOHnc1	EtOHnc2	Galnc1	Galnc2
7	CPR3	YML078W	-	YPDcl3	-	EtOHcl2	Galcl1	-	-	-	YPDnc4	-	EtOHnc2	Galnc1	Galnc2
7	KRE33	YNL132W	-	YPDcl3	-	EtOHcl2	Galcl1	YPDnc1	-	-	-	EtOHnc1	EtOHnc2	Galnc1	-
7	PHO89	YBR296C	-	YPDcl3	EtOHcl1	EtOHcl2	-	-	-	-	YPDnc4	EtOHnc1	-	Galnc1	Galnc2
7	UBC11	YOR339C	-	-	-	EtOHcl2	Galcl1	-	YPDnc2	-	-	EtOHnc1	EtOHnc2	Galnc1	Galnc2
7	SNQ2	YDR011W	YPDcl2	YPDcl3	EtOHcl1	EtOHcl2	Galcl1	-	YPDnc2	-	YPDnc4	-	-	-	-
7		YPL108W	-	-	-	-	Galcl1	-	-	YPDnc3	YPDnc4	EtOHnc1	EtOHnc2	Galnc1	Galnc2
7		YHR182C-A	-	-	EtOHcl1	-	-	YPDnc1	YPDnc2	-	YPDnc4	EtOHnc1	EtOHnc2	-	Galnc2
7		YFR039C	-	YPDcl3	-	EtOHcl2	Galcl1	YPDnc1	-	-	-	EtOHnc1	-	Galnc1	Galnc2
7		YPR011C	YPDcl2	-	-	-	Galcl1	-	YPDnc2	-	YPDnc4	-	EtOHnc2	Galnc1	Galnc2
7	SPS100	YHR139C	-	-	EtOHcl1	-	Galcl1	YPDnc1	YPDnc2	-	-	EtOHnc1	-	Galnc1	Galnc2
7	AIM7	YDR063W	YPDcl2	-	EtOHcl1	-	-	YPDnc1	YPDnc2	YPDnc3	-	EtOHnc1	-	Galnc1	-
7		YLR419W	YPDcl2	-	-	EtOHcl2	Galcl1	YPDnc1	-	-	-	EtOHnc1	EtOHnc2	Galnc1	-
7		YNR071C	-	-	EtOHcl1	-	Galcl1	YPDnc1	-	YPDnc3	-	EtOHnc1	-	Galnc1	Galnc2
7	HSP12	YFL014W	-	YPDcl3	-	EtOHcl2	Galcl1	YPDnc1	-	-	-	EtOHnc1	-	Galnc1	Galnc2
7	FSH1	YHR049W	YPDcl2	-	EtOHcl1	EtOHcl2	Galcl1	YPDnc1	-	YPDnc3	YPDnc4	-	-	-	-
7	ABZ2	YMR289W	-	YPDcl3	-	-	-	YPDnc1	-	-	YPDnc4	EtOHnc1	EtOHnc2	Galnc1	Galnc2
7	SLM5	YCR024C	YPDcl2	-	EtOHcl1	EtOHcl2	Galcl1	-	YPDnc2	-	YPDnc4	-	EtOHnc2	-	-
7		YMR254C	YPDcl2	-	EtOHcl1	-	Galcl1	-	-	-	YPDnc4	EtOHnc1	-	Galnc1	Galnc2
7	NAT4	YMR069W	-	-	EtOHcl1	EtOHcl2	-	YPDnc1	-	YPDnc3	-	EtOHnc1	EtOHnc2	-	Galnc2
7		YLR030W	YPDcl2	YPDcl3	-	EtOHcl2	Galcl1	-	YPDnc2	-	YPDnc4	-	EtOHnc2	-	-
7	TFC1	YBR123C	-	-	-	-	Galcl1	YPDnc1	YPDnc2	YPDnc3	YPDnc4	-	-	Galnc1	Galnc2
7		YOR331C	-	YPDcl3	-	EtOHcl2	Galcl1	YPDnc1	-	-	-	EtOHnc1	-	Galnc1	Galnc2
6		YBL095W	YPDcl2	-	EtOHcl1	-	Galcl1	-	-	YPDnc3	-	EtOHnc1	EtOHnc2	-	-
6	NUP84	YDL116W	-	-	EtOHcl1	-	-	-	-	-	YPDnc4	EtOHnc1	EtOHnc2	Galnc1	Galnc2
6		YGR266W	YPDcl2	YPDcl3	-	EtOHcl2	Galcl1	-	-	-	-	EtOHnc1	-	-	Galnc2
6	MCD1	YDL003W	-	-	-	EtOHcl2	-	-	-	YPDnc3	YPDnc4	-	EtOHnc2	Galnc1	Galnc2
6	MUQ1	YGR007W	YPDcl2	-	EtOHcl1	-	Galcl1	YPDnc1	-	YPDnc3	YPDnc4	-	-	-	-

6	KNS1	YLL019C	-	-	EtOHcl1	-	-	YPDnc1	-	YPDnc3	-	EtOHnc1	-	Galnc1	Galnc2
6	DPB11	YJL090C	YPDcl2	-	EtOHcl1	-	Galcl1	-	-	-	YPDnc4	EtOHnc1	-	Galnc1	-
6	YAP1802	YGR241C	-	YPDcl3	EtOHcl1	-	Galcl1	YPDnc1	-	YPDnc3	-	-	-	Galnc1	-
6	AXL2	YIL140W	YPDcl2	YPDcl3	EtOHcl1	EtOHcl2	Galcl1	-	-	-	-	-	-	Galnc1	-
6	XBP1	YIL101C	-	-	EtOHcl1	EtOHcl2	-	-	-	YPDnc3	-	EtOHnc1	EtOHnc2	Galnc1	-
6	MPT5	YGL178W	YPDcl2	YPDcl3	-	-	Galcl1	-	-	YPDnc3	-	-	EtOHnc2	Galnc1	-
6	MSH6	YDR097C	YPDcl2	-	EtOHcl1	EtOHcl2	Galcl1	-	-	-	-	-	-	Galnc1	Galnc2
6	ICP55	YER078C	-	YPDcl3	EtOHcl1	EtOHcl2	-	-	-	-	YPDnc4	EtOHnc1	-	-	Galnc2
6	KRE6	YPR159W	YPDcl2	YPDcl3	EtOHcl1	-	-	-	-	YPDnc3	-	-	-	Galnc1	Galnc2
6	SAS10	YDL153C	-	-	-	EtOHcl2	Galcl1	-	YPDnc2	YPDnc3	YPDnc4	-	EtOHnc2	-	-
6		YGL052W	YPDcl2	-	-	EtOHcl2	-	-	YPDnc2	YPDnc3	-	-	-	Galnc1	Galnc2
6		YDR249C	YPDcl2	YPDcl3	-	EtOHcl2	Galcl1	-	-	-	YPDnc4	-	EtOHnc2	-	-
6		YBL053W	YPDcl2	YPDcl3	-	EtOHcl2	Galcl1	-	-	-	-	EtOHnc1	-	-	Galnc2
6	SUC2	YIL162W	-	-	-	EtOHcl2	Galcl1	-	YPDnc2	-	-	EtOHnc1	EtOHnc2	Galnc1	-
6	RCK2	YLR248W	-	-	EtOHcl1	-	-	YPDnc1	-	-	-	EtOHnc1	EtOHnc2	Galnc1	Galnc2
6	RAX1	YOR301W	-	-	EtOHcl1	-	Galcl1	-	YPDnc2	YPDnc3	YPDnc4	-	-	-	Galnc2
6		YPL044C	YPDcl2	-	-	-	Galcl1	-	-	-	-	EtOHnc1	EtOHnc2	Galnc1	Galnc2
6	DFG16	YOR030W	-	-	-	-	-	YPDnc1	YPDnc2	YPDnc3	-	EtOHnc1	-	Galnc1	Galnc2
6		YCR049C	-	YPDcl3	-	-	-	YPDnc1	-	-	-	EtOHnc1	EtOHnc2	Galnc1	Galnc2
6		YCL041C	-	YPDcl3	EtOHcl1	-	-	YPDnc1	-	-	-	EtOHnc1	-	Galnc1	Galnc2
6	PNT1	YOR266W	-	-	-	-	Galcl1	YPDnc1	-	-	YPDnc4	EtOHnc1	-	Galnc1	Galnc2
6		YDR048C	-	-	-	EtOHcl2	Galcl1	-	YPDnc2	-	-	EtOHnc1	EtOHnc2	-	Galnc2
6	NAN1	YPL126W	-	YPDcl3	-	EtOHcl2	Galcl1	-	-	-	-	EtOHnc1	EtOHnc2	Galnc1	-
6	YHK8	YHR048W	-	-	EtOHcl1	EtOHcl2	Galcl1	YPDnc1	-	-	YPDnc4	EtOHnc1	-	-	-
6	LSB1	YGR136W	-	-	-	-	-	-	YPDnc2	YPDnc3	YPDnc4	-	EtOHnc2	Galnc1	Galnc2
6	MFA2	YNL145W	-	-	-	-	-	YPDnc1	YPDnc2	YPDnc3	-	-	EtOHnc2	Galnc1	Galnc2
6		YMR194C-[illegible]	-	YPDcl3	EtOHcl1	-	Galcl1	YPDnc1	-	-	-	EtOHnc1	-	-	Galnc2
6	SLM4	YBR077C	-	-	-	-	-	YPDnc1	-	YPDnc3	-	EtOHnc1	EtOHnc2	Galnc1	Galnc2
6	PHM7	YOL084W	YPDcl2	YPDcl3	-	-	Galcl1	-	YPDnc2	-	YPDnc4	-	-	Galnc1	-
6		YPL191C	-	YPDcl3	EtOHcl1	EtOHcl2	-	-	-	-	-	EtOHnc1	EtOHnc2	Galnc1	-
6	ALK2	YBL009W	YPDcl2	YPDcl3	-	EtOHcl2	Galcl1	-	YPDnc2	-	YPDnc4	-	-	-	-
6	TRS23	YDR246W	-	YPDcl3	EtOHcl1	-	-	YPDnc1	-	-	-	EtOHnc1	-	Galnc1	Galnc2
6	AIM10	YER087W	YPDcl2	-	-	EtOHcl2	-	-	-	YPDnc3	-	EtOHnc1	EtOHnc2	Galnc1	-
6		YAL059C-A	-	YPDcl3	EtOHcl1	-	-	-	YPDnc2	YPDnc3	YPDnc4	-	-	-	Galnc2

6		YJL027C	-	-	EtOHcl1	-	Galcl1	YPDnc1	-	YPDnc3	-	-	-	Galnc1	Galnc2
6	LTV1	YKL143W	YPDcl2	-	EtOHcl1	-	Galcl1	YPDnc1	-	-	-	-	-	Galnc1	Galnc2
6	RSB1	YOR049C	-	YPDcl3	EtOHcl1	-	-	-	-	-	-	EtOHnc1	EtOHnc2	Galnc1	Galnc2
6	SPS1	YDR523C	-	-	EtOHcl1	-	-	-	YPDnc2	YPDnc3	-	EtOHnc1	EtOHnc2	Galnc1	-
6	SEC17	YBL050W	-	YPDcl3	EtOHcl1	-	Galcl1	-	-	-	YPDnc4	EtOHnc1	-	Galnc1	-
6	AHA1	YDR214W	-	-	EtOHcl1	-	-	YPDnc1	YPDnc2	YPDnc3	-	-	EtOHnc2	-	Galnc2
6	SAY1	YGR263C	-	-	EtOHcl1	EtOHcl2	Galcl1	-	-	-	-	EtOHnc1	EtOHnc2	Galnc1	-
6		YNL146W	-	-	-	-	-	YPDnc1	YPDnc2	YPDnc3	-	-	EtOHnc2	Galnc1	Galnc2
6	HTB1	YDR224C	-	-	EtOHcl1	-	-	YPDnc1	-	-	YPDnc4	EtOHnc1	-	Galnc1	Galnc2
6		YPR108W-/	-	-	EtOHcl1	-	Galcl1	YPDnc1	-	YPDnc3	-	EtOHnc1	-	Galnc1	-
6	SHR5	YOL110W	-	-	EtOHcl1	-	Galcl1	-	YPDnc2	-	YPDnc4	-	-	Galnc1	Galnc2
6	UBC9	YDL064W	-	-	-	EtOHcl2	Galcl1	YPDnc1	-	-	-	EtOHnc1	EtOHnc2	-	Galnc2
6	CSH1	YBR161W	-	-	EtOHcl1	-	-	YPDnc1	-	-	-	EtOHnc1	EtOHnc2	Galnc1	Galnc2
6		YLR162W	YPDcl2	YPDcl3	EtOHcl1	EtOHcl2	Galcl1	-	-	-	-	-	-	Galnc1	-
6	NSA1	YGL111W	YPDcl2	-	-	EtOHcl2	-	YPDnc1	-	-	-	EtOHnc1	-	Galnc1	Galnc2
6	RPS10B	YMR230W	YPDcl2	-	EtOHcl1	-	Galcl1	-	YPDnc2	YPDnc3	-	-	-	-	Galnc2
6	RGI1	YER067W	YPDcl2	-	-	-	-	-	YPDnc2	YPDnc3	YPDnc4	EtOHnc1	-	Galnc1	-
6	FAA1	YOR317W	-	YPDcl3	EtOHcl1	EtOHcl2	Galcl1	-	-	YPDnc3	YPDnc4	-	-	-	-
6	UFD4	YKL010C	-	-	-	-	Galcl1	-	YPDnc2	-	YPDnc4	EtOHnc1	-	Galnc1	Galnc2
6	DCG1	YIR030C	YPDcl2	YPDcl3	-	EtOHcl2	-	-	YPDnc2	YPDnc3	YPDnc4	-	-	-	-
6	CSL4	YNL232W	-	-	EtOHcl1	EtOHcl2	-	YPDnc1	YPDnc2	YPDnc3	-	-	-	-	Galnc2
6		YLR414C	-	YPDcl3	EtOHcl1	-	-	-	-	-	-	EtOHnc1	EtOHnc2	Galnc1	Galnc2
6		YLR149C-A	-	-	-	-	Galcl1	YPDnc1	-	YPDnc3	-	EtOHnc1	-	Galnc1	Galnc2
6	MKK1	YOR231W	-	-	-	-	Galcl1	-	YPDnc2	-	YPDnc4	EtOHnc1	EtOHnc2	Galnc1	-
6	ADE3	YGR204W	YPDcl2	-	EtOHcl1	EtOHcl2	Galcl1	YPDnc1	-	-	-	-	-	Galnc1	-
6	CCT8	YJL008C	YPDcl2	-	EtOHcl1	-	Galcl1	YPDnc1	YPDnc2	-	YPDnc4	-	-	-	-
6	BSC2	YDR275W	YPDcl2	YPDcl3	-	-	-	YPDnc1	-	-	YPDnc4	-	-	Galnc1	Galnc2
6	VPS3	YDR495C	YPDcl2	YPDcl3	EtOHcl1	EtOHcl2	Galcl1	-	-	-	-	EtOHnc1	-	-	-
6	PTP1	YDL230W	-	-	-	-	Galcl1	-	YPDnc2	YPDnc3	YPDnc4	-	EtOHnc2	-	Galnc2
6		YOR050C	-	YPDcl3	-	-	-	-	-	YPDnc3	-	EtOHnc1	EtOHnc2	Galnc1	Galnc2
6	YPT6	YLR262C	-	-	-	EtOHcl2	Galcl1	YPDnc1	-	YPDnc3	-	EtOHnc1	-	-	Galnc2
6		YLR235C	-	-	EtOHcl1	-	-	YPDnc1	-	-	YPDnc4	-	EtOHnc2	Galnc1	Galnc2
6	RRP5	YMR229C	YPDcl2	-	EtOHcl1	EtOHcl2	Galcl1	-	-	-	-	-	EtOHnc2	-	Galnc2
6		YGR050C	-	YPDcl3	EtOHcl1	-	Galcl1	-	-	YPDnc3	-	-	EtOHnc2	-	Galnc2

6	ISU1	YPL135W	-	-	-	-	-	YPDnc1	YPDnc2	YPDnc3	YPDnc4	-	EtOHnc2	Galnc1	-
6	SCM4	YGR049W	-	-	EtOHcl1	-	Galcl1	-	-	YPDnc3	-	-	EtOHnc2	Galnc1	Galnc2
6	NAB6	YML117W	YPDcl2	YPDcl3	EtOHcl1	EtOHcl2	Galcl1	YPDnc1	-	-	-	-	-	-	-
6		YHR137C-A	-	-	-	-	Galcl1	YPDnc1	YPDnc2	YPDnc3	-	EtOHnc1	-	-	Galnc2
6		YOR314W	-	-	EtOHcl1	-	-	-	-	YPDnc3	YPDnc4	EtOHnc1	EtOHnc2	Galnc1	-
6	STP2	YHR006W	-	-	EtOHcl1	EtOHcl2	-	YPDnc1	-	YPDnc3	-	-	-	Galnc1	Galnc2
6	BRR2	YER172C	YPDcl2	-	EtOHcl1	EtOHcl2	Galcl1	-	YPDnc2	-	YPDnc4	-	-	-	-
6	ADE17	YMR120C	-	-	EtOHcl1	-	-	YPDnc1	-	-	-	EtOHnc1	EtOHnc2	Galnc1	Galnc2
6	BUD30	YDL151C	YPDcl2	YPDcl3	EtOHcl1	-	Galcl1	YPDnc1	-	-	-	EtOHnc1	-	-	-
6	URA7	YBL039C	YPDcl2	-	EtOHcl1	EtOHcl2	-	-	-	YPDnc3	YPDnc4	-	EtOHnc2	-	-
6	BDF1	YLR399C	-	YPDcl3	EtOHcl1	-	-	-	YPDnc2	YPDnc3	-	-	EtOHnc2	-	Galnc2
6	RPL28	YGL103W	YPDcl2	-	EtOHcl1	-	Galcl1	-	YPDnc2	YPDnc3	-	-	-	Galnc1	-
6	CBC2	YPL178W	-	-	EtOHcl1	-	-	YPDnc1	-	YPDnc3	-	EtOHnc1	-	Galnc1	Galnc2
6	CAR2	YLR438W	-	-	-	-	Galcl1	YPDnc1	-	-	-	EtOHnc1	EtOHnc2	Galnc1	Galnc2
6		YBR090C	-	-	EtOHcl1	-	-	YPDnc1	YPDnc2	-	-	EtOHnc1	EtOHnc2	Galnc1	-
6	RPF1	YHR088W	-	-	-	-	-	YPDnc1	YPDnc2	YPDnc3	-	EtOHnc1	EtOHnc2	-	Galnc2
6	HXT6	YDR343C	-	-	EtOHcl1	-	-	YPDnc1	YPDnc2	YPDnc3	-	-	-	Galnc1	Galnc2
6		YNL097C-B	-	-	EtOHcl1	-	-	YPDnc1	YPDnc2	-	YPDnc4	-	EtOHnc2	-	Galnc2
6	PRM9	YAR031W	-	-	EtOHcl1	EtOHcl2	-	-	YPDnc2	YPDnc3	-	-	EtOHnc2	-	Galnc2
6		YAL004W	-	-	-	EtOHcl2	Galcl1	-	YPDnc2	YPDnc3	-	EtOHnc1	-	-	Galnc2
6		YHL015W-A	-	-	-	-	-	YPDnc1	-	YPDnc3	-	EtOHnc1	EtOHnc2	Galnc1	Galnc2
6	POL30	YBR088C	-	-	-	EtOHcl2	Galcl1	YPDnc1	YPDnc2	-	-	EtOHnc1	-	Galnc1	-
6	CFT1	YDR301W	-	-	-	-	Galcl1	YPDnc1	YPDnc2	YPDnc3	YPDnc4	-	-	-	Galnc2
6	SDH4	YDR178W	YPDcl2	YPDcl3	EtOHcl1	-	Galcl1	-	-	YPDnc3	YPDnc4	-	-	-	-
6	MOT2	YER068W	YPDcl2	-	-	-	Galcl1	-	YPDnc2	YPDnc3	YPDnc4	-	EtOHnc2	-	-
6		YOR121C	-	-	EtOHcl1	-	Galcl1	YPDnc1	-	-	-	EtOHnc1	-	Galnc1	Galnc2
6	HER2	YMR293C	-	-	-	-	Galcl1	YPDnc1	-	YPDnc3	-	EtOHnc1	-	Galnc1	Galnc2
6	RPS9B	YBR189W	YPDcl2	YPDcl3	EtOHcl1	-	Galcl1	-	-	-	YPDnc4	-	EtOHnc2	-	-
6	GTO3	YMR251W	-	YPDcl3	EtOHcl1	-	-	-	-	-	-	EtOHnc1	EtOHnc2	Galnc1	Galnc2
6	SPR28	YDR218C	-	YPDcl3	-	EtOHcl2	Galcl1	-	-	-	YPDnc4	EtOHnc1	-	Galnc1	-
6	RPB4	YJL140W	YPDcl2	YPDcl3	-	-	Galcl1	YPDnc1	-	-	-	-	-	Galnc1	Galnc2
6	FZF1	YGL254W	-	-	EtOHcl1	-	-	YPDnc1	-	-	-	EtOHnc1	EtOHnc2	Galnc1	Galnc2
6		YGR073C	-	-	-	-	-	YPDnc1	YPDnc2	YPDnc3	-	EtOHnc1	EtOHnc2	-	Galnc2
6		YDR131C	YPDcl2	-	EtOHcl1	-	Galcl1	-	-	-	YPDnc4	-	EtOHnc2	-	Galnc2

5	MRPL24	YMR193W	YPDcl2	-	EtOHcl1	EtOHcl2	Galcl1	-	-	-	YPDnc4	-	-	-	-
5	GCN4	YEL009C	-	-	EtOHcl1	-	-	YPDnc1	-	YPDnc3	-	-	EtOHnc2	-	Galnc2
5	PET130	YJL023C	-	-	-	EtOHcl2	Galcl1	-	YPDnc2	YPDnc3	YPDnc4	-	-	-	-
5		YGR035C	YPDcl2	-	EtOHcl1	-	-	-	-	YPDnc3	-	EtOHnc1	-	-	Galnc2
5	NEO1	YIL048W	-	-	-	EtOHcl2	-	-	YPDnc2	YPDnc3	-	EtOHnc1	-	Galnc1	-
5	MEP1	YGR121C	-	-	-	EtOHcl2	Galcl1	-	-	-	-	EtOHnc1	EtOHnc2	-	Galnc2
5	RGT1	YKL038W	YPDcl2	YPDcl3	EtOHcl1	-	-	YPDnc1	-	-	-	-	-	Galnc1	-
5	RPL26B	YGR034W	YPDcl2	-	-	-	-	YPDnc1	-	-	-	EtOHnc1	-	Galnc1	Galnc2
5	SOL1	YNR034W	-	YPDcl3	-	-	Galcl1	-	YPDnc2	-	-	EtOHnc1	-	Galnc1	-
5		YJL028W	-	-	EtOHcl1	-	Galcl1	YPDnc1	-	YPDnc3	-	-	-	Galnc1	-
5	SPL2	YHR136C	-	-	-	-	-	-	YPDnc2	-	-	EtOHnc1	EtOHnc2	Galnc1	Galnc2
5		YLR456W	YPDcl2	-	-	EtOHcl2	Galcl1	-	-	-	-	EtOHnc1	-	Galnc1	-
5	HMRA1	YCR097W	YPDcl2	-	EtOHcl1	EtOHcl2	Galcl1	-	-	-	-	-	EtOHnc2	-	-
5	PMA2	YPL036W	-	YPDcl3	EtOHcl1	EtOHcl2	Galcl1	-	-	-	YPDnc4	-	-	-	-
5	GFD1	YMR255W	YPDcl2	-	EtOHcl1	-	Galcl1	-	-	-	-	EtOHnc1	-	-	Galnc2
5	ECM38	YLR299W	-	YPDcl3	EtOHcl1	-	Galcl1	YPDnc1	-	-	-	-	-	Galnc1	-
5	YPS5	YGL259W	YPDcl2	YPDcl3	EtOHcl1	-	Galcl1	-	-	-	-	EtOHnc1	-	-	-
5	NAB3	YPL190C	-	-	-	-	-	YPDnc1	-	YPDnc3	-	EtOHnc1	-	Galnc1	Galnc2
5	IRC14	YOR135C	-	-	-	-	-	YPDnc1	-	-	YPDnc4	EtOHnc1	-	Galnc1	Galnc2
5	ERG20	YJL167W	YPDcl2	YPDcl3	EtOHcl1	-	-	-	-	-	-	EtOHnc1	-	Galnc1	-
5	TSL1	YML100W	-	-	-	-	-	YPDnc1	YPDnc2	YPDnc3	-	EtOHnc1	-	-	Galnc2
5		YDR182W-/	-	YPDcl3	-	-	Galcl1	-	-	-	-	EtOHnc1	-	Galnc1	Galnc2
5	CCZ1	YBR131W	-	-	-	-	-	YPDnc1	YPDnc2	-	-	-	EtOHnc2	Galnc1	Galnc2
5	MUC1	YIR019C	-	-	EtOHcl1	-	-	YPDnc1	-	-	-	EtOHnc1	-	Galnc1	Galnc2
5		YOR338W	-	-	EtOHcl1	-	Galcl1	-	-	YPDnc3	YPDnc4	-	-	-	Galnc2
5		YFL015W-A	YPDcl2	YPDcl3	EtOHcl1	-	Galcl1	YPDnc1	-	-	-	-	-	-	-
5	RAS1	YOR101W	-	-	EtOHcl1	-	-	-	YPDnc2	YPDnc3	YPDnc4	-	EtOHnc2	-	-
5	AIM26	YKL037W	YPDcl2	YPDcl3	-	EtOHcl2	Galcl1	-	-	-	YPDnc4	-	-	-	-
5		YNL146C-A	-	-	-	-	Galcl1	YPDnc1	-	YPDnc3	-	-	-	Galnc1	Galnc2
5	KRE29	YER038C	-	-	-	-	Galcl1	-	YPDnc2	-	-	EtOHnc1	-	Galnc1	Galnc2
5		YKL096C-B	-	-	EtOHcl1	-	-	YPDnc1	-	-	-	EtOHnc1	EtOHnc2	-	Galnc2
5	PHR1	YOR386W	-	-	-	-	-	YPDnc1	-	YPDnc3	YPDnc4	EtOHnc1	EtOHnc2	-	-
5		YBR013C	YPDcl2	-	-	-	Galcl1	-	-	YPDnc3	-	EtOHnc1	-	-	Galnc2
5	RIM15	YFL033C	-	-	EtOHcl1	-	-	-	-	-	-	EtOHnc1	EtOHnc2	Galnc1	Galnc2

5	RKM4	YDR257C	-	-	-	-	Galcl1	YPDnc1	-	-	-	EtOHnc1	EtOHnc2	-	Galnc2
5	SLO1	YER180C-A	YPDcl2	YPDcl3	-	-	Galcl1	-	-	-	-	-	-	Galnc1	Galnc2
5	RNH202	YDR279W	-	YPDcl3	-	-	Galcl1	YPDnc1	-	-	-	EtOHnc1	-	Galnc1	-
5		YDL016C	-	-	-	-	-	-	YPDnc2	YPDnc3	-	EtOHnc1	-	Galnc1	Galnc2
5	PRE9	YGR135W	-	-	EtOHcl1	EtOHcl2	-	YPDnc1	-	-	-	-	EtOHnc2	-	Galnc2
5		YLR287C	-	-	-	-	Galcl1	-	-	YPDnc3	-	EtOHnc1	-	Galnc1	Galnc2
5	GRX6	YDL010W	-	-	-	-	-	YPDnc1	-	-	YPDnc4	EtOHnc1	EtOHnc2	Galnc1	-
5	KAP95	YLR347C	-	-	EtOHcl1	-	Galcl1	-	-	-	-	-	EtOHnc2	Galnc1	Galnc2
5	GIR2	YDR152W	-	-	EtOHcl1	-	-	YPDnc1	-	-	-	EtOHnc1	-	Galnc1	Galnc2
5		YDL073W	YPDcl2	-	-	-	Galcl1	YPDnc1	-	-	-	EtOHnc1	-	Galnc1	-
5		YJR085C	-	YPDcl3	-	EtOHcl2	Galcl1	-	-	-	-	EtOHnc1	-	Galnc1	-
5	RPL33B	YOR234C	-	YPDcl3	EtOHcl1	-	Galcl1	YPDnc1	-	-	-	-	-	Galnc1	-
5		YLR296W	-	-	-	-	Galcl1	YPDnc1	-	-	-	-	EtOHnc2	Galnc1	Galnc2
5		YPR136C	-	-	-	-	-	-	YPDnc2	-	-	EtOHnc1	EtOHnc2	Galnc1	Galnc2
5		YER079C-A	-	-	EtOHcl1	-	Galcl1	-	-	-	-	EtOHnc1	-	Galnc1	Galnc2
5		YML133W-	-	-	-	-	Galcl1	YPDnc1	YPDnc2	YPDnc3	-	EtOHnc1	-	-	-
5	UPC2	YDR213W	-	-	EtOHcl1	-	-	YPDnc1	-	-	-	EtOHnc1	-	Galnc1	Galnc2
5	LAT1	YNL071W	YPDcl2	-	-	-	Galcl1	-	-	-	-	-	EtOHnc2	Galnc1	Galnc2
5		YPR196W	YPDcl2	-	EtOHcl1	EtOHcl2	Galcl1	-	-	-	YPDnc4	-	-	-	-
5	CDC7	YDL017W	-	-	-	-	-	-	YPDnc2	YPDnc3	-	EtOHnc1	-	Galnc1	Galnc2
5	SYF1	YDR416W	-	-	EtOHcl1	-	Galcl1	-	YPDnc2	YPDnc3	-	-	-	-	Galnc2
5	RPL10	YLR075W	-	-	-	EtOHcl2	-	-	-	-	-	EtOHnc1	EtOHnc2	Galnc1	Galnc2
5	VPS36	YLR417W	-	-	-	-	Galcl1	YPDnc1	-	-	-	EtOHnc1	EtOHnc2	Galnc1	-
5		YHR140W	YPDcl2	-	EtOHcl1	EtOHcl2	-	-	-	-	YPDnc4	-	-	-	Galnc2
5	CMC1	YKL137W	YPDcl2	-	EtOHcl1	-	Galcl1	-	-	YPDnc3	-	-	-	-	Galnc2
5	SKS1	YPL026C	-	-	EtOHcl1	-	-	YPDnc1	-	-	YPDnc4	-	-	Galnc1	Galnc2
5	TEL1	YBL088C	YPDcl2	YPDcl3	EtOHcl1	-	Galcl1	-	-	-	-	-	-	-	Galnc2
5	AAC1	YMR056C	-	-	EtOHcl1	-	-	YPDnc1	-	YPDnc3	-	EtOHnc1	-	-	Galnc2
5	YAT1	YAR035W	-	-	-	-	Galcl1	YPDnc1	-	-	-	EtOHnc1	EtOHnc2	Galnc1	-
5	MMF1	YIL051C	-	-	EtOHcl1	-	Galcl1	-	-	-	-	EtOHnc1	-	Galnc1	Galnc2
5	RAS2	YNL098C	-	-	EtOHcl1	-	-	YPDnc1	YPDnc2	-	-	-	EtOHnc2	-	Galnc2
5	YRA1	YDR381W	-	-	EtOHcl1	EtOHcl2	Galcl1	-	-	-	-	EtOHnc1	-	Galnc1	-
5		YNL203C	YPDcl2	-	EtOHcl1	-	Galcl1	YPDnc1	-	-	-	-	EtOHnc2	-	-
5	SUS1	YBR111W-/	-	YPDcl3	-	-	-	-	-	-	-	EtOHnc1	EtOHnc2	Galnc1	Galnc2

5		YLL047W	-	YPDcl3	-	-	Galcl1	-	YPDnc2	YPDnc3	-	-	-	Galnc1	-
5		YKL027W	YPDcl2	-	EtOHcl1	-	-	-	YPDnc2	-	YPDnc4	-	-	Galnc1	-
5	RGM1	YMR182C	-	-	-	-	-	YPDnc1	YPDnc2	YPDnc3	-	EtOHnc1	-	Galnc1	-
5	MLP2	YIL149C	-	YPDcl3	EtOHcl1	-	Galcl1	-	-	-	-	-	EtOHnc2	-	Galnc2
5	CIT1	YNR001C	YPDcl2	YPDcl3	EtOHcl1	-	Galcl1	-	-	-	YPDnc4	-	-	-	-
5	RPS10A	YOR293W	-	-	-	-	-	YPDnc1	YPDnc2	YPDnc3	-	-	EtOHnc2	Galnc1	-
5	SHU2	YDR078C	YPDcl2	-	EtOHcl1	EtOHcl2	Galcl1	-	-	-	YPDnc4	-	-	-	-
5	BDF2	YDL070W	-	-	EtOHcl1	-	-	-	YPDnc2	YPDnc3	YPDnc4	-	EtOHnc2	-	-
5	ECM11	YDR446W	-	-	-	-	Galcl1	YPDnc1	-	-	-	EtOHnc1	-	Galnc1	Galnc2
5	ERG11	YHR007C	-	-	EtOHcl1	EtOHcl2	-	YPDnc1	-	-	-	-	-	Galnc1	Galnc2
5		YPL216W	-	-	EtOHcl1	EtOHcl2	Galcl1	-	-	-	YPDnc4	-	-	Galnc1	-
5		YDL160C-A	YPDcl2	-	EtOHcl1	-	Galcl1	-	-	-	YPDnc4	-	-	Galnc1	-
5	ADP1	YCR011C	-	-	EtOHcl1	-	-	YPDnc1	-	-	-	EtOHnc1	-	Galnc1	Galnc2
5	AKR2	YOR034C	-	-	-	-	Galcl1	-	-	-	YPDnc4	EtOHnc1	-	Galnc1	Galnc2
5	URA4	YLR420W	YPDcl2	-	-	-	Galcl1	-	-	-	YPDnc4	-	EtOHnc2	Galnc1	-
5	GLR1	YPL091W	-	-	EtOHcl1	EtOHcl2	Galcl1	YPDnc1	YPDnc2	-	-	-	-	-	-
5	FUI1	YBL042C	-	-	-	EtOHcl2	Galcl1	YPDnc1	-	-	-	EtOHnc1	-	Galnc1	-
5		YBR099C	YPDcl2	YPDcl3	EtOHcl1	-	Galcl1	-	-	-	-	EtOHnc1	-	-	-
5	RAD17	YOR368W	-	-	-	-	-	-	YPDnc2	-	YPDnc4	EtOHnc1	EtOHnc2	Galnc1	-
5	COX13	YGL191W	YPDcl2	-	EtOHcl1	-	-	YPDnc1	-	-	-	-	-	Galnc1	Galnc2
5		YDR132C	YPDcl2	YPDcl3	EtOHcl1	-	-	YPDnc1	-	-	YPDnc4	-	-	-	-
5	IVY1	YDR229W	-	-	-	-	-	YPDnc1	YPDnc2	YPDnc3	YPDnc4	-	-	Galnc1	-
5	SSA1	YAL005C	-	-	-	EtOHcl2	Galcl1	-	YPDnc2	YPDnc3	-	-	-	-	Galnc2
5	PGI1	YBR196C	-	-	EtOHcl1	-	-	-	-	YPDnc3	-	EtOHnc1	EtOHnc2	-	Galnc2
4	REG2	YBR050C	-	-	EtOHcl1	-	Galcl1	-	-	YPDnc3	-	EtOHnc1	-	-	-
4	MOH1	YBL049W	-	YPDcl3	-	-	-	YPDnc1	-	-	-	-	-	Galnc1	Galnc2
4	COX6	YHR051W	-	-	-	-	-	YPDnc1	-	-	-	EtOHnc1	EtOHnc2	-	Galnc2
4	DBP1	YPL119C	-	-	-	EtOHcl2	Galcl1	-	-	-	-	EtOHnc1	-	Galnc1	-
4		YHR032W	YPDcl2	-	EtOHcl1	-	-	YPDnc1	-	-	-	-	-	-	Galnc2
4		YNL181W	-	-	-	-	-	YPDnc1	-	-	-	EtOHnc1	-	Galnc1	Galnc2
4		YDR366C	-	-	EtOHcl1	-	-	-	-	-	-	EtOHnc1	EtOHnc2	Galnc1	-
4	CPR6	YLR216C	YPDcl2	-	EtOHcl1	-	Galcl1	-	-	-	YPDnc4	-	-	-	-
4	LYS2	YBR115C	-	-	EtOHcl1	-	Galcl1	-	YPDnc2	-	YPDnc4	-	-	-	-
4	ESF1	YDR365C	-	-	-	EtOHcl2	-	YPDnc1	-	-	-	-	-	Galnc1	Galnc2

4		YOL162W	YPDcl2	YPDcl3	-	-	Galcl1	-	-	-	YPDnc4	-	-	-	-
4	MDJ1	YFL016C	YPDcl2	-	-	-	Galcl1	-	-	-	-	-	-	Galnc1	Galnc2
4	MTC7	YEL033W	-	-	EtOHcl1	-	-	-	-	YPDnc3	-	EtOHnc1	-	-	Galnc2
4	BFR1	YOR198C	-	-	-	-	-	YPDnc1	-	-	-	EtOHnc1	EtOHnc2	Galnc1	-
4	JHD2	YJR119C	-	-	EtOHcl1	-	Galcl1	YPDnc1	-	-	-	-	EtOHnc2	-	-
4		YGL041C-B	-	YPDcl3	EtOHcl1	EtOHcl2	Galcl1	-	-	-	-	-	-	-	-
4		YNR064C	-	-	-	-	-	-	-	-	-	EtOHnc1	EtOHnc2	Galnc1	Galnc2
4	GTR2	YGR163W	-	YPDcl3	-	EtOHcl2	Galcl1	-	-	-	YPDnc4	-	-	-	-
4	RPL34B	YIL052C	-	-	EtOHcl1	-	-	-	YPDnc2	YPDnc3	-	-	-	-	Galnc2
4		YPR071W	-	-	EtOHcl1	-	Galcl1	-	YPDnc2	-	-	-	-	-	Galnc2
4	ADE13	YLR359W	YPDcl2	YPDcl3	-	EtOHcl2	Galcl1	-	-	-	-	-	-	-	-
4	PWP1	YLR196W	YPDcl2	YPDcl3	-	-	Galcl1	-	-	-	-	-	EtOHnc2	-	-
4	VMR1	YHL035C	-	YPDcl3	-	EtOHcl2	-	-	-	YPDnc3	-	-	-	-	Galnc2
4	MAK3	YPR051W	-	-	-	-	-	YPDnc1	-	-	-	EtOHnc1	-	Galnc1	Galnc2
4	COQ9	YLR201C	YPDcl2	-	EtOHcl1	-	Galcl1	-	-	YPDnc3	-	-	-	-	-
4		YDR537C	-	-	EtOHcl1	-	-	YPDnc1	YPDnc2	-	-	-	-	Galnc1	-
4	SMX2	YFL017W-A	-	-	EtOHcl1	-	-	YPDnc1	-	-	-	-	EtOHnc2	-	Galnc2
4		YEL018C-A	-	-	-	-	-	YPDnc1	-	YPDnc3	-	EtOHnc1	-	Galnc1	-
4	PMA1	YGL008C	-	-	-	-	-	YPDnc1	-	-	-	EtOHnc1	-	Galnc1	Galnc2
4	TGS1	YPL157W	-	-	EtOHcl1	-	-	YPDnc1	-	-	-	EtOHnc1	-	-	Galnc2
4	DON1	YDR273W	-	-	EtOHcl1	-	-	-	YPDnc2	YPDnc3	-	-	-	-	Galnc2
4	GPI13	YLL031C	YPDcl2	-	-	-	Galcl1	YPDnc1	YPDnc2	-	-	-	-	-	-
4		YPR142C	-	YPDcl3	-	-	Galcl1	-	-	-	-	EtOHnc1	-	Galnc1	-
4	HAS1	YMR290C	-	YPDcl3	EtOHcl1	-	Galcl1	-	-	-	-	-	-	-	Galnc2
4	LCB2	YDR062W	-	-	EtOHcl1	EtOHcl2	Galcl1	-	-	-	-	-	-	Galnc1	-
4	LCD1	YDR499W	-	YPDcl3	EtOHcl1	-	-	YPDnc1	-	-	-	-	-	-	Galnc2
4	FAR1	YJL157C	-	-	-	-	-	YPDnc1	-	YPDnc3	-	-	-	Galnc1	Galnc2
4	LIP1	YMR298W	YPDcl2	-	-	-	Galcl1	-	-	-	YPDnc4	-	-	Galnc1	-
4	FAF1	YIL019W	-	-	-	EtOHcl2	Galcl1	-	-	-	-	EtOHnc1	-	-	Galnc2
4	MRPL51	YPR100W	-	-	EtOHcl1	EtOHcl2	Galcl1	-	-	-	-	EtOHnc1	-	-	-
4		YDR095C	-	-	-	-	Galcl1	-	-	-	-	-	EtOHnc2	Galnc1	Galnc2
4		YPR036W-A	-	-	EtOHcl1	-	-	-	-	-	-	EtOHnc1	EtOHnc2	-	Galnc2
4	RAD55	YDR076W	-	YPDcl3	EtOHcl1	-	-	YPDnc1	-	-	-	-	-	-	Galnc2
4	CBS1	YDL069C	YPDcl2	-	EtOHcl1	-	Galcl1	-	-	-	-	-	-	Galnc1	-

4	CPT1	YNL130C	YPDcl2	-	-	EtOHcl2	Galcl1	-	-	-	YPDnc4	-	-	-	-
4		YDR381C-A	-	-	-	-	Galcl1	YPDnc1	-	-	-	EtOHnc1	EtOHnc2	-	-
4		YFR032C-B	-	-	EtOHcl1	-	-	-	-	YPDnc3	-	-	EtOHnc2	-	Galnc2
4	ELO1	YJL196C	YPDcl2	-	-	-	Galcl1	YPDnc1	-	-	-	EtOHnc1	-	-	-
4	THO1	YER063W	-	-	-	-	-	YPDnc1	-	-	-	EtOHnc1	-	Galnc1	Galnc2
4	MMS1	YPR164W	-	-	EtOHcl1	-	-	YPDnc1	-	-	-	EtOHnc1	-	-	Galnc2
4		YLR280C	-	-	-	-	-	YPDnc1	-	-	-	EtOHnc1	EtOHnc2	Galnc1	-
4	IRC16	YPR038W	YPDcl2	-	EtOHcl1	-	-	-	-	-	-	EtOHnc1	EtOHnc2	-	-
4	MRL1	YPR079W	-	YPDcl3	-	-	Galcl1	-	-	-	-	EtOHnc1	-	Galnc1	-
4	ITC1	YGL133W	-	-	-	-	-	-	YPDnc2	YPDnc3	-	EtOHnc1	-	-	Galnc2
4	BNA2	YJR078W	-	-	-	-	-	YPDnc1	-	-	-	EtOHnc1	-	Galnc1	Galnc2
4	DMC1	YER179W	-	-	-	-	-	YPDnc1	-	-	YPDnc4	EtOHnc1	-	-	Galnc2
4	RPL31B	YLR406C	-	-	-	EtOHcl2	Galcl1	-	-	-	-	-	EtOHnc2	Galnc1	-
4	RPL7B	YPL198W	-	-	-	EtOHcl2	Galcl1	-	-	-	YPDnc4	-	EtOHnc2	-	-
4	UTP8	YGR128C	-	-	-	-	Galcl1	-	YPDnc2	-	-	EtOHnc1	-	Galnc1	-
4	GYL1	YMR192W	YPDcl2	-	EtOHcl1	-	Galcl1	-	-	-	YPDnc4	-	-	-	-
4	ILV3	YJR016C	-	-	EtOHcl1	-	-	-	-	-	-	EtOHnc1	-	Galnc1	Galnc2
4	RPL29	YFR032C-A	-	-	EtOHcl1	-	Galcl1	YPDnc1	-	-	-	-	-	-	Galnc2
4		YGR025W	-	-	-	-	Galcl1	-	YPDnc2	-	YPDnc4	-	-	Galnc1	-
4		YAL045C	-	-	-	EtOHcl2	Galcl1	-	-	-	-	EtOHnc1	-	-	Galnc2
4	RRP17	YDR412W	-	-	-	-	Galcl1	YPDnc1	-	-	-	-	EtOHnc2	-	Galnc2
4	RPC34	YNR003C	-	-	-	-	Galcl1	-	YPDnc2	-	-	-	-	Galnc1	Galnc2
4	LSM8	YJR022W	-	YPDcl3	-	-	Galcl1	-	-	YPDnc3	-	-	-	-	Galnc2
4		YER188W	-	-	-	-	-	YPDnc1	-	-	-	EtOHnc1	-	Galnc1	Galnc2
4	RPL13B	YMR142C	YPDcl2	-	-	-	Galcl1	YPDnc1	-	-	-	-	-	Galnc1	-
4	NPL3	YDR432W	YPDcl2	-	EtOHcl1	-	Galcl1	-	-	-	YPDnc4	-	-	-	-
4	SEC10	YLR166C	YPDcl2	-	EtOHcl1	-	Galcl1	-	-	YPDnc3	-	-	-	-	-
4	KIN4	YOR233W	-	YPDcl3	EtOHcl1	-	Galcl1	YPDnc1	-	-	-	-	-	-	-
4		YCR102W-/	-	-	EtOHcl1	-	-	-	-	-	YPDnc4	-	-	Galnc1	Galnc2
4	AMA1	YGR225W	-	-	-	-	Galcl1	-	-	-	YPDnc4	EtOHnc1	-	Galnc1	-
4	RNR1	YER070W	-	-	-	-	-	YPDnc1	-	-	YPDnc4	-	-	Galnc1	Galnc2
4		YOR019W	-	-	-	-	Galcl1	-	-	-	-	EtOHnc1	EtOHnc2	-	Galnc2
4	RCR2	YDR003W	-	-	-	-	-	YPDnc1	-	-	-	EtOHnc1	-	Galnc1	Galnc2
4		YKL066W	YPDcl2	YPDcl3	-	-	-	-	-	YPDnc3	YPDnc4	-	-	-	-

4	GLG1	YKR058W	YPDcl2	-	-	EtOHcl2	Galcl1	-	YPDnc2	-	-	-	-	-	-
4	TIM21	YGR033C	-	-	EtOHcl1	-	-	YPDnc1	-	-	-	-	EtOHnc2	Galnc1	-
4		YOR314W-.	-	-	-	-	Galcl1	YPDnc1	-	-	-	EtOHnc1	-	Galnc1	-
4	IME4	YGL192W	YPDcl2	YPDcl3	-	-	Galcl1	YPDnc1	-	-	-	-	-	-	-
4		YBR056W-/	YPDcl2	-	EtOHcl1	-	Galcl1	-	-	-	-	-	-	-	Galnc2
4		YOR248W	YPDcl2	-	EtOHcl1	-	-	-	-	-	-	EtOHnc1	-	Galnc1	-
4	YSA1	YBR111C	-	-	-	-	-	-	-	-	-	EtOHnc1	EtOHnc2	Galnc1	Galnc2
4		YLR149C	-	-	EtOHcl1	-	-	-	-	-	-	EtOHnc1	-	Galnc1	Galnc2
4	DLD3	YEL071W	YPDcl2	-	-	-	-	-	YPDnc2	YPDnc3	YPDnc4	-	-	-	-
4	TIM12	YBR091C	-	-	-	-	-	YPDnc1	YPDnc2	-	-	-	EtOHnc2	Galnc1	-
4		YGL042C	-	YPDcl3	EtOHcl1	EtOHcl2	Galcl1	-	-	-	-	-	-	-	-
4	RED1	YLR263W	-	-	EtOHcl1	-	Galcl1	-	YPDnc2	-	-	-	-	Galnc1	-
4	GND1	YHR183W	YPDcl2	YPDcl3	-	-	Galcl1	-	-	YPDnc3	-	-	-	-	-
4	FLX1	YIL134W	-	-	EtOHcl1	-	Galcl1	YPDnc1	-	-	-	EtOHnc1	-	-	-
4	CUE3	YGL110C	-	-	-	-	Galcl1	-	YPDnc2	YPDnc3	YPDnc4	-	-	-	-
4		YOL037C	-	-	-	-	-	YPDnc1	-	-	-	EtOHnc1	-	Galnc1	Galnc2
4		YLR339C	-	-	-	EtOHcl2	Galcl1	-	-	-	-	EtOHnc1	-	Galnc1	-
4		YGL239C	-	-	-	EtOHcl2	-	-	YPDnc2	-	-	EtOHnc1	-	Galnc1	-
4	SPP381	YBR152W	-	-	-	-	Galcl1	YPDnc1	-	-	-	-	-	Galnc1	Galnc2
4	DEF1	YKL054C	-	-	EtOHcl1	-	-	YPDnc1	-	-	-	EtOHnc1	-	-	Galnc2
3	TIM17	YJL143W	-	-	EtOHcl1	EtOHcl2	-	-	-	-	-	-	-	Galnc1	-
3	AHP1	YLR109W	-	-	EtOHcl1	-	-	YPDnc1	-	-	-	EtOHnc1	-	-	-
3	TAD3	YLR316C	YPDcl2	-	EtOHcl1	-	Galcl1	-	-	-	-	-	-	-	-
3		YEL008W	YPDcl2	-	EtOHcl1	-	-	-	-	-	-	-	EtOHnc2	-	-
3	EHT1	YBR177C	YPDcl2	-	EtOHcl1	-	Galcl1	-	-	-	-	-	-	-	-
3		YOL107W	-	-	EtOHcl1	EtOHcl2	-	-	YPDnc2	-	-	-	-	-	-
3	BOI2	YER114C	-	-	-	-	Galcl1	-	-	-	-	EtOHnc1	-	Galnc1	-
3	YET3	YDL072C	YPDcl2	-	-	-	Galcl1	-	-	-	-	-	-	Galnc1	-
3	MTR4	YJL050W	-	YPDcl3	-	-	Galcl1	-	-	-	YPDnc4	-	-	-	-
3	NUP53	YMR153W	-	-	EtOHcl1	-	Galcl1	-	-	-	-	-	EtOHnc2	-	-
3	ARX1	YDR101C	YPDcl2	-	EtOHcl1	-	Galcl1	-	-	-	-	-	-	-	-
3	ADH7	YCR105W	-	-	-	EtOHcl2	-	-	-	-	-	-	-	Galnc1	Galnc2
3	HEM12	YDR047W	YPDcl2	-	-	EtOHcl2	Galcl1	-	-	-	-	-	-	-	-
3	SPC97	YHR172W	-	-	EtOHcl1	-	-	YPDnc1	-	-	-	-	-	-	Galnc2

3		YMR185W	-	-	EtOHcl1	-	Galcl1	-	-	-	YPDnc4	-	-	-	-
3	VMA13	YPR036W	-	-	EtOHcl1	-	Galcl1	-	-	YPDnc3	-	-	-	-	-
3	VPS63	YLR261C	YPDcl2	-	-	EtOHcl2	Galcl1	-	-	-	-	-	-	-	-
3	FKS3	YMR306W	-	-	EtOHcl1	-	-	-	-	-	-	-	-	Galnc1	Galnc2
3	CDC55	YGL190C	YPDcl2	-	EtOHcl1	-	-	-	-	-	-	-	-	-	Galnc2
3	TOS4	YLR183C	-	-	-	-	-	-	-	YPDnc3	YPDnc4	-	-	-	Galnc2
3	PRS1	YKL181W	YPDcl2	-	-	-	-	-	-	YPDnc3	-	-	-	-	Galnc2
3	ETR1	YBR026C	-	YPDcl3	-	-	Galcl1	-	-	-	-	-	-	Galnc1	-
3		YDR509W	-	-	EtOHcl1	-	-	YPDnc1	-	-	-	EtOHnc1	-	-	-
3	RPL20B	YOR312C	YPDcl2	-	-	-	-	YPDnc1	-	-	YPDnc4	-	-	-	-
3	ARR1	YPR199C	-	-	-	-	Galcl1	YPDnc1	-	YPDnc3	-	-	-	-	-
3	ALD5	YER073W	-	YPDcl3	-	-	Galcl1	-	-	YPDnc3	-	-	-	-	-
3		YLR415C	-	-	-	-	Galcl1	YPDnc1	-	-	YPDnc4	-	-	-	-
3	EMI1	YDR512C	-	-	-	-	Galcl1	YPDnc1	-	-	-	EtOHnc1	-	-	-
3	HSL1	YKL101W	-	YPDcl3	-	-	-	-	-	-	YPDnc4	EtOHnc1	-	-	-
3	NCE102	YPR149W	-	-	EtOHcl1	-	-	YPDnc1	-	-	-	EtOHnc1	-	-	-
3	SKI2	YLR398C	-	-	-	-	Galcl1	YPDnc1	-	-	-	-	-	-	Galnc2
3	PUT2	YHR037W	-	-	-	EtOHcl2	Galcl1	-	-	-	-	EtOHnc1	-	-	-
3	YAH1	YPL252C	-	-	-	-	Galcl1	-	-	-	YPDnc4	-	EtOHnc2	-	-
3		YLR285C-A	-	-	-	-	-	-	YPDnc2	-	YPDnc4	EtOHnc1	-	-	-
3	RTS1	YOR014W	YPDcl2	-	-	-	Galcl1	-	-	-	YPDnc4	-	-	-	-
3	PAU9	YBL108C-A	-	-	-	-	-	-	-	-	-	EtOHnc1	-	Galnc1	Galnc2
3		YMR086W	YPDcl2	-	EtOHcl1	-	Galcl1	-	-	-	-	-	-	-	-
3	FRS1	YLR060W	-	-	EtOHcl1	-	Galcl1	-	-	-	-	-	EtOHnc2	-	-
3		YGR031W	YPDcl2	-	-	-	Galcl1	-	-	-	-	-	-	-	Galnc2
3	APC4	YDR118W	YPDcl2	YPDcl3	-	-	Galcl1	-	-	-	-	-	-	-	-
3		YOL134C	-	-	-	EtOHcl2	-	-	-	-	-	EtOHnc1	-	Galnc1	-
3		YAL016C-A	-	-	-	-	-	YPDnc1	-	-	-	EtOHnc1	-	Galnc1	-
3	PPH21	YDL134C	YPDcl2	-	-	-	Galcl1	YPDnc1	-	-	-	-	-	-	-
3	SCD6	YPR129W	-	-	-	-	-	YPDnc1	-	YPDnc3	-	-	-	-	Galnc2
3	SNA3	YJL151C	YPDcl2	YPDcl3	-	-	Galcl1	-	-	-	-	-	-	-	-
3	ALF1	YNL148C	YPDcl2	-	-	EtOHcl2	Galcl1	-	-	-	-	-	-	-	-
3	RPL21A	YBR191W	YPDcl2	YPDcl3	-	-	Galcl1	-	-	-	-	-	-	-	-
3	CCW12	YLR110C	-	-	EtOHcl1	-	-	YPDnc1	-	-	-	EtOHnc1	-	-	-

3		YGR117C	YPDcl2	-	-	-	Galcl1	-	-	-	-	-	-	Galnc1	-
3	CNA1	YLR433C	YPDcl2	-	-	-	Galcl1	-	-	-	YPDnc4	-	-	-	-
3	TDA3	YHR009C	-	-	-	-	Galcl1	-	-	-	-	-	-	Galnc1	Galnc2
3		YBR126W-[illegible]	-	-	-	-	-	YPDnc1	YPDnc2	YPDnc3	-	-	-	-	-
3	RPN7	YPR108W	-	-	EtOHcl1	-	-	-	YPDnc2	-	-	-	-	Galnc1	-
3		YIR016W	-	-	EtOHcl1	-	-	-	YPDnc2	-	-	EtOHnc1	-	-	-
3	SRP102	YKL154W	-	-	EtOHcl1	-	-	-	-	YPDnc3	-	-	-	Galnc1	-
3	VPS5	YOR069W	-	-	-	-	Galcl1	-	-	YPDnc3	-	-	-	Galnc1	-
3	CIN2	YPL241C	YPDcl2	YPDcl3	-	-	Galcl1	-	-	-	-	-	-	-	-
3	SIP5	YMR140W	-	-	-	-	Galcl1	-	-	-	-	-	-	Galnc1	Galnc2
3		YNL120C	-	-	-	-	Galcl1	YPDnc1	-	-	-	-	-	-	Galnc2
3	PAU17	YLL025W	-	-	-	-	-	YPDnc1	-	-	-	-	EtOHnc2	Galnc1	-
3	IXR1	YKL032C	-	-	EtOHcl1	-	-	YPDnc1	-	-	-	-	-	-	Galnc2
3	FMP32	YFL046W	-	-	-	-	Galcl1	-	-	-	-	EtOHnc1	-	Galnc1	-
3	GDI1	YER136W	-	-	-	-	-	YPDnc1	-	-	-	EtOHnc1	-	Galnc1	-
3		YPR099C	-	-	EtOHcl1	EtOHcl2	Galcl1	-	-	-	-	-	-	-	-
3		YGR071C	YPDcl2	-	-	EtOHcl2	Galcl1	-	-	-	-	-	-	-	-
3	NUP170	YBL079W	YPDcl2	-	-	-	Galcl1	-	-	-	YPDnc4	-	-	-	-
3		YGL041C	-	-	-	-	-	-	YPDnc2	-	YPDnc4	-	-	-	Galnc2
3	NCP1	YHR042W	YPDcl2	-	-	-	-	-	YPDnc2	-	-	-	-	-	Galnc2
3		YLR366W	YPDcl2	YPDcl3	-	-	Galcl1	-	-	-	-	-	-	-	-
3		YBL039W-[illegible]	-	-	-	-	-	YPDnc1	-	-	-	EtOHnc1	-	Galnc1	-
3	ICY2	YPL250C	-	-	EtOHcl1	-	-	YPDnc1	-	-	-	-	-	-	Galnc2
3		YKR104W	-	-	-	-	-	-	-	-	YPDnc4	EtOHnc1	-	-	Galnc2
3	MDM35	YKL053C-A	-	-	EtOHcl1	-	-	YPDnc1	-	-	-	EtOHnc1	-	-	-
3		YDL187C	-	-	-	-	-	YPDnc1	-	-	-	EtOHnc1	-	Galnc1	-
3		YLR224W	YPDcl2	-	-	-	Galcl1	-	-	YPDnc3	-	-	-	-	-
3	ARF2	YDL137W	YPDcl2	-	EtOHcl1	-	-	-	-	-	-	-	-	-	Galnc2
3	RPA190	YOR341W	-	-	EtOHcl1	-	-	YPDnc1	-	-	YPDnc4	-	-	-	-
2	CUP1-2	YHR055C	-	-	-	EtOHcl2	-	-	-	-	-	-	EtOHnc2	-	-
2	PHO12	YHR215W	YPDcl2	-	-	-	Galcl1	-	-	-	-	-	-	-	-
2		YBR063C	-	-	-	-	Galcl1	-	-	-	YPDnc4	-	-	-	-
2	RHR2	YIL053W	-	-	-	-	-	-	-	YPDnc3	-	-	-	-	Galnc2
2	DOC1	YGL240W	-	-	-	-	-	-	-	-	-	-	-	Galnc1	Galnc2

2		YOR186W	-	-	-	-	Galcl1	-	-	-	-	-	-	-	Galnc2
2	PUS2	YGL063W	-	-	EtOHcl1	-	-	-	-	YPDnc3	-	-	-	-	-
2	HEF3	YNL014W	YPDcl2	-	-	-	Galcl1	-	-	-	-	-	-	-	-
2		YBR190W	-	-	EtOHcl1	-	Galcl1	-	-	-	-	-	-	-	-
2	SNA2	YDR525W-/	-	-	-	-	Galcl1	-	-	-	-	-	-	Galnc1	-
2	RDI1	YDL135C	YPDcl2	-	-	-	Galcl1	-	-	-	-	-	-	-	-
2		YFR010W-/	-	YPDcl3	-	-	Galcl1	-	-	-	-	-	-	-	-
2	PSK1	YAL017W	-	-	EtOHcl1	-	Galcl1	-	-	-	-	-	-	-	-
2		YDR524W-(	-	-	-	-	-	-	-	-	-	EtOHnc1	-	Galnc1	-
2	RPS30B	YOR182C	-	-	EtOHcl1	-	-	-	-	-	-	-	-	-	Galnc2
2		YFL032W	-	-	-	-	-	-	-	-	-	EtOHnc1	-	Galnc1	-
2		YGR068W-/	-	-	-	-	-	-	-	YPDnc3	-	EtOHnc1	-	-	-
2	SPA2	YLL021W	-	-	-	-	-	YPDnc1	-	YPDnc3	-	-	-	-	-
2	ARO10	YDR380W	-	-	EtOHcl1	-	-	-	-	-	-	-	-	Galnc1	-
2	SEC59	YMR013C	-	-	-	-	Galcl1	YPDnc1	-	-	-	-	-	-	-
2		YMR306C-/	-	-	-	-	-	-	-	-	-	-	-	Galnc1	Galnc2
2	FCY21	YER060W	-	-	EtOHcl1	-	-	-	-	-	-	EtOHnc1	-	-	-
2	SMP1	YBR182C	-	YPDcl3	-	-	Galcl1	-	-	-	-	-	-	-	-
2		YHL048C-A	-	-	EtOHcl1	-	Galcl1	-	-	-	-	-	-	-	-
2	AAH1	YNL141W	YPDcl2	-	-	-	-	-	-	YPDnc3	-	-	-	-	-
2		YHR214C-E	YPDcl2	-	-	-	Galcl1	-	-	-	-	-	-	-	-
2	GAS1	YMR307W	-	-	-	-	-	-	-	-	-	EtOHnc1	-	-	Galnc2
2	ARO2	YGL148W	-	-	-	-	-	-	YPDnc2	-	YPDnc4	-	-	-	-
2	FMP16	YDR070C	-	-	-	-	-	YPDnc1	-	-	-	-	-	Galnc1	-
2		YDR524C-A	-	-	EtOHcl1	-	-	-	-	-	-	-	-	-	Galnc2
2		YCR100C	-	YPDcl3	EtOHcl1	-	-	-	-	-	-	-	-	-	-
2	ASI1	YMR119W	-	-	-	-	-	YPDnc1	-	-	-	EtOHnc1	-	-	-
2		YNL114C	-	-	-	-	-	-	-	-	-	EtOHnc1	EtOHnc2	-	-
2	RPL12A	YEL054C	-	-	-	-	-	-	-	-	-	EtOHnc1	-	Galnc1	-
2		YDR524C-B	-	-	-	-	-	-	-	-	-	EtOHnc1	-	Galnc1	-
2	RPS15	YOL040C	-	-	EtOHcl1	-	-	-	-	-	-	-	-	-	Galnc2
2		YGR122W	-	-	EtOHcl1	EtOHcl2	-	-	-	-	-	-	-	-	-
2		YJR154W	-	-	-	-	-	-	-	-	-	EtOHnc1	-	-	Galnc2
2		YBR012C	YPDcl2	-	-	-	Galcl1	-	-	-	-	-	-	-	-

2		YGR125W	-	-	-	-	-	-	-	-	YPDnc4	-	-	Galnc1	-
2		YER066C-A	-	-	-	-	-	-	YPDnc2	-	YPDnc4	-	-	-	-
2	PMP1	YCR024C-A	-	-	EtOHcl1	-	-	YPDnc1	-	-	-	-	-	-	-
2	BEM1	YBR200W	-	-	-	-	-	-	-	-	-	EtOHnc1	-	Galnc1	-
2	JJJ1	YNL227C	-	-	-	EtOHcl2	Galcl1	-	-	-	-	-	-	-	-
2	SUR1	YPL057C	-	-	EtOHcl1	-	-	-	-	-	-	-	-	-	Galnc2
2	CBP4	YGR174C	-	-	EtOHcl1	-	Galcl1	-	-	-	-	-	-	-	-
2	PDI1	YCL043C	-	-	EtOHcl1	EtOHcl2	-	-	-	-	-	-	-	-	-
2		YOL014W	-	-	EtOHcl1	-	-	-	-	YPDnc3	-	-	-	-	-
2	PUF4	YGL014W	-	-	EtOHcl1	-	-	-	-	-	YPDnc4	-	-	-	-
2	LEU9	YOR108W	-	-	-	-	Galcl1	-	-	-	YPDnc4	-	-	-	-
2	GNP1	YDR508C	-	-	EtOHcl1	-	-	YPDnc1	-	-	-	-	-	-	-
2		YMR193C-/	-	-	-	EtOHcl2	-	-	-	-	-	EtOHnc1	-	-	-
2	CUP2	YGL166W	YPDcl2	YPDcl3	-	-	-	-	-	-	-	-	-	-	-
2	AIM9	YER080W	-	-	EtOHcl1	-	-	YPDnc1	-	-	-	-	-	-	-
2	PRP21	YJL203W	YPDcl2	-	-	-	Galcl1	-	-	-	-	-	-	-	-
2		YIL021C-A	-	-	-	EtOHcl2	-	-	-	-	-	-	-	Galnc1	-
2	ABF1	YKL112W	-	-	-	-	-	-	-	-	YPDnc4	-	-	-	Galnc2
2		YBL073W	-	-	-	EtOHcl2	Galcl1	-	-	-	-	-	-	-	-
2		YAL066W	-	-	-	-	Galcl1	-	-	-	-	-	EtOHnc2	-	-
2	HYP2	YEL034W	-	-	-	-	-	-	-	-	-	EtOHnc1	-	-	Galnc2
2	PGA1	YNL158W	YPDcl2	-	-	-	-	-	-	-	-	-	-	-	Galnc2
2	NHP6B	YBR089C-A	-	-	EtOHcl1	-	-	-	-	-	-	EtOHnc1	-	-	-
2	ENT5	YDR153C	-	-	EtOHcl1	-	-	YPDnc1	-	-	-	-	-	-	-
2	LSM7	YNL147W	-	-	-	-	Galcl1	-	-	-	-	-	-	Galnc1	-
1	PMT1	YDL095W	-	-	-	-	Galcl1	-	-	-	-	-	-	-	-
1		YDL071C	-	-	-	-	-	-	-	-	-	-	-	Galnc1	-
1	NAM8	YHR086W	-	YPDcl3	-	-	-	-	-	-	-	-	-	-	-
1	GAB1	YLR459W	-	-	-	-	Galcl1	-	-	-	-	-	-	-	-
1	RPL13A	YDL082W	-	-	-	-	-	-	-	-	-	EtOHnc1	-	-	-
1	RPL36A	YMR194W	-	-	-	-	-	-	-	-	-	EtOHnc1	-	-	-
1		YNL018C	-	-	-	-	Galcl1	-	-	-	-	-	-	-	-
1	MRS2	YOR334W	-	-	-	-	-	-	YPDnc2	-	-	-	-	-	-
1		YPL080C	-	-	EtOHcl1	-	-	-	-	-	-	-	-	-	-

1	URC2	YDR520C	-	-	-	-	Galcl1	-	-	-	-	-	-	-	-
1	SNT309	YPR101W	-	-	EtOHcl1	-	-	-	-	-	-	-	-	-	-
1		YMR172C-	-	-	EtOHcl1	-	-	-	-	-	-	-	-	-	-
1	VPS53	YJL029C	-	-	-	-	-	-	-	-	-	EtOHnc1	-	-	-
1	OLE1	YGL055W	-	-	-	-	Galcl1	-	-	-	-	-	-	-	-
1		YER147C-A	-	-	-	-	-	-	-	-	-	-	-	Galnc1	-
1	AGE1	YDR524C	-	-	-	-	-	-	-	-	-	EtOHnc1	-	-	-
1	POC4	YPL144W	-	-	EtOHcl1	-	-	-	-	-	-	-	-	-	-
1	NMD4	YLR363C	-	-	-	-	Galcl1	-	-	-	-	-	-	-	-
1	RFC1	YOR217W	-	-	-	-	-	-	-	-	-	-	-	Galnc1	-
1	SPF1	YEL031W	-	-	-	-	Galcl1	-	-	-	-	-	-	-	-
1		YDL086W	-	-	-	-	-	-	-	-	-	-	-	-	Galnc2
1	TEF2	YBR118W	-	-	-	-	-	YPDnc1	-	-	-	-	-	-	-
1	VPS16	YPL045W	-	-	-	-	Galcl1	-	-	-	-	-	-	-	-
1	CCC2	YDR270W	-	-	-	-	Galcl1	-	-	-	-	-	-	-	-
1	RPS24A	YER074W	-	-	-	-	Galcl1	-	-	-	-	-	-	-	-
1	MET6	YER091C	-	-	EtOHcl1	-	-	-	-	-	-	-	-	-	-
1		YPR050C	-	-	-	-	-	-	-	-	YPDnc4	-	-	-	-
1	COQ1	YBR003W	-	-	-	-	Galcl1	-	-	-	-	-	-	-	-
1		YLR225C	-	-	-	-	Galcl1	-	-	-	-	-	-	-	-
1	NET1	YJL076W	-	-	-	-	-	-	YPDnc2	-	-	-	-	-	-
1	RDS1	YCR106W	-	-	-	-	-	-	-	-	-	-	-	-	Galnc2
1	RPS25B	YLR333C	-	-	EtOHcl1	-	-	-	-	-	-	-	-	-	-
1	HOT1	YMR172W	-	-	EtOHcl1	-	-	-	-	-	-	-	-	-	-
1		YDL152W	-	-	-	-	Galcl1	-	-	-	-	-	-	-	-
1	UBP6	YFR010W	-	-	-	-	Galcl1	-	-	-	-	-	-	-	-
1	BDH1	YAL060W	-	-	-	-	-	-	-	-	-	-	-	-	Galnc2
1	STL1	YDR536W	-	-	-	-	Galcl1	-	-	-	-	-	-	-	-
1	SMF2	YHR050W	-	-	EtOHcl1	-	-	-	-	-	-	-	-	-	-
1	UPS2	YLR168C	-	-	-	-	-	YPDnc1	-	-	-	-	-	-	-
1	UBP12	YJL197W	-	-	-	-	-	-	-	-	-	-	-	Galnc1	-
1	MMS4	YBR098W	-	-	-	-	Galcl1	-	-	-	-	-	-	-	-
1		YBR225W	-	-	-	-	-	-	-	YPDnc3	-	-	-	-	-
1		YDL159C-B	-	-	-	EtOHcl2	-	-	-	-	-	-	-	-	-

1	APC1	YNL172W	YPDcl2	-	-	-	-	-	-	-	-	-	-	-	-
1	CDC1	YDR182W	-	-	-	-	-	-	-	-	-	-	-	Galnc1	-
1	DIE2	YGR227W	-	-	-	-	-	-	-	-	-	EtOHnc1	-	-	-
1		YDL063C	-	-	-	-	-	-	YPDnc2	-	-	-	-	-	-
1	FCF1	YDR339C	-	-	-	EtOHcl2	-	-	-	-	-	-	-	-	-
1		YPL035C	-	-	-	-	Galcl1	-	-	-	-	-	-	-	-
1	GCY1	YOR120W	-	-	-	-	-	-	-	-	-	-	-	Galnc1	-
1		YGL188C-A	-	-	-	-	-	YPDnc1	-	-	-	-	-	-	-
1	HHF1	YBR009C	-	-	EtOHcl1	-	-	-	-	-	-	-	-	-	-
1	DUS4	YLR405W	-	-	-	-	-	-	-	-	-	EtOHnc1	-	-	-
1	GCN20	YFR009W	-	-	-	-	Galcl1	-	-	-	-	-	-	-	-
1		YNL171C	YPDcl2	-	-	-	-	-	-	-	-	-	-	-	-
1	OPI9	YLR338W	-	-	EtOHcl1	-	-	-	-	-	-	-	-	-	-
1	RPS26A	YGL189C	-	-	-	-	-	YPDnc1	-	-	-	-	-	-	-
1	VMA8	YEL051W	-	-	-	-	-	-	-	-	-	-	-	Galnc1	-
1	ISC10	YER180C	-	-	-	-	-	-	-	-	-	EtOHnc1	-	-	-
1	NOP13	YNL175C	-	-	-	-	Galcl1	-	-	-	-	-	-	-	-
1	THI12	YNL332W	-	-	EtOHcl1	-	-	-	-	-	-	-	-	-	-
1		YER145C-A	-	-	-	-	-	-	-	-	-	-	-	Galnc1	-
1	TRM5	YHR070W	-	-	EtOHcl1	-	-	-	-	-	-	-	-	-	-
1	PRE6	YOL038W	-	-	-	-	-	-	-	-	-	-	EtOHnc2	-	-
